# Risk phenotypes of diabetes and association with COVID-19 severity and death: an update of a living systematic review and meta-analysis

**DOI:** 10.1007/s00125-023-05928-1

**Published:** 2023-05-19

**Authors:** Sabrina Schlesinger, Alexander Lang, Nikoletta Christodoulou, Philipp Linnerz, Kalliopi Pafili, Oliver Kuss, Christian Herder, Manuela Neuenschwander, Janett Barbaresko, Michael Roden

**Affiliations:** 1 Institute for Biometrics and Epidemiology, German Diabetes Center, Leibniz Center for Diabetes Research at Heinrich Heine University Düsseldorf, Düsseldorf, Germany; 2 German Center for Diabetes Research (DZD), Partner Düsseldorf, München-Neuherberg, Germany; 3 Institute for Clinical Diabetology, German Diabetes Center, Leibniz Center for Diabetes Research at Heinrich Heine University Düsseldorf, Düsseldorf, Germany; 4 Centre for Health and Society, Faculty of Medicine, Heinrich Heine University Düsseldorf, Düsseldorf, Germany; 5 Department of Endocrinology and Diabetology, Medical Faculty and University Hospital Düsseldorf, Heinrich Heine University Düsseldorf, Düsseldorf, Germany

**Keywords:** COVID-19, Diabetes, Meta-analysis, SARS-CoV-2, Systematic review

## Abstract

**Aims/hypothesis:**

To provide a systematic overview of the current body of evidence on high-risk phenotypes of diabetes associated with COVID-19 severity and death.

**Methods:**

This is the first update of our recently published living systematic review and meta-analysis. Observational studies investigating phenotypes in individuals with diabetes and confirmed SARS-CoV-2 infection with regard to COVID-19-related death and severity were included. The literature search was conducted from inception up to 14 February 2022 in PubMed, Epistemonikos, Web of Science and the COVID-19 Research Database and updated using PubMed alert to 1 December 2022. A random-effects meta-analysis was used to calculate summary relative risks (SRRs) with 95% CIs. The risk of bias was evaluated using the Quality in Prognosis Studies (QUIPS) tool and the certainty of evidence using the GRADE approach.

**Results:**

A total of 169 articles (147 new studies) based on approximately 900,000 individuals were included. We conducted 177 meta-analyses (83 on COVID-19-related death and 94 on COVID-19 severity). Certainty of evidence was strengthened for associations between male sex, older age, blood glucose level at admission, chronic insulin use, chronic metformin use (inversely) and pre-existing comorbidities (CVD, chronic kidney disease, chronic obstructive pulmonary disease) and COVID-19-related death. New evidence with moderate to high certainty emerged for the association between obesity (SRR [95% CI] 1.18 [1.04, 1.34], *n*=21 studies), HbA_1c_ (53–75 mmol/mol [7–9%]: 1.18 [1.06, 1.32], *n*=8), chronic glucagon-like peptide-1 receptor agonist use (0.83 [0.71, 0.97], *n*=9), pre-existing heart failure (1.33 [1.21, 1.47], *n*=14), pre-existing liver disease (1.40 [1.17, 1.67], *n*=6), the Charlson index (per 1 unit increase: 1.33 [1.13, 1.57], *n*=2), high levels of C-reactive protein (per 5 mg/l increase: 1.07 [1.02, 1.12], *n*=10), aspartate aminotransferase level (per 5 U/l increase: 1.28 [1.06, 1.54], *n*=5), eGFR (per 10 ml/min per 1.73 m^2^ increase: 0.80 [0.71, 0.90], *n*=6), lactate dehydrogenase level (per 10 U/l increase: 1.03 [1.01, 1.04], *n*=7) and lymphocyte count (per 1×10^9^/l increase: 0.59 [0.40, 0.86], *n*=6) and COVID-19-related death. Similar associations were observed between risk phenotypes of diabetes and severity of COVID-19, with some new evidence on existing COVID-19  vaccination status (0.32 [0.26, 0.38], *n*=3), pre-existing hypertension (1.23 [1.14, 1.33], *n*=49), neuropathy and cancer, and high IL-6 levels. A limitation of this study is that the included studies are observational in nature and residual or unmeasured confounding cannot be ruled out.

**Conclusions/interpretation:**

Individuals with a more severe course of diabetes and pre-existing comorbidities had a poorer prognosis of COVID-19 than individuals with a milder course of the disease.

**Registration:**

PROSPERO registration no. CRD42020193692.

**Previous version:**

This is a living systematic review and meta-analysis. The previous version can be found at https://link.springer.com/article/10.1007/s00125-021-05458-8

**Funding:**

The German Diabetes Center (DDZ) is funded by the German Federal Ministry of Health and the Ministry of Culture and Science of the State North Rhine-Westphalia. This study was supported in part by a grant from the German Federal Ministry of Education and Research to the German Center for Diabetes Research (DZD).

**Graphical Abstract:**

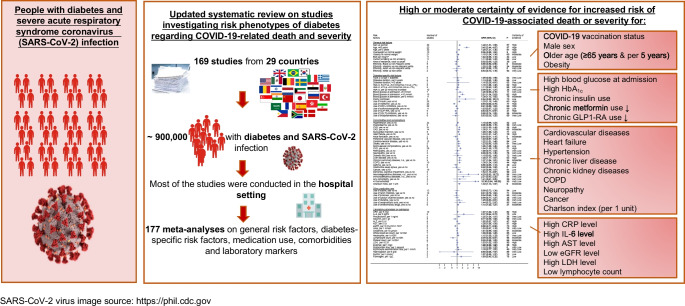

**Supplementary Information:**

The online version contains peer-reviewed but unedited supplementary material available at 10.1007/s00125-023-05928-1.


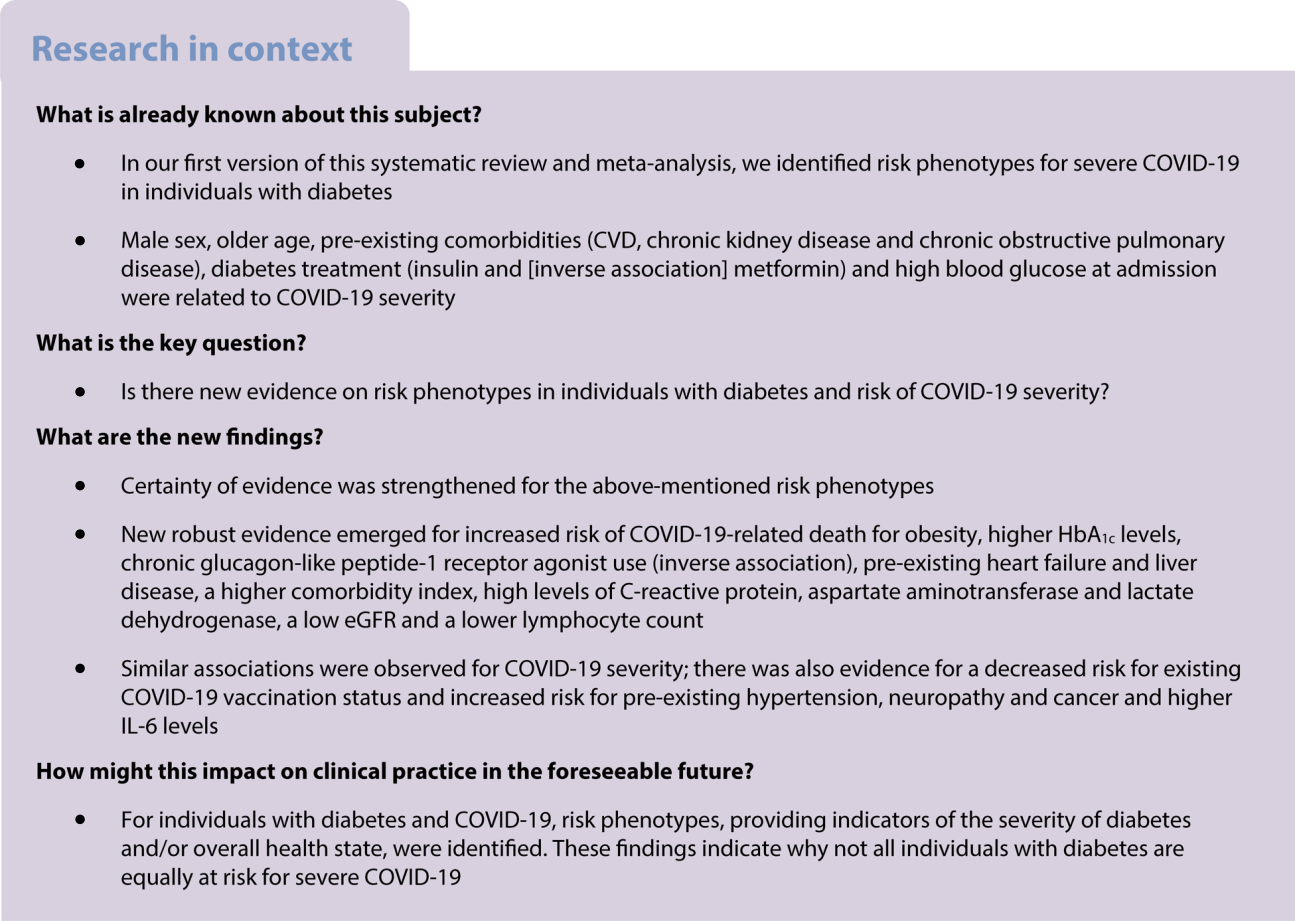


## Introduction

In our recent living systematic review and meta-analysis, we identified several risk phenotypes for severe acute respiratory syndrome coronavirus-2 (SARS-CoV-2) infection in individuals with diabetes, based on 22 studies [1]. There was moderate to high certainty of evidence that male sex, older age (≥65 years), pre-existing CVD, chronic kidney disease (CKD) and chronic obstructive pulmonary disease (COPD), diabetes treatment (insulin and [inverse association] metformin) and high blood glucose level at admission were associated with COVID-19-related death or disease severity. Since then, numerous studies on this topic have been published and thus new evidence is available. To provide the best current body of evidence, our aim was to update the living systematic review and meta-analysis on associations between risk phenotypes of diabetes and confirmed SARS-CoV-2 infection associated with COVID-19-related death and severity.

## Methods

This is the first update of our living systematic review and meta-analysis and the methods are described in detail in our previous study [1]. The update was conducted and reported according to the Preferred Reporting Items for Systematic Reviews and Meta-Analyses (PRISMA) statement [2].

### Search strategy and selection criteria

The systematic literature search was updated to 14 February 2022, using the same search terms and databases (PubMed, Epistemonikos, Web of Science and the COVID-19 Research Database) as in the original study (see electronic supplementary material [ESM] Table 1). From 15 February 2022 until 1 December 2022, we used only the PubMed alert based on our search terms because 96% of the relevant studies up to 14 February 2022 were identified in PubMed and thus the inclusion of the further databases did not justify the additional work and expense.

We included studies of any design that reported risk estimates (HRs, RRs or ORs with 95% CIs) for associations between phenotypes (general characteristics of participants, diabetes-specific characteristics, presence of diabetes-related complications and underlying comorbidities, chronic medication use and laboratory variables) and COVID-19-related death and severity of COVID-19 in individuals with diabetes and WHO-defined confirmed SARS-CoV-2 infection (https://apps.who.int/iris/handle/10665/337834). We excluded studies without primary clinical data (e.g. modelling studies), editorials, letters without primary data, commentaries, reviews, articles not in English and guidelines. Studies that focused on mixed populations, including individuals without diabetes or without COVID-19, were also excluded. If articles were based on the same cohort/data, we selected the study with the largest number of cases. If the studies were based on the same number of cases, we selected the study with the more favourable adjustment set. We contacted study authors for missing data, to query implausible data or for further information if needed [3–10].

### Data extraction and risk of bias assessment

Study selection, data extraction (ESM Table 2), assessment of risk of bias using the Quality in Prognosis Studies (QUIPS) tool [11] (ESM Methods; ESM Table 3) and assessment of certainty of evidence using the GRADEpro approach [12] were conducted independently by two investigators and, if necessary, a third investigator was consulted and consensus was reached through discussion.

### Statistical analysis

Summary relative risks (SRRs) and 95% CIs were calculated by random-effects meta-analysis using the DerSimonian and Laird method. The data from the original systematic review and meta-analysis [1] were combined with the findings from the new studies. We followed our original analysis plan and calculated *I*^2^ as a measure of heterogeneity, assessed publication bias by generating funnel plots and applying Egger’s test, and stratified our meta-analyses by risk of bias due to confounding (low/moderate risk vs high risk of bias). All meta-analyses were conducted for COVID-19-related death and severity (defined as a composite endpoint including death, tracheal intubation for mechanical ventilation, acute respiratory distress syndrome, septic shock, intensive care unit admission, multiple organ dysfunction or failure, or hospital admission). We conducted sensitivity analyses by calculating 95% CIs using the Hartung–Knapp–Sidik–Jonkman method, which provides more adequate error rates than the DerSimonian and Laird method, particularly for meta-analyses based on small numbers of studies. All statistical analyses were conducted with Stata software version 15.1 (Stata Corporation, USA).

## Results

### Literature search and characteristics of included studies

**Fig. 1:**
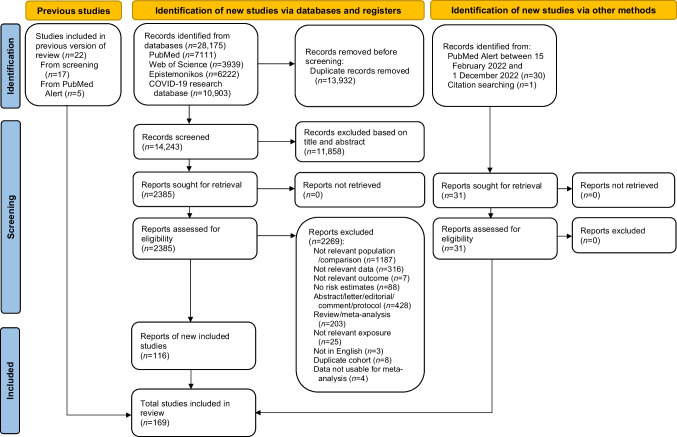
Flow chart of the literature search. Based on Page et al [2]. For more information, see http://www.prisma-statement.org/

In total, 32,325 records (28,175 new) were identified from the database searches. After exclusion of duplicates, the titles and abstracts of 16,789 articles (14,243 new) were screened, of which 2598 articles (2385 new) were read in full. Excluded studies with corresponding reasons are shown in ESM Table 4. Finally, 169 publications were included, of which 147 were new publications (Fig. 1) [3–10, 13–173].

We conducted 177 meta-analyses (83 on COVID-19-related death and 94 on COVID-19 severity), compared with 77 meta-analyses in our original systematic review and meta-analysis [1]. The number of included individuals per study ranged from 24 (smallest study) to 235,248 (largest study). In total, our meta-analyses included 859,262 individuals with diabetes and confirmed SARS-CoV-2 infection for COVID-19-related death and 927,975 for COVID-19 severity (compared with 15,063 individuals with diabetes and confirmed SARS-CoV-2 infection for COVID-19-related death and 17,687 for COVID-19 severity in the original meta-analysis [1]). Most of the publications (*n*=76) were from Asia (China, *n*=28; Iran, *n*=13 ; South Korea, *n*=11; Turkey, *n*=7; India, *n*=5; Hong Kong, *n*=3; Saudi Arabia, *n*=2; Israel, *n*=2; Japan, *n*=2; Singapore, *n*=1; Philippines;* n*=1, Kuwait, *n*=1), with 46 from Europe (Italy, *n*=12; UK, *n*=9; France, *n*=8; Spain, *n*=8; Sweden, *n*=2; Russia, *n*=2; Belgium, *n*=1; Romania, *n*=1; Denmark, *n*=1; the Netherlands, *n*=1; Greece, *n*=1), 35 from North America (USA, *n*=31; Mexico, *n*=4), four from Africa (Egypt, *n*=3; South Africa, *n*=1) and three from South America (Brazil, *n*=2; Peru, *n*=1). Five studies were performed in an international setting. The majority of the studies were conducted in a hospital setting and used data from hospital-based records (*n*=136); 33 studies used registry or insurance data. Regarding diabetes type, 78 publications included individuals with only type 2 diabetes, three included individuals with only type 1 diabetes and 38 focused on both type 1 and type 2 diabetes; in 50 publications diabetes type was not specified. The characteristics of the studies are shown in detail in ESM Table 5.

Risk of bias was low in 35 studies, moderate in 67 studies, high in 66 studies and unclear in one study (ESM Fig. 1). The main reason for a high risk of bias was insufficient adjustment for confounding factors and/or inappropriate statistical analysis and reporting of the findings (ESM Fig. 2).

The results of the meta-analyses can be found in ESM Figs. 3–97. Details of the papers included in the meta-analyses are provided in ESM Table 5.

### General risk factors and COVID-19-related death and COVID-19 severity in individuals with diabetes and COVID-19

**Fig. 2:**
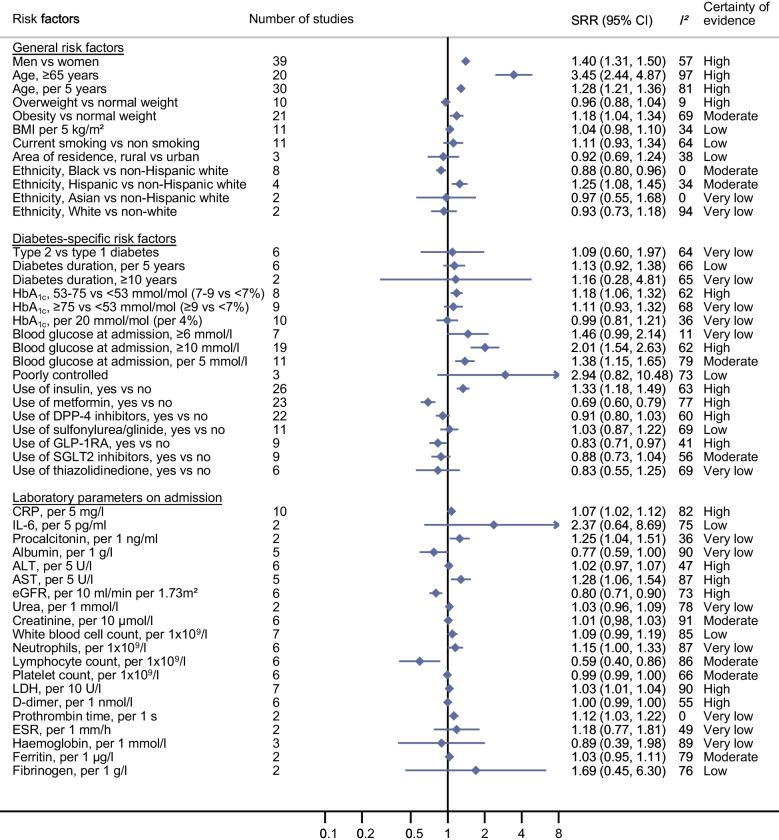
Prognostic factors and COVID-19-associated death in individuals with diabetes and COVID-19: general risk factors, diabetes-specific risk factors and laboratory variables. See ESM Figs. 3–97 for full details of the meta-analyses. Poorly controlled blood glucose was defined as a lowest fasting blood glucose of ≥3.9 mmol/l and a highest 2 h plasma glucose level >10.0 mmol/l during the observation window. ESR, erythrocyte sedimentation rate

**Fig. 3:**
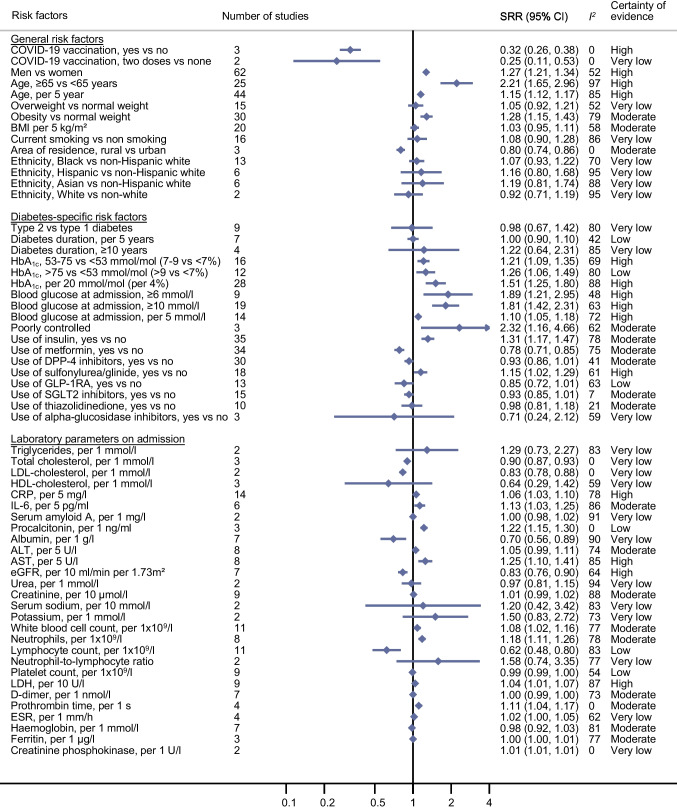
Prognostic factors and severity of COVID-19 in individuals with diabetes and COVID-19: general risk factors, diabetes-specific risk factors and laboratory variables. See ESM Figs. 3–97 for full details of the meta-analyses. Severity was defined as a composite endpoint including death, tracheal intubation for mechanical ventilation, acute respiratory distress syndrome, septic shock, intensive care unit admission, multiple organ dysfunction or failure, or hospital admission. See Fig. 2 for the definition of poorly controlled blood glucose. ESR, erythrocyte sedimentation rate

Updated meta-analyses (Fig. 2; ESM Table 6) confirmed a high certainty of evidence for the association between male sex and increased risk of COVID-19-related death (SRR 1.40 [95% CI 1.31, 1.50], *n*=39 studies [ESM Fig. 5]). For older age the certainty of evidence was now also rated as high (age ≥65 years: SRR 3.45 [95% CI 2.44, 4.87], *n*=20 studies [ESM Fig. 6]; age per 5 year increase: SRR 1.28 [95% CI 1.21, 1.36], *n*=30 studies [ESM Fig. 7]). New evidence emerged that obesity in patients with diabetes is related to an increased risk of COVID-19-related death (SRR 1.18 [95% CI 1.04, 1.34], *n*=21 studies, moderate certainty of evidence [ESM Fig. 9]). Similar associations were observed for COVID-19 severity (Fig. 3; ESM Table 7). There were no clear and consistent associations between being overweight, smoking status, area of residence and ethnicity and risk of COVID-19-related death and COVID-19 severity (certainty of evidence ranged from very low to moderate). For COVID-19 severity, new evidence became available for an association between vaccination against COVID-19 and lower risk of severe disease (SRR 0.32 [95% CI 0.26, 0.38], *n*=3 studies, high certainty of evidence [ESM Fig. 4]).

### Diabetes-specific risk factors and COVID-19-related death and COVID-19 severity in individuals with diabetes and COVID-19

Since the initial review [1], several new studies have been published on diabetes type and duration and COVID-19-related death and COVID-19 severity, but the estimates remain imprecise and the certainty of evidence for these associations is low or very low (Figs. 2 and 3; ESM Tables 6 and 7). HbA_1c_ level was not linearly related to COVID-19-related death (per 20 mmol/mol [per 4%] increase: SRR 0.99 [95% CI 0.81, 1.21], *n*=10 studies, very low certainty of evidence), but was linearly related to COVID-19 severity (per 20 mmol/mol [per 4%] increase: SRR 1.51 [95% CI 1.25, 1.80], *n*=28 studies, high certainty of evidence [ESM Fig. 22]). Using a cut-off of 53−75 mmol/mol (7–9%) vs <53 mmol/mol (<7%), high certainty of evidence was found for an increased risk of both outcomes (SRR for death: 1.18 [95% CI 1.06, 1.32], *n*=8 studies; SRR for severity: 1.21 [95% CI 1.09, 1.35], *n*=16 studies [ESM Fig. 20]). High blood glucose levels at admission were also related to an increased risk of both outcomes (per 5 mmol/l increase: SRR for death 1.38 [95% CI 1.15, 1.65], *n*=11 studies, moderate certainty of evidence; SRR for severity 1.10 [95% CI 1.05, 1.18], *n*=14 studies, high certainty of evidence [ESM Fig. 25]). Study findings on blood glucose thresholds (especially ≥10 mmol/l at admission) also indicated a higher risk of both outcomes, with high certainty of evidence (≥10 mmol/l: SRR for death 2.01 [95% CI 1.54, 2.63], *n*=19 studies; SRR for severity 1.81 [95% CI 1.42, 2.31], *n*=19 studies [ESM Fig. 24]).

Several new studies were available on diabetes treatment (Figs. 2 and 3; ESM Tables 6 and 7). There was high certainty of evidence that insulin use was related to a 33% increased risk of COVID-19-related death (SRR 1.33 [95% CI 1.18, 1.49], *n*=26 studies [ESM Fig. 27]), while metformin use was associated with a 31% decreased risk (SRR 0.69 [95% CI 0.60, 0.79], *n*=23 studies [ESM Fig. 28]). New evidence with high certainty emerged that glucagon-like peptide-1 receptor agonist (GLP-1RA) use was also associated with a lower risk of COVID-19-related death (SRR 0.83 [95% CI 0.71, 0.97], *n*=9 studies [ESM Fig. 31]). There was also evidence for a reduced risk of COVID-19-related death with use of dipeptidyl peptidase 4 (DPP-4) inhibitors (SRR 0.91 [95% CI 0.80, 1.03], *n*=22 studies, high certainty of evidence [ESM Fig. 29]) and use of sodium–glucose cotransporter 2 (SGLT2) inhibitors (SRR 0.88 [95% CI 0.73, 1.04], *n*=9 studies, moderate certainty of evidence [ESM Fig. 32]). For the other diabetes medications, there were no clear associations with risk of COVID-19-related death (Fig. 2; ESM Table 6). Similar findings were observed for COVID-19 severity (Fig. 3; ESM Table 7).

### Laboratory variables on admission and COVID-19-related death and COVID-19 severity in individuals with diabetes and COVID-19

The results for laboratory markers are shown in Figs. 2 and 3 and ESM Tables 6 and 7. There was new evidence with high certainty that C-reactive protein (CRP) level at admission was related to an increased risk of COVID-19-related death and COVID severity (per 5 mg/l increase: SRR for death 1.07 [95% CI 1.02, 1.12], *n*=10 studies; SRR for severity 1.06 [95% CI 1.03, 1.10], *n*=14 studies [ESM Fig. 73]). IL-6 level was also associated with severity of COVID-19 (per 5 pg/ml increase: SRR 1.13 [95% CI 1.03, 1.25], *n*=6 studies, moderate certainty of evidence [ESM Fig. 74]).

There was new evidence that higher aspartate aminotransferase (AST) levels at admission were associated with a higher risk of COVID-19-related death (per 5 U/l increase: SRR 1.28 [95% CI 1.06, 1.54], *n*=5 studies, high certainty of evidence; similar findings for severity [ESM Fig. 79]). For alanine aminotransferase (ALT), no clear associations were observed (ESM Fig. 78). New evidence was also found for an association of higher eGFR with decreased risk of COVID-19-related death (per 10 ml/min per 1.73m^2^ increase: SRR 0.80 [95% CI 0.71, 0.90], *n*=6 studies, high certainty of evidence; similar findings for severity [ESM Fig. 80]).

Lymphocyte count was also inversely associated with COVID-19-related death and COVID-19 severity (per 1 × 10^9^/l increase: SRR for death 0.59 [95% CI 0.40, 0.86], *n*=6 studies, moderate certainty of evidence; SRR for severity 0.62 [95% CI 0.48, 0.80] *n*=11 studies, low certainty of evidence [ESM Fig. 87]). Lactate dehydrogenase (LDH) level was also related to an increased risk of COVID-19-related death and COVID-19 severity, with high certainty of evidence for both (per 10 U/l increase: SRR for death 1.03 [95% CI 1.01, 1.04], *n*=7 studies; SRR for severity 1.04 [95% CI 1.01, 1.07], *n*=9 studies [ESM Fig. 91]).

### Comorbidities, complications and medication use and COVID-19-related death and COVID-19 severity in individuals with diabetes and COVID-19

**Fig. 4:**
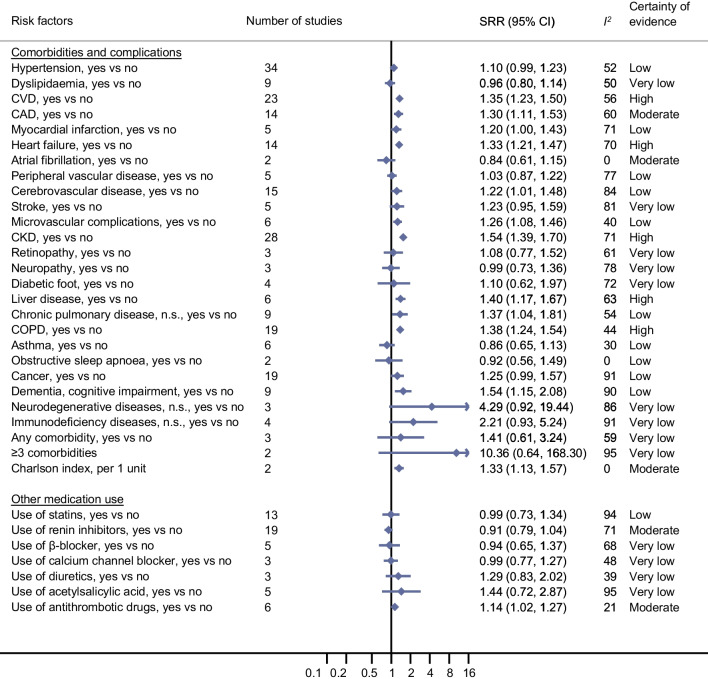
Prognostic factors and COVID-19-associated death in individuals with diabetes and COVID-19: comorbidities and complications and other medication use. See ESM Figs. 3–97 for full details of the meta-analyses. Renin inhibitors included ACE inhibitors, ARBs and non-specified RAS inhibitors. n.s., not specified

**Fig. 5:**
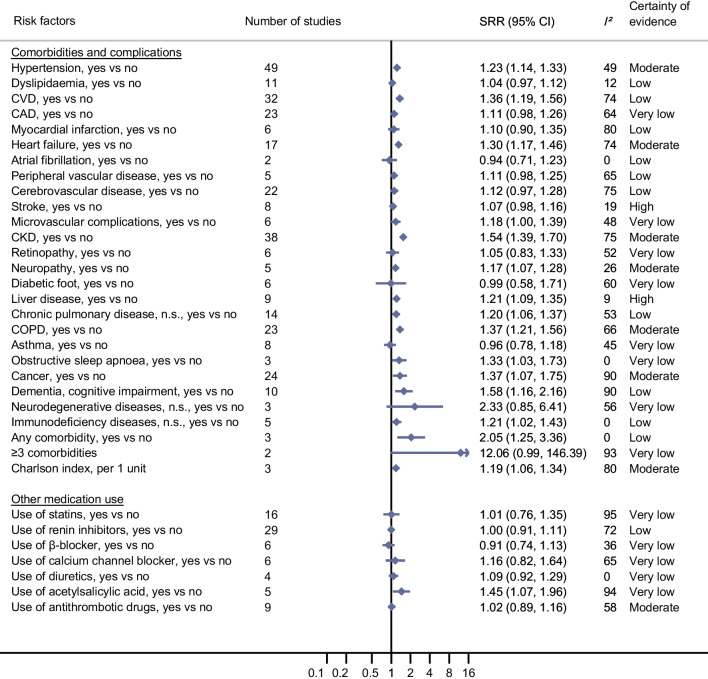
Prognostic factors and severity of COVID-19 in individuals with diabetes and COVID-19: comorbidities and complications and other medication use. See ESM Figs. 3–97 for full details of the meta-analyses and Fig. 3 for the definition of severity. Renin inhibitors included ACE inhibitors, ARBs and non-specified RAS inhibitors. n.s., not specified

In the updated meta-analyses, it was confirmed that the certainty of evidence was high for an association of pre-existing CVD with COVID-19-related death (SRR 1.35 [95% CI 1.23, 1.50], *n*=23 studies [Fig. 4; ESM Table 6; ESM Fig. 37]). New evidence with high certainty was also found for an association of heart failure (SRR 1.33 [95% CI 1.21, 1.47], *n*=14 studies [ESM Fig. 40]), CKD (SRR 1.54 [95% CI 1.39, 1.70], *n*=28 studies [ESM Fig 46]), liver disease (SRR 1.40 [95% CI 1.17, 1.67], *n*=6 studies [ESM Fig. 50]) and COPD (SRR 1.38 [95% CI 1.24, 1.54], *n*=19 studies [ESM Fig. 51]) with COVID-19-related death. New evidence with moderate certainty was identified for an association between coronary artery disease (CAD) (SRR 1.30 [95% CI 1.11, 1.53], *n*=14 studies [ESM Fig. 38]) and a comorbidity index (Charlson index) (per 1 unit increase: SRR 1.33 [95% CI 1.13, 1.57], *n*=2 studies [ESM Fig. 61]) and COVID-19-related death (Fig. 4; ESM Table 6). Similar associations were seen for COVID-19 severity (Fig. 5; ESM Table 7). While no clear association with COVID-19-related death was found for pre-existing hypertension, neuropathy and cancer, there was evidence with moderate certainty of an association of all three comorbidities with COVID-19 severity (SRR 1.23 [95% CI 1.14, 1.33], *n*=49 studies; 1.17 [95% CI 1.07, 1.28],* n*=5 studies; and 1.37 [95% CI 1.07, 1.75], *n*=24 studies, respectively [ESM Figs. 35, 48 and 55, respectively; ESM Tables 6 and 7]).

For medication use (other than diabetes medications), the certainty of evidence was moderate for use of antithrombotic drugs associated with an increased risk of COVID-19-related death but not with COVID-19 severity (SRR for death 1.14 [95% CI 1.02, 1.27], *n*=6 studies; SRR for severity 1.02 [0.89, 1.16], *n*=9 studies [Figs. 4 and 5; ESM Fig. 68]). New evidence emerged on the use of acetylsalicylic acid, also pointing to an increased risk, especially for COVID-19 severity, but the certainty of evidence was very low (ESM Fig. 67; ESM Tables 6 and 7).

### Subgroup analysis, heterogeneity, publication bias and sensitivity analysis

For each association, meta-analyses were stratified by risk of bias due to confounding (ESM Figs. 3–97). For associations that showed apparently different findings in the stratified meta-analysis, we conducted meta-regression adjusted by risk of bias due to confounding (ESM Table 8). Effect modification by adjustment status was observed for HbA_1c_ ≥75 mmol/mol (≥9%), use of statins and use of renin inhibitors with regard to COVID-19-related death and COVID-19 severity. For HbA_1c_ ≥75 mmol/mol (≥9%), a clear increased risk was observed for both outcomes for studies with a low/moderate risk of bias due to confounding (SRR for death 1.31 [95% CI 1.18, 1.44]; SRR for severity 1.47 [95% CI 1.31, 1.66]), but imprecisely estimated inverse associations were found for studies with a high risk of bias due to confounding (SRR for death 0.89 [95% CI: 0.75, 1.04]; SRR for severity 0.91 [95% CI 0.68, 1.21] [ESM Fig. 21]). For chronic use of statins and renin inhibitors, inverse associations for studies with a low/moderate risk of bias due to confounding were observed for COVID-19-related death and COVID-19 severity, but there was an increased risk of both outcomes in studies with a high risk of bias due to confounding (ESM Figs. 62 and 63).

Heterogeneity was particularly high for the laboratory markers, probably because of the different analytical methods and reference ranges used (Figs. 2 and 3; ESM Tables 6 and 7).

Findings on potential publication bias and small study effects are shown in ESM Figs. 98–132. According to Egger’s test, there was a suggestion of publication bias for the association of obesity, blood glucose per unit increase at admission and unspecified chronic obstructive diseases with COVID-19-related death, as well as for the association of overweight, obesity, blood glucose per unit increase at admission, use of thiazolidinediones, CKD, unspecified chronic pulmonary diseases, CRP level and lymphocyte count with COVID-19 severity, and the funnel plots show that studies with null or negative findings were missing (ESM Figs. 101, 102, 110, 117, 124, 125, 130 and 132). For insulin use and severity (ESM Fig. 111), hypertension and death/severity (ESM Fig. 118) and CVD and death (ESM Fig. 120), Egger’s tests also suggested publication bias; however, the funnel plots did not show specific patterns, only that small studies tended to be absent. In a sensitivity analysis, we calculated the 95% CIs by applying the Hartung–Knapp–Sidik–Jonkman method. In general, the findings were comparable to the results using the DerSimonian and Laird method. The few discrepancies were mainly observed for meta-analyses based on low numbers of primary studies (ESM Tables 9 and 10).

## Discussion

This updated systematic review and meta-analysis included 169 studies, of which 147 were new studies, with data from more than 910,000 new participants. In total, 177 meta-analyses were conducted to provide the best available evidence on risk phenotypes in diabetes regarding COVID-19-related death and COVID-19 severity. The evidence was strengthened that male sex, older age, blood glucose level at admission, use of insulin, use of metformin (inversely), lymphocyte count at admission (inversely) and pre-existing comorbidities such as CVD, CKD and COPD are associated with worse COVID-19-related outcomes. New robust evidence emerged that COVID-19 vaccination status, obesity, higher HbA_1c_ levels, chronic GLP-1RA use (inversely), pre-existing hypertension, heart failure, liver disease, neuropathy, cancer, the Charlson index, higher levels of CRP, IL-6, AST and LDH, and higher eGFR (inversely) are related to COVID-19-related death and/or COVID-19 severity in people with diabetes.

In this updated systematic review and meta-analysis, obesity was now identified as a risk factor for severe COVID-19 among patients with diabetes and confirmed SARS-CoV-2 infection. This is in line with findings among the general population [174] and has been confirmed in Mendelian randomisation analyses [175]. Interestingly, smoking, which has been identified as a causal risk factor for COVID-19 in the general population [176], was not clearly associated with COVID-19-related death and COVID-19 severity in populations with diabetes. We speculate that the low number of smokers among people with diabetes might explain our findings.

For diabetes-specific risk factors, such as diabetes type and duration, only a few studies are available that met our inclusion criteria. Thus, the certainty of evidence was low or very low and the estimates were very imprecise. Findings from population-based studies, including total populations of people with diabetes (but not all with confirmed SARS-CoV-2 infection), were inconsistent. For example, one study found an increased risk of COVID-19-related death for participants with type 2 diabetes compared with those with type 1 diabetes [177], whereas another study found no differences in COVID-19-related death or COVID-19 severity by type of diabetes [178]. Another study showed that both type 1 and type 2 diabetes were associated with COVID-19 severity and that the RR was similar (about threefold) for both types compared with people without diabetes [4]. For HbA_1c_, the association was clearer for COVID-19 severity than for COVID-19-related death, with a non-linear association for death. Population-based studies (also including people without SARS-CoV-2 infection and/or individuals without diabetes) also reported positive associations between higher HbA_1c_ levels and COVID-19 severity [178–180]. In addition, among the general population, a dose–response meta-analysis showed a linear increase in risk of COVID-19 severity for blood glucose levels [181], which was also observed in our meta-analysis including only people with diabetes. High blood glucose levels could be an indicator for poorly controlled diabetes, although it is also possible that blood glucose levels at admission were high because of COVID-19 infection, reflecting stress hyperglycaemia. A recent Mendelian randomisation analysis suggested that glycaemic traits and type 2 diabetes per se do not seem to increase the risk of COVID-19 severity [182]. Beyond this, it has been speculated that there is a bidirectional association between diabetes/blood glucose levels and COVID-19 [183, 184], and long-term studies exploring this relationship are warranted.

In this update we also identified several studies on the chronic use of glucose-lowering drugs, including insulin, metformin, DPP-4 inhibitors, sulfonylurea/glinides, GLP-1RAs, SGLT2 inhibitors, thiazolidinedione and alpha-glucosidase inhibitors. There was moderate to high certainty of evidence that insulin use was associated with an increased risk and use of metformin and GLP-1RA use were associated with a decreased risk of COVID-19-related death. Use of SGLT2 inhibitors and DPP-4 inhibitors was also associated with less severe illness. As discussed in our original review, we speculate that chronic insulin use can be seen as an indicator of more severe diabetes. For the other glucose-lowering medications, the certainty of evidence was low or very low, mainly because of a serious or even very serious risk of bias, inconsistency between studies and imprecise estimates. Another meta-analysis and a nationwide population study from England (including a population with diabetes but not all with SARS-CoV-2 infection) found similar associations between the use of glucose-lowering drugs and COVID-19 severity to those found in this study [185, 186]. These studies also reported a decreased risk for use of SGLT2 inhibitors but an increased risk for DPP-4 inhibitors, which was not seen in our meta-analyses.

In accordance with findings from the general population, we identified pre-existing CVD, CKD and COPD as clear risk factors for COVID-19 severity in people with diabetes [187–190]. New evidence emerged that heart failure, liver disease and pre-existing hypertension, neuropathy and cancer are also related to a worse course of COVID-19, which was also observed among the general population [174, 191–194].

With regard to other medications (not glucose-lowering drugs), the certainty of evidence was moderate for an association between the chronic use of antithrombotic drugs and increased risk of COVID-19-related death but not COVID-19 severity. This treatment is used for CVD prevention and therefore it can be seen as an indicator of early CVD. The findings on chronic use of statins and renin inhibitors merit further discussion. Interestingly, in meta-analyses stratified by risk of bias due to confounding, we observed inverse associations between statin and renin inhibitor use and COVID-19-related death and severity for studies with a low/moderate risk of bias due to confounding and an increased risk for studies with a high risk of bias due to confounding. Effect modification by adjustment for confounding was present. Systematic reviews and meta-analyses as well as Mendelian randomisation analyses among the general population also indicated a lower risk of severe COVID-19 with chronic use of statins and renin inhibitors, supporting our findings from meta-analyses adjusted for important confounders [195–198].

We also found robust new evidence that higher levels of inflammatory biomarkers (CRP, IL-6) at admission are associated with COVID-19-related death and disease severity. In addition, markers of liver disease (AST) and kidney disease (eGFR) were also related to worse outcomes. As these markers were measured at admission, the direction of the associations is not clear, and it has also been shown that COVID-19 causes systemic inflammation and leads to liver injury [199, 200].

Overall, the findings of our updated systematic review and meta-analysis support our hypothesis that it is not diabetes alone that influences the course of COVID-19, but rather the severity of diabetes and a person’s general health status that are important predictors of COVID-19 severity.

The following study limitations need to be taken into account. First, 39% of the studies were at high risk of bias, mainly because of inadequate adjustment and selection of important confounders. However, we stratified all meta-analyses by adjustment status and the findings were robust, with some exceptions as discussed above. Second, most of the included studies did not account for treatment of COVID-19 in the hospital setting and, thus, we could not consider this aspect in our meta-analyses. Third, the findings cannot be translated to all individuals with diabetes and SARS-CoV-2 infection, as most of the studies were conducted in the hospital setting and thus included people with a more severe form of COVID-19 and not those with a mild course of the disease. Fourth, we detected high levels of heterogeneity in some of the meta-analyses. We explored the influence of risk of bias due to confounding in stratified meta-analyses and meta-regression and heterogeneity could be partly explained. However, further aspects, for example geographic location or sex, were not investigated.

In conclusion, the update of our systematic review and meta-analysis provides new evidence on risk phenotypes of diabetes and COVID-19-related death and severity of COVID-19. There is robust evidence that vaccination against COVID-19, male sex, older age, obesity, higher HbA_1c_ levels, high blood glucose level at admission, chronic use of insulin, metformin (inversely) and GLP-1RAs (inversely), pre-existing comorbidities, including CVD, hypertension, heart failure, liver disease, CKD, neuropathy, COPD and cancer, a high comorbidity index, and high levels of CRP, IL-6, AST and LDH, a low eGFR and a low lymphocyte count at admission are all related to COVID-19-related death and COVID-19 severity among individuals with diabetes and confirmed SARS-CoV-2 infection.

## Supplementary Information

Below is the link to the electronic supplementary material.

Supplementary file1 (PDF 39647 KB)

## Data Availability

Data were extracted from published research papers, all of which are available and accessible. All datasets generated during the current study are available from the corresponding author on reasonable request. The study protocol has been published (PROSPERO registration no. CRD42020193692; www.crd.york.ac.uk/PROSPERO/) and is available without restrictions.

## Abbreviations

ACE: Angiotensin-converting enzyme
ALT: Alanine aminotransferase
ARB: Angiotensin II receptor blockers
AST: Aspartate aminotransferase
CAD: Coronary artery disease
CKD: Chronic kidney disease
COPD: Chronic obstructive pulmonary disease
CRP: C-reactive protein
DPP-4: Dipeptidyl peptidase 4
GLP-1RA: Glucagon-like peptide-1 receptor agonist
LDH: Lactate dehydrogenase
RAS: Renin–angiotensin system
SARS-CoV-2: Severe acute respiratory syndrome coronavirus-2
SGLT2: Sodium–glucose cotransporter 2
SRR: Summary relative risk
