# Supplementary material for: Risk phenotypes of diabetes and association with COVID-19 severity and death: an update of a living systematic review and meta-analysis
Source: Diabetologia. 2023 May 19;66(8):1395–412. doi: 10.1007/s00125-023-05928-1 (PMC10198038; doi:10.1007/s00125-023-05928-1)
Supplement: Supplementary file 1 — Supplementary file1 (PDF 39647 KB) [file 125_2023_5928_MOESM1_ESM.pdf]

Electronic supplementary material (ESM)

Risk phenotypes of diabetes and association with COVID-19 severity and death – an update of living a systematic review and meta-analysis

Sabrina Schlesinger[1,2], Alexander Lang[1], Nikoletta Christodoulou[1], Philipp Linnerz[1], Kalliopi Pafili[2,3], Oliver Kuss[1,2,4], Christian Herder[2,3,5], Manuela Neuenschwander[1,2], Janett Barbaresko[1], Michael Roden[2,3,5]

[1] Institute for Biometrics and Epidemiology, German Diabetes Center, Leibniz Center for Diabetes Research at Heinrich Heine University Düsseldorf, Düsseldorf, Germany;

[2] German Center for Diabetes Research (DZD), Partner Düsseldorf, München-Neuherberg, Germany;

[3] Institute for Clinical Diabetology, German Diabetes Center, Leibniz Center for Diabetes Research at Heinrich Heine University Düsseldorf, Düsseldorf, Germany;

[4] Centre for Health and Society, Faculty of Medicine, Heinrich-Heine-University, Düsseldorf, Germany;

[5] Department of Endocrinology and Diabetology, Medical Faculty and University Hospital Düsseldorf, Heinrich Heine University Düsseldorf, Düsseldorf, Germany.

Correspondence:

Sabrina Schlesinger PhD, Institute for Biometrics and Epidemiology, German Diabetes Center, Auf'm Hennekamp 65, 40225 Düsseldorf, Germany. Tel. +49 211 3382231. Email: sabrina.schlesinger@ddz.de

Table of contents

ESM Methods: Details on risk of bias assessment 9
ESM Table 1: Search strategy 10
ESM Table 2: Extracted data 11
ESM Table 3: Signaling questions for risk of bias assessment using QUIPS 12
ESM Table 4: List of excluded studies for first version and current update combined 18
ESM Table 5: Characteristics of included studies 19
ESM Table 6: Certainty of evidence for associations between phenotypes of patients with diabetes and SARS-CoV-2 regarding COVID-19 death using the GRADE tool 65
ESM Table 7: Certainty of evidence for associations between phenotypes of patients with diabetes and SARS-CoV-2 regarding COVID-19 severity using the GRADE tool 75
ESM Table 8: Meta-regression: Adjustment for risk of bias due to confounding, for associations showing differences in stratified analyses by confounding 86
ESM Table 9: Comparison of the 95% CIs derived by the DerSimonian-Laird method versus the Hartung-Knapp-Sidik-Jonkman method for associations between phenotypes of patients with diabetes and SARS-CoV-2 regarding COVID-19 death...87
ESM Table 10: Comparison of the 95% CIs derived by the DerSimonian-Laird method versus the Hartung-Knapp-Sidik-Jonkman method for associations between phenotypes of patients with diabetes and SARS-CoV-2 regarding COVID-19 severity 90
ESM Figure 1: Risk of bias of each study for each domain and overall...............................97
ESM Figure 2: Risk of bias of judgements within each bias domain....................................98
ESM Figure 3: Meta-analysis on **COVID-19 vaccination status, yes vs. no** and A) death and B) and severity of COVID-19 in patients with diabetes and COVID-19..........................99
ESM Figure 4: Meta-analysis on **COVID-19 vaccination status, two doses vs. none** and A) death and B) and severity of COVID-19 in patients with diabetes and COVID-19 100
ESM Figure 5: Meta-analysis on **men compared to women** and A) death and B) severity of COVID-19 in patients with diabetes and COVID-19...101
ESM Figure 6: Meta-analysis on **age ≥65 years** and A) death and B) and severity of COVID-19 in patients with diabetes and COVID-19 102
ESM Figure 7: Meta-analysis on **age per 1 year** and A) death and B) severity of COVID-19 in patients with diabetes and COVID-19 103
ESM Figure 8: Meta-analysis on **overweight** compared to normal weight and A) death and B) severity of COVID-19 in patients with diabetes and COVID-19 104
ESM Figure 9: Meta-analysis on **obesity** compared to normal weight and A) death and B) severity of COVID-19 in patients with diabetes and COVID-19..105
ESM Figure 10: Meta-analysis on **BMI**, per 1 kg/m² increase and A) death and B) severity of COVID-19 in patients with diabetes and COVID-19..106
ESM Figure 11: Meta-analysis on **smoking** compared to non-smoking and A) death and B) severity of COVID-19 in patients with diabetes and COVID-19..107
ESM Figure 12: Meta-analysis on **area of residence** (rural compared to urban) and A) death and B) severity of COVID-19 in patients with diabetes and COVID-19 108
ESM Figure 13: Meta-analysis on **ethnicity (black vs. non-Hispanic white)** and A) death and B) severity of COVID-19 in patients with diabetes and COVID-19 109
ESM Figure 14: Meta-analysis on **ethnicity (Hispanic vs. non-Hispanic white)** and A) death and B) severity of COVID-19 in patients with diabetes and COVID-19.....................110

ESM Figure 15: Meta-analysis on **ethnicity (Asian vs. non-Hispanic white)** and A) death and B) severity of COVID-19 in patients with diabetes and COVID-19 111
ESM Figure 16: Meta-analysis on **ethnicity (White vs. Non-white)** and A) death and B) severity of COVID-19 in patients with diabetes and COVID-19...112
ESM Figure 17: Meta-analysis on **type 2 vs type 1 diabetes** and A) death and B) severity of COVID-19 in patients with diabetes and COVID-19...113
ESM Figure 18: Meta-analysis on **diabetes duration**, per 1 year and A) death and B) severity of COVID-19 in patients with diabetes and COVID-19...114
ESM Figure 19: Meta-analysis on **diabetes duration**, **≥10 years** and A) death and B) severity of COVID-19 in patients with diabetes and COVID-19...115
ESM Figure 20: Meta-analysis on **HbA_{1c} 53-75 vs <53 mmol/mol (7-9 vs <7%)** and A) death and B) severity of COVID-19 in patients with diabetes and COVID-19.....................116
ESM Figure 21: Meta-analysis on **HbA_{1c} ≥75 vs <53 mmol/mol (≥9 vs <7%)** and A) death and B) severity of COVID-19 in patients with diabetes and COVID-19 117
ESM Figure 22: Meta-analysis on **HbA_{1C}, per 1%** increase and A) death and B) severity of COVID-19 in patients with diabetes and COVID-19...118
ESM Figure 23: Meta-analysis on **blood glucose ≥6 - <10 mmol/l** at admission and A) death and B) severity of COVID-19in patients with diabetes and COVID-19......................119
ESM Figure 24 Meta-analysis on **blood glucose ≥10 mmol/l** at admission and A) death and B) severity of COVID-19 in patients with diabetes and COVID-19 120
ESM Figure 25: Meta-analysis on **blood glucose per 1 mmol/l increase** at admission and A) death and B) severity of COVID-19 in patients with diabetes and COVID-19 121
ESM Figure 26: Meta-analysis for **poorly vs. well-controlled at admission** and A) death and B) severity of COVID-19 in patients with diabetes and COVID-19 122
ESM Figure 27: Meta-analysis on **insulin use** compared to non-use and A) death and B) severity of COVID-19 in patients with diabetes and COVID-19...123
ESM Figure 28: Meta-analysis on **metformin use** compared to non-use and A) death and B) severity of COVID-19 in patients with diabetes and COVID-19...124
ESM Figure 29: Meta-analysis on **DPP-4-inhibitors use** compared to non-use and A) death and B) severity of COVID-19 in patients with diabetes and COVID-19 125
ESM Figure 30: Meta-analysis on use of **sulfonylurea/glinides/secretagogues** compared to non-use and A) death and B) severity of COVID-19 in patients with diabetes and COVID-19...126
ESM Figure 31: Meta-analysis on use of **GLP 1-RA** compared to non-use and A) death and B) severity of COVID-19 in patients with diabetes and COVID-19 127
ESM Figure 32: Meta-analysis on use of **SGLT-2i** compared to non-use and A) death and B) severity of COVID-19 in patients with diabetes and COVID-19...128
ESM Figure 33: Meta-analysis on use of **thiazolidinedione** compared to non-use and A) death and B) severity of COVID-19 in patients with diabetes and COVID-19.....................129
ESM Figure 34: Meta-analysis on use of **alpha-glucosidase inhibitors** compared to non-use and A) death and B) severity of COVID-19 in patients with diabetes and COVID-19....130
ESM Figure 35: Meta-analysis on **hypertension** compared to no hypertension and A) death and B) severity of COVID-19 in patients with diabetes and COVID-19 131
ESM Figure 36: Meta-analysis on **dyslipidaemia** compared to no dyslipidaemia and A) death and B) severity of COVID-19 in patients with diabetes and COVID-19.....................132

ESM Figure 37: Meta-analysis on pre-existing **cardiovascular disease (CVD)** compared to no CVD and A) death and B) severity of COVID-19 in patients with diabetes and COVID-19 133
ESM Figure 38: Meta-analysis on pre-existing **coronary artery disease (CAD)** compared to no CAD and A) death and B) severity of COVID-19 in patients with diabetes and COVID-19 134
ESM Figure 39: Meta-analysis on pre-existing **myocardial infarction (MI)** compared to no MI and A) death and B) severity of COVID-19 in patients with diabetes and COVID-19 135
ESM Figure 40: Meta-analysis on pre-existing **heart failure (HF)** compared to no HF and A) death and B) severity of COVID-19 in patients with diabetes and COVID-19......................136
ESM Figure 41: Meta-analysis on pre-existing **atrial fibrillation (AF)** compared to no AF and A) death and B) severity of COVID-19 in patients with diabetes and COVID-19 137
ESM Figure 42: Meta-analysis on pre-existing **peripheral vascular disease (PVD)** compared to no PVD and A) death and B) severity of COVID-19 in patients with diabetes and COVID-19....................138
ESM Figure 43: Meta-analysis on pre-existing **cerebrovascular disease** compared to no cerebrovascular disease and A) death and B) severity of COVID-19 in patients with diabetes and COVID-19....................139
ESM Figure 44: Meta-analysis on pre-existing **stroke** compared to no stroke and A) death and B) severity of COVID-19 in patients with diabetes and COVID-19 140
ESM Figure 45: Meta-analysis on pre-existing **microvascular diseases (MVD)** compared to no MVD and A) death and B) severity of COVID-19 in patients with diabetes and COVID-19 141
ESM Figure 46: Meta-analysis on pre-existing **chronic kidney disease (CKD)** compared to no CKD and A) death and B) severity of COVID-19 in patients with diabetes and COVID-19 142
ESM Figure 47: Meta-analysis on pre-existing **retinopathy** compared to no retinopathy and A) death and B) severity of COVID-19 in patients with diabetes and COVID-19.................143
ESM Figure 48: Meta-analysis on pre-existing **neuropathy** compared to no neuropathy and A) death and B) severity of COVID-19 in patients with diabetes and COVID-19.................144
ESM Figure 49: Meta-analysis on pre-existing **diabetic foot** compared to no diabetic foot and A) death and B) severity of COVID-19 in patients with diabetes and COVID-19 145
ESM Figure 50: Meta-analysis on pre-existing **liver disease** compared to no liver disease and A) death and B) severity of COVID-19 in patients with diabetes and COVID-19 146
ESM Figure 51: Meta-analysis on pre-existing **chronic pulmonary disease** (not specified) compared to no chronic pulmonary disease and A) death and B) severity of COVID-19 in patients with diabetes and COVID-19....................147
ESM Figure 52: Meta-analysis on pre-existing **chronic obstructive pulmonary disease (COPD)** compared to no COPD and A) death and B) severity of COVID-19 in patients with diabetes and COVID-19 148
ESM Figure 53: Meta-analysis on pre-existing **asthma** compared to no asthma and A) death and B) severity of COVID-19 in patients with diabetes and COVID-19 149
ESM Figure 54: Meta-analysis on pre-existing **obstructive sleep apnoea (OSA)** compared to no OSA and A) death and B) severity of COVID-19 in patients with diabetes and COVID-19....................150
ESM Figure 55: Meta-analysis on pre-existing **cancer** compared to no cancer and A) death and B) severity of COVID-19 in patients with diabetes and COVID-19 151

ESM Figure 56: Meta-analysis on pre-existing **dementia/cognitive impairment** compared to no dementia and A) death and B) severity of COVID-19 in patients with diabetes and COVID-19....152
ESM Figure 57: Meta-analysis on pre-existing **neurodegenerative disease (not specified)** compared to no neurodegenerative disease and A) death and B) severity of COVID-19 in patients with diabetes and COVID-19....153
ESM Figure 58: Meta-analysis on pre-existing **immunodeficiency disorders (not specified)** compared to immunodeficiency disorders and A) death and B) severity of COVID-19 in patients with diabetes and COVID-19....154
ESM Figure 59: Meta-analysis on **any comorbidities** compared to no comorbidities and A) death and B) severity of COVID-19 in patients with diabetes and COVID-19....155
ESM Figure 60: Meta-analysis on **≥3 comorbidities** compared to no comorbidities and A) death and B) severity of COVID-19 in patients with diabetes and COVID-19....156
ESM Figure 61: Meta-analysis on **Charlson index**, per 1 unit compared to no comorbidities and A) death and B) severity of COVID-19 in patients with diabetes and COVID-19....157
ESM Figure 62: Meta-analysis on **use of statins** compared to non-use and A) death and B) severity of COVID-19 in patients with diabetes and COVID-19....158
ESM Figure 63: Meta-analysis on **use of renin inhibitor** compared to non-use and A) death and B) severity of COVID-19 in patients with diabetes and COVID-19....159
ESM Figure 64: Meta-analysis on **use of beta-blockers** compared to non-use and A) death and B) severity of COVID-19 in patients with diabetes and COVID-19....160
ESM Figure 65: Meta-analysis on **use of calcium channel blocker** compared to non-use and A) death and B) severity of COVID-19 in patients with diabetes and COVID-19....161
ESM Figure 66: Meta-analysis on **use of diuretics** compared to non-use and A) death and B) severity of COVID-19 in patients with diabetes and COVID-19....162
ESM Figure 67: Meta-analysis on **use of acetylsalicylic acid (ASA)** compared to non-use and A) death and B) severity of COVID-19 in patients with diabetes and COVID-19....163
ESM Figure 68: Meta-analysis on **use of antithrombotic drugs (antiplatelet/ anticoagulant)** compared to non-use and A) death and B) severity of COVID-19 in patients with diabetes and COVID-19....164
ESM Figure 69: Meta-analysis on **triglycerides**, per 1 per mmol/l and A) death and B) severity of COVID-19 in patients with diabetes and COVID-19....165
ESM Figure 70: Meta-analysis on **total cholesterol,** per 1 per mmol/l and A) death and B) severity of COVID-19 in patients with diabetes and COVID-19....166
ESM Figure 71: Meta-analysis on **LDL-cholesterol,** per 1 per mmol/l and A) death and B) severity of COVID-19 in patients with diabetes and COVID-19....167
ESM Figure 72: Meta-analysis on **HDL-cholesterol,** per 1 per mmol/l and A) death and B) severity of COVID-19 in patients with diabetes and COVID-19....168
ESM Figure 73: Meta-analysis on **C-reactive protein (CRP**), per 1 mg/l and A) death and B) severity of COVID-19 in patients with diabetes and COVID-19....169
ESM Figure 74: Meta-analysis on **IL-6**, per 1 pg/ml and A) death and B) severity of COVID-19 in patients with diabetes and COVID-19....170
ESM Figure 75: Meta-analysis on **serum amyloid A**, per 1 mg/l and A) death and B) severity of COVID-19 in patients with diabetes and COVID-19....171
ESM Figure 76: Meta-analysis on **procalcitonin**, per 1 ng/ml and A) death and B) severity of COVID-19 in patients with diabetes and COVID-19....172

ESM Figure 77: Meta-analysis on **albumin**, per 1 g/l and A) death and B) severity of COVID-19 in patients with diabetes and COVID-19 173
ESM Figure 78: Meta-analysis on **ALT**, per 1U/l and A) death and B) severity of COVID-19 in patients with diabetes and COVID-19 174
ESM Figure 79: Meta-analysis on **AST** per 1U/l and A) death and B) severity of COVID-19 in patients with diabetes and COVID-19....................175
ESM Figure 80: Meta-analysis on **eGFR**, per 1 ml/min per 1.73 m^2 and A) death and B) severity of COVID-19 in patients with diabetes and COVID-19....................176
ESM Figure 81: Meta-analysis on **urea**, per 1 mmol/l and A) death and B) severity of COVID-19 in patients with diabetes and COVID-19....................177
ESM Figure 82: Meta-analysis on **creatinine,** per 1 µmol/l and A) death and B) severity of COVID-19 in patients with diabetes and COVID-19....................178
ESM Figure 83: Meta-analysis on **creatine phosphokinase (CPK),** per 1 U/l and A) death and B) severity of COVID-19 in patients with diabetes and COVID-19 179
ESM Figure 84: Meta-analysis on **serum sodium,** per 1 mmol/l and A) death and B) severity of COVID-19 in patients with diabetes and COVID-19....................180
ESM Figure 85: Meta-analysis on **potassium,** per 1 mmol/l and A) death and B) severity of COVID-19 in patients with diabetes and COVID-19....................181
ESM Figure 86: Meta-analysis on **white blood cell count**, per $1x10^9$/l and A) death and B) severity of COVID-19 in patients with diabetes and COVID-19....................182
ESM Figure 87: Meta-analysis on **lymphocyte count**, per $1x10^9$/l and A) death and B) severity of COVID-19in patients with diabetes and COVID-19....................183
ESM Figure 88: Meta-analysis on **neutrophils**, per $1x10^9$/l and A) death and B) severity of COVID-19 in patients with diabetes and COVID-19....................184
ESM Figure 89: Meta-analysis on **neutrophil-to-lymphocyte ratio** and A) death and B) severity of COVID-19 in patients with diabetes and COVID-19....................185
ESM Figure 90: Meta-analysis on **platelet count**, per $1x10^9$/l and A) death and B) severity of COVID-19 in patients with diabetes and COVID-19....................186
ESM Figure 91: Meta-analysis on **lactate dehydrogenase**, per 1 unit/l and A) death and B) severity of COVID-19 in patients with diabetes and COVID-19....................187
ESM Figure 92: Meta-analysis on **d-dimer**, per 1 mg/l and A) death and B) severity of COVID-19 in patients with diabetes and COVID-19....................188
ESM Figure 93: Meta-analysis on **prothrombin time**, per 1 s and A) death and B) severity of COVID-19 in patients with diabetes and COVID-19....................189
ESM Figure 94: Meta-analysis on **erythrocyte sedimentation rate**, per 1 mm/h and A) death and B) severity of COVID-19 in patients with diabetes and COVID-19....................190
ESM Figure 95: Meta-analysis on **haemoglobin**, per 1 g/dl and A) death and B) severity of COVID-19 in patients with diabetes and COVID-19....................191
ESM Figure 96: Meta-analysis on **ferritin, per 1 pmol/L** and A) death and B) severity of COVID-19 in patients with diabetes and COVID-19....................192
ESM Figure 97: Meta-analysis on **fibrinogen**, per 1 g/l and A) death and B) severity of COVID-19 in patients with diabetes and COVID-19....................193
ESM Figure 98: Funnel plot for association for **men versus women** and A) death and B) severity of COVID-19 in patients with diabetes and COVID -19....................194
ESM Figure 99: Funnel plot for association between **age ≥65 years** and A) death and B) severity of COVID-19 in patients with diabetes and COVID -19....................195

ESM Figure 100: Funnel plot for association between **age per 5 year** increase and A) death and B) severity of COVID-19 in patients with diabetes and COVID-19 196
ESM Figure 101: Funnel plot for association between **overweight** and A) death and B) severity of COVID-19 in patients with diabetes and COVID-19..197
ESM Figure 102: Funnel plot for association between **obesity** and A) death and B) severity of COVID-19 in patients with diabetes and COVID-19..198
ESM Figure 103: Funnel plot for association between **BMI per 1 kg/m²** and A) death and B) severity of COVID-19 in patients with diabetes and COVID-19..199
ESM Figure 104: Funnel plot for association between **smoking** and A) death and B) severity of COVID-19 in patients with diabetes and COVID-19..200
ESM Figure 105: Funnel plot for association between **ethnicity (Black vs. Non-Hispanic white)** and A) death and B) severity of COVID-19 in patients with diabetes and COVID-19 201
ESM Figure 106: Funnel plot for association between **HbA$_{1c}$ 53-75 vs <53 mmol/mol (7-9 vs <7%)** and A) death and B) severity of COVID-19 in patients with diabetes and COVID-19 202
ESM Figure 107: Funnel plot for association between **HbA$_{1c}$ ≥75 vs <53 mmol/mol (≥9 vs <7%)** and A) death and B) severity of COVID-19 in patients with diabetes and COVID-19.203
ESM Figure 108: Funnel plot for association between **HbA$_{1c}$ per 1% increase** and A) death and B) severity of COVID-19 in patients with diabetes and COVID-19 204
ESM Figure 109: Funnel plot for association between **glucose ≥10 mmol/l** and A) death and B) severity of COVID-19 in patients with diabetes and COVID-19 205
ESM Figure 110: Funnel plot for association between **glucose per 1 mmol/l** and A) death and B) severity of COVID-19 in patients with diabetes and COVID -19 206
ESM Figure 111: Funnel plot for association between **insulin** and A) death and B) severity of COVID-19 in patients with diabetes and COVID-19..207
ESM Figure 112: Funnel plot for association between **metformin** and A) death and B) severity of COVID-19 in patients with diabetes and COVID -19...208
ESM Figure 113: Funnel plot for association between **DPP-4 inhibitor use** and A) death and B) severity of COVID-19 in patients with diabetes and COVID-19 209
ESM Figure 114: Funnel plot for association between **GLP 1-RA use** and A) death and B) severity of COVID-19 in patients with diabetes and COVID-19..210
ESM Figure 115: Funnel plot for association between **sulfonylurea/glinides/ secretagogues** and A) death and B) severity of COVID-19 in patients with diabetes and COVID-19...211
ESM Figure 116: Funnel plot for association between **SGLT-2i use** and A) death and B) severity of COVID-19 in patients with diabetes and COVID-19..212
ESM Figure 117: Funnel plot for association between **thiazolidinedione use** and A) death and B) severity of COVID-19 in patients with diabetes and COVID-19 213
ESM Figure 118: Funnel plot for association between **hypertension** and A) death and B) severity of COVID-19 in patients with diabetes and COVID -19...214
ESM Figure 119: Funnel plot for association between **dyslipidemia** and A) death and B) severity of COVID-19 in patients with diabetes and COVID -19...215
ESM Figure 121: Funnel plot for association between **CAD** and A) death and B) severity of COVID-19 in patients with diabetes and COVID-19...217
ESM Figure 122: Funnel plot for association between **HF** and A) death and B) severity of COVID-19 in patients with diabetes and COVID-19...218

ESM Figure 123: Funnel plot for association between **cerebrovascular diseases** and A) death and B) severity of COVID-19 in patients with diabetes and COVID-19......................219
ESM Figure 124: Funnel plot for association between **CKD** and A) death and B) severity of COVID-19 in patients with diabetes and COVID-19...220
ESM Figure 125: Funnel plot for association between **chronic pulmonary disease** (not specified) and A) death and B) severity of COVID-19 in patients with diabetes and COVID-19 221
ESM Figure 126: Funnel plot for association between **COPD** and A) death and B) severity of COVID-19 in patients with diabetes and COVID-19...222
ESM Figure 127: Funnel plot for association between **cancer** and A) death and B) severity of COVID-19 in patients with diabetes and COVID-19...223
ESM Figure 128: Funnel plot for association between **statins use** and A) death and B) severity of COVID-19 in patients with diabetes and COVID-19...224
ESM Figure 129: Funnel plot for association between **RAAS inhibitor** use and A) death and B) severity of COVID-19 in patients with diabetes and COVID-19 225
ESM Figure 130: Funnel plot for association between **CRP** and A) death and B) severity of COVID-19 in patients with diabetes and COVID-19...226
ESM Figure 131: Funnel plot for association between **white blood cell count** and A) death and B) severity of COVID-19 in patients with diabetes and COVID-19 227
ESM Figure 132: Funnel plot for association between **lymphocyte count** and A) death and B) severity of COVID-19 in patients with diabetes and COVID-19 228
References..229

ESM Methods: Details on risk of bias assessment

Each domain was rated as low, moderate or high risk of bias, or no information. Determining the overall risk of bias of each study, we put special emphasis on the domains comprising study confounding, statistical analysis/reporting and study participation. Studies were judged as high risk of bias if one of these domains was judged as high risk of bias. Studies were judged as low risk of bias, if all domains were judged as low risk of bias, or if confounding and statistical analysis/reporting were low risk of bias, and none of the other domains were judged as high risk of bias. In other cases, studies were rated as moderate risk of bias.

**ESM Table 1:** Search strategy	
PubMed	
#1	diabetes mellitus[MeSH Terms]) OR diabetes OR diabetic*
#2	covid19 OR covid-19 OR covid OR corona OR new-corona OR novel-corona OR coronavir* OR SARS-CoV-2 OR nCoV OR 2019-nCoV
#3	Combine: #1 AND #2
Epistemonikos	
#1	advanced_title_en:(diabetes mellitus OR diabetes OR diabetic*) OR advanced_abstract_en:(diabetes mellitus OR diabetes OR diabetic*)
#2	advanced_title_en:(covid19 OR covid-19 OR covid OR corona OR new-corona OR novel-corona OR coronavir* OR SARS-CoV-2 OR nCoV OR 2019-nCoV) OR advanced_abstract_en:(covid19 OR covid-19 OR covid OR corona OR new-corona OR novel-corona OR coronavir* OR SARS-CoV-2 OR nCoV OR 2019-nCoV)
#3	Combine: #1 AND #2
Web of Science	
#1	TOPIC: (diabetes mellitus OR diabetes OR diabetic*)
#2	TOPIC: (covid19 OR covid-19 OR covid OR corona OR new-corona OR novel-corona OR coronavir* OR SARS-CoV-2 OR nCoV OR 2019-nCoV)
#3	Combine: #1 AND #2
COVID-19 Research Database	
#1	(tw:(diabetes mellitus OR diabetes OR diabetic*))
#2	(tw:(covid19 OR covid-19 OR covid OR corona OR new-corona OR novel-corona OR coronavir* OR SARS-CoV-2 OR nCoV OR 2019-nCoV))
#3	Combine: #1 AND #2

**ESM Table 2:** Extracted data	
Information of included publications	• The first author's last name<br>• Date of publication<br>• Study design<br>• Geographic area<br>• Number of participants<br>• Number of cases
Patients' characteristics	• Age<br>• Sex<br>• BMI<br>• Smoking status<br>• Ethnicity
Diabetes-specific characteristics	• Type of diabetes<br>• Duration of diabetes<br>• Glycaemic control<br>• Diabetes treatment
Metabolic parameters	• Blood pressure/ hypertension<br>• Inflammatory biomarkers<br>• Liver enzymes<br>• Specific laboratory markers
Diabetes-related complications	• Macrovascular diseases (CVD: coronary heart diseases and stroke etc.)<br>• Microvascular diseases (nephropathy, neuropathy, retinopathy)
Comorbidities	• Respiratory diseases<br>• Cancer<br>• Immunosuppressive conditions
Outcome	• Definition of outcome<br>• Outcome assessment
Findings	• Crude risk estimates and 95% CIs<br>• If available multivariable-adjusted risk estimates with 95% CIs<br>• Confounders

**ESM Table 3:** Signaling questions for risk of bias assessment using QUIPS	
**Risk of Bias assessment using the Cochrane QUIPS tool**	
**Signalling question**	**Authors‘ judgement**
**Study participation: yes/partial yes/no/partial no/unclear**	**The study sample adequately represents the population of interest**
a) Adequate participation in the study by eligible people	Patients with confirmed COVID-19 (PCR or clinically) and diagnosed diabetes mellitus according to the ADA or other internationally recognized standards. High risk of bias, if high proportion of participants without confirmed COVID diagnosis were included
b) Description of the source population or population of interest	Source population or population of interest is clearly described (e.g. region, setting e.g. hospital)
c) Description of the baseline study sample	Baseline study sample is clearly described (characteristics table)
d) Adequate description of the sampling frame and recruitment	Recruitment, selection criteria and key characteristics of the source population clearly described, e.g. low risk of bias for sampling in hospitals using medical files
e) Adequate description of the period and place of recruitment	Time period and place of recruitment are clearly described, e.g. name of hospital and date. Answer partial yes, if date is not specified, because we know the approximate time (beginning 2020).
f) Adequate description of inclusion and exclusion criteria	Inclusion and exclusion criteria are clearly described and presented. Answer unclear, if no exclusion criteria are defined
**Study participation: risk of bias rating (high/low/unclear)**	**High**: most items are answered with ’no’ or if signalling question a is answered with ‘no’; **Low**: all items answered with ’yes’; **Unclear**: most items are answered with ’unclear’ Note: potentially a single item may introduce a high risk of bias, depending on study specifics
**Study attrition: Yes/partial yes/no/partial no/unclear**	**The study data available (i.e., participants not lost to follow-up) adequately represent the study sample**

a) Adequate response rate for study participants	Response rate during follow-up sufficient. For most of the studies not applicable because patients were in hospital and followed-up for this time period.
b) Attempts to collect information on participants who dropped out described	Attempts to collect information on participants who dropped out are described (e.g. via registers). Can be ignored, if loss to follow-up is low.
c) Reasons for loss to follow-up provided	Information about the reason participants were lost to follow-up are set up (e.g. participants refused). Can be ignored, if loss to follow-up is low.
d) Adequate description of participants lost to follow-up	Key characteristics of participants lost to follow-up are described (Age, sex, comorbidities). Can be ignored, if loss to follow-up is low.
e) No important differences between participants who completed the study and those who did not	No important differences in baseline characteristics between responders and non-responders. Can be ignored, if loss to follow-up is low.
**Study attrition: risk of bias rating (high/low/unclear)**	**High**: most items are answered with ’no’; **Low**: all items answered with ’yes’; **Unclear**: most items are answered with ’unclear’ Note: potentially a single item may introduce a high risk of bias, depending on study specifics
**Prognostic factor measurements: yes/partial yes/no/partial no/unclear**	**The PF is measured in a similar way for all participants**

a) Clear definition or description provided	Clear definition of the investigated phenotypes: patients' characteristics (age, sex, BMI, smoking status, ethnicity), metabolic parameters (e.g. glycaemic control, blood pressure, blood lipids, inflammatory biomarkers, liver enzymes), diabetes-related complications (macro- and microvascular diseases), comorbidities (respiratory diseases, cancer, immunosuppressive conditions)
b) Adequately valid and reliable method of measurement	Valid and reliable methods of assessment of the phenotypes listed above.<br>The following methods were considered as reliable and valid: assessed at hospital admission/data collection from medical files, contact of the patient's general/specialist practitioners, regular pharmacist or biomedical laboratory
c) Continuous variables reported or appropriate cut points used	Appropriate cut points:<br>BMI: ≥25 kg/m² for overweight and ≥30 kg/m² for obesity<br>$HbA_{1}c$ >53 mmol/mol/ >7%
d) Same method and setting of measurement used in all study participants	Measurements of the phenotypes are the same for all study participants
e) Adequate proportion of the study sample had complete data	Number in final model with complete data
f) Appropriate methods of imputation were used for missing data	Multiple imputations is a valid method. Complete case analysis for exposure and outcome variables are required.
**Prognostic factor measurements: risk of bias rating (high/low/unclear)**	**High**: most items are answered with 'no'; **Low**: all items answered with 'yes'; **Unclear**: most items are answered with 'unclear' Note: potentially a single item may introduce a high risk of bias, depending on study specifics
**Outcome measurement: yes/partial yes/no/partial no/unclear**	**The outcome of interest is measured in a similar way for all participants**

a) Clear definition of the outcome provided	Clear definition for the outcomes: death and composite endpoint/severity of COVID-19. How was the composite endpoint/severity of COVID-19 defined? Answer yes, if outcome is defined as in-hospital mortality. Answer unclear, if outcome is defined as “all-cause mortality” or mortality/death/lethality without further specification
b) Use of adequately valid and reliable methods of outcome measurement	Valid and reliable methods of assessment of the outcomes. E. g. collection of COVID-19-related clinical data during the hospital stay
c) Use of same method and setting of outcome measurement in all study participants	Measurements of the outcomes are the same for all study participants.
**Outcome measurement: risk of bias rating (high/low/unclear)**	**High**: most items are answered with ’no’; **Low**: all items answered with ’yes’; **Unclear**: most items are answered with ’unclear’ Note: potentially a single item may introduce a high risk of bias, depending on study specifics
**Study confounding: yes/partial yes/no/partial no/unclear**	**Important potential confounding factors are appropriately accounted for**

a) Measurement of all important confounders	Minimal adjusted models should include: Age, sex, BMI/overweight/obesity, at least one comorbid condition, as most important confounders
b) Provision of clear definitions of the important confounders measured	Measurements of confounders are described and defined
c) Adequately valid and reliable measurements of all important confounders	Measurements of confounding factors are valid and reliable (assessed at hospital admission, medical records, contact of the patient's general/specialist practitioners or regular pharmacist)
d) Use of the same method and setting of confounding measurement in all study participants	The method and setting of confounding measurement are the same for all participants
e) Appropriate imputation methods used for missing confounders (if applicable)	Appropriate methods of imputation for missing covariate data are applied and described.
f) Important potential confounders were accounted for the study design	Most important potential confounders are accounted for in the study design (e.g., matching for key variables, stratification). Minimal adjusted models should include: Age, sex, BMI/overweight/obesity, at least one comorbid condition, as most important confounders
g) Important confounders were accounted for in the analysis	Most important potential confounders are accounted for in the analysis (i.e., use of multivariable analysis). Minimal adjusted models should include: Age, sex, BMI/overweight/obesity, at least one comorbid condition, as most important confounders
**Study confounding measurement: risk of bias rating (high/low/unclear)**	**High**: most items are answered with 'no' or if signalling questions f) and g) are answered with 'no'; **Low**: all items answered with 'yes'; **Unclear**: most items are answered with 'unclear' Note: potentially a single item may introduce a high risk of bias, depending on study specifics
**Statistical analysis and reporting: yes/partial yes/no/partial no/unclear**	**Statistical Analysis and Reporting**

a) Sufficient presentation of data to assess the adequacy of the analytic strategy	There is sufficient presentation of data to assess the adequacy of the analysis (e.g. findings are displayed in a table or in the text). The results are expressed as risk ratios (e.g. hazard ratios, relative risks, odds ratios) with corresponding 95% confidence intervals
b) Strategy for model building is appropriate and based on conceptual framework model	The selection of the confounders are described and appropriate (e.g. selection of existing knowledge). Stepwise regression is not appropriate, with the exception that the known important confounders were selected.
c) Statistical model is adequate for the study design	Multivariable logistic regression or cox proportional hazard model or Weibull analysis are applied. Univariate methods are not appropriate.
d) No selective reporting of results	There is no selective reporting of results (e.g. findings are shown for a specific age group, time period etc.). Answer yes (no selective reporting), if data were missing in the manuscript and authors sent additional information via email, but note in the judgement that data were missing in the article and additional information was received via email.
**Statistical analysis and reporting: risk of bias rating (high/low/unclear)**	**High**: most items are answered with ’no’; **Low**: all items answered with ’yes’; **Unclear**: most items are answered with ’unclear’ Note: potentially a single item may introduce a high risk of bias, depending on study specifics

**ESM Table 4:** List of excluded studies for first version and current update combined	
**Reasons for exclusion**	**References**
Not relevant population/ not relevant comparison group, e.g. patients without diabetes and/or without COVID-19	[1-1239]
Not relevant data	[1240-1573]
Data not usable for meta-analysis	[1574-1577]
No risk estimates	[1578-1712]
Not relevant exposure	[1713-1737]
Not relevant outcome	[1738-1744]
Abstract/letter/editorial/comment/protocol	[1745-2224]
Review/meta-analysis	[2225-2450]
Not in English	[2451-2455]
Duplicate cohort	[2456-2465]

ESM Table 5: Characteristics of included studies

Author, year	Country, setting, time period	Study design, Follow-up	Sex, mean age, type of diabetes	Number of participants and cases	Outcome	Outcome assessment	Relevant exposure	Exposure assessment	Adjustment factors
Abe, 2020 [2466]	USA<br>Hospitals-based<br>Grady Memorial Hospital, Georgia,<br>1st April to 30th May 2020	Retrospective study, ND	m/w, 56 years, ND	71 participants,<br>52 severe COVID cases	Severe COVID (Composite cardiovascular complications)	Electronic medical records	Sex, HbA_{1c}	Medical records	Unadjusted
Acharya, 2020 [2467]	South Korea<br>Hospitals-based<br>Dongguk University Gyeongju Hospital or Andong Medical Center, Gyeong-sangbuk-do, 18th February to 30th June 2020	Retrospective study, ND	m/w, 69.8 years, T2D	55 participants,<br>11 deaths	Death	Medical records	Age, BMI, sex, smoking, hypertension, CVD, cerebrovascular disease, dementia, cancer, blood glucose, HbA_{1c}	Medical records	Age
Agarwal, 2020 [2468]	USA<br>Hospitals-based<br>Montefiore Medical Center, 11th March to 7th May 2020	Retrospective study, ND	m/w, 67.9 years, T1D & T2D	1,279 participants,<br>394 deaths	Death	Electronic health records	Insulin, CVD	Electronic health records	Age, sex, BMI, insurance, HbA_{1c}, insulin treatment, non-insulin treatment, hypertension, COPD, CVD, CKD
Aghaaliakbari, 2020 [2469]	Iran<br>Hospitals-based<br>Firoozgar Hospital	Prospective study, 8 days	m/w, 64.4 years, ND	153 participants,<br>40 deaths	Death	Medical records	Age, ACE inhibitors, coronary artery disease	Medical records	Age, ACE inhibitors, Coronary Artery Disease, Immunosuppression, AKI, ICU Admission

	1st March to 30th June 2020								
Ahmed, 2021 [2470]	UK<br>Hospitals-based<br>Brighton and Sussex University Hospitals (BSUH): Royal Sussex County Hospital and Princess Royal Hospital<br>10th March to 30th June 2020	Retrospective study,<br>28 days	m/w,<br>76 years,<br>T1D & T2D	140 participants,<br><br>42 deaths	Death	Electronic medical records	Sex, asthma, COPD, heart disease, cancer, hypertension, CKD, diabetes type, average glucose, HbA_{1c}	Electronic medical records	Unadjusted
Alhakak, 2021 [2471]	Denmark<br><br>National register data<br><br>26th February 2020 to 31st December 2020	Retrospective study,<br>30 days	m/w,<br>74,3 years,<br>ND	1,178 participants,<br><br>272 deaths,<br>512 severe COVID cases	Death, Severe COVID (Severe COVID, ICU admission or death)	Danish National Patient Registry	HbA_{1c} , sex, age, ischemic heart disease, hearth failure, atrial fibrillation, stroke, hypertension, COPD, cancer, CKD, statins, beta-blockers, Calcium channel blockers, RAS inhibitor, acetylsalicylic acid, anticoagulants	Danish National Patient Registry	Age, sex, ischemic heart disease, heart failure, atrial fibrillation, stroke, peripheral artery disease, hypertension, COPD, cancer, CKD, cholesterol-lowering drugs, beta-blockers, calcium channel blockers, RAS inhibitors, acetylsalicylic acid, anticoagulants
Alrashed, 2021 [2472]	Saudi Arabia<br>Hospitals-based<br><br>King Fahad Medical City and Prince Mohammed Bin Abdulaziz hospital, Riyadh, April to June	Retrospective study,<br>ND	m/w,<br>46.9 years (for the entire population, na for participants with diabetes),<br>ND	126 participants,<br><br>103 severe COVID cases	Severe COVID	Electronic medical records	ACEI/ARB	Electronic medical records	Age, sex, BMI, hypertension, renal disease, number of comorbidities

Al Hayek, 2020 [2473]	Saudi Arabia<br>Hospitals-based<br><br>Prince Sultan Military Medical City, Riyadh Saudi Arabia, May to July 2020	Retrospective study, ND	m/w, 57.6 years, T2D	806 participants,<br><br>387 severe COVID cases	Severe COVID (Hospitalisation)	Electronic medical records	Age, HbA_{1c}, CVD, hypertension, cerebrovascular disease, chronic pulmonary disease, cancer, CKD, insulin, metformin, DPP-4 inhibitors	Electronic medical records	unadjusted
Aon, 2022 [2474]	Kuwait<br>Hospitals-based<br><br>Jahra Hospital, Kuwait, November 2020 to September 2021	Retrospective study, 28 days	m/w, 59.4 years, T1D & T2D	395 participants,<br><br>118 severe COVID cases	Severe COVID (ICU admission, IMV or in-hospital death)	Electronic medical records	Age, sex, creatinine, lymphocytes, LDH, d-dimer, haemoglobin	Electronic medical records	Age, sex, creatinine, lymphocytes, LDH, d-dimer, haemoglobin, stress hyperglycaemia ratio
Assaad, 2022 [2475]	USA<br>Hospitals-based<br><br>11 hospitals affiliated with Northwell Health, New York state, March to October 2020	Retrospective study, ND	m/w, 57 years, T2D	696 participants,<br><br>86 deaths	Death	Electronic medical records	Age, sex, BMI, insulin	Electronic medical records	Age, ethnicity, BMI, acute kidney injury, sex, intubation, insulin, steroid use, hydroxychloroquine, azithromycin, toclizumab, plasma, pulmonary embolism
Bello-Chavolla, 2020 [2476]	Mexico<br>National register data<br><br>Mexican epidemiologic surveillance database, up to May 18th 2020	Retrospective study, 30 days	m/w, 57.2 years, ND	9,460 participants,<br><br>2,062 deaths	Death	Open source dataset of the General Directorate of Epidemiology of the Mexican Ministry of Health	Age, sex, smoking, COPD, asthma, CKD	Open source dataset of the General Directorate of Epidemiology of the Mexican Ministry of Health	Age, sex, smoking, CVD, immunosuppression, COPD, asthma, CKD, hypertension, obesity

Barrett, 2021 [2477]	USA<br>Hospitals-based<br><br>842 hospitals that contributed data to the Premier Healthcare Database Special COVID-19 Release, March to November 2020	Retrospective study,<br>ND	m/w,<br>ND,<br>T1D & T2D	112,692 participants,<br><br>20,717 deaths, 59,831 severe COVID cases	Death, Severe COVID	Premier Healthcare Database Special COVID-19 Release (PHD-CSR)	Diabetes type	Premier Healthcare Database Special COVID-19 Release (PHD-CSR)	Age, sex, ethnicity, payer type, census region, hospital area, admission month, diabetic ketoacidosis
Borzouei, 2021 [2478]	Iran<br>Hospitals-based<br><br>Sina Hospital and Beheshti Hospital, Hamadan province, January 2020 to January 2021	Retrospective study,<br>ND	m/w,<br>65.2 years,<br>ND	420 participants,<br><br>85 deaths	Death	Electronic medical records	Smoking, residence, blood glucose, hypertension, pulmonary diseases	Electronic medical records	Smoking and residence adjusted for age, residence, smoking status, addiction, fatigue, diarrhoea, chills, anorexia, dyspnoea, blood urea, prothrombin time, glutamic-oxaloacetic transaminase, alkaline phosphatase<br><br>Blood glucose, hypertension and pulmonary diseases unadjusted
Boye, 2021 [2479]	USA<br>Registry data<br><br>UnitedHealth Group (UHG) Clinical Discovery Database, 15th January to 15th July 2020	Retrospective study,<br>ND	m/w,<br>71.6 years,<br>T2D	9,531 participants,<br><br>1,002 deaths 5,263 severe COVID cases	Death, Severe COVID (Hospitalisation)	UnitedHealth Group (UHG) Clinical Discovery Database	Age, sex, CVD, myocardial infarction, heart failure, peripheral vascular disease, CKD, COPD, cancer, dementia, immunosuppressants, insulin, metformin, sulfonylurea, anticoagulants, RAS inhibitor	UnitedHealth Group (UHG) Clinical Discovery Database	Age, sex, ethnicity, geographic region, income, insurance type, charlosn comorbidity index, chronic pulmonary disease, hear failure, renal disease, CVD, CKD, neuropathy, peripheral vascular disease, metformin, sulfonylurea, thiazolidinediones, GLP-1 receptor agonists, DPP-4 inhibitors, SGLT-2 inhibitors, insulin, HbA_{1c}
Calapod, 2021 [2480]	Romania<br>Hospitals-based	Prospective study,<br>16 days	m/w,<br>66.3 years,<br>T2D	138 participants,	Severe COVID	Medical records	Sex, obesity, ferritin	Medical records	Sex, BMI, dyspnoea, ferritin, CRP, AST, ALT and FIB-4

	Bucharest Emergency University Hospital,<br>October 2020 to February 2021			88 severe COVID cases					
Cao, 2021 [2481]	China<br>Hospitals-based<br>Huoshenshan hospital, Wuhan, 4th February to 23rd March 2020 and followed up until 31st March	Retrospective study, ND	m/w, ND, ND	231 participants, ND	Severe COVID (severe pneumonia)	Electronic medical records	BMI	Electronic medical records	Age, sex, comorbidity score
Cariou, 2020 (a) [2482]	France<br>Hospitals-based<br>All French hospitals, 10th March to 10th April 2020,<br>CORONADO study	Prospective study, 7 days	m/w, 69.8 years, T1D & T2D	1,317 participants,<br>140 deaths, 382 severe COVID cases	Death, severe COVID (MV and/or death)	Medical files	Type of diabetes, HbA_{1c} , diabetic retinopathy, CKD, diabetic foot, ischemic heart disease, cerebrovascular disease	Medical files, if needed, general or specialist practitioner, regular pharmacist or biomedical laboratory	Univariate and different multivariate models available e.g. age, sex, hypertension, microvascular diseases, macrovascular disease, heart failure, cancer, treated OSA, β-blocker, metformin, insulin, loop diuretics, ARBs and/or ACE inhibitors and/or MRAs
Cariou, 2020 (b) [2483]	France<br>Hospitals-based<br>68 French hospitals, 10th March to 10th April 2020,<br>CORONADO study	Prospective study, 28 days	m/w, 70.9 years, T2D	2,449 participants,<br>514 deaths	Severe COVID (MV and/or death)	Medical files	Statin	Medical files, if needed, general or specialist practitioner, regular pharmacist or biomedical laboratory	Age, sex, ethnicity, BMI, arterial hypertension, history of micro- or macrovascular diabetes complications, heart failure, treated obstructive sleep apnoea, COPD, metformin, DPP-4 inhibitors, GLP-1 receptor agonists, insulin, ezetimibe, RAS-blockers
Chai, 2022 [2484]	China<br>Hospitals-based<br>Headquarter, West Hospital,	Retrospective study, 365 days (until 17th March 2021)	m/w, ND, ND	439 participants,<br>63 deaths	Death (1-year mortality)	Electronic medical records	Age, blood glucose	Electronic medical records	Age, blood glucose, hyperuricemia

	and Tumor center of Union Hospital, Central Hospital of Wuhan, Dongfeng Hospital, Hubei Province, 1st January to 18th March 2020								
Charoenngam, 2021 [2485]	USA<br>Hospitals-based<br><br>Boston University Medical Center hospital,<br>1st March to 4th August 2020	Retrospective study,<br>ND	m/w,<br>64.2 years,<br>T2D	458 participants,<br><br>61 deaths,<br>70 intubation	Death,<br>Severe COVID (Intubation)	Electronic medical records	Blood glucose	Electronic medical records	Age, sex, BMI, smoking, alcohol use, hypertension, dyslipidaemia, CHD, cerebrovascular disease, COPD, asthma, CLD, end-stage renal disease, cancer, HIV infection, heart failure
Chen, 2020 (a) [2486]	China<br>Hospitals-based<br><br>Central Hospital of Wuhan,<br>1st January to 17th March 2020	Retrospective study,<br>ND	m/w,<br>66.0 years,<br>ND	136 participants,<br><br>26 deaths,<br>93 severe COVID cases	Death,<br>severe COVID (poor prognosis)	Electronic medical records, CT, evaluation by experienced clinicians	Sex, hypertension, CVD, CKD, lymphocytes, albumin, ALT, AST, LDH, urea, creatinine, blood glucose, CRP, d-dimer, metformin, insulin, sulfonylureas, DPP4 inhibitor, α-glycosidase	Electronic medical records	Analyses for sex, hypertension, CVD, CKD, lymphocytes, ALT, AST, LDH, urea and d-dimer unadjusted<br><br>Analyses for albumin, creatinine, glucose, CRP, metformin, insulin, sulfonylureas and DPP4 inhibitors adjusted for age, albumin, creatinine, glucose, CRP, and usage of a specific medication (yes/no)
Chen, 2020 (b) [2487]	China<br>Hospitals-based<br><br>Wuhan Jinyintan Hospital,<br>25th December 2019 to 3rd March 2020	Retrospective study,<br>7 days	m/w,<br>63.4 years,<br>T2D	138 participants,<br><br>49 deaths	Death	Electronic medical records	D-dimer, antiplatelet and anticoagulant therapy, number of complications	Electronic medical records	Age, sex, hypertension, chronic lung disease, chronic liver disease, chronic kidney disease, coronary heart disease, malignant tumour, antibiotic and antiviral therapy<br>Analyses for number of complications: unadjusted

Chen, 2022 (c) [2488]	China, Hospitals-based<br><br>Huangshi Central Hospital, Huangshi Hospital of Traditional Chinese Medicine, Huangshi Youse Hospital, February to April 2020	Retrospective, study, ND	m/w, 61 years, T2D	85 participants,<br><br>23 severe COVID cases	Severe COVID	Electronic medical records	Age, albumin, lymphocytes, white blood cells, haemoglobin, CRP, IL-6	Electronic medical records	Arm medium circumference, albumin, prealbumin, national risk screening-2002 score, lymphocytes, PaO_2/FiO_2 ratio
Cheng, 2020 [2489]	China Hospitals-based<br><br>General Hospital of Central Theater Command, December 2019 to February 2020	Retrospective study, ND	m/w, 63 years, T2D	103 participants,<br><br>ND	Severe COVID	Electronic medical records	HbA_{1c}	Electronic medical records	Age, sex, ALT, urea, creatinine, HbA1c, LDH, CRP, IL-6, PCT, neutrophil count, lymphocyte count, CD3+T cells, and CD16+CD56 cells
Choi, 2020 [2490]	South Korea Health insurance data<br><br>South Korean population, up to 15th May 2020	Retrospective study, ND	m/w, ND, T1D & T2D	566 participants,<br><br>68 deaths, 94 severe COVID cases	Death, severe COVID (Severe COVID or death)	HIRA database	ARB/ACEI	HIRA database	Age, sex, region of hospitals, chronic lung disease, major neurologic diseases, Charlson comorbidity index, treatment modalities, presence of severe infection
Chung, 2020 [2491]	South Korea Hospitals-based<br><br>Yeungnam University Medical Center in Daegu,	Retrospective study, 28 days	m/w, 66.3 years, ND	29 participants,<br><br>13 severe COVID cases	Severe COVID (Severe and critical outcome)	Electronic medical records	Age, HbA_{1c}	Electronic medical records	Age, sex, smoking, HbA_{1c} , serum glucose levels

	time period not specified								
Corcillo, 2020 [2492]	UK<br>Hospitals-based<br>12th March to 7th April 2020	Retrospective study,<br>ND	m/w,<br>68 years,<br>T1D & T2D	187 participants,<br>49 severe COVID cases	Severe COVID (Intubation)	Hospital medical records	Retinopathy	NHS Diabetic Eye Screening data	Age, BMI, duration of diabetes, macrovascular complications, CKD, retinopathy
Crouse, 2020 [2493]	USA<br>Hospitals-based<br><br>University of Alabama at Birmingham Hospital,<br>25th February to 22nd June 2020	Retrospective study,<br>ND	m/w,<br>ND,<br>T1D & T2D	239 participants,<br><br>45 deaths	Death	Electronic medical records	Age, ethnicity, sex, obesity, hypertension, diabetes type, insulin, metformin	Electronic medical records	Age, sex, hypertension, BMI, metformin, insulin, ethnicity (mutual adjustment for the covariates)
Dalan, 2020 [2494]	Singapore<br>Hospitals-based<br><br>National Centre of Infectious diseases,<br>up to 15th April 2020	Retrospective study,<br>ND	m/w,<br>ND,<br>T2D	76 participants,<br><br>ND	Severe COVID (MV)	Medical records	DPP-4 inhibitors, SGLT-2 inhibitors, sulfonylureas	Medical records	Age, sex, BMI, statin, ethnicity, HbA1c, antihypertensive medication, diabetes medication, SBP, DBP
de Abajo, 2020 [2495]	Spain<br>Hospitals-based<br><br>Seven hospitals in Madrid,<br>1st March to 24th March 2020	Case-population study, ND	m/w,<br>69.1 years (for the entire population, na for participants with diabetes),<br>ND	1,440 participants,<br><br>182 severe COVID cases	Severe COVID (Admission to hospital)	Electronic primary health-care records	RAS inhibitors	Hospital medical records	Age, sex, hypertension, COPD, asthma, dyslipidaemia, cerebrovascular disease, cancer, ischemic heart disease, heart failure, atrial fibrillation, thromboembolic disease, chronic renal failure
Demirci, 2022 [2496]	Turkey<br>National register data	Retrospective study,<br>ND	m/w,<br>41 years,<br>T1D & T2D	652 participants,<br><br>60 deaths,	Death, Severe COVID (ICU	Electronic medical records	Diabetes type	Electronic medical records	Age, sex, HbA_{1c} , CT findings of COVID-19, hypertension, Asthma/ COPD, lymphopenia

	National Electronic Database of the Turkish Ministry of Health, 11th March to 30th May 2020			91 severe COVID cases	admission and/or intubation)				
De Souza, 2022 [2497]	Brazil<br><br>National register data<br><br>SIVEP-Gripe	Prospective study, ND	m/w, ND, ND	114,144 participants,<br><br>51,378 deaths	Death	SIVEP-Gripe	Ethnicity	SIVEP-Gripe	Region, ethnicity, ICU admission, IMV, fever, cough, odynophagia, dyspnoea, respiratory distress, SpO_2, diarrhoea, ageusia
Djuric, 2022 [2498]	Italy<br>National register data<br><br>Residents of the Reggio Emilia Province in the COVID-19 Surveillance Registry by the Italian National Institute of Health, 26th February to 10th August 2021	Retrospective study, 45 days	m/w, 50 years, T2D	2,570 participants,<br><br>317 deaths	Death	COVID-19 Surveillance Registry	Obesity, diabetes duration, HbA_{1c}, hypertension, CVD, coronary artery disease, heart failure, cerebrovascular disease, CKD, COPD, cancer, dementia, OAD, OAD + insulin, insulin, triglycerides	COVID-19 Surveillance Registry	Immigration background, CCI, COPD, Ischemic cardiopathy, dementia, CKD, cancer, hypertension, obesity, heart failure, arrhythmias, vascular diseases, cerebrovascular disease, triglycerides, HbA_{1c}, diabetic drugs
Do, 2020 [2499]	South Korea<br>Health insurance data<br><br>South Korean population, up to 15th May 2020	Retrospective study, ND	m/w, 61 years, T2D	1,865 participants,<br><br>150 deaths, 85 severe COVID cases	Death, severe COVID (MV)	HIRA database	Analyses for death: Hypertension<br><br>Analyses for MV: Age, sex, insulin, metformin, CCI Score, hypertension	HIRA database	Age, sex, CCI score, hypertension<br><br>Analyses for Insulin: unadjusted
Duan, 2021 [2500]	China<br>Hospitals-based<br><br>Huoshenshan Hospital, Wuhan	Retrospective study, 17 days	m/w, 66 years, T2D	206 participants,<br><br>ND	Severe COVID (ICU or in-hospital death)	Electronic medical records	Blood glucose	Electronic medical records	Age, sex, severity classification on admission, hypertension, CVD, COPD, comorbidities, antivirus

	4th February to 30th March 2020								drugs, convalescent plasma transfusion therapy, steroids
Elibol, 2021 [2501]	Turkey<br>Hospitals-based<br><br>Kaysari City Training and Research Hospital,<br>1st March to 15th September 2020	Retrospective study, ND	m/w, 63.3 years, T2D	432 participants,<br><br>91 deaths	Death	Electronic medical records	Age, diabetes duration, coronary artery disease, blood glucose, CRP, LDH, ferritin, d-dimer, fibrinogen	Electronic medical records	Age, diabetes duration, asthma, coronary artery disease, CKD, apache II, glasgow coma scale, blood glucose, CRP, urea, LDH, ferritin, d-dimer, fibrinogen
Emami, 2021 [2502]	Iran<br>Hospitals-based<br><br>Hospitals affiliated with Shiraz University of Medical Sciences, Fars province,<br>25th February to 10th July 2020	Retrospective study, ND	m/w, 61.3 years, ND	458 participants,<br><br>50 deaths	Death	Hospital medical records	Sex, hypertension, CVD, cancer, CKD, liver disease	Hospital medical records	Age, sex, hypertension, CVD, cancer, CKD, chronic liver disease, primary immunodeficiency
Emral, 2021 [2503]	Turkey<br>National register data<br><br>Turkish Ministry of Health National Electronic Database,<br>11th March to 30th May 2020	Retrospective study, ND	m/w 54 years, T2D	33,478 participants,<br><br>2,565 deaths	Death	Turkish Ministry of Health National Electronic Database	Age, sex, hypertension, dyslipidaemia, obesity, asthma/COPD, heart failure, CKD, microvascular complications, CVD, cancer, RAS inhibitors, statins, acetylsalicylic acid, insulin, HbA_{1c}, DPP-4 inhibitors	Turkish Ministry of Health National Electronic Database	Age, sex, micro- and macrovascular complications, ACEIs/ARBs, statins, insulin, metformin, other OADs
Erol, 2022 [2504]	Turkey<br>Hospitals-based	Retrospective study, 12 days	m/w 62.5 years, T2D	217 participants,	Death,	Electronic medical records	DPP-4 inhibitors	Electronic medical records	Age, sex, BMI

	Sisli Hamidiye Etfal Training and Research Hospital,<br>March to October 2020			24 deaths,<br>31 severe COVID cases	Severe COVID (MV)				
Fernandez -Pombo, 2021 [2505]	Spain<br>Hospitals-based<br>Healthcare area of Santiago de Compostela and Barbanza,<br>March to May 2020	Retrospective study,<br>ND	m/w,<br>71.6 years,<br>T1D & T2D	136 participants,<br>62 cases	Severe COVID (Hospitalis ation)	Electronic medical records	Age, sex, hypertension, obesity, ischemic cardiopathy, stroke, sleep apnoea, smoking, HbA1c	Electronic medical records	Age, sex, hypertension, obesity, ischemic cardiopathy, stroke, atherosclerosis, sleep apnoea, smoking, HbA_{1c}
Ferraninni, 2022 [2506]	Sweden<br>National register data<br>Swedish National Patient Registry linked with Cause of Death Registry,<br>1st February 2020 to 15th May 2021	Retrospective study,<br>30 days	m/w,<br>72 years,<br>T2D	9,538 participants,<br>2,145 deaths	Death	Cause of Death Registry	SGLT-2 inhibitor, DPP-4 inhibitors, GLP-1 receptor agonists,	Swedish National Patient Registry	PSM with age, sex, having children, country of birth, income, education, living alone, region, obesity, and several comorbidities (e.g. COPD, heart failure, liver disease) and pharmacological therapies (e.g. antiplatelet and anticoagulants, insulin, metformin)
Fox, 2020 [2507]	USA<br>Hospitals-based<br>Einstein Medical Center, Philadelphia,<br>1st March to 24th April 2020	Retrospective study,<br>ND	m/w,<br>66.4 years,<br>ND	166 participants,<br>45 deaths	Death	Hospital medical records	Age, sex, BMI, ethnicity, COPD, asthma, heart failure, atrial fibrillation, coronary artery disease, hypertension, CKD	Hospital medical records	Age, sex, BMI, ethnicity, COPD, asthma, heart failure, coronary artery disease, hypertension, atrial fibrillation, CKD

Fu, 2022 [2508]	China<br>Hospitals-based<br><br>Tianyou Hospital, at the Wuhan University of Science and Technology, 8th January to 7th March 2020	Retrospective study, 19 days	m/w, 68 years, T2D	108 participants,<br><br>16 deaths	Death	Electronic medical records	Age, diabetes duration, CVD, serum amyloid A, blood glucose	Electronic medical records	Age, serum amyloid A and blood glucose adjusted for age, serum amyloid A, blood glucose and diabetes treatment<br><br>Diabetes duration and CVD unadjusted
Fuku-shima, 2022 [2509]	Japan<br>Hospitals-based<br><br>Japan COVID-19 Task Force (>70 hospitals),<br>February 2020 to October 2021	Retrospective study, ND	m/w, 63.2 years, ND	562 participants,<br><br>106 severe COVID cases	Severe COVID	Electronic medical records	Age, sex, BMI, smoking, hypertension, CVD, chronic liver disease, CKD, HbA_{1c}	Electronic medical records	HbA1c adjusted for age, sex, BMI, smoking, hypertension, CVD, CKD<br><br>All other phenotypes unadjusted
Ghany, 2021 [2510]	USA<br>Hospitals-based<br><br>Medical Centers in 8 US states, 1st January to 14th August 2020	Retrospective study, ND	m/w, ND, ND	593 participants,<br><br>59 deaths, 102 severe COVID cases	Death, severe COVID (ARDS)	Electronic medical records	Metformin	Electronic medical records	Age, sex, CCI score, hypertension, ejection fraction
Giorda, 2021 [2511]	Italy<br>Hospitals-based<br><br>Network of diabetes clinics of Piedmont, 21st February to 31st May 2020	Retrospective study, 30 days	m/w, ND, T2D	1,882 participants,<br><br>512 deaths, 236 severe COVID cases	Death, Severe COVID (ICU admission)	Electronic medical records	Age, sex, smoking, BMI, OAD, insulin, HbA_{1c}, neuropathy, retinopathy, coronary heart disease, heart failure, hypertension, cerebrovascular disease, COPD, CKD, cancer, diabetic foot, liver disease	Electronic medical records	Age, sex, education, diabetes duration, smoking, BMI, OAD, insulin, HbA_{1c}, neuropathy, retinopathy, coronary heart disease, heart failure, hypertension, cerebrovascular disease, COPD, CKD, cancer, diabetic foot, liver disease

Gregory, 2020 [2512]	USA<br>Hospitals-based<br><br>Epic Clarity data warehouse at Vanderbilt University Medical Center, 17th March to 7th August 2020	Prospective study,<br>14 days	m/w,<br>32 years,<br>T1D	37 participants,<br><br>9 severe COVID cases	Severe COVID	Electronic medical records	Age, sex, BMI, HbA_{1c}, diabetes duration, CKD, retinopathy, neuropathy, RAS inhibitors, hypertension, asthma	Electronic medical records	Age, sex, BMI, smoking, race, hypertension
Hadjadj, 2022 [2513]	International (USA and UK)<br>Hospitals-based<br><br>Northwell Health System hospitals in the New York area (AMERICADO – USA),<br>1st January to 31st May 2020<br>and the ABCD COVID-19 audit (UK),<br>up to 8th December 2020	Retrospective study,<br>ND	m/w,<br>68 years,<br>T2D (USA),<br>74.3 years,<br>T1D & T2D (UK)	7,367 participants,<br>1,251 deaths (USA)<br><br>1,809 participants,<br>678 deaths (UK)	Death	Electronic medical records<br><br>AMERICADO (USA),<br>ABCD COVID-19 (UK)	Microvascular complications	Electronic medical records<br><br>AMERICADO (USA),<br>ABCD COVID-19 (UK)	Age, sex, CVD, hypertension
Hammad, 2021 [2514]	Egypt<br>Hospitals-based<br><br>Mansoura University Hospital,<br>July to November 2020	Retrospective study,<br>ND	m/w,<br>63.1 years,<br>T2D	118 participants,<br><br>45 deaths	Death	Electronic medical records	Age, sex, albumin, ALT	Electronic medical records	Age, sex, albumin, ALT, bilirubin, arterial oxygen partial pressure

Harris, 2022 [2515]	International (Spain and UK)<br>Hospital-based<br><br>Six hospitals in the HM Hospitales group (Spain) and the ABCD COVID-19 audit (UK)	Retrospective study,<br>ND	m/w,<br>74.7 years, T1D & T2D (Spain),<br>71.7 years, T1D & T2D (UK)	406 participants,<br>79 deaths (Spain),<br><br>1,849 participants,<br>676 deaths (UK)	Death	Electronic medical records<br><br>HM hospitals group (Spain),<br>ABCD COVID-19 (UK)	RAS inhibitors, statin	Electronic medical records<br><br>HM hospitals group (Spain),<br>ABCD COVID-19 (UK)	Age, sex, diabetes type, macrovascular complications, hypertension, CKD
Heald, 2022 [2516]	UK<br>Hospitals-based<br><br>Greater Manchester Care Record (GMCR) database,<br>1st January 2020 to 31st May 2021	Retrospective study,<br>ND	m/w,<br>39,4 years (T1D) &<br>63,1 years (T2D),<br>T1D & T2D	14,087 participants,<br><br>2,246 severe COVID cases	Severe COVID (Hospitalis ation)	Greater Manchester Care Record (GMCR) database	Age, sex, BMI, smoking, ethnicity, HbA1c, metformin, hypertension, COPD, asthma, acetylsalicylic acid , ACEI, cholesterol, LDL, HDL, eGFR	Greater Manchester Care Record (GMCR) database	Unadjusted
Huang, 2020 [2517]	China<br>Hospitals-based<br><br>Central Hospital of Wuhan,<br>1st January to 24th March 2020	Retrospective study,<br>ND	m/w,<br>66 years,<br>ND	256 participants,<br><br>54 deaths,<br>107 severe COVID cases	Death, severe COVID	Electronic medical records	Analyses for Death:<br>Age, cerebrovascular disease, CKD, leukocytes, neutrophils<br><br>Analyses for severe COVID:<br>Cerebrovascular disease, CHD, COPD, neutrophils lymphocytes,<br>D-dimer, serum urea, LDH, creatinine kinase, procalcitonin	Electronic medical records	Analyses for leukocytes, neutrophils and death:<br>Age, cerebrovascular disease, chronic renal disease, bilateral pneumonia, multiple military mottling and ground-glass opacity, immunoglobulin, non-invasive mechanical ventilation<br><br>All other analyses: unadjusted
Hui, 2020 [2518]	China<br>Hospitals-based<br><br>Tongji Hospital, Wuhan,	Retrospective study,<br>ND	m/w,<br>71 years,<br>ND	55 participants,<br><br>44 deaths	Death	Electronic medical records	Age, control of hyperglycaemia	Electronic medical records	Age, neutrophils, lymphocytes, platelets, LDH, serum creatinine, urea nitrogen, troponin, n terminal pro brain natriuretic peptide, procalcitonin, CRP, IL-2

	28th January to 10th March 2020								receptor, IL -6, IL-10, TNF-α, control of hyperglycemia
Ikram, 2022 [2519]	South Africa<br>Hospitals-based<br><br>King Edward VIII Hospital, KwaZulu-Natal, 1st June to 31st September 2020	Retrospective study, ND	m/w, ND, ND	79 participants,<br><br>21 deaths, 16 severe COVID cases	Death, severe COVID (MV)	Electronic medical records	Blood glucose	Electronic medical records	Unadjusted
Iqbal, 2021 [2520]	UK<br>Hospitals-based<br><br>Sheffield Teaching Hospitals (Northern General and Royal Hallamshire Hospitals),<br>29th February to 1st May 2020	Retrospective study, ND	m/w, 71.8 years, T1D & T2D	156 participants,<br><br>54 deaths	Death	Electronic medical records	Age, sex, obesity, HbA_{1c}, blood glucose	Electronic medical records	Age, BMI, index of multiple deprivation, respiratory rate at admission, albumin, sodium, potassium, urea, creatinine, neutrophil-lymphocyte ratio, activated partial thromboplastin time, use of anticoagulants
Izzi-Engbeaya, 2020 [2521]	UK<br>Hospitals-based<br><br>Charing Cross Hospital, Hammersmith Hospital and St Mary's Hospital, London,<br>9th March to 22nd April 2020	Retrospective study, 30 days	m/w, 68.5 years, T1D & T2D	337 participants,<br><br>48 severe COVID cases	Severe COVID (Death/ ICU)	Electronic medical records	Age, sex, ethnicity, diabetes type, diabetes duration, HbA_{1c}, blood glucose, hypertension, insulin, GLP-1 receptor antagonist, metformin, sulphonylurea, SGLT-2 inhibitor, DPP-4 inhibitors, ischemic heart disease, eGFR, stroke, active foot disease, dyslipidaemia, heart failure, COPD, cancer, statin, RAS inhibitors, antiplatelet drug, white cell count,	Electronic medical records	Age, sex, diabetes type, duration of diabetes, insulin, GLP-1 receptor antagonist, metformin, sulphonylurea, SGLT-2 inhibitor, DPP-4 inhibitors, total number of medications, HbA_{1c}, glucose, Average CBG in first 72hr after COVID-19 diagnosis, ischemic heart disease, eGFR, hypertension, ethnicity, stroke, active foot disease, hyperlipidaemia, heart failure, COPD, active cancer, statin, RAS

							haemoglobin, platelet count, neutrophils, lymphocytes, CRP		inhibitors, antiplatelet drug, white cell count, haemoglobin, platelet count, neutrophils, lymphocytes, serum sodium, CRP, capillary blood glucose, temperature, respiratory rate on diagnosis, heart rate, systolic blood pressure, diastolic blood pressure, NEWS, inspired oxygen, oxygen saturations, maximum inspired oxygen required<br><br>Analyses for duration of diabetes, SGLT-2 inhibitor, HbA1c, blood glucose and eGFR:<br>unadjusted
Jayaswal, 2021 [2522]	India<br>Hospitals-based<br><br>Sardar Vallabh Bhai Patel COVID Hospital,<br>13th July to 14th October 2020	Retrospective study,<br>ND	m/w,<br>ND,<br>ND	238 participants,<br><br>29 deaths,<br>62 severe COVID cases	Death, severe COVID	Electronic medical records	Hypertension	Electronic medical records	Age
Kabootari, 2021 [2523]	Iran<br>Hospitals-based<br><br>Tertiary referral center in Golestan province,<br>February to August 2020	Retrospective study,<br>ND	m/w,<br>61,8 years,<br>T2D	560 participants,<br><br>165 Death	Death	Electronic medical records	Age, area of residence, blood glucose, hypertension, stroke, pulmonary disease, cognitive impairment, OADs, insulin, OADs and insulin, beta-blocker, RAS inhibitors, statins, eGFR, creatinine, white blood cells, neutrophils, lymphocytes, platelets, LDH	Electronic medical records	Age, sex, area of residence, cognitive impairment, stroke, pulmonary disease, hypertension, OADs, insulin, OADs and insulin, beta-blocker, RAS inhibitors and statins are adjusted for demographic data (e.g. age, sex, BMI), diabetes-related complications and drug history

									White blood cells, neutrophils, lymphocytes, platelets, LDH, creatinine, eGFR and blood glucose are adjusted for demographic data, diabetes-related complications, drug history, vital signs, pulse oximetry data and laboratory test data
Kang, 2021 [2524]	South Korea<br>Medical insurance data<br><br>South Korea Disease Control and Prevention Agency (KDCA), 19th January to 30th April, 2020	Retrospective study,<br>ND	m/w,<br>ND,<br>ND	501 participants,<br><br>ND	Death	South Korea Disease Control and Prevention Agency (KDCA)	BMI	South Korea Disease Control and Prevention Agency (KDCA)	Age, sex, hypertension, CKD, cancer, dementia
Khalili, 2020 (a) [2525]	Iran<br>Hospitals-based<br><br>Imam Hossein Medical Center, Tehran, February to May 2020	Retrospective study,<br>90 days	m/w,<br>66.4 years,<br>T2D	127 participants,<br><br>29 deaths	Death	Electronic medical records	Age, Charlson Index, diabetic foot infection, insulin	Electronic medical records	Age, Charlson Index, diabetic foot infection, insulin
Khalili, 2021 (b) [2526]	Iran<br>Hospitals-based<br><br>Imam Hossein Medical Center, Tehran, February to May 2020	Retrospective study,<br>90 days	m/w,<br>66.4 years,<br>T2D	127 participants,<br><br>36 severe COVID cases	Severe COVID (AKI)	Electronic medical records	Age, sex, BMI, CVD, hypertension, COPD, statins, ACEI/ARB, diuretics, calcium channel blocker, HbA_{1c}	Electronic medical records	Sex, CVD, ARB/ACEI, diuretics, calcium channel blocker, statins, HbA_{1c}, CT severity score, invasive mechanical ventilation, haematuria, proteinuria
Khunti, 2022 [2527]	UK<br>Hospitals-based	Retrospective study,<br>ND	m/w,<br>72 years,<br>T2D	3,039 participants,<br><br>1,082 deaths	Death	Electronic medical records (ABCD)	SGLT-2 inhibitors	Electronic medical records (ABCD)	Age, sex, ethnicity, blood glucose, insulin, micro- and macrovascular complications

	ABCD-audit (network of 40 centers), up to 8th December								
Kim, 2020 [2528]	South Korea<br><br>Hospitals-based<br><br>5 university hospitals in Daegu,<br>18th February to 31st March 2020	Retrospective study, ND	m/w, 68.3 years, ND	235 participants,<br><br>44 deaths, 65 severe COVID cases	Death, severe COVID	Electronic medical records	Glucose, white blood cells, haemoglobin, platelets, CRP, eGFR, AST, ALT, LDH, insulin, albumin, sulfonylureas, DPP-4 inhibitors, SGLT-2 inhibitors, RAS inhibitors, Metformin,	Electronic medical records	Age, sex
Kristan, 2021 [2529]	USA<br>Hospitals-based<br><br>Hospital system composed of 13 member hospitals,<br>March to 15th June 2020	Retrospective study, ND	m/w, 62 years, T1D & T2D	832 participants,<br><br>151 deaths, 631 severe COVID Cases	Death, severe COVID (hospital admission)	Electronic medical records	Age, sex, BMI, ethnicity, HbA_{1c}, hypertension, dyslipidaemia, coronary artery disease, cerebrovascular disease, microvascular complications, CKD, COPD, OADs, OADs and insulin, insulin, DPP-4 inhibitors	Electronic medical records	Analyses for age, BMI, HbA1c, dyslipidaemia and DPP-4 inhibitors and Death are mutually adjusted<br><br>Analyses for age, health insurance, HbA_{1c}, CKD and DPP-4 inhibitors and severe COVID are mutually adjusted<br><br>All other associations are unadjusted
Lalau, 2020 [2530]	France<br>Hospitals-based<br><br>68 French hospitals,<br>10th March to 10th April 2020,<br>CORONADO study	Prospective study, 28 days	m/w, 70.9 years, T2D	2,449 participants,<br><br>857 severe COVID cases	Severe COVID (MV and/or death)	Medical files	Metformin	Medical files, if needed, general or specialist practitioner, regular pharmacist or biomedical laboratory	Age, sex, BMI, HbA_{1c}, hypertension, diabetic kidney disease, ischemic heart disease, heart failure, cancer, treated OSA, RAS inhibitors, thiazide diuretics, mineralocorticoid receptor antagonists, beta-blockers, sulfonylurea insulin, DPP-4 inhibitors, statins, anti-platelet/ anticoagulant agents, corticosteroids

Lampa-sona, 2020 [2531]	Italy<br>Hospitals-based<br><br>Instituto di Ricovero e Cura a Carattere Scientifico (IRCCS) San Raffaele Hospital, Milan,<br>25th February to 19th April 2020	Retrospective study, 59 days	m/w, ND, ND	139 participants,<br><br>28 deaths	Death	Electronic medical records	Age	Electronic medical records	Age, sex
Laurenzi 2021 [2532]	Italy<br>Hospitals-based<br><br>Instituto di Ricovero e Cura a Carattere Scientifico (IRCCS) San Raffaele Hospital, Milan,<br>25th February to 2nd May 2020	Retrospective study, 222 days	m/w, 71 years, T1D & T2D	121 participants,<br><br>42 deaths	Death	Electronic medical records	BMI, hypertension, coronary artery disease, CKD, COPD, cancer, insulin, metformin, statins, acetylsalicylic acid, RAS inhibitors, beta-blocker, calcium channel blockers, CRP, IL-6, procalcitonin, albumin, ALT, AST, creatinine, white blood cells, neutrophils, lymphocytes, platelets, LDH, d-dimer, haemoglobin, fibrinogen, erythrocyte sedimentation rate, ferritin	Electronic medical records	Age, sex
Lee, 2021 [2533]	South Korea<br>Health insurance data<br><br>South Korean population,<br>19th January to 16th April 2020	Retrospective study, 60 days	m/w, ND, T2D	1,874 participants,<br><br>133 deaths	Death	HIRA database	Statins	HIRA database	Age, sex, hypertension, cancer, COPD, stroke, coronary artery disease, heart failure, CKD

Lei,<br>2020<br>[2534]	China<br>Hospitals-based<br><br>Guangzhou Eighth People's Hospital,<br>15th January 10th March 2020	Retrospective study,<br>ND	m/w,<br>62.5 years,<br>T1D & T2D	24 participants,<br><br>5 severe COVID cases	Severe COVID (ICU admission)	Electronic medical records	Age, sex, hypertension, CVD, blood glucose, HbA_{1c}, platelet count, creatine phosphokinase	Electronic medical records	Analyses for age and HbA1c:<br>Age, respiratory rate, HbA_{1c}, AST<br><br>All other analyses: unadjusted
Leon Pedroza, 2021<br>[2535]	Mexico<br>National register data<br>Mexican epidemiologic surveillance database,<br>28th February to 25th June 2020	Retrospective study,<br>ND	m/w,<br>ND,<br>T2D	33,492 participants,<br><br>ND	Death	SISVER	Obesity, CVD hypertension,	SISVER	Age, sex
Li,<br>2020 (a)<br>[2536]	China<br>Hospitals-based<br><br>Wuhan Union hospital of Tongji Medical College and Jinyintan Hospital,<br>31st December 2019 to 5th April 2020	Retrospective study,<br>ND	m/w,<br>65.0 years,<br>T1D & T2D	132 participants,<br><br>15 deaths,<br>31 severe COVID cases	Death, severe COVID (in-hospital complications)	Electronic medical records	Age, sex, diabetes duration, comorbidities, hypertension, CVD, cancer, cerebrovascular disease, chronic pulmonary disease, CKD, liver disease, lymphocytes, blood glucose	Electronic medical records	Analyses for glucose adjusted for:<br>Age, hypertension, total CT score of the pulmonary involvement, lymphocyte count, fasting plasma glucose, d-dimer, ALT, LDH creatinine, CRP<br><br>Other analyses unadjusted
Li,<br>2020 (b)<br>[2537]	China<br>Hospitals-based<br><br>Wuhan Red Cross Hospital,<br>23rd January to 19th March 2020	Retrospective study,<br>ND	m/w,<br>66.8 years,<br>T2D	131 participants,<br><br>23 deaths	Death	Electronic medical records	Metformin	Electronic medical records	unadjusted
Liu,<br>2020 (a)	China<br>Hospitals-based	Retrospective study, ND	m/w,	64 participants,	Severe COVID	Electronic medical records	HbA1c, blood glucose, CRP, prothrombin time,	Electronic medical records	Plasma glucose at admission

[2538]	Guanggu branch of Tongji Hospital, Wuhan, 3rd to 26th February 2020		66.0 years, ND	12 severe COVID cases	(MV and/or death)		metformin, insulin, α-glucosidase inhibitors, RAS inhibitors, calcium channel blockers, statins		
Liu, 2020 (b) [2539]	China<br>Hospitals-based<br><br>Renmin Hospital, Wuhan, 23rd January to 19th March 2020	Retrospective study, ND	m/w, 64.5 years, T2D	134 participants,<br><br>82 severe COVID cases	Severe COVID	Medical records	Neutrophil- lymphocyte ratio	Medical records	Age, sex, BMI, hypertension
Llanera, 2022 [2540]	UK<br>Hospitals-based<br><br>Secondary and tertiary hospitals in Cheshire and Merseyside regions in the North West of England, 1st January to 30th June 2020	Retrospective study, 7 days	m/w, 74,1 years, T1D & T2D	1,004 participants,<br><br>241 deaths	Death	Electronic health records (ACCREDIT)	Diabetes type, blood glucose, hypertension, CVD, ischemic heart disease, peripheral vascular disease, microvascular complications, CKD, retinopathy, neuropathy, diabetic foot, COPD, insulin, DPP-4 inhibitors, RAS inhibitors	Electronic health records (ACCREDIT)	Unadjusted
Llaurado, 2022 [2541]	International (Spain and UK)<br>Hospitals-based<br><br>Hospital del Mar and Hospital de la Santa Creu I Sant Pau, Barcelona (Spain) and the ABCD COVID-19 audit (UK),	Retrospective study, ND	m/w, 71.1 years, T1D & T2D (Spain), 72.5 years, T1D & T2D (UK)	344 participants, 88 deaths, 59 severe COVID cases (Spain)<br><br>1,846 participants, 715 deaths, 193 severe COVID cases (UK)	Death, Severe COVID (ICU admission)	Electronic medical records<br><br>HM hospitals group (Spain), ABCD COVID-19 (UK)	Macrovascular disease	Electronic medical records<br><br>HM hospitals group (Spain), ABCD COVID-19 (UK)	Age, sex, diabetes type, arterial hypertension, microvascular complications, ethnicity, BMI

	March to October 2020								
Lombardi, 2022 [2542]	USA<br>Hospitals-based<br><br>Montefiore Medical Center, New York City,<br>March to May 2020	Retrospective study, ND	m/w, 68.1 years, T1D & T2D	1,938 participants,<br><br>601 deaths, 271 severe COVID cases	Death, severe COVID (MV)	Electronic medical records	Age, sex, BMI, HbA_{1c}, blood glucose, hypertension, CKD, COPD, CRP, d-dimer	Electronic medical records	Age, sex, ethnicity, BMI, HbA_{1c}, comorbidities, inflammatory markers, corticosteroid therapy
Longmore, 2021 [2543]	International<br>Hospitals-based<br><br>69 hospitals in 11 countries, 17th January to 2nd June 2020	Retrospective study, ND	m/w, ND, T1D & T2D	904 participants for death analysis, 216 deaths<br><br>654 participants for MV analysis, 144 severe COVID cases	Death, severe COVID (MV)	Electronic medical records	BMI	Electronic medical records	Age, sex, CVD, respiratory conditions, hypertension
Lopez-Huamanrayme, 2021 [2544]	Peru<br>Hospitals-based<br><br>Hospital Nacional Alberto Sabogal Sologuren, Callao province, 23rd March to July 2020	Retrospective study, ND	m/w, 63 years, T2D	248 participants,<br><br>97 deaths	Death	Electronic medical records	Age, sex, blood glucose	Electronic medical records	Age, sex, oxygen saturation level on admission, number of prior comorbidities, hyperglycaemia on admission
Luk, 2021 [2545]	Hong Kong<br>Hospitals-based<br><br>Hong Kong Hospital Authority (HA) including all public hospitals	Retrospective study, ND	m/w, 65,3 years, T2D	1,220 participants,<br><br>90 deaths, 235 severe COVID cases	Death, severe COVID (ICU, MV or death)	Electronic medical records	Metformin, sulphonylureas, DPP-4 inhibitors, insulin	Electronic medical records	Age, sex, diabetes duration, smoking, HbA_{1c}, hypertension, coronary heart disease, heart failure, cerebrovascular disease, CKD, chronic liver disease, COPD, cancer, glucose-lowering drugs, statins and

	and general outpatient departments<br><br>23rd January 2020 to 28th February 2021								RAS inhibitors, other glucose-lowering drugs
Ma, 2022 [2546]	USA<br>Hospitals-based<br><br>St Luke's University Health Network (SLUHN)<br><br>16th March 2020 to 15th February 2021	Retrospective study, ND	m/w, ND, T2D	1,356 participants,<br><br>43 deaths	Death	Electronic medical records	Metformin	Electronic medical records	Age, sex, ethnicity, comorbidities, urea, creatinine, ALT, bilirubin
Madaschi, 2022 [2547]	Italy<br>Hospitals-based<br><br>Spedali Civili di Brescia,<br>1st February 2020 to 31st March 2021	Retrospective study, ND	m/w, 72 years, T1D & T2D	291 participants,<br><br>74 deaths	Death	Electronic medical records (Diabsars)	Blood glucose	Electronic medical records (Diabsars)	Age, sex, BMI
Mannucci, 2022 [2548]	Italy<br>Registry data<br><br>COVID-19 surveillance registry of the Padova Local Health Unit (LHU), Veneto region<br>1st March to 31st December 2020	Retrospective study, 90 days	m/w, 69.2 years, T2D	1,923 participants,<br><br>167 deaths, 456 severe COVID cases	Death, severe COVID (Hospitalis ation)	COVID-19 surveillance registry	Age, sex, diabetes duration, hypertension, dyslipidaemia, CVD, CKD, chronic respiratory diseases, cancer, insulin, immunosuppression, metformin, DPP-4 inhibitors, GLP-1 receptor agonists, sulfonylureas, SGLT-2 inhibitors, thiazolidinedione	COVID-19 surveillance registry	Age, sex, diabetes duration, hypertension, dyslipidaemia, CVD, CKD, primary immunodeficiency, acquired immunodeficiency, cancer, chronic respiratory diseases

Mari-muthu, 2022 [2549]	India<br>Hospitals-based<br>Tertiary care center in Bangalore,<br>June to September 2020	Retrospective study, ND	m/w, 56,1 years, T1D & T2D	200 participants,<br>48 deaths	Death	Electronic medical records	Sex, smoking, hypertension, CVD, CKD, chronic pulmonary diseases, cancer	Electronic medical records	Smoking, CKD and CVD are adjusted for smoking, CKD, CVD and breathlessness<br>All other phenotypes are unadjusted
Mehta, 2021 [2550]	USA<br>Hospitals-based<br>University of California San Francisco Medical Center, Northern California,<br>1st January to 31st August 2020	Retrospective study, ND	m/w, 59.2 years, ND	111 participants,<br>8 deaths, 34 severe COVID cases	Death, severe COVID (MV)	Electronic medical records	Age, sex, obesity, HbA_{1c}, blood glucose, insulin	Electronic medical records	Analyses for insulin, HbA_{1c} and blood glucose are adjusted for age and sex<br>All other phenotypes are unadjusted
Meijer, 2021 [2551]	Netherlands<br>Hospitals-based<br>8 hospitals in the CovidPredict Clinical course cohort,<br>3rd March to 1st October 2020	Prospective study, 40 days	m/w, 69.4 years, T2D	565 participants,<br>184 deaths, 111 severe COVID cases	Death, severe COVID (MV)	Electronic medical records (CovidPredict Clinical course cohort)	DPP-4 inhibitors	Electronic medical records (CovidPredict Clinical course cohort)	Age, sex, BMI, comorbidities
Merzon, 2020 [2552]	Israel<br>Health insurance data<br>Leumit Health Services,<br>1st February to 30th April 2020	Retrospective study, ND	m/w, 61.8 years, ND	183 participants,<br>46 severe COVID cases	Severe COVID (Hospitalis ation)	LHS electronic medical records	Age, sex, HbA_{1c}, smoking, dementia, hypertension, ischemic heart disease, cerebrovascular accident, congestive heart failure, chronic pulmonary disease, obesity	LHS electronic medical records	Age, sex, SES, depression/anxiety, schizophrenia, dementia, hypertension, ischemic heart disease, cerebrovascular accident, congestive heart failure, chronic lung disease, obesity
Miguel-Yanes, 2022	Spain	Retrospective study, ND	m/w, 71.7 years, T2D	26,270 participants,	Death	Electronic medical records	Age, sex, MI, heart failure, peripheral vascular disease,	Electronic medical records	Age, MI, heart failure, peripheral vascular disease, cerebrovascular disease,

[2553]	Hospitals-based<br><br>Spanish national Hospital Discharge Database (SNHDD),<br>1st March to 31st December 2020			10,454 deaths			cerebrovascular disease, CKD, liver disease, cancer, dementia		dementia, COPD, rheumatoid disease, liver disease, CKD, cancer, non-invasive MV, invasive MV, ICU admission, sex
Mirani, 2020 [2554]	Italy<br>Hospitals-based<br><br>Humanitas Clinical and Research Hospital, Milan,<br>20th February to 9th April 2020	Retrospective study,<br>ND	m/w,<br>71 years,<br>T2D	90 participants,<br><br>38 deaths	Death	Electronic medical records	Age, sex, blood glucose, obesity, stroke, DPP-4 inhibitors, diuretics, sulfonylureas, antiplatelet / anticoagulants	Electronic medical records	Age, sex<br><br>Analyses for sex: unadjusted
Mohamed, 2021 [2555]	Egypt<br>Hospitals-based<br><br>Zagazig University Hospital,<br>20th May to 20th July 2020	Retrospective study,<br>ND	m/w,<br>57.7 years,<br>T2D	141 participants,<br><br>44 severe COVID cases	Severe COVID (ICU, MV or death)	Electronic medical records	Age, sex, obesity, HbA_{1c}, hypertension, dyslipidaemia, chronic lung disease, CRP, ALT, AST, IL-6, creatinine, white blood cells, lymphocytes, LDH, prothrombin time	Electronic medical records	Age, BMI, hypertension, chronic lung disease, SpO_2, HbA_{1c}, white blood cells, lymphocytes, prothrombin time, IL-6, CRP, LDH, ALT, AST, creatinine, CT severity score<br><br>Analyses for sex and dyslipidaemia are unadjusted
Mondal, 2021 (a) [2556]	India<br>Hospitals-based<br><br>Tertiary care hospital of Eastern India,<br>1st June to 30th November 2020	Prospective study,<br>ND	m/w,<br>59.4 years,<br>T2D	196 participants,<br><br>26 severe COVID cases	Severe COVID (DKA)	Medical records	BMI, HbA_{1c}, IL-6	Medical records	Age, BMI, diabetes duration, COVID severity, CRP, IL-6, D-dimer

Mondal, 2022 (b) [2557]	India<br>Hospitals-based<br>Tertiary care hospital in West Bengal,<br>January to December 2021	Prospective study,<br>ND	m/w,<br>59.9 years,<br>T2D	216 participants,<br><br>104 deaths,<br>140 severe COVID cases	Death, severe COVID (ICU, MV, sepsis or multiple organ dys-function syndrome)	Medical records	Vaccination status, blood glucose	Medical records	Age, SpO_2, COVID-19 severity, HbA_{1c}, vaccination status, Charlson comorbidity index, blood glucose, stress hyperglycaemia ratio
Morse, 2021 [2558]	USA<br>Healthcare data<br>Facilities affiliated with a large healthcare system,<br>4th December 2019 to 7th December 2020	Retrospective study,<br>ND	m/w,<br>62.9 years,<br>ND	32,544 participants,<br><br>4,185 deaths	Death	Electronic medical records	Blood glucose	Electronic medical records	Age, BMI, COVID-19 severity, SpO_2 on admission
Myers, 2021 [2559]	USA<br>Hospitals-based<br>Northwell Health System hospital in the New York area,<br>1st January to 31st May 2020	Retrospective study,<br>ND	m/w,<br>68 years,<br>T2D	3,846 participants,<br><br>953 deaths	Death	Medical records	Age, sex, COPD, heart failure, CAD, CKD, hypertension, MI, HbA_{1c}, asthma, cancer, stroke, hyperlipidaemia	Medical records	Analyses for age, sex, COPD, MI adjusted for: Age, sex, COPD, MI, ventilation<br><br>All other analyses: unadjusted
Nikniaz, 2021 [2560]	Iran<br>Hospitals-based<br>AzarCoRe (East Azar Covid-19 Registry) study, East Azerbaijan,<br>ND	Prospective study,<br>ND	m/w,<br>65.1 years,<br>ND	317 participants,<br><br>67 deaths,<br>71 severe COVID cases	Death, severe COVID (MV)	Electronic medical records	BMI	Self-reported	Age, sex, smoking status, CVD, respiratory diseases, hypertension, kidney diseases, carcinoma, liver diseases, and autoimmune diseases, glucose concentration, hs-CRP
Numa-guchi, 2022	Japan<br>Hospitals-based	Retrospective study,<br>ND	m/w,<br>78 years,<br>ND	138 participants,<br>ND	Severe COVID	Electronic medical records	HbA_{1c}	Electronic medical records	Age, sex, BMI, smoking, eGFR, CRP, days from disease onset to hospital

[2561]	Osaka City Juso Hospital, November 2020 to May 2021								admission, COVID-19 severity
Nyland, 2021 [2562]	USA TriNetX COVID-19 Research Network of 50 large health care organizations, 1st January to 1st July 2020	Retrospective study, 28 days	m/w, 62.2 years, T2D	12,954 participants, 1,067 deaths, 3,403 severe COVID cases	Death, severe COVID (Respiratory complications)	TriNetX COVID-19 Research Network	Thiazolidinedione, GLP-1 receptor agonists, DPP-4 inhibitors	TriNetX COVID-19 Research Network	Age, sex, race, ethnicity, BMI, hypertension, ischemic heart disease, cerebrovascular disease, heart failure, CKD, overweight and obesity
Oh, 2020 [2563]	South Korea Health insurance data South Korean population, up to 26th June 2020	Retrospective study, ND	m/w, ND, T2D	2,047 participants, 174 deaths	Death	NHIS-COVID-19 cohort database	Age, sex, metformin, Charlson Index, peripheral vascular disease, CKD, dementia, liver disease, chronic pulmonary disease, cerebrovascular disease, heart failure, MI, cancer, DPP-4 inhibitors, thiazolidinedione, sulfonylurea, insulin	NHIS-COVID-19 cohort database	Age, sex, metformin, income, residence, underlying disability, Charlson Index, peripheral vascular disease, CKD, dementia, peptic ulcer disease, hemiplegia or paraplegia, liver disease, chronic pulmonary disease, cerebrovascular disease, heart failure, MI, malignancy, metastatic solid tumour, DPP-4 inhibitors, thiazolidinedione, sulfonylurea, insulin
Ojeda-Fernandez, 2022 [2564]	Italy Registry data DB-Covid-19 Database, Lombardy region, 15th February 2020 to 15th March 2021	Retrospective study, ND	m/w, 74.1 years, ND	13,112 participants, 2,229 deaths, 378 severe COVID cases	Death, severe COVID (ICU)	Electronic medical records	Metformin	Electronic medical records	Age, sex, duration of diabetes, drug-derived complexity index, comorbidities of interest
O'Malley, 2020	USA Hospitals-based	Retrospective study,	m/w, 39.9 years,	113 participants,	Severe COVID	Electronic medical records	Age, sex, HbA_{1c}, CVD, CKD	Electronic medical records	Age, sex, race, obesity, HbA_{1c}

[2565]	52 sites across 22 US states, 1st March to 22nd August 2020	ND	T1D	58 severe COVID cases	(Hospitalisation)				
Orioli, 2020 [2566]	Belgium<br>Hospitals-based<br>Cliniques Universitaires Saint-Luc, Brussels,<br>1st March to 6th May 2020	Retrospective study, ND	m/w, 67 years, T1D & T2D	64 participants,<br>10 deaths	Death	Electronic medical records	Age, sex, BMI, CVD, hypertension, OSA, CKD, cognitive impairment, ACEI/ARBS, diuretics, β-blockers, statins, antiplatelet drugs	Electronic medical records	unadjusted
Ortega, 2021 [2567]	Spain<br>Hospitals-based<br>6 general hospitals from the HM Hospitales group,<br>27th January to 24th April 2020	Retrospective study, ND	m/w, 71.7 years, ND	448 participants,<br>118 deaths, 139 severe COVID cases	Death, severe COVID (IMV or death)	Electronic medical records	Age, sex, obesity, hypertension, dyslipidaemia, CVD, heart failure, CKD COPD	Electronic medical records	Age, sex, obesity, hypertension, dyslipidaemia, CVD, heart failure, CKD COPD
Ouchi, 2022 [2568]	Spain,<br>Healthcare records data<br>Primary Health Care records of the Information System for Research in Primary Care (SIDIAP), Catalonia,	Retrospective study, ND	m/e, 71.5 years, T2D	31,006 participants,<br>4,678 deaths, 8,035 severe COVID cases	Death, severe COVID	Electronic medical records (SIDIAP)	Metformin	Electronic medical records (SIDIAP)	Age, sex, obesity, smoking, HbA_{1c}, comorbidities, concomitant drugs

	March to 30th June 2020								
Palazzuoli, 2020 [2569]	Italy<br>Hospitals-based<br>Coracle multi-center registry, regions of Piedmont, Lombardy, Tuscany and Lazio,<br>22nd February to 1st April 2020	Retrospective study, ND	m/w, 69 years, ND	143 participants, ND	Death	Electronic medical records	RAS inhibitors	Electronic medical records	Unadjusted
Patel, 2021 [2570]	USA<br>Hospitals-based<br>3rd March to 5th May 2020	Retrospective study, ND	m/w, 66 years, T2D	506 participants,<br>125 deaths, 124 severe COVID cases	Death, severe COVID (MV)	Electronic medical records	HbA_{1c}	Electronic medical records	Unadjusted
Pazoki, 2021 (a) [2571]	Iran<br>Hospitals-based<br>Sina Hospital, Tehran, 20th February to 14th May 2020	Retrospective study, ND	m/w, 65 years, ND	176 participants,<br>54 deaths	Death	Electronic medical records	Age, sex, urea, cerebrovascular disease, neutrophils-to-lymphocytes ratio, CRP	Electronic medical records	Analyses for age and urea adjusted for: Age, sex, cerebrovascular disease, temperature, oxygen saturation, neutrophils-to-lymphocytes ratio, urea, CRP<br>All other analyses: unadjusted

Pazoki, 2021 (b) [2572]	Iran<br>Hospitals-based<br><br>Sina hospital, Tehran,<br>20th February to 29th October 2020	Retrospective study,<br>228 days	m/w,<br>65.4 years,<br>ND	393 participants,<br><br>310 severe COVID cases	Severe COVID (acute respiratory distress syndrome, acute cardiac injury, acute kidney injury or acute liver injury)	Electronic medical records	Age, sex, BMI, blood glucose, hypertension, cardiac disease, cerebrovascular disease, CKD, chronic lung disease, cancer, insulin, metformin, DPP-4 inhibitors, sulfonylureas, statins, RAS inhibitors, beta-blockers, antiplatelet, calcium channel blockers, CRP, ALT, AST, urea, creatinine, white blood cells, neutrophils, lymphocytes, neutrophils-lymphocyte ratio, platelets, haemoglobin, erythrocyte sedimentation rate, sodium	Electronic medical records	Analyses for metformin, sulfonylureas, DPP-4 inhibitors, insulin, RAS inhibitors, beta-blockers, calcium channel blockers, statins, antiplatelet are adjusted for age, sex, hypertension, cardiac disease, cerebrovascular disease, chronic lung disease, cancer, white blood cells, CRP, urea and AST<br>All other phenotypes are age-adjusted
Perez-Belmonte 2020 [2573]	Spain<br>Hospitals-based<br><br>Spanish Society of Internal Medicine's registry of COVID-19 patients (SEMI-COVID-19 Registry),<br>1st March to 19th July 2020	Retrospective study,<br>ND	m/w,<br>74.9 years,<br>T2D	Metformin:<br>498 participants,<br>158 deaths,<br>179 severe COVID cases<br><br>DPP4i:<br>210 participants,<br>85 deaths,<br>87 severe COVID cases<br><br>Insulin:<br>258 participants,<br>97 deaths,<br>111 severe COVID cases	Death, Severe COVID (ICU, MV or death)	SEMI-COVID-19 Registry	Metformin, DPP-4 inhibitors, insulin,	SEMI-COVID-19 Registry	Age, sex, smoking, hypertension, dyslipidaemia, CKD, cerebrovascular disease, COPD, atrial fibrillation, CAD, heart failure, obesity, dementia, Barthel index, Charlson Comorbidity Index, ACEI, ARB, anticoagulant, statin, admission blood glucose, serum creatinine, transaminase levels

Petrakis, 2022 [2574]	Greece<br>Hospitals-based<br><br>University Hospital of Alexandroupolis	Retrospective study, ND	m/w, 69.2 years, T2D	133 participants,<br><br>24 deaths, 23 severe COVID cases	Death, severe COVID (Intubation)	Electronic medical records	Blood glucose, metformin	Electronic medical records	Age, sex
Pettrone, 2021 [2575]	USA<br>Hospitals-based<br><br>6 acute hospitals and outpatient clinics, Atlanta, Georgia,<br>1st March to 7th April 2020	Retrospective study, ND	m/w, ND, ND	79 participants,<br><br>55 severe COVID cases	Severe COVID (Hospitalis ation)	Electronic medical records	HbA_{1c}	Electronic medical records	Age, sex, race, healthcare personnel status, hypertension
Phan, 2021 [2576]	France<br>Hospitals-based<br><br>Tertiary care academic center, Paris,<br>1st March to 30th April 2020	Prospective study, 21 days	m/w, 66 years, T2D	81 participants,<br><br>20 deaths, 40 severe COVID cases	Death, Severe COVID (ICU or death)	Electronic medical records	IL-6	Electronic medical records	Age, sex, BMI
Pulido-Perez, 2021 [2577]	Mexico<br>Hospitals-based<br><br>University Hospital of Puebla,<br>24th March to 3rd November 2020	Retrospective study, ND	m/w, 64.2 years, T2D	61 participants,<br><br>26 deaths	Death	Electronic medical records	Creatinine	Electronic medical records	Age, sex, BMI
Ramesh, 2021 [2578]	India<br>Hospitals-based	Retrospective study, ND	m/w, 58.4 years, ND	102 participants,<br><br>46 deaths	Death	Electronic medical records	eGFR, d-dimer	Electronic medical records	Unadjusted

	King George Hospital, Visakhapatnam, Andhra Pradesh,<br>October and November 2020								
Ramos Rincon, 2021 [2579]	Spain<br>Hospitals-based Spanish Society of Internal Medicine's SEMI-COVID-19 Registry (160 Hospitals in Spain),<br>1st March to 29th May	Retrospective study, ND	m/w, 85.9 years, T2D	790 participants,<br><br>385 deaths	Death	SEMI-COVID-19 Registry	SGLT 2 inhibitor, GLP 1 receptor antagonist, acetylsalicylic acid, ACEI/ARB, statins	SEMI-COVID-19 Registry	Age, sex, acquisition, BMI, comorbidities (e.g. hypertension, heart failure), symptoms (e.g. dyspnoea), physical examination (e.g. oxygen saturation, temperature), COVID-19 disease severity, laboratory findings (e.g. neutrophils, lymphocytes), medication (e.g. metformin, insulin)
Rastad, 2020 (a) [2580]	Iran<br>Hospitals-based<br><br>Hospitalized inpatients in Alborz province,<br>20th February to 25th March 2020	Retrospective study, ND	m/w, 54.8 years (for the entire population, ND for participants with diabetes), ND	267 participants,<br><br>ND	Death	Electronic medical records	Age, white blood cells, lymphocytes, neutrophils, AST, ALT, albumin, creatinine, LDH, CRP, erythrocyte sedimentation rate, haemoglobin	Electronic medical records	Age, sex, laboratory tests
Rastad, 2020 (b) [2581]	Iran<br>Hospitals-based<br><br>Hospitalized inpatients in Alborz province,<br>20th February to 27th April 2020	Retrospective study, ND	m/w, 63.8 years, ND	455 participants,<br><br>79 deaths,<br>65 severe COVID cases	Death, severe COVID (Ventilation)	Electronic medical records	Age, sex, hypertension, CVD, CKD, comorbidities, number of comorbidities, ACEI/ARBS, statins, blood glucose	Electronic medical records	Analyses for death and age, CKD and comorbidities adjusted for:<br>Age, CKD, comorbidities, neutrophils, haemoglobin, creatinine and LDH<br><br>Analyses for ventilation and age, CKD adjusted for:<br>Age, CKD, neutrophils, AST, creatinine and LDH are mutually adjusted

Rezaei, 2021 [2582]	Iran<br>Hospitals-based<br><br>National registry of hospitalized patients with COVID,<br>18th February to 22nd December 2020	Retrospective study,<br>ND	m/w,<br>63.6 years,<br>ND	37,338 participants,<br><br>6,920 deaths	Death	Electronic medical records	Age, sex, CVD, COPD, cancer, CKD, liver disease, immune deficiency disorder	Electronic medical records	Analyses for age and sex are adjusted for ICU admission, ventilation and comorbidities<br><br>All other analyses are adjusted for age, sex, ICU admission and ventilation
Rhee, 2021 [2583]	South Korea<br>Health insurance data<br><br>South Korean population,<br>up to 17th May 2020	Retrospective study,<br>ND	m/w,<br>61.8 years,<br>ND	832 participants,<br><br>34 severe COVID cases	Severe COVID (Intensive care or death)	HIRA database	RAS inhibitors	HIRA database	Age, sex, hypertension, dyslipidaemia, CVD, cerebrovascular disease, CKD, asthma, COPD, cancer, metformin, sulfonylurea, meglitinide, TZD, DPP-4 inhibitor, SGLT2 inhibitor, AGI, insulin, ACEi, ARB, beta blocker, diuretics, CCB, statin, fibrate
Riahi, 2020 [2584]	USA<br>Hospitals-based<br><br>Einstein Medical Center, Philadelphia,<br>1st March to 24th April 2020	Retrospective study,<br>ND	m/w,<br>66.4 years,<br>T1D+T2D	166 participants,<br><br>45 deaths	Death	Hospital medical records	HbA_{1c}	Hospital medical records	Age, sex, HbA_{1c}, race, HbA1c, steroid dose
Roussell, 2021 [2585]	France<br>Hospitals-based<br><br>68 hospitals, CORONADO study,<br>10th March to 10th April 2020	Prospective study,<br>28 days	m/w,<br>70.9 years,<br>T2D	2,449 participants,<br><br>512 deaths,<br>857 severe COVID cases	Death, severe COVID (MV and/or death)	Medical files	DPP-4 inhibitors	Medical files, if needed, general or specialist practitioner, regular pharmacist or biomedical laboratory	Age, sex, BMI, eGFR, HbA_{1c}, hypertension, ischemic heart disease, heart failure, cancer, treated OSA, metformin, sulphonylurea, GLP-1, insulin, corticosteroids, RAS blockers, statins, thiazide diuretics, antiplatelet therapy

Ruan, 2021 [2586]	UK<br>Hospitals-based<br><br>Association of British Clinical Diabetologists (ABCD) COVID-19 diabetes national audit,<br>March to October 2020	Retrospective study,<br>ND	m/w,<br>62 years,<br>T1D	196 participants,<br><br>53 deaths<br>68 severe COVID cases	Death, severe COVID (Death/ ICU)	Electronic medical records	Sex, BMI, HbA_{1c}, blood glucose, hypertension, microvascular disease, dementia, asthma, COPD, cancer, metformin, RAS inhibitors, creatinine	Electronic medical records	Age
Sadidi, 2022 [2587]	Iran<br>Hospitals-based<br><br>Military medical centers at AJA University of Medical Sciences, Tehran,<br>February to November 2020	Retrospective study,<br>ND	m/w,<br>66.1 years,<br>T2D	220 participants,<br><br>46 deaths	Death	Electronic medical records	Age, BMI, hypertension, DPP-4 inhibitors, metformin	Electronic medical records	Age, BMI, past medical history, hypertension, DPP-4 inhibitors, metformin
Sari-gumba, 2021 [2588]	Philippines<br>Hospitals-based<br><br>Makati Medical Center,<br>1st March to 31st August 2020	Retrospective study,<br>ND	m/w,<br>59.8 years,<br>T2D	156 participants,<br><br>25 deaths	Death	Electronic medical records	Diabetes duration, HbA1c, blood glucose, comorbidities	Electronic medical records	Age, sex, comorbidities
Satman, 2021 [2589]	Turkey<br>National register data<br><br>National COVID-19 registry of the	Retrospective study,<br>30 days	m/w,<br>53 years,<br>T2D	18,658 participants,<br><br>1,162 deaths<br>8,172 severe COVID cases	Death, severe COVID (Hospitalis ation)	National COVID-19 registry of the Turkish Ministry of Health	Age, sex, smoking, BMI, HbA_{1c}, hypertension, dyslipidaemia, obesity, chronic pulmonary disease, heart failure, CVD, microvascular complications, cancer,	National COVID-19 registry of the Turkish Ministry of Health	Age, sex<br><br>Analyses for smoking, education, BMI, D-dimer, CRP, statins and aspirin are unadjusted

	Turkish Ministry of Health, 11th March to 30th May 2020						RAS blocker, insulin, statins, aspirin		
Savarese, 2020 [2590]	Sweden<br>National register data<br><br>Swedish National Patient Registry (NPR), 2nd February to 31st May 2020	Retrospective study, 30 days	m/w, 72 years (for the entire population, ND for participants with diabetes), T1D & T2D	2,692 participants,<br><br>846 deaths	Death	Cause of Death Registry	RAS inhibitors	Swedish National Patient Registry	Age, sex, living alone, education, number of children, income, residence, country of birth, medication (e.g. insulin, RAS inhibitors), defibrillator, comorbidities (e.g. hypertension, heart failure, COPD, MI, stroke, cancer, dementia)
Saygili, 2021 [2591]	Turkey<br>Hospitals-based<br><br>Canakkale Onsekiz Mart University Medical Faculty Hospital,<br>12th March to 22nd December 2020	Retrospective study, 134 days	m/w, 66 years, ND	240 participants,<br><br>86 deaths	Death	Electronic medical records	Age, metformin, ALT, eGFR	Electronic medical records	Age, sex, blood glucose, ALT, eGFR, number of hospitalisation days, diabetes duration, chronic pulmonary disease, coronary artery disease, cerebrovascular disease, hypertension, hyperlipidaemia
Seiglie, 2020 [2592]	USA<br>Hospitals-based<br><br>Massachusetts General Hospital,<br>11th March to 30th April 2020	Prospective study, 14 days	m/w, 66.7 years, ND	168 participants,<br><br>28 deaths, 66 severe COVID cases	Death, severe COVID (MV)	Manual chart review	Age, sex, HbA1c, BMI, race/ethnicity	Manual chart review and Enterprise Data Warehouse (EDW)	Age, sex, ethnicity, coronary artery disease or myocardial infarction, chronic heart failure, hypertension, COPD/asthma, cancer, liver disease, renal disease
Shah, 2020 [2593]	USA<br>Hospitals-based<br><br>Three Phoebe Putney hospitals,	Retrospective study, ND	m/w, 60.1 years (for the entire population, ND for participants	228 participants,<br><br>ND	Death, severe COVID (Death, new dialysis requireme	Electronic medical records	ACE-inhibitor/AT1 blocker	Electronic medical records	Age, BMI, sex, hypertension, coronary artery disease, congestive heart failure, COPD, asthma, CKD, cancer, immunosuppression, chronic liver disease, drug abuse, alcohol abuse,

	2nd March to 22nd May 2020		with diabetes), ND		nt, MV or ICU care)				smoking, ESRD on dialysis and presentation severity
Shang, 2020 (a) [2594]	China<br>Hospitals-based<br>Wuhan No.7 Hospital,<br>25th December 2019 to 20th March 2020	Retrospective study, ND	m/w, 59.0 years (for the entire population, ND for participants with diabetes), ND	84 participants,<br>17 deaths	Death	Electronic medical records	Insulin	Electronic medical records	Unadjusted
Shang, 2021 (b) [2595]	China<br>Hospitals-based<br>Huoshenshan Hospital, Wuhan,<br>February to April 2020	Retrospective study, ND	m/w, 65.2 years, ND	80 participants,<br>19 deaths	Death	Electronic medical records	LDH	Electronic medical records	CRP, LDH
Shauly-Aharonov, 2021 [2596]	Israel<br>Healthcare data<br>Meuhedet healh maintencance organistion (HMO),<br>1st March to 31st October	Retrospective study, ND	m/w, ND, ND	1,718 participants,<br>258 severe COVID cases	Severe COVID	Electronic medical records	Age, sex, BMI, blood glucose, ischemic heart disease, hypertension	Electronic medical records	Age, sex, BMI, ischemic heart disease, hypertension, sector
Shesta-kova, 2022 [2597]	Russia<br>National register data<br>National Diabetes Register (NDR),	Retrospective study, ND	m/w, 64.9 years, T1D & T2D	235,248 participants<br>35,088 deaths	Death	Electronic medical records	Vaccination status, age, sex, obesity, diabetes duration, HbA_{1c}, insulin, metformin, DPP-4 inhibitors, GLP-1 receptor agonists,	Electronic medical records	Vaccination status, age, sex, obesity, diabetes duration, HbA_{1c}, insulin, metformin, DPP-4 inhibitors, GLP-1 receptor agonists, sulfonylureas, SGLT-2 inhibitors

	20th March 2020 to 25th November 2021						sulfonylureas, SGLT-2 inhibitors		
Shi, 2020 [2598]	China<br>Hospitals-based<br><br>Renmin Hospital of Wuhan University and Zhongnan Hospital of Wuhan University,<br>1st January to 8th March 2020	Retrospective study, ND	m/w, 64.0 years, ND	153 participants,<br><br>31 deaths	Death	Electronic medical records	Age, hypertension, COPD, blood glucose, white blood cells, neutrophils, lymphocytes, platelets, CRP, prothrombin time, creatinine, eGFR, total cholesterol, triglycerides, procalcitonin,	Electronic medical records	Mutual adjustment for covariates in the analyses for age, sex, hypertension, CVD and COPD<br><br>Other analyses unadjusted
Shukla, 2021 [2599]	USA<br>Hospitals-based<br><br>NewYork-Presbyterian (NYP) Weill Cornell Medical Center, NYP Queens Hospital and NYP Lower Manhattan Hospital,<br>1st March to 13th May 2020	Retrospective study, ND	m/w, 69 years, T1D & T2D	1,134 participants,<br><br>476 severe COVID cases	Severe COVID (ICU, MV or death)	Electronic medical records	Age, sex, obesity, ethnicity, coronary artery disease, heart failure, cerebrovascular disease, hypertension, pulmonary disease, CKD, smoking, metformin, GLP-1 receptor agonists, DPP-4 inhibitors, sulfonylureas, insulin, SGLT-2 inhibitors	Electronic medical records	Age, sex, ethnicity, BMI, coronary artery disease, heart failure, cerebrovascular disease, hypertension, pulmonary disease, CKD, smoking, medication
Silverii, 2020 [2600]	Italy<br>Hospitals-based<br><br>Sicilian population,<br>up to 14th May 2020	Retrospective study, ND	m/w, 73.3 years, T2D	159 participants,<br><br>59 deaths	Death	Electronic medical records	Metformin, thiazolidinedione, insulin, sulfonylureas, DPP-4 inhibitor, SGTL-2 inhibitors, GLP-1 receptor agonists	Electronic medical records	Unadjusted
Smati, 2020 [2601]	France<br>Hospitals-based	Prospective study, 7 days	m/w, 70.1 years, T2D	1,965 participants,	Death, severe COVID (IMV or death)	Medical files	BMI	Medical files, if needed, general or specialist practitioner, regular pharmacist or biomedical laboratory	Age, sex, smoking, microvascular complications, macrovascular complications, hypertension, non-alcoholic fatty liver

	French hospitals, CORONADO,<br>10th March to 10th April 2020			190 deaths, 546 severe COVID cases					disease, COPD, treated obstructive sleep apnoea, insulin, GLP1-RA
Solerte, 2020 [2602]	Italy<br>Hospitals-based<br><br>Seven academic medical centers in Northern Italy,<br>1st March to 30th April 2020	Retrospective case-control study, 30 days	m/w, 69.0 years, T2D	338 participants<br><br>94 deaths, 23 severe COVID cases	Death, severe COVID (MV)	Electronic medical records	Age, sex, cancer, CVD, CKD, antiviral DPP-4 inhibitors	Electronic medical records	Age, sex, cancer, CVD, CKD, sitagliptin, hydroxychloroquine, antiviral agents
Soliman, 2021 [2603]	Egypt<br>Hospitals-based<br><br>Kasr Alainy Hospital, Cairo,<br>1st March to 31st May 2020	Prospective RCT, 42 days	m/w, 71 years, T2D	56 participants,<br><br>10 deaths	Death	Electronic medical records	Age, sex, COPD, hypertension	Electronic medical records	Unadjusted
Sonmez, 2021 [2604]	Turkey<br>National register data<br><br>Turkish Ministry of Health database (TurCoviDia),<br>11th March to 30th May 2020	Retrospective study, 30 days	m/w, 61 years, T2D	9,213 participants,<br><br>2,065 severe COVID cases	Severe COVID (ICU admission)	National COVID-19 registry of the Turkish Ministry of Health	Age	National COVID-19 registry of the Turkish Ministry of Health	Age, sex, HbA_{1c}, pulmonary CT findings, hypertension, dyslipidaemia, obesity, asthma/COPD, heart failure, CVD, CKD, cancer, lymphopenia, insulin, RAS blockers, statins, acetylsalicylic acid
Stevens, 2021 [2605]	USA<br>Hospitals-based<br><br>Columbia University Irving Medical Center, New York,<br>1st March to 31st May 2020	Retrospective study, 30 days	m/w, 68.4 years, T1D & T2D	955 participants,<br><br>157 severe COVID cases	Severe COVID (diabetic ketoacido sis)	Electronic medical records	HbA1c	Electronic medical records	Age, sex, BMI, ethnicity, hypertension, CKD, pulmonary disease, liver disease, SGLT-2 inhibitors, HbA_{1c}, steroid use, pressor or inotrope requirement, renal replacement therapy, acute kidney injury, ventilation, lactic acid

Strollo, 2021 [2606]	Italy<br>Hospitals-based<br><br>Osservatorio per la Salute, Autonomous Province of Bolzano,<br>up to 27th April 2020	Retrospective study,<br>ND	m/w,<br>76.7 years,<br>T2D	193 participants,<br><br>44 deaths	Death	Electronic medical records	DPP-4 inhibitors	Electronic medical records	Unadjusted
Tallon, 2022 [2607]	USA<br>Healthcare data<br><br>Cerner Real-World DataTM (87 US health system facilities),<br>December 2019 to mid-September 2020	Retrospective study,<br>ND	m/w,<br>62.2 years,<br>T1D & T2D	23,372 participants,<br><br>16,031 severe COVID cases	Severe COVID (Hospitalisation)	Electronic medical records	Age, sex, obesity, ethnicity, diabetes type, HbA_{1c}	Electronic medical records	Age, sex, diabetes type, ethnicity, payer, BMI, region, hyperglycaemia and acidosis, HbA_{1c}
Tamura, 2021 [2608]	Brazil<br>Hospitals-based<br><br>Santa Caterina Hospital, São Paulo,<br>10th March to 13th November 2020	Retrospective study,<br>ND	m/w,<br>64.6 years,<br>T1D & T2D	188 participants,<br><br>19 deaths,<br>42 severe COVID cases	Death, severe COVID (MV)	Electronic medical records	Age, sex, BMI, obesity, smoking, cardiopathy, hypertension, COPD, neurological disease, CKD, dyslipidaemia, immunosuppression, asthma, metformin	Electronic medical records	Analyses for age and cardiopathy with death as well as analyses for age and CKD with severity are adjusted for age, sex and comorbidities<br><br>All other phenotypes are unadjusted
Tian, 2021 [2609]	UK<br><br>Salford area,<br>March to October 2020	Retrospective study,<br>ND	m/w,<br>ND,<br>T2D	308 participants,<br><br>56 deaths	Death	Health records	Age	Health records	Age, sex, HbA_{1c}, diastolic blood pressure, BMI, creatinine, smoking, urine/creatinine ratio
Tramunt, 2021 [2610]	France<br>Hospitals-based	Prospective study,<br>28 days	m/w,<br>70 years,<br>T1D & T2D	2,380 participants,	Severe COVID (MV or death)	Medical files	Sex	Medical files, if needed, general or specialist practitioner,	Age, BMI, smoking, microvascular complications, hypertension, macrovascular

	French hospitals, CORONADO, 10th March to 10th April 2020			844 severe COVID cases				regular pharmacist or biomedical laboratory	complications, COPD, treated OSA
Tuan, 2022 [2611]	USA TriNetX COVID-research network datsbase of 55 health care organisations, 1st January 2020 to 30th June 2021	Retrospective study, ND	m/w, ND, T1D & T2D	16,511 participants, 1,809 deaths, 1510 severe COVID cases	Death, severe COVID (MV)	Electronic medical records (TriNetX research network database)	Neuropathy	Electronic medical records (TriNetX research network database)	PSM including age, sex, ethnicity, HbA_{1c}, obesity, hypertension, respiratory diseases, ischemic heart disease, heart failure, cerebrovascular disease, nicotine dependence, alcohol related disorders, socioeconomic status
Valle, 2022 [2612]	Spain Hospitals-based University Hospital, Santiago de Compostela, March 2020 to January 2021	Retrospective study, ND	m/w, 74.5 years, ND	159 participants, 36 deaths	Death	Electronic medical records	Age, sex, hypertension, dyslipidaemia, coronary artery disease, smoking, cancer, obesity, CKD, blood glucose, HbA_{1c}	Electronic medical records	Age, sex, hypertension, dyslipidaemia, coronary artery disease, smoking, cancer, obesity, CKD, blood glucose, HbA_{1c}
Vargas Vazquez, 2021 [2613]	Mexico Hospitals-based Instituto Nacional de Ciencias Médicas y Nutrición Salvador Zubirán, Mexico City, 16th March to 1st July 2020	Prospective study, ND	w/m, 57 years, T2D	109 participants, 34 deaths 52 severe COVID cases	Death, severe COVID	Electronic medical records	Blood glucose	Electronic medical records	Age, sex, BMI, Charlson index

Vasbinder, 2022 [2614]	International<br>Hospitals-based<br><br>International Study of Inflammation in COVID-19 (ISIC) in five ISIC sites (USA, Denmark, Greece and Germany),<br>1st February 2020 to 1st June 2021	Retrospective multicentre study,<br>ND	m/w,<br>64 years,<br>T1D & T2D	686 participants,<br><br>116 deaths,<br>259 severe COVID cases	Death,<br>Severe COVID (MV, renal replaceme nt therapy or death)	Electronic medical records	Age, sex, ethnicity, BMI, smoking, blood glucose hypertension, coronary artery disease, heart failure, CKD, diabetes type, insulin, metformin, SGLT-2 inhibitor, GLP-1 receptor agonists, sulfonylureas, DPP-4 inhibitors, thiazolidinediones	Electronic medical records	Age, sex, ethnicity, BMI, smoking, hypertension, coronary artery disease, heart failure, CKD, eGFR, blood glucose
Wander, 2021 [2615]	USA<br>Health care data<br><br>Corporate Data Warehouse (CDW) from the Veterans Health Administration (VHA),<br>1st March 2020 to 10th March 2021	Retrospective study,<br>30 days	m/w,<br>67.7 years,<br>ND	64,892 participants,<br><br>4,943 deaths<br>4,265 severe COVID cases	Death,<br>severe COVID (ICU)	Electronic medical records (Corporate Data Warehouse)	Age, sex, obesity, ethnicity, residence, smoking, HbA_{1c}, hypertension, CVD, heart failure, insulin, metformin, DPP-4 inhibitors, GLP-1 receptor agonists, sulfonylureas, SGLT-2 inhibitors, statins, thiazolidinedione, RAS inhibitors, antiplatelet	Electronic medical records (Corporate Data Warehouse)	Age, sex, ethnicity, BMI, smoking, RAS inhibitors, statins, platelet inhibitors, hypertension, CVD, heart failure, HbA_{1c}, glucose-lowering medication
Wang, 2020 (a) [2616]	China<br>Hospitals-based<br><br>Renmin Hospital of Wuhan University,<br>11th January to 6th February 2020	Retrospective study,<br>ND	m/w,<br>66 years,<br>T2D	67 participants,<br><br>51 severe COVID cases	Severe COVID (Poor therapeu-tic effect)	Electronic medical records	Sex, chronic pulmonary disease, CVD	Electronic medical records	unadjusted
Wang, 2021 (b) [2617]	USA<br>Health care data	Retrospective study,<br>ND	m/w,<br>67.6 years,<br>T2D	16,504 participants,	Severe COVID	Electronic medical records	Age, sex, obesity, HbA1c, hypertension, coronary artery	Electronic medical records	adjusted, longitudinal HbA1c, Hypertension, Sex, Nephropathy, Obesity, ARB,

	OptumLabs Data Warehouse (OLDW) with data across the USA,<br>20th January to 6th November 2020			2,952 severe COVID cases		(OptumLabs Data Warehouse)	disease, stroke, CKD, retinopathy, insulin, neuropathy, metformin, RAS inhibitors	(OptumLabs Data Warehouse)	Neuropathy, ACEi, Stroke, Age, Insulin, Retinopathy, Coronary Artery Disease, Metformin, Metformin + Insulin, Corticosteroid
Wang, 2021 (c) [2618]	China<br>Hospitals-based<br><br>Five hospitals in Wuhan,<br>1st January to 17th March 2020	Retrospective study,<br>ND	m/w,<br>60 years,<br>ND	150 participants,<br><br>42 severe COVID cases	Severe COVID (ICU, MV or death)	Electronic medical records	HDL cholesterol	Electronic medical records	Age, sex, hospital
Wargny, 2021 [2619]	France<br>Hospitals-based<br><br>68 French hospitals, CORONADO study,<br>10th March to 10th April 2020	Prospective study,<br>28 days	m/w,<br>69.7 years,<br>T1D & T2D	2,796 participants,<br><br>577 deaths, 800 severe COVID cases	Death, Severe COVID	Medical files	Age, sex, ethnicity, BMI, diabetes duration, HbA_{1c}, hypertension, dyslipidaemia, smoking, microvascular complications, macrovascular complications, heart failure, NAFLD or liver cirrhosis, cancer, COPD, treated OSA, metformin, GLP-1 receptor agonists, sulfonylureas/glinides, insulin, β-blockers, diuretics, calcium channel blockers, ARBs/ACEi/MRA, statins, Anti-platelet and anticoagulant therapy, admission plasma glucose, eGFR, AST, white cell count, platelet count, CRP	Medical files, if needed, general or specialist practitioner, regular pharmacist or biomedical laboratory	Analyses for death and age, sex, BMI, hypertension, micro- and macrovascular complications, COPD, treated OSA, metformin, insulin, ARBs/ACEi/MRA, statins, anticoagulants, eGFR, white cell count, platelets and CRP adjusted for:<br>Age, sex, BMI, hypertension, micro- and macrovascular complications, COPD, treated OSA, metformin, insulin, ARBs/ACEi/MRA, statins, anticoagulation therapy, time between symptom onset and hospital admission,<br>dyspnoea, eGFR, AST, white cell count, platelets, CRP<br><br>All other analyses for death and all analyses for severe COVID adjusted for age

Wong, 2021 (a) [2620]	Hong Kong<br>Hospitals-based<br><br>Hong Kong Hospital Authority (HA),<br>21st January 2020 and 31st January 2021	Retrospective study,<br>ND	m/w,<br>65.2 years,<br>T2D	1,214 participants,<br><br>115 deaths,<br>239 severe COVID cases	Death severe COVID (IMV or death)	Electronic medical records	DPP-4 inhibitors	Electronic medical records	PSM-weighting including age, sex, comorbidities (e.g. hypertension, CKD, obesity), long-term medications (e.g. RAS inhibitors, insulin, metformin), drug use during hospital and laboratory parameters (e.g. white blood cells, CRP, eGFR)
Wong, 2022 (b) [2621]	USA<br>National registry data<br><br>National COVID Cohort Collaborative (N3C) Database,<br>up to 22nd July 2021	Retrospective study,<br>30 days	m/w,<br>62.1 years,<br>T2D	39,616 participants,<br><br>2,242 deaths,<br>2,779 severe COVID cases	Death, severe COVID (MV or ECMO)	Electronic medical records	Age, sex, obesity, ethnicity, HbA_{1c}, MI, heart failure, peripheral vascular disease, cerebrovascular disease, stroke, CKD, liver disease, chronic pulmonary disease, cancer, dementia, insulin, metformin, DPP-4 inhibitors, sulfonylureas, GLP-1 receptor agonists, SGLT-2 inhibitors, thiazolidinedione	Electronic medical records	Age, sex, ethnicity, BMI, MI, heart failure, peripheral vascular disease, stroke, dementia, pulmonary disease, liver disease, CKD, cancer, HIV, metformin, GLP-1 receptor agonists, DPP-4 inhibitors, SGLT-2 inhibitors, sulfonylureas, thiazolidinedione, insulin
Wong, 2022 (c) [2622]	Hong Kong<br>Hospitals-based<br><br>Hong Kong Hospital Authority (HA),<br>21st January 2020 and 31st January 2021	Retrospective study,<br>16 days	m/w,<br>65.2 years,<br>T2D	1,214 participants,<br><br>115 deaths	Death	Electronic medical records	Metformin	Electronic medical records	PSM-weighting including age, sex, comorbidities (e.g. hypertension, CKD, obesity), long-term medications (e.g. RAS inhibitors, insulin, metformin), drug use during hospital and laboratory parameters (e.g. white blood cells, CRP, eGFR)
Wu, 2021 [2623]	China<br>Hospitals-based<br><br>21 hospitals in Hubei province,<br>31st December 2019 to 21st April 2020	Retrospective study,<br>28 days	m/w,<br>59 years,<br>T2D	946 participants,<br><br>106 deaths	Death	Electronic medical records	Hyperlipidaemia	Electronic medical records	Age, sex, blood glucose level, CRP, ALT, hospital site

Xiao, 2021 [2624]	China Hospitals-based<br><br>HuoShenShan Hospital, Jinyintan Hospital and Taikang Tongji Hospital, Wuhan,<br>4th February to 31st March 2020	Retrospective study, ND	m/w, 66 years, ND	325 participants,<br><br>17 deaths, 115 severe COVID cases	Death, severe COVID	Electronic medical records	CVD, lung disease, CRP, neutrophils, platelets	Electronic medical records	CVD, lung disease, CRP, neutrophils, platelets, dyspnoea, cough, creatinine kinase-MB
Xu, 2020 [2625]	China<br>Hospitals-based<br><br>Renmin Hospital of Wuhan University,<br>30th January to April 26th 2020	Case series study, ND	m/w, 66.0 years, T2D	114 participants,<br><br>27 deaths	Death	Electronic medical records	Blood glucose	Electronic medical records	Age, sex, cerebral diseases cardiovascular diseases, chronic renal diseases, digestive diseases pulmonary diseases, NEWS2
Yan, 2020 [2626]	China<br>Hospitals-based<br><br>14 hospitals in Zhejiang Province,<br>10th January to 28th February 2020	Retrospective study, ND	m/w, ND T2D	58 participants,<br><br>21 severe COVID cases	Severe COVID	Electronic medical records	Metformin, insulin, α-glycosidase inhibitors, sulfonylureas, thiazolidinedione, DPP-4 inhibitor	Electronic medical records	Age, sex, BMI
Yeh, 2022 [2627]	USA<br>Hospitals-based<br><br>PaTH Toward a Learning Health System (PaTH) clinical data research network including five academic health	Retrospective study, 30 days	m/w, 62.3 years, T2D	4,944 participants,<br><br>586 severe COVID cases	Severe COVID (ICU, intubation or death)	Electronic medical records	Age, sex, BMI, ethnicity, Charlson index, insulin, metformin, DPP-4 inhibitors, GLP-1 receptor agonists, SGLT-2 inhibitors,	Electronic medical records	Age, sex, ethnicity, smoking, CCI score, time of COVID-19 diagnosis, BMI, systolic and diastolic blood pressure, HDL, LDL, HbA_{1c}, insulin metformin DPP-4 inhibitors, GLP-1 receptor agonists, SGLT2 inhibitors

	systems in Pennsylvania,<br>March 2020 to February 2021								
Yoo, 2022 [2628]	South Korea<br>Hospitals-based<br>Seobuk Hospital, Seoul,<br>1st June to 30th November 2020	Retrospective study, ND	m/w, 63 years, T2D	129 participants,<br><br>59 severe COVID cases	Severe COVID	Electronic medical records	Vaccination status, age, sex, BMI, obesity, HbA_{1c}, blood glucose, albumin, AST	Electronic medical records	Analyses for HbA_{1c} and blood glucose are adjusted for age, sex, BMI, time to admission, systolic blood pressure, vaccination status<br>All other phenotypes are unadjusted
You, 2020 [2629]	South Korea<br><br>Health insurance data<br><br>South Korean population, up to 31st March 2020	Retrospective study, ND	m/w, ND, T2D	495 participants,<br><br>9 ventilations, 68 oxygen therapies, 33 ICU admissions	Severe COVID (Ventilation, oxygen therapy, ICU admission)	HIRA database	SGLT2 inhibitors, sulfonylureas, thiazolidinedione	HIRA database	Age, sex, insurance type, hypertension, MI, heart failure, peripheral artery diseases, cerebrovascular diseases, dementia, COPD, asthma, connective tissue diseases, pulmonary diseases, liver diseases, hemiplegia, renal diseases, cancer
Zeltyn-Abramov, 2021 [2630]	Russia<br>Hospitals-based<br><br>City hospital No. 52,<br>15th April to 30th July 2020	Retrospective study, ND	m/w, 68 years, T2D	53 participants,<br><br>24 deaths	Death	Electronic medical records	Sex, BMI, blood glucose, CVD, MI, insulin	Electronic medical records	Unadjusted
Zhan, 2022 [2631]	China<br>Hospitals-based<br><br>HuoShenShan Hospital and Taikang-Tongji Hospital,<br>12th February to 10th April 2020	Prospective study, ND	m/w, 65 years, T2D	574 participants,<br><br>24 deaths, 51 severe COVID cases	Death, Severe COVID (ICU, MV or death)	Electronic medical records	Age, sex, glycaemic control, hypertension, CVD, coronary heart disease, cerebrovascular disease, CKD, liver disease, cancer	Electronic medical records	Analysis for glycaemic control are adjusted for age, sex and disease severity<br><br>Analysis for CVD are adjusted for glycaemic control, dyspnoea and disease severity<br><br>All other phenotypes are unadjusted

Zhang, 2020 (a) [2632]	China<br>Hospitals-based<br><br>Zhongnan Hospital of Wuhan University,<br>3rd January to 14th April 2020	Retrospective study, ND	m/w, 62 years, T2D	74 participants,<br><br>27 severe COVID cases	Severe COVID	Electronic medical records	Serum amyloid A	Electronic medical records	Unadjusted
Zhang, 2020 (b) [2633]	China<br>Hospitals-based<br><br>Central hospital of Wuhan,<br>25th January to 14th February 2020	Retrospective study, ND	m/w, 65.5 years, T2D	52 participants,<br><br>21 severe COVID cases	Severe COVID	Electronic medical records	Age, sex, diabetes duration, HbA_{1c}, BMI, total cholesterol, HDL, LDL, triglycerides, IL-6, thiazolidinedione, CKD, diabetic foot, erythrocyte sedimentation rate	Electronic medical records	Analyses for age, sex and troponin adjusted for: Age, sex, fasting plasma glucose, troponin, α-glucosidase inhibitor<br><br>All other analyses: unadjusted
Zhu, 2020 [2634]	China<br>Hospitals-based<br><br>19 hospitals in Hubei Province,<br>30th December 2019 to 20th March 2020	Retrospective study, 28 days	m/w, 62.7 years, T2D	810 participants,<br><br>61 deaths, 133 severe COVID cases	Death, severe COVID (ARDS)	Electronic medical records	Blood glucose control	Electronic medical records	Age, sex, hospital sites, indicators of the severity of COVID-19, hypertension, CHD, cerebrovascular diseases, chronic liver diseases, and chronic renal diseases

ND: no data

ABCD: Association of British Clinical Diabetologists, ACE: angiotensin converting enzyme, ALT: alanine aminotransferase, ARB: angiotensin II receptor blocker, AST: aspartate aminotransferase, AT1: angiotensin II receptor type 1, BMI: body mass index, CCB: calcium channel blocker, CHD: coronary heart disease, CKD: chronic kidney disease, COPD: chronic obstructive pulmonary disease, CORONADO: Coronavirus SARS-CoV-2 and Diabetes Outcomes, CPK: creatine phosphokinase, CRP: C-reactive protein, CT: computed tomography, CVD: cardiovascular disease, DBP: diastolic blood pressure, DPP-4: dipeptidyl peptidase 4, eGFR: estimated glomerular filtration rate, ESDR: end-stage renal disease GGT: γ-glutamyl transferase, GLP-1 RA: glucagon-like peptide 1-receptor agonist, HIRA: Health Insurance Review & Assessment Service ICU: intensive care unit, LDH: lactate dehydrogenase, LHS: Leumit Health Services, MRA: mineralocorticoid-receptor antagonist, MV: mechanical ventilation, NAFLD: non-alcoholic fatty liver disease, NEWS2: National Early Warning Score 2, OSA: obstructive sleep apnoea, PCR: polymerase chain reaction, RAAS: renin-angiotensin-aldosterone system, RAS: renin-angiotensin system, SBP: systolic blood pressure, SES: socioeconomic status, SGLT-2: sodium-glucose co-transporter-2, T1D: type 1 diabetes, T2D: type 2 diabetes, TZD: thiazolidinedione.

ESM Table 6: Certainty of evidence for associations between phenotypes of patients with diabetes and SARS-CoV-2 regarding COVID-19 death using the GRADE tool

Certainty assessment							Relative risk (95% CI)	Certainty
№ of studies	Study design	Risk of bias	Inconsistency	Indirectness	Imprecision	Other considerations		
**Male sex**								
39	observational studies	not serious	not serious	not serious	not serious	none	**RR 1.40** (1.31 to 1.50)	⊕⊕⊕⊕ HIGH
**Age: ≥65**								
20	observational studies	not serious [a]	not serious	not serious	not serious	strong association	**RR 3.45** (2.44 to 4.87)	⊕⊕⊕⊕ HIGH
**Age: per 5 years**								
30	observational studies	not serious [a]	not serious	not serious	not serious	dose response gradient	**RR 1.28** (1.21 to 1.36)	⊕⊕⊕⊕ HIGH
**BMI: per 5 kg/m²**								
11	observational studies	very serious [b]	not serious	not serious	not serious	none	**RR 1.04** (0.98 to 1.10)	⊕⊕◯◯ LOW
**Overweight vs normal weight**								
10	observational studies	not serious	not serious	not serious	not serious	none	**RR 0.96** (0.88 to 1.04)	⊕⊕⊕⊕ HIGH
**Obesity vs normal weight**								
21	observational studies	not serious	not serious	not serious	not serious	potential publication bias	**RR 1.18** (1.04 to 1.34)	⊕⊕⊕◯ MODERATE
**Smoking: smoker vs non-smoker**								
11	observational studies	serious [c]	not serious	not serious	serious [d]	none	**RR 1.11** (0.93 to 1.34)	⊕⊕◯◯ LOW
**Area of residence: rural vs urban**								
3	observational studies	not serious	not serious	not serious	very serious [e]	none	**RR 0.92** (0.69 to 1.24)	⊕⊕◯◯ LOW
**Ethnicity: Black vs Non-Hispanic white**								
8	observational studies	not serious	not serious	serious [f]	not serious	none	**RR 0.88** (0.80 to 0.96)	⊕⊕⊕◯ MODERATE
**Ethnicity: Hispanic vs Non-Hispanic white**								

ESM Table 6: Certainty of evidence for associations between phenotypes of patients with diabetes and SARS-CoV-2 regarding COVID-19 death using the GRADE tool

Certainty assessment							Relative risk (95% CI)	Certainty
№ of studies	Study design	Risk of bias	Inconsistency	Indirectness	Imprecision	Other considerations		
4	observational studies	not serious	not serious	serious [f]	not serious	none	**RR 1.25** (1.08 to 1.45)	⊕⊕⊕◯ MODERATE
**Ethnicity: Asian vs Non-Hispanic white**								
2	observational studies	not serious	not serious	serious [f]	very serious [e]	none	**RR 0.97** (0.55 to 1.68)	⊕◯◯◯ VERY LOW
**Ethnicity: White vs Non-white**								
2	observational studies	very serious [b]	very serious [g]	not serious	serious [d]	none	**RR 0.93** (0.73 to 1.18)	⊕◯◯◯ VERY LOW
**Type 2 vs type 1 diabetes**								
6	observational studies	very serious [b]	serious [h]	not serious	very serious [e]	none	**RR 1.09** (0.60 to 1.97)	⊕◯◯◯ VERY LOW
**Diabetes duration: per 5 years**								
6	observational studies	serious [c]	not serious	not serious	serious [d]	none	**RR 1.13** (0.92 to 1.38)	⊕⊕◯◯ LOW
**Diabetes duration: ≥10 years**								
2	observational studies	not serious	serious [h]	not serious	very serious [e]	none	**RR 1.16** (0.28 to 4.81)	⊕◯◯◯ VERY LOW
**HbA_{1c}: 53-75 vs <53 mmol/mol (7-9 vs <7%)**								
8	observational studies	not serious	not serious	not serious	not serious	none	**RR 1.18** (1.06 to 1.32)	⊕⊕⊕⊕ HIGH
**HbA_{1c}: ≥75 vs <53 mmol/mol (≥9 vs <7%)**								
9	observational studies	serious [c]	serious [h]	not serious	serious [d]	none	**RR 1.11** (0.93 to 1.32)	⊕◯◯◯ VERY LOW
**HbA_{1c}: per 20 mmol/mol (per 4%)**								
10	observational studies	very serious [b]	not serious	not serious	serious [d]	none	**RR 0.99** (0.81 to 1.21)	⊕◯◯◯ VERY LOW
**Blood glucose at admission: >6 - <11.1 mmol/l**								
7	observational studies	very serious [b]	not serious	not serious	serious [d]	none	**RR 1.46** (0.99 to 2.14)	⊕◯◯◯ VERY LOW

ESM Table 6: Certainty of evidence for associations between phenotypes of patients with diabetes and SARS-CoV-2 regarding COVID-19 death using the GRADE tool

Certainty assessment							Relative risk (95% CI)	Certainty
№ of studies	Study design	Risk of bias	Inconsistency	Indirectness	Imprecision	Other considerations		
**Blood glucose at admission: >10 mmol/l**								
19	observational studies	not serious [a]	not serious	not serious	not serious	none	**RR 2.01** (1.54 to 2.63)	⊕⊕⊕⊕ HIGH
**Blood glucose at admission: per 5 mmol/l**								
11	observational studies	serious [c]	not serious	not serious	not serious	dose response gradient, potential publication bias	**RR 1.38** (1.15 to 1.65)	⊕⊕⊕◯ MODERATE
**Poorly vs well-controlled**								
3	observational studies	serious [c]	serious [h]	not serious	serious [d]	strong association	**RR 2.94** (0.82 to 10.48)	⊕⊕◯◯ LOW
**Use of insulin vs non-use**								
26	observational studies	not serious	not serious	not serious	not serious	none	**RR 1.33** (1.18 to 1.49)	⊕⊕⊕⊕ HIGH
**Use of metformin vs non-use**								
23	observational studies	not serious	not serious	not serious	not serious	none	**RR 0.69** (0.60 to 0.79)	⊕⊕⊕⊕ HIGH
**Use of DPP-4 inhibitors vs non-use**								
22	observational studies	not serious	not serious	not serious	not serious	none	**RR 0.91** (0.80 to 1.03)	⊕⊕⊕⊕ HIGH
**Use of sulfonylurea/glinides/secretagogues vs non-use**								
11	observational studies	not serious	serious [h]	not serious	serious [d]	none	**RR 1.03** (0.87 to 1.22)	⊕⊕◯◯ LOW
**Use of GLP1-RA vs non-use**								
9	observational studies	not serious	not serious	not serious	not serious	none	**RR 0.83** (0.71 to 0.97)	⊕⊕⊕⊕ HIGH
**Use of SGLT-2i vs non-use**								
9	observational studies	not serious	serious [h]	not serious	serious [d]	none	**RR 0.88** (0.73 to 1.04)	⊕⊕⊕◯ MODERATE
**Use of thiazolidinedione vs non-use**								

ESM Table 6: Certainty of evidence for associations between phenotypes of patients with diabetes and SARS-CoV-2 regarding COVID-19 death using the GRADE tool

Certainty assessment							Relative risk (95% CI)	Certainty
№ of studies	Study design	Risk of bias	Inconsistency	Indirectness	Imprecision	Other considerations		
6	observational studies	not serious	serious [h]	not serious	very serious [e]	none	**RR 0.83** (0.55 to 1.25)	⊕◯◯◯ VERY LOW
**Hypertension**								
34	observational studies	serious [c]	not serious	not serious	serious [d]	none	**RR 1.10** (0.99 to 1.23)	⊕⊕◯◯ LOW
**Dyslipidaemia**								
9	observational studies	very serious [b]	serious [h]	not serious	not serious	none	**RR 0.96** (0.80 to 1.14)	⊕◯◯◯ VERY LOW
**Total cardiovascular disease**								
23	observational studies	not serious	not serious	not serious	not serious	none [i]	**RR 1.35** (1.23 to 1.50)	⊕⊕⊕⊕ HIGH
**Coronary artery disease**								
14	observational studies	serious [c]	not serious	not serious	not serious	none	**RR 1.30** (1.11 to 1.53)	⊕⊕⊕◯ MODERATE
**Myocardial infarction**								
5	observational studies	serious [c]	not serious	not serious	serious [d]	none	**RR 1.20** (1.00 to 1.43)	⊕⊕◯◯ LOW
**Heart failure**								
14	observational studies	not serious	not serious	not serious	not serious	none	**RR 1.33** (1.21 to 1.47)	⊕⊕⊕⊕ HIGH
**Atrial fibrillation**								
2	observational studies	not serious	not serious	not serious	serious [d]	none	**RR 0.84** (0.61 to 1.15)	⊕⊕⊕◯ MODERATE
**Peripheral vascular disease**								
5	observational studies	not serious	serious [h]	not serious	serious [d]	none	**RR 1.03** (0.87 to 1.22)	⊕⊕◯◯ LOW
**Cerebrovascular disease**								
15	observational studies	serious [c]	serious [h]	not serious	not serious	none	**RR 1.22** (1.01 to 1.48)	⊕⊕◯◯ LOW

ESM Table 6: Certainty of evidence for associations between phenotypes of patients with diabetes and SARS-CoV-2 regarding COVID-19 death using the GRADE tool

Certainty assessment							Relative risk (95% CI)	Certainty
№ of studies	Study design	Risk of bias	Inconsistency	Indirectness	Imprecision	Other considerations		
**Stroke**								
5	observational studies	serious [c]	serious [h]	not serious	serious [d]	none	**RR 1.23** (0.95 to 1.59)	⊕◯◯◯ VERY LOW
**Microvascular complications**								
6	observational studies	serious [c]	not serious	serious [j]	not serious	none	**RR 1.26** (1.08 to 1.46)	⊕⊕◯◯ LOW
**Chronic kidney disease**								
28	observational studies	not serious	not serious	not serious	not serious	none	**RR 1.54** (1.39 to 1.70)	⊕⊕⊕⊕ HIGH
**Retinopathy**								
3	observational studies	very serious [b]	not serious	not serious	very serious [e]	none	**RR 1.08** (0.77 to 1.52)	⊕◯◯◯ VERY LOW
**Neuropathy**								
3	observational studies	not serious	serious [h]	not serious	very serious [e]	none	**RR 0.99** (0.73 to 1.36)	⊕◯◯◯ VERY LOW
**Diabetic foot**								
4	observational studies	very serious [b]	serious [h]	not serious	very serious [e]	none	**RR 1.10** (0.62 to 1.97)	⊕◯◯◯ VERY LOW
**Liver disease**								
6	observational studies	not serious	not serious	not serious	not serious	none	**RR 1.40** (1.17 to 1.67)	⊕⊕⊕⊕ HIGH
**Chronic pulmonary diseases, not specified**								
9	observational studies	not serious	not serious	serious [i]	not serious	potential publication bias	**RR 1.37** (1.04 to 1.81)	⊕⊕◯◯ LOW
**Chronic obstructive pulmonary disease**								
19	observational studies	not serious [a]	not serious	not serious	not serious	none	**RR 1.38** (1.24 to 1.54)	⊕⊕⊕⊕ HIGH
**Asthma**								

ESM Table 6: Certainty of evidence for associations between phenotypes of patients with diabetes and SARS-CoV-2 regarding COVID-19 death using the GRADE tool

Certainty assessment							Relative risk (95% CI)	Certainty
№ of studies	Study design	Risk of bias	Inconsistency	Indirectness	Imprecision	Other considerations		
6	observational studies	serious [c]	not serious	not serious	serious [d]	none	**RR 0.86** (0.65 to 1.13)	⊕⊕◯◯ LOW
**Obstructive sleep apnoea**								
2	observational studies	not serious	not serious	not serious	very serious [e]	none	**RR 0.92** (0.56 to 1.49)	⊕⊕◯◯ LOW
**Cancer**								
19	observational studies	not serious	serious [h]	not serious	serious [d]	none	**RR 1.25** (0.99 to 1.57)	⊕⊕◯◯ LOW
**Dementia/cognitive impairment**								
9	observational studies	serious	serious [h]	serious [j]	not serious	none	**RR 1.54** (1.15 to 2.08)	⊕⊕◯◯ LOW
**Neurodegenerative diseases, not specified**								
3	observational studies	very serious [b]	not serious	serious [j]	very serious [d,k]	none	**RR 4.29** (0.94 to 19.44)	⊕◯◯◯ VERY LOW
**Immunodeficiency diseases, not specified**								
4	observational studies	not serious	serious [h]	serious [j]	serious [d]	none	**RR 2.21** (0.93 to 5.24)	⊕◯◯◯ VERY LOW
**Any comorbidity**								
3	observational studies	very serious [b]	serious [h]	not serious	very serious [e]	none	**RR 1.41** (0.61 to 3.24)	⊕◯◯◯ VERY LOW
**≥3 comorbidities**								
2	observational studies	very serious [b]	very serious [l]	not serious	extremely serious [e,k]	strong association	**RR 10.36** (0.64 to 168.30)	⊕◯◯◯ VERY LOW
**Charlson index: per 1 unit**								
2	observational studies	serious [c]	not serious	not serious	not serious	none	**RR 1.33** (1.13 to 1.57)	⊕⊕⊕◯ MODERATE
**Use of statins vs non-use**								

ESM Table 6: Certainty of evidence for associations between phenotypes of patients with diabetes and SARS-CoV-2 regarding COVID-19 death using the GRADE tool

Certainty assessment							Relative risk (95% CI)	Certainty
№ of studies	Study design	Risk of bias	Inconsistency	Indirectness	Imprecision	Other considerations		
13	observational studies	not serious	serious [h]	not serious	very serious [e]	none	**RR 0.99** (0.73 to 1.34)	⊕⊕◯◯ LOW
**Use of renin inhibitor vs non-use**								
19	observational studies	not serious	serious [h]	not serious	not serious	none	**RR 0.91** (0.79 to 1.04)	⊕⊕⊕◯ MODERATE
**Use of beta-blockers vs non-use**								
5	observational studies	serious [c]	serious [h]	not serious	very serious [e]	none	**RR 0.94** (0.65 to 1.37)	⊕◯◯◯ VERY LOW
**Use of calcium channel blocker vs non-use**								
3	observational studies	serious [c]	not serious	not serious	very serious [e]	none	**RR 0.99** (0.77 to 1.27)	⊕◯◯◯ VERY LOW
**Use of diuretics vs non-use**								
3	observational studies	very serious [b]	not serious	not serious	serious [d]	none	**RR 1.29** (0.83 to 2.02)	⊕◯◯◯ VERY LOW
**Use of acetylsalicylic acid vs non-use**								
5	observational studies	not serious	serious [h]	not serious	very serious [e]	none	**RR 1.44** (0.72 to 2.87)	⊕◯◯◯ VERY LOW
**Use of use of antithrombotic drugs (antiplatelet/anticoagulant) vs non-use**								
6	observational studies	serious [c]	not serious	not serious	not serious	none	**RR 1.14** (1.02 to 1.27)	⊕⊕⊕◯ MODERATE
**CRP: per 5 mg/l**								
10	observational studies	serious [c]	not serious	not serious	not serious	dose response gradient, [i]	**RR 1.07** (1.02 to 1.12)	⊕⊕⊕⊕ HIGH
**IL-6: per 5 pg/ml**								
2	observational studies	not serious	not serious	not serious	very serious [e,m]	none	**RR 2.37** (0.64 to 8.69)	⊕⊕◯◯ LOW
**Procalcitonin: per 1 ng/ml**								
2	observational studies	very serious [b]	not serious	not serious	serious [m]	dose response gradient	**RR 1.25** (1.04 to 1.51)	⊕◯◯◯ VERY LOW

ESM Table 6: Certainty of evidence for associations between phenotypes of patients with diabetes and SARS-CoV-2 regarding COVID-19 death using the GRADE tool

Certainty assessment							Relative risk (95% CI)	Certainty
№ of studies	Study design	Risk of bias	Inconsistency	Indirectness	Imprecision	Other considerations		
**Albumin: per 1 g/l**								
5	observational studies	very serious [b]	not serious	not serious	serious [d]	none	**RR 0.77** (0.59 to 1.00)	⊕◯◯◯ VERY LOW
**ALT: per 5 unit/l**								
6	observational studies	not serious	not serious	not serious	not serious	none	**RR 1.02** (0.97 to 1.07)	⊕⊕⊕⊕ HIGH
**AST: per 5 unit/l**								
5	observational studies	serious [c]	not serious	not serious	not serious	dose response gradient	**RR 1.28** (1.06 to 1.54)	⊕⊕⊕⊕ HIGH
**eGFR: per 10 ml/min per 1.73 m^2**								
6	observational studies	serious [c]	not serious	not serious	not serious	dose response gradient	**RR 0.80** (0.71 to 0.90)	⊕⊕⊕⊕ HIGH
**Urea: per 1 mmol/l**								
2	observational studies	extremely serious [n]	serious [g]	not serious	serious [m]	none	**RR 1.03** (0.96 to 1.09)	⊕◯◯◯ VERY LOW
**Creatinine: per 10 µmol/l**								
6	observational studies	serious [c]	not serious	not serious	not serious	none	**RR 1.01** (0.98 to 1.03)	⊕⊕⊕◯ MODERATE
**White blood cell count: per 1x10^9/l**								
7	observational studies	serious [c]	serious [h]	not serious	not serious	none	**RR 1.09** (0.99 to 1.19)	⊕⊕◯◯ LOW
**Neutrophils: per 1x10^9/l**								
6	observational studies	very serious [b]	serious [o]	not serious	serious [d]	none	**RR 1.15** (1.00 to 1.33)	⊕◯◯◯ VERY LOW
**Lymphocyte count: per 1x10^9/l**								
6	observational studies	serious [c]	serious [o]	not serious	not serious	dose response gradient	**RR 0.59** (0.40 to 0.86)	⊕⊕⊕◯ MODERATE
**Platelet count: per 1x10^9/l**								

ESM Table 6: Certainty of evidence for associations between phenotypes of patients with diabetes and SARS-CoV-2 regarding COVID-19 death using the GRADE tool

Certainty assessment							Relative risk (95% CI)	Certainty
№ of studies	Study design	Risk of bias	Inconsistency	Indirectness	Imprecision	Other considerations		
6	observational studies	serious [c]	not serious	not serious	not serious	none	**RR 0.99** (0.99 to 1.00)	⊕⊕⊕◯ MODERATE
**Lactate dehydrogenase: per 10 unit/l**								
7	observational studies	not serious	not serious	not serious	not serious	none	**RR 1.03** (1.01 to 1.04)	⊕⊕⊕⊕ HIGH
**D-dimer: per 1 nmol/l**								
6	observational studies	not serious	not serious	not serious	not serious	none	**RR 1.00** (0.99 to 1.00)	⊕⊕⊕⊕ HIGH
**Prothrombin time: per 1 second**								
2	observational studies	extremely serious [n]	not serious	not serious	serious [m]	dose-response gradient	**RR 1.12** (1.03 to 1.22)	⊕◯◯◯ VERY LOW
**Erythrocyte sedimentation rate: per 1 mm/h**								
2	observational studies	very serious [b]	not serious	not serious	very serious [e,m]	none	**RR 1.18** (0.77 to 1.81)	⊕◯◯◯ VERY LOW
**Haemoglobin: per 1 mmol/l**								
3	observational studies	serious [c]	very serious [g]	not serious	serious [d]	none	**RR 0.89** (0.39 to 1.98)	⊕◯◯◯ VERY LOW
**Ferritin: per 1 ug/l**								
2	observational studies	not serious	serious [l]	not serious	not serious	none	**RR 1.03** (0.95 to 1.11)	⊕⊕⊕◯ MODERATE
**Fibrinogen: per 1 g/l**								
2	observational studies	not serious	serious [l]	not serious	very serious [e]	none	**RR 1.69** (0.45 to 6.30)	⊕⊕◯◯ LOW

CI: Confidence interval; RR: Risk ratio

Explanations:

a. High proportion (>25-50%) of evidence from studies with high risk of bias; however the estimate was robust in stratified analysis in studies with low/moderate risk of bias.

b. Very high proportion (>50-90%) of evidence from studies with high risk of bias.

c. High proportion (>25-50%) of evidence from studies with high risk of bias.
d. 95% CI includes the null value and includes important benefit (RR<0.80) OR harm (RR>1.20).
e. 95% CI includes the null value and includes important benefit (RR<0.80) AND harm (RR>1.20).
f. Comparator not consistent across the studies and indirectness possible.
g. Opposite direction of effect estimates and no overlap of 95% CIs.
h. Opposite direction of effect estimates and minimal overlap of 95% CIs.
i. Eggers test indicated potential publication bias, but mainly small studies were missing and publication bias can be neglected.
j. Composite endpoint; diseases are not further specified/defined and indirectness is possible.
k. Extremely wide 95% CI.
l. No overlap of 95% CI.
m. Number of participants: n<400.
n. Extremely high proportion (>90-100%) of evidence from studies with high risk of bias.
o. Minimal overlap of 95% CIs.

ESM Table 7: Certainty of evidence for associations between phenotypes of patients with diabetes and SARS-CoV-2 regarding COVID-19 severity using the GRADE tool

Certainty assessment							Relative risk (95% CI)	Certainty
№ of studies	Study design	Risk of bias	Inconsistency	Indirectness	Imprecision	Other considerations		
**COVID-19 vaccination: yes vs no**								
3	observational studies	not serious	not serious	not serious	not serious	none	**RR 0.32** (0.26 to 0.38)	⊕⊕⊕⊕ HIGH
**COVID-19 vaccination: 2 doses vs none**								
2	observational studies	extremely serious [a]	not serious	not serious	Serious [q]	none	**RR 0.25** (0.11 to 0.53)	⊕◯◯◯ VERY LOW
**Male sex**								
62	observational studies	not serious [b]	not serious	not serious	not serious	none	**RR 1.27** (1.21 to 1.34)	⊕⊕⊕⊕ HIGH
**Age: ≥65 years**								
25	observational studies	not serious [b]	not serious	not serious	not serious	strong association	**RR 2.21** (1.65 to 2.96)	⊕⊕⊕⊕ HIGH
**Age, per 5 years**								
44	observational studies	not serious [b]	not serious	not serious	not serious	dose response gradient	**RR 1.15** (1.12 to 1.17)	⊕⊕⊕⊕ HIGH
**BMI, per 5 kg/m²**								
20	observational studies	not serious [c]	serious [d]	not serious	not serious	none	**RR 1.03** (0.95 to 1.11)	⊕⊕⊕◯ MODERATE
**Overweight**								
15	observational studies	not serious	serious [d]	not serious	serious [e]	publication bias suspected	**RR 1.05** (0.92 to 1.21)	⊕◯◯◯ VERY LOW
**Obesity**								
30	observational studies	not serious	not serious	not serious	not serious	publication bias suspected	**RR 1.28** (1.15 to 1.43)	⊕⊕⊕◯ MODERATE
**Smoking: smoker vs not smoker**								
16	observational studies	serious [f]	serious [d]	not serious	serious [e]	none	**RR 1.08** (0.90 to 1.28)	⊕◯◯◯ VERY LOW

ESM Table 7: Certainty of evidence for associations between phenotypes of patients with diabetes and SARS-CoV-2 regarding COVID-19 severity using the GRADE tool

Certainty assessment							Relative risk (95% CI)	Certainty
№ of studies	Study design	Risk of bias	Inconsistency	Indirectness	Imprecision	Other considerations		
**Area of residence: rural vs urban**								
3	observational studies	not serious	not serious	serious [g]	not serious	none	**RR 0.80** (0.74 to 0.86)	⊕⊕⊕◯ MODERATE
**Ethnicity: Black vs Non-Hispanic white**								
13	observational studies	serious [f]	serious [d]	serious [h]	serious [e]	none	**RR 1.07** (0.93 to 1.22)	⊕◯◯◯ VERY LOW
**Ethnicity: Hispanic vs Non-Hispanic white**								
6	observational studies	not serious	serious [d]	serious [h]	serious [e]	none	**RR 1.16** (0.80 to 1.68)	⊕◯◯◯ VERY LOW
**Ethnicity: Asian vs Non-Hispanic white**								
6	observational studies	very serious [i]	serious [d]	serious [g,h]	serious [e]	none	**RR 1.19** (0.81 to 1.74)	⊕◯◯◯ VERY LOW
**Ethnicity: White vs Non-white**								
2	observational studies	very serious [i]	very serious [j]	not serious	serious [e]	none	**RR 0.92** (0.71 to 1.19)	⊕◯◯◯ VERY LOW
**Type 2 vs type 1 diabetes**								
9	observational studies	serious [f]	serious [d]	not serious	very serious [k]	none	**RR 0.98** (0.67 to 1.42)	⊕◯◯◯ VERY LOW
**Diabetes duration, per 5 years**								
7	observational studies	serious [f]	serious [d]	not serious	not serious	none	**RR 1.00** (0.90 to 1.10)	⊕⊕◯◯ LOW
**Diabetes duration: ≥10**								
4	observational studies	serious [f]	serious [d]	not serious	very serious [k]	none	**RR 1.22** (0.64 to 2.31)	⊕◯◯◯ VERY LOW
**HbA_{1c}: 53-75 vs <53 mmol/mol (7-9 vs <7%)**								
16	observational studies	not serious	not serious	not serious	not serious	none	**RR 1.21** (1.09 to 1.35)	⊕⊕⊕⊕ HIGH
**HbA_{1c}: ≥75 vs <53 mmol/mol (≥9 vs <7%)**								

ESM Table 7: Certainty of evidence for associations between phenotypes of patients with diabetes and SARS-CoV-2 regarding COVID-19 severity using the GRADE tool

Certainty assessment							Relative risk (95% CI)	Certainty
№ of studies	Study design	Risk of bias	Inconsistency	Indirectness	Imprecision	Other considerations		
12	observational studies	serious [f]	serious [d]	not serious	not serious	none	**RR 1.26** (1.06 to 1.49)	⊕⊕◯◯ LOW
**HbA1c, per 20 mmol/mol (per 4%)**								
28	observational studies	not serious [b]	serious [d]	not serious	not serious	dose response gradient, publication bias can be neglected [l]	**RR 1.51** (1.25 to 1.80)	⊕⊕⊕⊕ HIGH
**Blood glucose at admission: ≥6 - <10 vs <6 mmol/L**								
9	observational studies	not serious	not serious	not serious	not serious	none	**RR 1.89** (1.21 to 2.95)	⊕⊕⊕⊕ HIGH
**Blood glucose at admission: ≥10**								
19	observational studies	not serious [b]	not serious	not serious	not serious	none	**RR 1.81** (1.42 to 2.31)	⊕⊕⊕⊕ HIGH
**Blood glucose at admission, per 5 mmol/l**								
14	observational studies	not serious [b]	not serious	not serious	not serious	dose response gradient, publication bias suspected	**RR 1.10** (1.05 to 1.18)	⊕⊕⊕⊕ HIGH
**Poorly vs well-controlled at admission**								
3	observational studies	not serious	serious [d]	serious [g]	not serious	strong association	**RR 2.32** (1.16 to 4.66)	⊕⊕⊕◯ MODERATE
**Use of insulin vs non-use**								
35	observational studies	not serious	serious [d]	not serious	not serious	none [l]	**RR 1.31** (1.17 to 1.47)	⊕⊕⊕◯ MODERATE
**Use of metformin vs non-use**								
34	observational studies	not serious	serious [d]	not serious	not serious	none	**RR 0.78** (0.71 to 0.85)	⊕⊕⊕◯ MODERATE
**Use of DPP-4 inhibitors vs non-use**								
30	observational studies	not serious	serious [d]	not serious	not serious	none	**RR 0.93** (0.86 to 1.01)	⊕⊕⊕◯ MODERATE
**Use of sulfonylurea/glinide/secretagogues vs non-use**								
18	observational studies	not serious	not serious	not serious	not serious	none	**RR 1.15** (1.02 to 1.29)	⊕⊕⊕⊕ HIGH

ESM Table 7: Certainty of evidence for associations between phenotypes of patients with diabetes and SARS-CoV-2 regarding COVID-19 severity using the GRADE tool

Certainty assessment							Relative risk (95% CI)	Certainty
№ of studies	Study design	Risk of bias	Inconsistency	Indirectness	Imprecision	Other considerations		
**Use of GLP1-RA vs non-use**								
13	observational studies	not serious	serious [d]	not serious	serious [e]	none	**RR 0.85** (0.72 to 1.01)	⊕⊕◯◯ LOW
**Use of SGLT-2i vs non-use**								
15	observational studies	not serious	serious [d]	not serious	not serious	none	**RR 0.93** (0.85 to 1.01)	⊕⊕⊕◯ MODERATE
**Use of thiazolidinedione vs non-use**								
10	observational studies	not serious	not serious	not serious	not serious	publication bias suspected	**RR 0.98** (0.81 to 1.18)	⊕⊕⊕◯ MODERATE
**Use of alpha-glucosidase inhibitors vs non-use**								
3	observational studies	very serious [i]	serious [d]	not serious	very serious [k, q]	none	**RR 0.71** (0.24 to 2.12)	⊕◯◯◯ VERY LOW
**Hypertension**								
49	observational studies	not serious [b]	serious [d]	not serious	not serious	none [l]	**RR 1.23** (1.14 to 1.33)	⊕⊕⊕◯ MODERATE
**Dyslipidaemia**								
11	observational studies	very serious [i]	not serious	not serious	not serious	none	**RR 1.04** (0.97 to 1.12)	⊕⊕◯◯ LOW
**Total Cardiovascular disease (CVD)**								
32	observational studies	serious [f]	serious [d]	not serious	not serious	none	**RR 1.36** (1.19 to 1.56)	⊕⊕◯◯ LOW
**Coronary artery disease (CAD)**								
23	observational studies	serious [f]	serious [d]	not serious	serious [e]	none	**RR 1.11** (0.98 to 1.26)	⊕◯◯◯ VERY LOW
**Myocardial infarction (MI)**								
6	observational studies	not serious	serious [d]	not serious	serious [e]	none	**RR 1.10** (0.90 to 1.35)	⊕⊕◯◯ LOW
**Heart failure (HF)**								

ESM Table 7: Certainty of evidence for associations between phenotypes of patients with diabetes and SARS-CoV-2 regarding COVID-19 severity using the GRADE tool

Certainty assessment							Relative risk (95% CI)	Certainty
№ of studies	Study design	Risk of bias	Inconsistency	Indirectness	Imprecision	Other considerations		
17	observational studies	not serious	serious [d]	not serious	not serious	none [l]	**RR 1.30** (1.17 to 1.46)	⊕⊕⊕◯ MODERATE
**Atrial fibrillation (AF)**								
2	observational studies	not serious	not serious	not serious	very serious [k]	none	**RR 0.94** (0.71 to 1.23)	⊕⊕◯◯ LOW
**Peripheral vascular disease (PVD)**								
5	observational studies	not serious	serious [d]	not serious	serious [e]	none	**RR 1.11** (0.98 to 1.25)	⊕⊕◯◯ LOW
**Cerebrovascular disease**								
22	observational studies	not serious [b]	serious [d]	not serious	serious [e]	none	**RR 1.12** (0.97 to 1.28)	⊕⊕◯◯ LOW
**Stroke**								
8	observational studies	not serious	not serious	not serious	not serious	none	**RR 1.07** (0.98 to 1.16)	⊕⊕⊕⊕ HIGH
**Microvascular diseases (MVD)**								
6	observational studies	very serious [i]	not serious	serious [m]	serious [e]	none	**RR 1.18** (1.00 to 1.39)	⊕◯◯◯ VERY LOW
**Chronic kidney disease (CKD)**								
38	observational studies	not serious [b]	not serious	not serious	not serious	publication bias suspected	**RR 1.54** (1.39 to 1.70)	⊕⊕⊕◯ MODERATE
**Retinopathy**								
6	observational studies	serious [f]	serious [d]	not serious	serious [e]	none	**RR 1.05** (0.83 to 1.33)	⊕◯◯◯ VERY LOW
**Neuropathy**								
5	observational studies	not serious	not serious	serious [g]	not serious	none	**RR 1.17** (1.07 to 1.28)	⊕⊕⊕◯ MODERATE
**Diabetic foot**								
6	observational studies	very serious [i]	serious [d]	not serious	very serious [k]	none	**RR 0.99** (0.58 to 1.71)	⊕◯◯◯ VERY LOW

ESM Table 7: Certainty of evidence for associations between phenotypes of patients with diabetes and SARS-CoV-2 regarding COVID-19 severity using the GRADE tool

Certainty assessment							Relative risk (95% CI)	Certainty
№ of studies	Study design	Risk of bias	Inconsistency	Indirectness	Imprecision	Other considerations		
**Liver disease**								
9	observational studies	not serious	not serious	not serious	not serious	none	**RR 1.21** (1.09 to 1.35)	⊕⊕⊕⊕ HIGH
**Chronic pulmonary diseases, not specified**								
14	observational studies	not serious	not serious	serious [m]	not serious	publication bias suspected	**RR 1.20** (1.06 to 1.37)	⊕⊕◯◯ LOW
**Chronic obstructive pulmonary disease (COPD)**								
23	observational studies	not serious [b]	serious [d]	not serious	not serious	none	**RR 1.37** (1.21 to 1.56)	⊕⊕⊕◯ MODERATE
**Asthma**								
8	observational studies	very serious [i]	not serious	not serious	serious [e]	none	**RR 0.96** (0.78 to 1.18)	⊕◯◯◯ VERY LOW
**Obstructive sleep apnoea**								
3	observational studies	extremely serious [a]	not serious	serious [g]	not serious	none	**RR 1.33** (1.03 to 1.73)	⊕◯◯◯ VERY LOW
**Cancer**								
24	observational studies	not serious [b]	serious [d]	not serious	not serious	none	**RR 1.37** (1.07 to 1.75)	⊕⊕⊕◯ MODERATE
**Dementia/cognitive impairment**								
10	observational studies	not serious	serious [d]	serious [m]	not serious	none	**RR 1.58** (1.16 to 2.16)	⊕⊕◯◯ LOW
**Neurodegenerative disease, not specified**								
3	observational studies	very serious [i]	not serious	serious [m]	serious [e]	none	**RR 2.33** (0.85 to 6.41)	⊕◯◯◯ VERY LOW
**Immunodeficiency disorders, not specified**								
5	observational studies	not serious	not serious	very serious [g,m]	not serious	none	**RR 1.21** (1.02 to 1.43)	⊕⊕◯◯ LOW
**Any comorbidity**								

ESM Table 7: Certainty of evidence for associations between phenotypes of patients with diabetes and SARS-CoV-2 regarding COVID-19 severity using the GRADE tool

Certainty assessment							Relative risk (95% CI)	Certainty
№ of studies	Study design	Risk of bias	Inconsistency	Indirectness	Imprecision	Other considerations		
3	observational studies	very serious [i]	not serious	serious [g]	not serious	strong association	**RR 2.05** (1.25 to 3.36)	⨁⨁◯◯ LOW
**≥3 comorbidities vs no comorbidities**								
2	observational studies	very serious [i]	serious [n]	serious [g]	extremely serious [o]	strong association	**RR 12.06** (0.99 to 146.39)	⨁◯◯◯ VERY LOW
**Charlson index, per 1 unit**								
3	observational studies	not serious	not serious	serious [g]	not serious	none	**RR 1.19** (1.06 to 1.34)	⨁⨁⨁◯ MODERATE
**Use of statins vs non-use**								
16	observational studies	not serious	serious [d]	not serious	very serious [k]	none	**RR 1.01** (0.76 to 1.35)	⨁◯◯◯ VERY LOW
**Use of renin inhibitors vs non-use**								
29	observational studies	serious [f]	serious [d]	not serious	not serious	none	**RR 1.00** (0.91 to 1.11)	⨁⨁◯◯ LOW
**Use of beta-blockers vs non-use**								
6	observational studies	very serious [i]	not serious	not serious	serious [e]	none	**RR 0.91** (0.74 to 1.13)	⨁◯◯◯ VERY LOW
**Use of calcium channel blocker (CCB) vs non-use**								
6	observational studies	serious [f]	serious [d]	not serious	serious [e]	none	**RR 1.16** (0.82 to 1.64)	⨁◯◯◯ VERY LOW
**Use of diuretics vs non-use**								
4	observational studies	extremely serious [a]	not serious	serious [g]	serious [e]	none	**RR 1.09** (0.92 to 1.29)	⨁◯◯◯ VERY LOW
**Use of acetylsalicylic acid (ASA) vs non-use**								
5	observational studies	serious [f]	serious [d]	serious [g]	not serious	none	**RR 1.45** (1.07 to 1.96)	⨁◯◯◯ VERY LOW
**Use of use of antithrombotic drugs (antiplatelet/anticoagulant) vs non-use**								
9	observational studies	not serious	serious [d]	not serious	not serious	none	**RR 1.02** (0.89 to 1.16)	⨁⨁⨁◯ MODERATE

ESM Table 7: Certainty of evidence for associations between phenotypes of patients with diabetes and SARS-CoV-2 regarding COVID-19 severity using the GRADE tool

Certainty assessment							Relative risk (95% CI)	Certainty
№ of studies	Study design	Risk of bias	Inconsistency	Indirectness	Imprecision	Other considerations		
**Triglycerides, per 1mmol/l**								
2	observational studies	extremely serious [a]	serious [d]	not serious	very serious [k, q]	none	**RR 1.29** (0.73 to 2.27)	⊕◯◯◯ VERY LOW
**Total cholesterol, per 1mmol/l**								
3	observational studies	extremely serious [a]	not serious	serious [g]	not serious	none	**RR 0.90** (0.87 to 0.93)	⊕◯◯◯ VERY LOW
**LDL, per 1mmol/l**								
2	observational studies	extremely serious [a]	not serious	serious [g]	not serious	none	**RR 0.83** (0.78 to 0.88)	⊕◯◯◯ VERY LOW
**HDL, per 1mmol/l**								
3	observational studies	very serious [i]	not serious	serious [g]	very serious [k]	none	**RR 0.64** (0.29 to 1.42)	⊕◯◯◯ VERY LOW
**CRP, per 5 mg/l**								
14	observational studies	not serious [b]	not serious [n]	not serious	not serious	dose response gradient, publication bias suspected	**RR 1.06** (1.03 to 1.10)	⊕⊕⊕⊕ HIGH
**IL-6, per 5 pg/ml**								
6	observational studies	very serious [i]	not serious [n]	not serious	not serious	dose response gradient	**RR 1.13** (1.03 to 1.25)	⊕⊕⊕◯ MODERATE
**Serum amyloid A, per 1 mg/l**								
2	observational studies	extremely serious [a]	not serious	not serious	serious [q]	none	**RR 1.00** (0.98 to 1.02)	⊕◯◯◯ VERY LOW
**Procalcitonin, per 1 ng/ml**								
3	observational studies	extremely serious [a]	not serious	not serious	not serious	dose response gradient	**RR 1.22** (1.15 to 1.30)	⊕⊕◯◯ LOW
**Albumin, per 1 g/l**								
7	observational studies	very serious [i]	serious [d]	not serious	not serious	none	**RR 0.70** (0.56 to 0.89)	⊕◯◯◯ VERY LOW
**ALT, per 5 unit/l**								
8	observational studies	serious [f]	not serious	not serious	not serious	none	**RR 1.05** (0.99 to 1.11)	⊕⊕⊕◯ MODERATE

ESM Table 7: Certainty of evidence for associations between phenotypes of patients with diabetes and SARS-CoV-2 regarding COVID-19 severity using the GRADE tool

Certainty assessment							Relative risk (95% CI)	Certainty
№ of studies	Study design	Risk of bias	Inconsistency	Indirectness	Imprecision	Other considerations		
**AST, per 5 unit/l**								
8	observational studies	not serious	not serious	not serious	not serious	dose response gradient	**RR 1.25** (1.10 to 1.41)	⊕⊕⊕⊕ HIGH
**eGFR per 10 ml/min per 1.73 m^2**								
7	observational studies	serious [f]	not serious	not serious	not serious	dose response gradient	**RR 0.83** (0.76 to 0.90)	⊕⊕⊕⊕ HIGH
**Urea, per 1 mmol/l**								
2	observational studies	extremely serious [a]	very serious [j]	serious [g]	not serious	none	**RR 0.97** (0.81 to 1.15)	⊕◯◯◯ VERY LOW
**Creatinine, per 10 µmol/l**								
9	observational studies	serious [f]	not serious	not serious	not serious	none	**RR 1.01** (0.99 to 1.02)	⊕⊕⊕◯ MODERATE
**Creatinine phosphokinase, per 1 U/l**								
2	observational studies	extremely serious [a]	not serious	not serious	Serious [q]	none	**RR 1.01** (1.01 to 1.01)	⊕◯◯◯ VERY LOW
**Serum sodium, per 10 mmol/l**								
2	observational studies	very serious [i]	very serious [j]	serious [g]	very serious [k]	none	**RR 1.20** (0.42 to 3.42)	⊕◯◯◯ VERY LOW
**Potassium, per 1 mmol/l**								
2	observational studies	serious [f]	not serious	serious [g]	serious [e]	none	**RR 1.50** (0.83 to 2.72)	⊕◯◯◯ VERY LOW
**White blood cell count, per 1x10^9/l**								
11	observational studies	serious [f]	serious [d]	not serious	not serious	dose response gradient	**RR 1.08** (1.02 to 1.16)	⊕⊕⊕◯ MODERATE
**Neutrophils, per 1x10^9/l**								
8	observational studies	very serious [i]	not serious	not serious	not serious	dose response gradient	**RR 1.18** (1.11 to 1.26)	⊕⊕⊕◯ MODERATE
**Lymphocyte count, per 1x10^9/l**								

ESM Table 7: Certainty of evidence for associations between phenotypes of patients with diabetes and SARS-CoV-2 regarding COVID-19 severity using the GRADE tool

Certainty assessment							Relative risk (95% CI)	Certainty
**№ of studies**	**Study design**	**Risk of bias**	**Inconsistency**	**Indirectness**	**Imprecision**	**Other considerations**		
11	observational studies	serious [f]	serious [d]	not serious	not serious	dose response gradient, publication bias suspected	**RR 0.62** (0.48 to 0.80)	⨁⨁◯◯ LOW
**Neutrophil-to-lymphocyte ratio**								
2	observational studies	very serious [i]	serious [p]	not serious	very serious [k]	none	**RR 1.58** (0.74 to 3.35)	⨁◯◯◯ VERY LOW
**Platelet count, per 1x10^9/l**								
9	observational studies	very serious [i]	not serious	not serious	not serious	none	**RR 0.99** (0.99 to 1.00)	⨁⨁◯◯ LOW
**Lactatdehydrogenase, per 10 unit/l**								
9	observational studies	not serious	not serious	not serious	not serious	dose response gradient	**RR 1.04** (1.01 to 1.07)	⨁⨁⨁⨁ HIGH
**D-dimer, per 1 nmol/l**								
7	observational studies	not serious	serious [d]	not serious	not serious	none	**RR 1.00** (0.99 to 1.00)	⨁⨁⨁◯ MODERATE
**Prothrombin time per 1 s**								
4	observational studies	serious [f]	not serious	not serious	not serious	dose response gradient	**RR 1.11** (1.04 to 1.17)	⨁⨁⨁◯ MODERATE
**Erythrocyte sedimentation rate, per 1 mm/h**								
4	observational studies	extremely serious [a]	not serious	not serious	not serious	none	**RR 1.02** (1.00 to 1.05)	⨁◯◯◯ VERY LOW
**Haemoglobin, per 1 mmol/l**								
7	observational studies	not serious	serious [d]	not serious	not serious	none	**RR 0.98** (0.92 to 1.03)	⨁⨁⨁◯ MODERATE
**Ferritin, per 1 ug/l**								
3	observational studies	serious [f]	not serious	not serious	not serious	none	**RR 1.00** (1.00 to 1.01)	⨁⨁⨁◯ MODERATE

CI: Confidence interval; RR: Risk ratio

Explanations:

a. Extremely high proportion (>90-100%) of evidence from studies with high risk of bias.
b. High proportion (>25-50%) of evidence from studies with high risk of bias; however the estimate was robust in stratified analysis in studies with low/moderate risk of bias.
c. Very high proportion (>50-90%) of evidence from studies with high risk of bias, however the estimate was robust in stratified analysis in studies with low/moderate risk of bias.
d. Opposite direction of effect estimates and minimal overlap of 95% CIs of some studies.
e. 95% CI includes the null value and includes important benefit OR harm.
f. High proportion (>25-50%) of evidence from studies with high risk of bias.
g. Large differences in the composite endpoint and indirectness is possible.
h. Comparator not consistent across the studies and indirectness is possible.
i. Very high proportion (>50-90%) of evidence from studies with high risk of bias.
j. Opposite direction of effect estimates and no overlap of 95% CIs of some studies
k. 95% CI includes the null value and includes important benefit AND harm.
l. Egger's test indicated potential publication bias, but mainly small studies were missing and publication bias can be neglected (not downgraded for publication bias).
m. Composite exposure; diseases are not further specified/defined and indirectness is possible.
n. No overlap of 95% CI of some estimates.
o. Extremely wide 95% CI.
p. minimal overlap of 95% CI.
q.

ESM Table 8: Meta-regression: Adjustment for risk of bias due to confounding, for associations showing differences in stratified analyses by confounding

Exposure	SRR between studies	Lower 95% CI	Higher 95% CI	P between studies
Death				
Type of diabetes	2.42	0.40	14.70	0.245
**HbA_{1c} ≥75 mmol/mol (≥9 %)**	**0.67**	**0.51**	**0.90**	**0.014**
Poorly controlled	0.12	3.84e-06	3892.65	0.236
Use of DPP-4	1.20	0.74	1.95	0.442
Use of thiazolidinedione	1.80	0.33	9.90	0.394
**Use of statins**	**2.19**	**1.51**	**3.18**	**0.001**
**Use of RAAS**	**1.62**	**1.00**	**2.64**	**0.050**
Use of antithrombotic drugs	1.15	0.88	1.49	0.216
Haemoglobin	0.45	0.003	81.93	0.304
Severity				
Type of diabetes	2.09	0.87	5.03	0.088
Diabetes duration >10 years	0.37	0.11	1.29	0.076
**HbA_{1c} ≥75 mmol/mol (≥9 %)**	**0.62**	**0.46**	**0.85**	**0.006**
Poorly controlled	0.22	0.00001	4332.39	0.299
Use of DPP-4	1.11	0.85	1.47	0.432
Use of GLP1-RA	1.46	0.90	2.38	0.111
Use of thiazolidinedione	1.91	0.92	4.00	0.077
Use of alpha glucosidase	0.26	6.95e-09	9782754.0	0.507
**Use of statins**	**2.03**	**1.47**	**2.79**	**<0.0001**
**Use of RAAS**	**1.47**	**1.08**	**2.02**	**0.018**
Use of antithrombotic drugs	0.88	0.56	1.38	0.520
Serum sodium	insufficient observations			
Serum potassium	insufficient observations			
Haemoglobin	0.66	0.39	1.12	0.101

Data in bold indicate effect modification by adjustment status.

ESM Table 9: Comparison of the 95% CIs derived by the DerSimonian-Laird method versus the Hartung-Knapp-Sidik-Jonkman method for associations between phenotypes of patients with diabetes and SARS-CoV-2 regarding COVID-19 death

	Summary RR	95% CIs from the DerSimonian-Laird method		95% CIs from the Hartung-Knapp-Sidik-Jonkman method	
**General risk factors**					
Sex (n=39)	1.40	1.31	1.50	1.29	1.52
Age ≥65y (n=20)	3.45	2.44	4.87	2.29	5.19
Age per 5 y (n=30)	1.28	1.21	1.36	1.19	1.38
BMI, per 5 kg/m² (n=11)	1.04	0.98	1.10	0.97	1.11
Overweight (n=10)	0.96	0.88	1.04	0.87	1.05
Obesity (n=21)	1.18	1.04	1.34	1.00	1.40
Smoking (n=11)	1.11	0.93	1.34	0.89	1.39
Area of residence: rural vs. urban (n=3)	0.92	0.69	1.24	0.48	1.77
Ethnicity: Black vs. Non-Hispanic white (n=8)	0.88	0.80	0.96	0.79	0.98
Ethnicity: Hispanic vs. Non-Hispanic white (n=4)	1.25	1.08	1.45	0.96	1.63
Ethnicity: Asian vs. Non-Hispanic white (n=2)	0.97	0.55	1.68	0.26	35.51
Ethnicity: White vs. Non-white (n=2)	0.93	0.73	1.18	0.20	4.31
**Diabetes-specific risk factors**					
Type 2 vs type 1 (n=5)	1.09	0.60	1.97	0.47	2.52
Diabetes duration, per 5 year (n=5)	1.13	0.92	1.38	0.81	1.57
Diabetes duration, ≥10 year (n=2)	1.16	0.28	4.81	0.0001	11702.72
HbA_{1c}, 53-75 vs <53 mmol/mol (7-9 %) (n=8)	1.18	1.06	1.32	1.03	1.35
HbA_{1c}, >75 vs <53 mmol/mol (≥9 %) (n=9)	1.11	0.93	1.32	0.91	1.36
HbA_{1c}, per 20 mmol/mol (per 4 %) (n=10)	0.99	0.81	1.21	0.79	1.25
Glucose ≥6 mmol/l (n=7)	1.46	0.99	2.14	0.90	2.25
Glucose ≥10 mmol/l (n=19)	2.01	1.54	2.63	1.42	2.87
Glucose per 5 mmol/l (n=11)	1.38	1.15	1.65	1.11	1.71
Poorly controlled (n=3)	2.94	0.82	10.48	0.13	65.59
Insulin use (n=26)	1.33	1.18	1.49	1.12	1.57
Metformin use (n=23)	0.69	0.60	0.79	0.57	0.84
DDP-4 use (n=22)	0.91	0.80	1.03	0.77	1.06
GLP1-RA (n=9)	0.83	0.71	0.97	0.69	0.99
SGLT-2i (n=9)	0.88	0.73	1.04	0.70	1.09
Sulfonylurea/glinides/secretagogues use (n=11)	1.03	0.87	1.22	0.82	1.29
Thiazolidinedione (n=6)	0.83	0.55	1.25	0.48	1.44
**Comorbidities and complications**					
Hypertension (n=34)	1.10	0.99	1.23	0.98	1.25
Dyslipidaemia (n=9)	0.96	0.80	1.14	0.76	1.21

CVD (n=23)	1.35	1.23	1.50	1.18	1.56
CAD (n=14)	1.30	1.11	1.53	1.06	1.60
Myocardial infarction (MI) (n=5)	1.20	1.00	1.43	0.91	1.58
Heart failure (HF) (n=14)	1.33	1.21	1.47	1.20	1.48
Atrial fibrillation (AF) (n=2)	0.84	0.61	1.15	0.11	6.56
Peripheral vascular disease (PVD) (n=5)	1.03	0.87	1.22	0.81	1.31
Cerebrovascular disease (n=15)	1.22	1.01	1.48	0.91	1.63
Stroke (n=5)	1.23	0.95	1.59	0.83	1.82
Microvascular complications (n=6)	1.26	1.08	1.46	0.99	1.59
CKD (n=28)	1.54	1.39	1.70	1.33	1.78
Retinopathy (n=3)	1.08	0.77	1.52	0.42	2.77
Neuropathy (n=3)	0.99	0.73	1.36	0.50	1.97
Diabetic foot (n=4)	1.10	0.62	1.97	0.27	4.49
Liver disease (n=6)	1.40	1.17	1.67	1.09	1.78
Chronic pulmonary diseases, not specified (n=9)	1.37	1.04	1.81	0.97	1.93
COPD (n=19)	1.38	1.24	1.54	1.22	1.57
Asthma (n=6)	0.86	0.65	1.13	0.59	1.25
Obstructive sleep apnoea (n=2)	0.92	0.56	1.49	0.04	21.36
Cancer (n=19)	1.25	0.99	1.57	0.93	1.68
Dementia/cognitive impairment (n=9)	1.54	1.15	2.08	1.09	2.19
Neurodegenerative diseases, not specified (n=3)	4.29	0.94	19.44	0.16	118.51
Immunodeficiency disorders, not specified (n=4)	2.21	0.93	5.24	0.37	13.18
Any comorbidities (n=3)	1.41	0.61	3.24	0.22	8.92
≥3 comorbidities (n=2)	10.36	0.64	168.30	1.47e-07	7.32e+08
Charlson index per 1 unit (n=2)	1.33	1.13	1.57	0.46	3.88
**Other medication use**					
Statins (n=13)	0.99	0.73	1.34	0.71	1.39
Acetylsalicylic acid (ASA) (n=5)	1.44	0.72	2.87	0.54	3.83
RAAS (n=19)	0.91	0.79	1.04	0.75	1.11
Beta-blockers (n=5)	0.94	0.65	1.37	0.50	1.78
Diuretics (n=3)	1.29	0.83	2.02	0.43	3.93
Antithrombotic drugs (n=6)	1.14	1.02	1.27	0.98	1.32
Calcium channel blocker (CCB) (n=3)	0.99	0.77	1.27	0.57	1.72
**Laboratory parameters on admission**					
CRP, per 5 mg/l (n=10)	1.07	1.02	1.12	0.99	1.15
IL-6, per 5 pg/ml (n=2)	2.37	0.64	8.69	0.001	10939.37
Procalcitonin, per 1 ng/ml (n=2)	1.25	1.04	1.51	0.38	4.18
Albumin, per 1 g/l (n=5)	0.77	0.59	1.00	0.40	1.47
ALT, per 5 unit/l (n=6)	1.02	0.97	1.07	0.94	1.10
AST, per 5 unit/l (n=5)	1.28	1.06	1.54	0.85	1.91

eGFR per 10 ml/min per 1.73 m^2 (=6)	0.80	0.71 0.90	0.68 0.96
Urea, per 1 mmol/l (n=2)	1.03	0.96 1.09	0.68 1.55
Creatinine, per 10 µmol/l (n=6)	1.01	0.98 1.03	0.94 1.07
White blood cell count, per $1x10^9$/ (n=7)	1.09	0.99 1.19	0.91 1.29
Lymphocyte count, per $1x10^9$/ (n=6)	0.59	0.40 0.86	0.21 1.65
Neutrophils, per $1x10^9$/ (n=6)	1.15	1.00 1.33	0.87 1.53
Platelet count, per $1x10^9$/ (n=6)	0.99	0.99 1.00	0.98 1.00
LDH, per 10 unit/l (n=7)	1.03	1.01 1.04	0.97 1.09
D-dimer, per nmol/l (n=6)	1.00	0.99 1.00	0.99 1.01
Prothrombin time, per 1 s (n=2)	1.12	1.03 1.22	0.64 1.94
Erythrocyte sedimentation rate, per 1 mm/h (n=2)	1.18	0.77 1.81	0.73 19.05
Haemoglobin, per 1 mmol/l (n=3)	0.89	0.39 1.98	0.11 7.02
Ferritin, per 1 ug/l	1.03	0.95 1.11	0.63 1.68
Fibrinogen, per 1 g/l (n=2)	1.69	0.45 6.30	0. 0003 8629.12

Grey highlighted rows present associations that exclude the null value when applying the DerSimonian-Laird method, but include the null value when calculating the 95% CI using the Hartung-Knapp-Sidik-Jonkman method.

ESM Table 10: Comparison of the 95% CIs derived by the DerSimonian-Laird method versus the Hartung-Knapp-Sidik-Jonkman method for associations between phenotypes of patients with diabetes and SARS-CoV-2 regarding COVID-19 severity

	Summary RR	95% CIs from the DerSimonian-Laird method	95% CIs from the Hartung-Knapp-Sidik-Jonkman method
**General risk factors**			
COVID-19 vaccination status, yes vs. no (n=3)	0.32	0.26 0.38	0.21 0.48
COVID-19 vaccination status, 2 doses vs. none (n=2)	0.25	0.11 0.53	0.002 35.50
Sex (n=62)	1.27	1.21 1.34	1.20 1.35
Age ≥65y (n=25)	2.21	1.65 2.96	1.53 3.18
Age per 5 y (n=44)	1.15	1.12 1.17	1.10 1.19
BMI, per 5 kg/m² (n=20)	1.03	0.95 1.11	0.92 1.15
Overweight (n=15)	1.05	0.92 1.21	0.90 1.23
Obesity (n=30)	1.28	1.15 1.43	1.13 1.47
Smoking (n=16)	1.08	0.90 1.28	0.87 1.33
Area of residence: rural vs. urban (n=3)	0.80	0.74 0.86	0.67 0.95
Ethnicity: Black vs. Non-Hispanic white (n=13)	1.07	0.93 1.22	0.91 1.24
Ethnicity: Hispanic vs. Non-Hispanic white (n=6)	1.16	0.80 1.68	0.70 1.92
Ethnicity: Asian vs. Non-Hispanic white fduration(n=6)	1.19	0.81 1.74	0.64 2.19
Ethnicity: White vs. Non-white (n=2)	0.92	0.71 1.19	0.17 4.98
**Diabetes-specific risk factors**			
Type 2 vs type 1 (n=9)	0.98	0.67 1.42	0.61 1.55
Diabetes duration per 5y (n=7)	0.995	0.90 1.10	0.86 1.16
Diabetes duration ≥10y (n=4)	1.22	0.64 2.31	0.43 3.44
HbA_{1c}, 53-75 vs <75 mmol/mol (7-9 %) (n=16)	1.21	1.09 1.35	1.06 1.38
HbA_{1c}, ≥75 vs <53 mmol/mol (≥9 %) (n=12)	1.26	1.06 1.49	0.99 1.60
HbA_{1c} per 20 mmol/mol (per 4%) (n=28)	1.51	1.25 1.80	1.12 2.02
Glucose ≥6 mmol/l (n=9)	1.89	1.21 2.95	1.12 3.19
Glucose ≥11 mmol/l (n=19)	1.81	1.42 2.31	1.30 2.52
Glucose per 5 mmol/l (n=14)	1.10	1.05 1.18	0.97 1.24
Poorly controlled (n=3)	2.32	1.16 4.66	0.35 15.30
Insulin use (n=35)	1.31	1.17 1.47	1.11 1.55
Metformin use (n=34)	0.78	0.71 0.85	0.69 0.88
DDP-4 use (n=30)	0.93	0.86 1.01	0.85 1.02
GLP1-RA (n=13)	0.85	0.72 1.01	0.71 1.03
Sulfonylurea/glinides/secretagogues use (n=18)	1.15	1.02 1.29	1.01 1.31
SGLT-2i (n=15)	0.93	0.85 1.01	0.84 1.02
Thiazolidinedione (n=10)	0.98	0.81 1.18	0.78 1.22

Alpha-glucosidase inhibitors use (n=3)	0.71	0.24	2.12	0.48	10.57
**Comorbidities and complications**					
Hypertension (n=49)	1.23	1.14	1.33	1.13	1.34
Dyslipidaemia (n=11)	1.04	0.97	1.12	0.96	1.13
CVD (n=32)	1.36	1.19	1.56	1.13	1.64
CAD (n=22)	1.11	0.98	1.26	0.95	1.29
Myocardial infarction (MI) (n=5)	1.10	0.90	1.35	0.81	1.50
Heart failure (n=17)	1.30	1.17	1.46	1.14	1.50
Atrial fibrillation (AF) (n=2)	0.94	0.71	1.23	0.16	5.45
Peripheral vascular disease (PVD) (n=5)	1.11	0.98	1.25	0.93	1.32
Cerebrovascular disease (n=22)	1.12	0.97	1.28	0.93	1.33
Stroke (n=8)	1.07	0.98	1.16	0.96	1.19
Microvascular complications (n=6)	1.18	1.00	1.39	0.93	1.49
CKD (n=38)	1.54	1.39	1.70	1.34	1.77
Retinopathy (n=6)	1.05	0.83	1.33	0.69	1.61
Neuropathy (n=5)	1.17	1.07	1.28	1.02	1.34
Diabetic foot (n=6)	0.99	0.58	1.71	0.40	2.45
Liver disease (n=9)	1.21	1.09	1.35	1.07	1.38
Chronic pulmonary diseases, not specified (n=14)	1.20	1.06	1.37	1.02	1.43
COPD (n=23)	1.37	1.21	1.56	1.15	1.63
Asthma (n=8)	0.96	0.78	1.18	0.74	1.25
Obstructive sleep apnea (n=3)	1.33	1.04	1.76	0.76	2.36
Cancer (n=24)	1.37	1.03	1.73	1.05	1.77
Dementia/cognitive impairment (n=10)	1.58	1.16	2.16	1.08	2.32
Neurodegenerative diseases, not specified (n=3)	2.33	0.85	6.41	0.24	22.84
Immunodeficiency disorders, not specified (n=5)	1.21	1.02	1.43	0.95	1.53
Any comorbidities (n=3)	2.05	1.25	3.36	0.69	6.06
≥3 comorbidities (n=2)	12.06	0.99	146.39	1.13e-06	1.29e+08
Charlson index per 1 unit (n=3)	1.19	1.06	1.34	0.88	1.62
**Other medication use**					
Statins (n=16)	1.01	0.76	1.35	0.74	1.39
Acetylsalicylic acid (ASA) (n=5)	1.45	1.07	1.96	0.92	2.26
RAAS (n=29)	1.00	0.91	1.11	0.87	1.17
Beta-blockers (n=6)	0.91	0.74	1.13	0.68	1.24
Diuretics (n=4)	1.09	0.92	1.29	0.83	1.44
Antithrombotic drugs (n=9)	1.02	0.89	1.16	0.86	1.20
Calcium channel blocker (CCB) (n=6)	1.16	0.82	1.64	0.61	2.21
**Laboratory parameters on admission**					
Triglycerides, per 1 per mmol/l (n=2)	1.29	0.73	2.27	0.03	50.81

Total cholesterol, per 1 per mmol/l (n=3)	0.90	0.87	0.93	0.84	0.97
LDL-cholesterol, per 1 per mmol/l (n=2)	0.83	0.78	0.88	0.56	1.23
HDL, per 1 per mmol/l (n=3)	0.64	0.29	1.42	0.11	3.70
CRP, per 5 mg/l (n=14)	1.06	1.03	1.10	1.01	1.12
IL-6, per 5 pg/ml (n=6)	1.13	1.03	1.25	0.83	1.54
Serum amyloid A, per 1 mg/l (n=2)	1.00	0.98	1.02	0.88	1.13
Procalcitonin, per 1 ng/ml (n=3)	1.22	1.15	1.30	1.06	1.40
Albumin, per 1 g/l (n=7)	0.70	0.56	0.89	0.39	1.27
ALT, per 5 unit/l (n=8)	1.05	0.99	1.11	0.95	1.16
AST, per 5 unit/l (n=8)	1.25	1.10	1.41	1.01	1.55
eGFR per 10 ml/min per 1.73 m^2 (n=7)	0.83	0.76	0.90	0.74	0.93
Urea, per 1 mmol/l (n=2)	0.97	0.81	1.15	0.31	3.01
Creatinine, per 10 µmol/l (n=9)	1.01	0.99	1.02	0.97	1.04
Creatine phosphokinase, per 1 U/l (n=2)	1.01	1.01	1.01	0.99	1.03
Serum sodium , per 10 mmol/l (n=2)	1.20	0.42	3.42	0.001	1074.63
Potassium, per 1 mmol/l (n=2)	1.50	0.83	2.72)	0.03	71.20
White blood cell count, per $1x10^9$/l (n=11)	1.08	1.02	1.16	0.98	1.20
Neutrophils, per $1x10^9$/l (n=8)	1.18	1.11	1.26	1.09	1.29
Lymphocyte count, per $1x10^9$/ (n=11)	0.62	0.48	0.80	0.39	0.98
Neutrophil-to-lymphocyte ratio (n=2)	1.58	0.74	3.35	0.12	207.47
Platelet count, per $1x10^9$/ (n=9)	0.99	0.99	1.00	0.99	1.00
LDH, per 10 unit/l (n=9)	1.04	1.01	1.07	0.95	1.13
D-dimer, per 1 nmol/l (n=7)	1.00	0.99	1.00	0.99	1.01
Prothrombin time, per 1 s (n=4)	1.11	1.04	1.17	1.01	1.22
Erythrocyte sedimentation rate, per 1 mm/h (n=2)	1.02	1.00	1.05	0.99	1.06
Haemoglobin, per 1 mmol/l (n=7)	0.98	0.92	1.03	0.83	1.15
Ferritin, per 1 ug/l (n=3)	1.00	1.00	1.01	1.00	1.01

Grey highlighted rows present associations that exclude the null value when applying the DerSimonian-Laird method, but include the null value when calculating the 95% CI using the Hartung-Knapp-Sidik-Jonkman method.

Risk of bias domains

	D1	D2	D3	D4	D5	D6	Overall
Abe 2020	+	+	+	+	X	X	X
Acharya 2020	+	+	+	+	X	X	X
Agarwal 2020	+	+	+	+	+	+	+
Aghaaliakbari 2020	+	+	?	+	X	?	X
Ahmed 2021	+	+	+	+	X	X	X
Alhakak 2022	+	+	-	+	-	+	-
Al Hayek 2020	+	+	+	+	X	X	X
Aon 2022	+	+	+	+	-	-	-
Alrashed 2021	+	-	+	+	+	+	-
Assad 2022	?	+	+	+	-	-	-
Barrett 2021	+	+	-	+	-	+	-
Bello-Chavolla 2020	+	+	-	?	+	+	+
Borzouei 2021	+	+	+	+	X	X	X
Boye 2021	+	+	+	+	-	-	-
Calapod 2021	-	+	-	+	X	X	X
Cao 2021	+	+	+	+	+	+	+
Cariou 2020	+	+	+	+	-	-	-
Cariou 2021	+	+	+	+	+	+	+
Chai 2022	-	+	+	-	X	X	X
Charoenngam 2021	+	+	+	+	+	+	+
Chen (a) 2020	+	+	+	+	X	X	X
Chen (b) 2020	+	+	+	+	-	-	-
Chen 2022	?	+	+	+	X	X	X
Cheng 2020	-	+	+	+	-	-	-
Choi 2020	+	+	+	+	-	+	-
Chung 2020	+	+	+	+	-	+	-
Crouse 2021	+	?	-	?	+	+	+
Corcillo 2021	?	?	+	+	-	-	-
Dalan 2020	X	?	?	?	+	+	-
de Abajo 2020	-	X	+	+	-	+	-
Demirci 2022	+	+	+	+	-	-	-
De Souza 2022	-	+	?	+	X	X	X
Djuric 2022	+	+	+	+	-	+	-
Do 2020	+	+	+	+	-	+	-
Duan 2022	+	+	+	+	-	+	-

Elibol 2021	+	+	+	+	-	X	X
Emami 2021	+	+	-	?	-	+	-
Emral 2021	-	+	+	+	-	+	-
Erol 2022	+	+	+	+	-	+	-
Fernández-Pombo 2021	+	+	+	+	+	+	+
Ferrannini 2022	+	+	+	+	+	+	+
Fox 2021	+	+	+	+	+	+	+
Fu 2022	+	+	-	+	X	X	X
Fukushima 2022	+	+	+	+	X	X	X
Ghany 2021	?	+	+	+	-	+	-
Giroda 2021	+	+	+	+	+	+	+
Gregory 2021	+	+	+	+	X	X	X
Hadjadj 2022	+	+	+	+	-	+	-
Hammad 2021	?	+	+	+	-	-	X
Harris 2022	+	+	+	+	-	+	-
Heald 2022	+	+	+	+	X	X	X
Huang 2020	+	+	+	+	X	X	X
Hui 2020	-	?	+	+	X	X	X
Ikram 2022	?	+	+	+	X	X	X
Iqbal 2021	+	+	+	+	X	X	X
Izzi-Engbeaya 2021	+	+	+	+	-	-	-
Jayaswal 2021	+	+	+	+	X	X	X
Kabootari 2022	+	+	+	+	-	X	X
Kang 2021	+	+	-	+	+	+	-
Khalili (a) 2021	?	?	+	+	X	X	X
Khalili (b) 2021	?	?	+	+	X	X	X
Khunti 2022	+	+	+	+	-	+	-
Kim 2020	+	+	+	+	-	+	-
Kristan 2021	+	+	+	+	X	X	X
Lalau 2021	+	+	+	+	+	+	+
Lampasona 2021	+	+	+	+	X	X	X
Laurenzi 2021	+	+	+	+	-	-	-
Lee 2021	+	+	+	+	-	+	-
Lei 2020	+	+	+	+	X	X	X
Leon Pedroza 2021	+	+	-	?	-	-	-
Li (a) 2020	+	+	+	+	X	X	X
Li (b) 2020	+	+	+	+	X	X	X

Liu 2020	+	?	+	+	X	X	X
Liu 2021	X	?	+	+	+	+	X
Llanera 2022	+	+	+	+	X	X	X
Llaurado 2022	+	+	+	+	+	+	+
Lombardi 2022	+	+	+	+	+	-	+
Lopez-Huamanrayme 2021	+	+	+	+	-	-	-
Longmore 2021	+	+	+	+	+	+	+
Luk 2021	+	+	+	+	-	+	-
Ma 2022	+	+	+	+	+	+	+
Madaschi, 2022	+	+	+	+	-	+	-
Mannucci 2022	-	+	+	+	-	+	-
Marimuthu 2021	+	+	+	+	X	X	X
Mehta	+	+	+	+	X	X	X
Meijer	+	+	+	+	+	+	+
Merzon 2020	+	+	+	+	+	+	+
Miguel-Yanes 2022	?	?	-	+	-	-	-
Mirani 2020	+	+	+	+	-	-	-
Mohamed 2021	+	+	+	+	-	X	X
Mondal 2021	-	+	?	+	X	X	X
Mondal 2022	-	+	+	+	X	X	X
Morse 2021	+	+	+	+	X	-	X
Myers 2021	-	+	?	+	X	X	X
Nikniaz 2021	-	+	-	+	+	-	-
Numaguchi 2022	-	+	+	+	+	+	-
Nyland 2021	?	?	?	+	+	+	?
Oh 2021	+	+	+	+	-	+	-
Ojeda-Fernandez 2022	+	+	+	+	-	+	-
O'Malley 2021	?	?	-	+	-	+	-
Orioloi 2021	+	+	+	+	X	X	X
Ortega	+	+	-	+	+	+	-
Ouchi 2022	+	+	+	+	+	-	-

Palazzuoli 2020	+	+	+	+	X	X	X
Patel 2021	+	+	+	+	X	X	X
Pazoki (a) 2021	+	+	+	+	X	X	X
Pazoki (b) 2021	+	+	+	+	X	X	X
Pérez-Belmonte 2020	+	+	+	+	+	+	+
Petrakis, 2022	-	+	+	+	-	-	-
Pettrone 2021	X	?	?	+	-	+	X
Phan	?	+	+	+	-	+	-
Pulido-Perez 2021	+	+	+	+	-	+	-
Ramesh 2021	+	+	+	+	X	X	X
Ramos Rincon 2021	-	+	+	+	+	-	-
Rastad (a) 2020	+	+	-	+	X	X	X
Rastad (b) 2020	-	+	-	+	X	X	X
Rezaei 2021	+	+	+	+	X	X	X
Rhee 2021	+	+	+	+	-	+	-
Riahi 2021	+	+	+	+	-	-	-
Roussel 2021	+	+	+	+	+	+	+
Ruan 2021	?	?	-	+	X	-	X
Sadidi, 2022	+	+	+	?	+	+	+
Sarigumba	+	+	+	+	-	+	-
Satman 2021	+	+	+	+	-	-	-
Savarese 2021	+	+	+	+	+	+	+
Saygili 2022	+	+	+	+	-	+	-
Seiglie 2021	+	?	+	+	+	+	+
Shah 2020	-	-	+	+	+	+	+
Shang (a) 2021	+	+	+	+	X	X	X
Shang (b) 2021	+	+	+	+	X	X	X
Shauly Aharonov	+	+	+	+	+	+	+
Shestakova 2022	+	+	+	+	-	+	-
Shi 2020	+	+	-	+	X	-	X
Shukla	?	+	+	+	+	+	+
Silveri 2021	?	?	?	+	X	X	X
Smati 2021	+	-	+	+	+	+	+
Solerte 2020	+	+	?	+	-	-	-
Soliman 2022	X	+	+	+	X	X	X
Sonmez 2021	X	+	+	+	X	X	X
Stevens 2021	+	+	+	+	+	+	+
Strollo, 2021	-	+	+	+	X	X	X

Tallon, 2022	-	+	+	+	-	+	-
Tamura 2021	+	+	+	+	X	X	X
Tian 2021	?	?	+	+	+	+	+
Tramunt 2021	+	+	+	+	+	+	+
Tuan 2022	+	+	+	+	+	+	+
Valle 2022	-	+	+	+	+	+	-
Vargas Vazquez 2021	+	+	+	+	+	+	+
Vasbinder 2022	+	+	+	+	-	+	-
Wander 2021	+	+	+	+	+	+	+
Wang 2020	+	+	+	+	X	X	X
Wang (a) 2021	?	+	+	+	+	+	+
Wang (b) 2021	+	+	-	+	-	+	-
Wargny 2021	+	+	+	+	-	-	-
Wong (a) 2022	+	+	+	+	+	+	+
Wong (b) 2022	-	+	+	+	+	+	-
Wong (c) 2022	+	+	+	+	+	+	+
Wu 2021	-	+	+	+	-	+	-
Xiao	+	+	+	+	X	X	X
Xu 2020	+	-	+	+	-	+	-
Yan 2020	+	+	+	+	-	+	-
Yeh 2022	?	+	+	+	-	-	-
Yoo 2022	+	+	+	+	-	X	X
You 2020	+	+	+	+	-	+	-
Zeltyn-Abramov 2021	X	+	+	+	X	X	X
Zhan 2022	+	+	+	+	X	X	X
Zhang (a) 2020	+	+	+	+	X	X	X
Zhang (b) 2020	+	+	+	+	X	X	X
Zhu 2020	+	+	+	+	-	+	-

ESM Figure 1: Risk of bias of each study for each domain and overall

Risk of bias was visualized by using the robvis visualization tool.[2635]

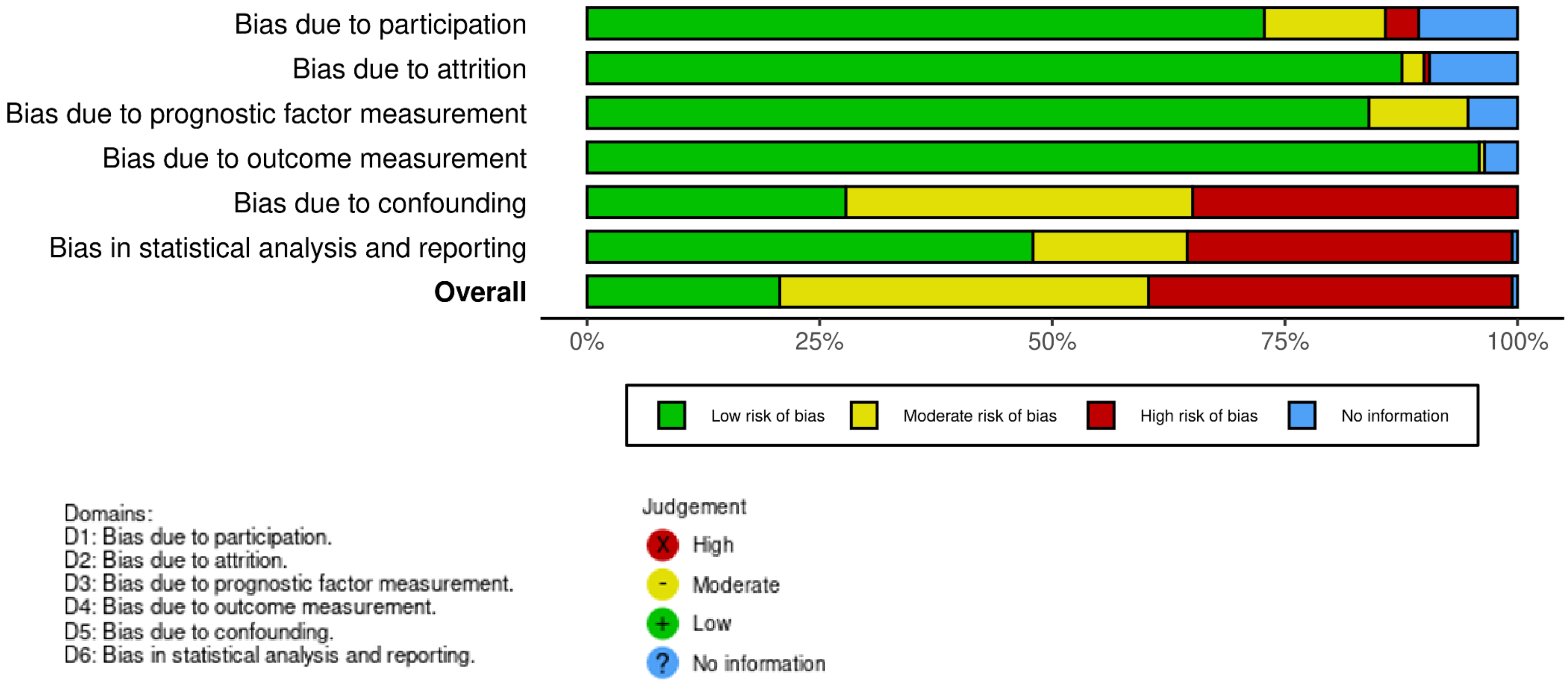

ESM Figure 2: Risk of bias of judgements within each bias domain

Risk of bias was visualized by using the robvis visualization tool.[2635]

A) Death

No data

B) Severity of COVID-19

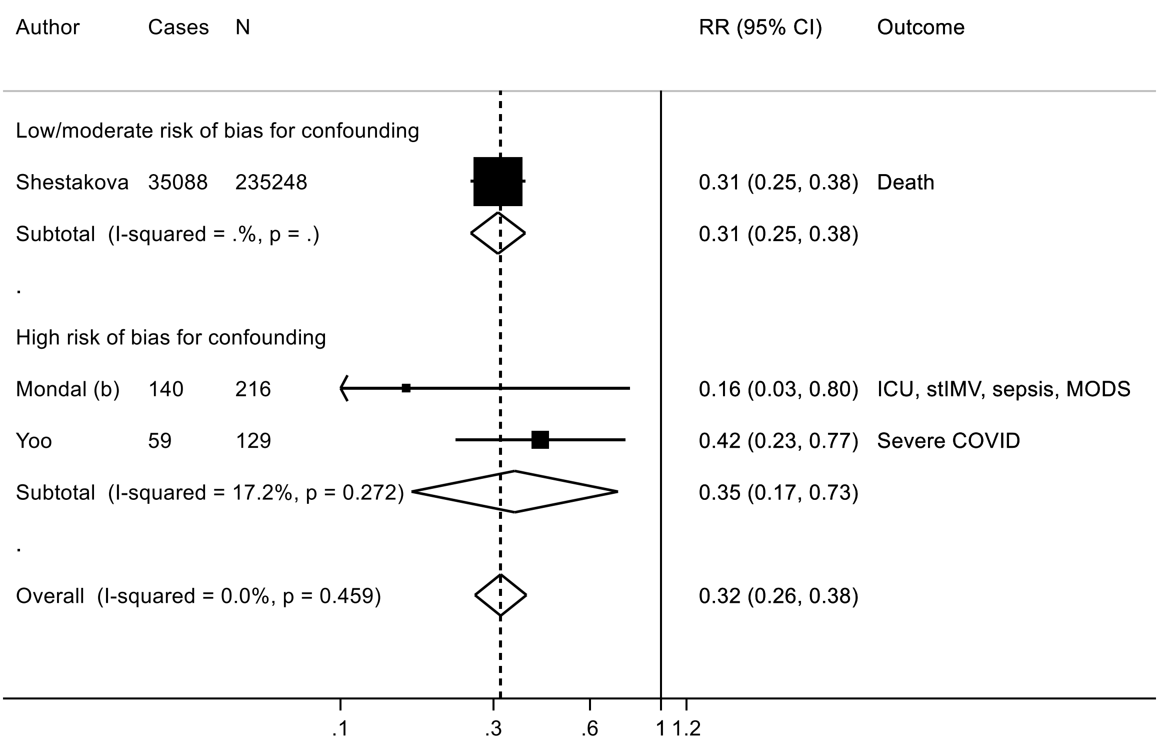

ESM Figure 3: Meta-analysis on **COVID-19 vaccination status, yes vs. no** and A) death and B) and severity of COVID-19 in patients with diabetes and COVID-19

A) Death

No data

B) Severity of COVID-19

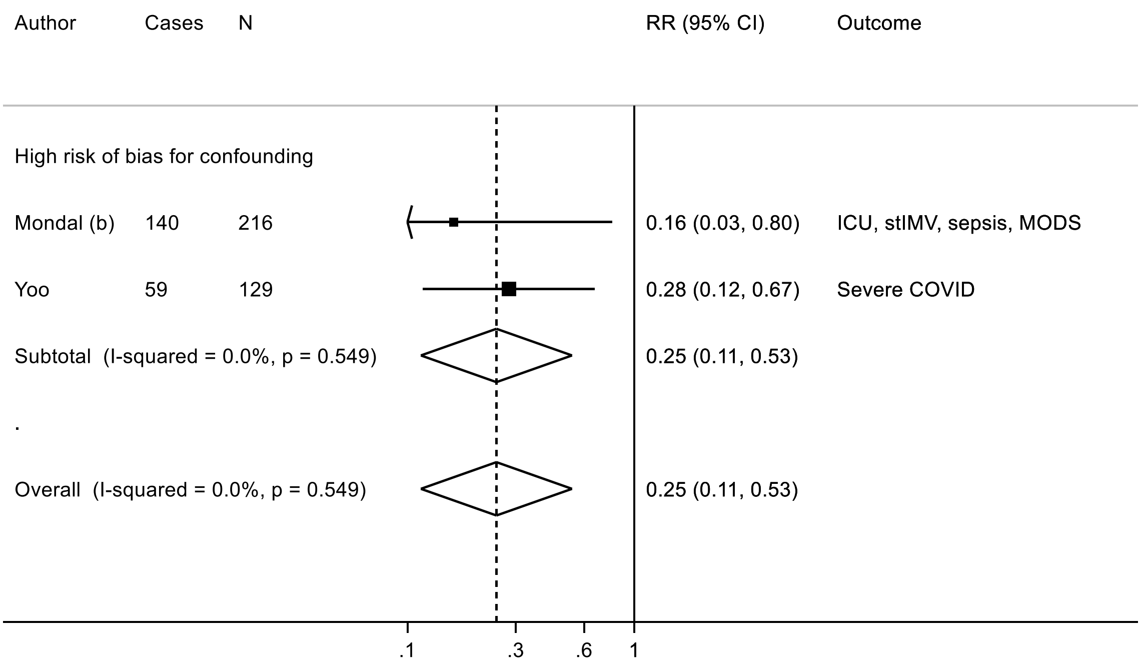

ESM Figure 4: Meta-analysis on **COVID-19 vaccination status, two doses vs. none** and A) death and B) and severity of COVID-19 in patients with diabetes and COVID-19

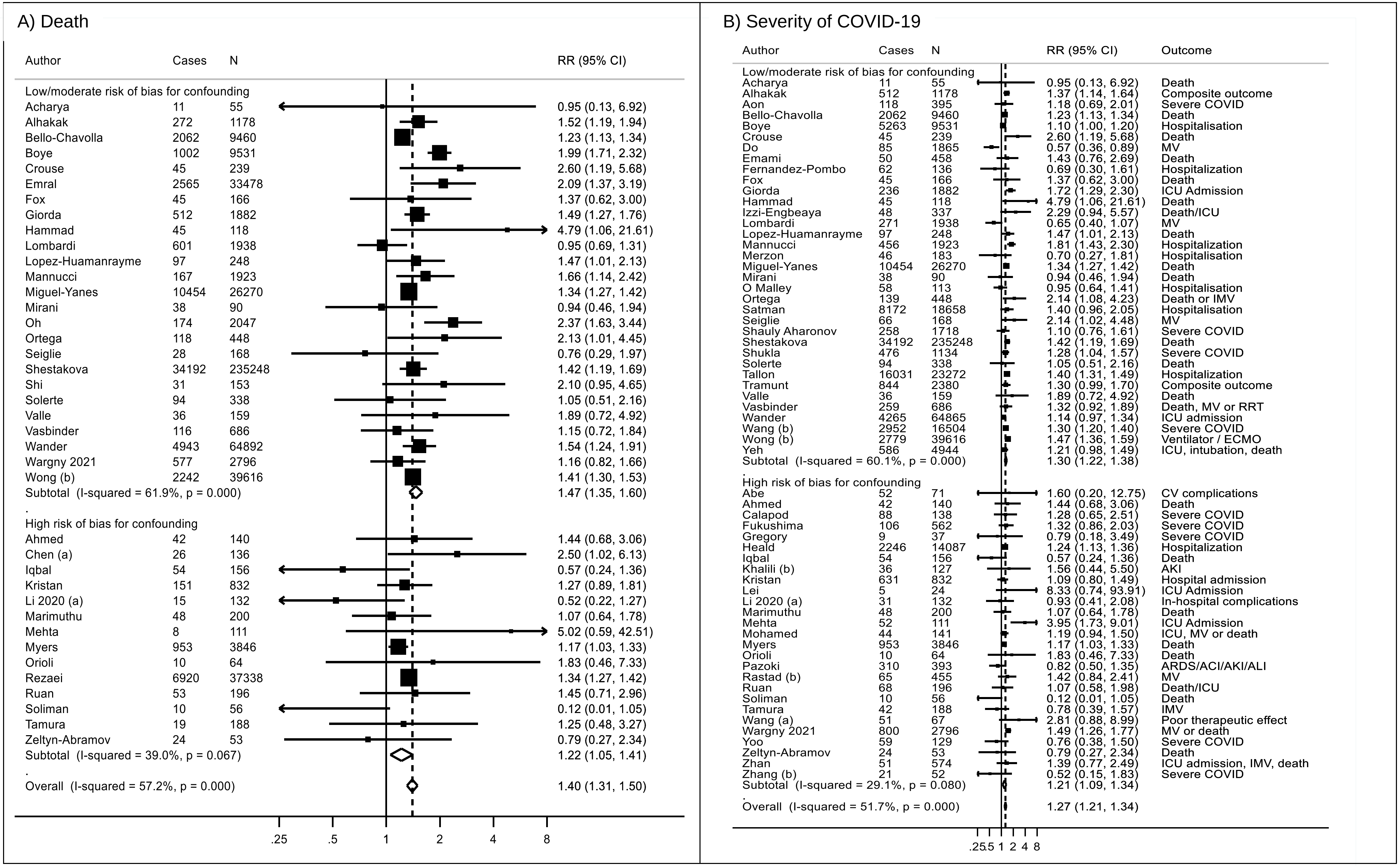

ESM Figure 5: Meta-analysis on **men compared to women** and A) death and B) severity of COVID-19 in patients with diabetes and COVID-19

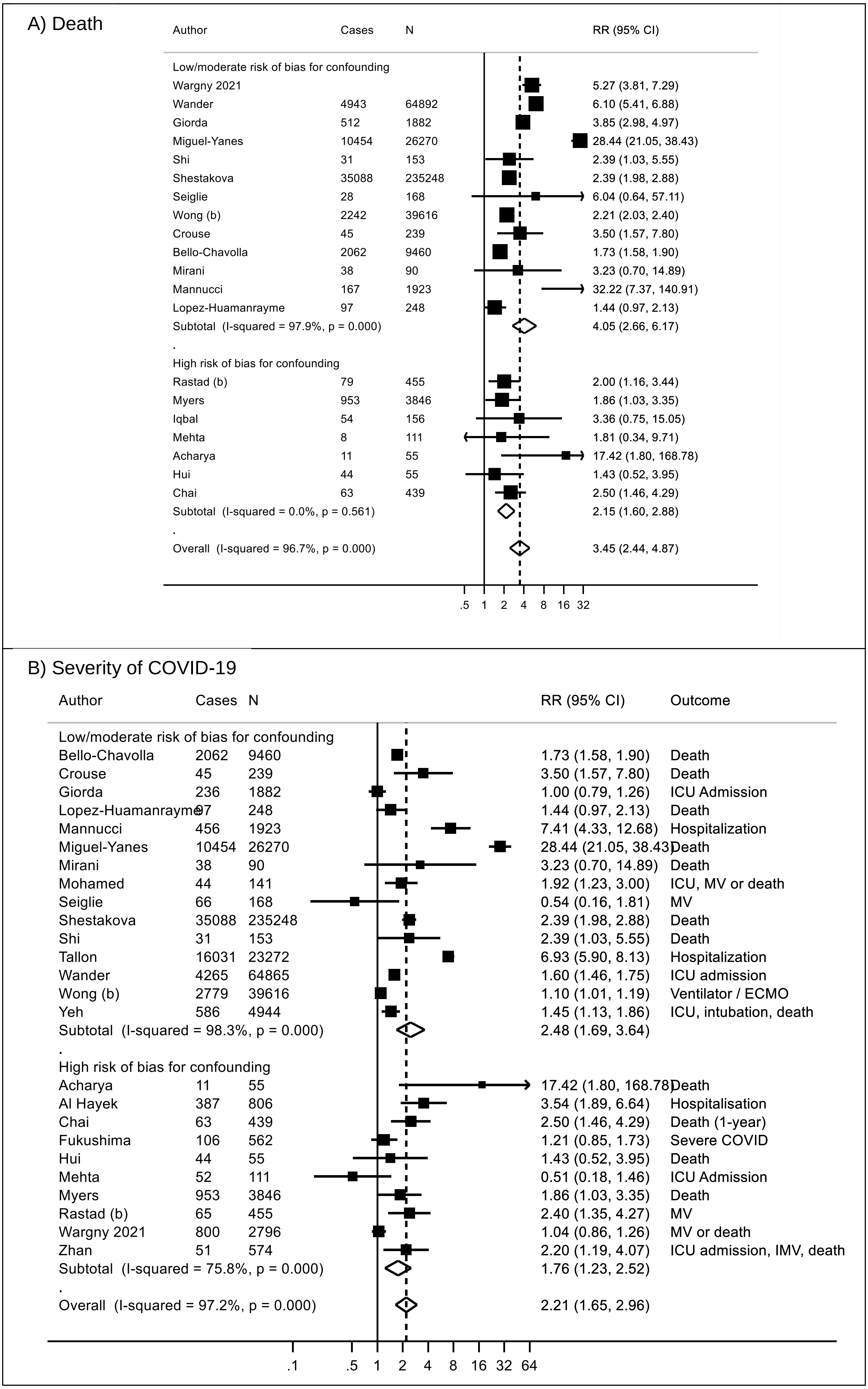

ESM Figure 6: Meta-analysis on **age ≥65 years** and A) death and B) and severity of COVID-19 in patients with diabetes and COVID-19

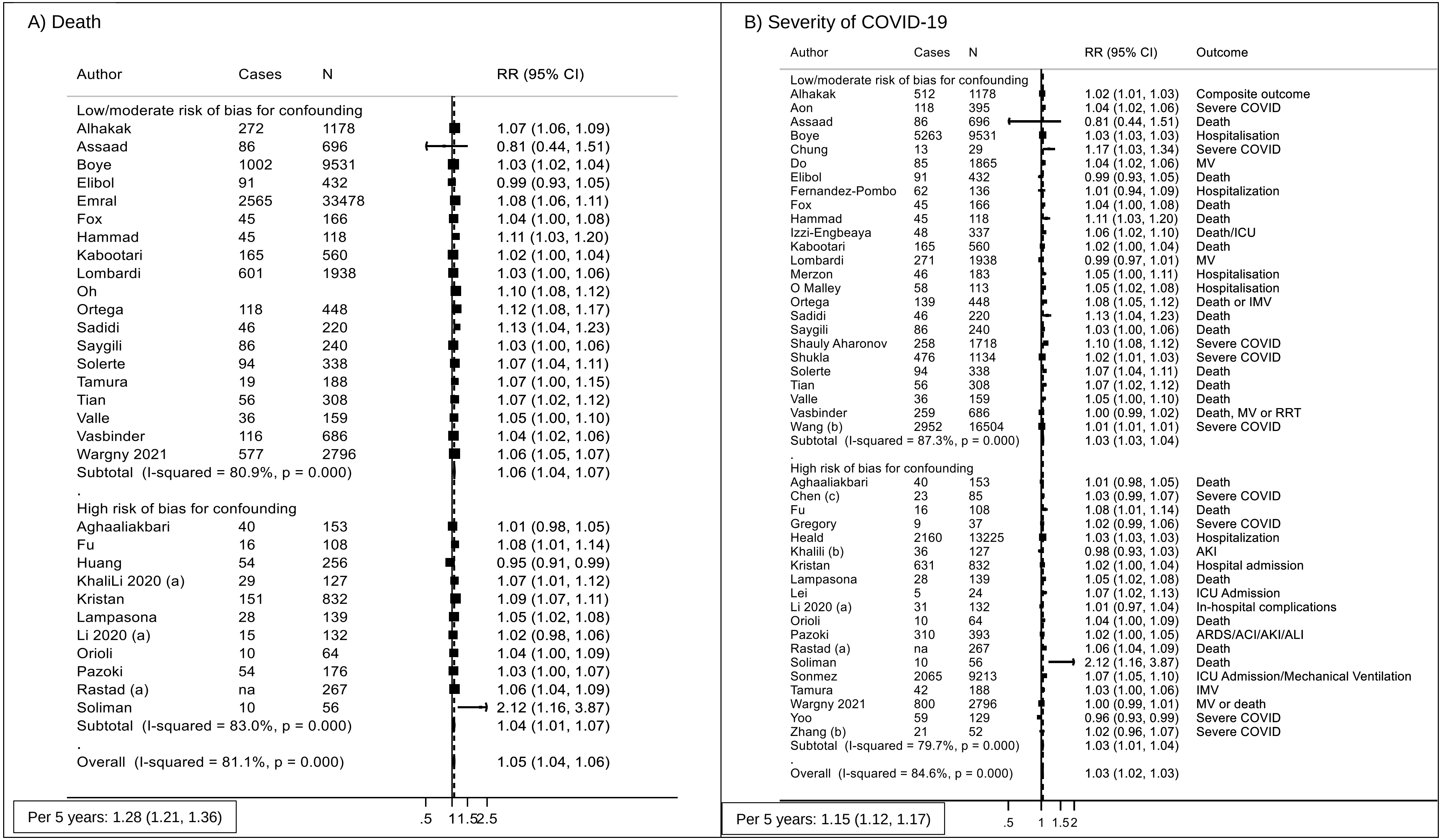

ESM Figure 7: Meta-analysis on **age per 1 year** and A) death and B) severity of COVID-19 in patients with diabetes and COVID-19

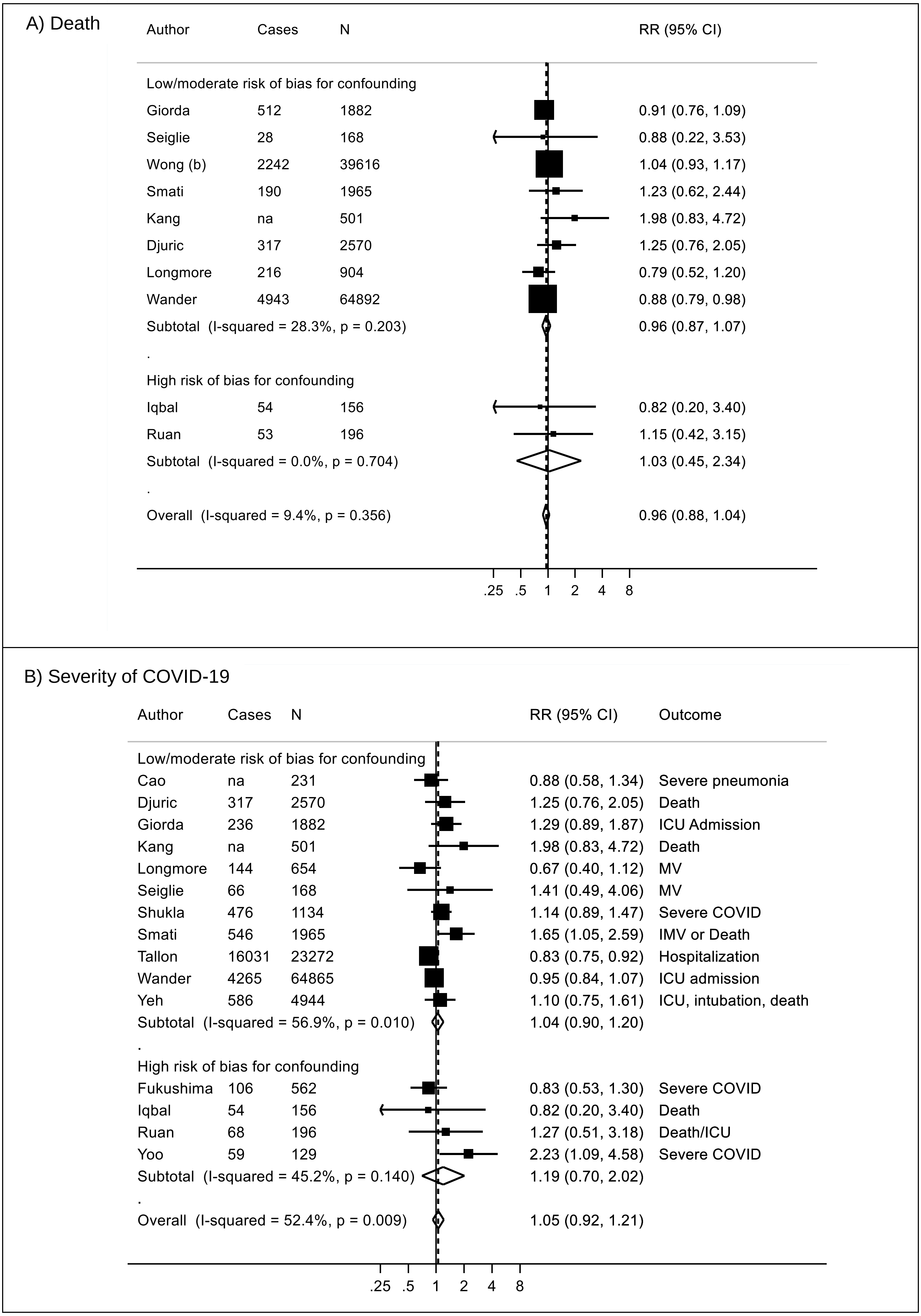

ESM Figure 8: Meta-analysis on **overweight** compared to normal weight and A) death and B) severity of COVID-19 in patients with diabetes and COVID-19

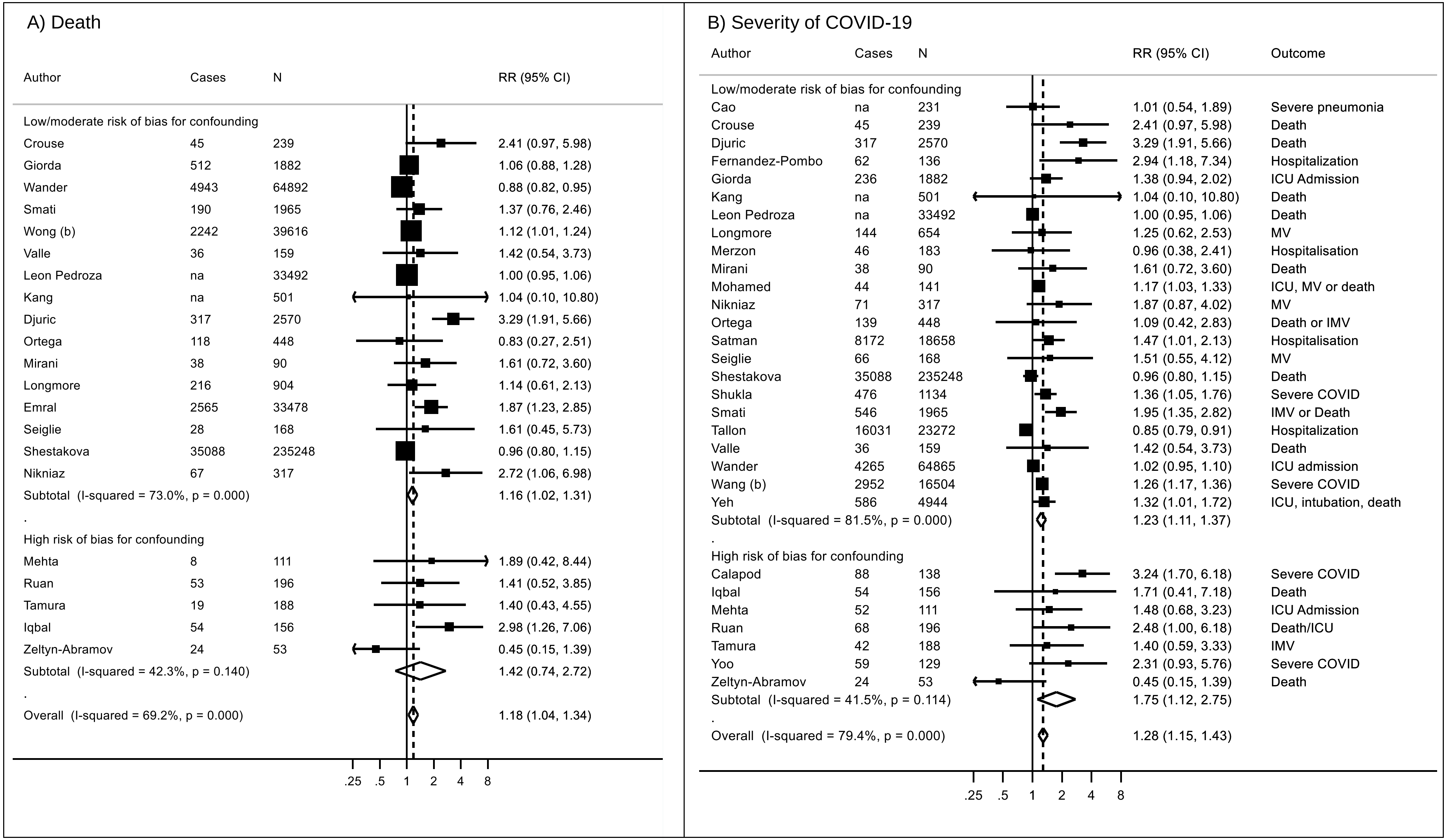

ESM Figure 9: Meta-analysis on **obesity** compared to normal weight and A) death and B) severity of COVID-19 in patients with diabetes and COVID-19

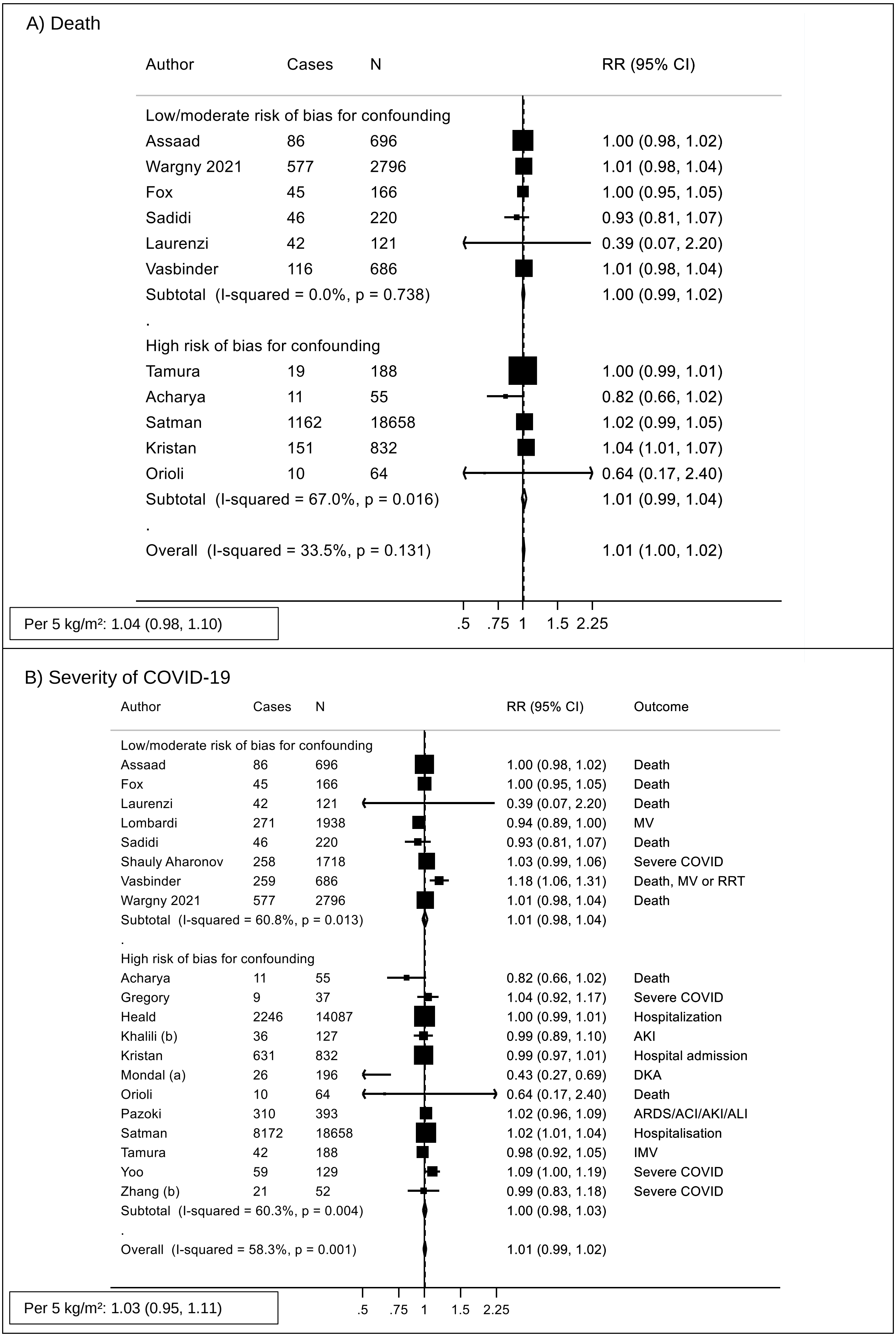

ESM Figure 10: Meta-analysis on **BMI**, per 1 kg/m² increase and A) death and B) severity of COVID-19 in patients with diabetes and COVID-19

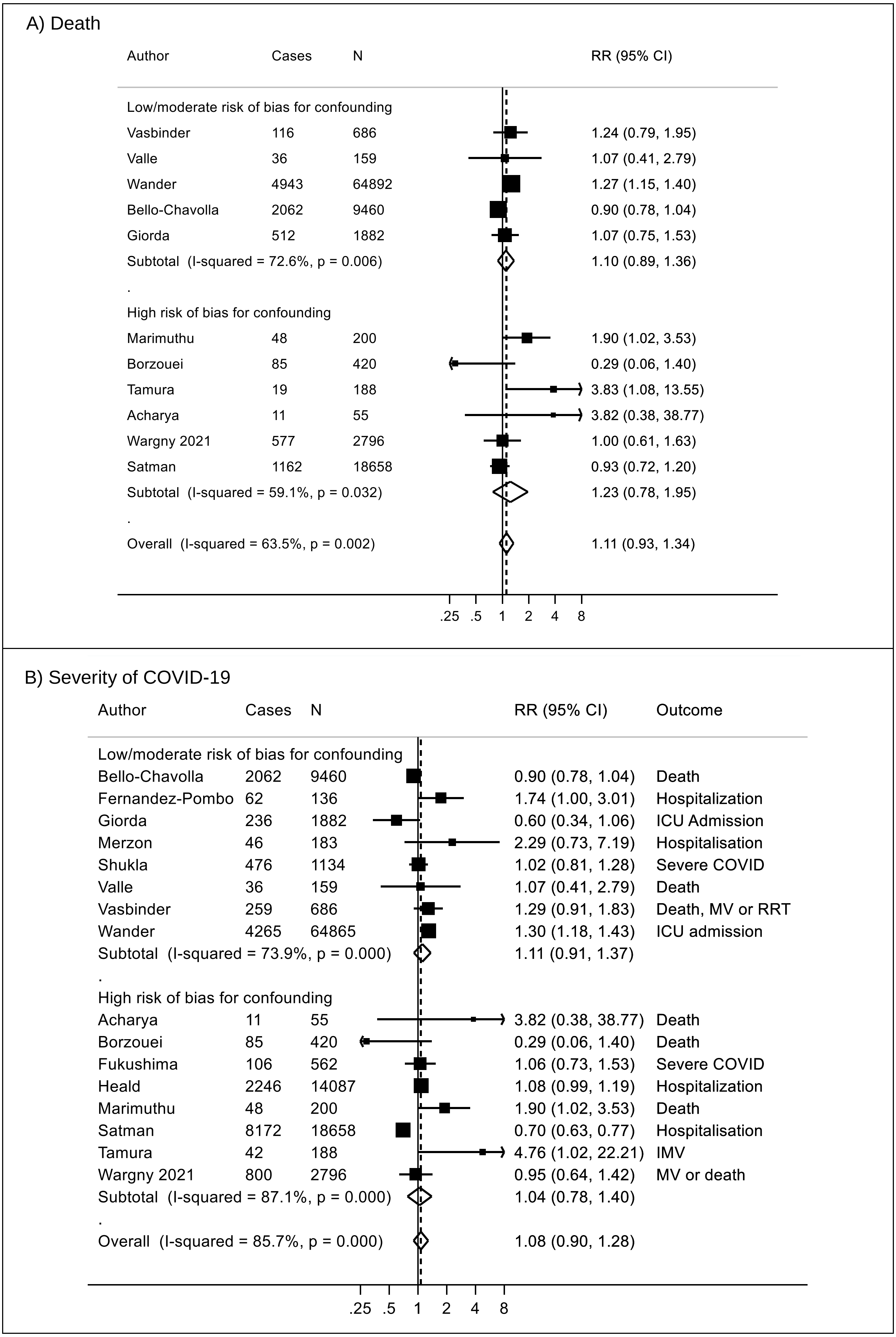

ESM Figure 11: Meta-analysis on **smoking** compared to non-smoking and A) death and B) severity of COVID-19 in patients with diabetes and COVID-19

A) Death

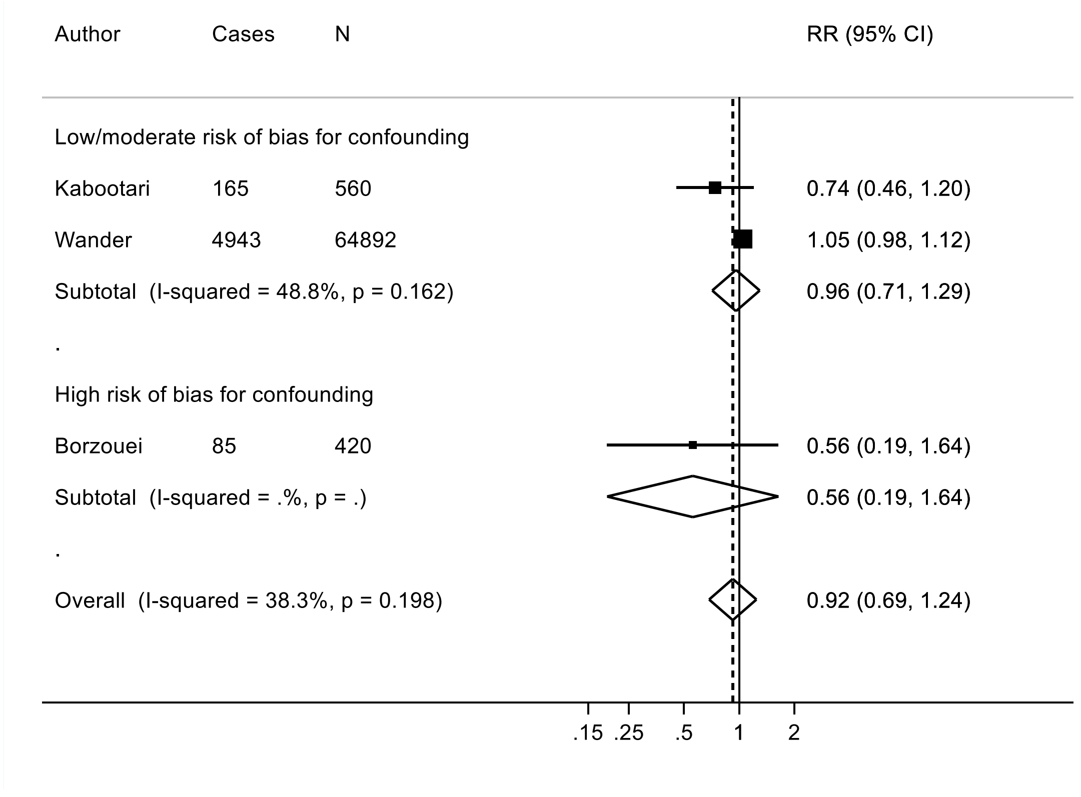

B) Severity of COVID-19

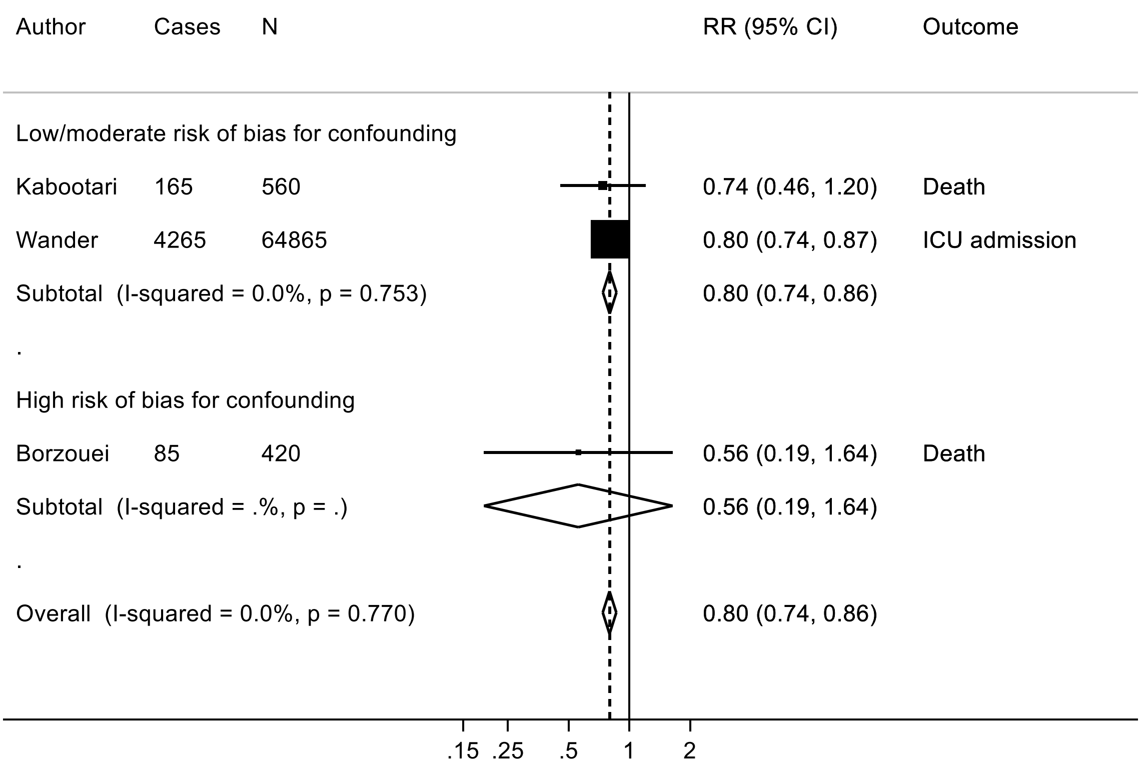

ESM Figure 12: Meta-analysis on **area of residence** (rural compared to urban) and A) death and B) severity of COVID-19 in patients with diabetes and COVID-19

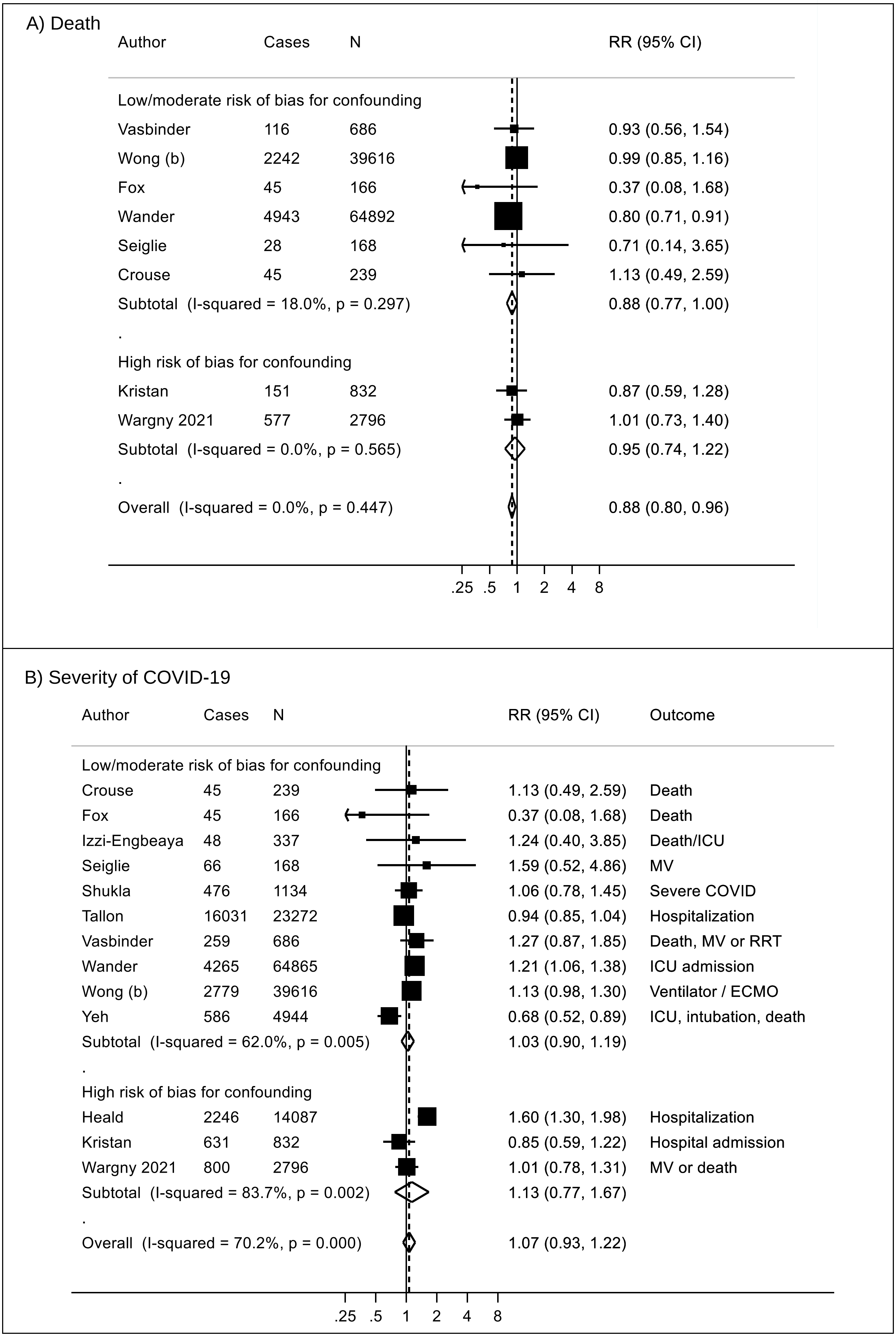

ESM Figure 13: Meta-analysis on **ethnicity (black vs. non-Hispanic white)** and A) death and B) severity of COVID-19 in patients with diabetes and COVID-19

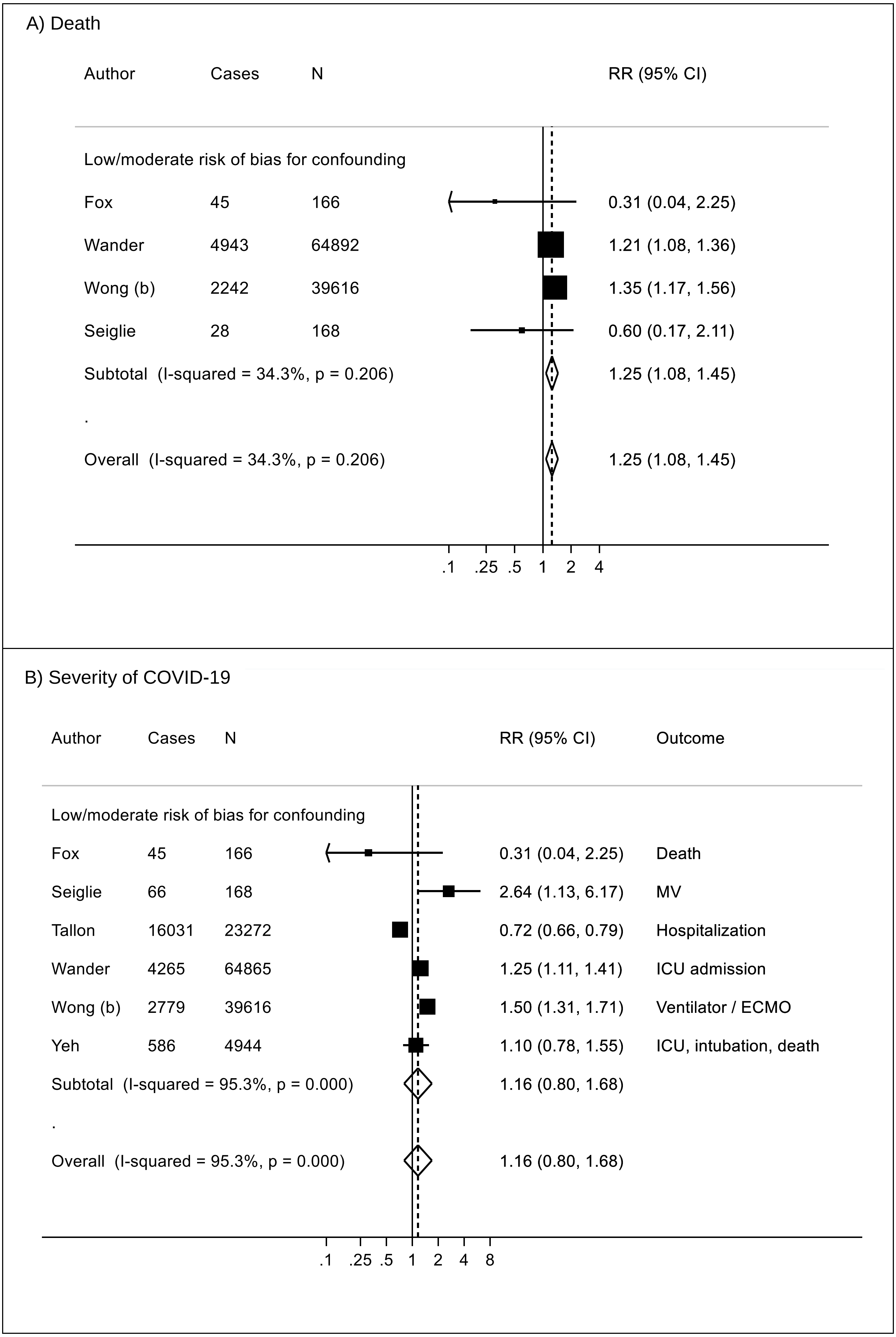

ESM Figure 14: Meta-analysis on **ethnicity (Hispanic vs. non-Hispanic white)** and A) death and B) severity of COVID-19 in patients with diabetes and COVID-19

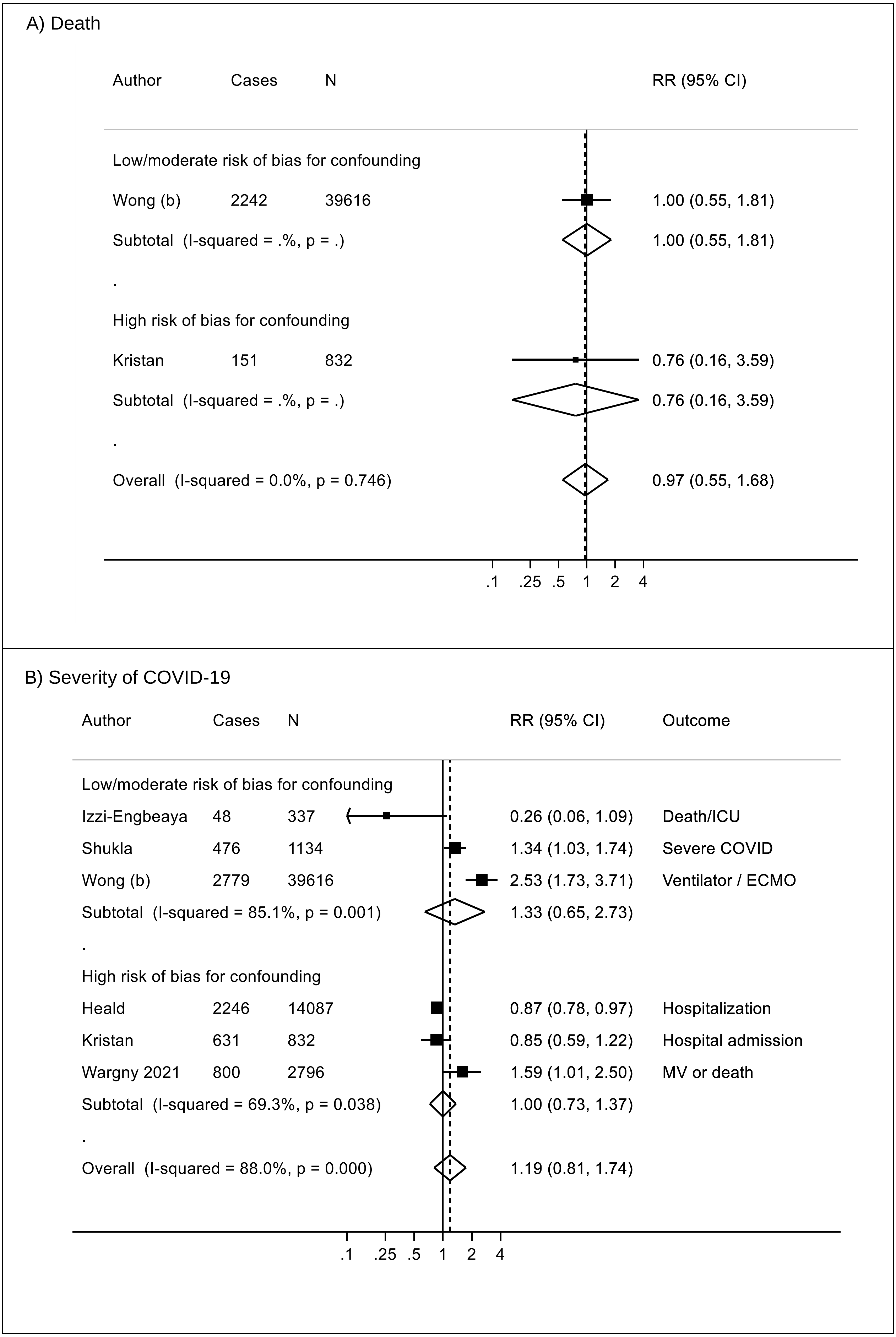

ESM Figure 15: Meta-analysis on **ethnicity (Asian vs. non-Hispanic white)** and A) death and B) severity of COVID-19 in patients with diabetes and COVID-19

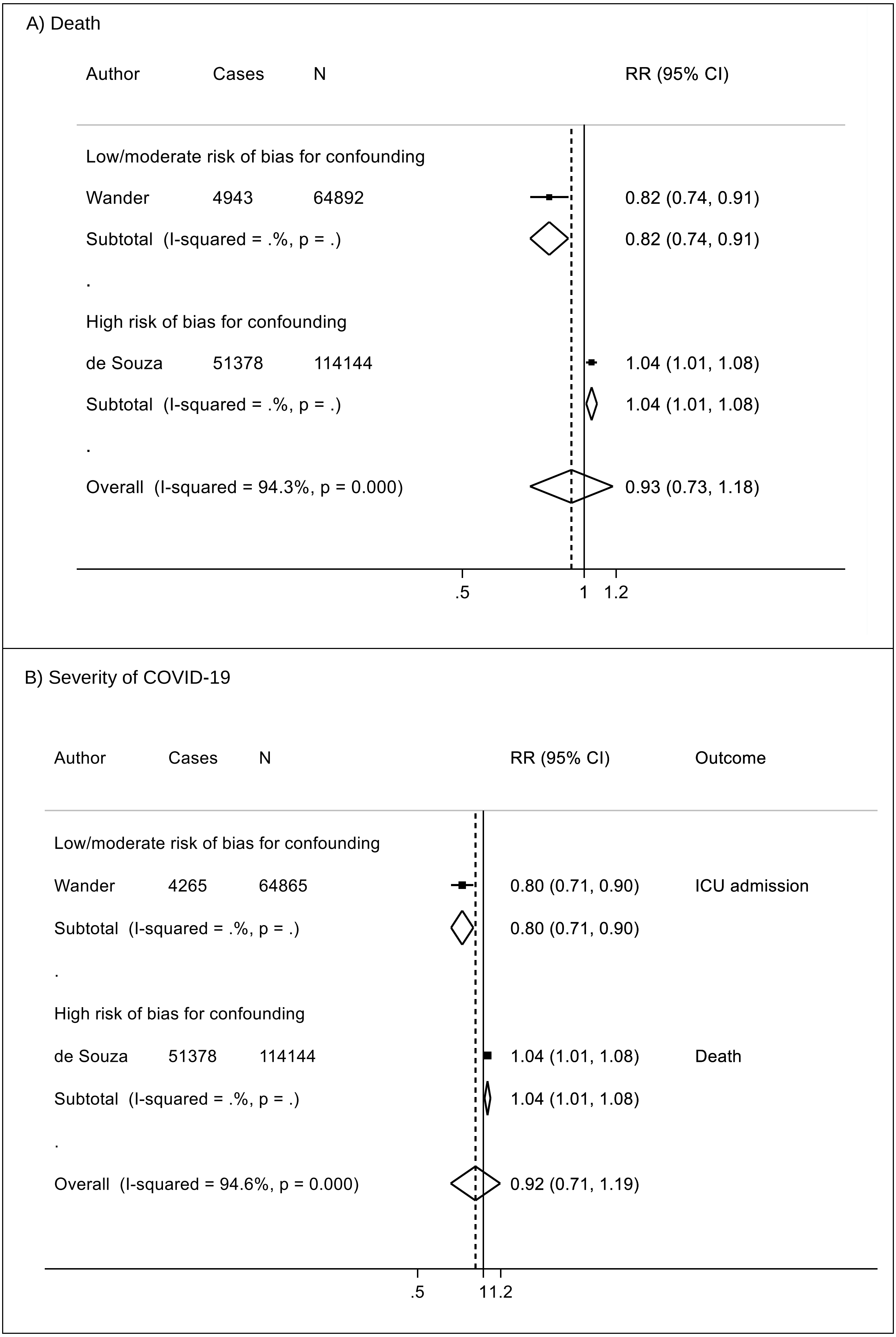

ESM Figure 16: Meta-analysis on **ethnicity (White vs. Non-white)** and A) death and B) severity of COVID-19 in patients with diabetes and COVID-19

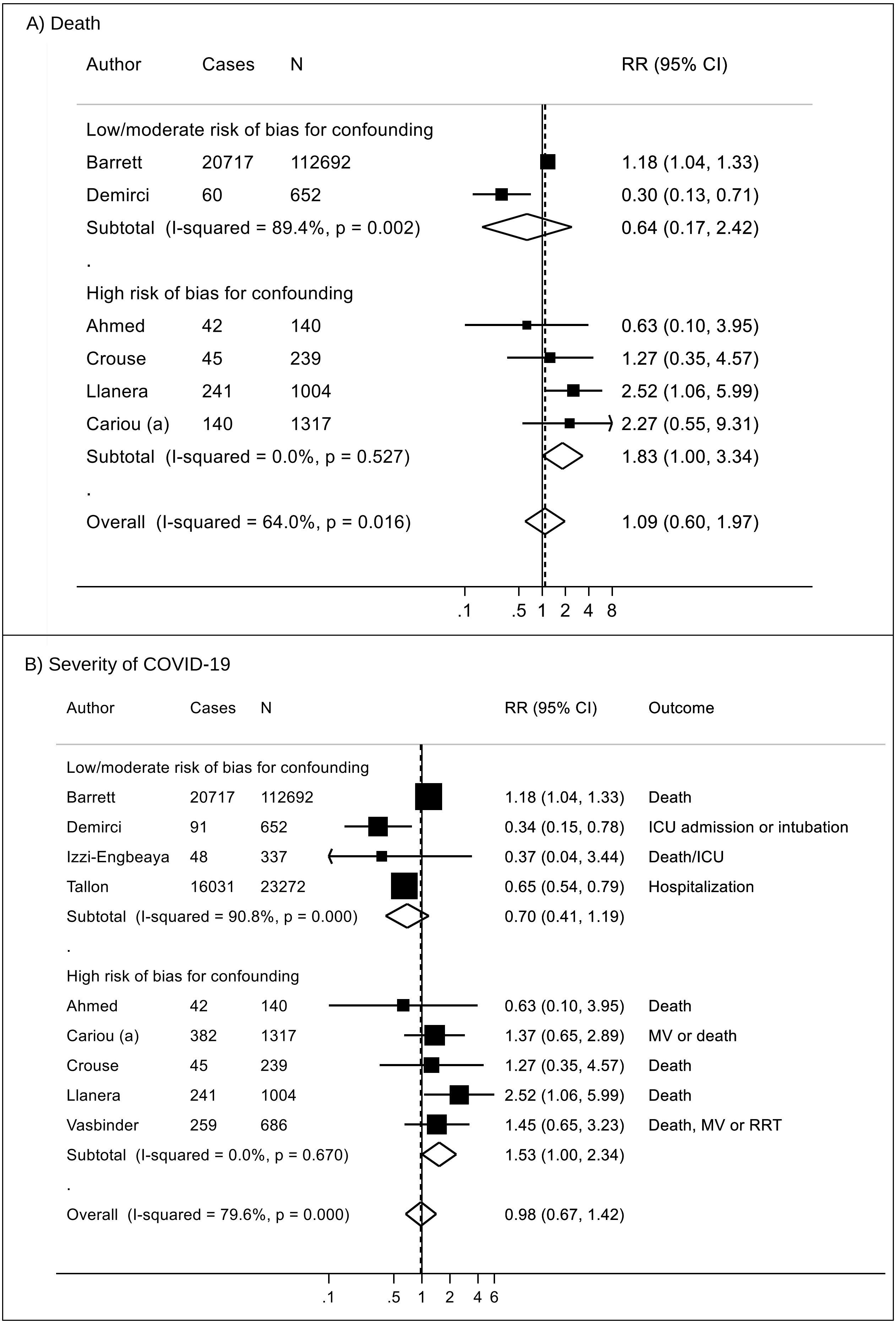

ESM Figure 17: Meta-analysis on **type 2 vs type 1 diabetes** and A) death and B) severity of COVID-19 in patients with diabetes and COVID-19

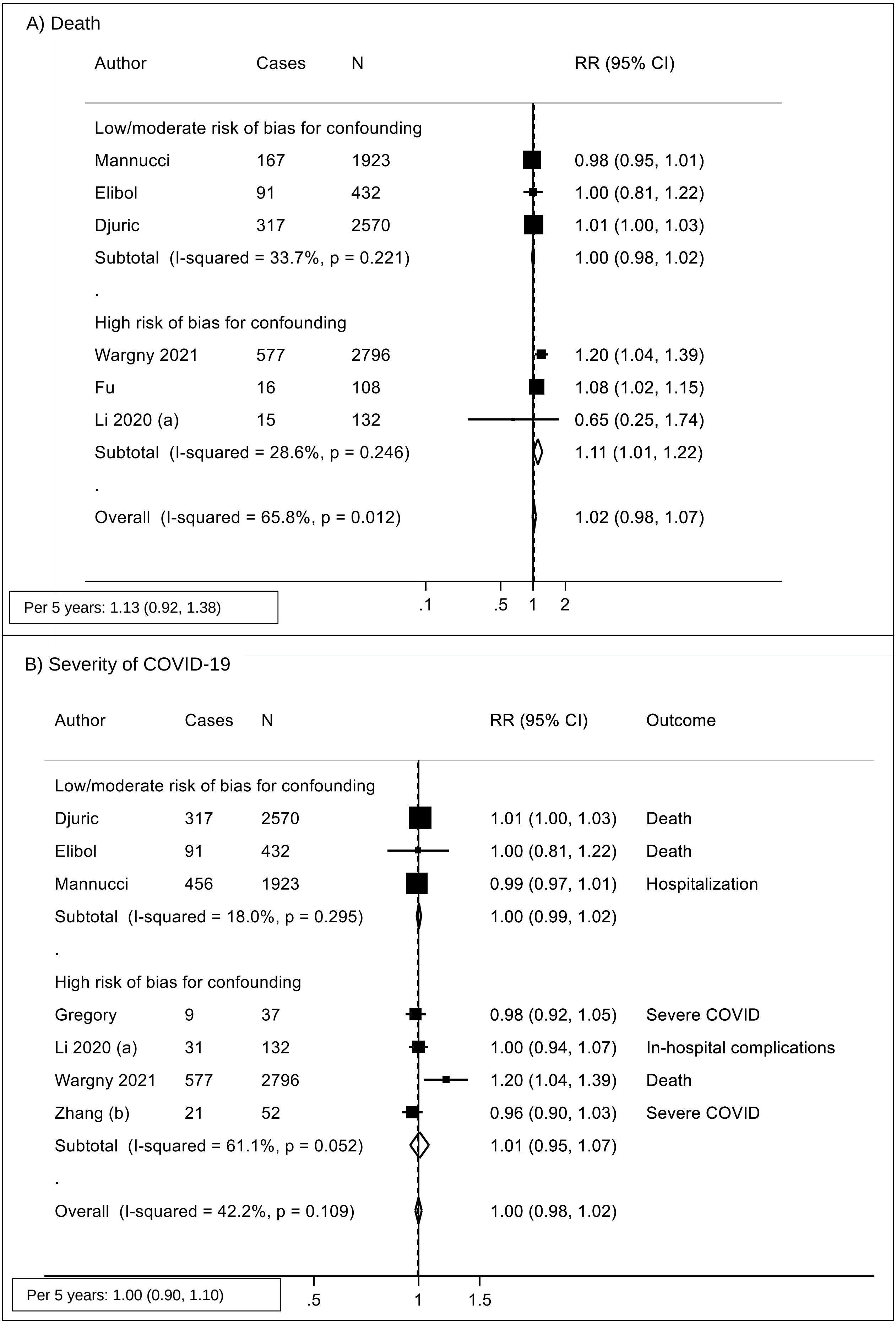

ESM Figure 18: Meta-analysis on **diabetes duration**, per 1 year and A) death and B) severity of COVID-19 in patients with diabetes and COVID-19

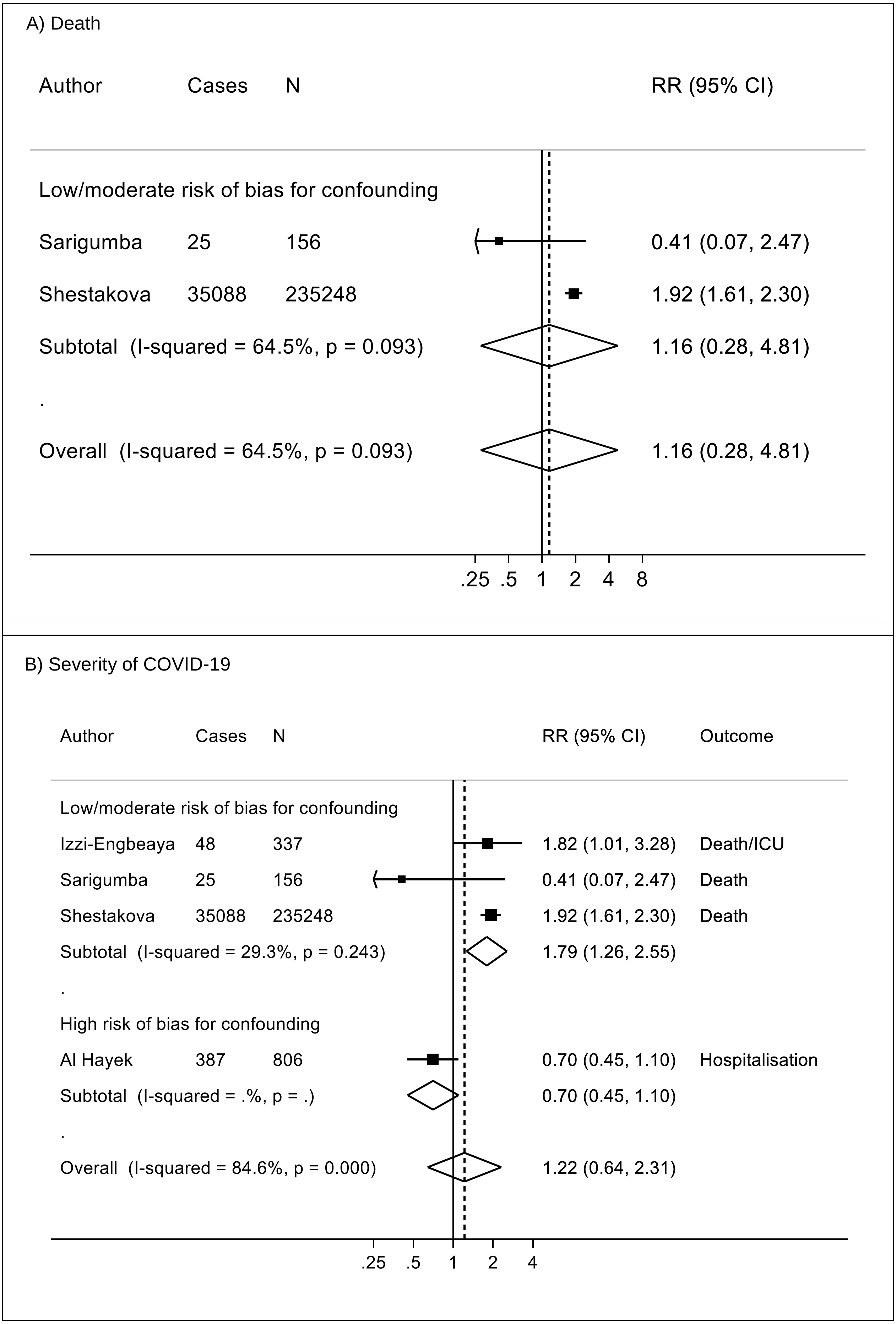

ESM Figure 19: Meta-analysis on **diabetes duration**, **≥10 years** and A) death and B) severity of COVID-19 in patients with diabetes and COVID-19

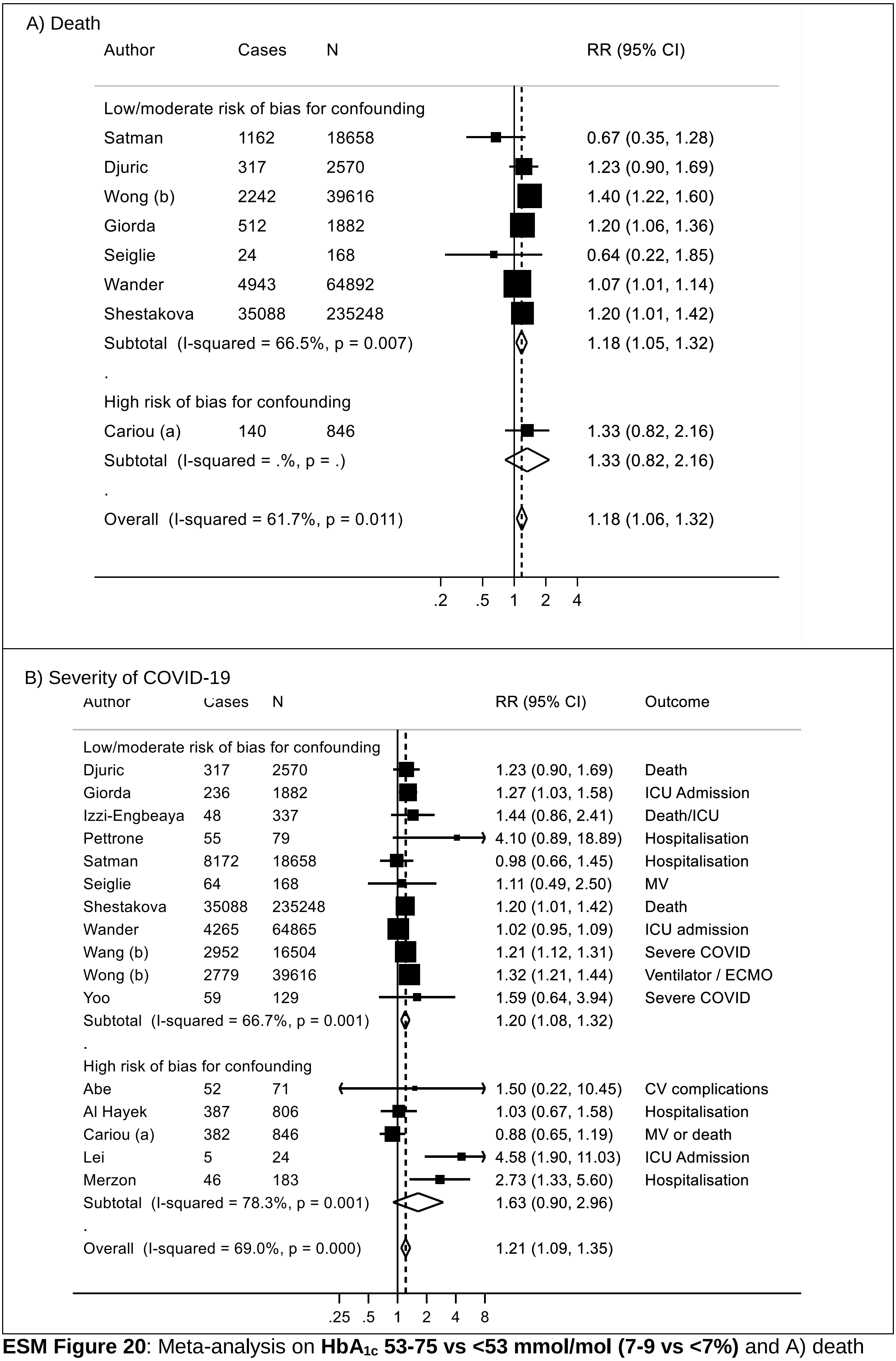

ESM Figure 20: Meta-analysis on **HbA_{1c} 53-75 vs <53 mmol/mol (7-9 vs <7%)** and A) death and B) severity of COVID-19 in patients with diabetes and COVID-19

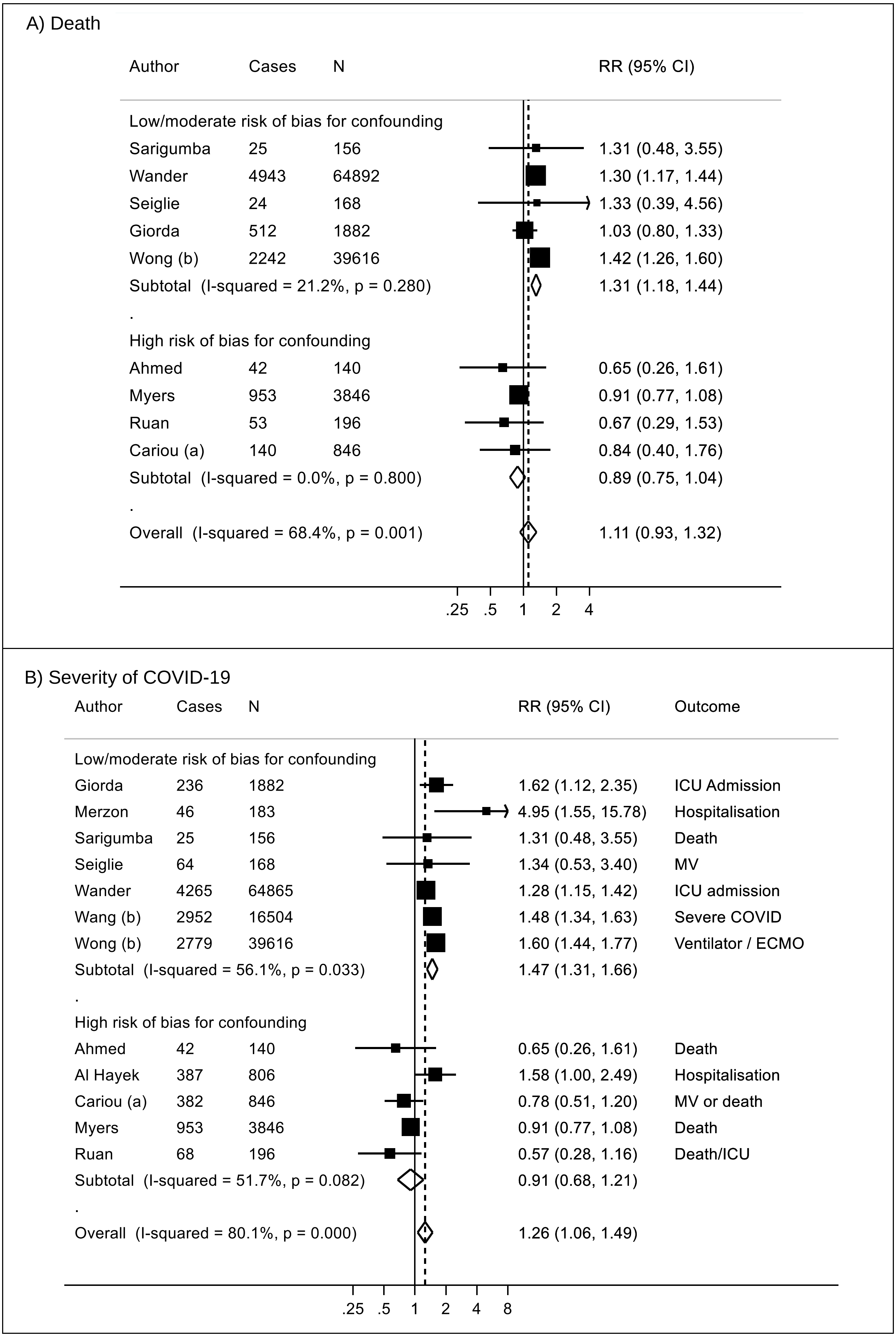

ESM Figure 21: Meta-analysis on **HbA$_{1c}$ ≥75 vs <53 mmol/mol (≥9 vs <7%)** and A) death and B) severity of COVID-19 in patients with diabetes and COVID-19

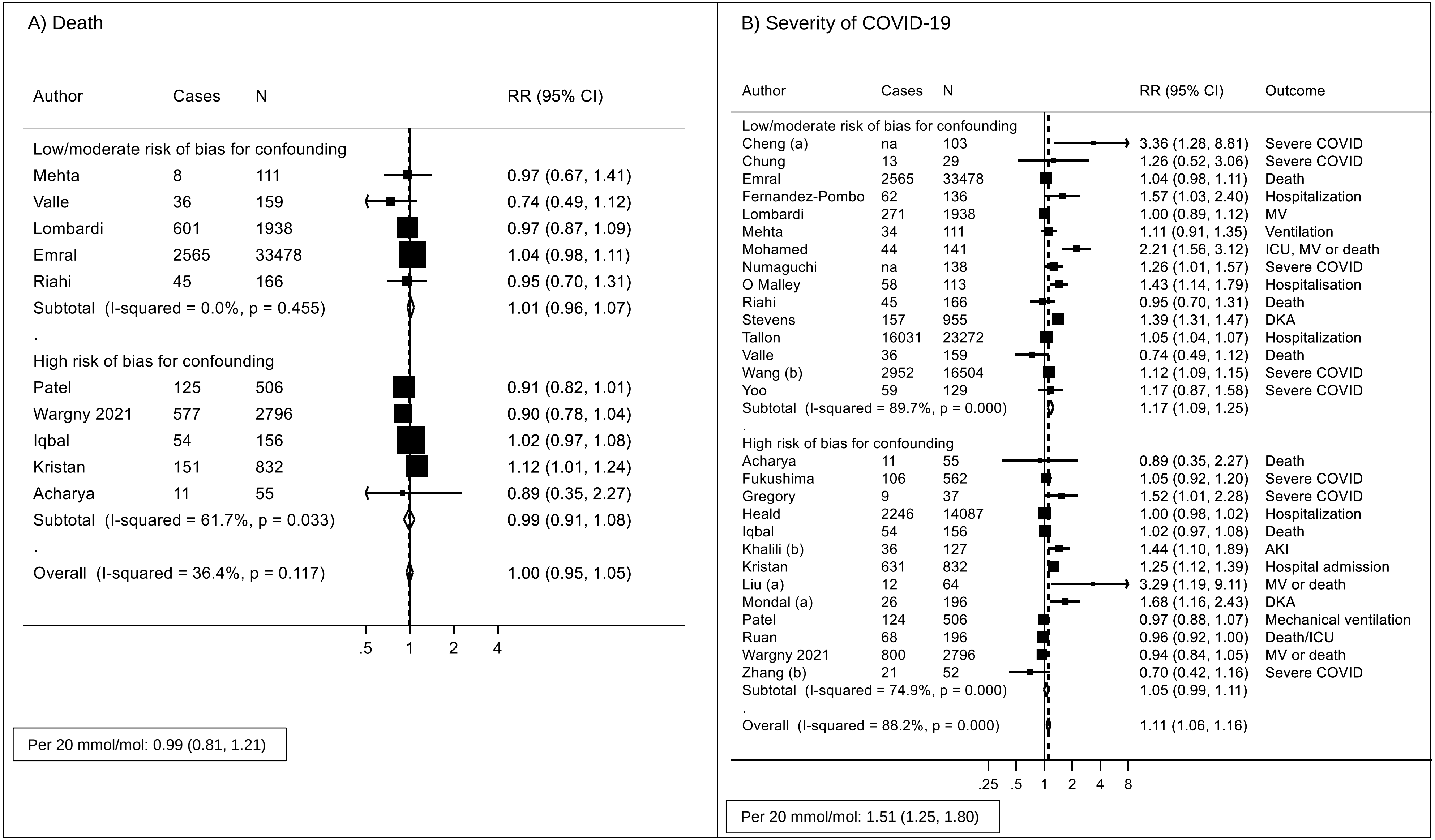

ESM Figure 22: Meta-analysis on **HbA_{1C}, per 1%** increase and A) death and B) severity of COVID-19 in patients with diabetes and COVID-19

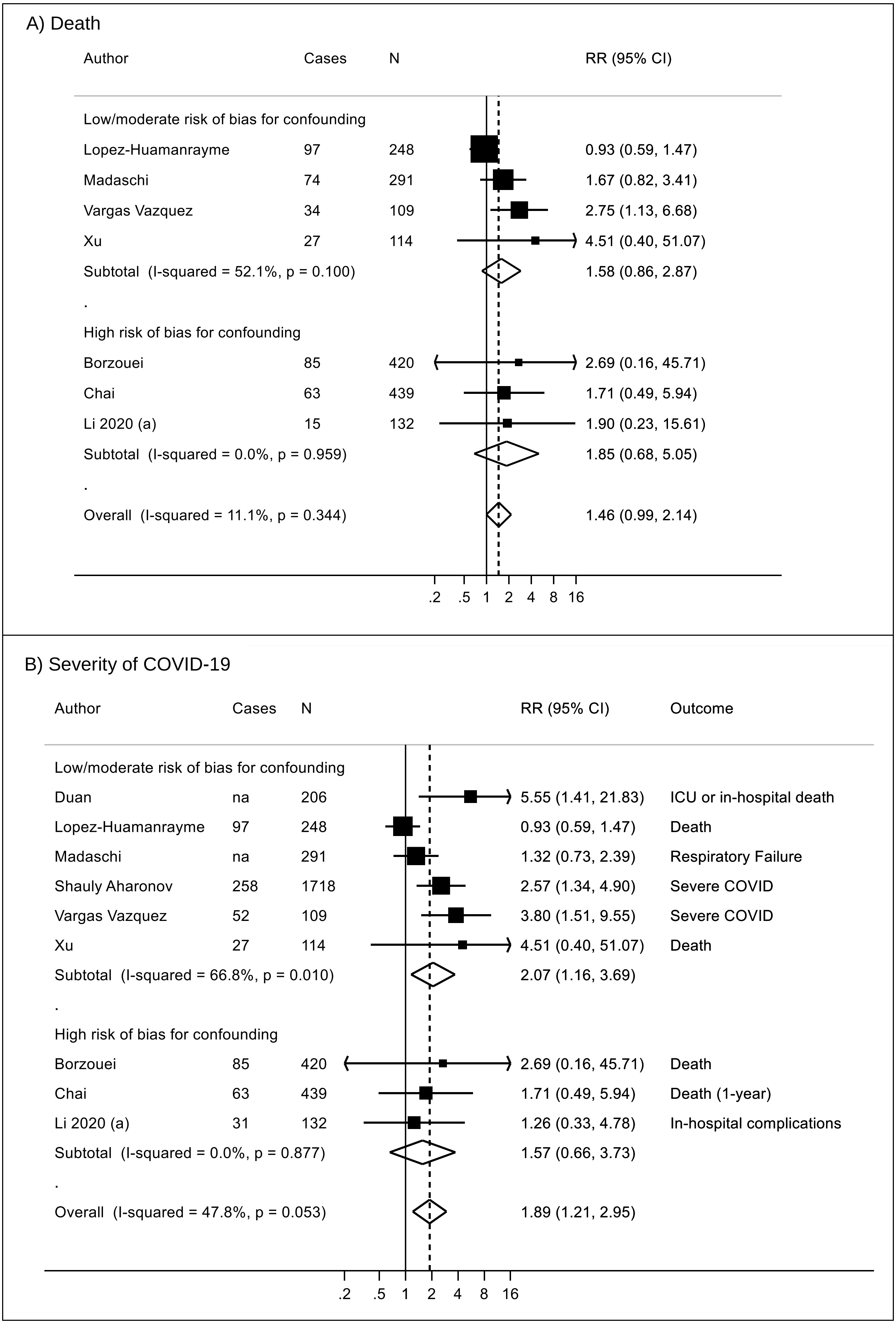

ESM Figure 23: Meta-analysis on **blood glucose ≥6 - <10 mmol/l** at admission and A) death and B) severity of COVID-19in patients with diabetes and COVID-19

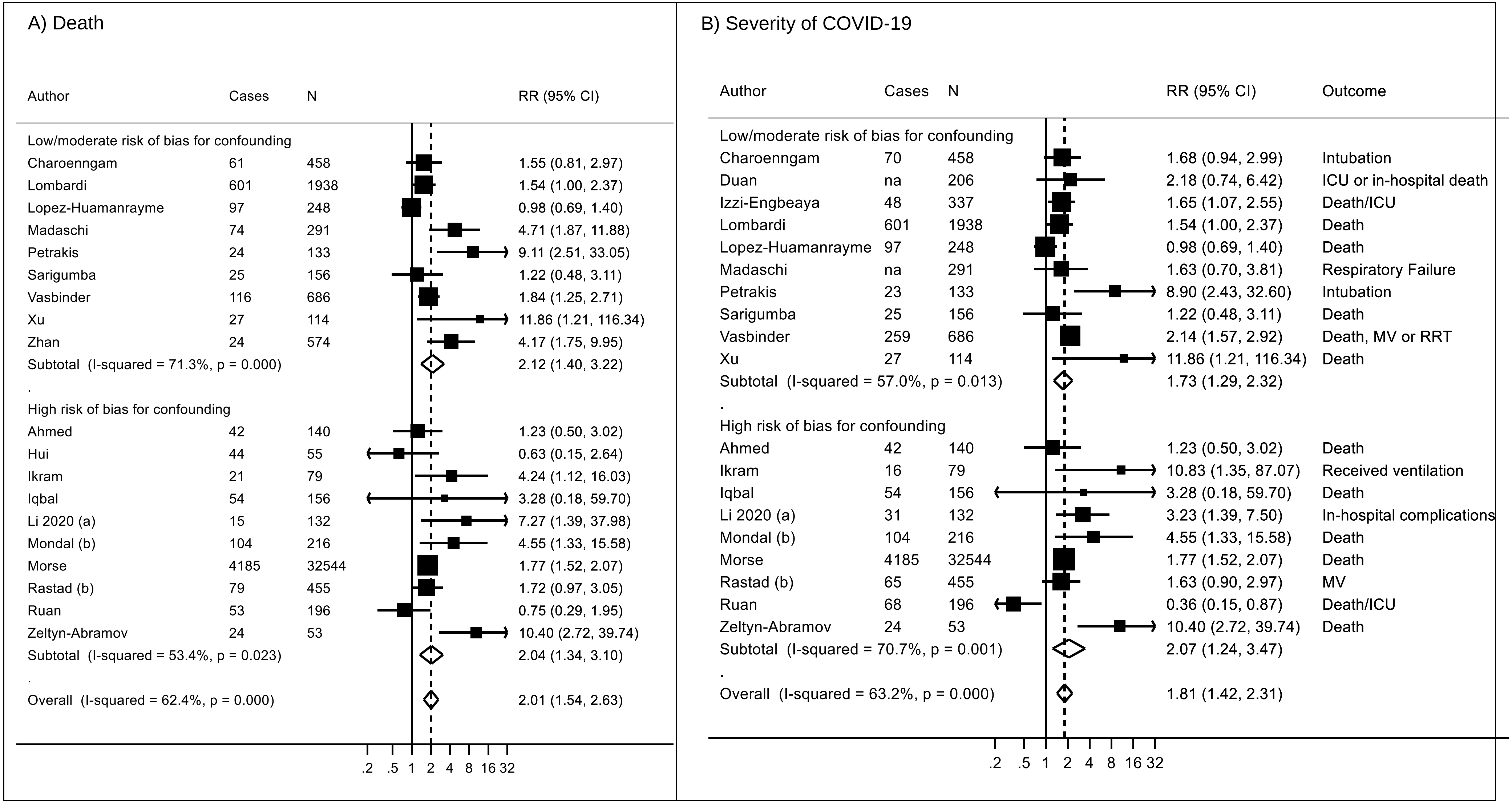

ESM Figure 24 Meta-analysis on **blood glucose ≥10 mmol/l** at admission and A) death and B) severity of COVID-19 in patients with diabetes and COVID-19

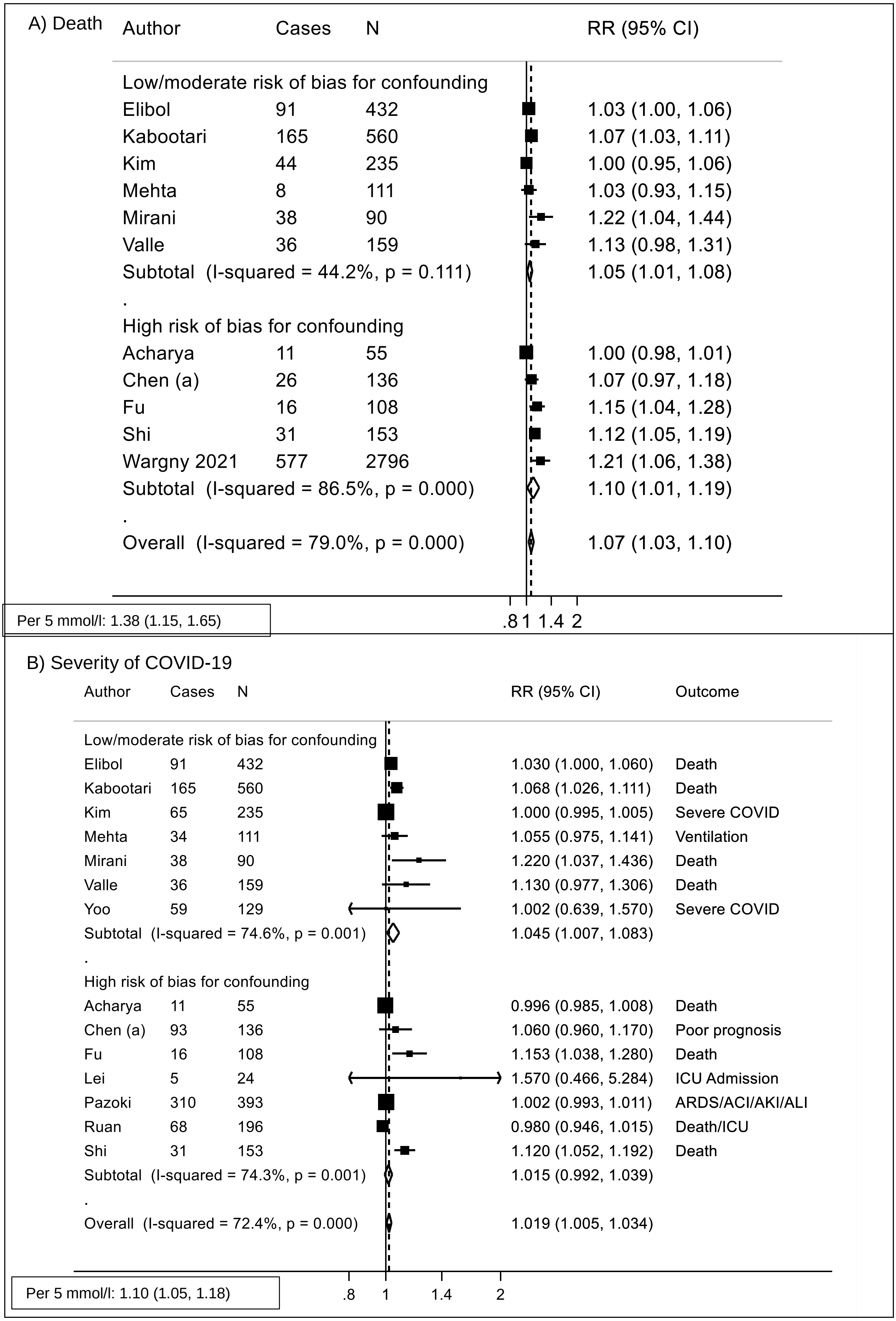

ESM Figure 25: Meta-analysis on **blood glucose per 1 mmol/l increase** at admission and A) death and B) severity of COVID-19 in patients with diabetes and COVID-19

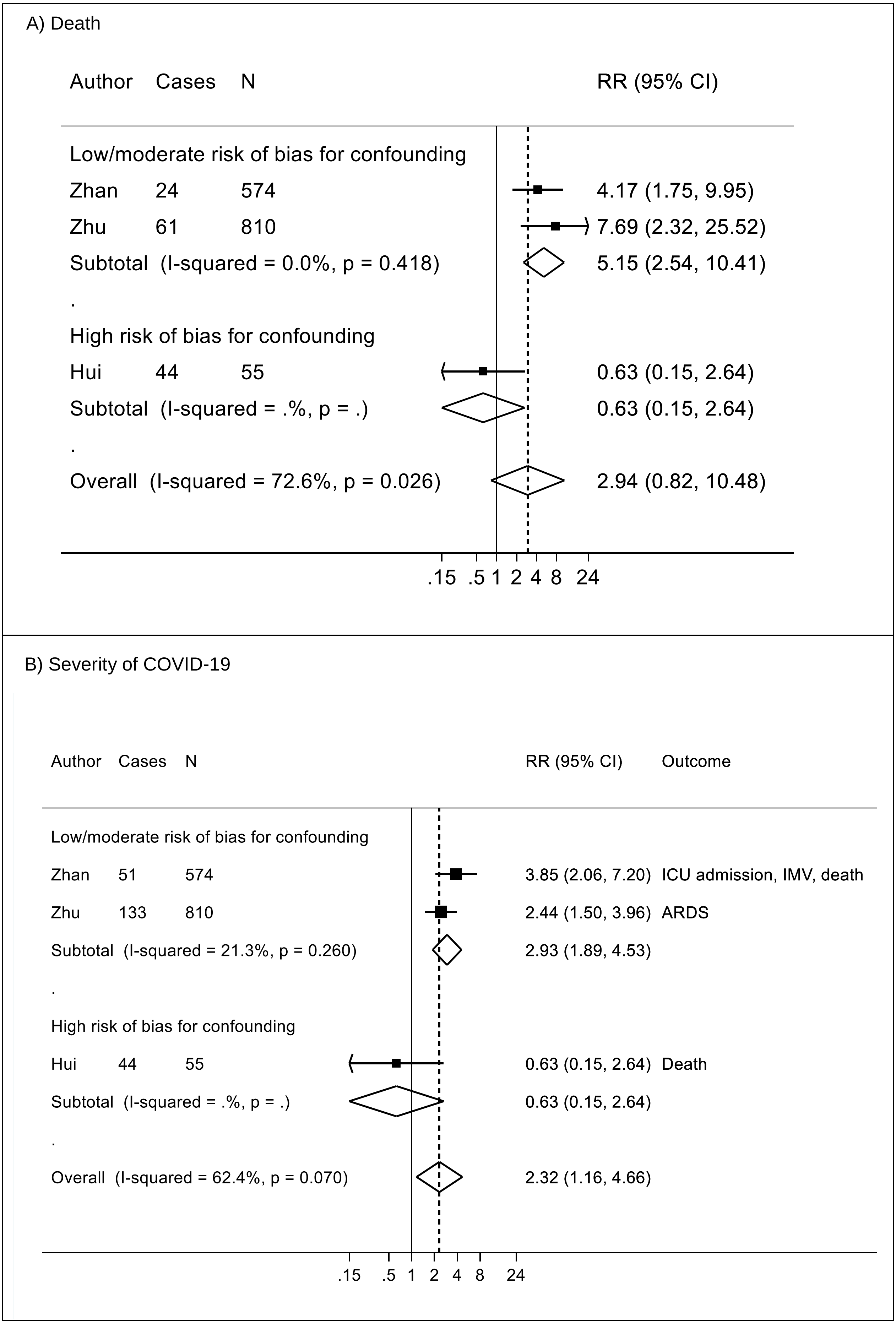

ESM Figure 26: Meta-analysis for **poorly vs. well-controlled at admission** and A) death and B) severity of COVID-19 in patients with diabetes and COVID-19

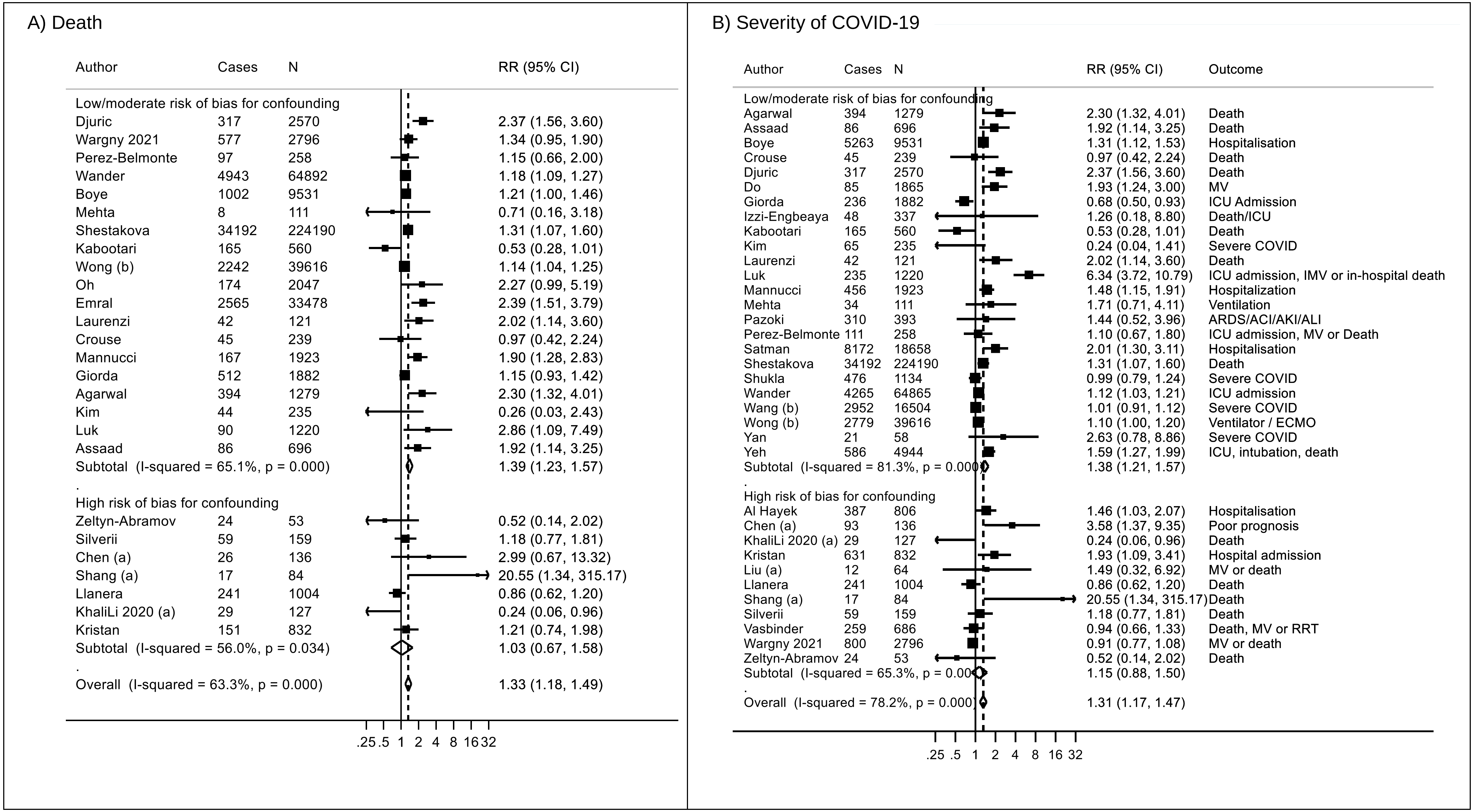

ESM Figure 27: Meta-analysis on **insulin use** compared to non-use and A) death and B) severity of COVID-19 in patients with diabetes and COVID-19

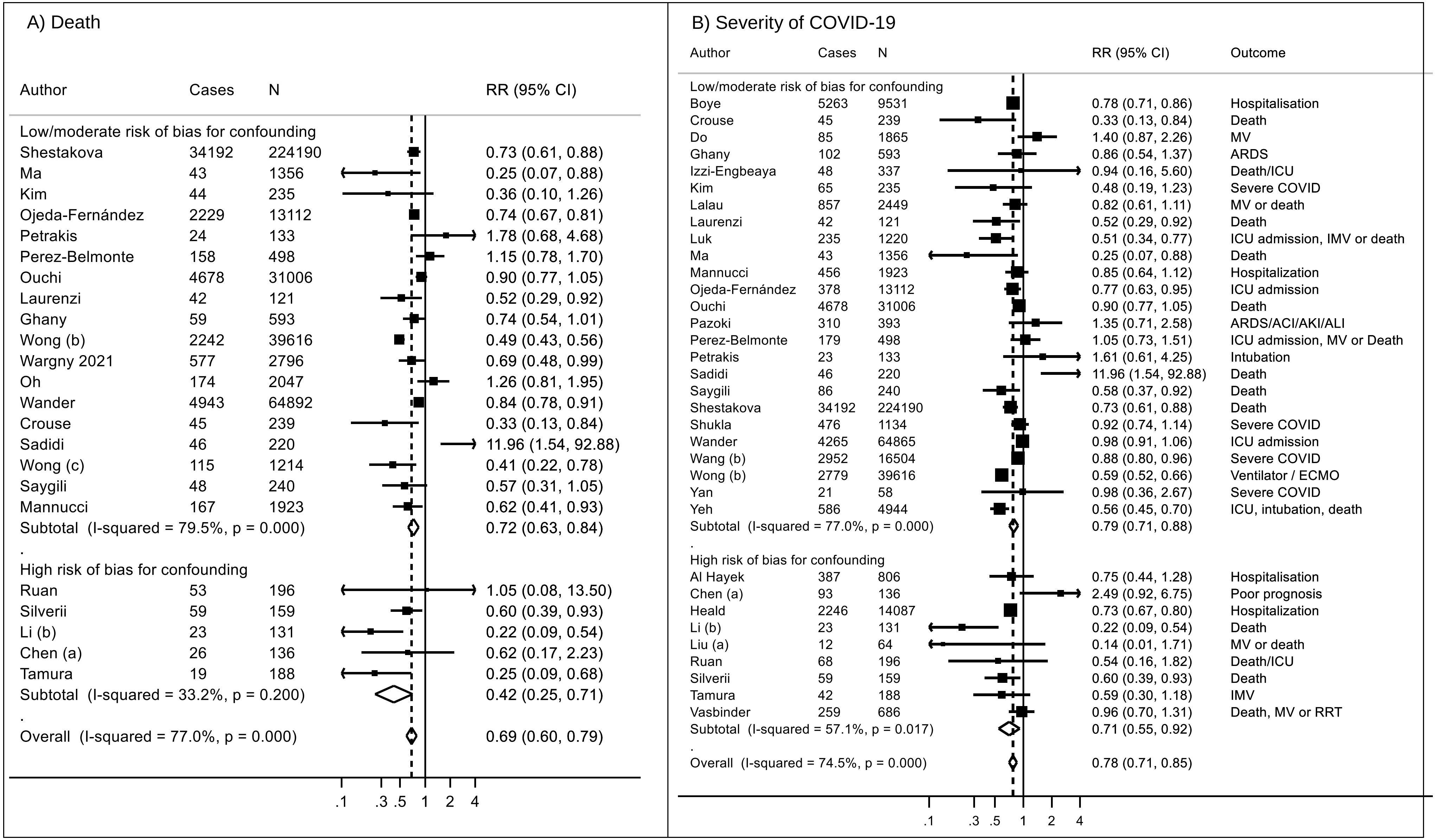

ESM Figure 28: Meta-analysis on **metformin use** compared to non-use and A) death and B) severity of COVID-19 in patients with diabetes and COVID-19

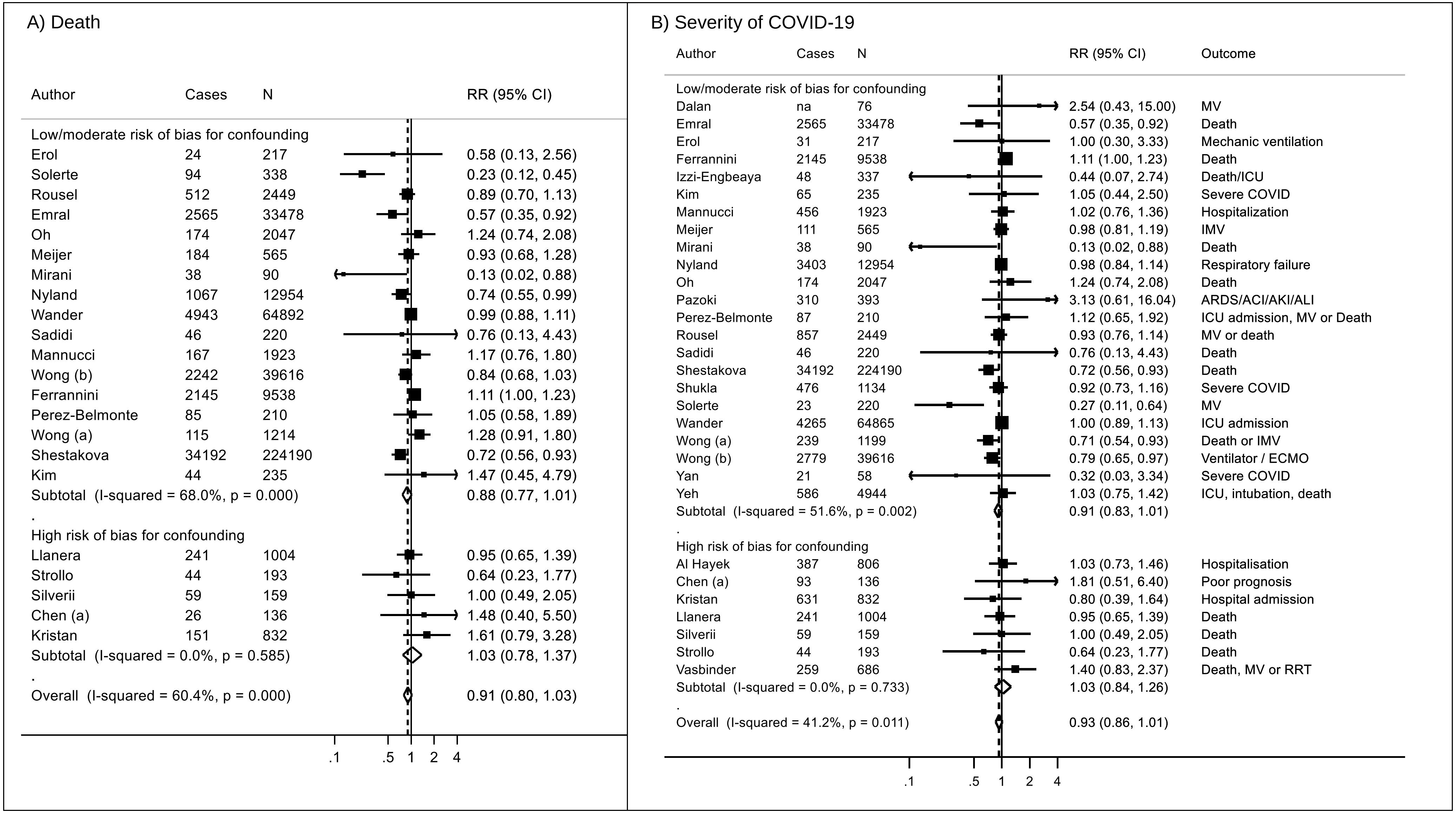

ESM Figure 29: Meta-analysis on **DPP-4-inhibitors use** compared to non-use and A) death and B) severity of COVID-19 in patients with diabetes and COVID-19

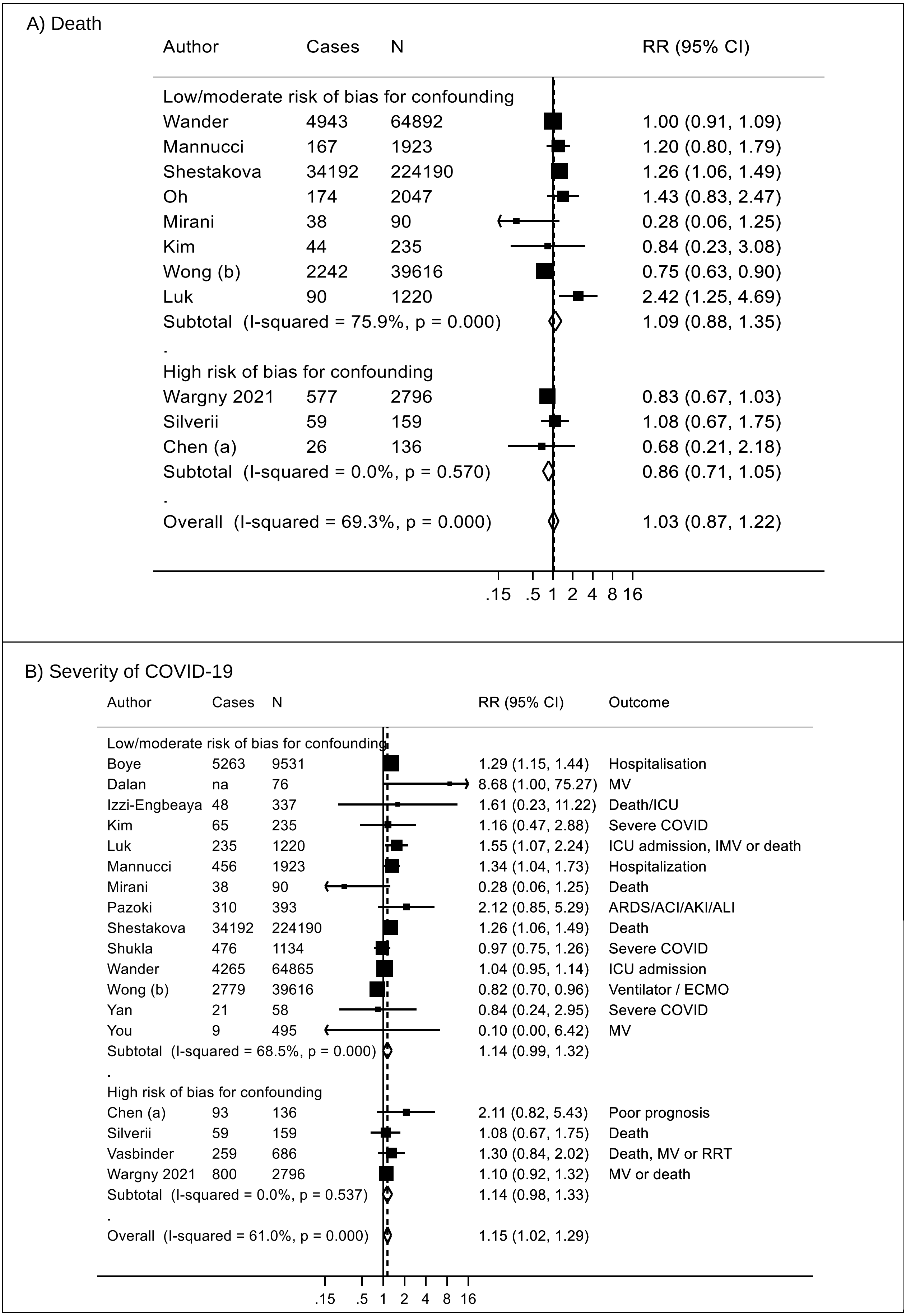

ESM Figure 30: Meta-analysis on use of **sulfonylurea/glinides/secretagogues** compared to non-use and A) death and B) severity of COVID-19 in patients with diabetes and COVID-19

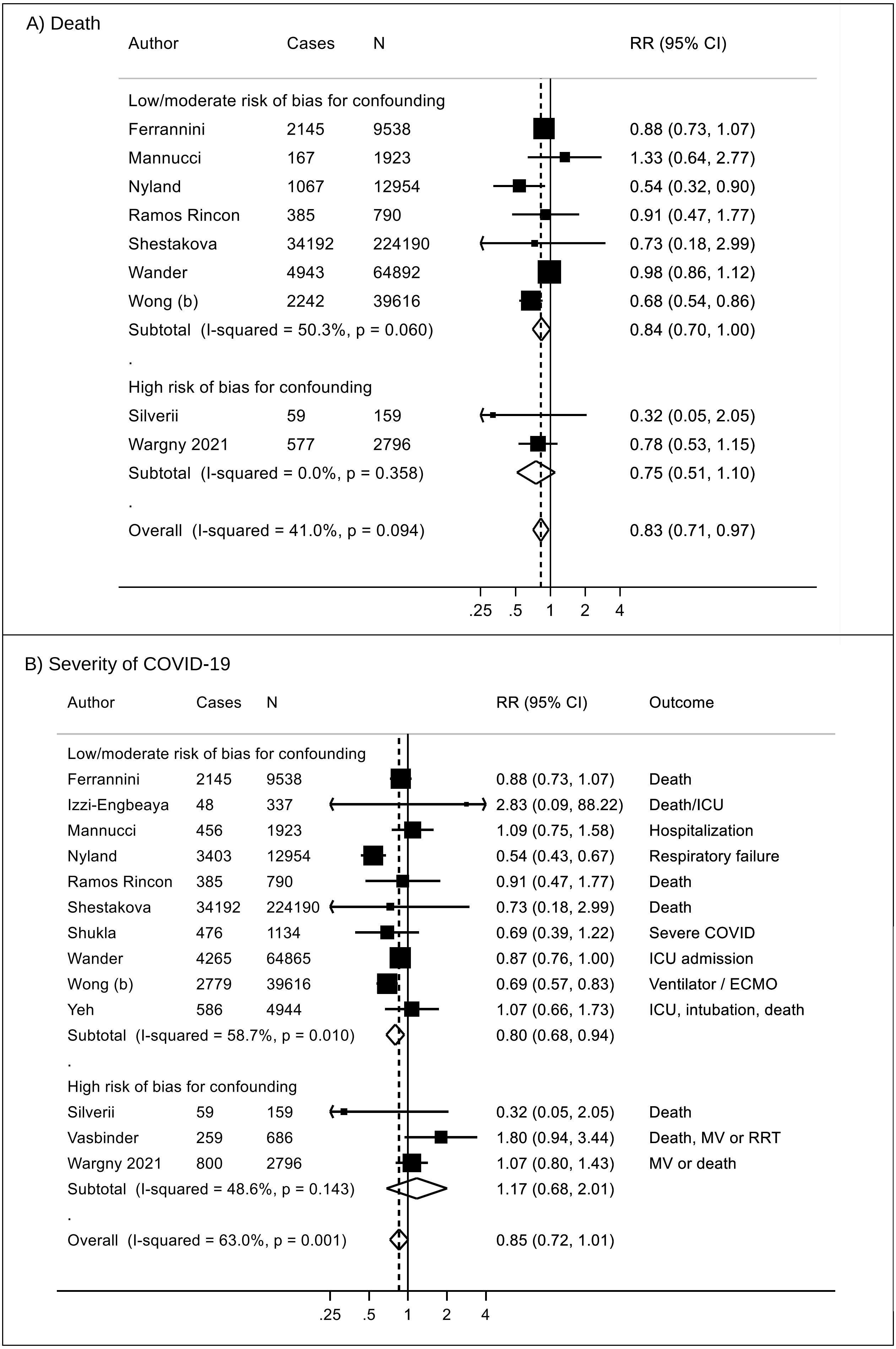

ESM Figure 31: Meta-analysis on use of **GLP 1-RA** compared to non-use and A) death and B) severity of COVID-19 in patients with diabetes and COVID-19

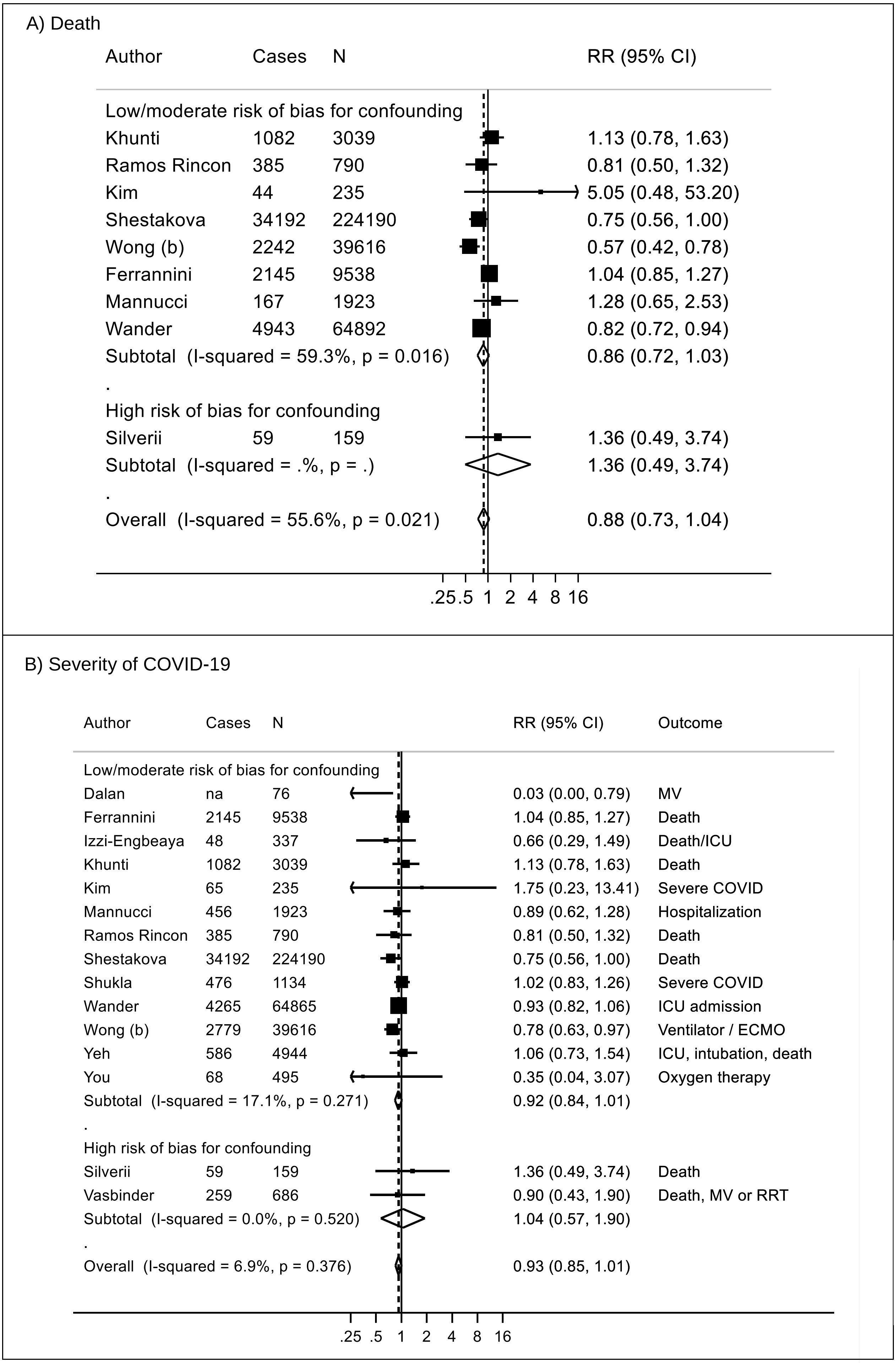

ESM Figure 32: Meta-analysis on use of **SGLT-2i** compared to non-use and A) death and B) severity of COVID-19 in patients with diabetes and COVID-19

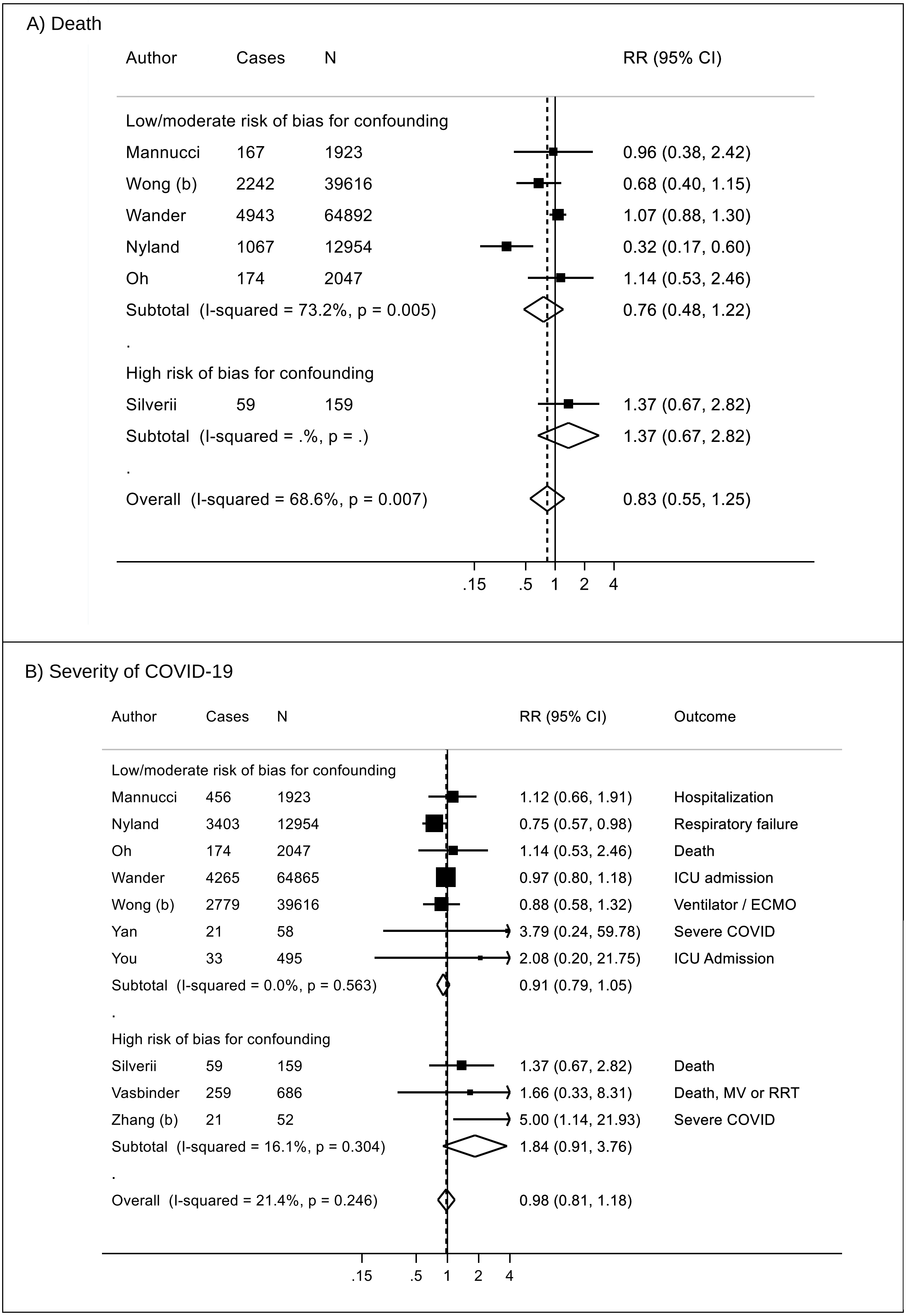

ESM Figure 33: Meta-analysis on use of **thiazolidinedione** compared to non-use and A) death and B) severity of COVID-19 in patients with diabetes and COVID-19

A) Death

No data

B) Severity of COVID-19

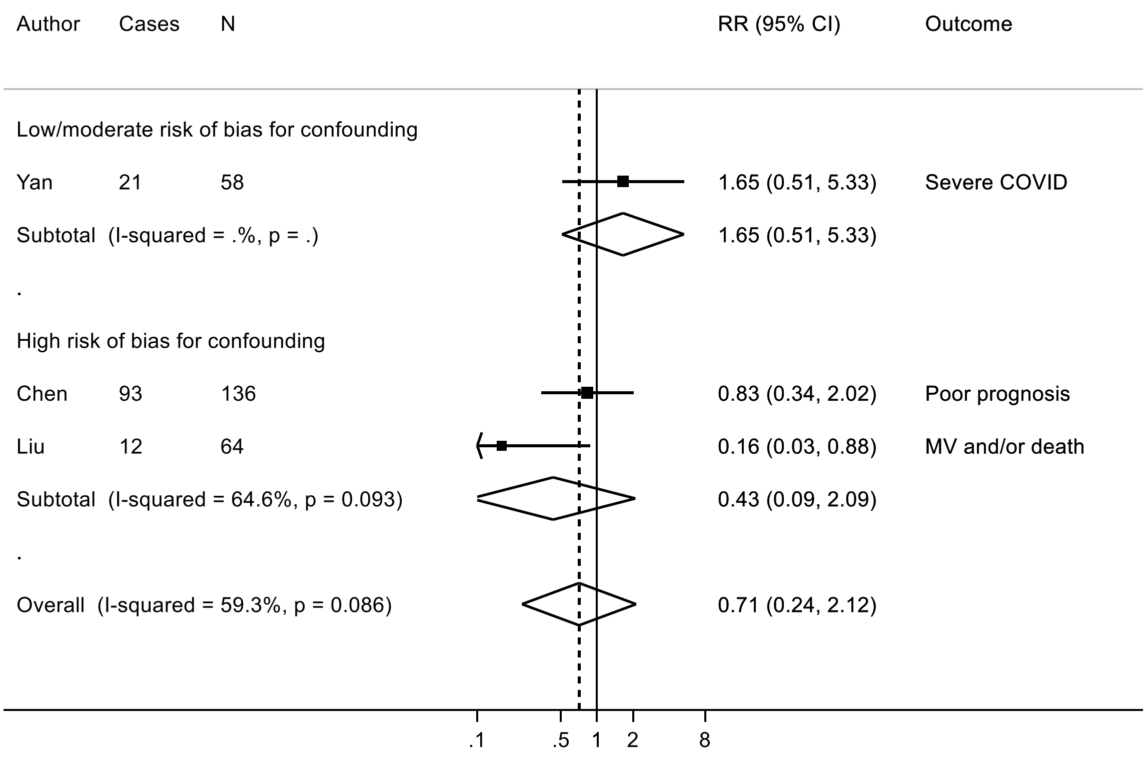

ESM Figure 34: Meta-analysis on use of **alpha-glucosidase inhibitors** compared to non-use and A) death and B) severity of COVID-19 in patients with diabetes and COVID-19

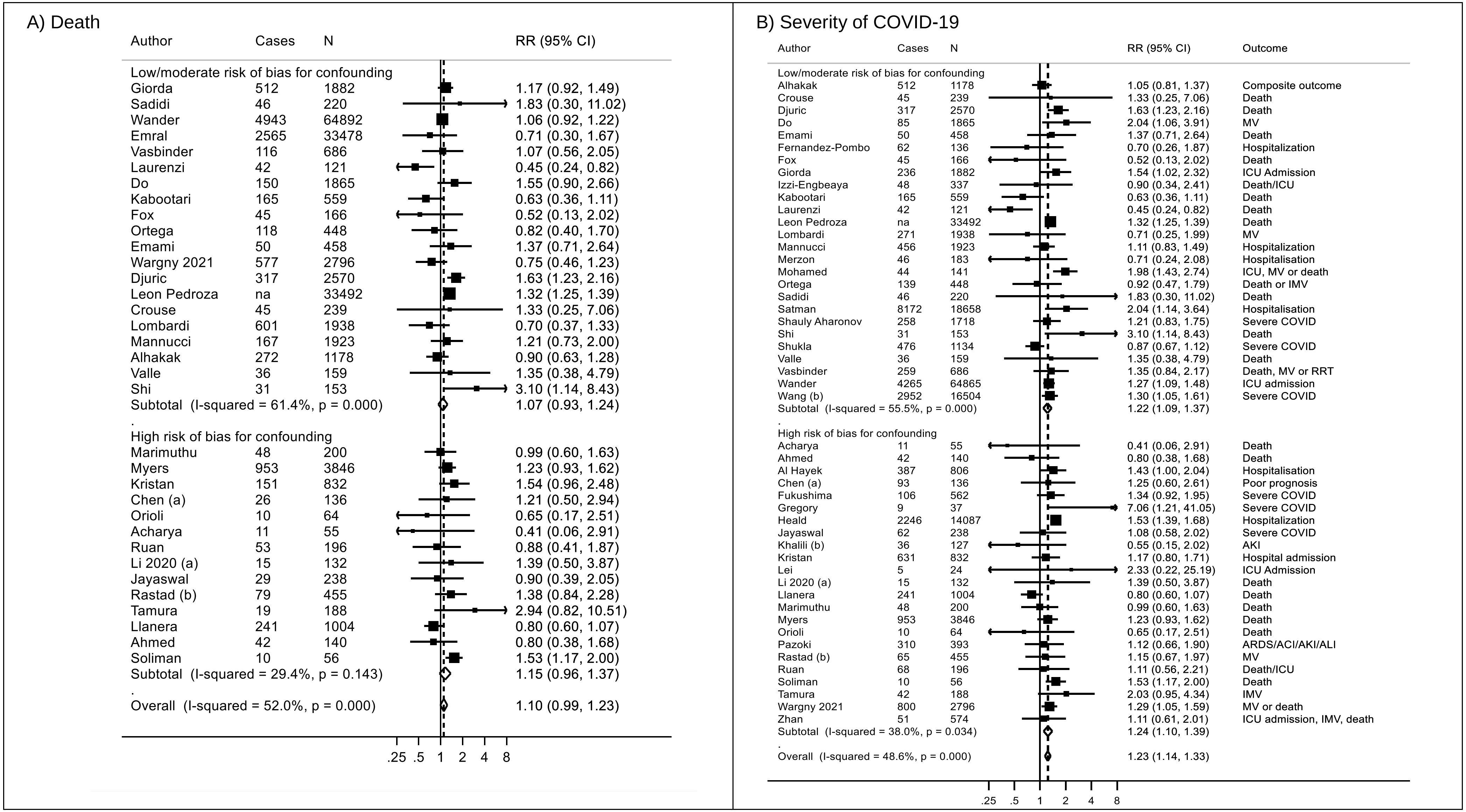

ESM Figure 35: Meta-analysis on **hypertension** compared to no hypertension and A) death and B) severity of COVID-19 in patients with diabetes and COVID-19

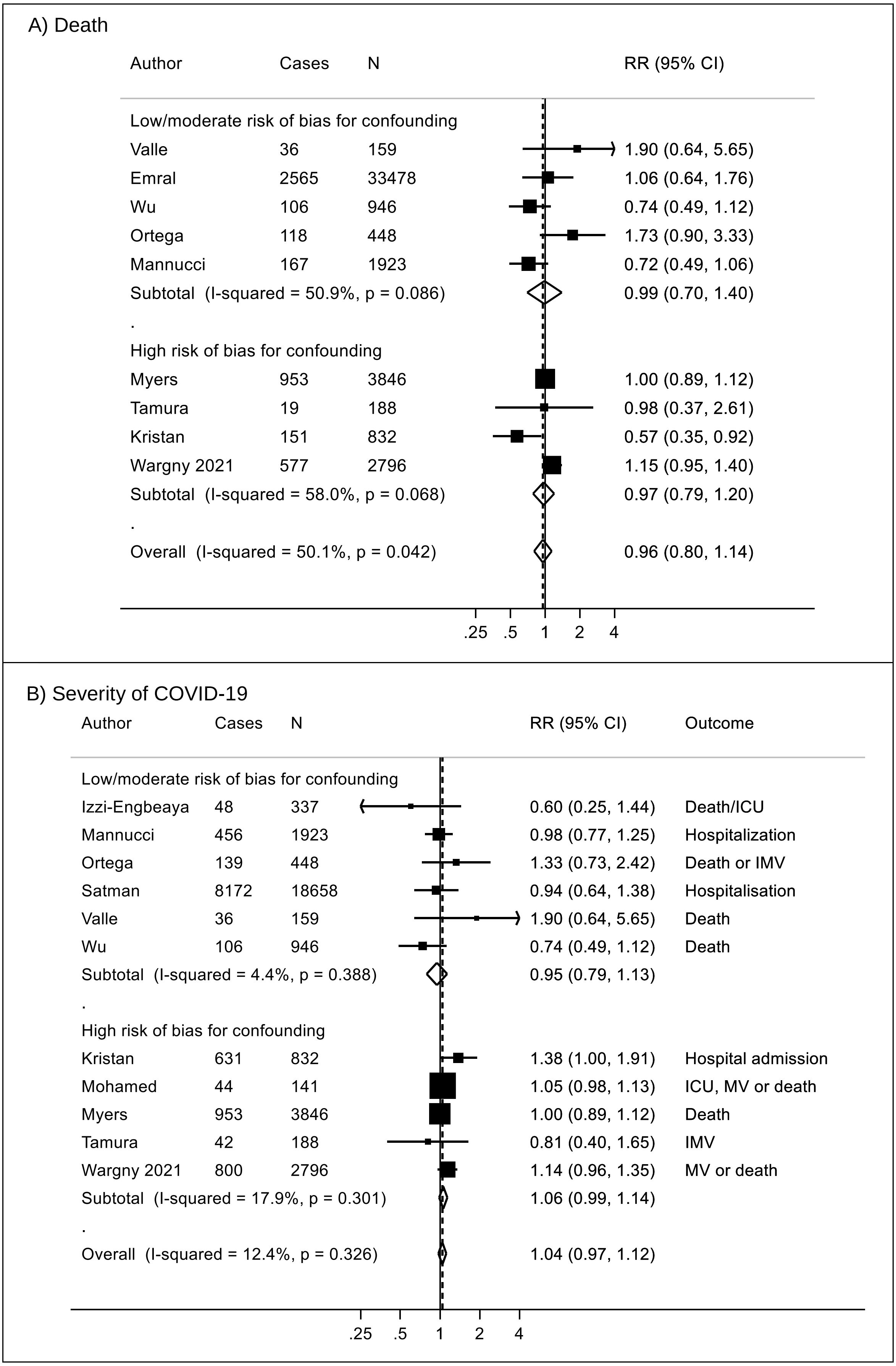

ESM Figure 36: Meta-analysis on **dyslipidaemia** compared to no dyslipidaemia and A) death and B) severity of COVID-19 in patients with diabetes and COVID-19

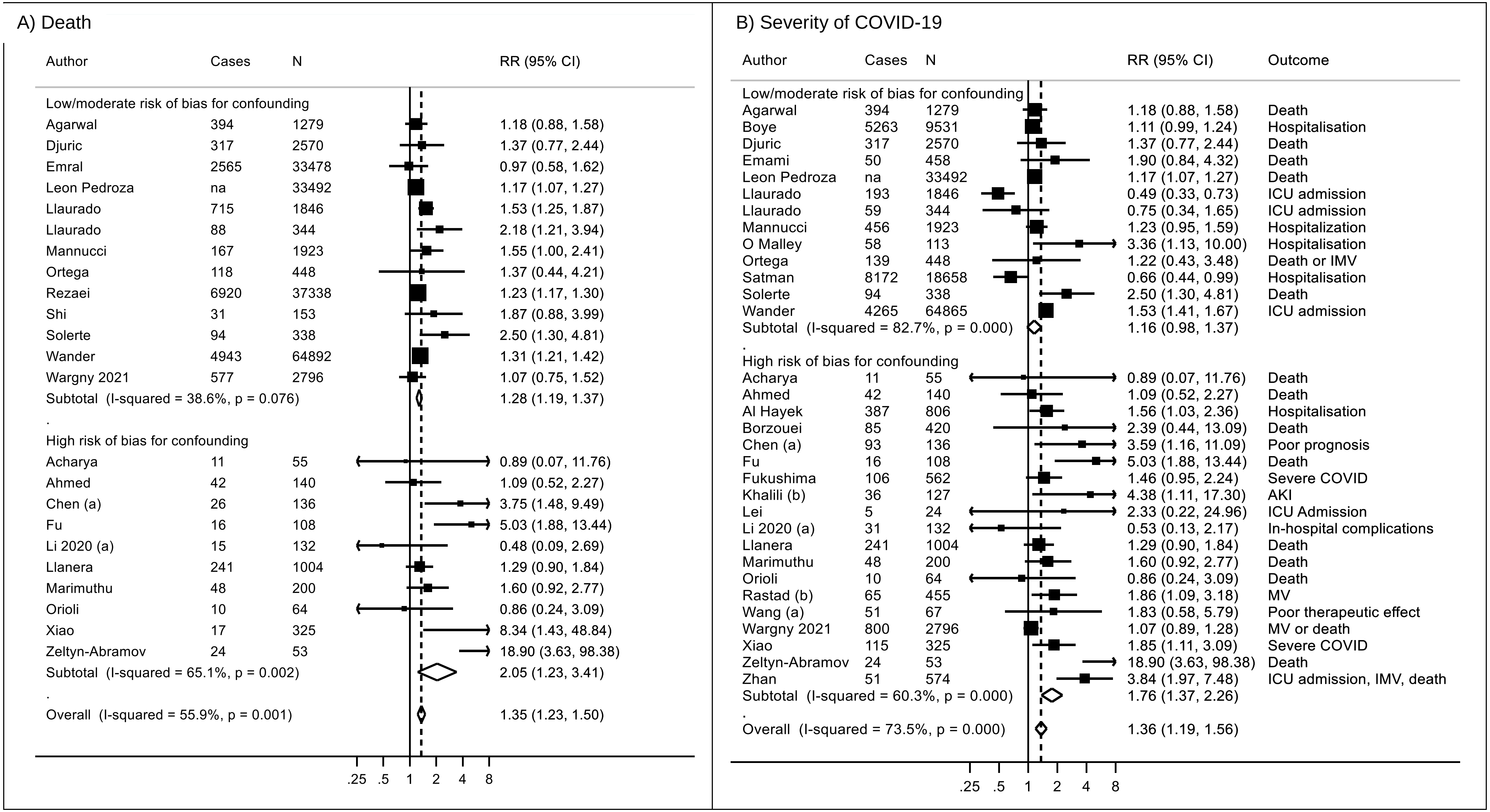

ESM Figure 37: Meta-analysis on pre-existing **cardiovascular disease (CVD)** compared to no CVD and A) death and B) severity of COVID-19 in patients with diabetes and COVID-19

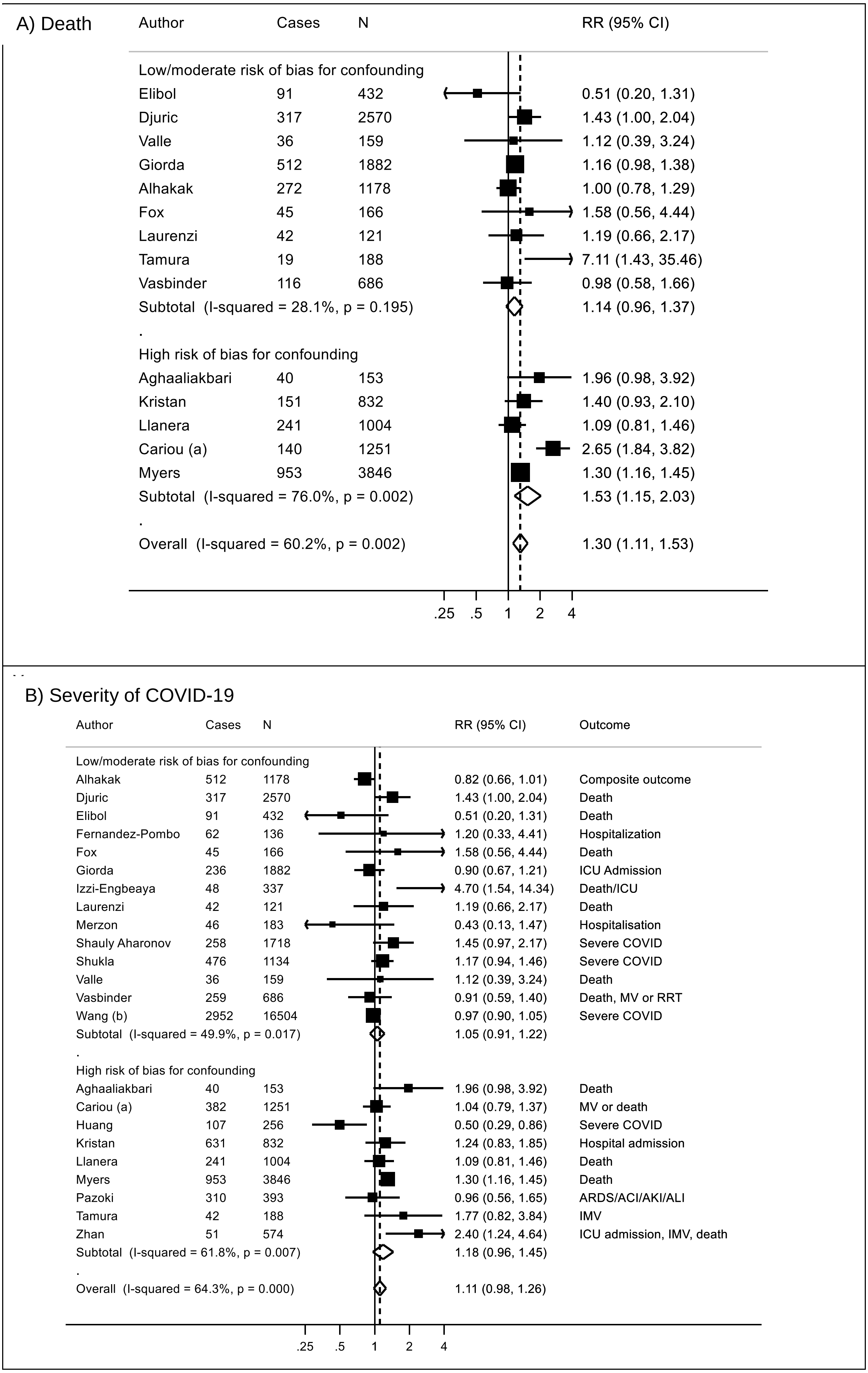

ESM Figure 38: Meta-analysis on pre-existing **coronary artery disease (CAD)** compared to no CAD and A) death and B) severity of COVID-19 in patients with diabetes and COVID-19

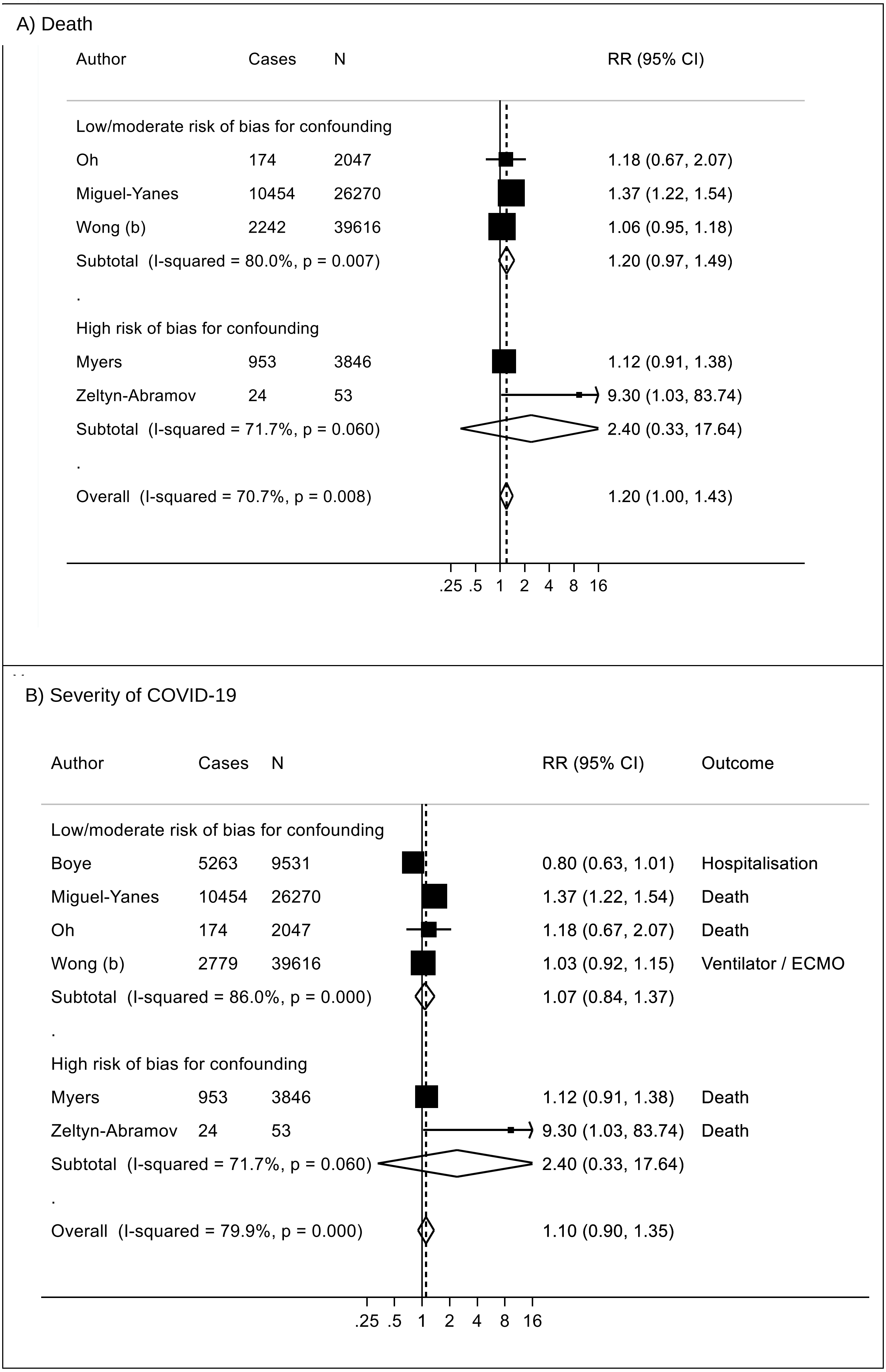

ESM Figure 39: Meta-analysis on pre-existing **myocardial infarction (MI)** compared to no MI and A) death and B) severity of COVID-19 in patients with diabetes and COVID-19

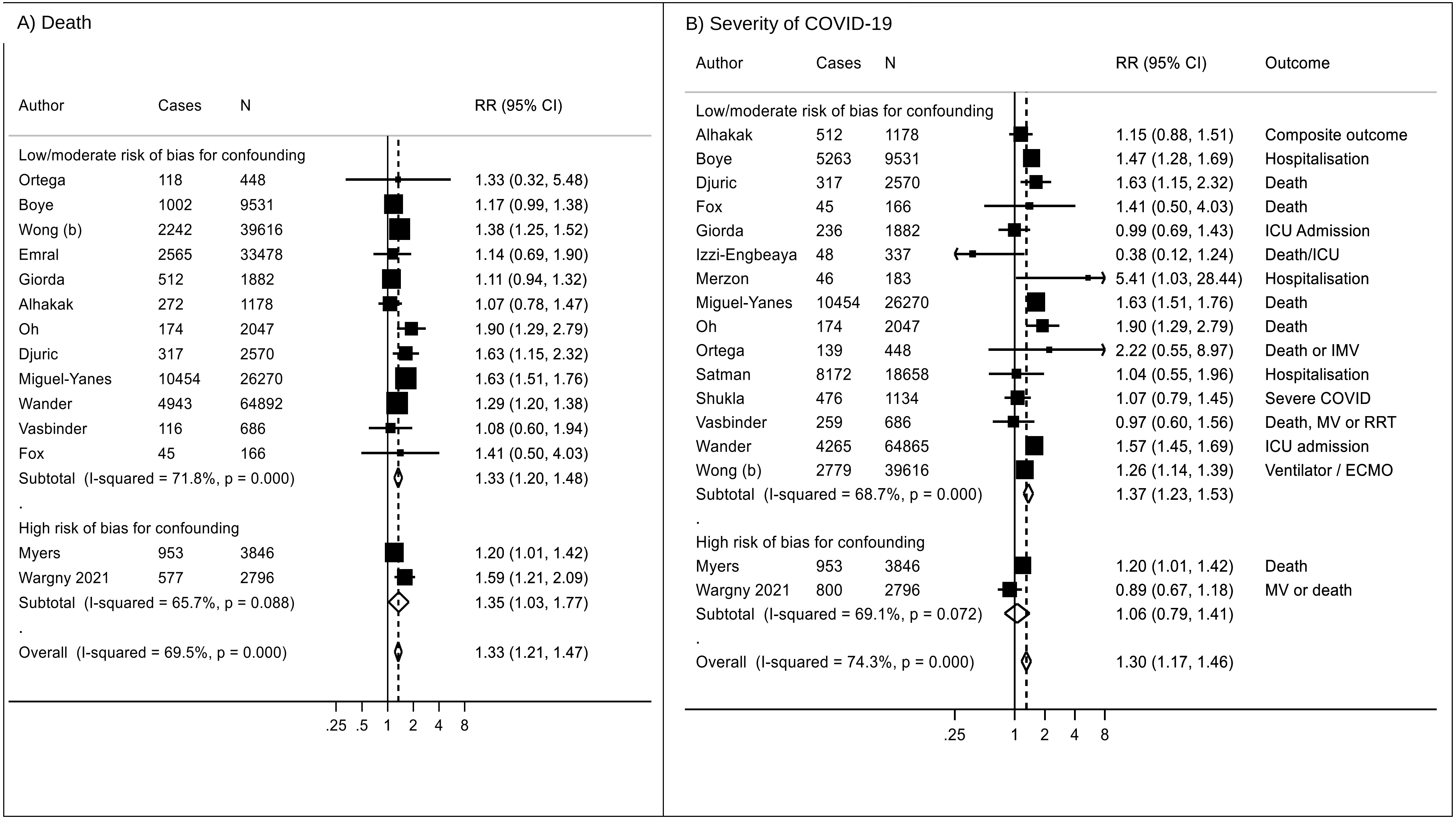

ESM Figure 40: Meta-analysis on pre-existing **heart failure (HF)** compared to no HF and A) death and B) severity of COVID-19 in patients with diabetes and COVID-19

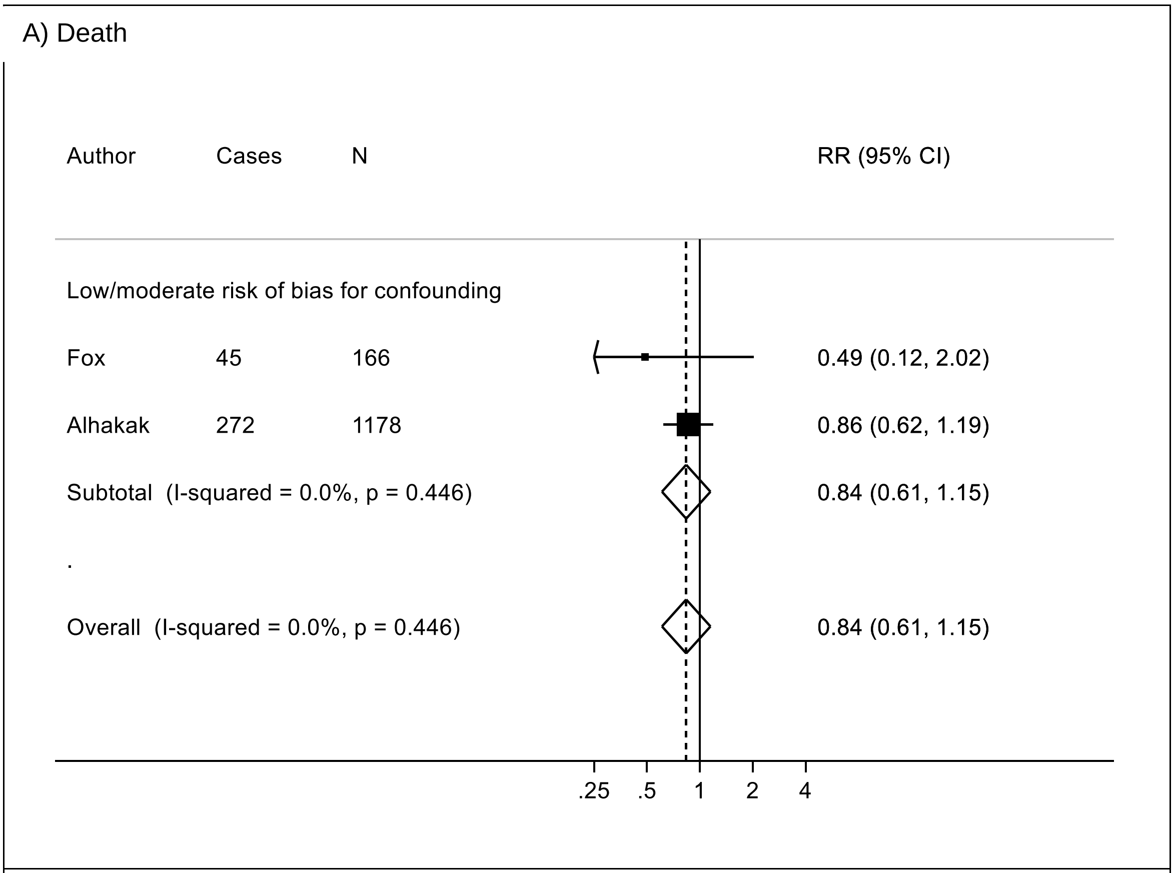

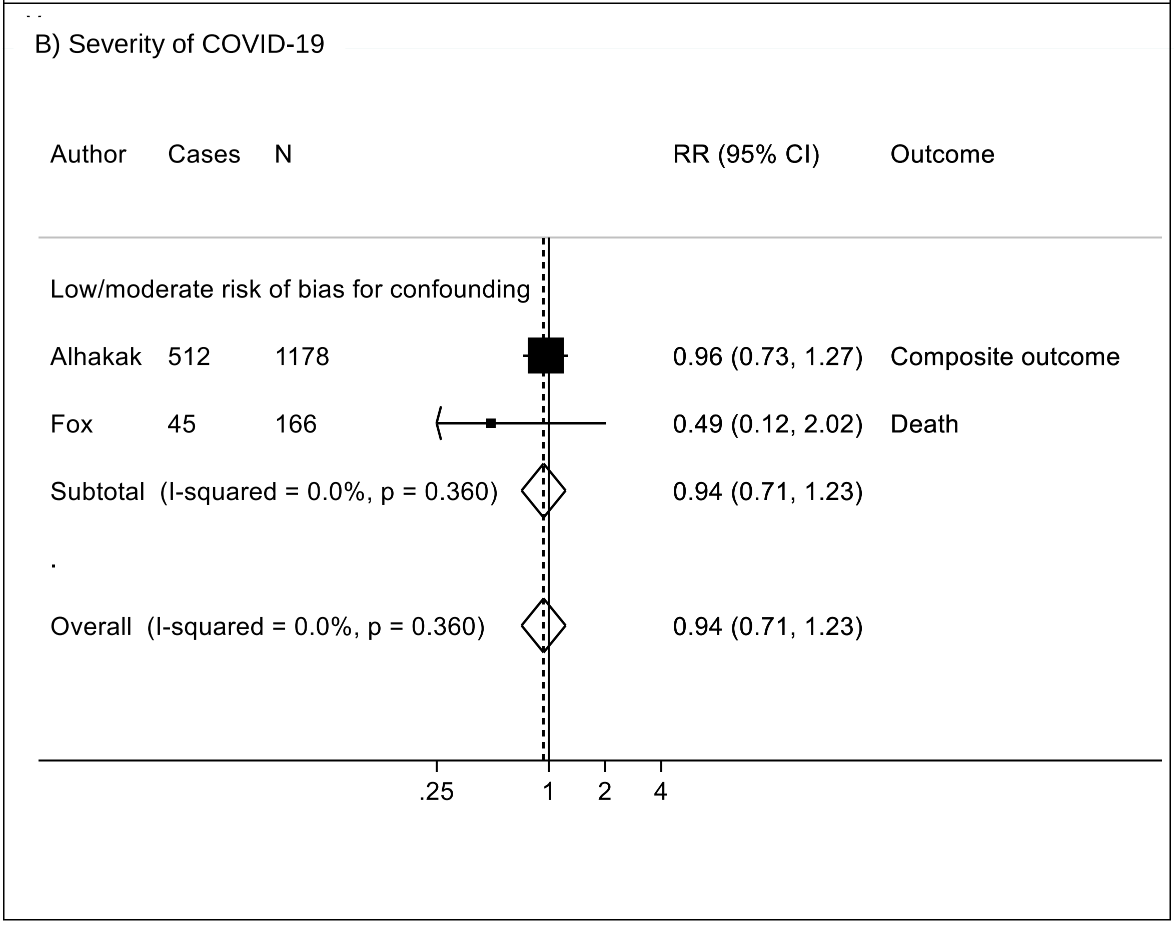

ESM Figure 41: Meta-analysis on pre-existing **atrial fibrillation (AF)** compared to no AF and A) death and B) severity of COVID-19 in patients with diabetes and COVID-19

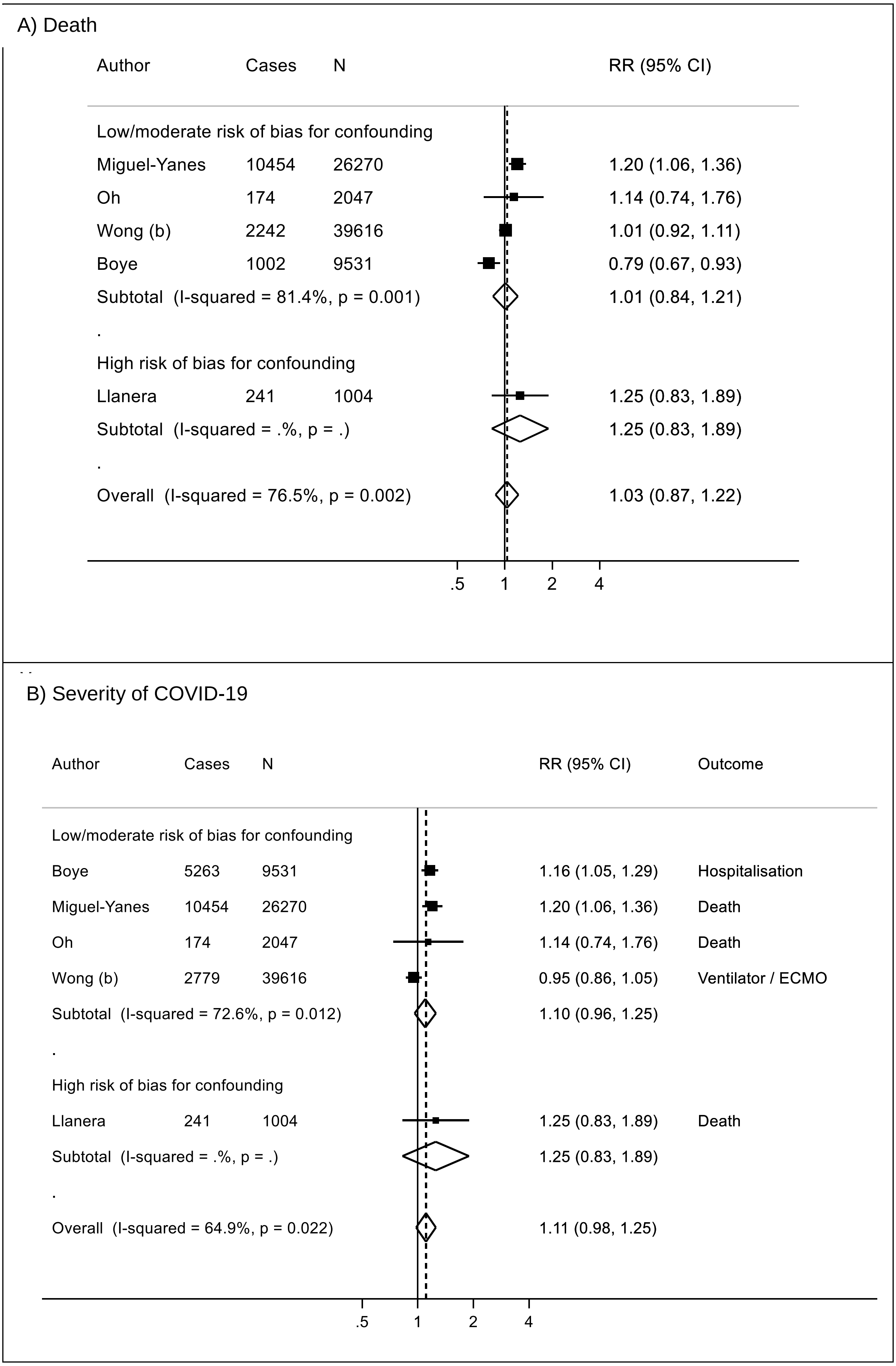

ESM Figure 42: Meta-analysis on pre-existing **peripheral vascular disease (PVD)** compared to no PVD and A) death and B) severity of COVID-19 in patients with diabetes and COVID-19

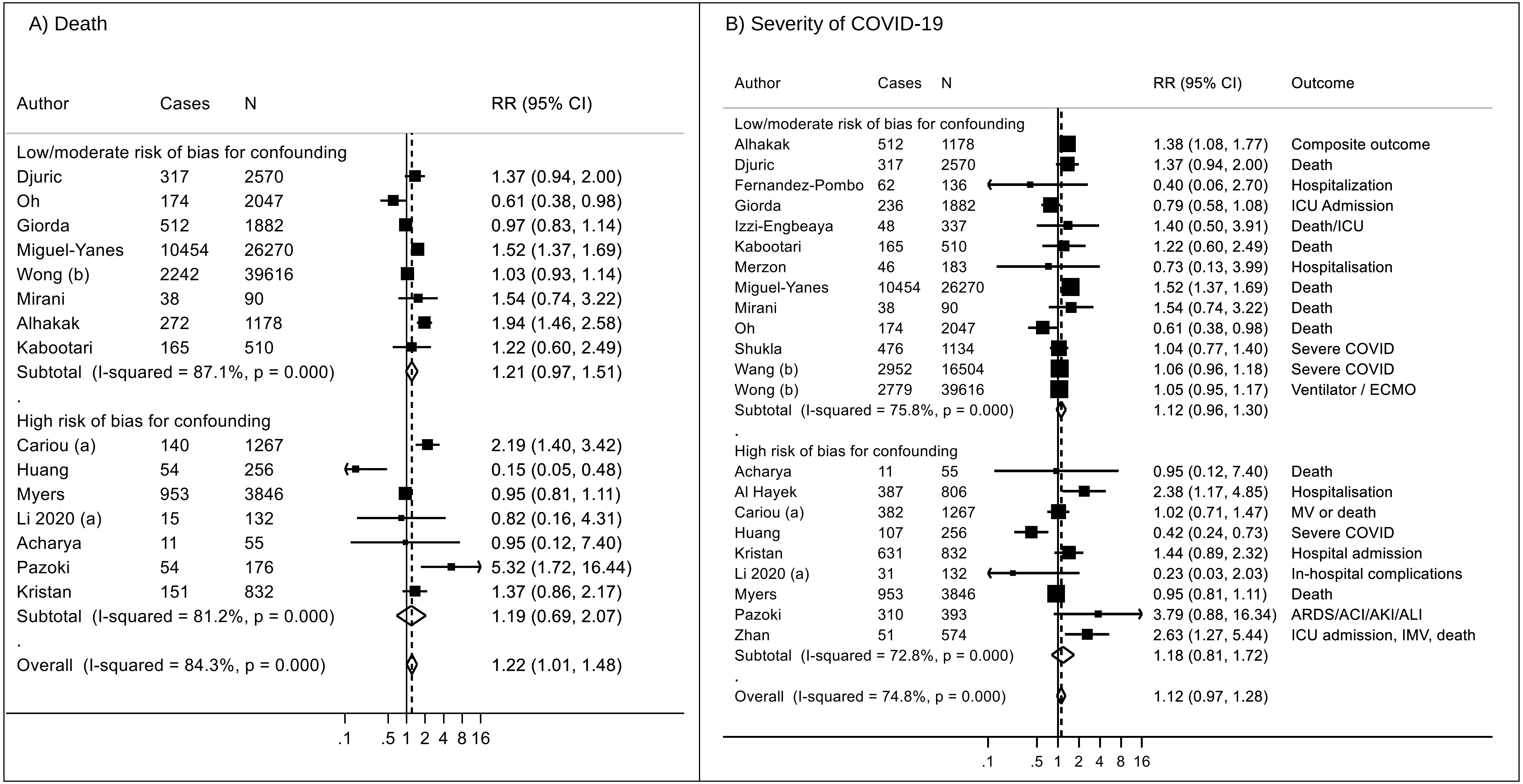

ESM Figure 43: Meta-analysis on pre-existing **cerebrovascular disease** compared to no cerebrovascular disease and A) death and B) severity of COVID-19 in patients with diabetes and COVID-19

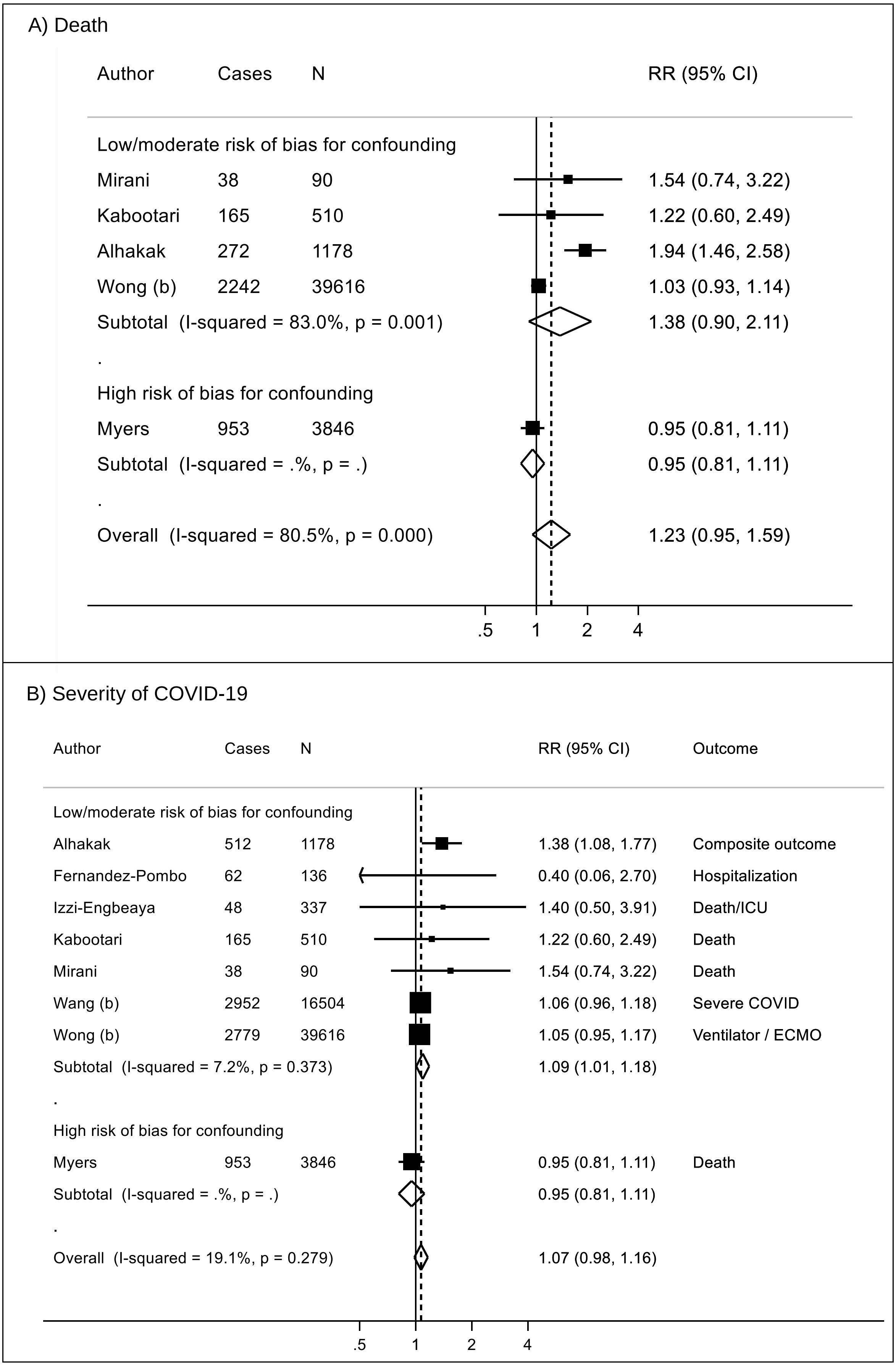

ESM Figure 44: Meta-analysis on pre-existing **stroke** compared to no stroke and A) death and B) severity of COVID-19 in patients with diabetes and COVID-19

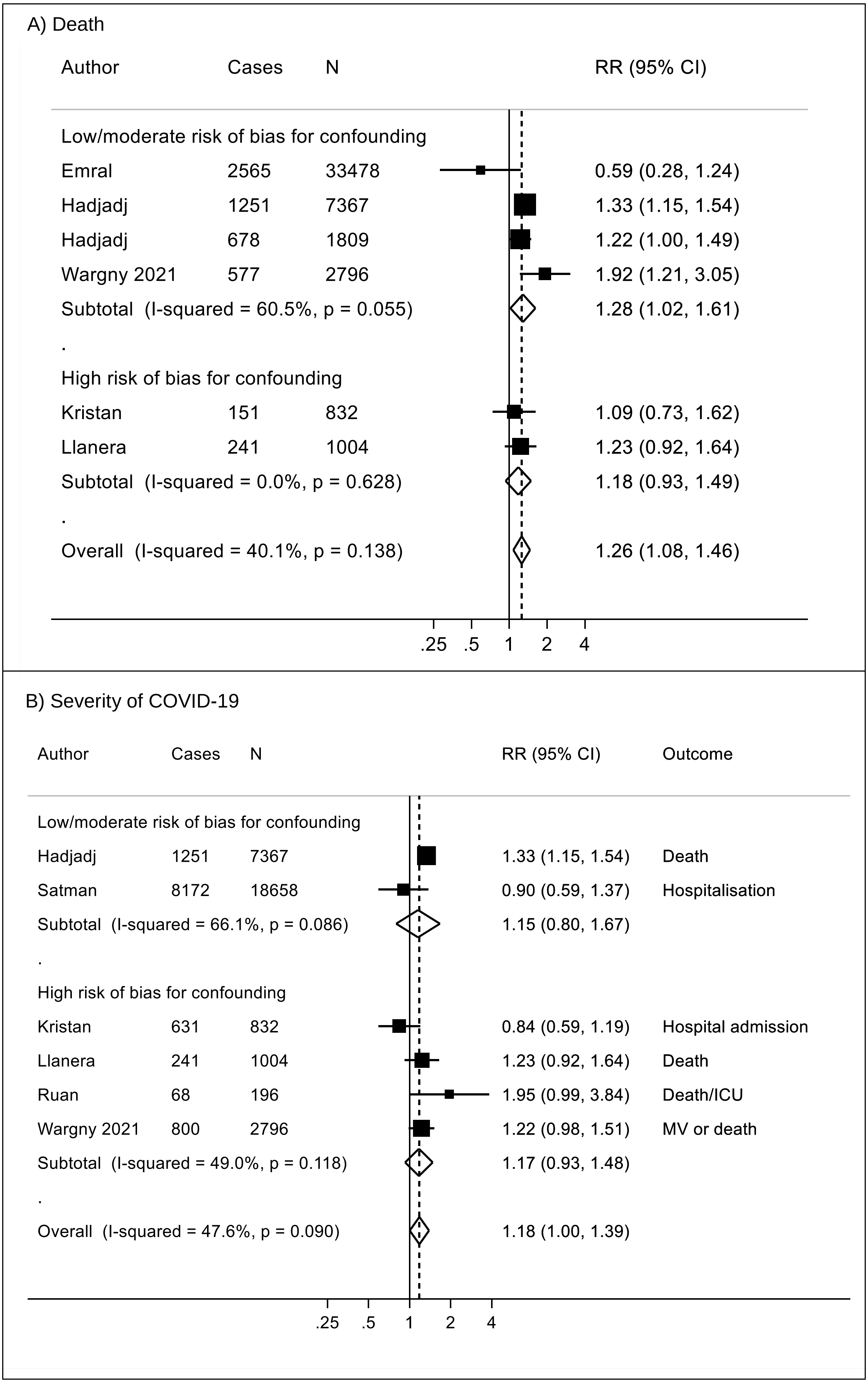

ESM Figure 45: Meta-analysis on pre-existing **microvascular diseases (MVD)** compared to no MVD and A) death and B) severity of COVID-19 in patients with diabetes and COVID-19

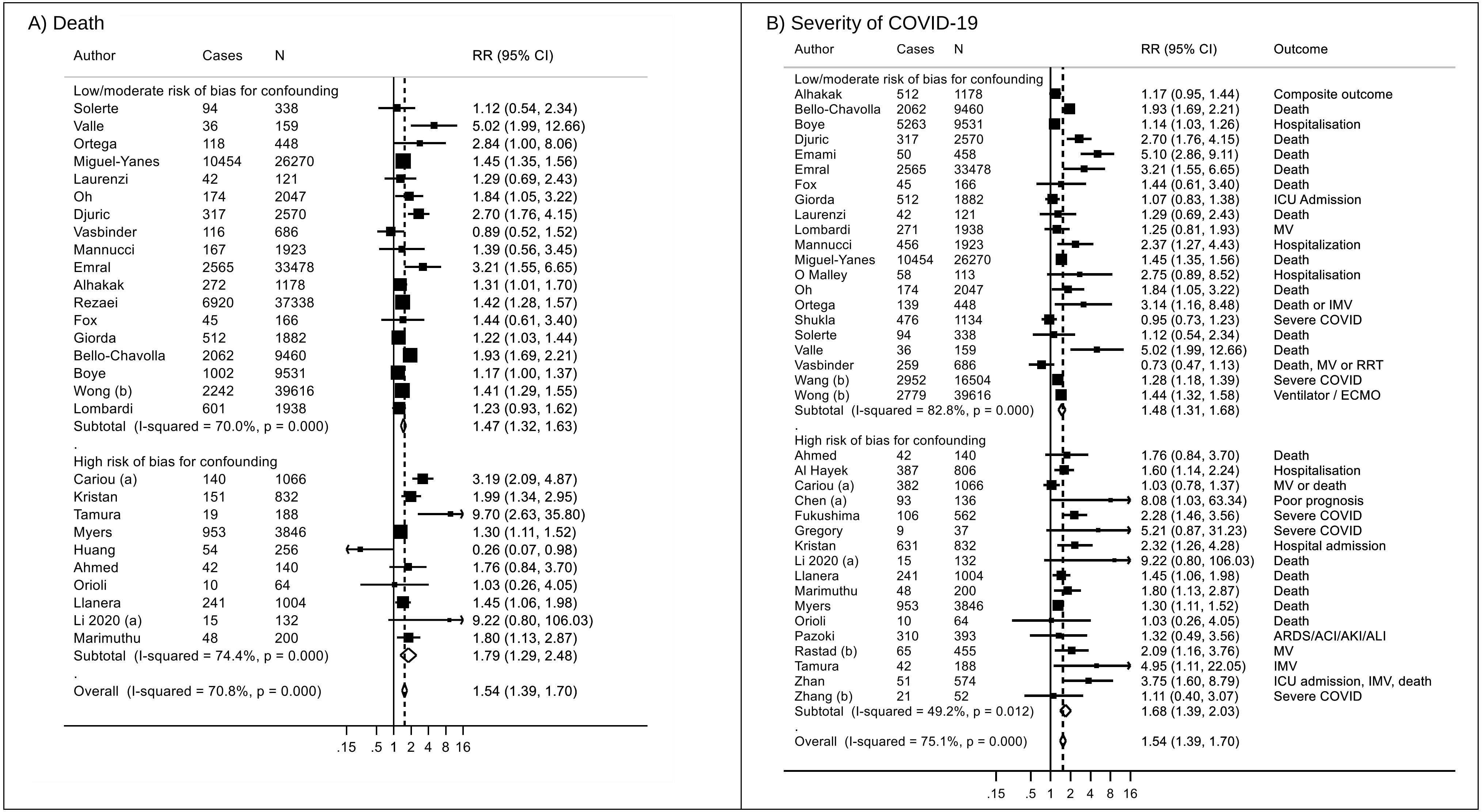

ESM Figure 46: Meta-analysis on pre-existing **chronic kidney disease (CKD)** compared to no CKD and A) death and B) severity of COVID-19 in patients with diabetes and COVID-19

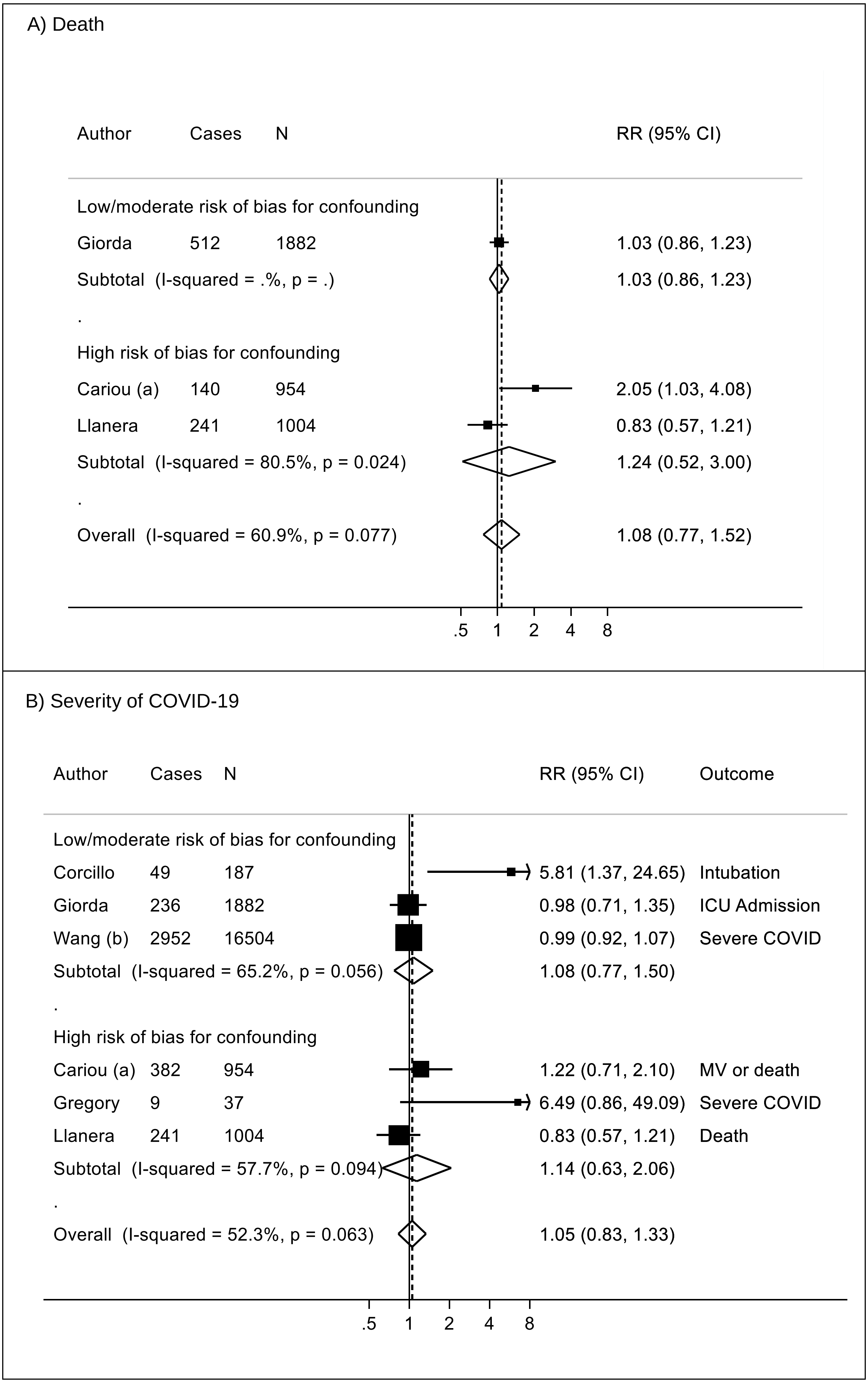

ESM Figure 47: Meta-analysis on pre-existing **retinopathy** compared to no retinopathy and A) death and B) severity of COVID-19 in patients with diabetes and COVID-19

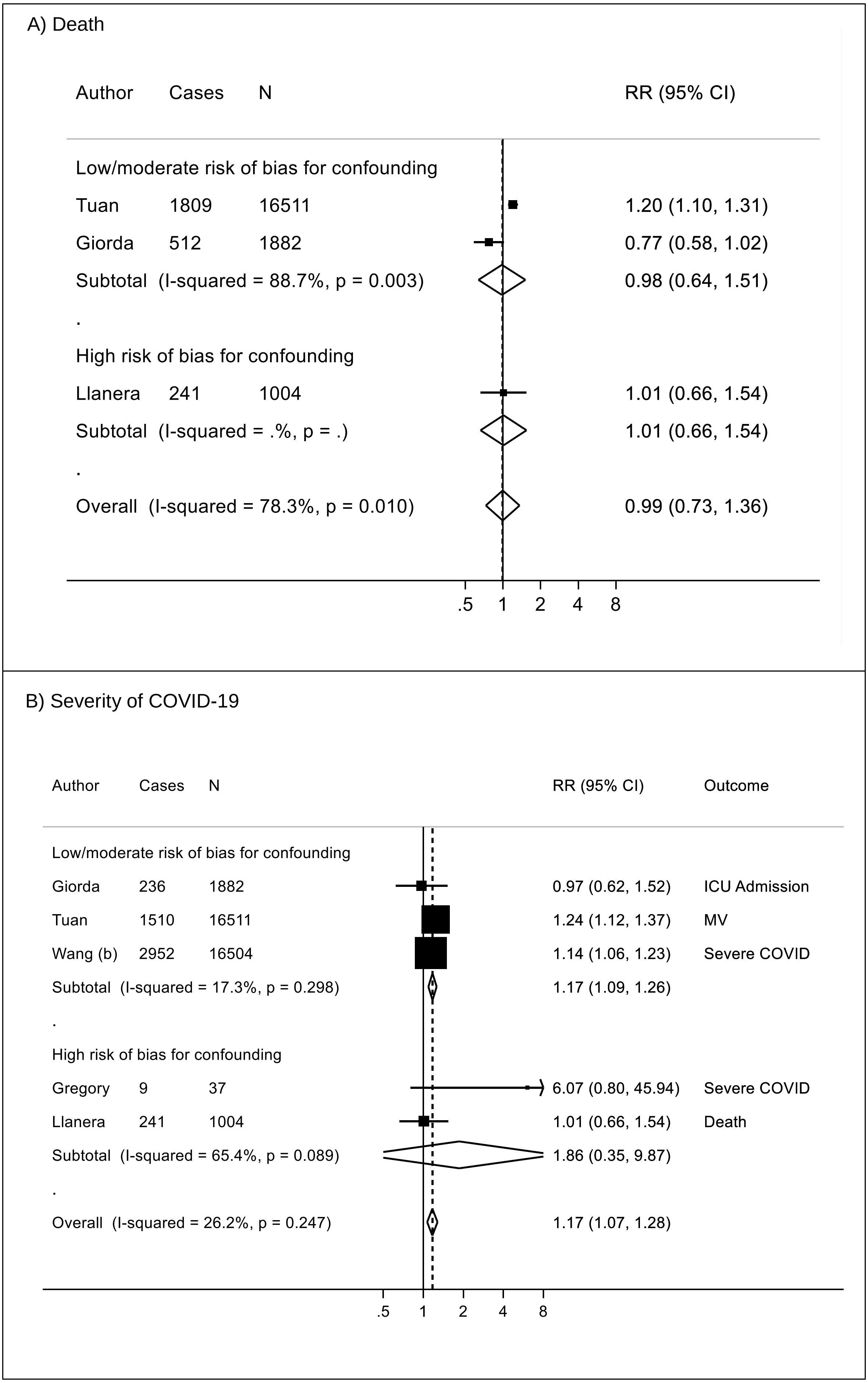

ESM Figure 48: Meta-analysis on pre-existing **neuropathy** compared to no neuropathy and A) death and B) severity of COVID-19 in patients with diabetes and COVID-19

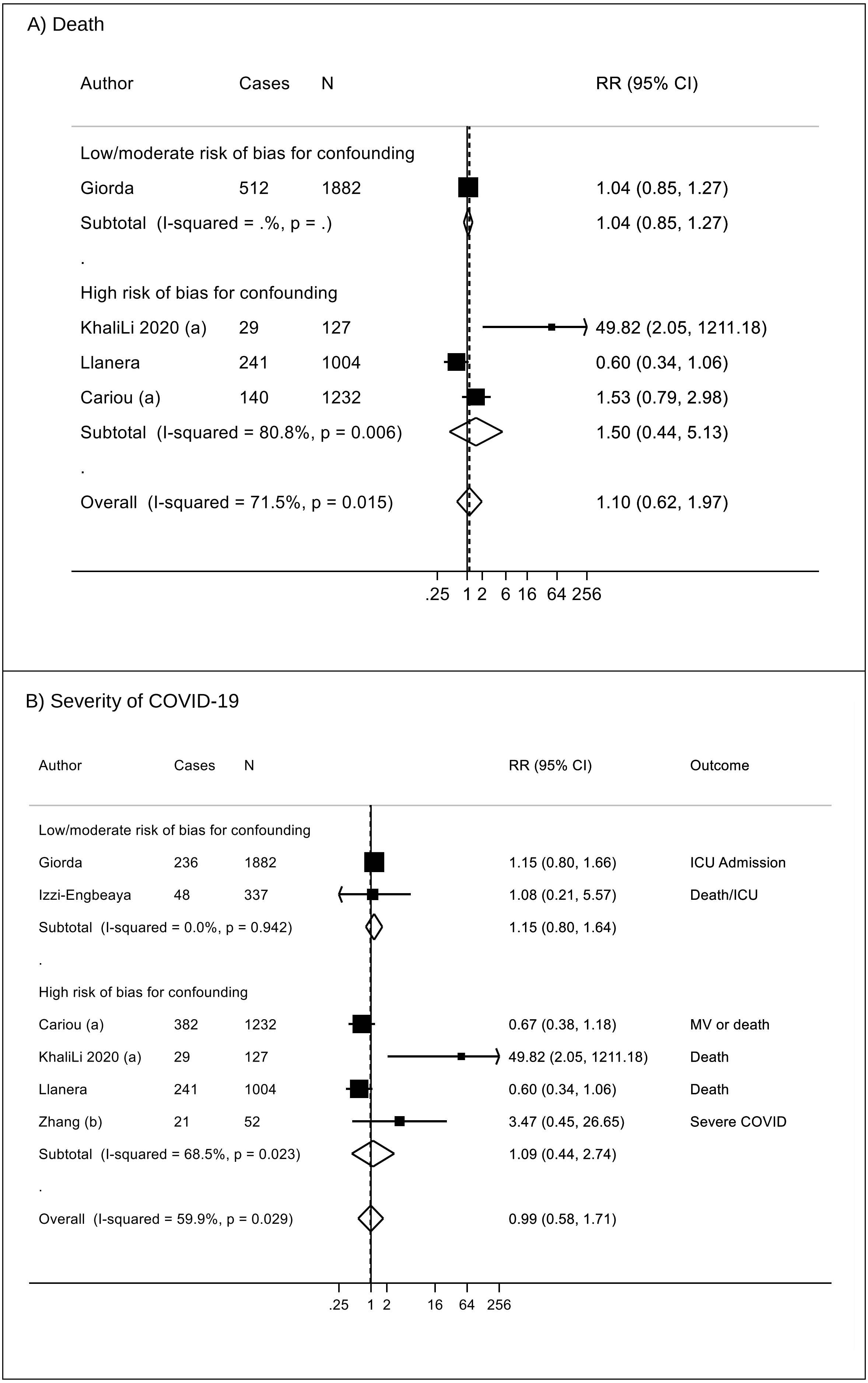

ESM Figure 49: Meta-analysis on pre-existing **diabetic foot** compared to no diabetic foot and A) death and B) severity of COVID-19 in patients with diabetes and COVID-19

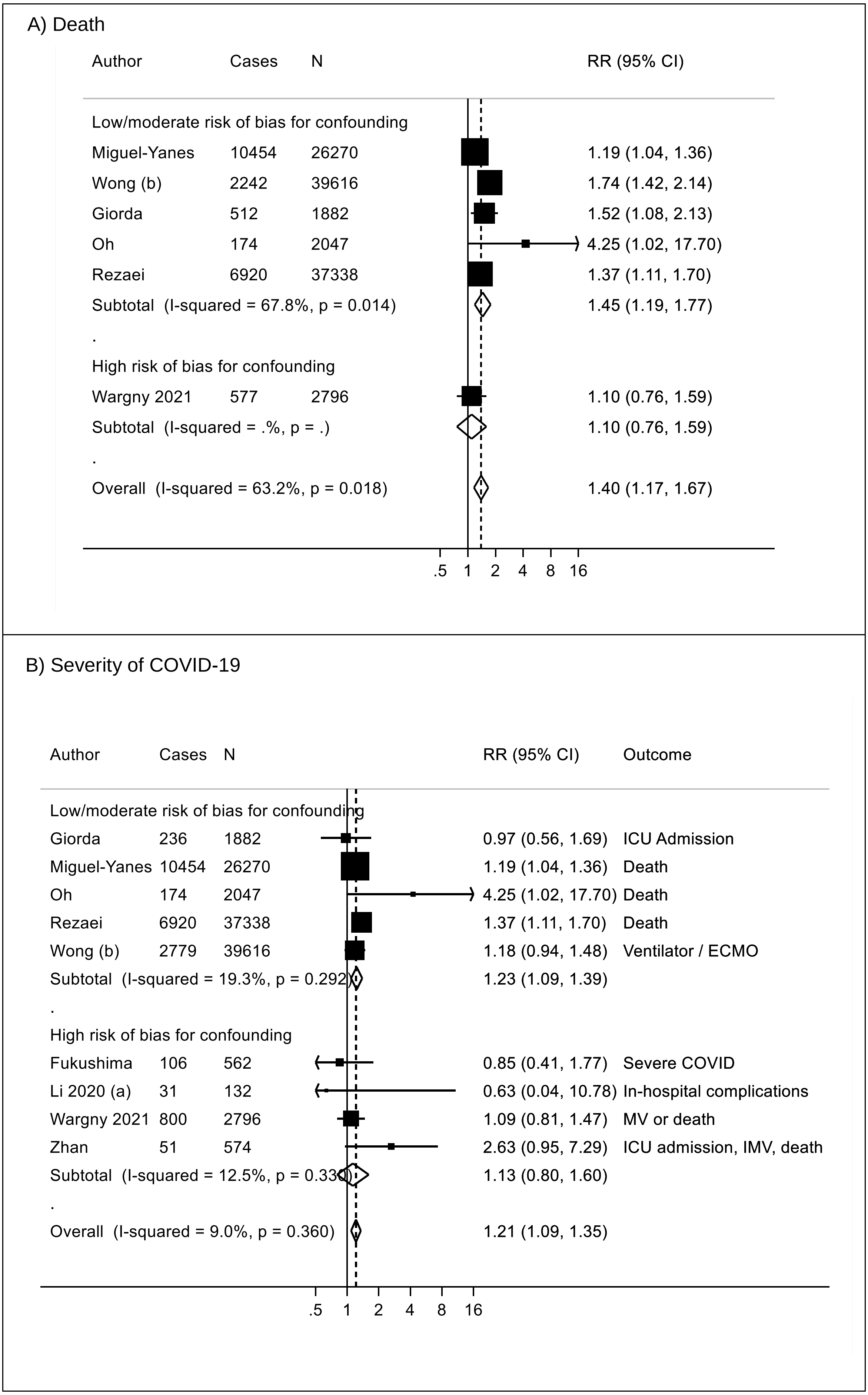

ESM Figure 50: Meta-analysis on pre-existing **liver disease** compared to no liver disease and A) death and B) severity of COVID-19 in patients with diabetes and COVID-19

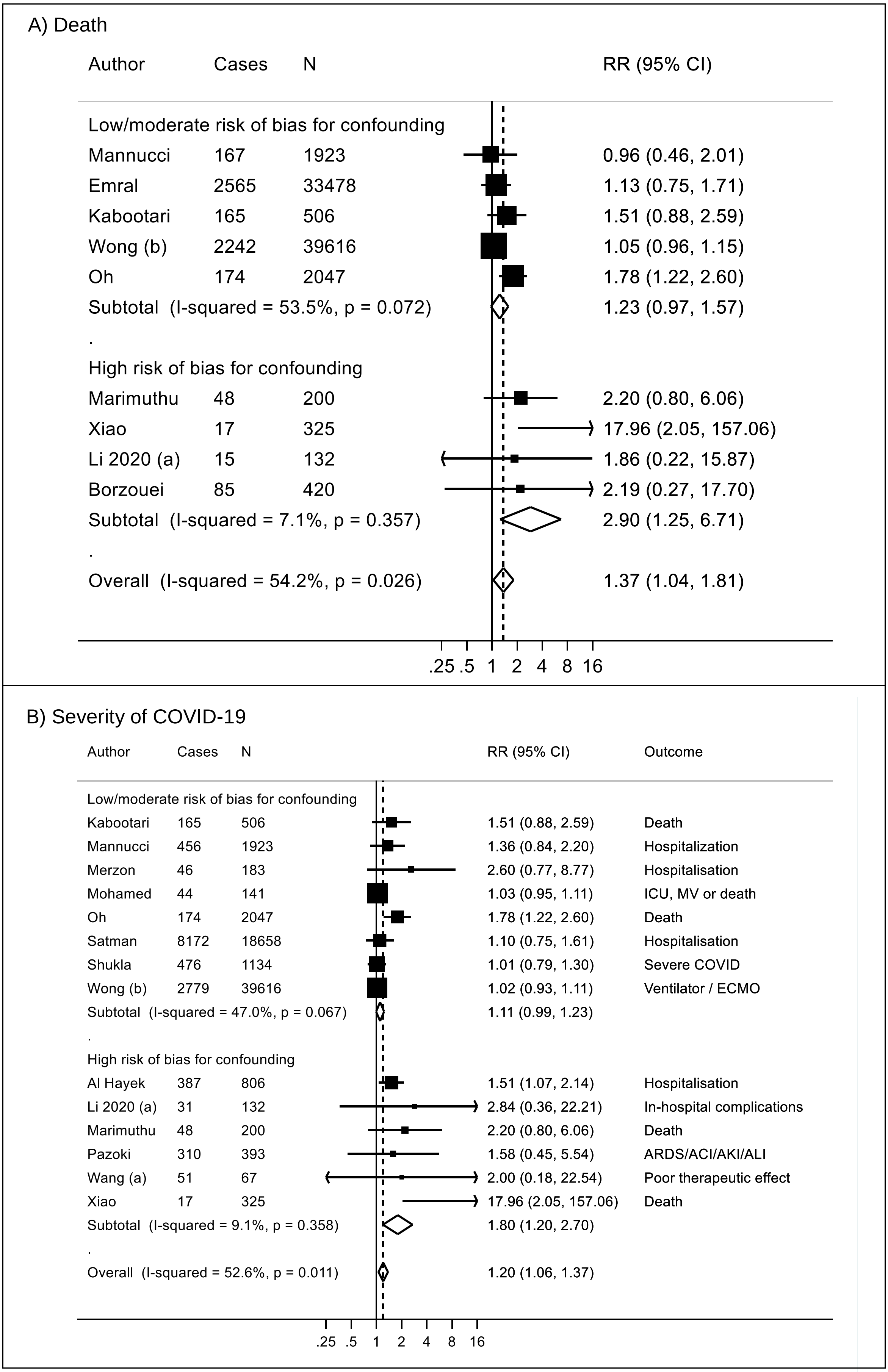

ESM Figure 51: Meta-analysis on pre-existing **chronic pulmonary disease** (not specified) compared to no chronic pulmonary disease and A) death and B) severity of COVID-19 in patients with diabetes and COVID-19

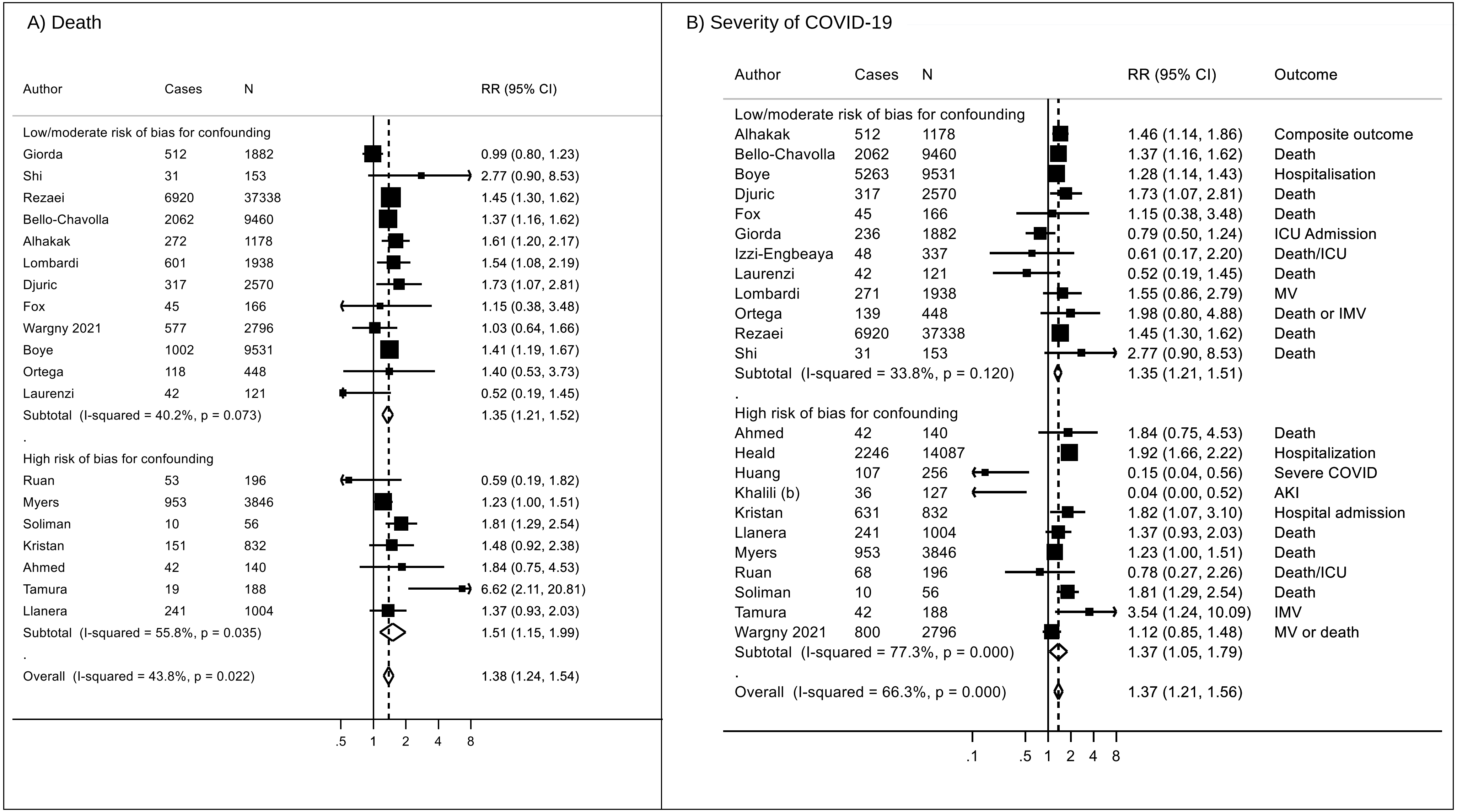

ESM Figure 52: Meta-analysis on pre-existing **chronic obstructive pulmonary disease (COPD)** compared to no COPD and A) death and B) severity of COVID-19 in patients with diabetes and COVID-19

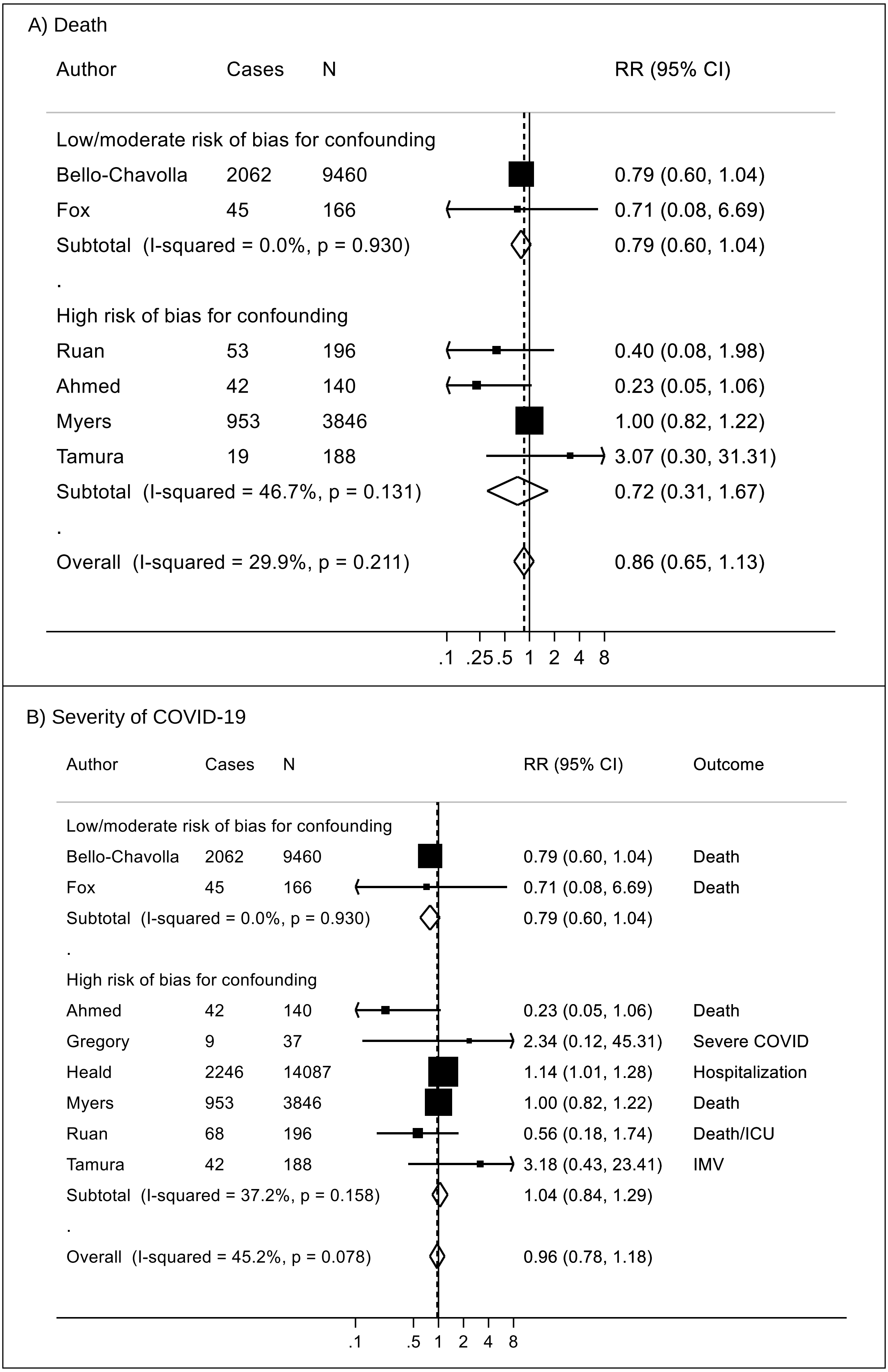

ESM Figure 53: Meta-analysis on pre-existing **asthma** compared to no asthma and A) death and B) severity of COVID-19 in patients with diabetes and COVID-19

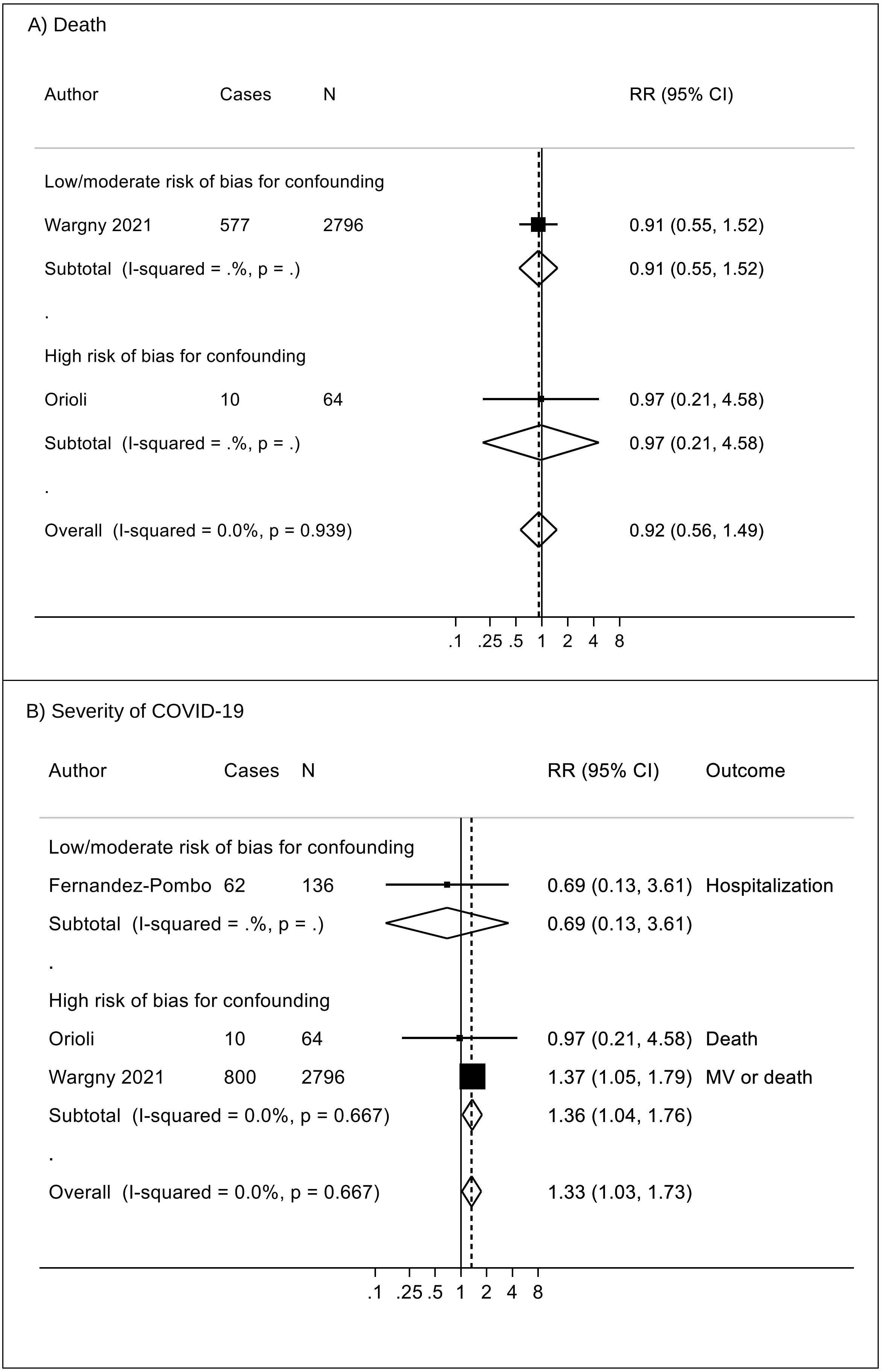

ESM Figure 54: Meta-analysis on pre-existing **obstructive sleep apnoea (OSA)** compared to no OSA and A) death and B) severity of COVID-19 in patients with diabetes and COVID-19

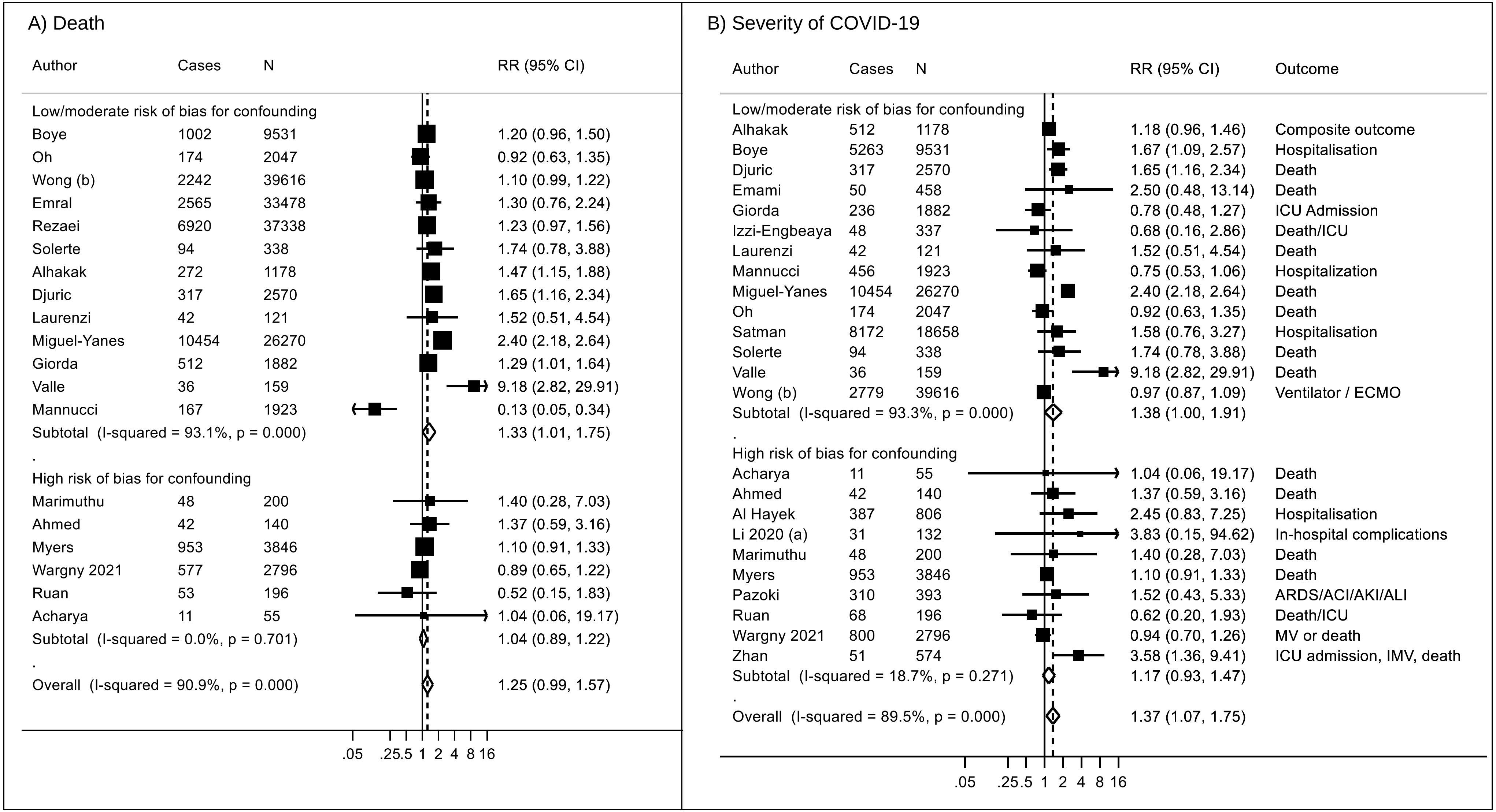

ESM Figure 55: Meta-analysis on pre-existing **cancer** compared to no cancer and A) death and B) severity of COVID-19 in patients with diabetes and COVID-19

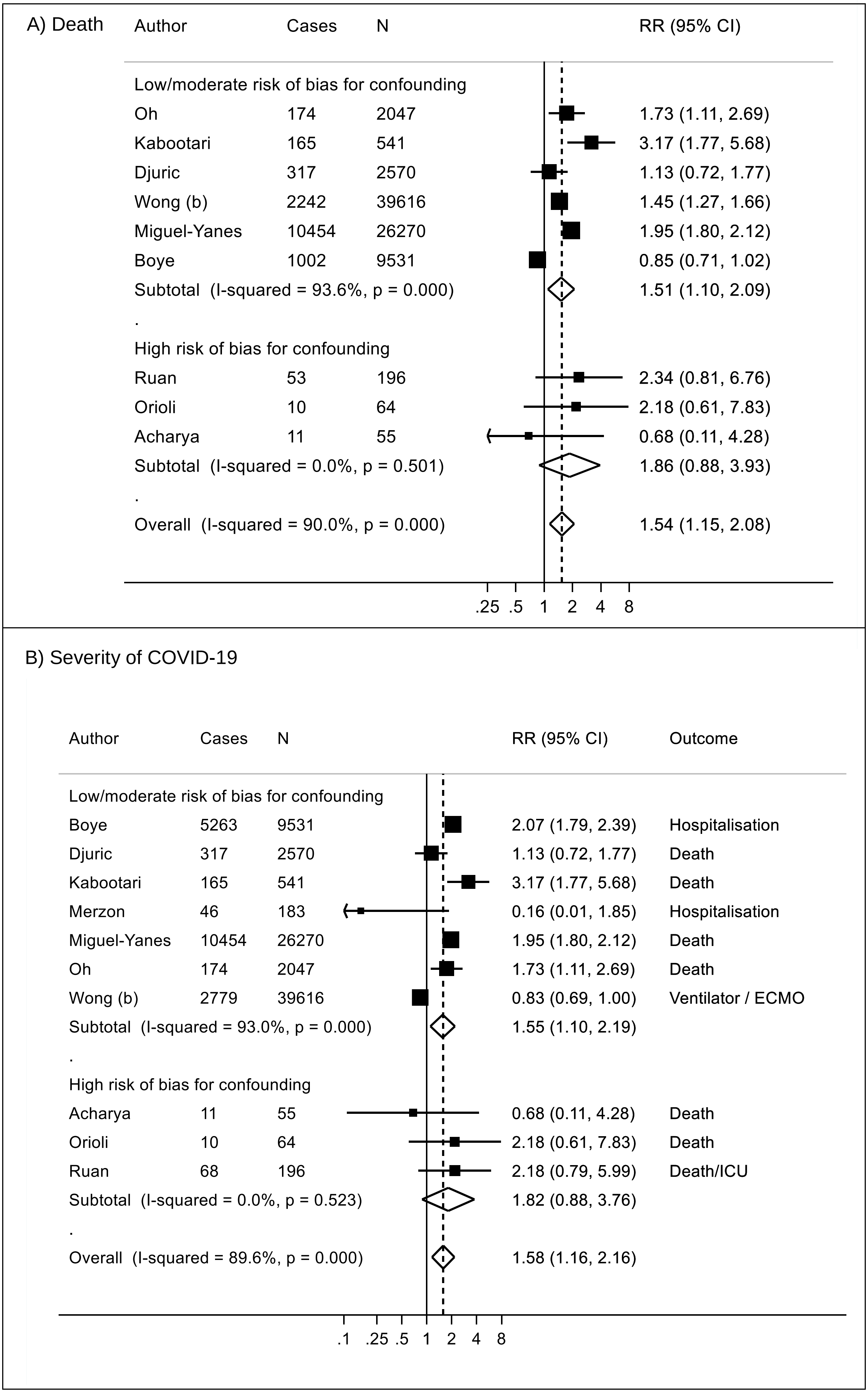

ESM Figure 56: Meta-analysis on pre-existing **dementia/cognitive impairment** compared to no dementia and A) death and B) severity of COVID-19 in patients with diabetes and COVID-19

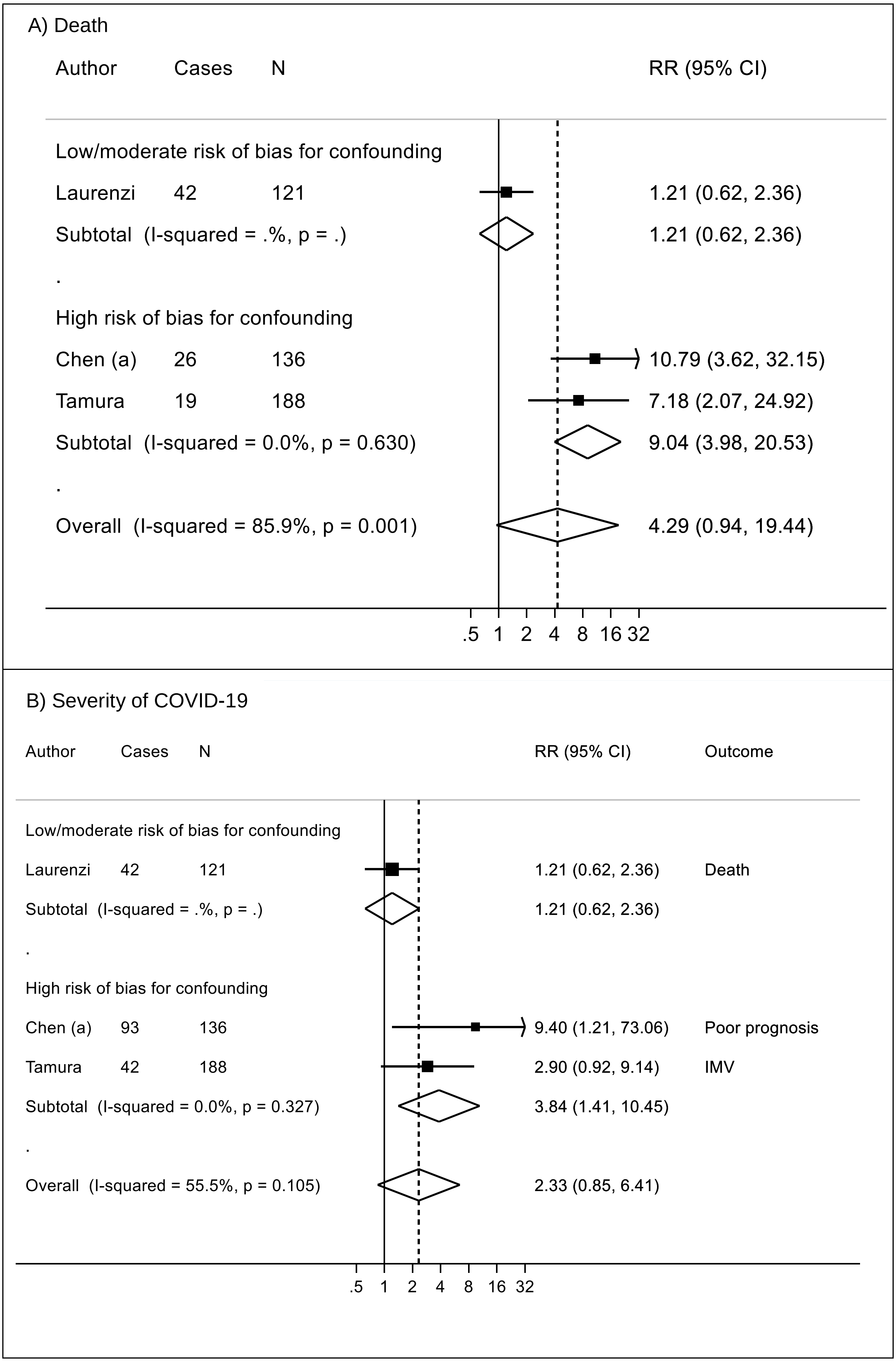

ESM Figure 57: Meta-analysis on pre-existing **neurodegenerative disease (not specified)** compared to no neurodegenerative disease and A) death and B) severity of COVID-19 in patients with diabetes and COVID-19

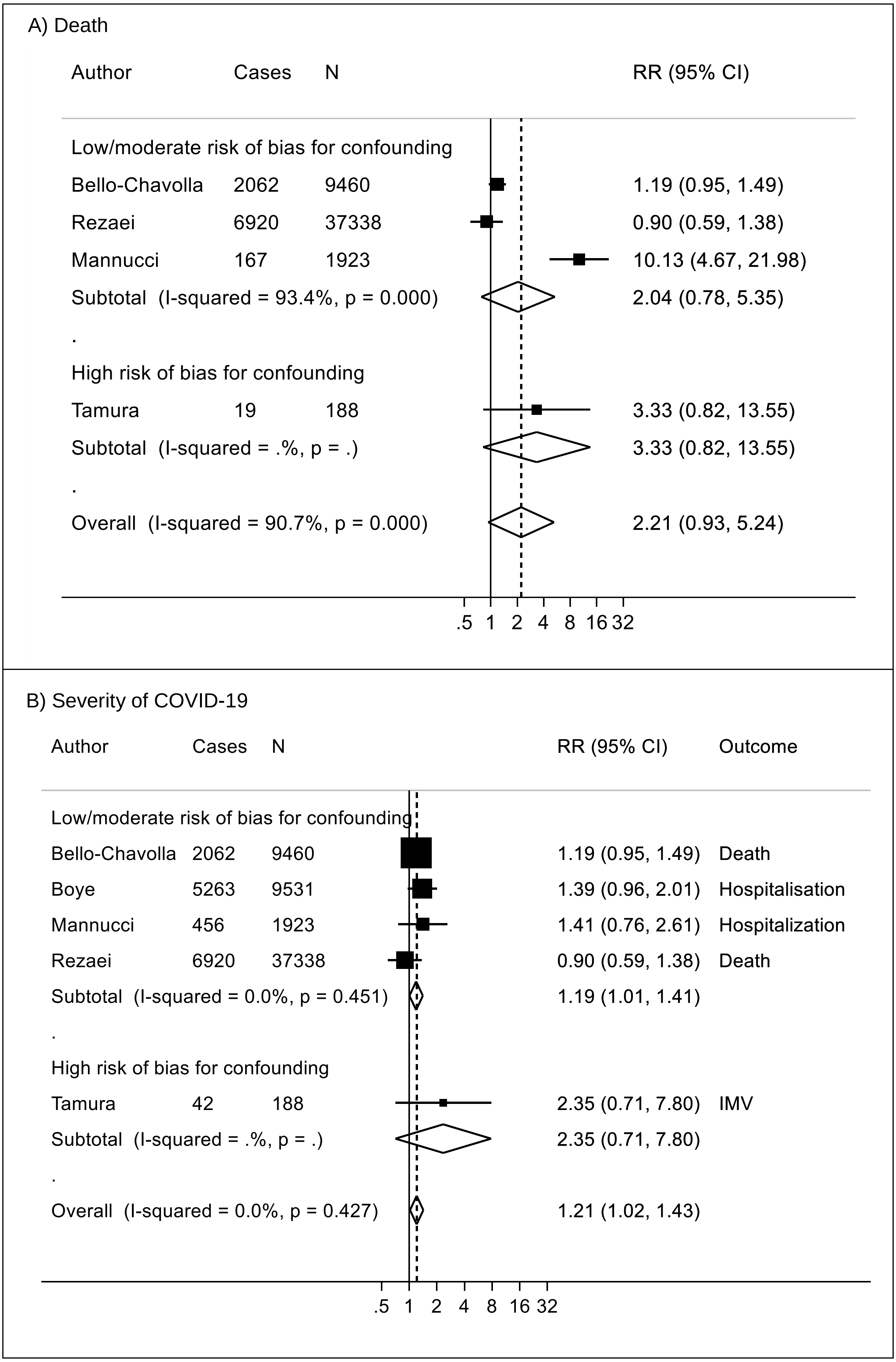

ESM Figure 58: Meta-analysis on pre-existing **immunodeficiency disorders (not specified)** compared to immunodeficiency disorders and A) death and B) severity of COVID-19 in patients with diabetes and COVID-19

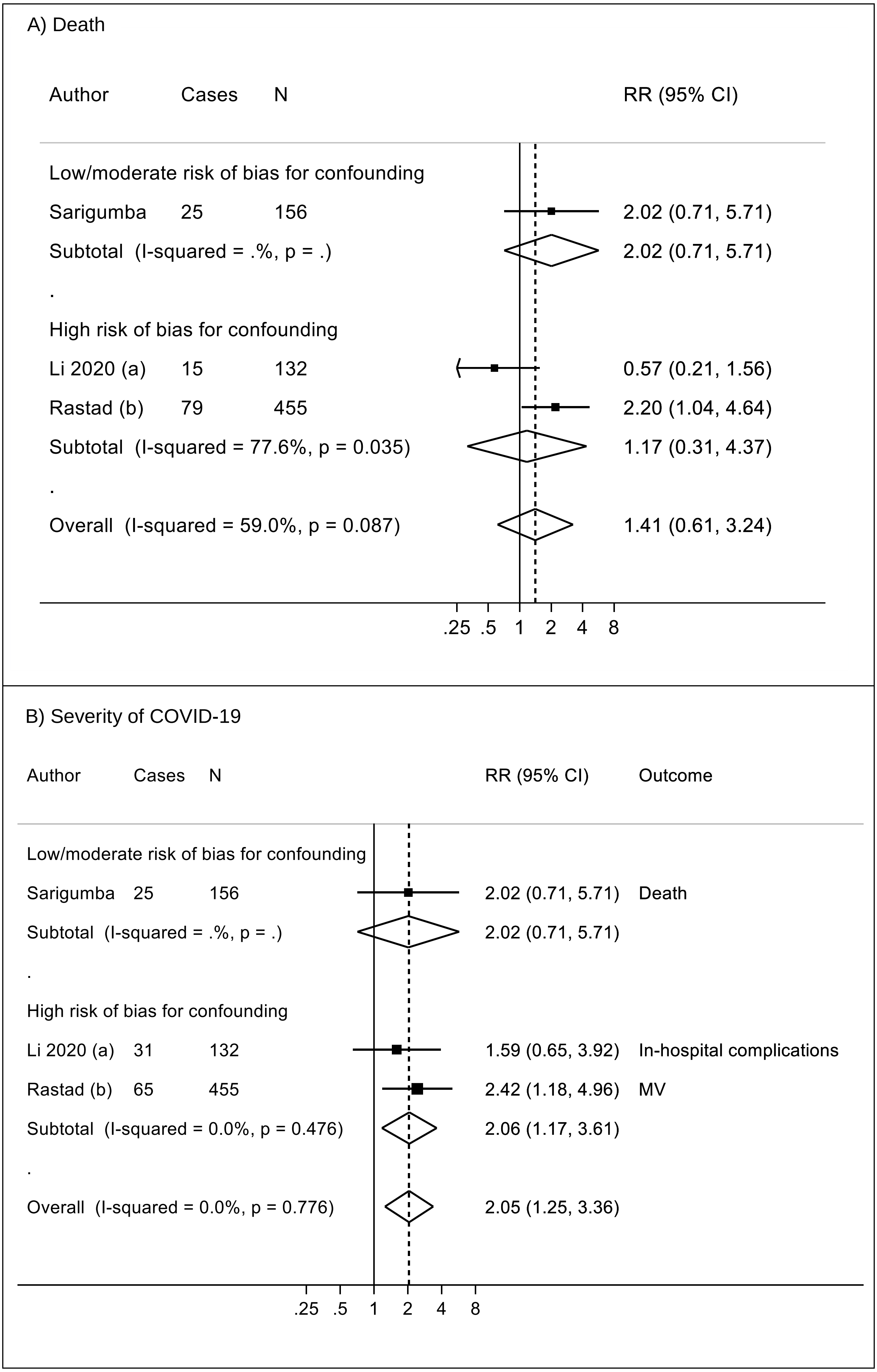

ESM Figure 59: Meta-analysis on **any comorbidities** compared to no comorbidities and A) death and B) severity of COVID-19 in patients with diabetes and COVID-19

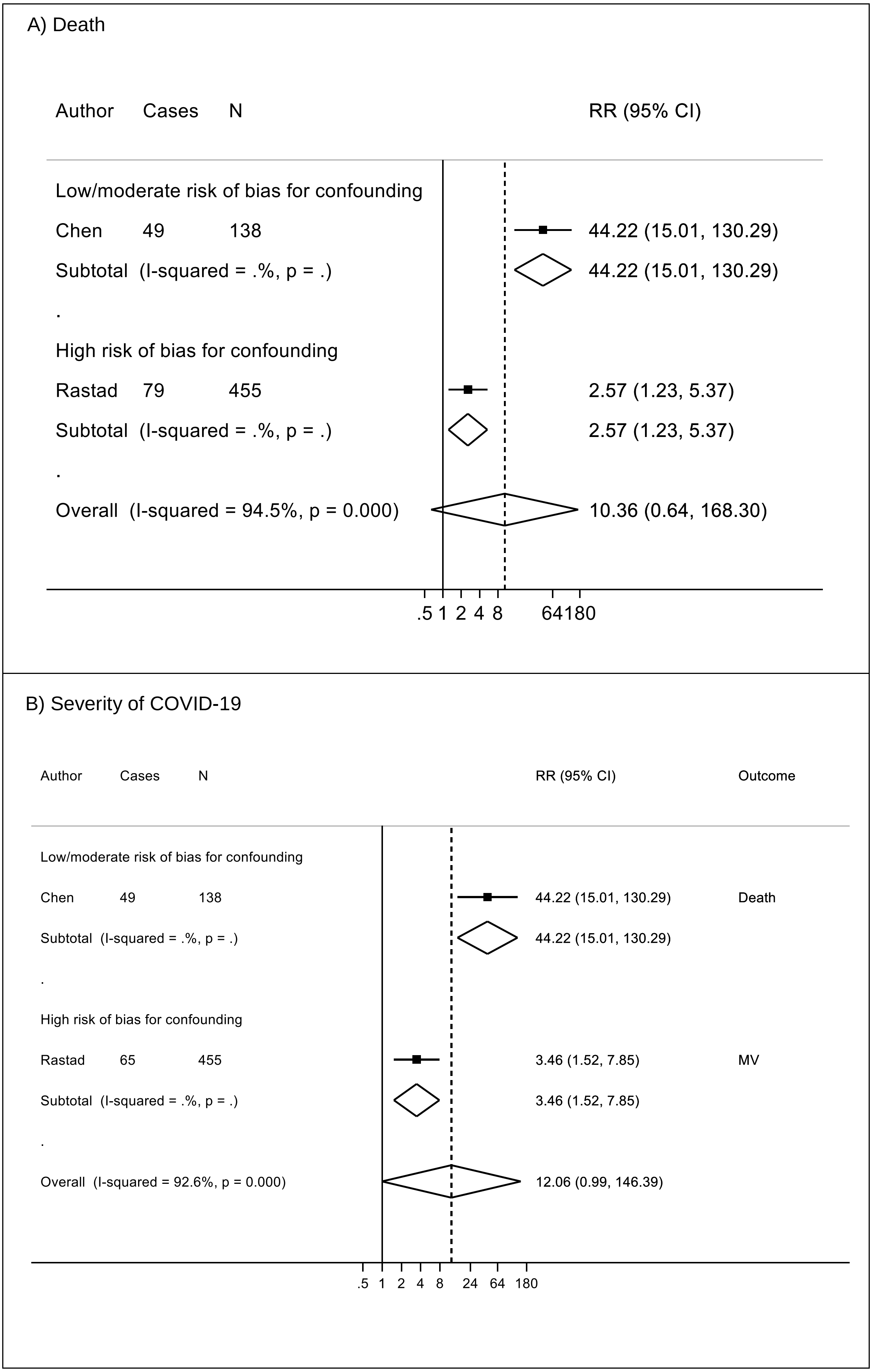

ESM Figure 60: Meta-analysis on **≥3 comorbidities** compared to no comorbidities and A) death and B) severity of COVID-19 in patients with diabetes and COVID-19

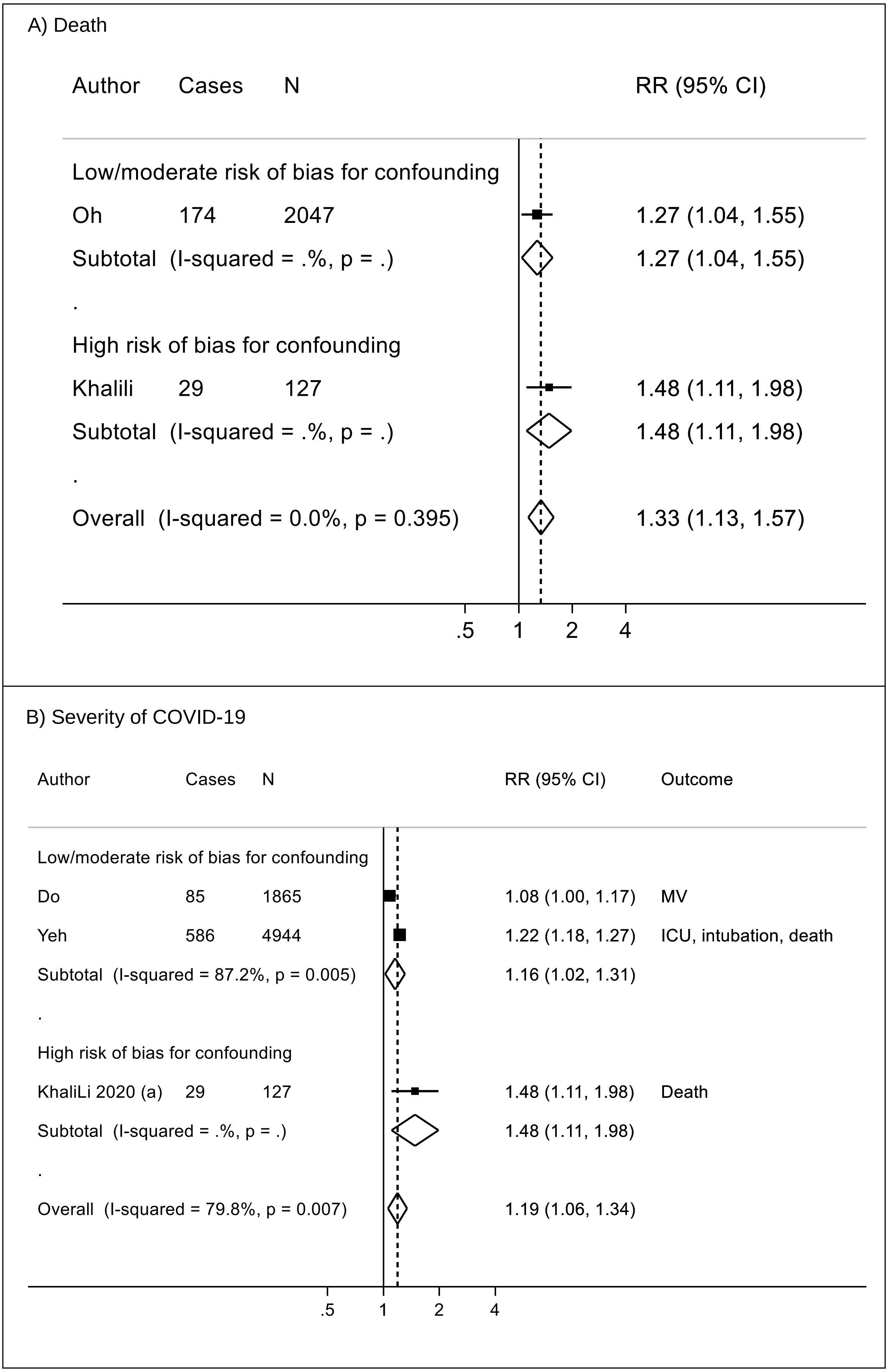

ESM Figure 61: Meta-analysis on **Charlson index**, per 1 unit compared to no comorbidities and A) death and B) severity of COVID-19 in patients with diabetes and COVID-19

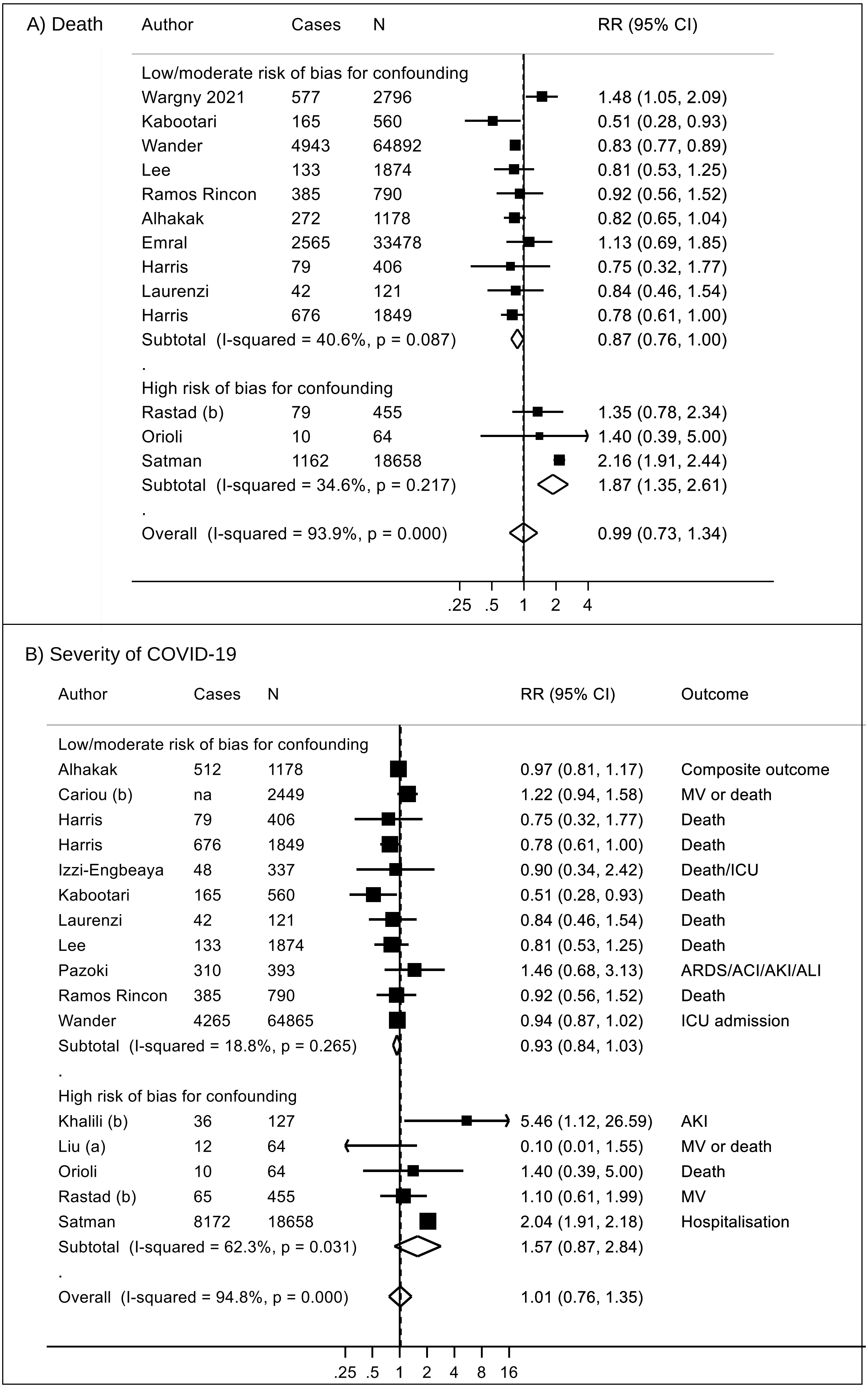

ESM Figure 62: Meta-analysis on **use of statins** compared to non-use and A) death and B) severity of COVID-19 in patients with diabetes and COVID-19

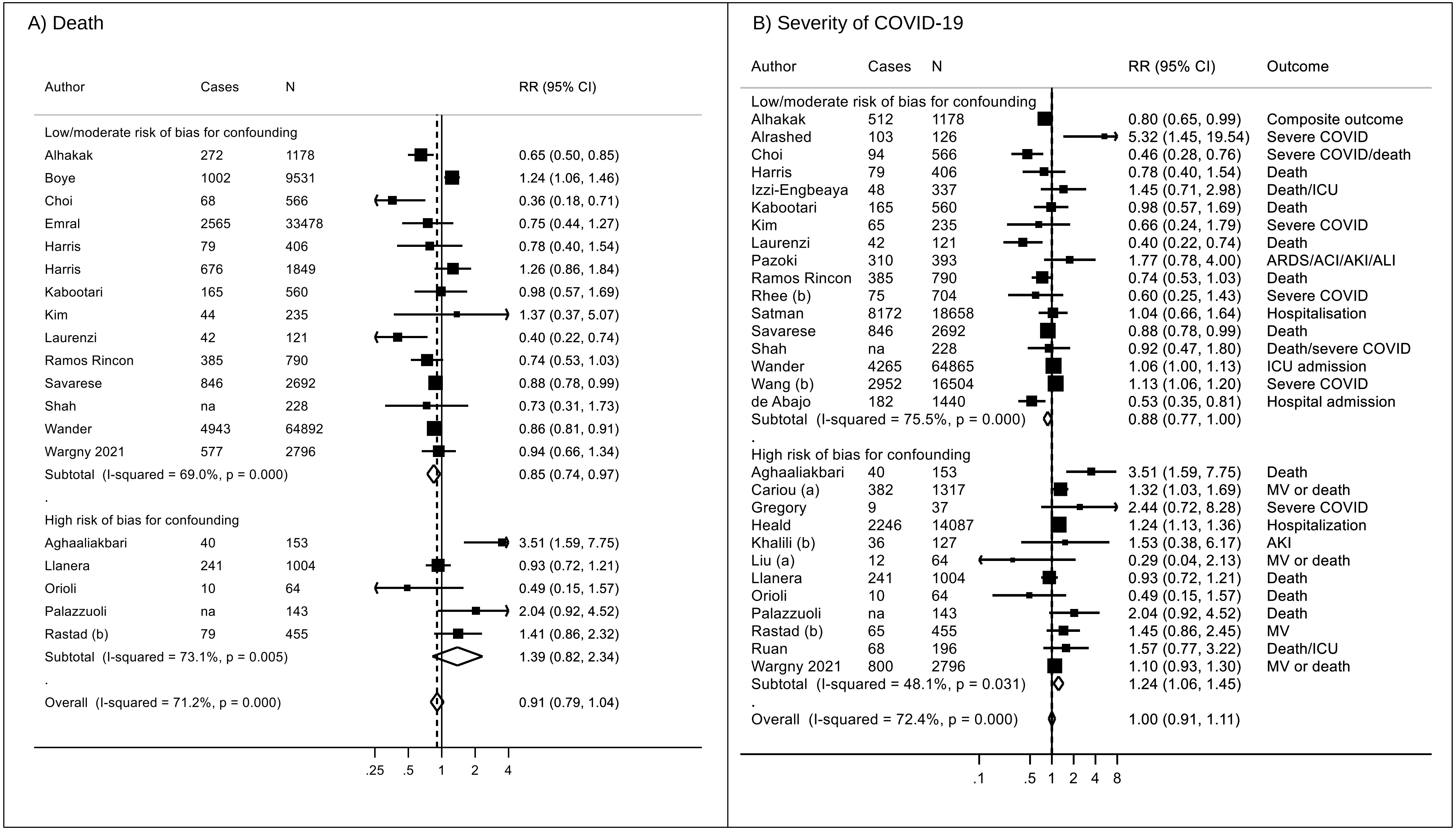

ESM Figure 63: Meta-analysis on **use of renin inhibitor** compared to non-use and A) death and B) severity of COVID-19 in patients with diabetes and COVID-19

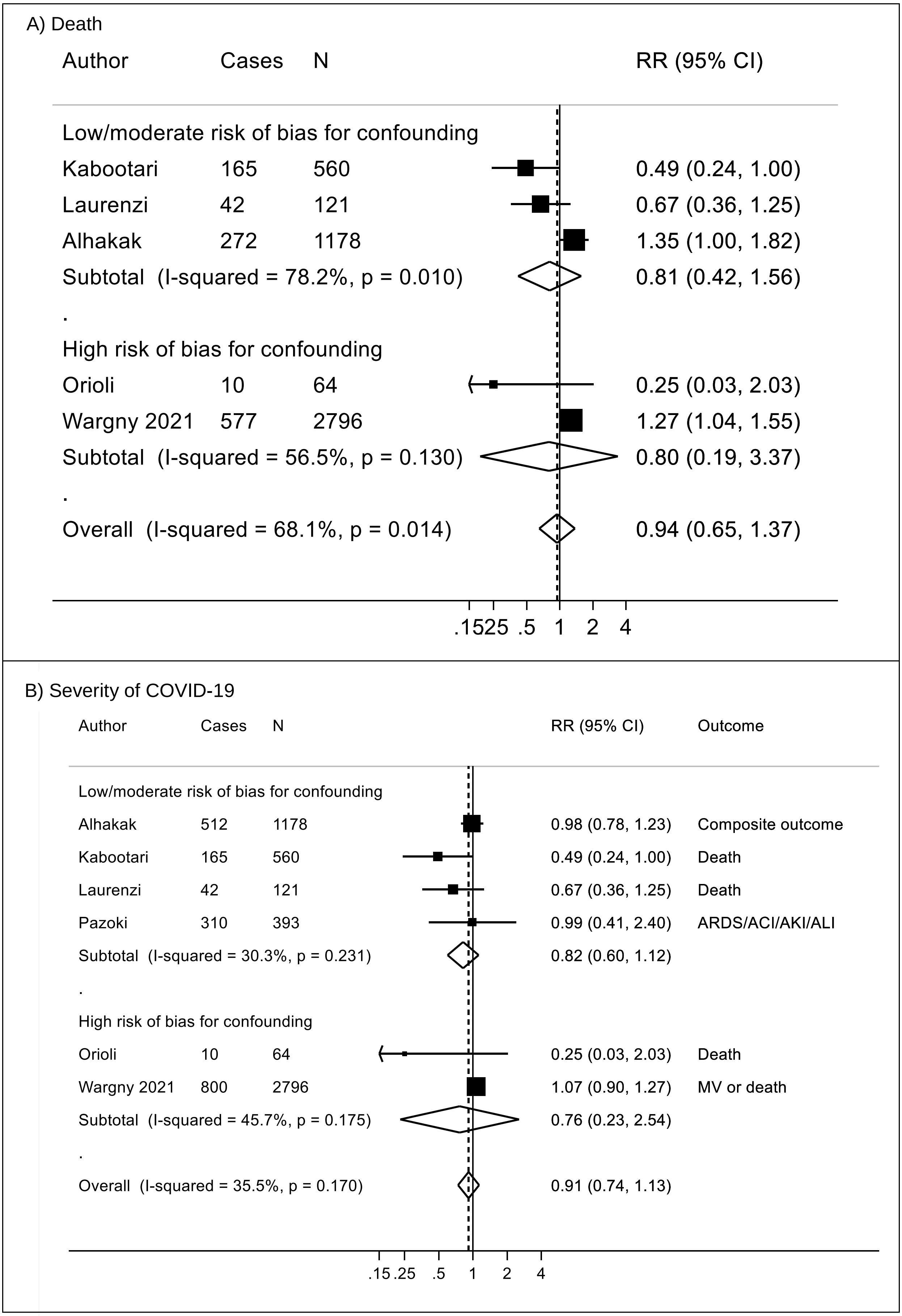

ESM Figure 64: Meta-analysis on **use of beta-blockers** compared to non-use and A) death and B) severity of COVID-19 in patients with diabetes and COVID-19

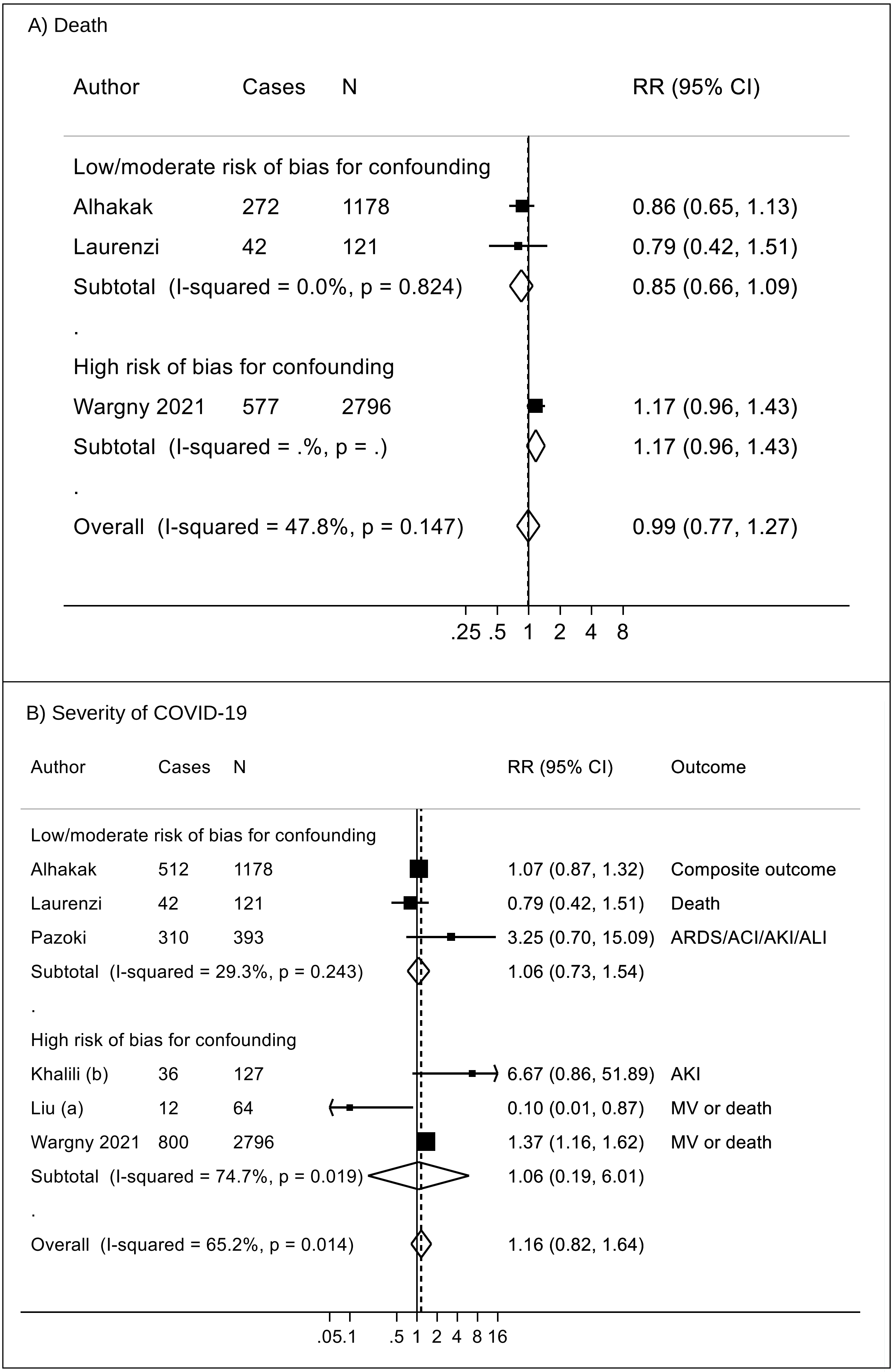

ESM Figure 65: Meta-analysis on **use of calcium channel blocker** compared to non-use and A) death and B) severity of COVID-19 in patients with diabetes and COVID-19

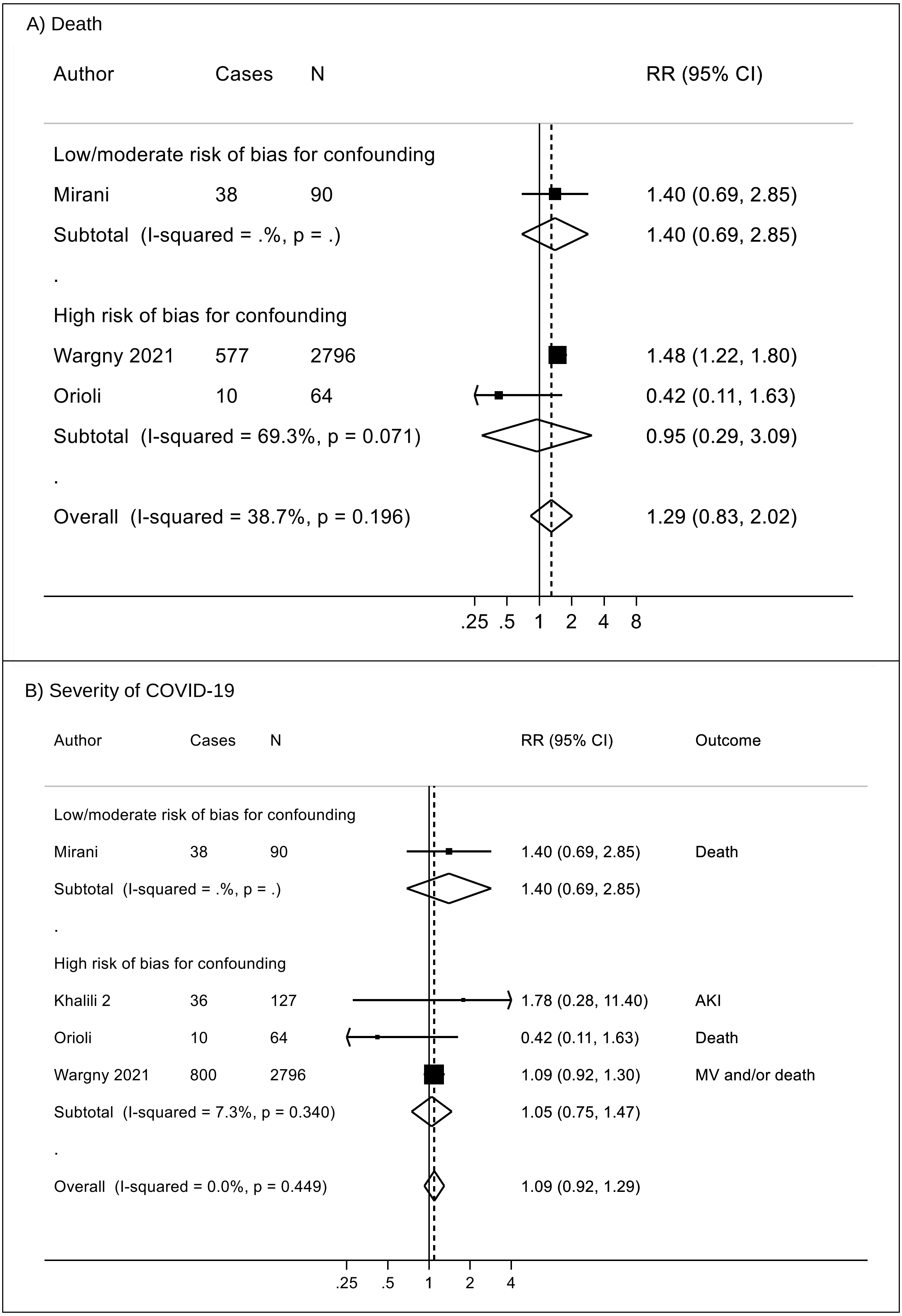

ESM Figure 66: Meta-analysis on **use of diuretics** compared to non-use and A) death and B) severity of COVID-19 in patients with diabetes and COVID-19

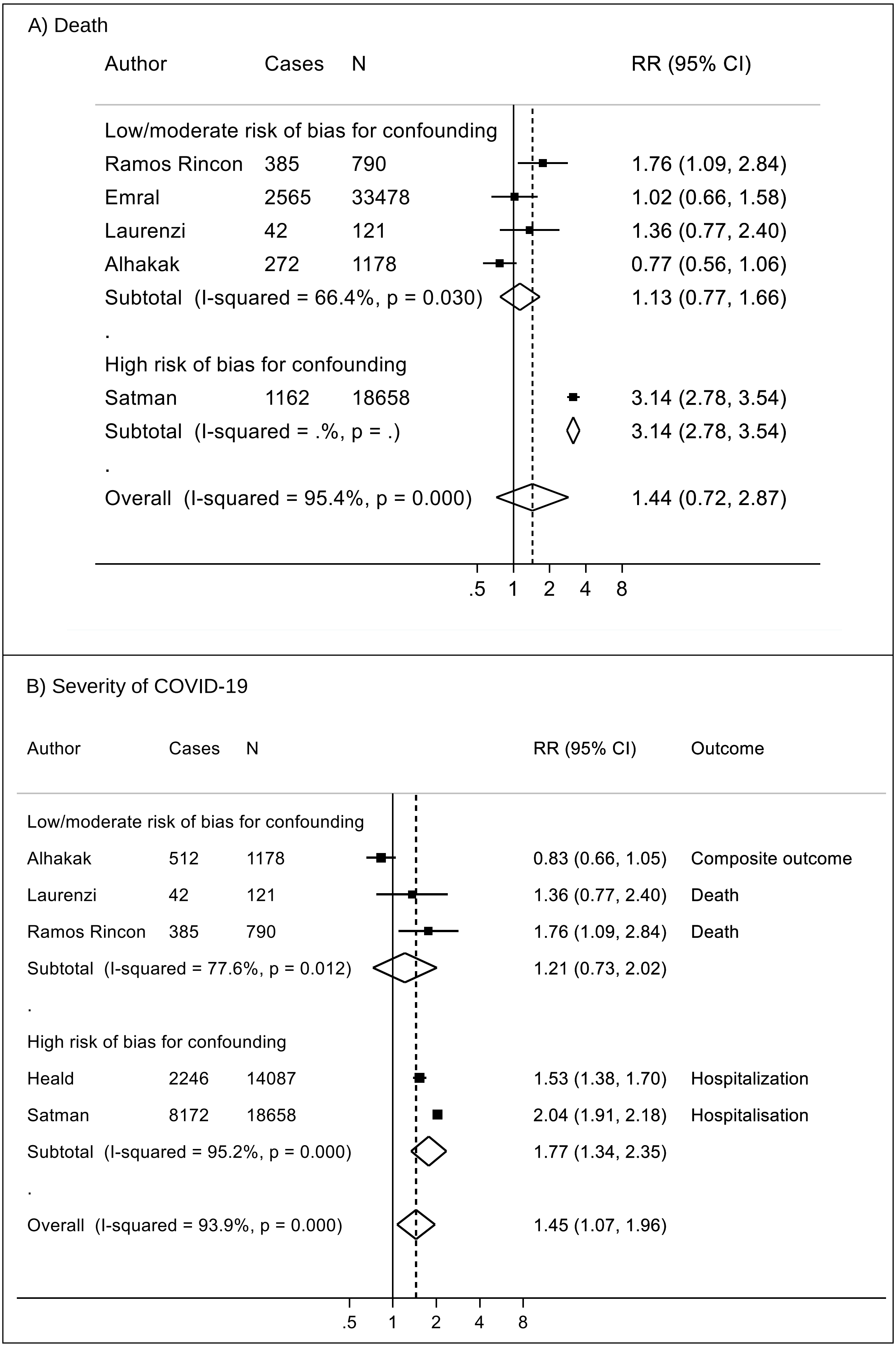

ESM Figure 67: Meta-analysis on **use of acetylsalicylic acid (ASA)** compared to non-use and A) death and B) severity of COVID-19 in patients with diabetes and COVID-19

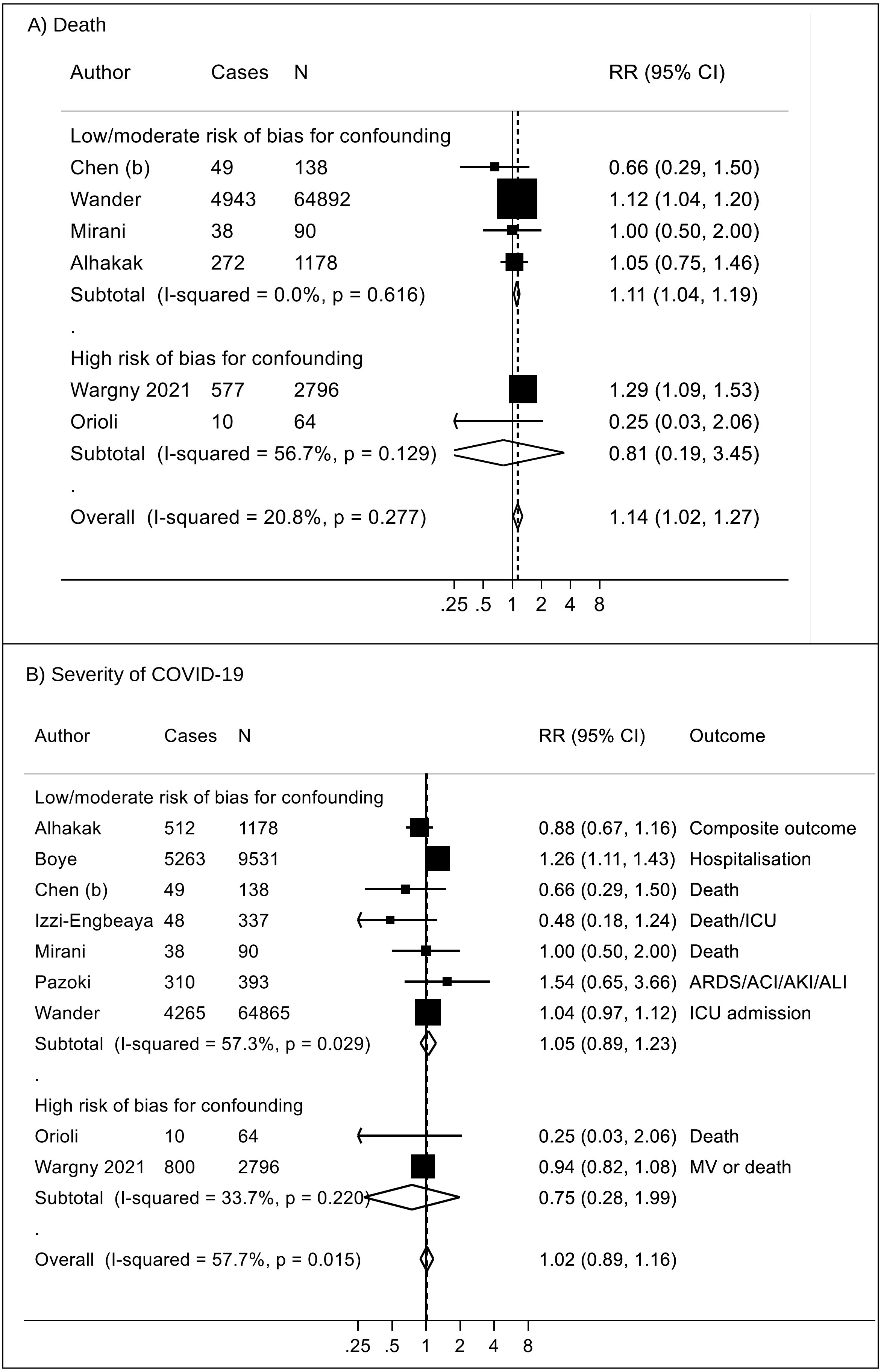

ESM Figure 68: Meta-analysis on **use of antithrombotic drugs (antiplatelet/ anticoagulant)** compared to non-use and A) death and B) severity of COVID-19 in patients with diabetes and COVID-19

A) Death

No data

B) Severity of COVID-19

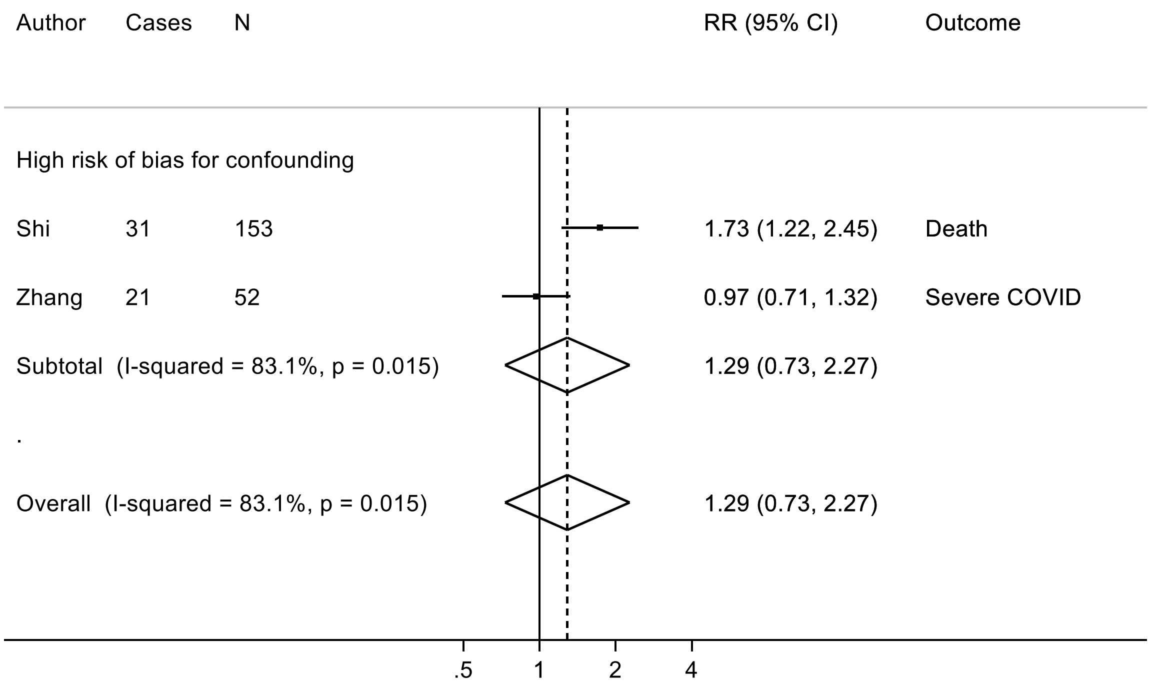

ESM Figure 69: Meta-analysis on **triglycerides**, per 1 per mmol/l and A) death and B) severity of COVID-19 in patients with diabetes and COVID-19

A) Death

No data

B) Severity of COVID-19

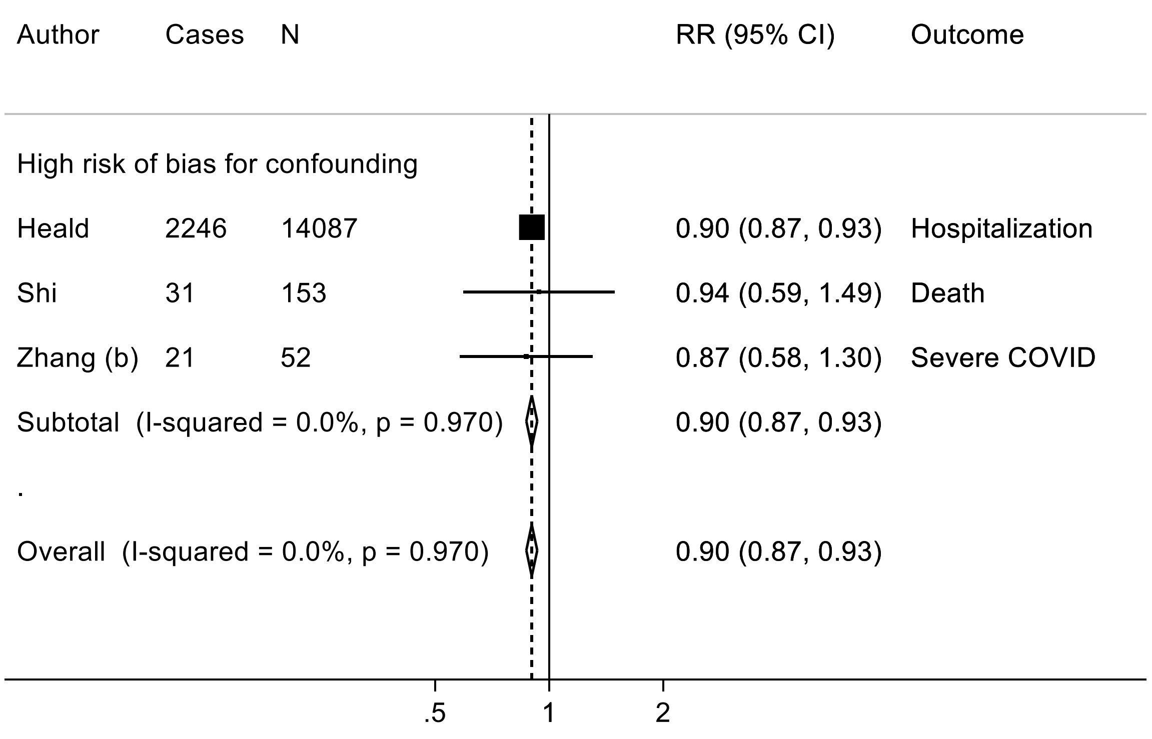

ESM Figure 70: Meta-analysis on **total cholesterol,** per 1 per mmol/l and A) death and B) severity of COVID-19 in patients with diabetes and COVID-19

A) Death

No data

B) Severity of COVID-19

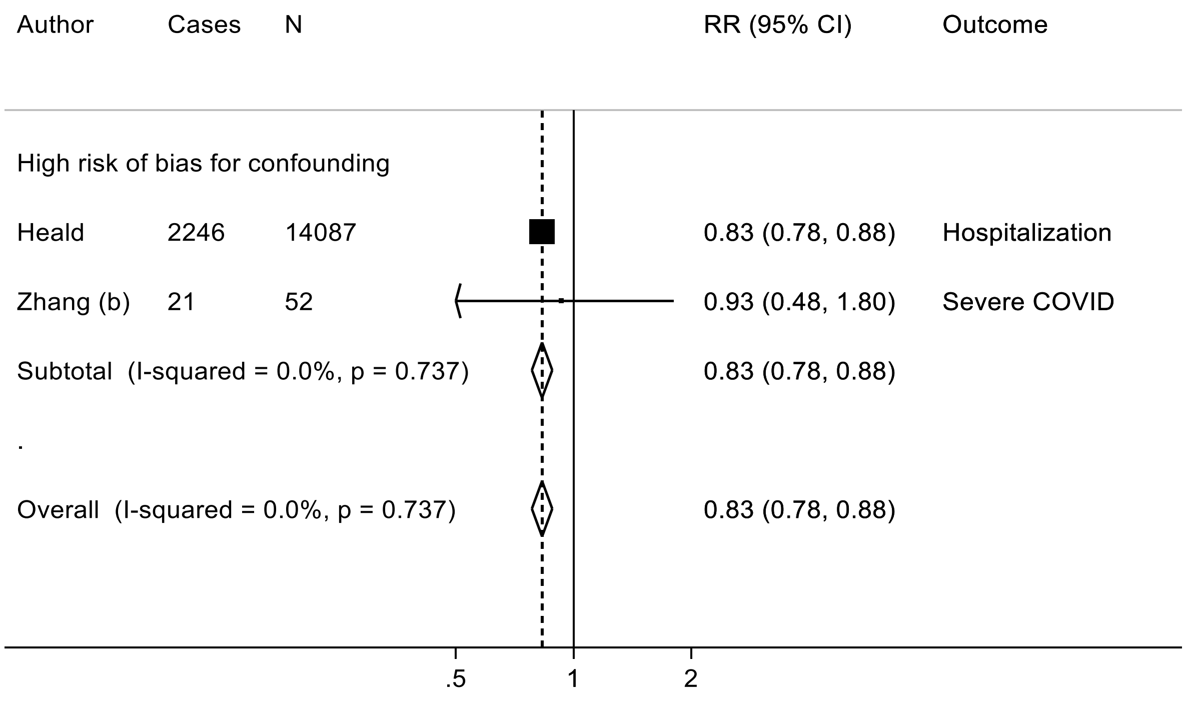

ESM Figure 71: Meta-analysis on **LDL-cholesterol,** per 1 per mmol/l and A) death and B) severity of COVID-19 in patients with diabetes and COVID-19

A) Death

No data

B) Severity of COVID-19

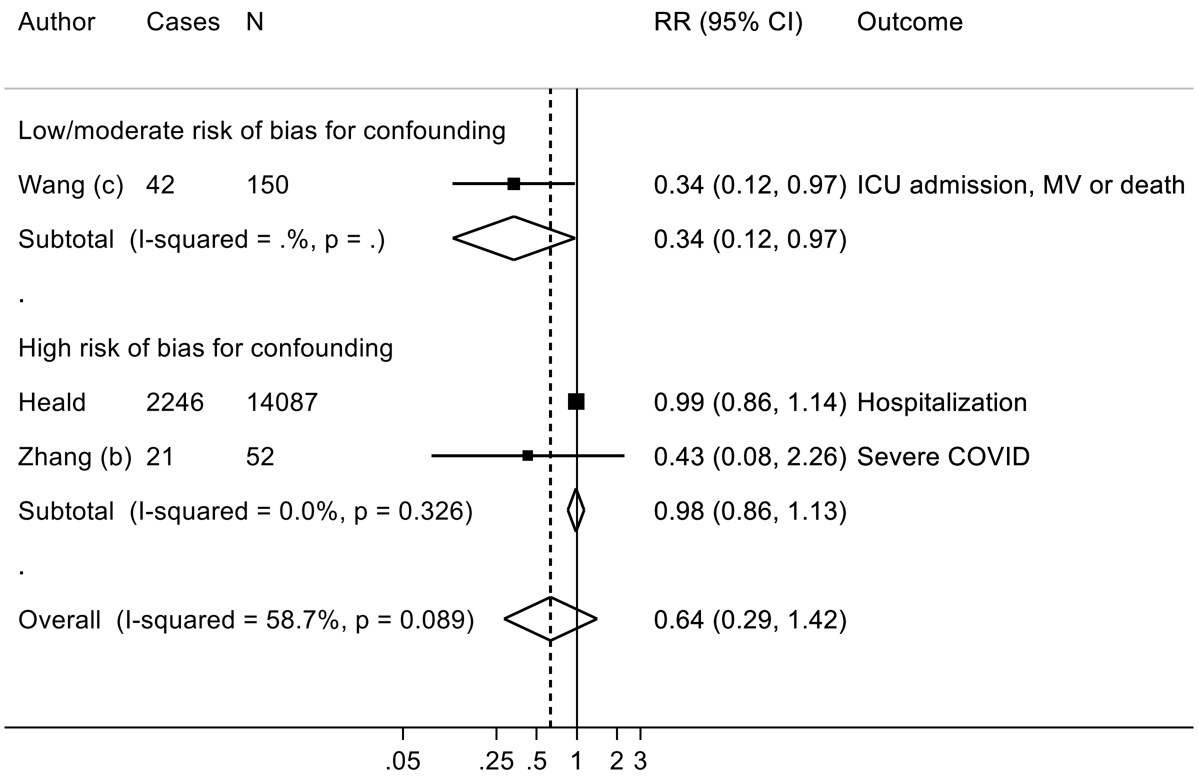

ESM Figure 72: Meta-analysis on **HDL-cholesterol,** per 1 per mmol/l and A) death and B) severity of COVID-19 in patients with diabetes and COVID-19

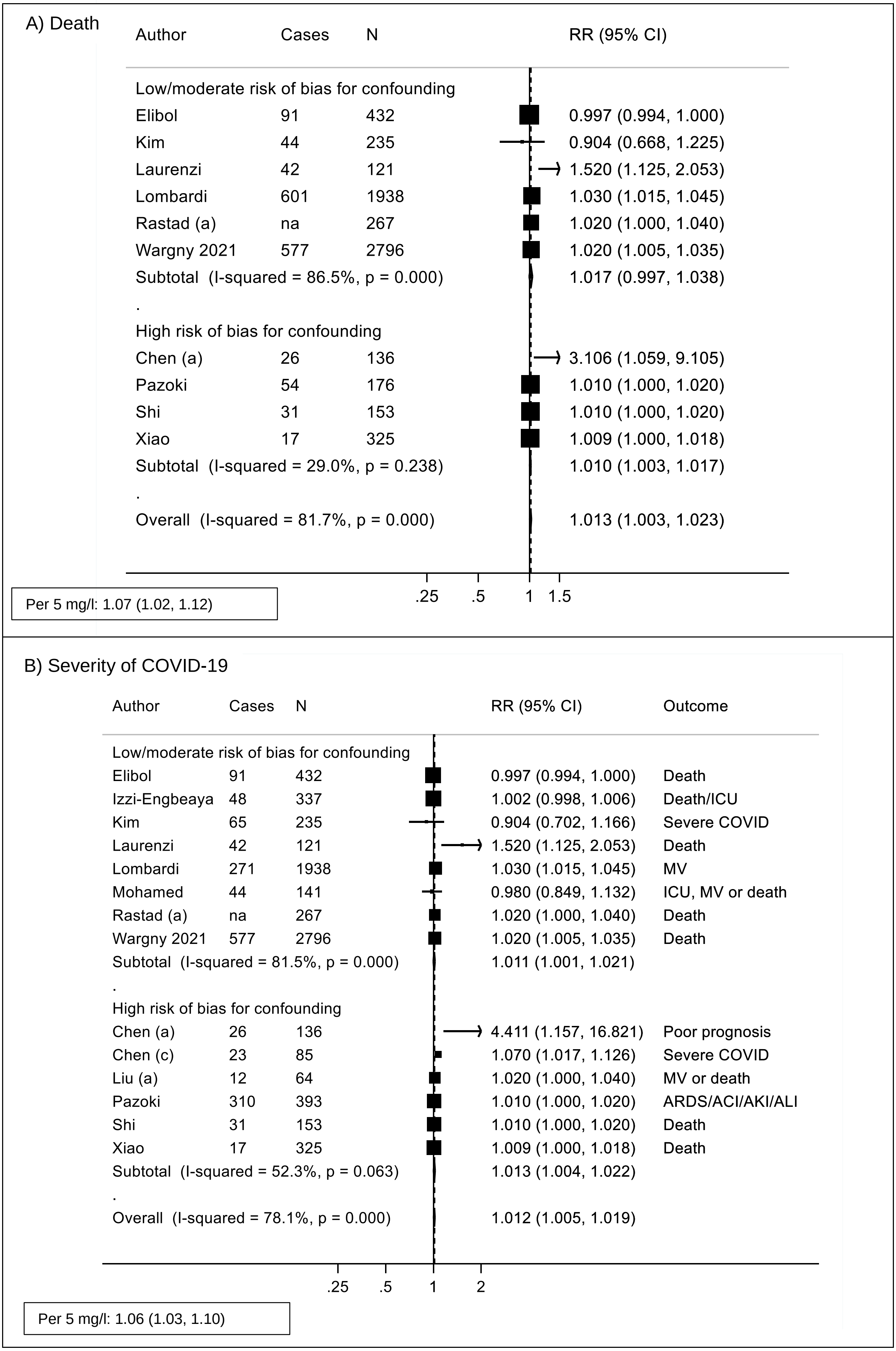

ESM Figure 73: Meta-analysis on **C-reactive protein (CRP)**, per 1 mg/l and A) death and B) severity of COVID-19 in patients with diabetes and COVID-19

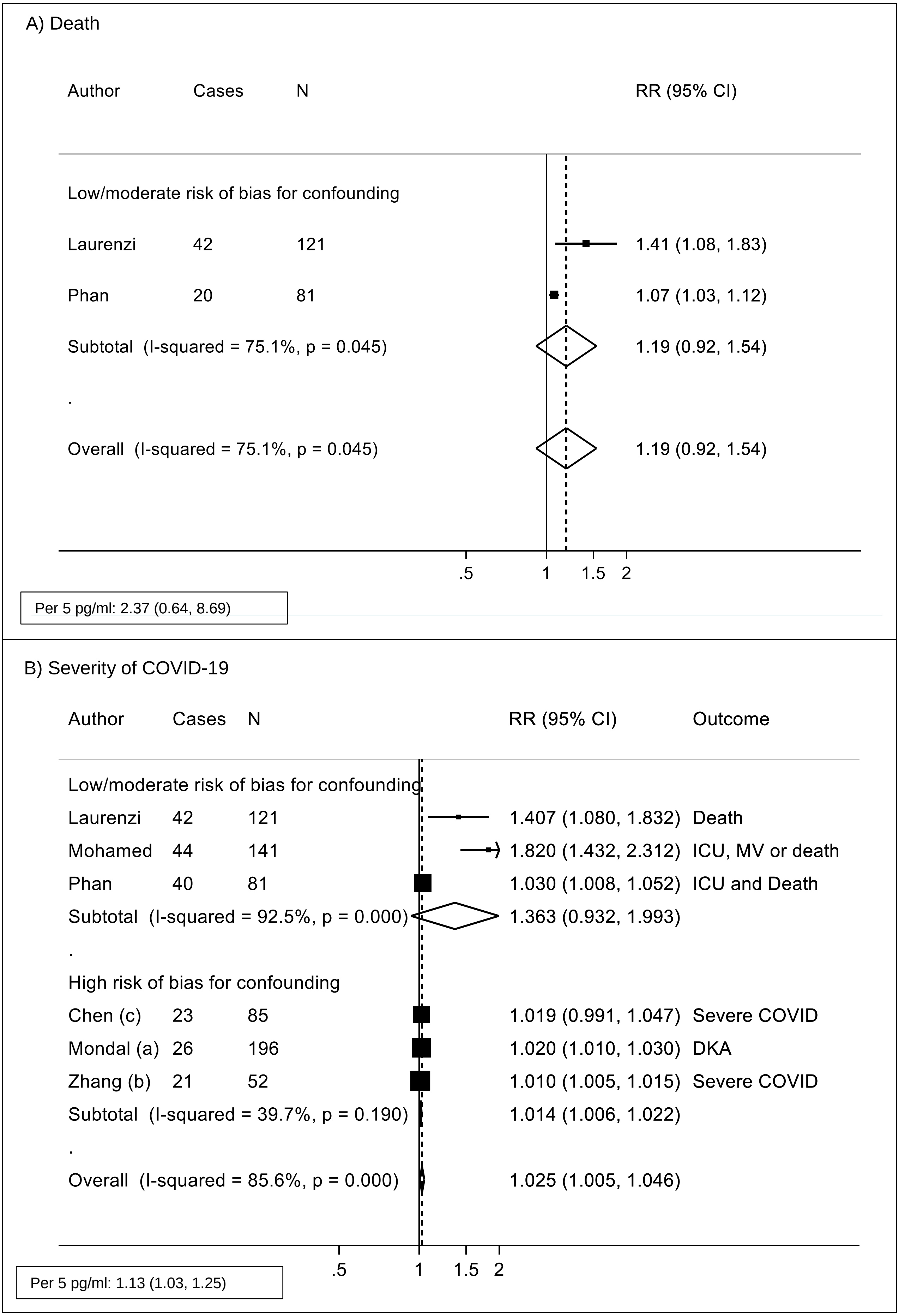

ESM Figure 74: Meta-analysis on **IL-6**, per 1 pg/ml and A) death and B) severity of COVID-19 in patients with diabetes and COVID-19

A) Death

No data

B) Severity of COVID-19

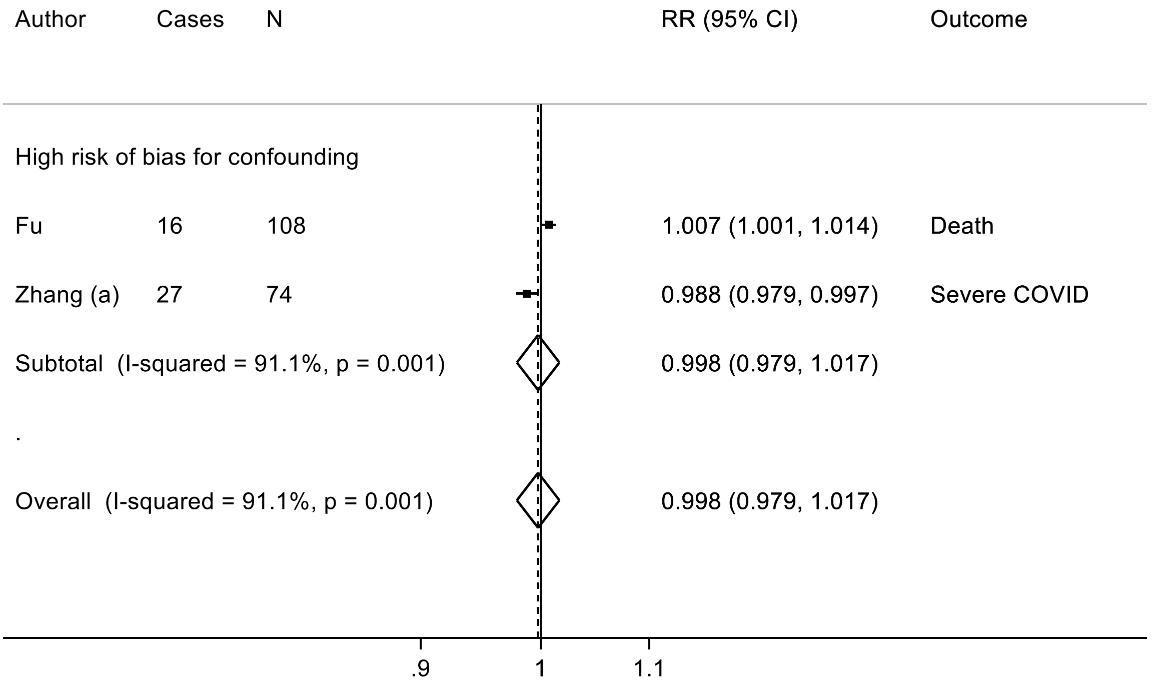

ESM Figure 75: Meta-analysis on **serum amyloid A**, per 1 mg/l and A) death and B) severity of COVID-19 in patients with diabetes and COVID-19

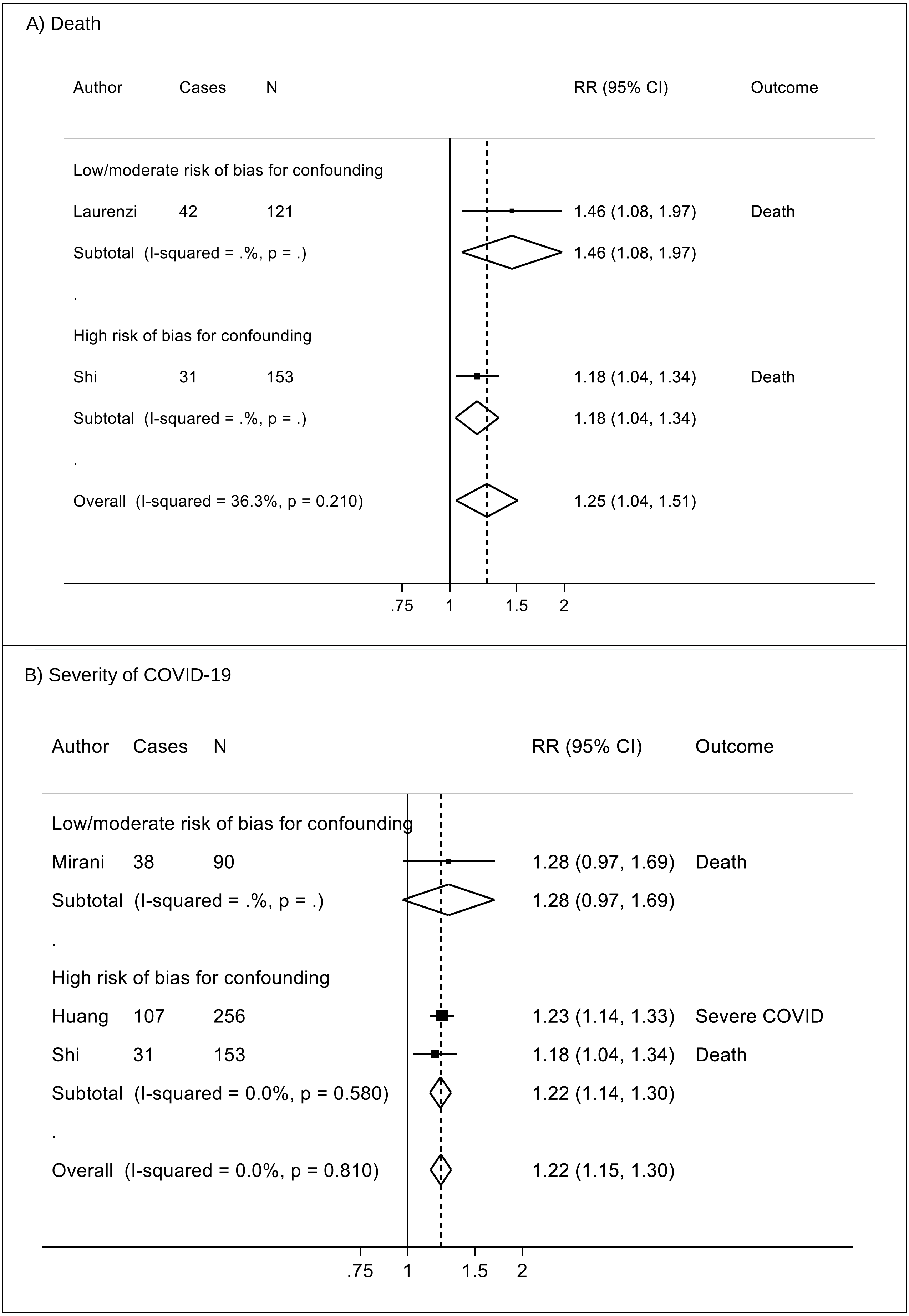

ESM Figure 76: Meta-analysis on **procalcitonin**, per 1 ng/ml and A) death and B) severity of COVID-19 in patients with diabetes and COVID-19

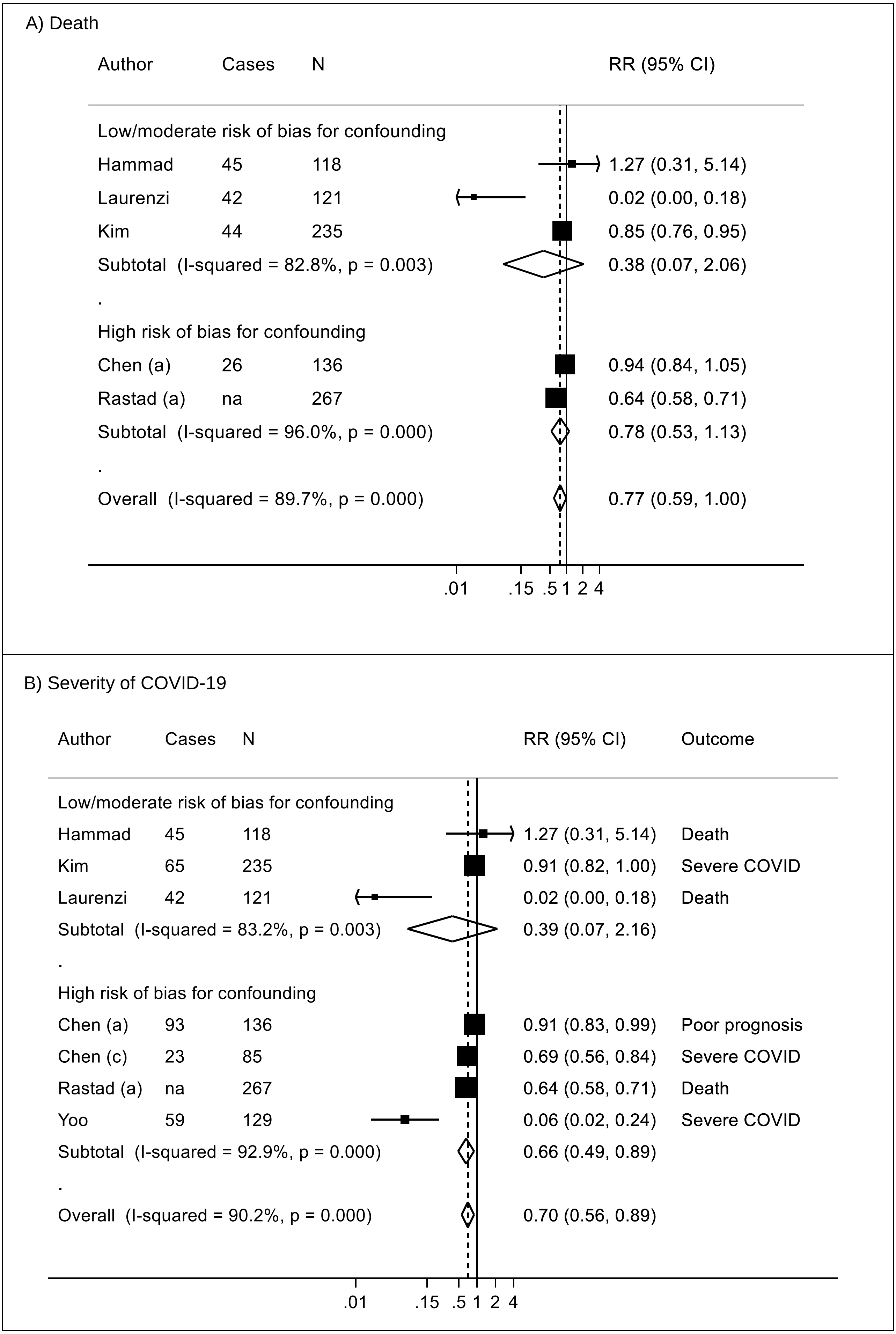

ESM Figure 77: Meta-analysis on **albumin**, per 1 g/l and A) death and B) severity of COVID-19 in patients with diabetes and COVID-19

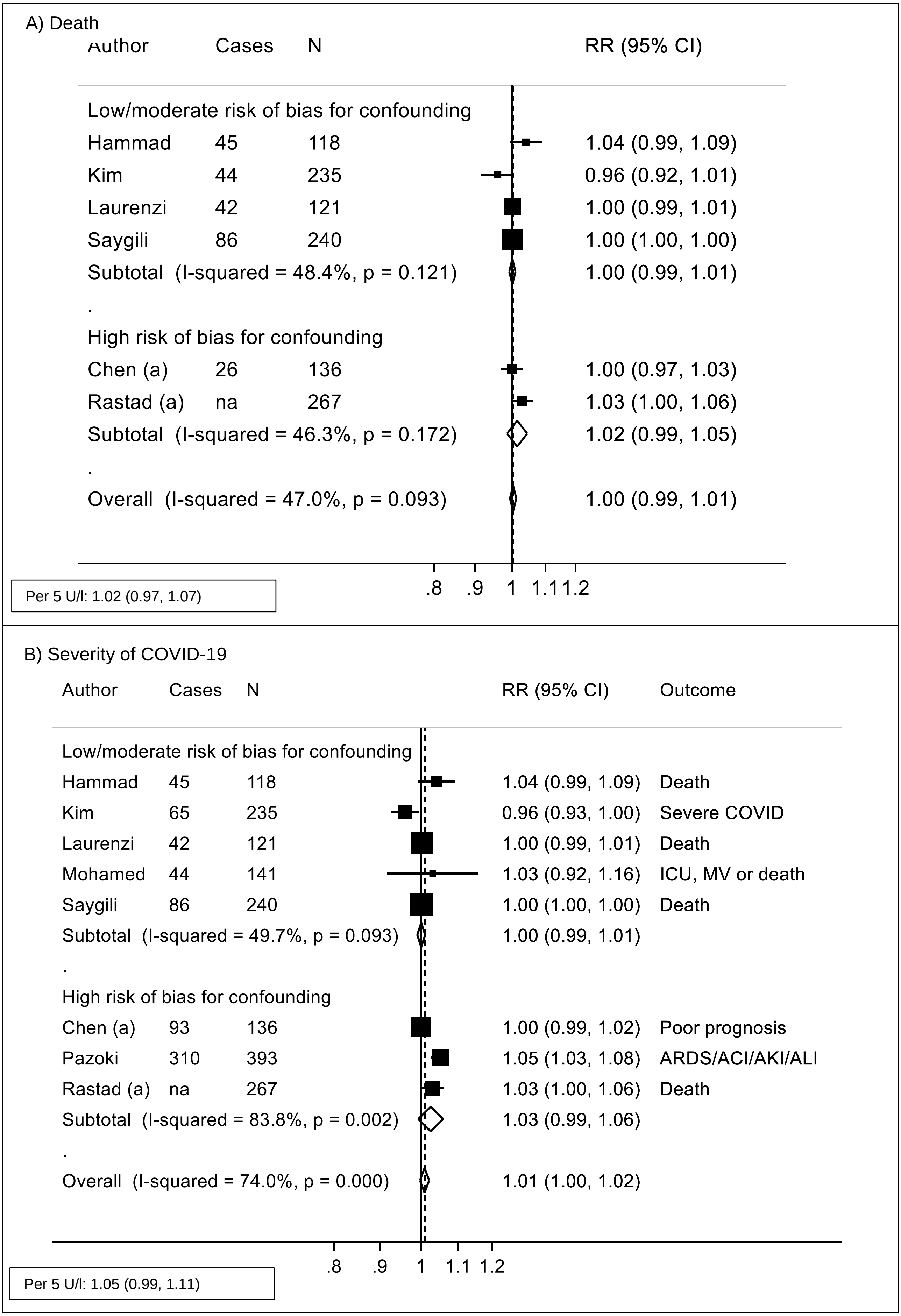

ESM Figure 78: Meta-analysis on **ALT**, per 1U/l and A) death and B) severity of COVID-19 in patients with diabetes and COVID-19

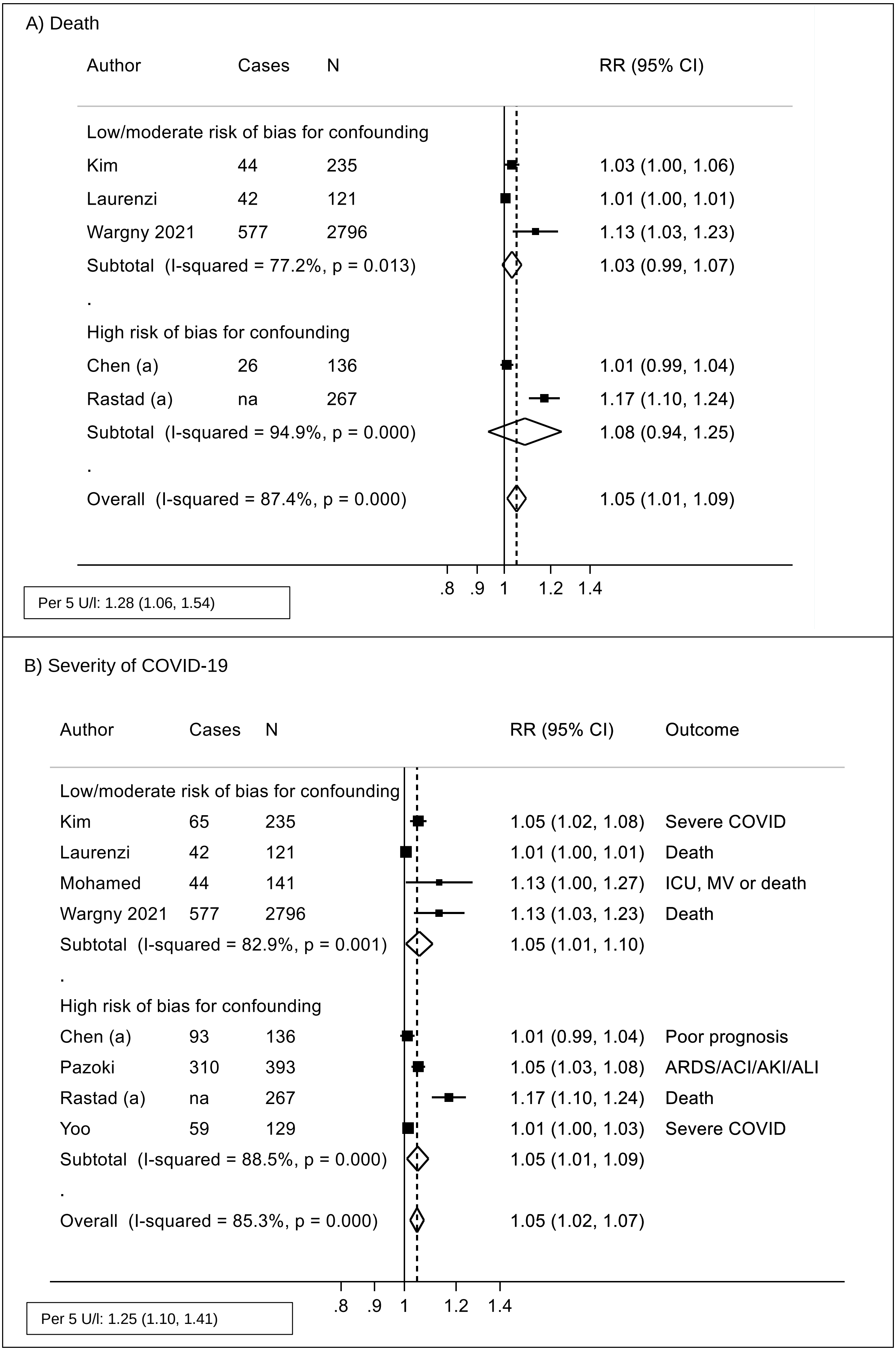

ESM Figure 79: Meta-analysis on **AST** per 1U/l and A) death and B) severity of COVID-19 in patients with diabetes and COVID-19

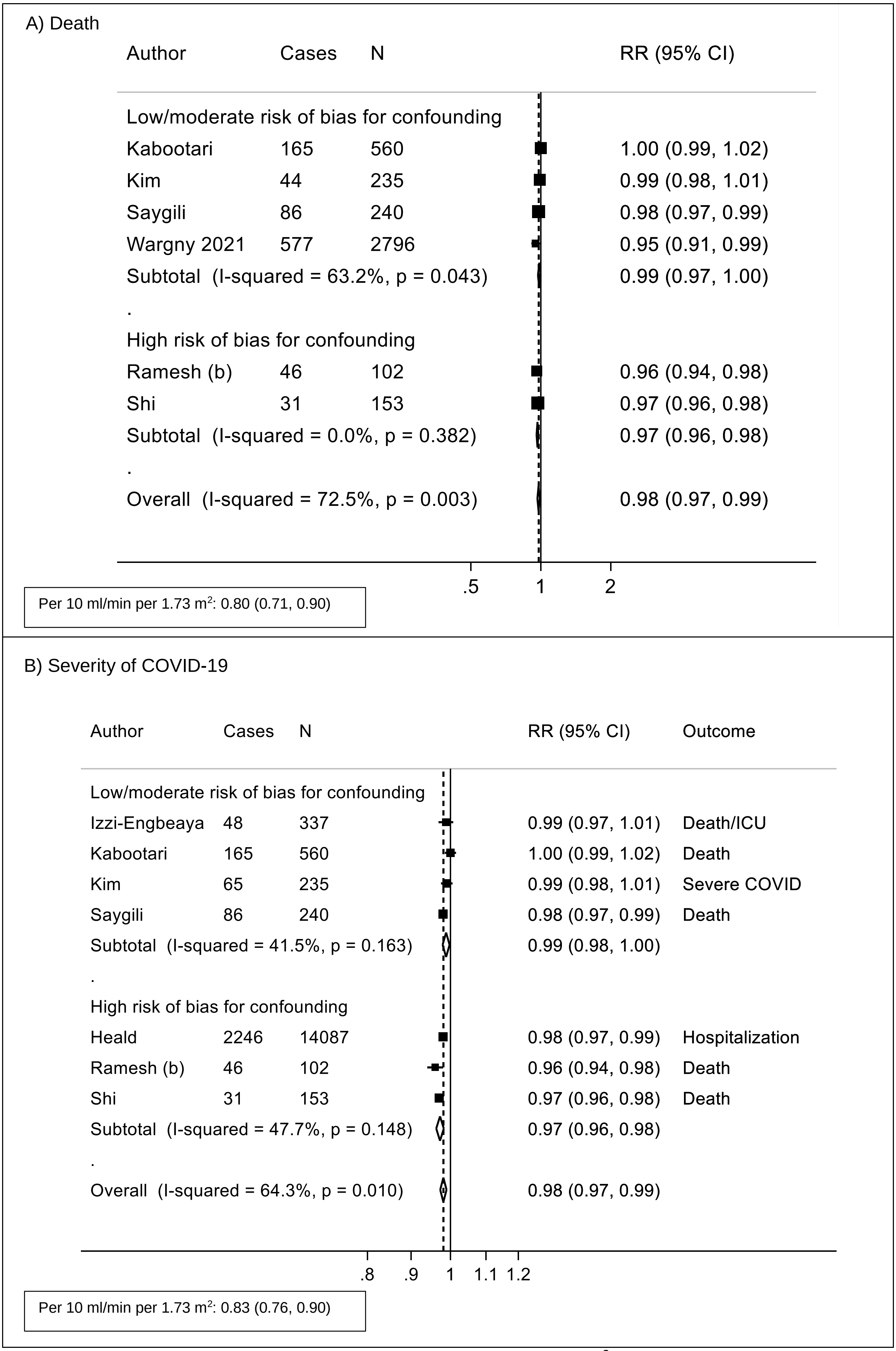

ESM Figure 80: Meta-analysis on **eGFR**, per 1 ml/min per 1.73 m^2 and A) death and B) severity of COVID-19 in patients with diabetes and COVID-19

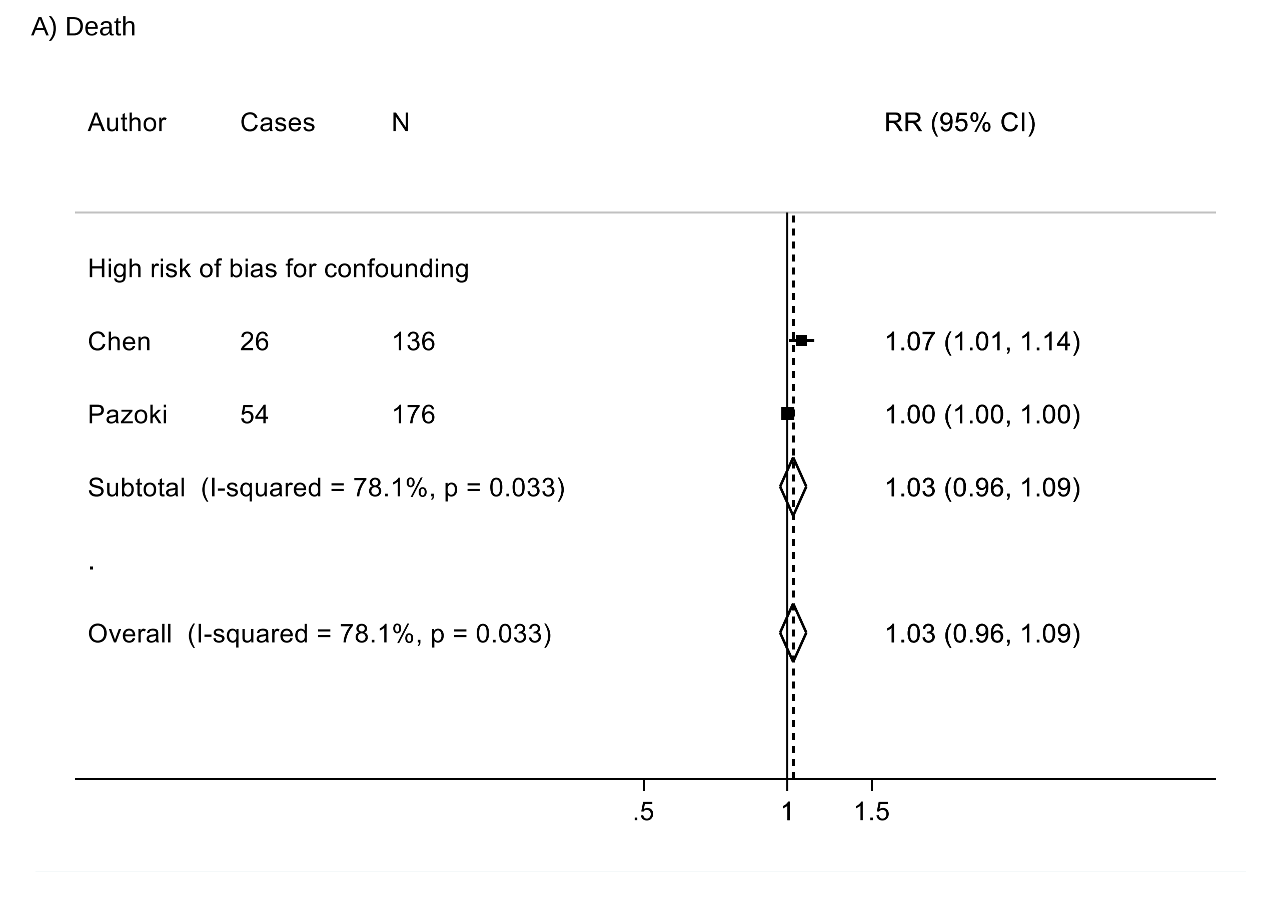

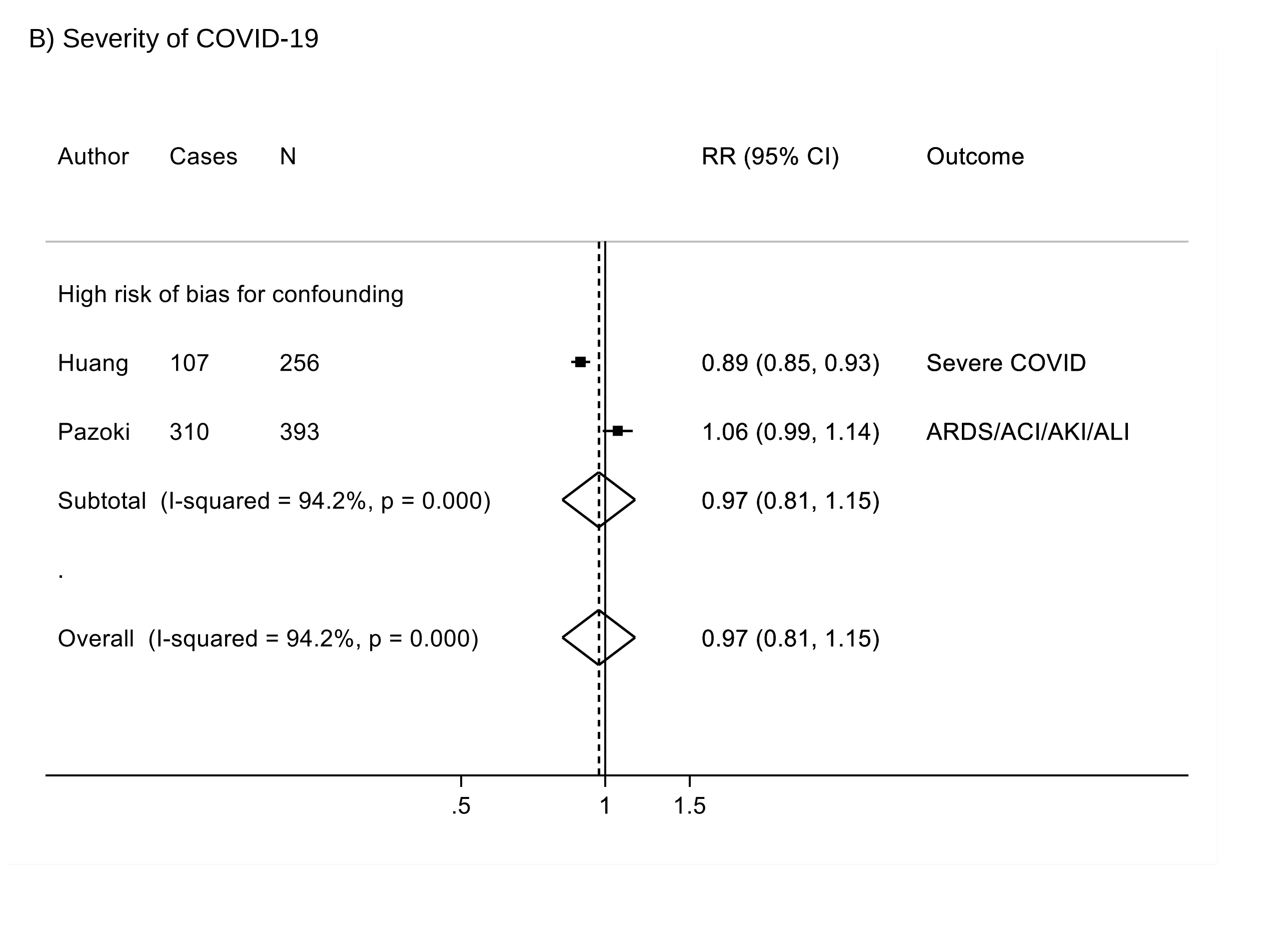

ESM Figure 81: Meta-analysis on **urea**, per 1 mmol/l and A) death and B) severity of COVID-19 in patients with diabetes and COVID-19

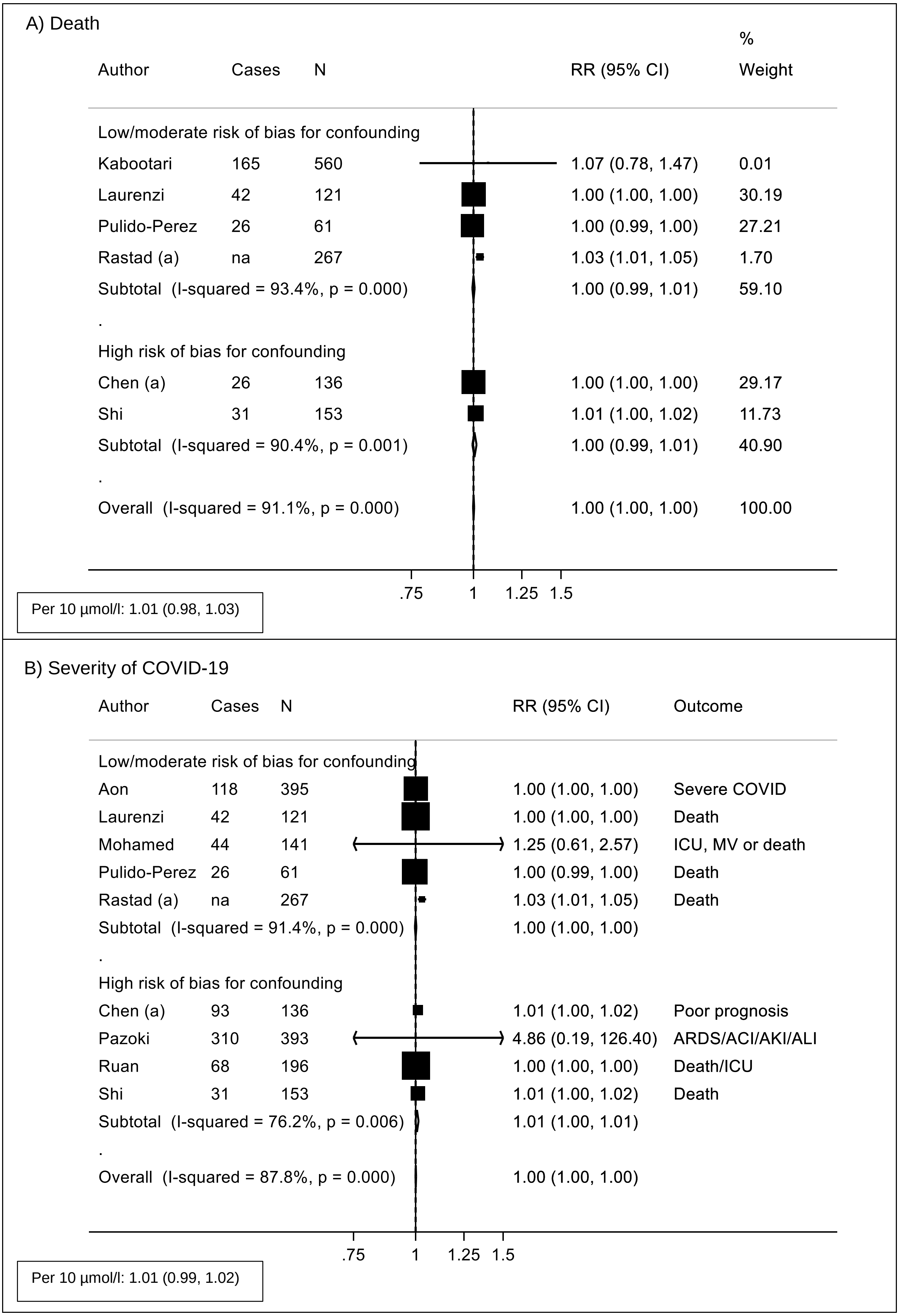

ESM Figure 82: Meta-analysis on **creatinine,** per 1 µmol/l and A) death and B) severity of COVID-19 in patients with diabetes and COVID-19

A) Death

No data

B) Severity of COVID-19

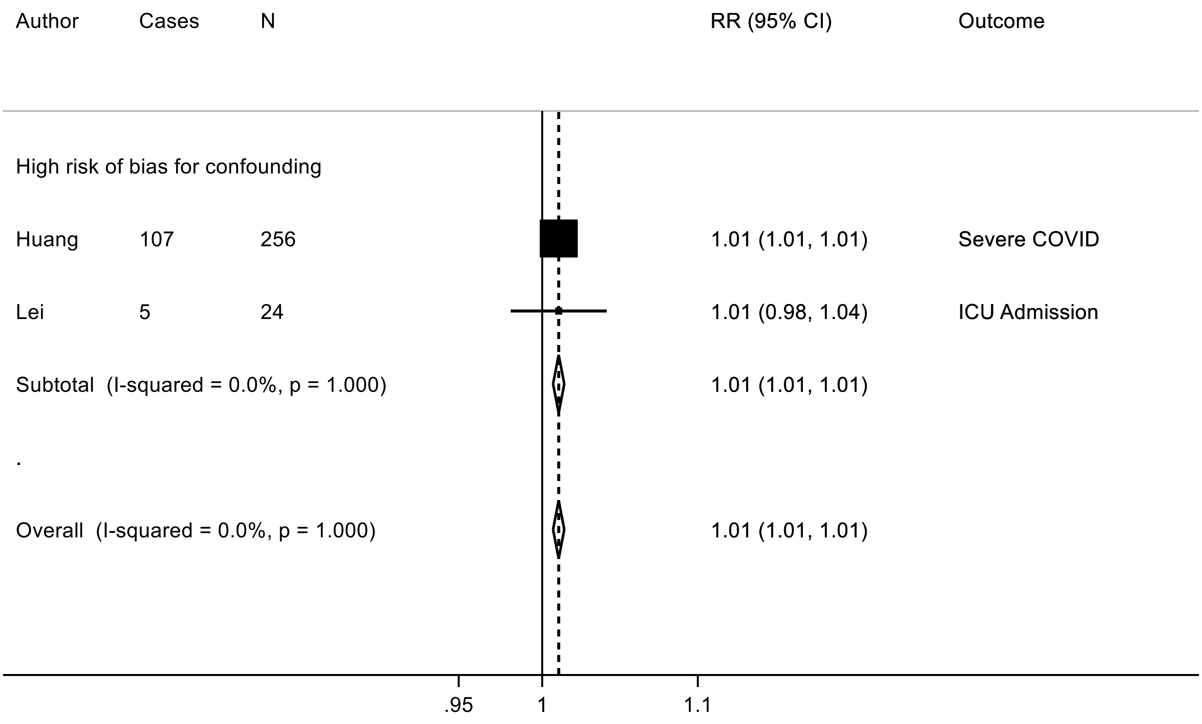

ESM Figure 83: Meta-analysis on **creatine phosphokinase (CPK),** per 1 U/l and A) death and B) severity of COVID-19 in patients with diabetes and COVID-19

A) Death

No data

B) Severity of COVID-19

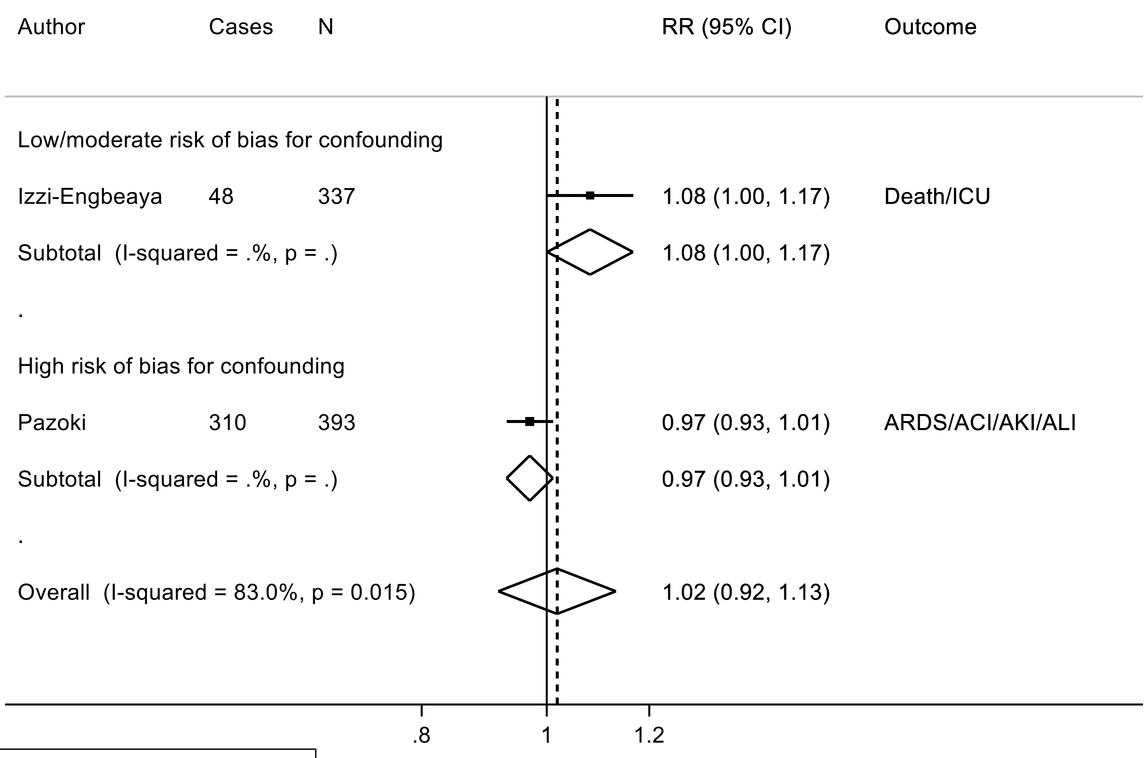

Per 10 mmol/l: 1.20 (0.42, 3.42)

ESM Figure 84: Meta-analysis on **serum sodium,** per 1 mmol/l and A) death and B) severity of COVID-19 in patients with diabetes and COVID-19

A) Death

No data

B) Severity of COVID-19

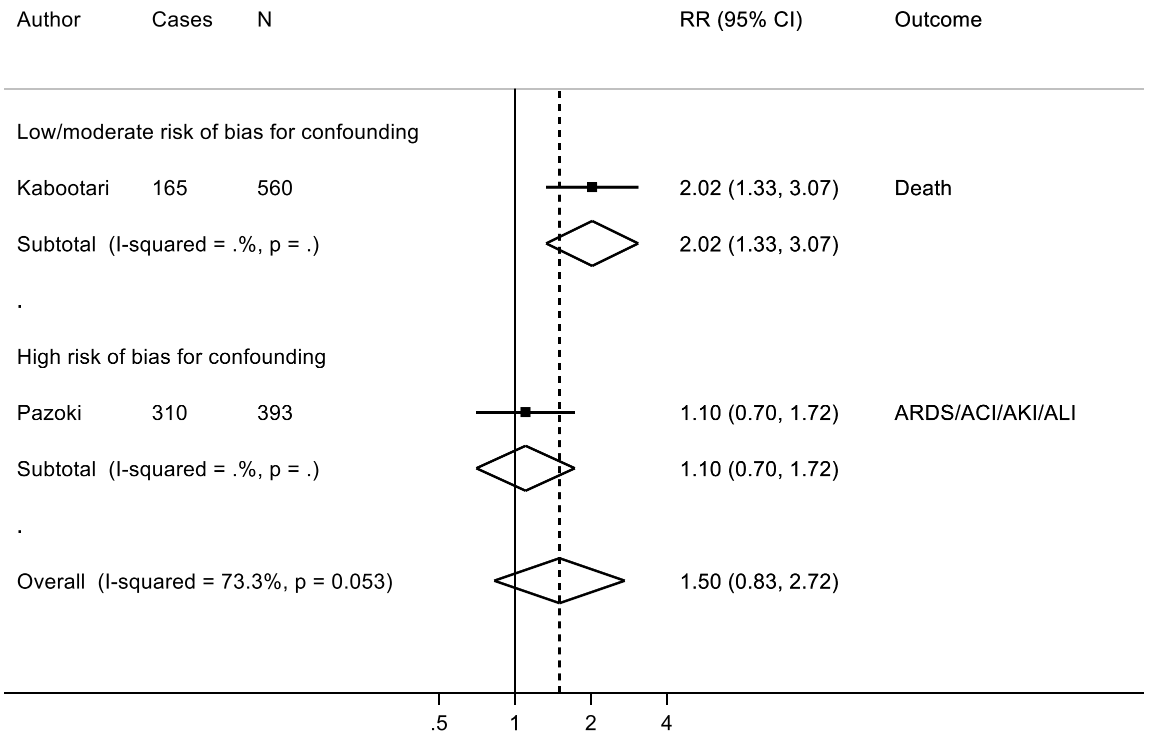

ESM Figure 85: Meta-analysis on **potassium,** per 1 mmol/l and A) death and B) severity of COVID-19 in patients with diabetes and COVID-19

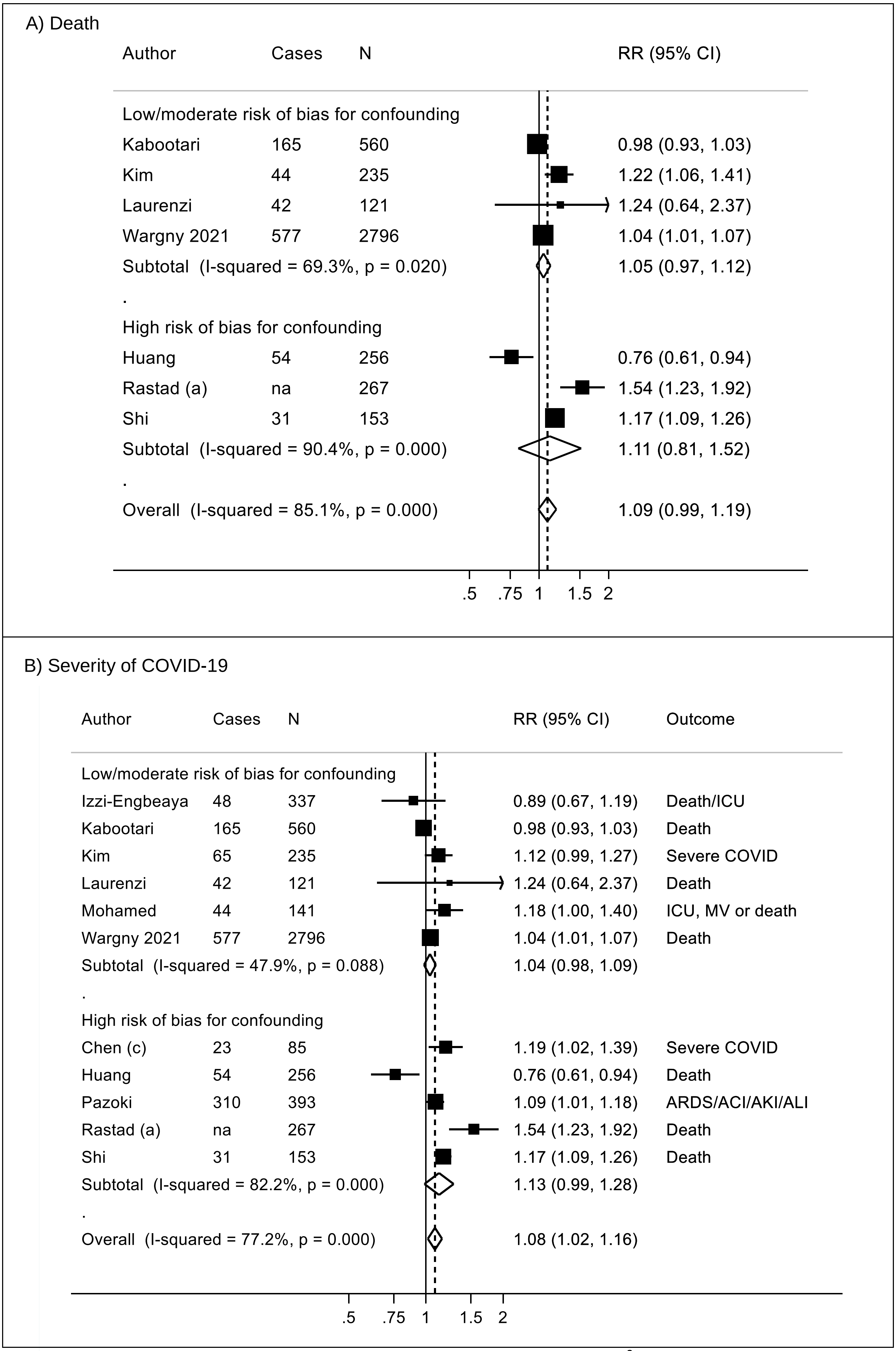

ESM Figure 86: Meta-analysis on **white blood cell count**, per 1x10^9/l and A) death and B) severity of COVID-19 in patients with diabetes and COVID-19

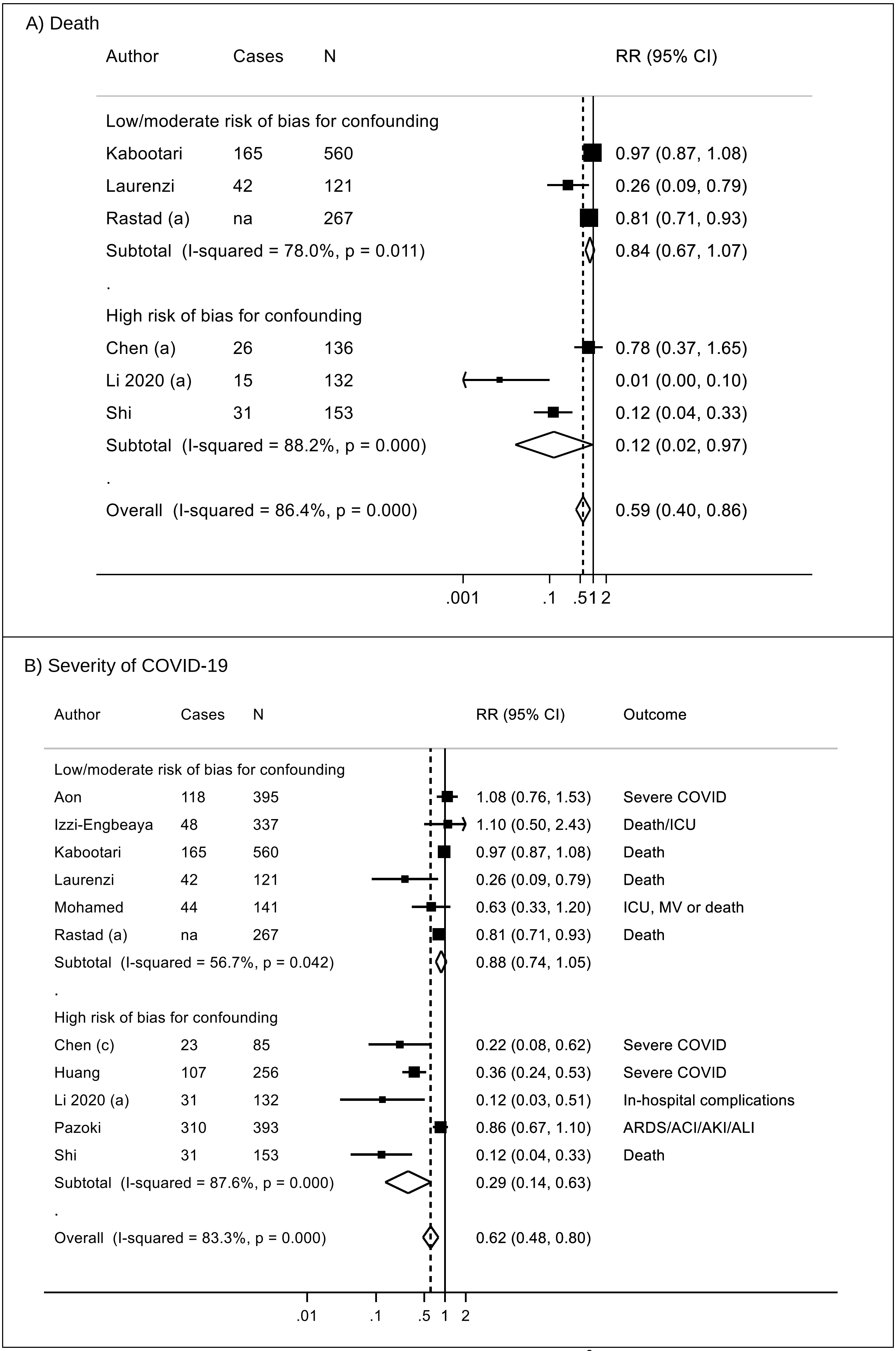

ESM Figure 87: Meta-analysis on **lymphocyte count**, per 1x10^{9}/l and A) death and B) severity of COVID-19in patients with diabetes and COVID-19

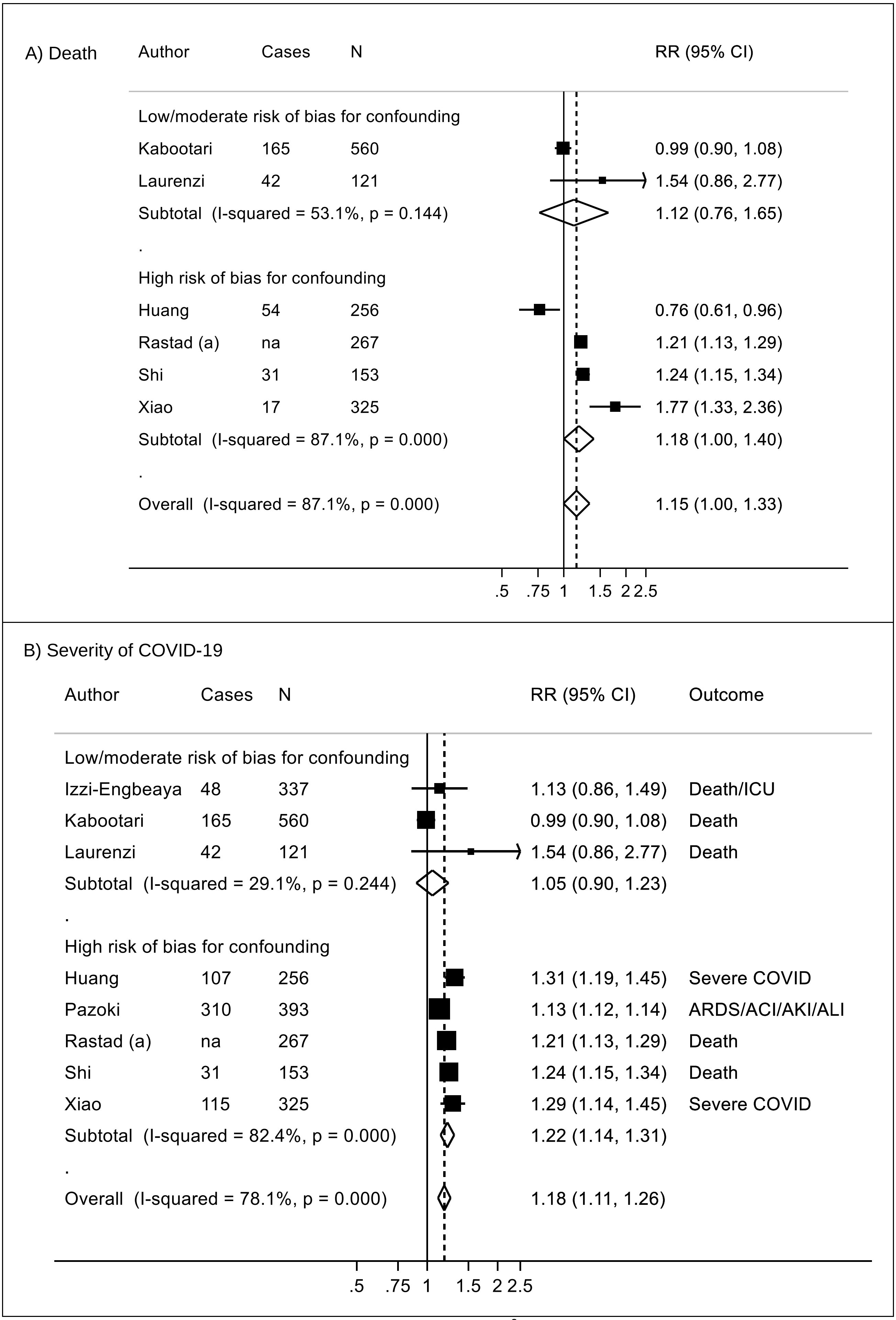

ESM Figure 88: Meta-analysis on **neutrophils**, per 1x10^9/l and A) death and B) severity of COVID-19 in patients with diabetes and COVID-19

A) Death

no data

B) Severity of COVID-19

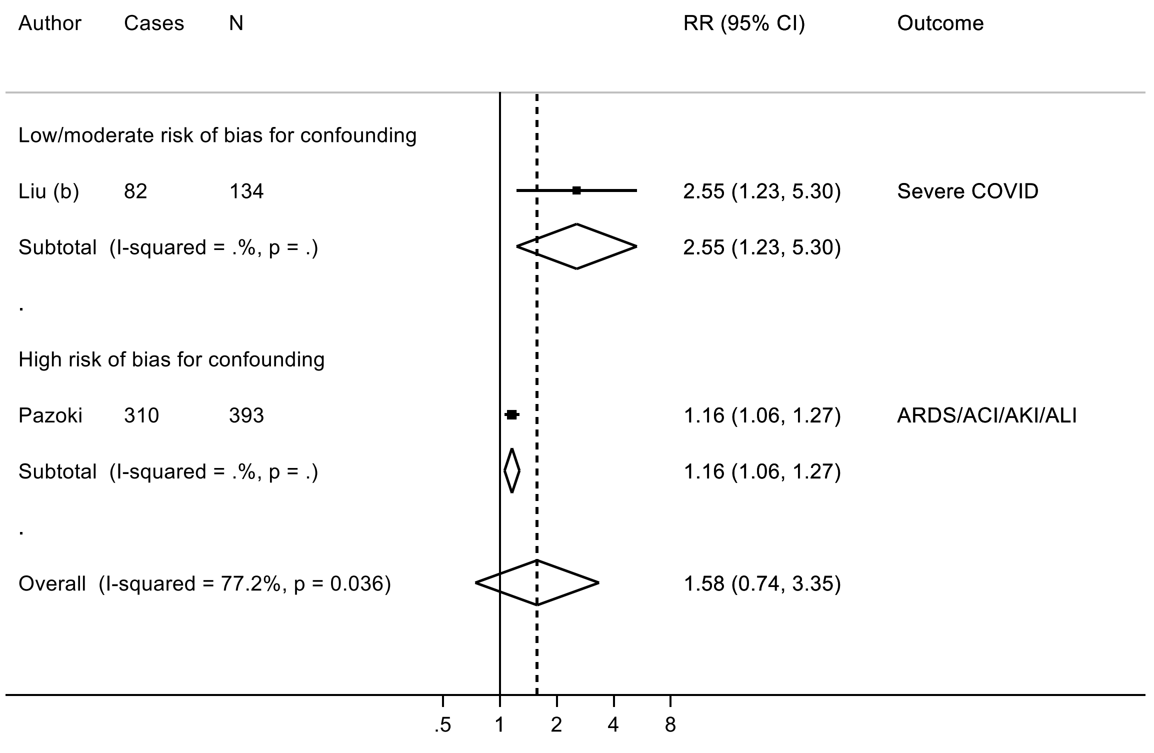

ESM Figure 89: Meta-analysis on **neutrophil-to-lymphocyte ratio** and A) death and B) severity of COVID-19 in patients with diabetes and COVID-19

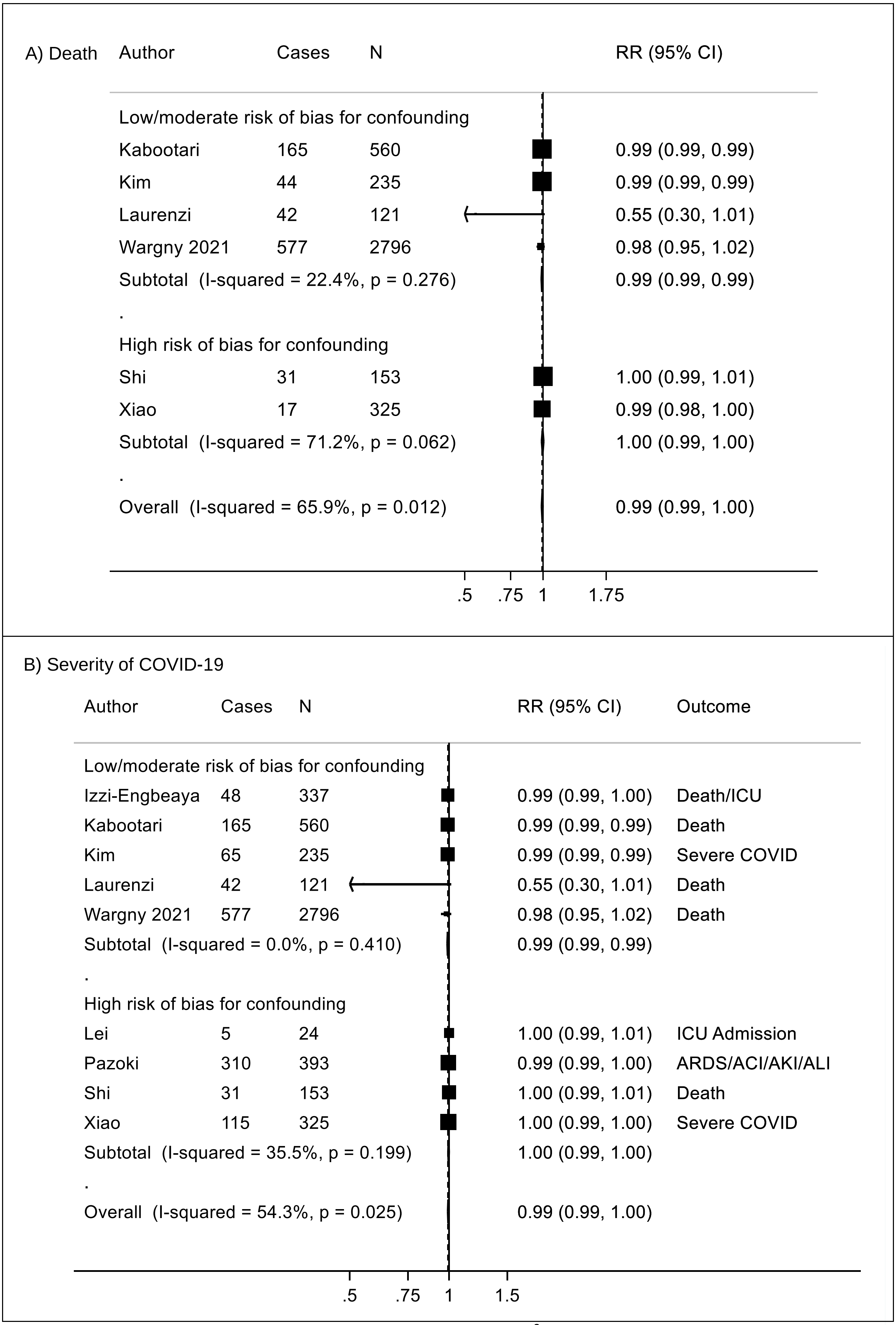

ESM Figure 90: Meta-analysis on **platelet count**, per 1x10^9/l and A) death and B) severity of COVID-19 in patients with diabetes and COVID-19

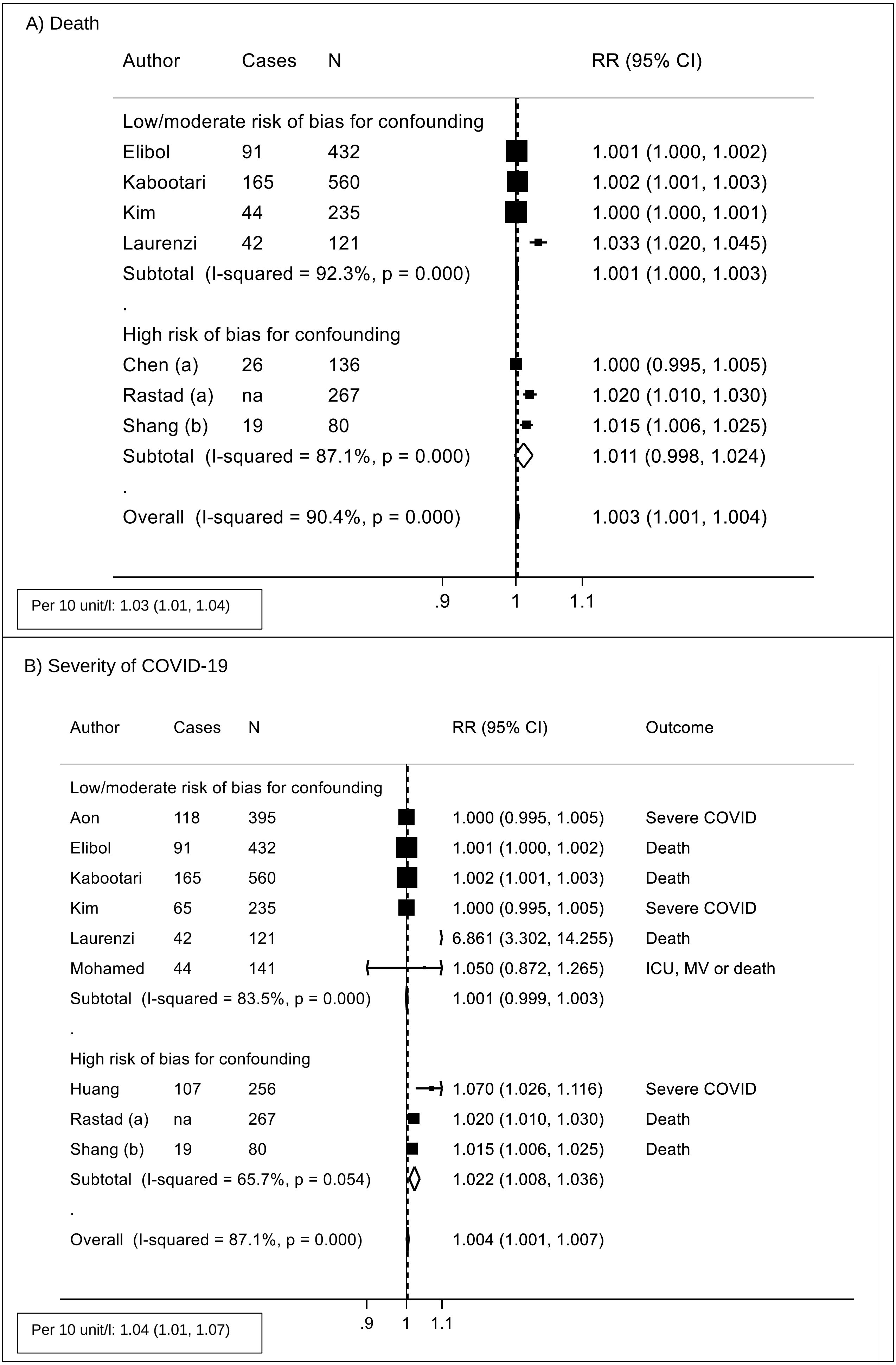

ESM Figure 91: Meta-analysis on **lactate dehydrogenase**, per 1 unit/l and A) death and B) severity of COVID-19 in patients with diabetes and COVID-19

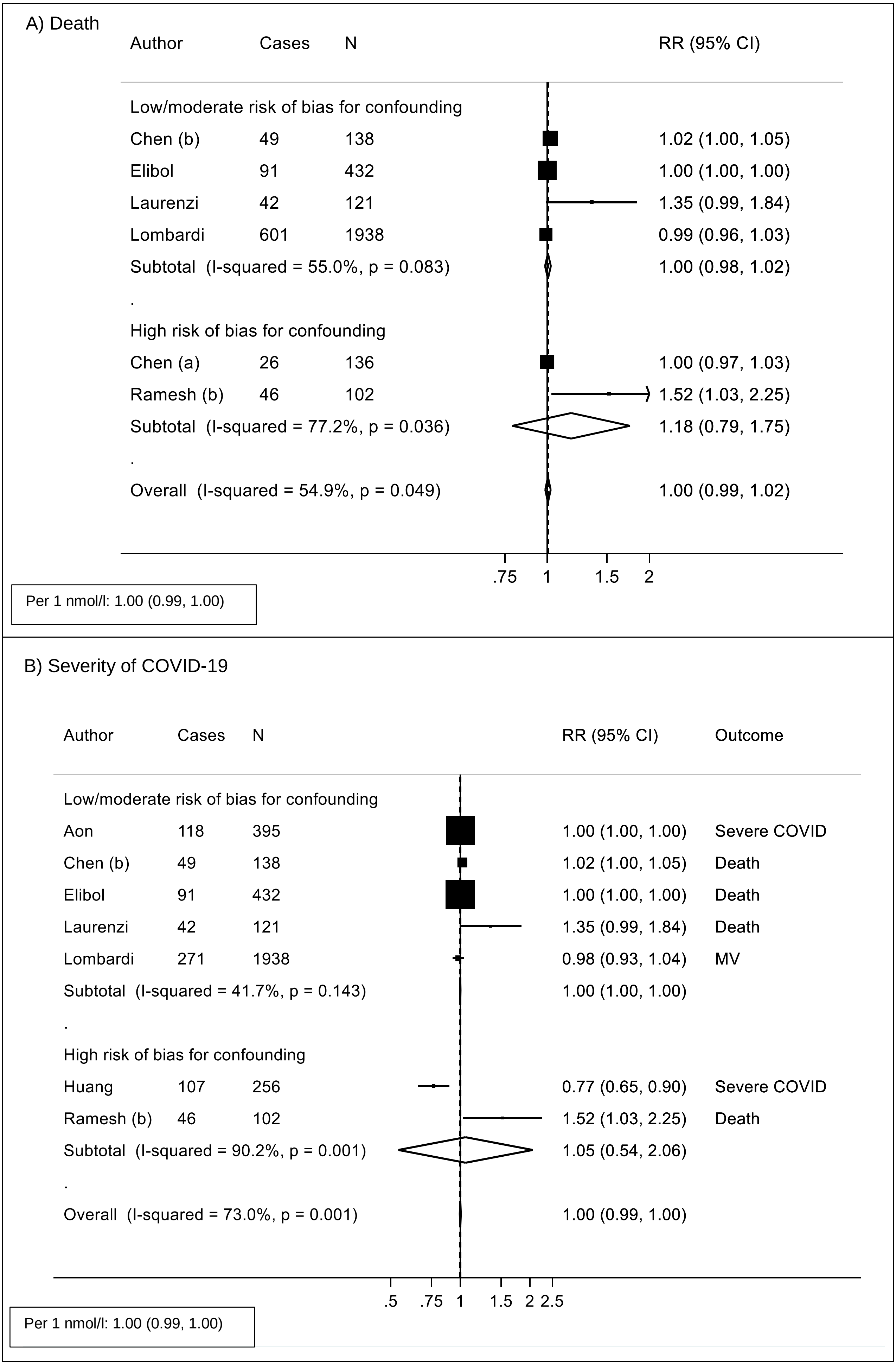

ESM Figure 92: Meta-analysis on **d-dimer**, per 1 mg/l and A) death and B) severity of COVID-19 in patients with diabetes and COVID-19

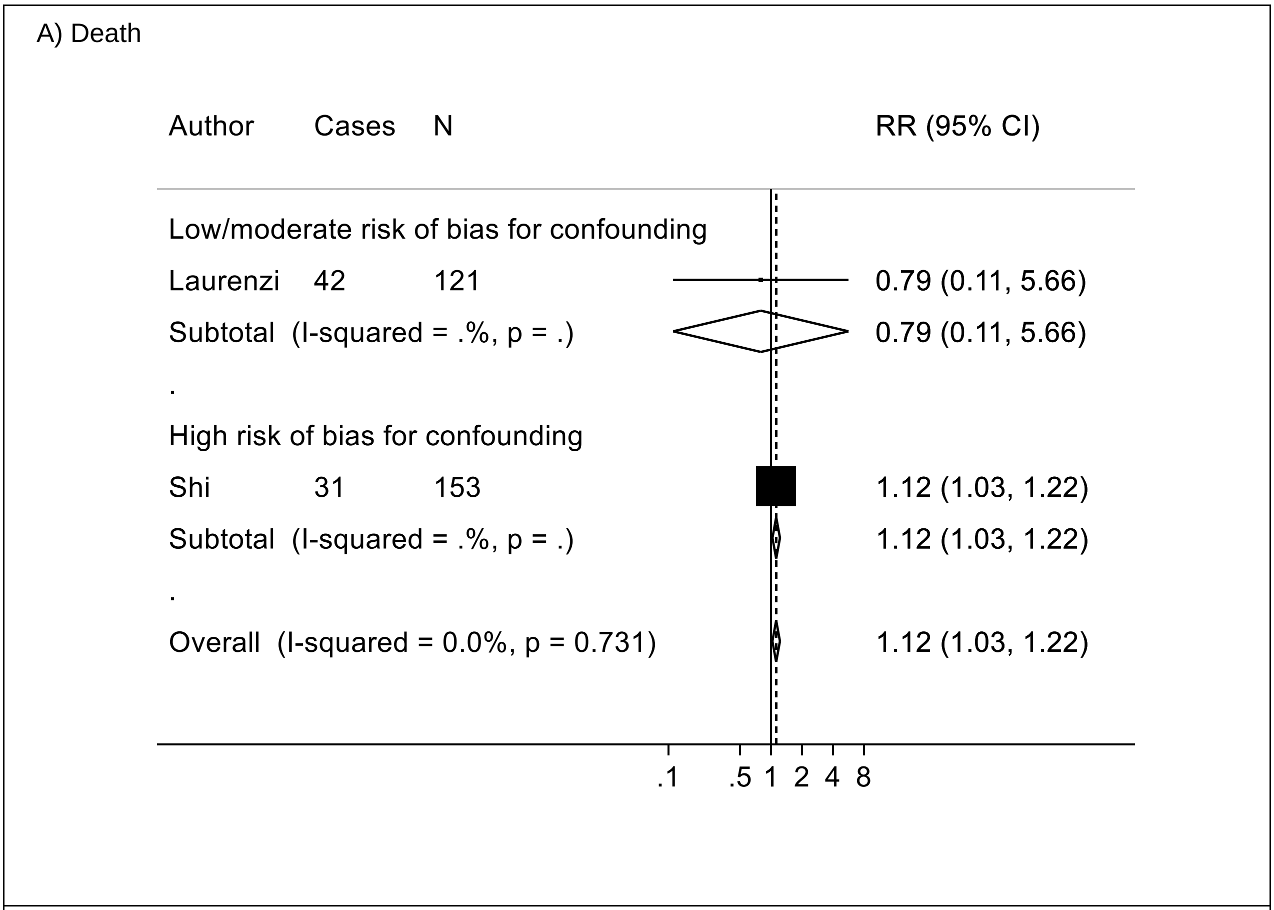

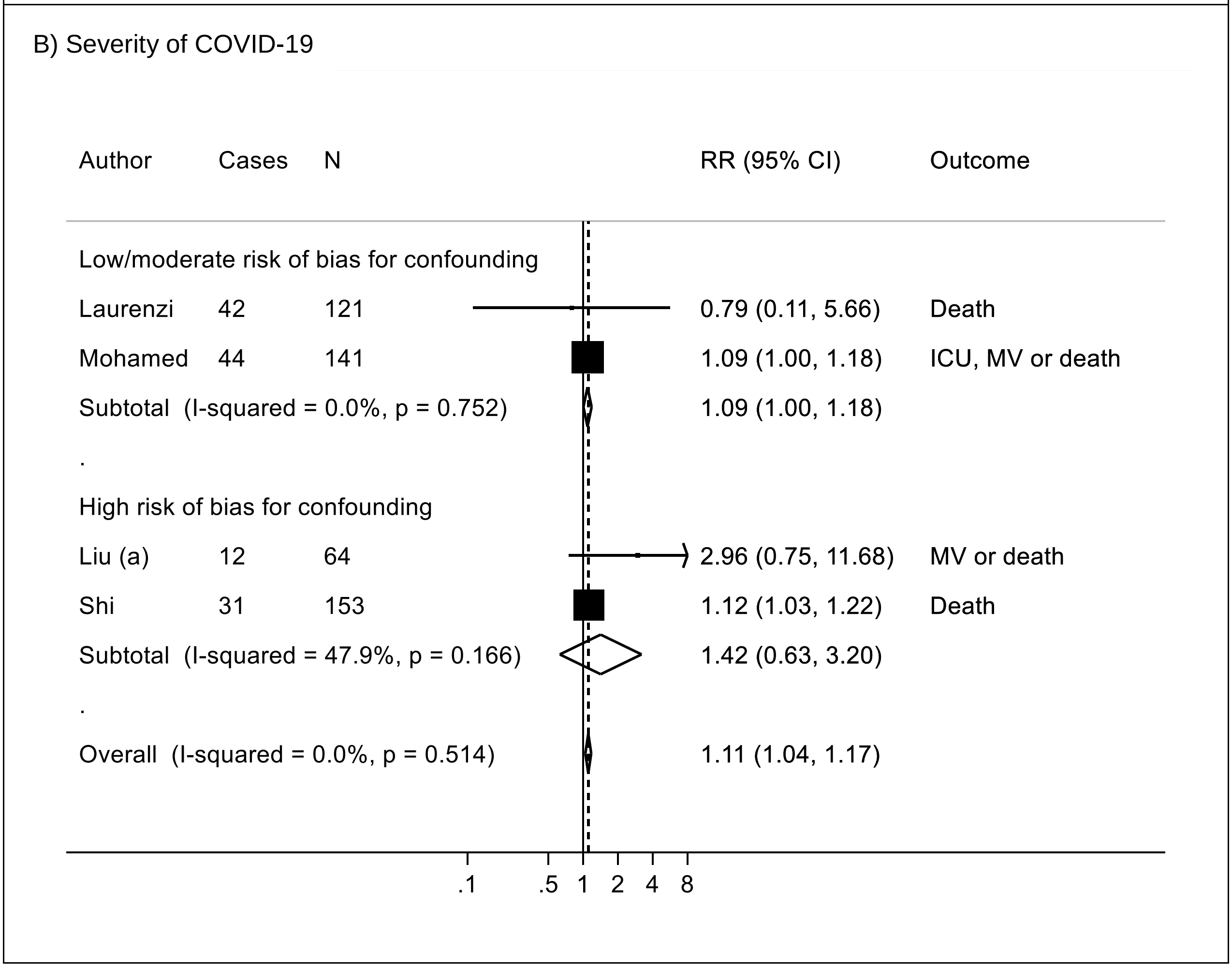

ESM Figure 93: Meta-analysis on **prothrombin time**, per 1 s and A) death and B) severity of COVID-19 in patients with diabetes and COVID-19

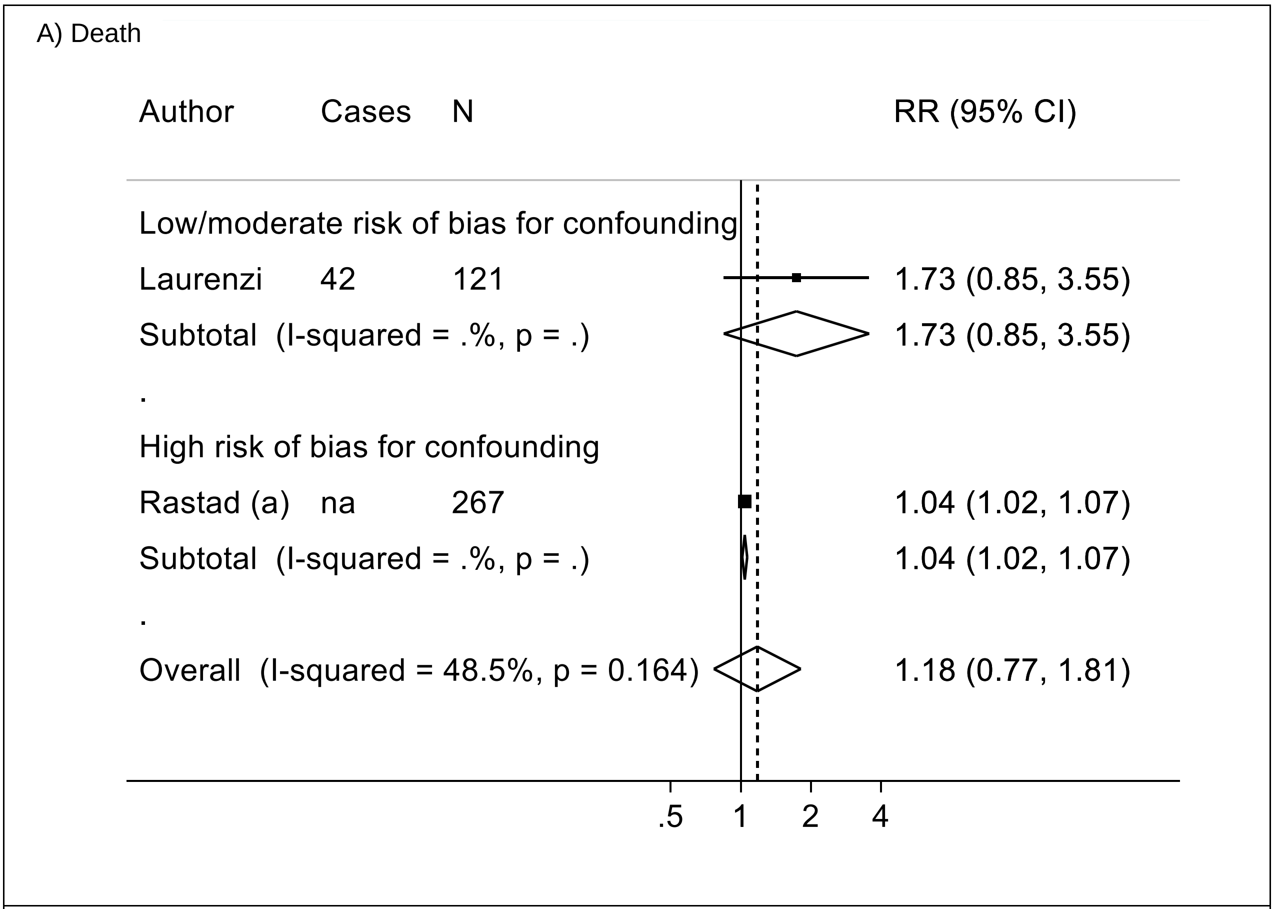

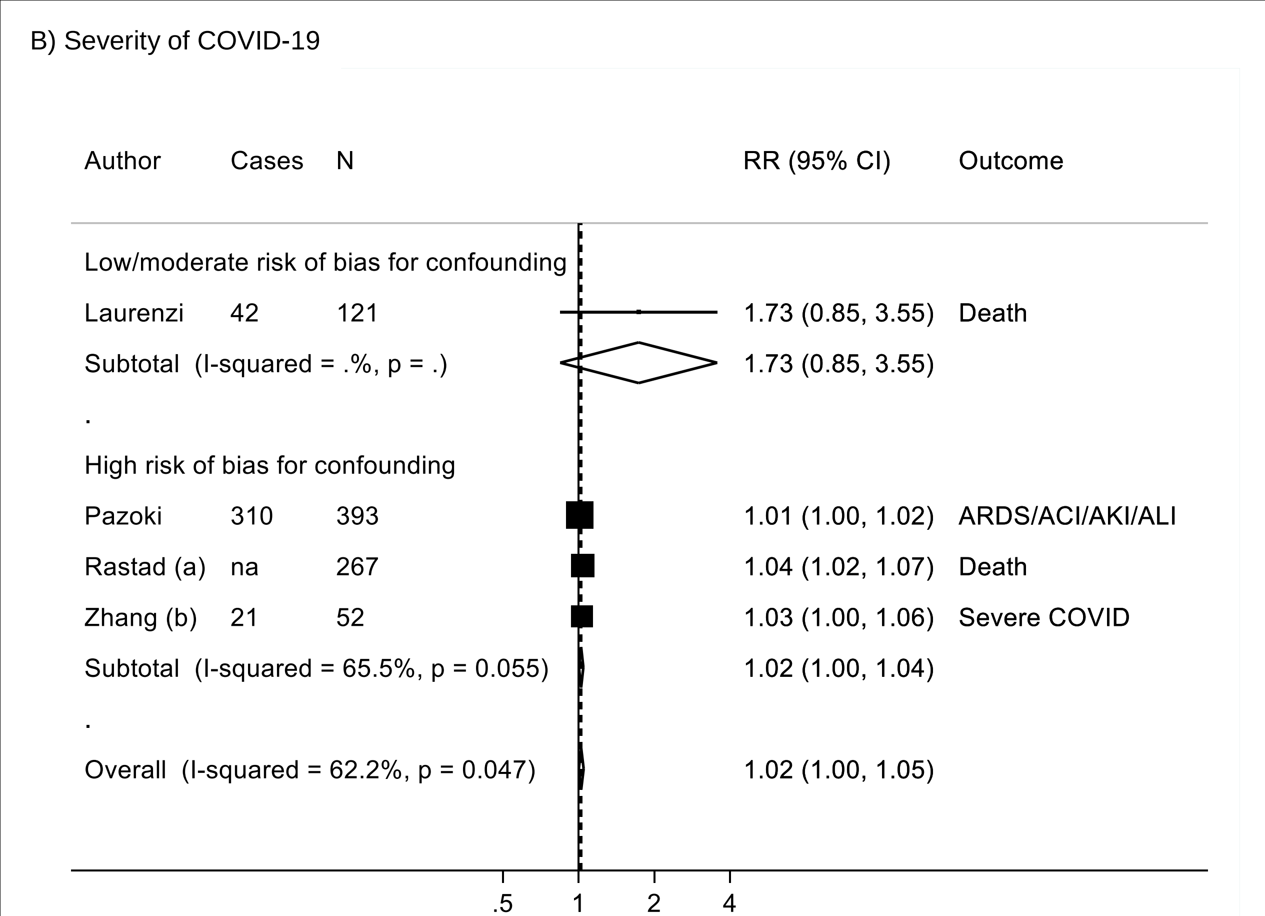

ESM Figure 94: Meta-analysis on **erythrocyte sedimentation rate**, per 1 mm/h and A) death and B) severity of COVID-19 in patients with diabetes and COVID-19

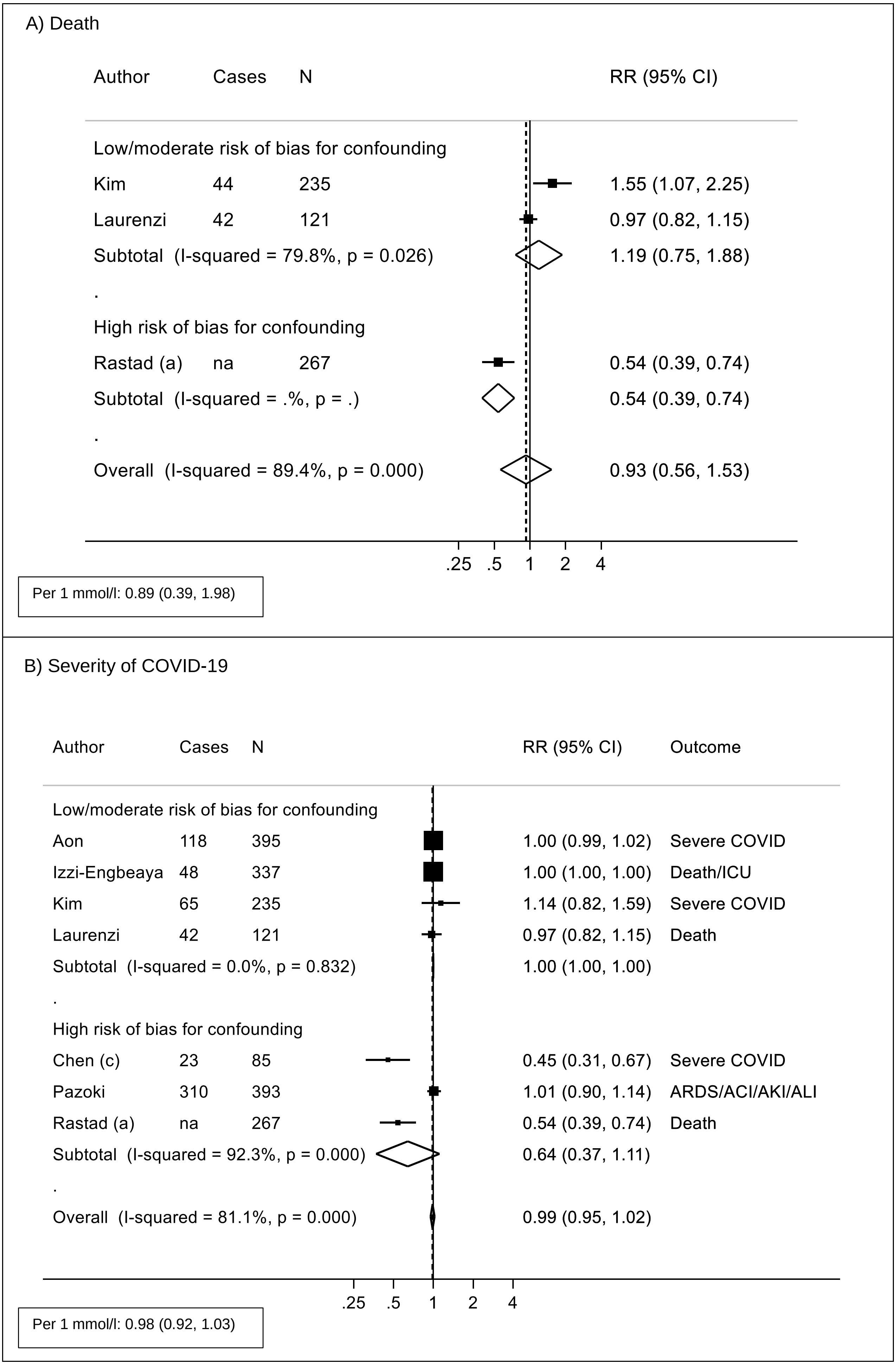

ESM Figure 95: Meta-analysis on **haemoglobin**, per 1 g/dl and A) death and B) severity of COVID-19 in patients with diabetes and COVID-19

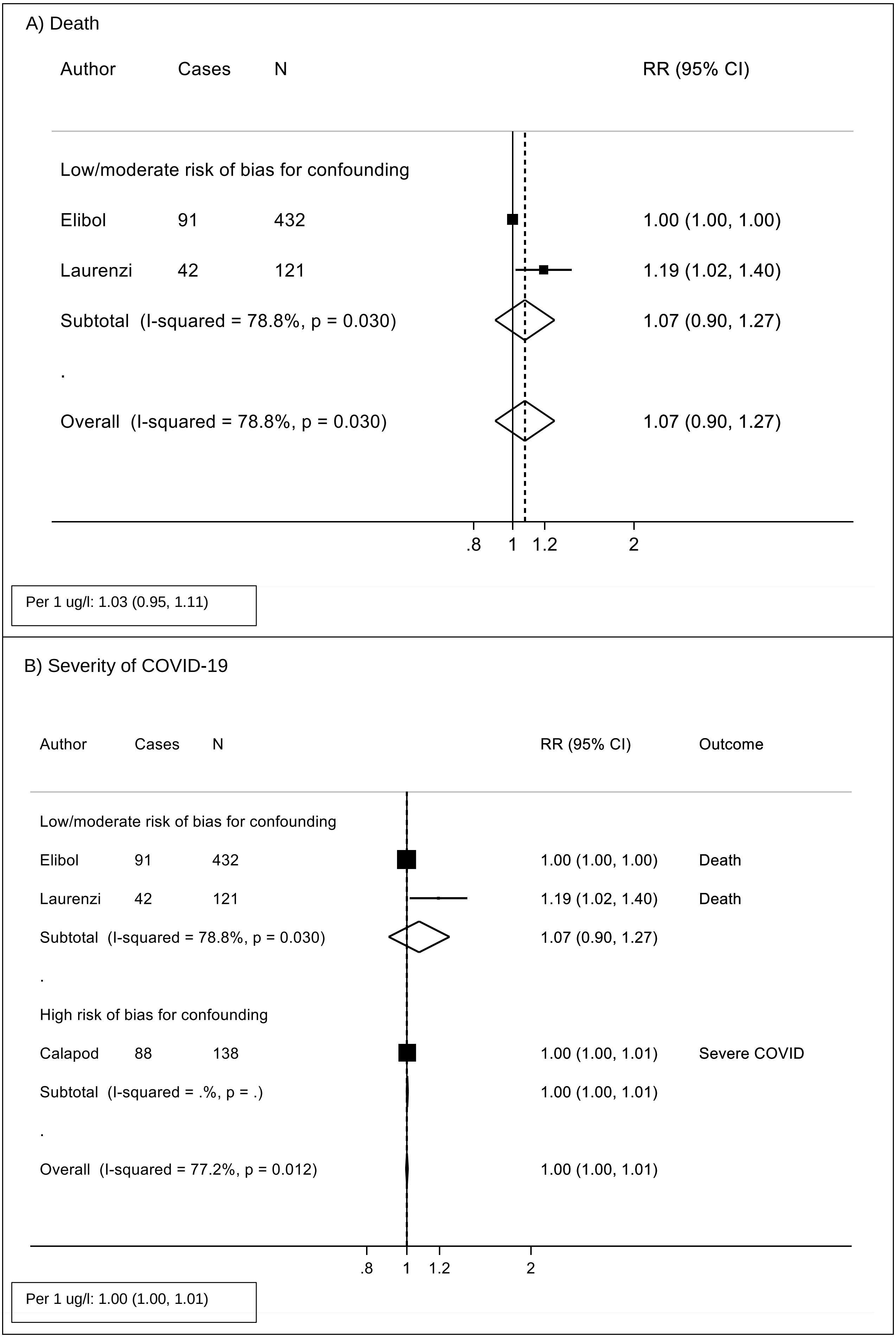

ESM Figure 96: Meta-analysis on **ferritin, per 1 pmol/L** and A) death and B) severity of COVID-19 in patients with diabetes and COVID-19

A) Death

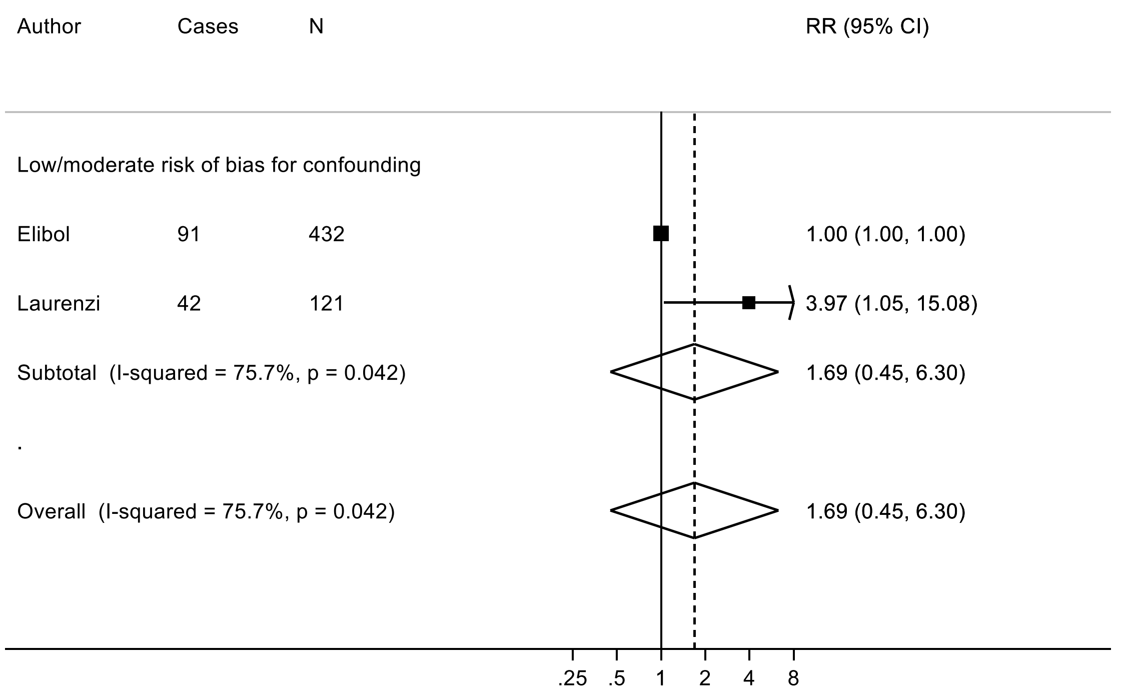

B) Severity of COVID-19

No data

ESM Figure 97: Meta-analysis on **fibrinogen**, per 1 g/l and A) death and B) severity of COVID-19 in patients with diabetes and COVID-19

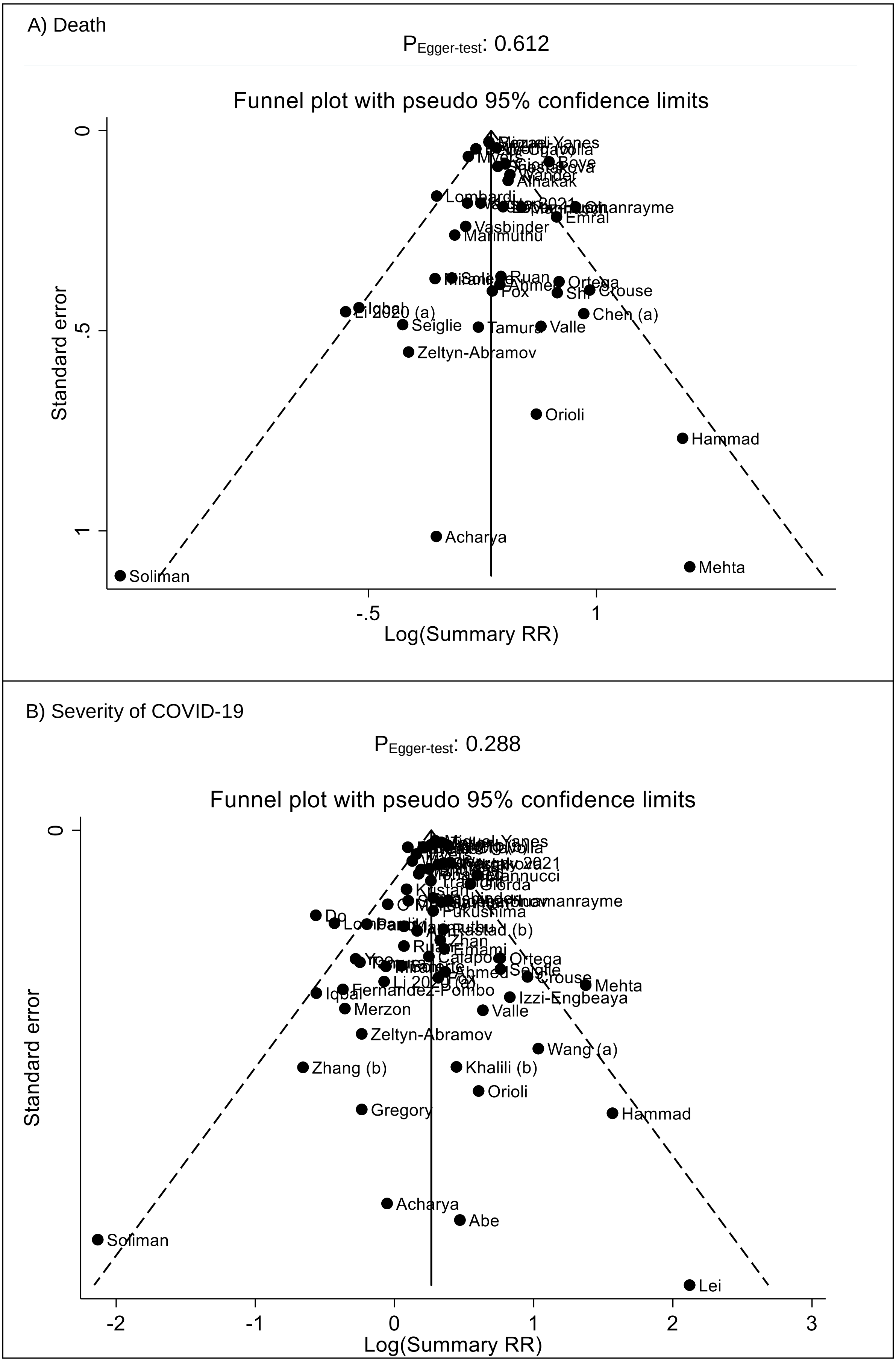

ESM Figure 98: Funnel plot for association for **men versus women** and A) death and B) severity of COVID-19 in patients with diabetes and COVID -19

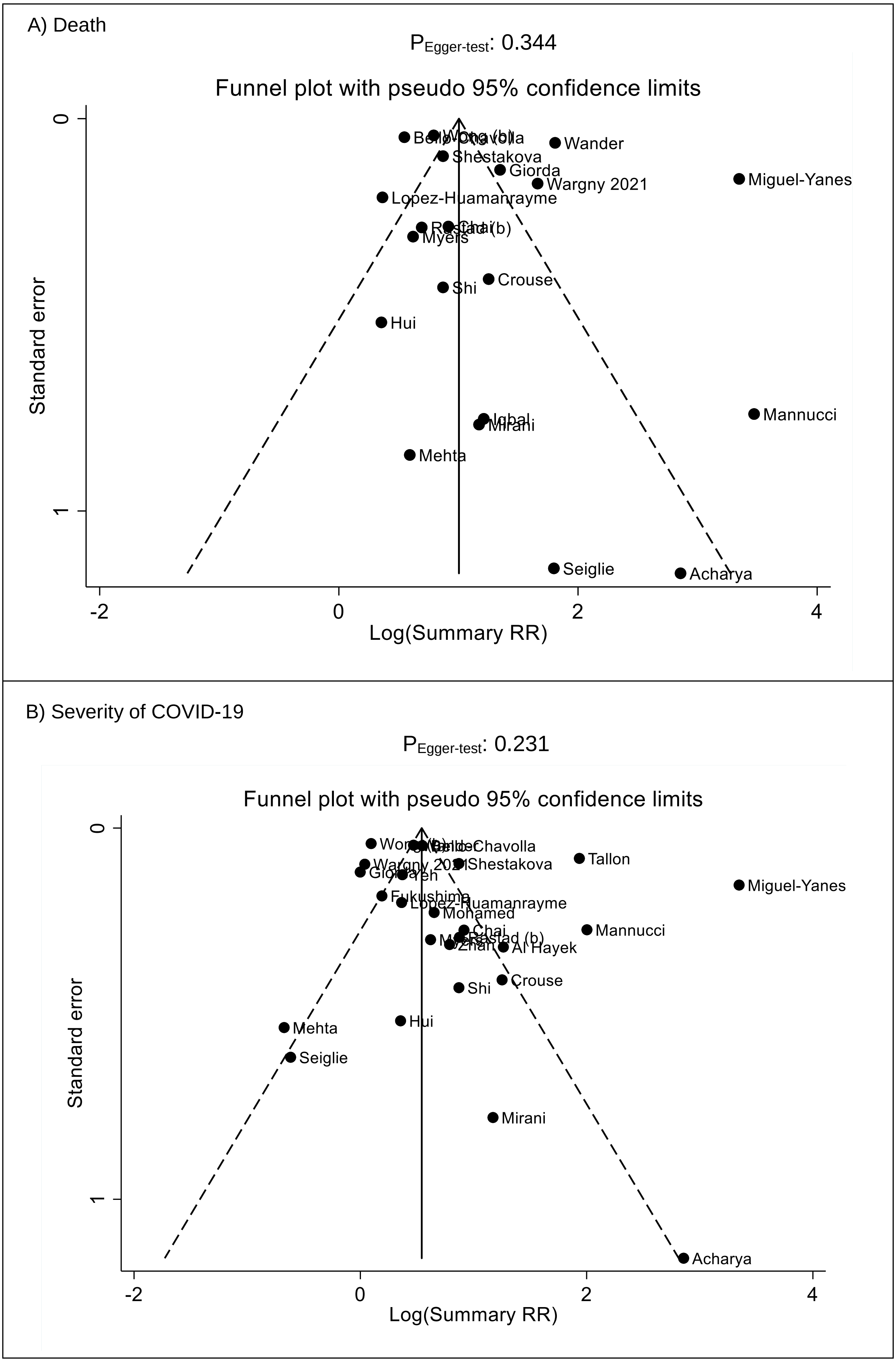

ESM Figure 99: Funnel plot for association between **age ≥65 years** and A) death and B) severity of COVID-19 in patients with diabetes and COVID -19

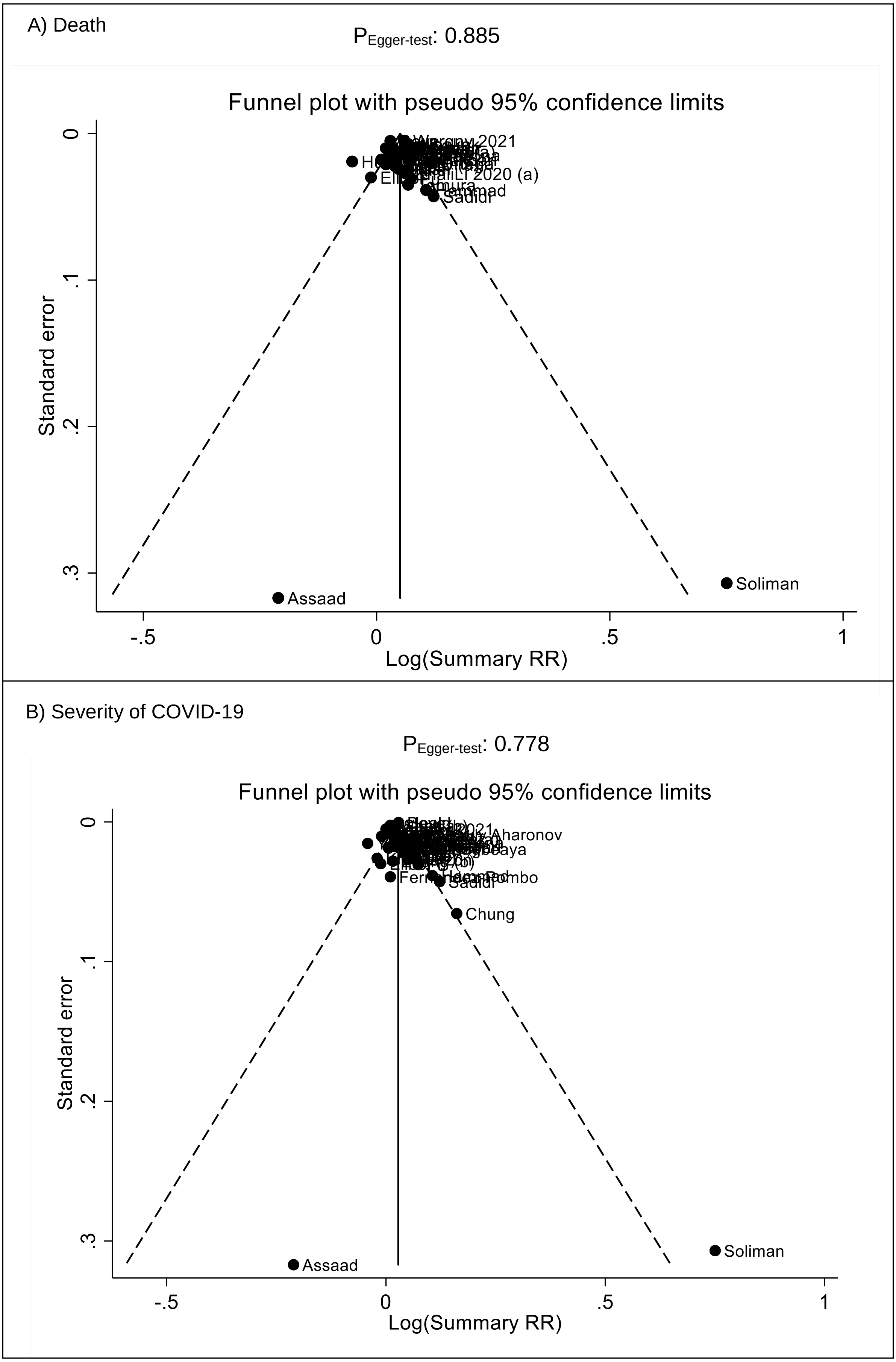

ESM Figure 100: Funnel plot for association between **age per 5 year** increase and A) death and B) severity of COVID-19 in patients with diabetes and COVID-19

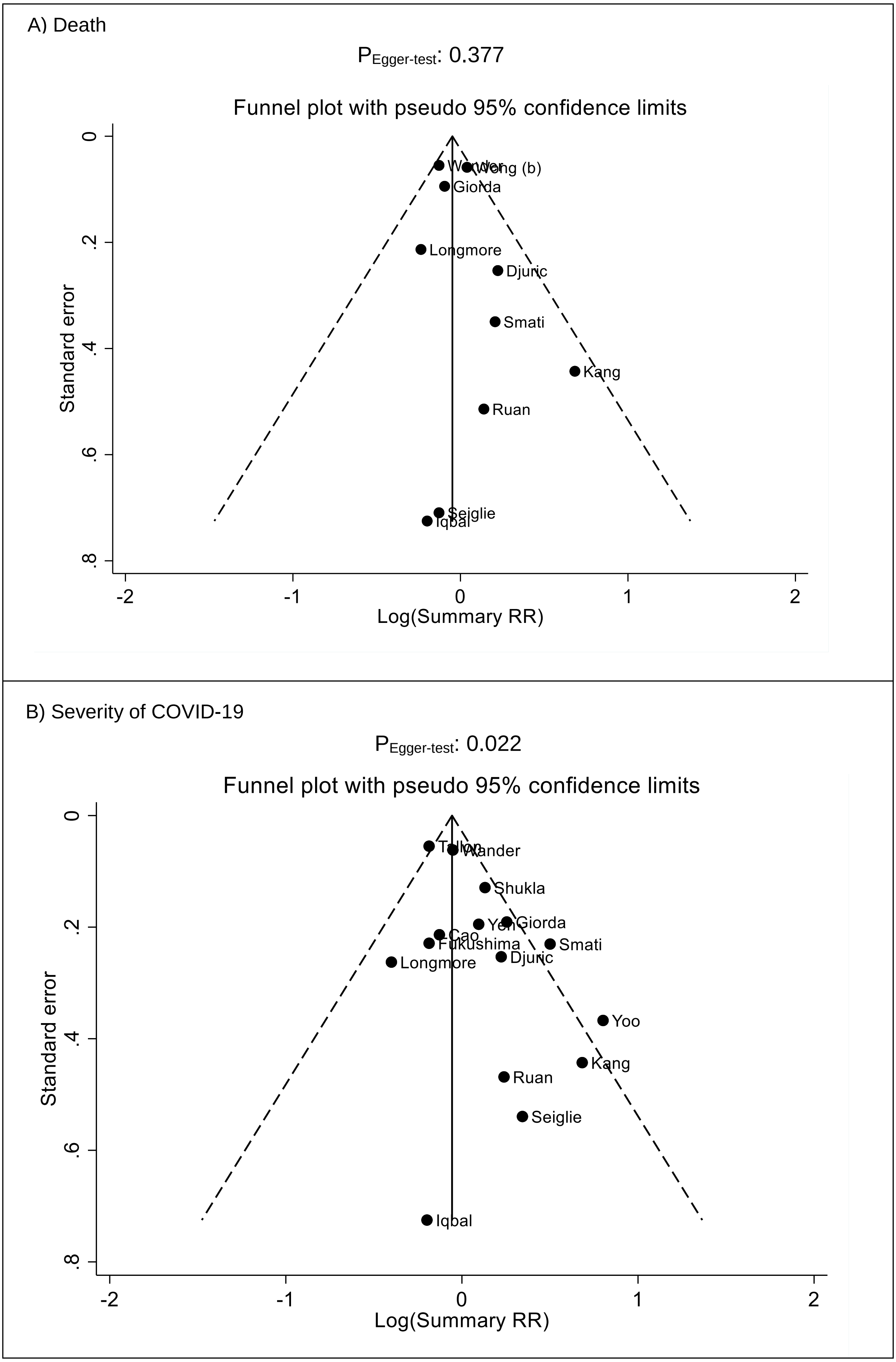

ESM Figure 101: Funnel plot for association between **overweight** and A) death and B) severity of COVID-19 in patients with diabetes and COVID-19

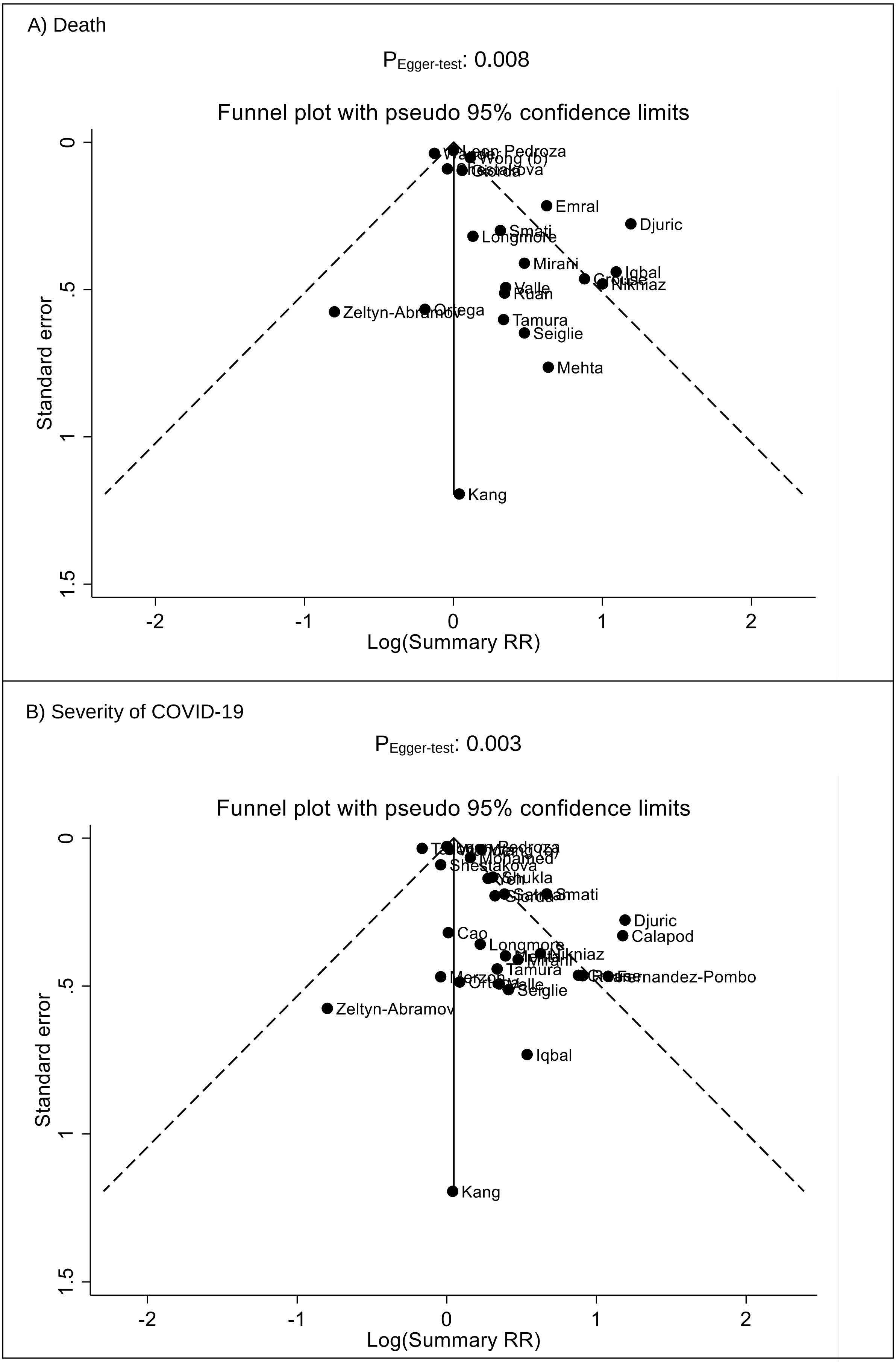

ESM Figure 102: Funnel plot for association between **obesity** and A) death and B) severity of COVID-19 in patients with diabetes and COVID-19

A) Death

$P_{Egger\text{-}test}$: 0. 870

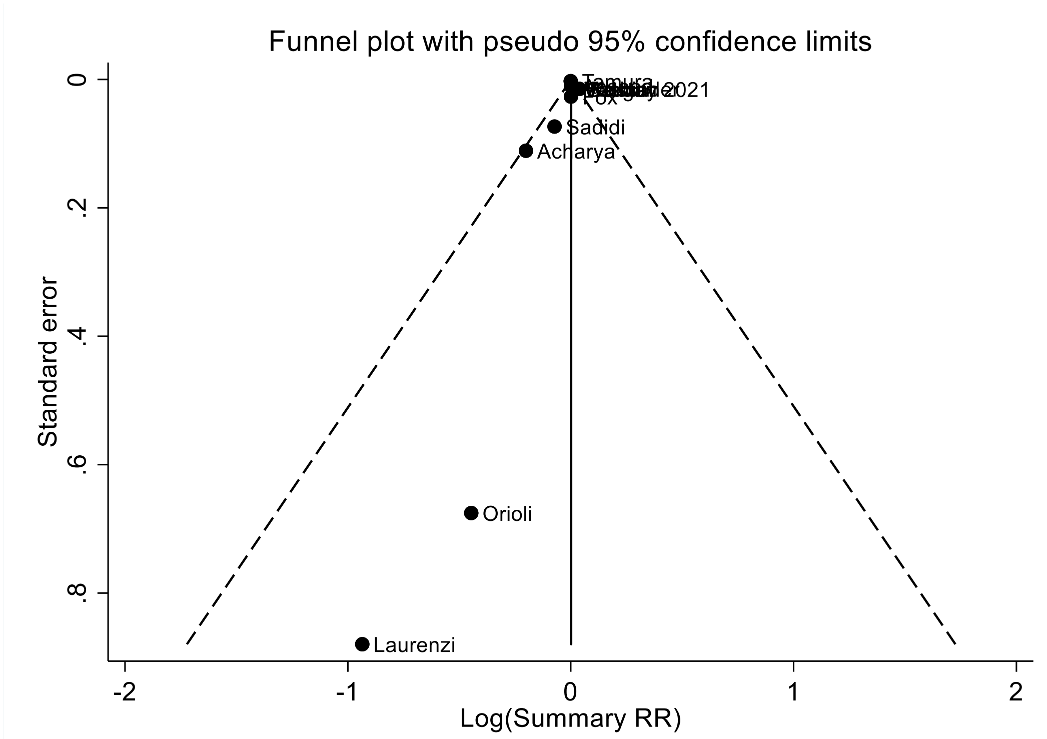

B) Severity of COVID-19

$P_{Egger\text{-}test}$: 0.355

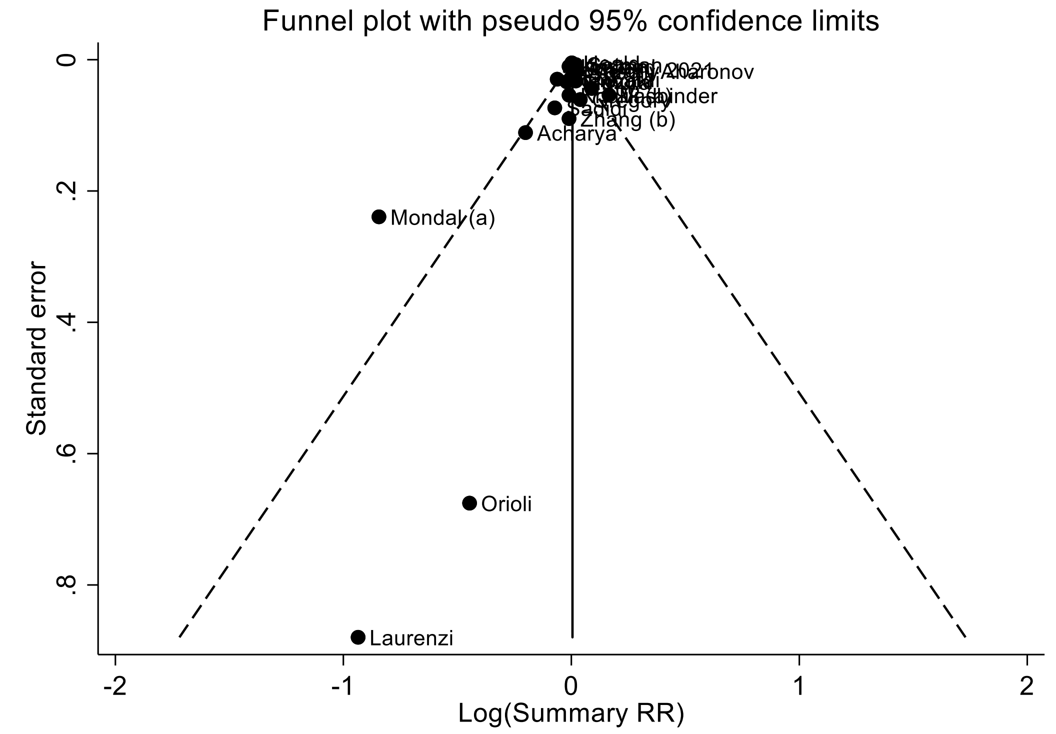

ESM Figure 103: Funnel plot for association between **BMI per 1 kg/m²** and A) death and B) severity of COVID-19 in patients with diabetes and COVID-19

A) Death

$P_{Egger\text{-}test}$: 0.963

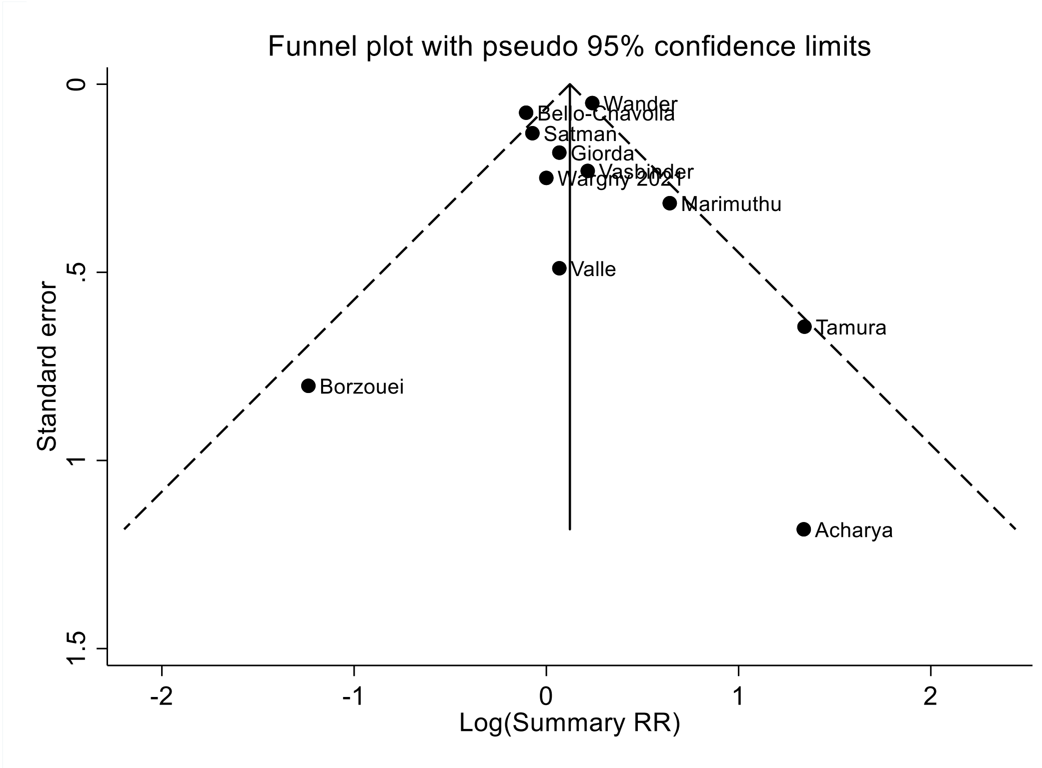

B) Severity of COVID-19

$P_{Egger\text{-}test}$: 0.533

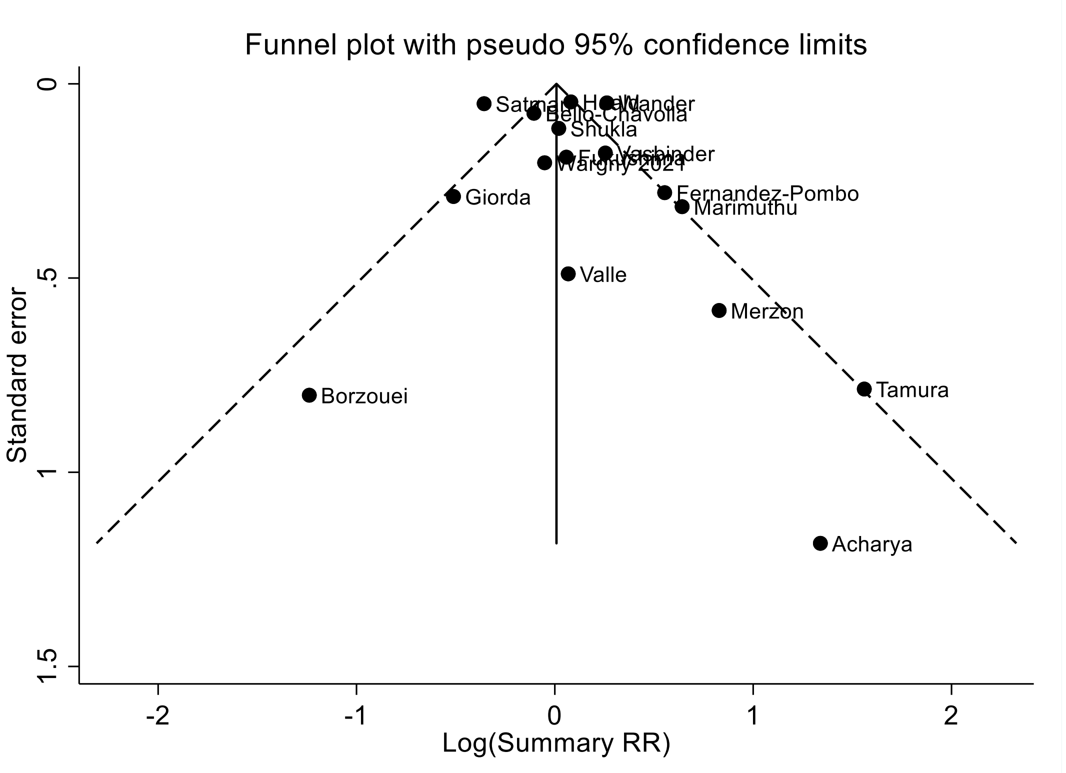

ESM Figure 104: Funnel plot for association between **smoking** and A) death and B) severity of COVID-19 in patients with diabetes and COVID-19

A) Death

No data

B) Severity of COVID-19

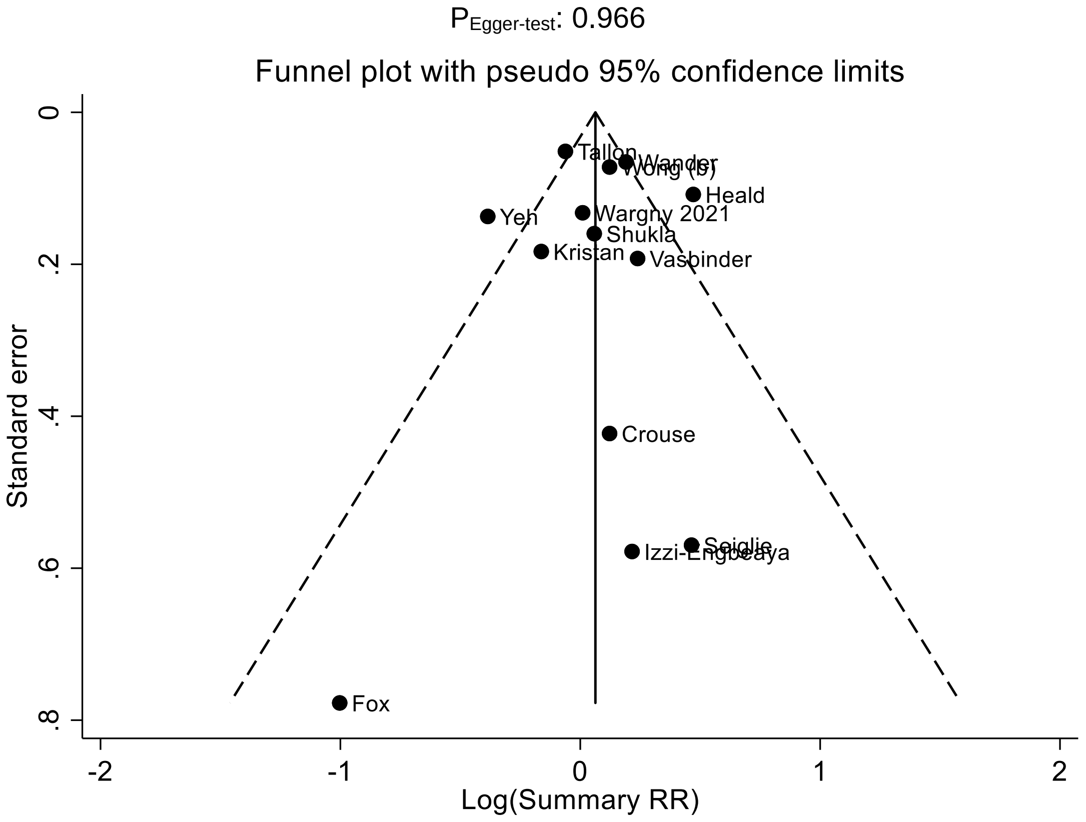

ESM Figure 105: Funnel plot for association between **ethnicity (Black vs. Non-Hispanic white)** and A) death and B) severity of COVID-19 in patients with diabetes and COVID-19

A) Death

No data

B) Severity of COVID-19

$P_{Egger\text{-}test}$: 0.159

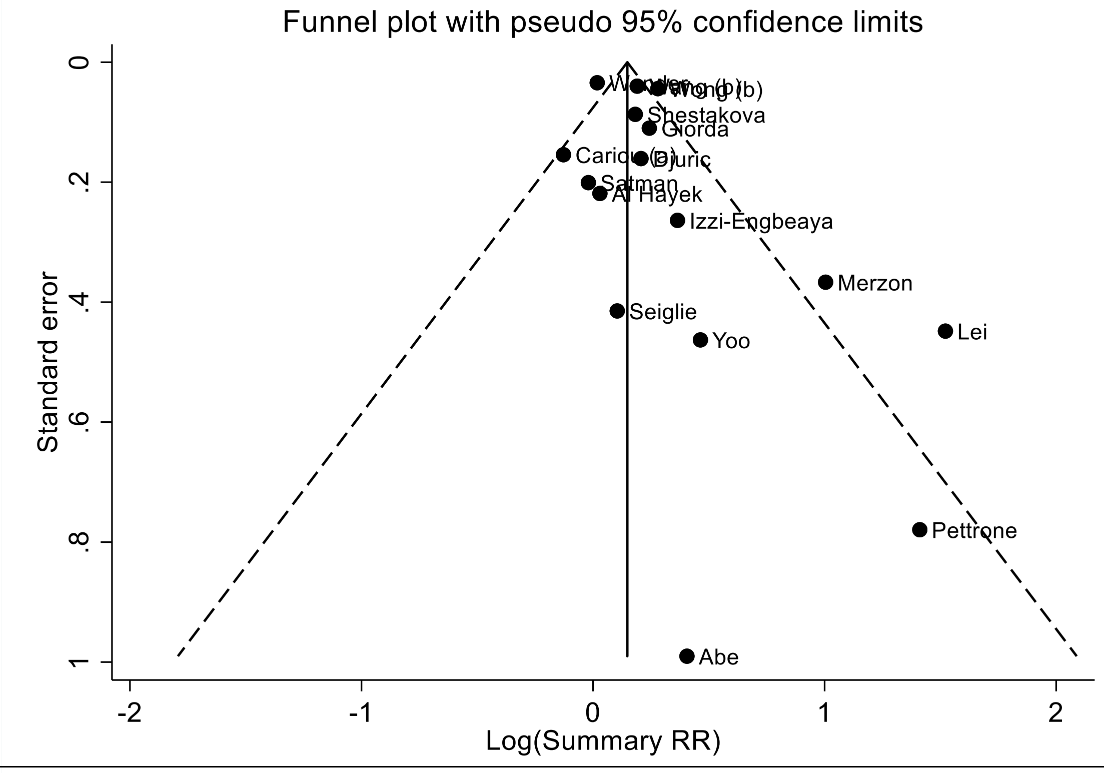

ESM Figure 106: Funnel plot for association between **HbA_{1c} 53-75 vs <53 mmol/mol (7-9 vs <7%)** and A) death and B) severity of COVID-19 in patients with diabetes and COVID-19

A) Death

No data

B) Severity of COVID-19

$P_{Egger\text{-}test}$: 0.411

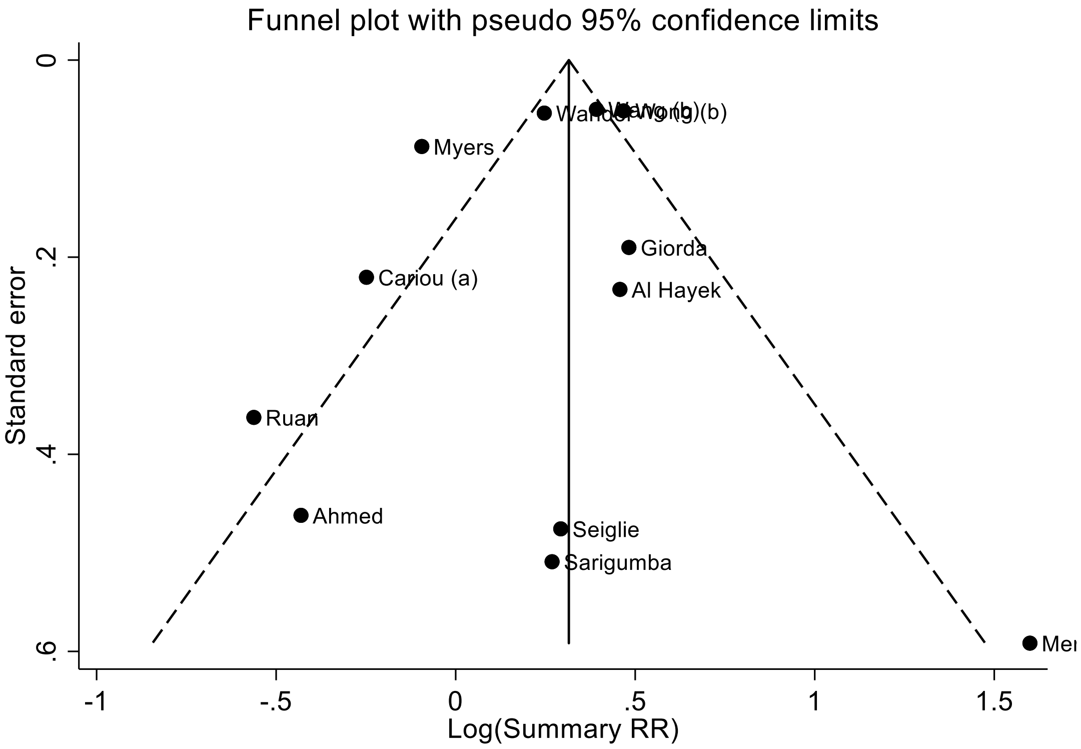

ESM Figure 107: Funnel plot for association between **HbA_{1c} ≥75 vs <53 mmol/mol (≥9 vs <7%)** and A) death and B) severity of COVID-19 in patients with diabetes and COVID-19

A) Death

$P_{Egger\text{-}test}$: 0.167

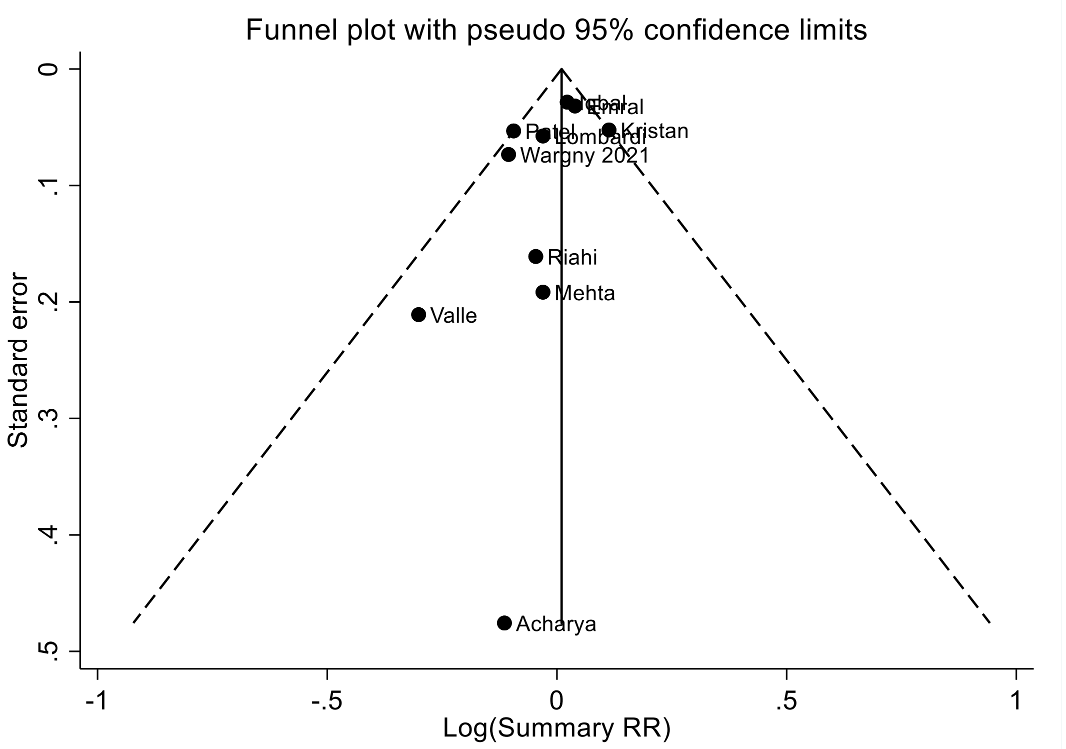

B) Severity of COVID-19

$P_{Egger\text{-}test}$: 0.095

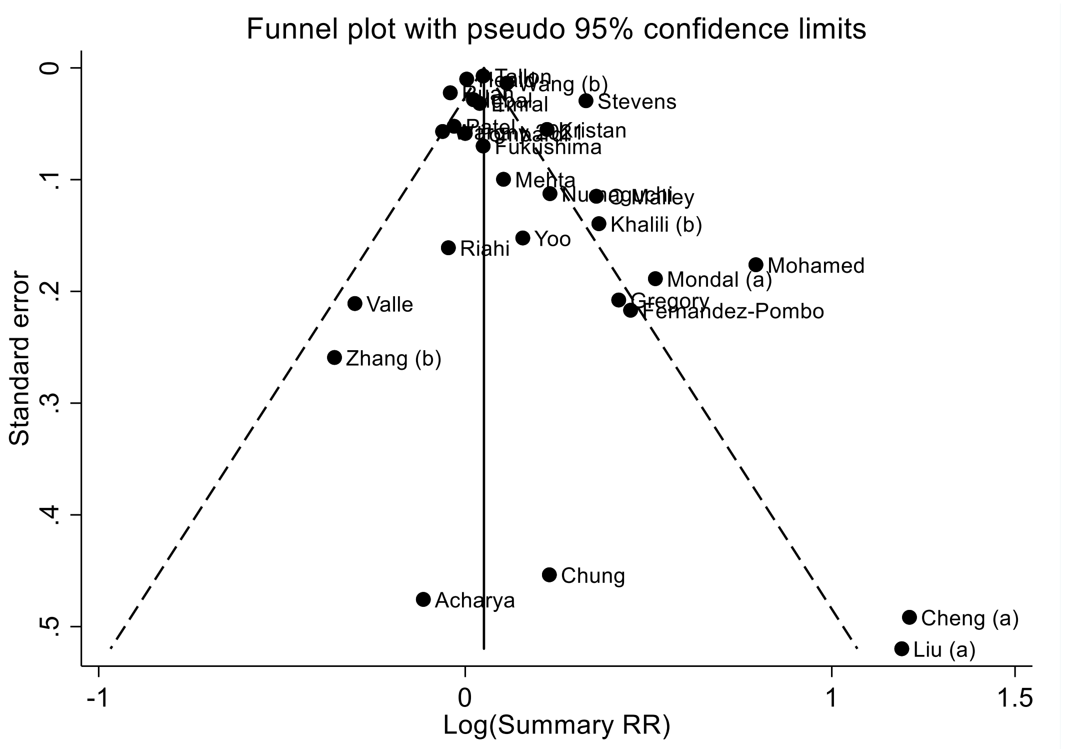

ESM Figure 108: Funnel plot for association between **HbA_{1c} per 1% increase** and A) death and B) severity of COVID-19 in patients with diabetes and COVID-19

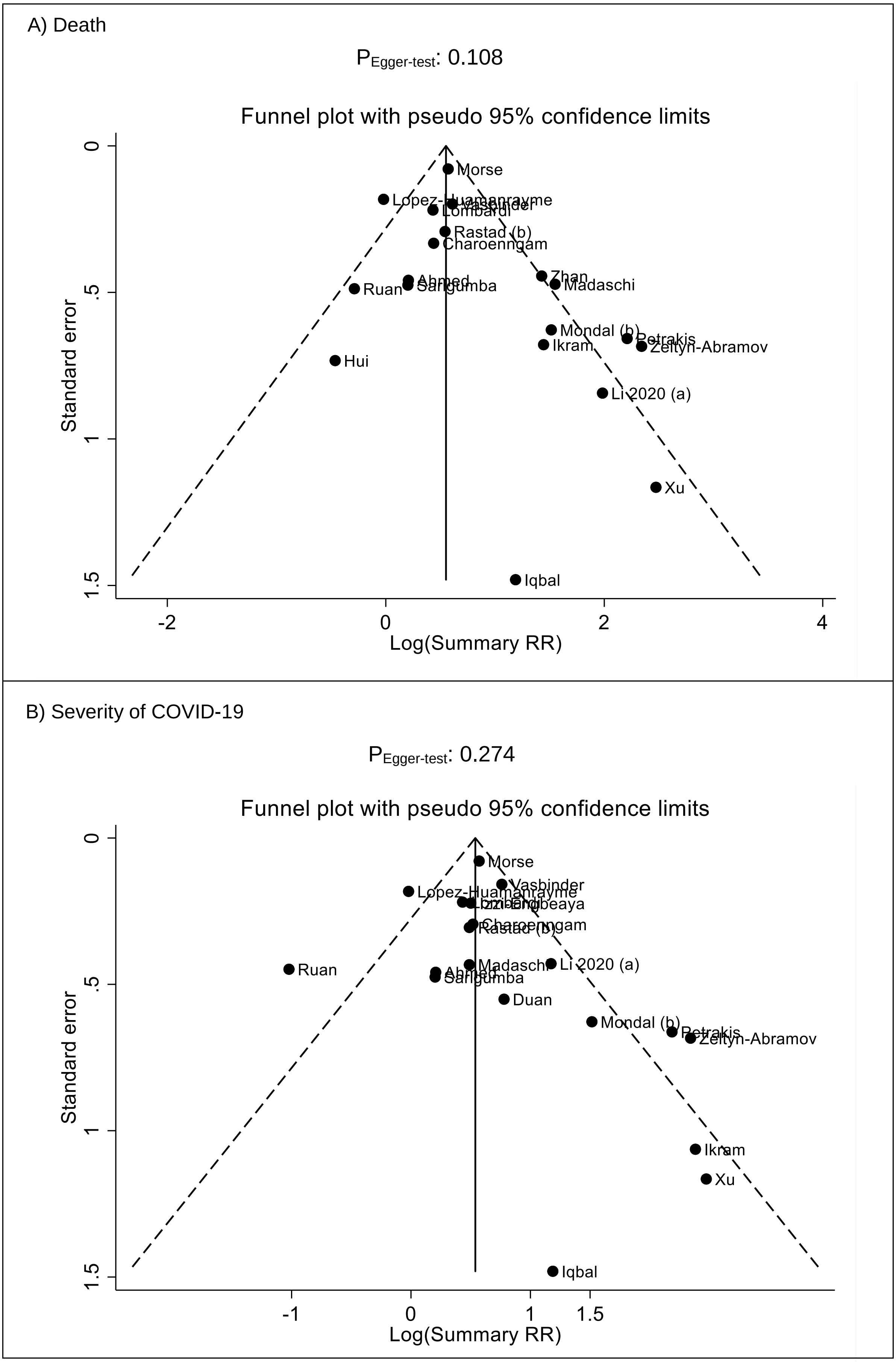

ESM Figure 109: Funnel plot for association between **glucose ≥10 mmol/l** and A) death and B) severity of COVID-19 in patients with diabetes and COVID-19

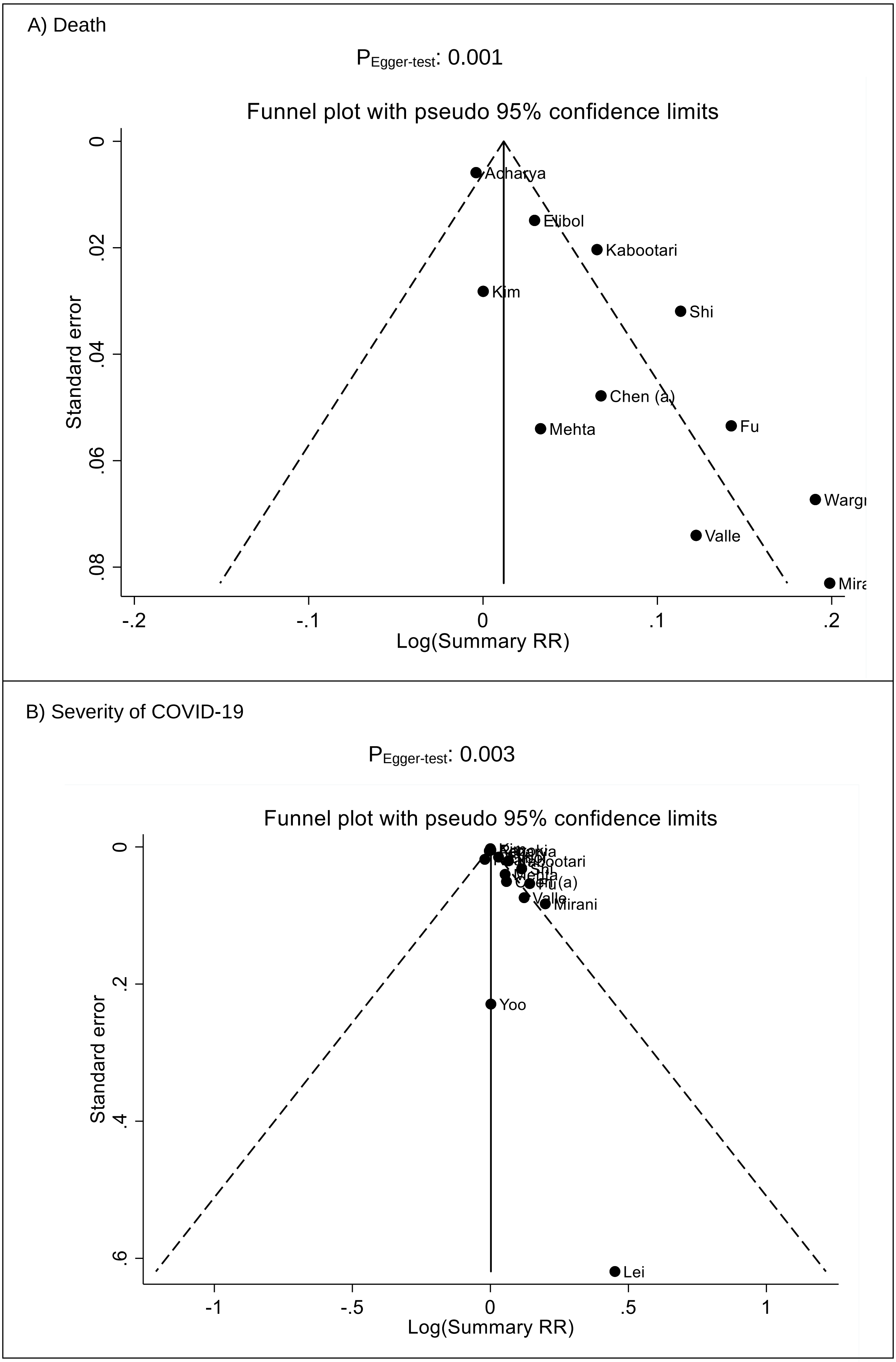

ESM Figure 110: Funnel plot for association between **glucose per 1 mmol/l** and A) death and B) severity of COVID-19 in patients with diabetes and COVID -19

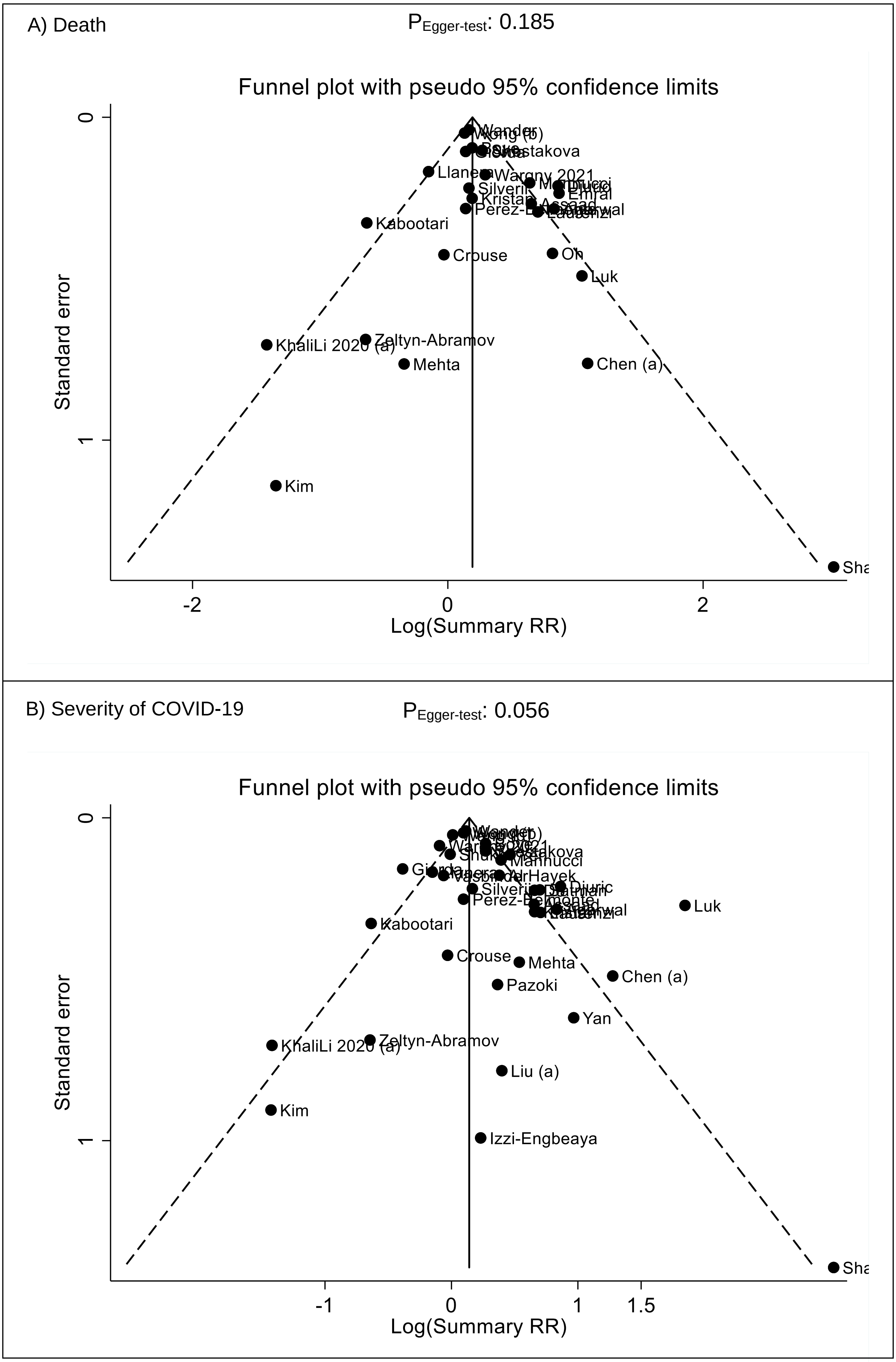

ESM Figure 111: Funnel plot for association between **insulin** and A) death and B) severity of COVID-19 in patients with diabetes and COVID-19

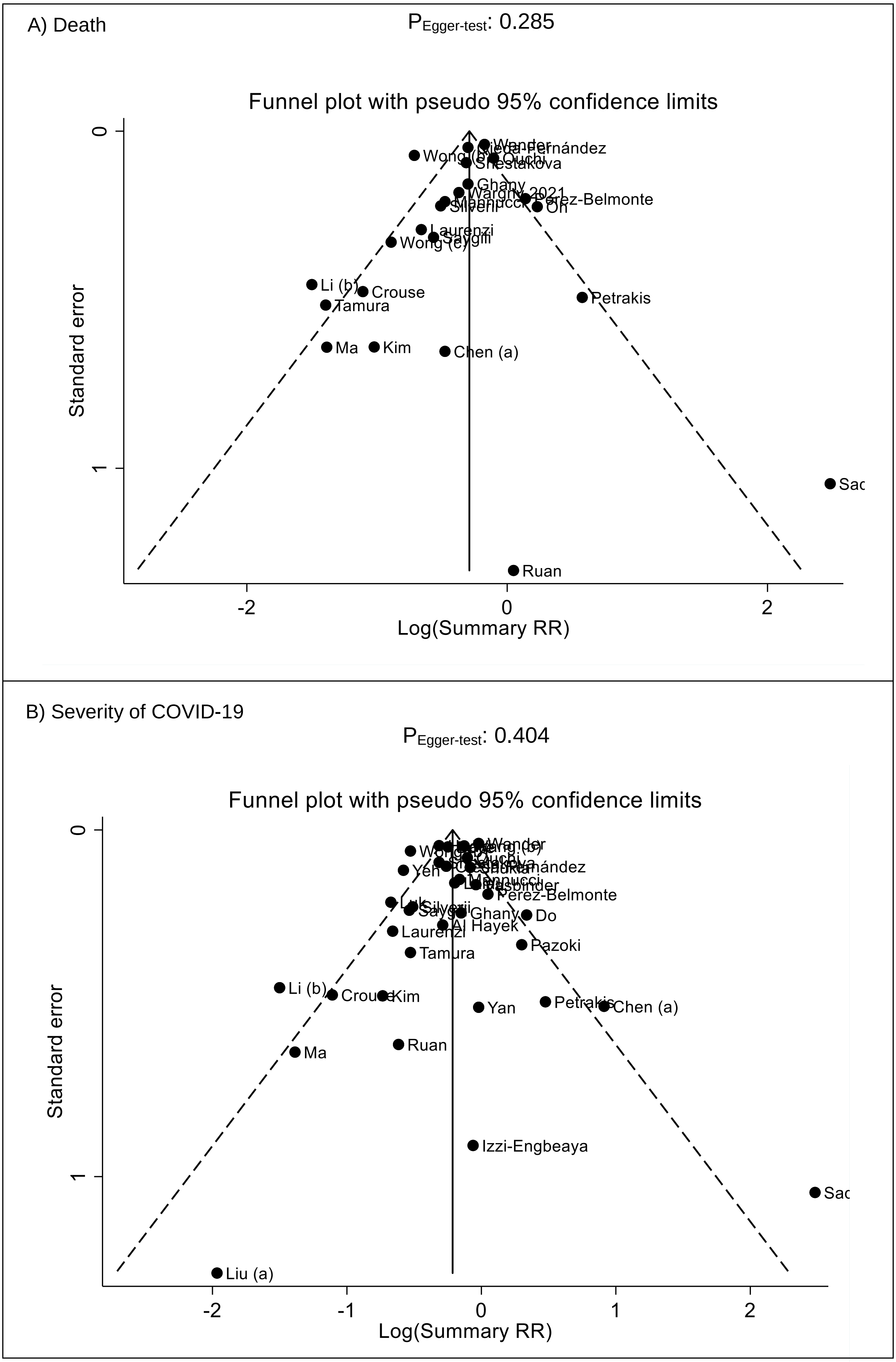

ESM Figure 112: Funnel plot for association between **metformin** and A) death and B) severity of COVID-19 in patients with diabetes and COVID -19

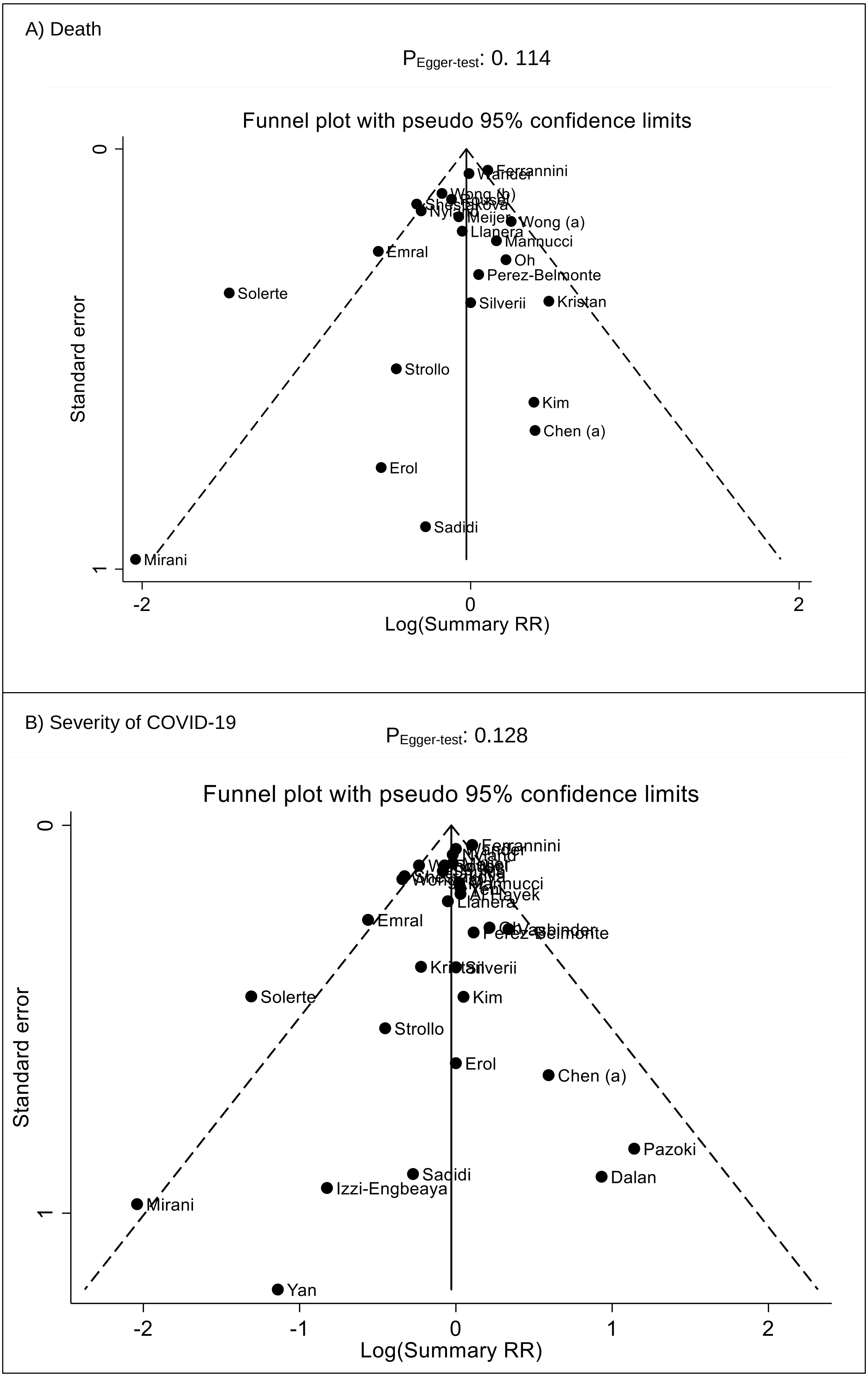

ESM Figure 113: Funnel plot for association between **DPP-4 inhibitor use** and A) death and B) severity of COVID-19 in patients with diabetes and COVID-19

A) Death

No data

B) Severity of COVID-19

$P_{Egger\text{-}test}$: 0.525

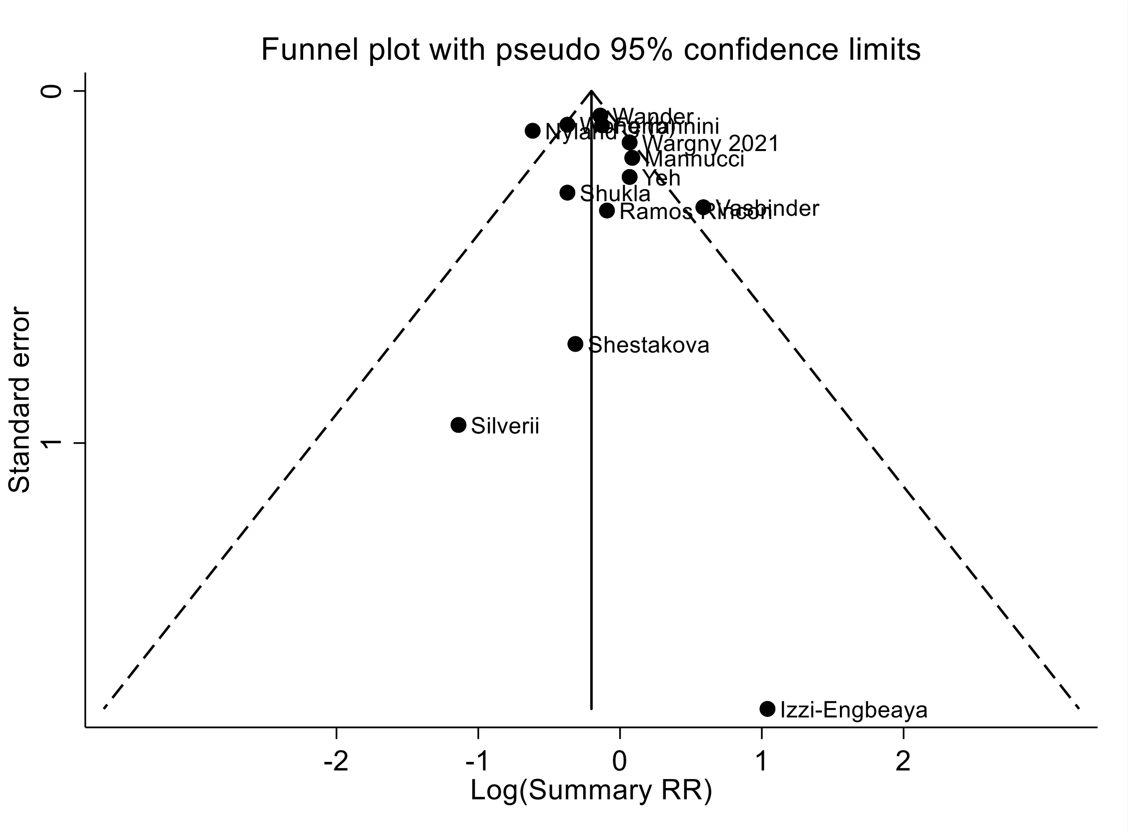

ESM Figure 114: Funnel plot for association between **GLP 1-RA use** and A) death and B) severity of COVID-19 in patients with diabetes and COVID-19

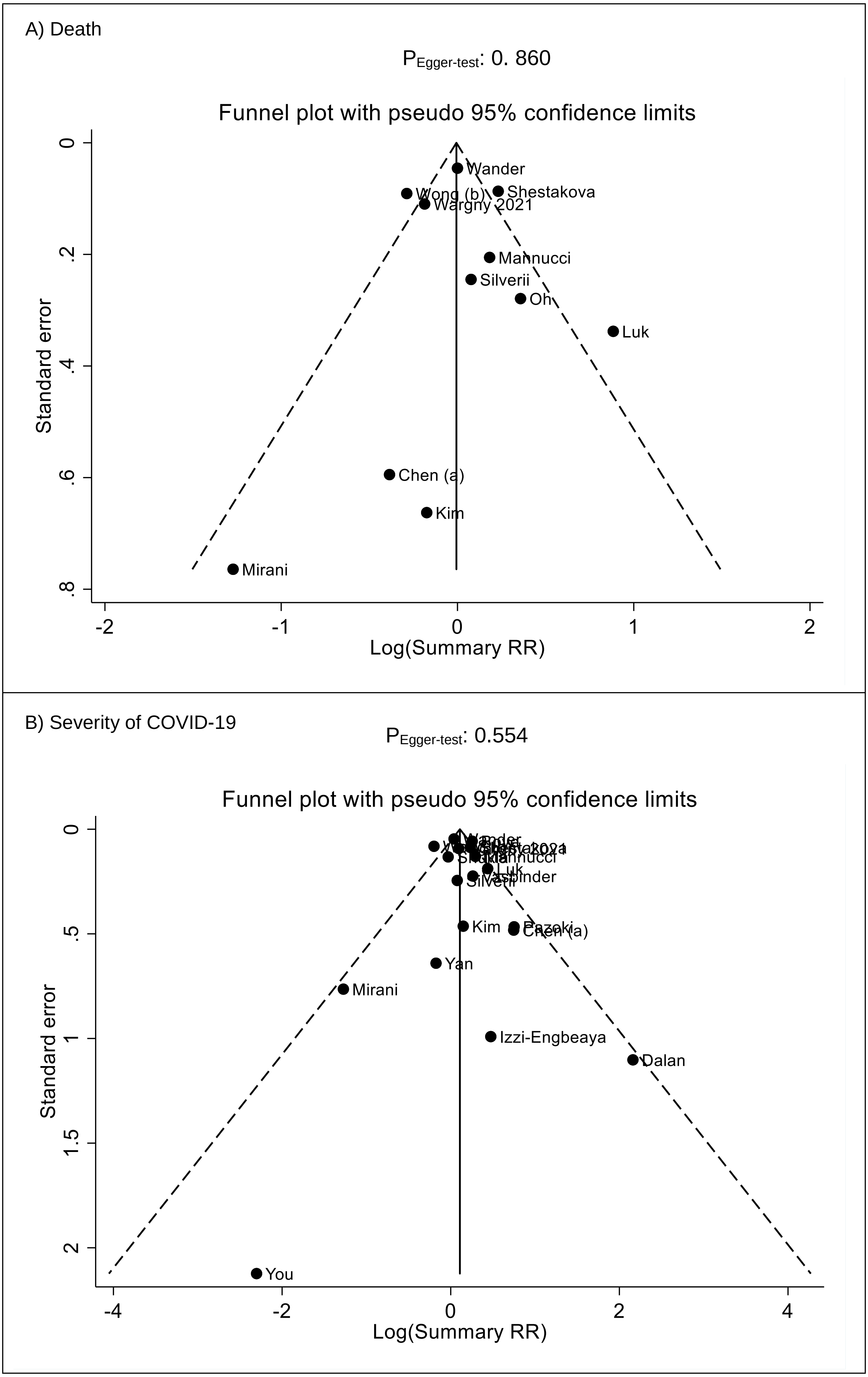

ESM Figure 115: Funnel plot for association between **sulfonylurea/glinides/ secretagogues** and A) death and B) severity of COVID-19 in patients with diabetes and COVID-19

A) Death

No data

B) Severity of COVID-19

$P_{Egger\text{-}test}$: 0.525

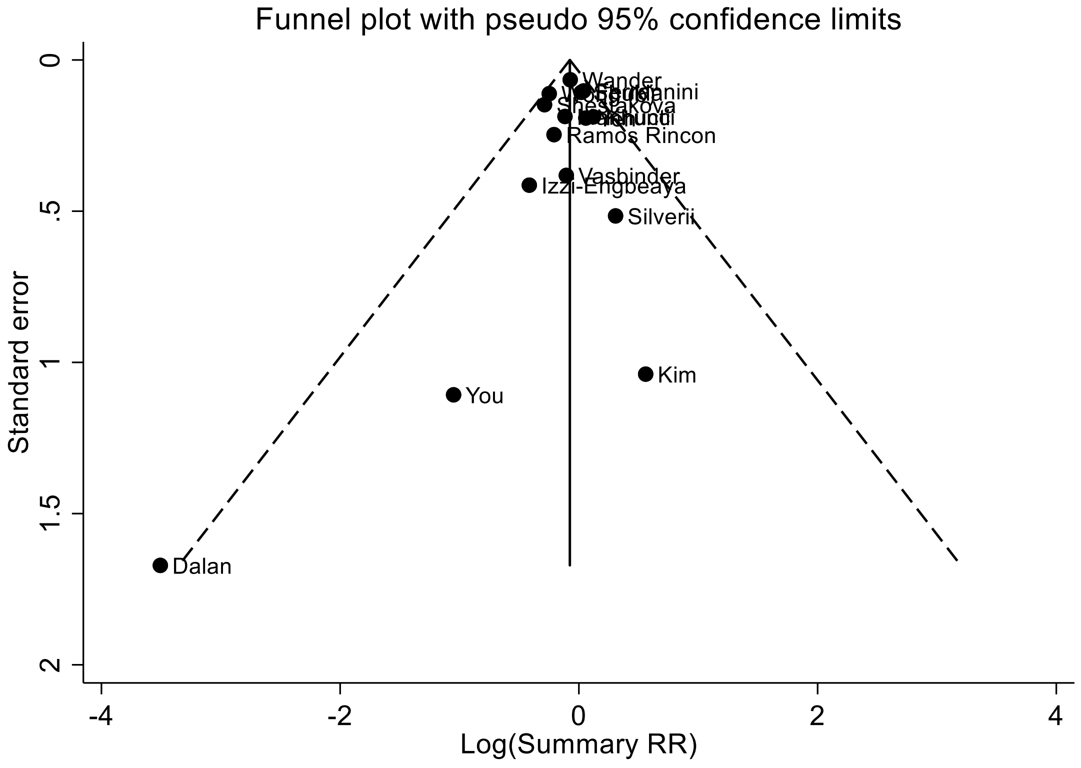

ESM Figure 116: Funnel plot for association between **SGLT-2i use** and A) death and B) severity of COVID-19 in patients with diabetes and COVID-19

A) Death

No data

B) Severity of COVID-19

$P_{Egger\text{-}test}$: 0.018

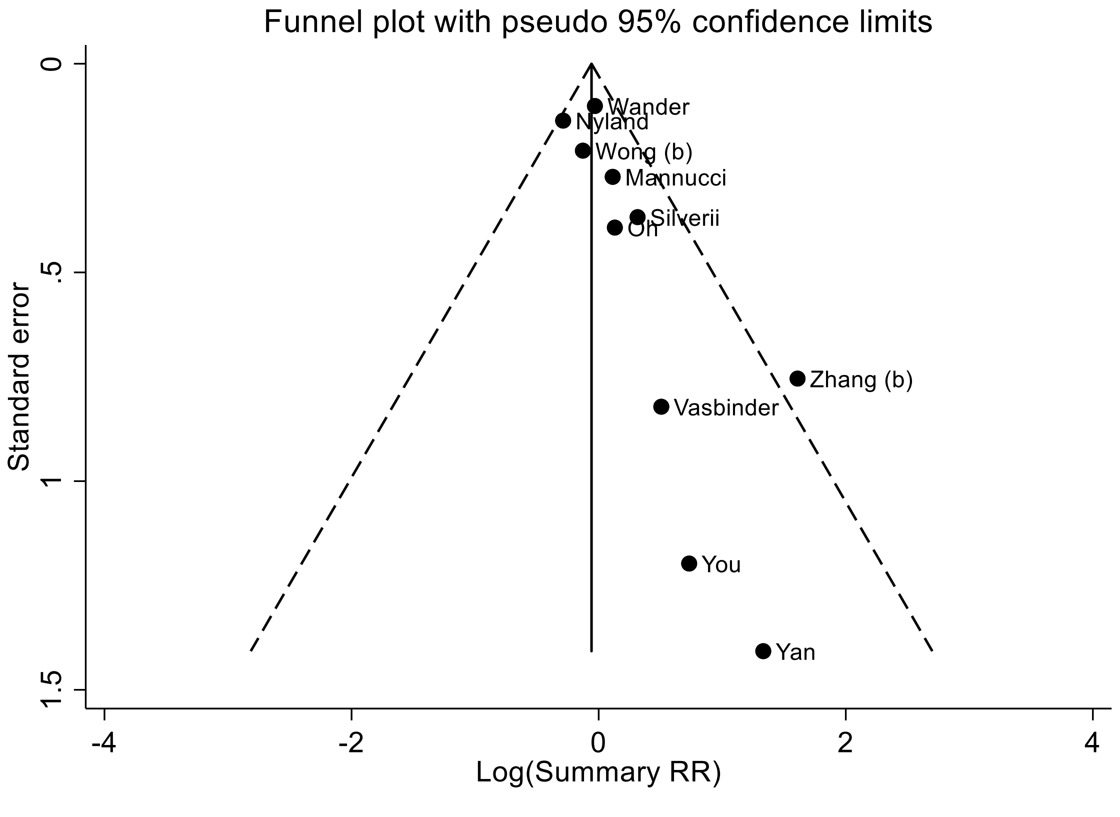

ESM Figure 117: Funnel plot for association between **thiazolidinedione use** and A) death and B) severity of COVID-19 in patients with diabetes and COVID-19

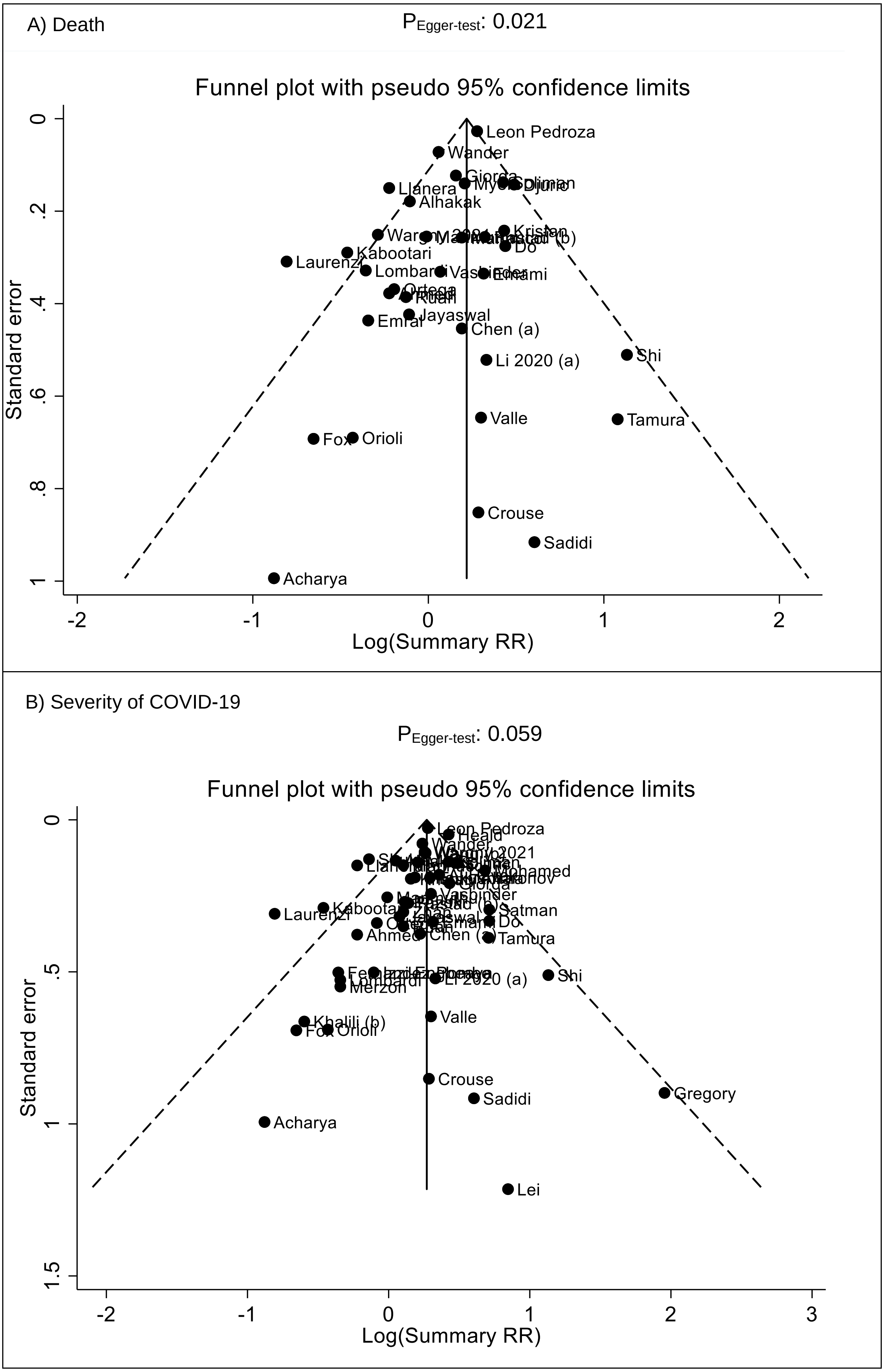

ESM Figure 118: Funnel plot for association between **hypertension** and A) death and B) severity of COVID-19 in patients with diabetes and COVID -19

A) Death

no data

B) Severity of COVID-19

$P_{Egger\text{-}test}$: 0.842

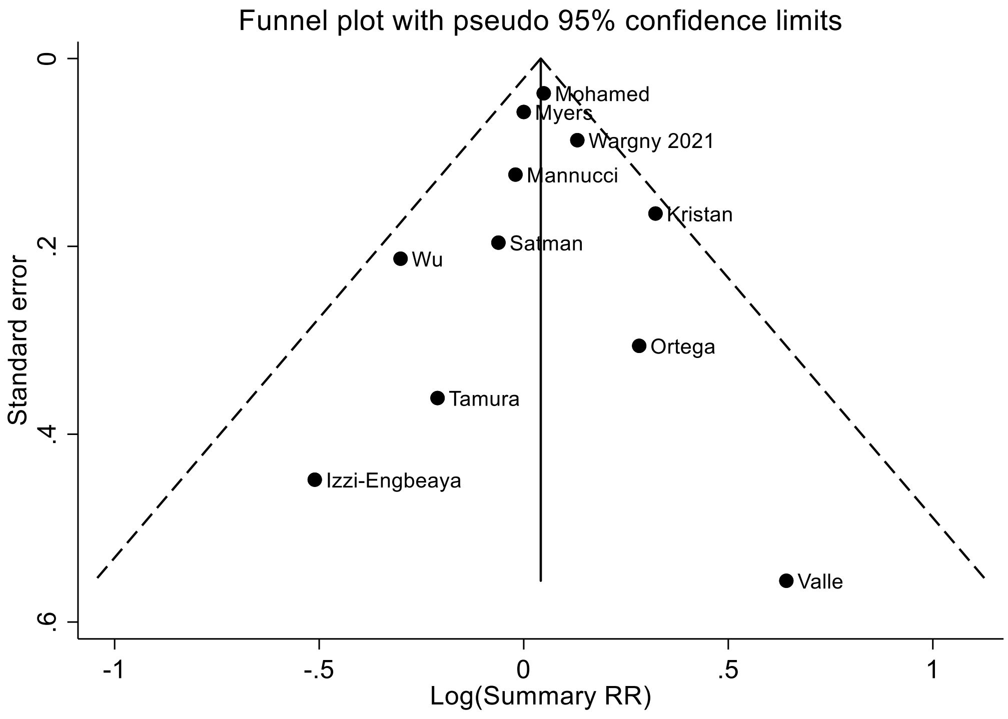

ESM Figure 119: Funnel plot for association between **dyslipidemia** and A) death and B) severity of COVID-19 in patients with diabetes and COVID -19

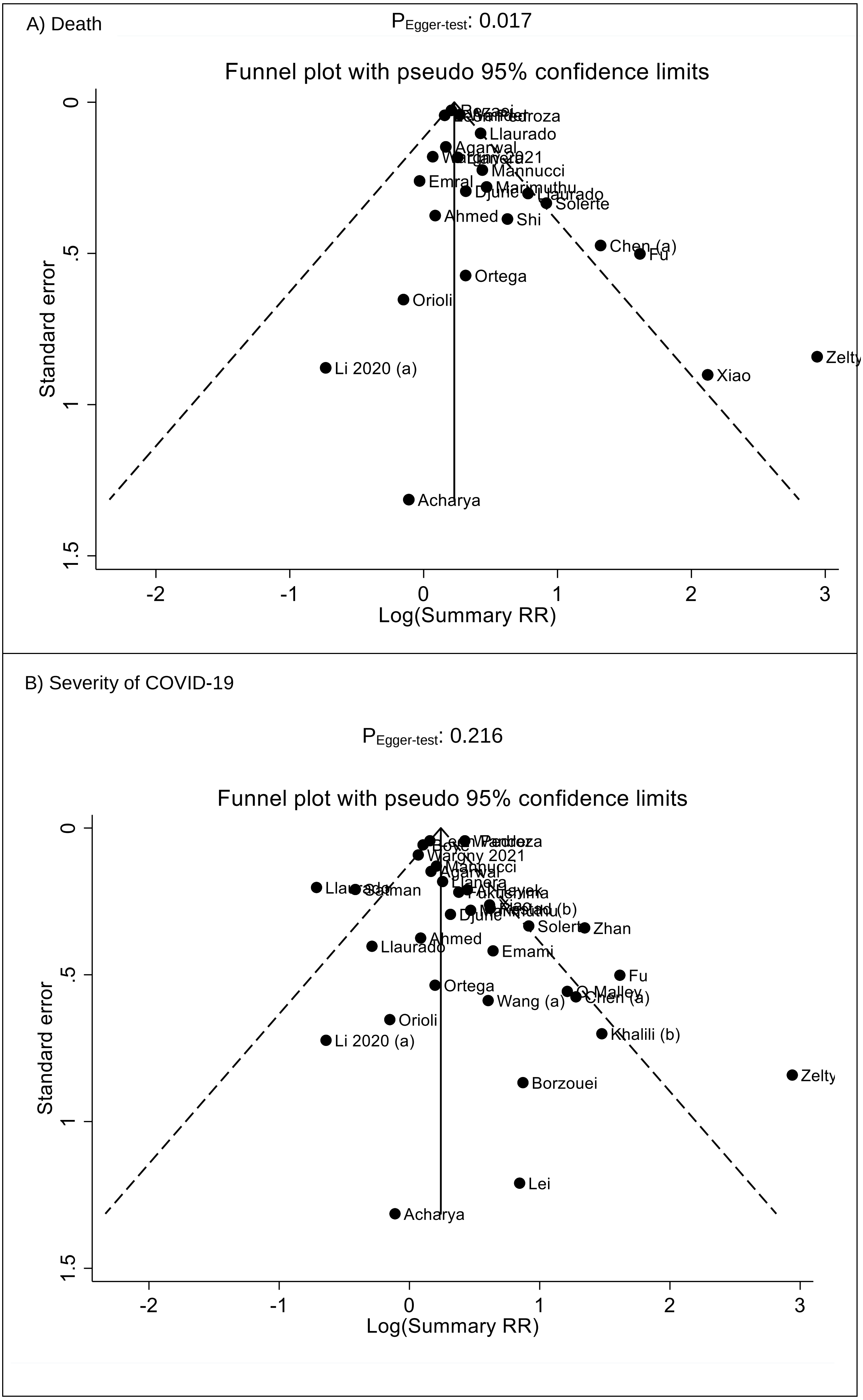

ESM Figure 120: Funnel plot for association between **CVD** and A) death and B) severity of COVID-19 in patients with diabetes and COVID-19

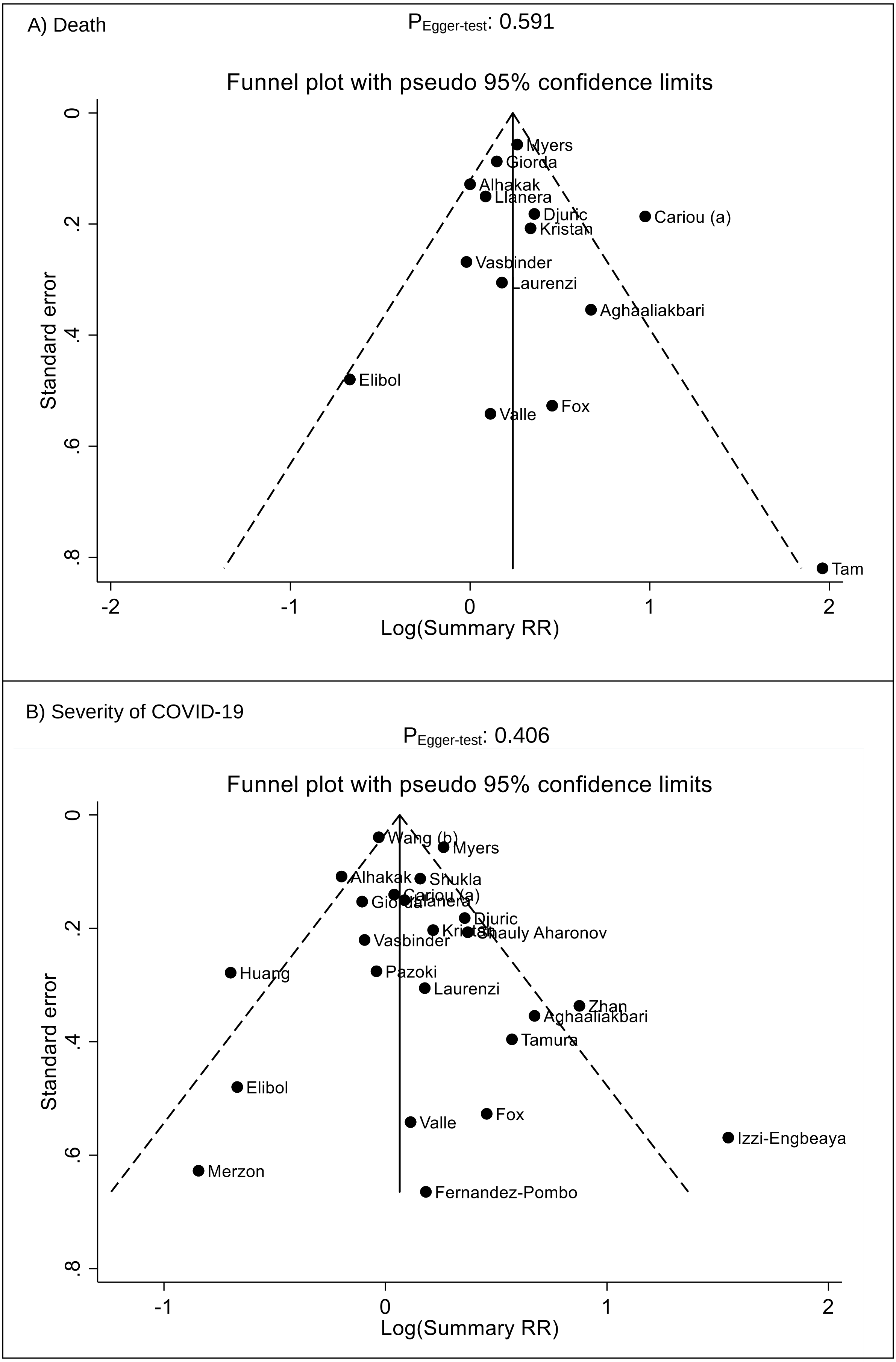

ESM Figure 121: Funnel plot for association between **CAD** and A) death and B) severity of COVID-19 in patients with diabetes and COVID-19

A) Death

$P_{Egger\text{-}test}$: 0.545

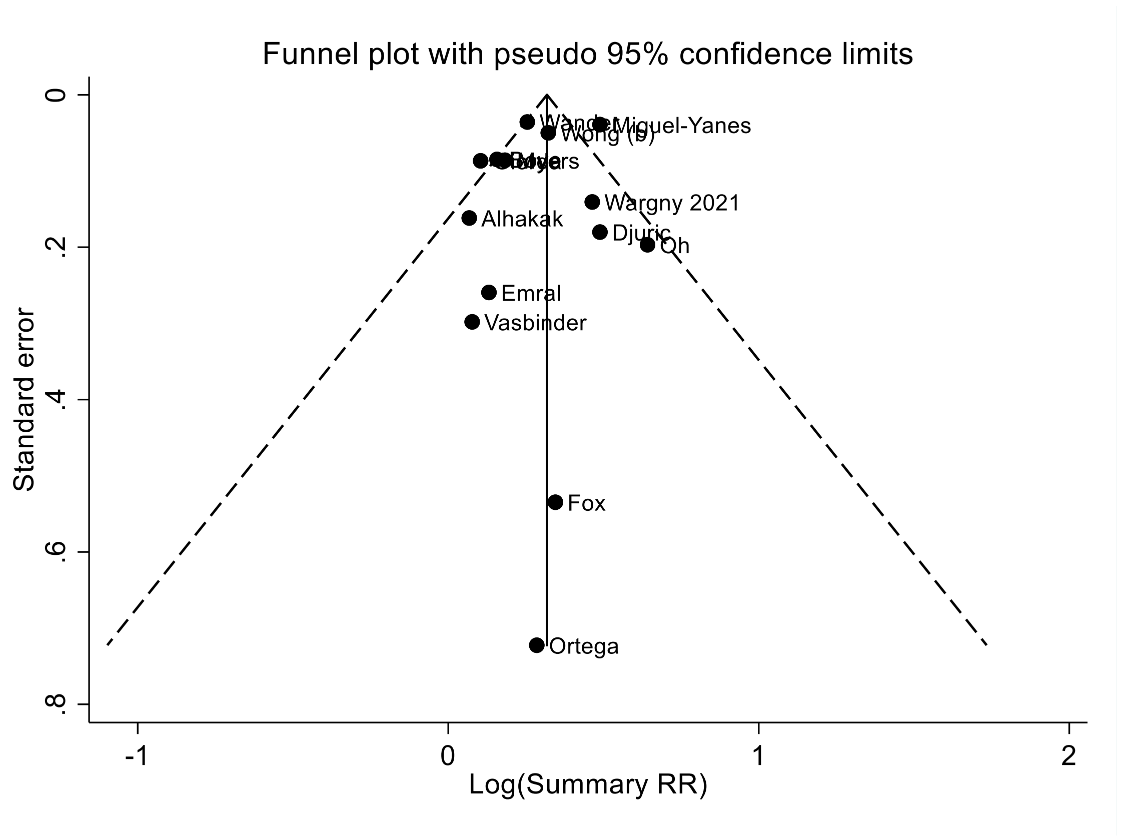

B) Severity of COVID-19

$P_{Egger\text{-}test}$: 0.097

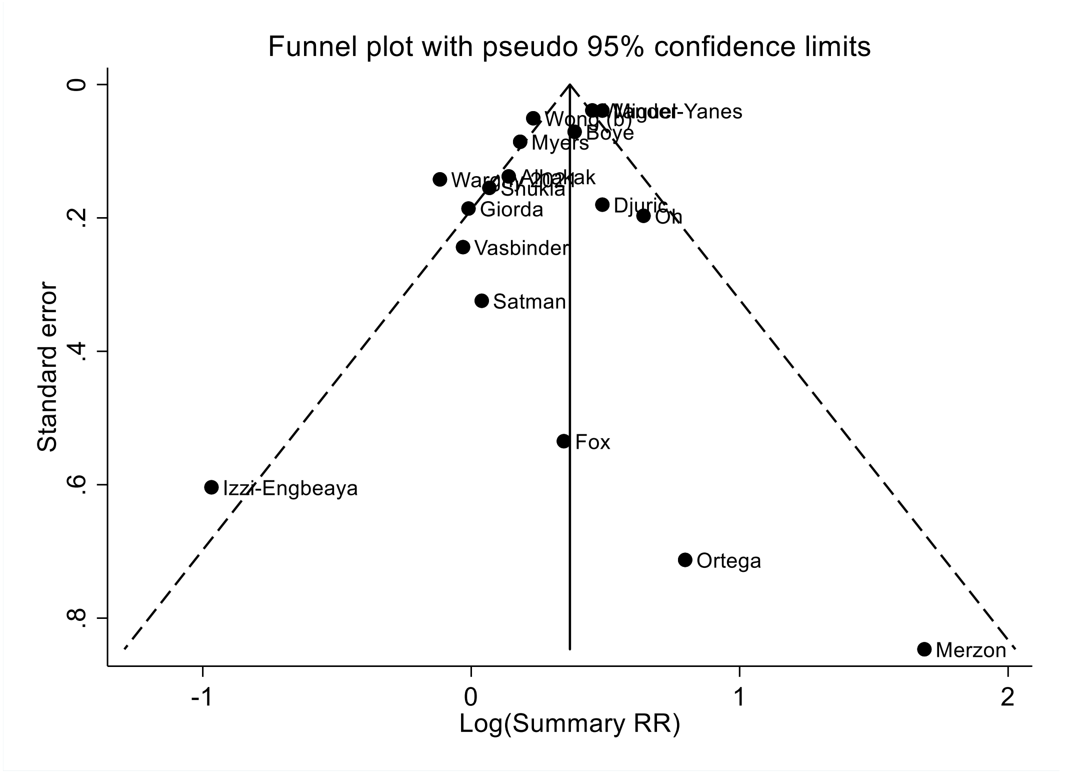

ESM Figure 122: Funnel plot for association between **HF** and A) death and B) severity of COVID-19 in patients with diabetes and COVID-19

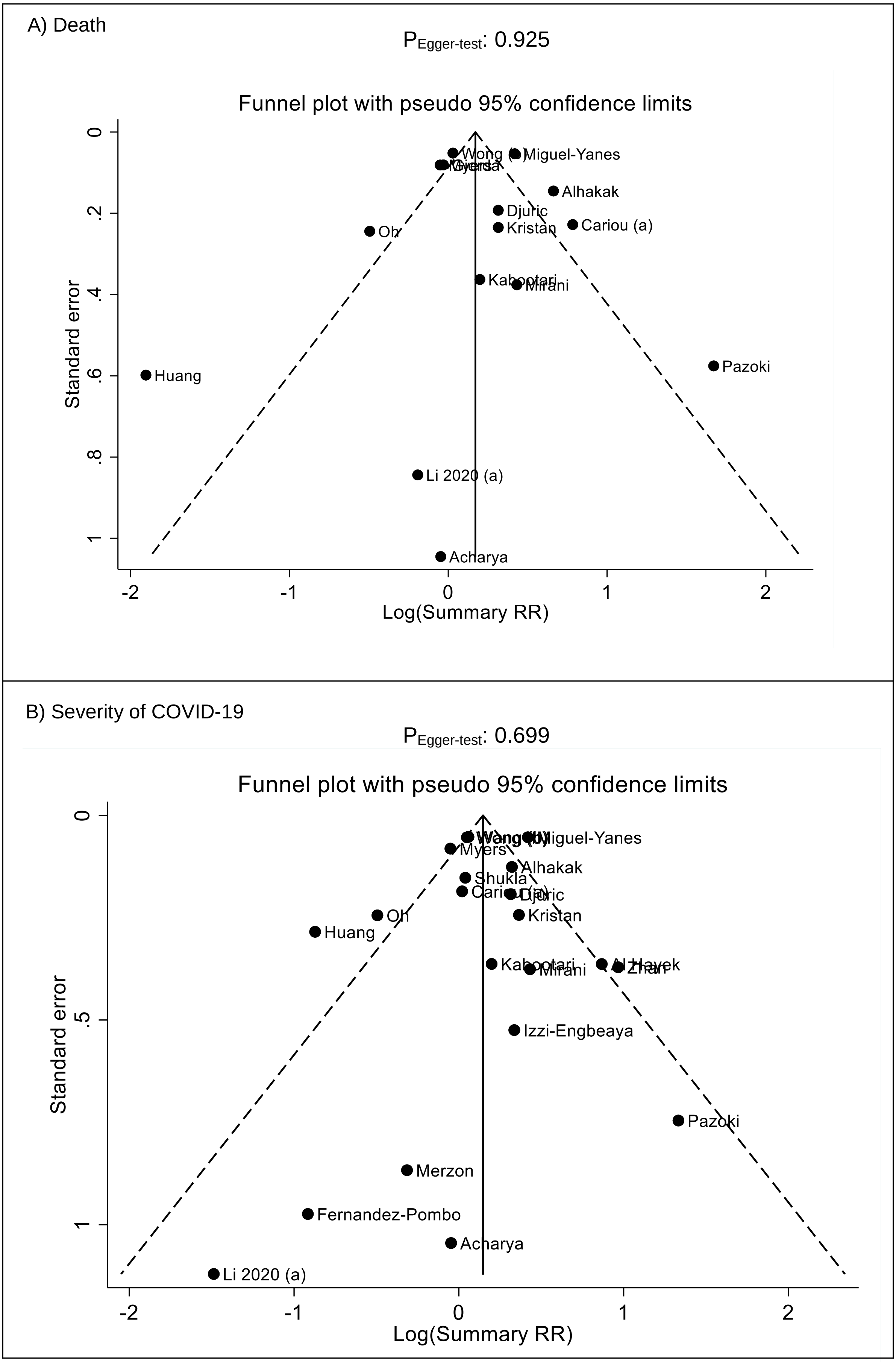

ESM Figure 123: Funnel plot for association between **cerebrovascular diseases** and A) death and B) severity of COVID-19 in patients with diabetes and COVID-19

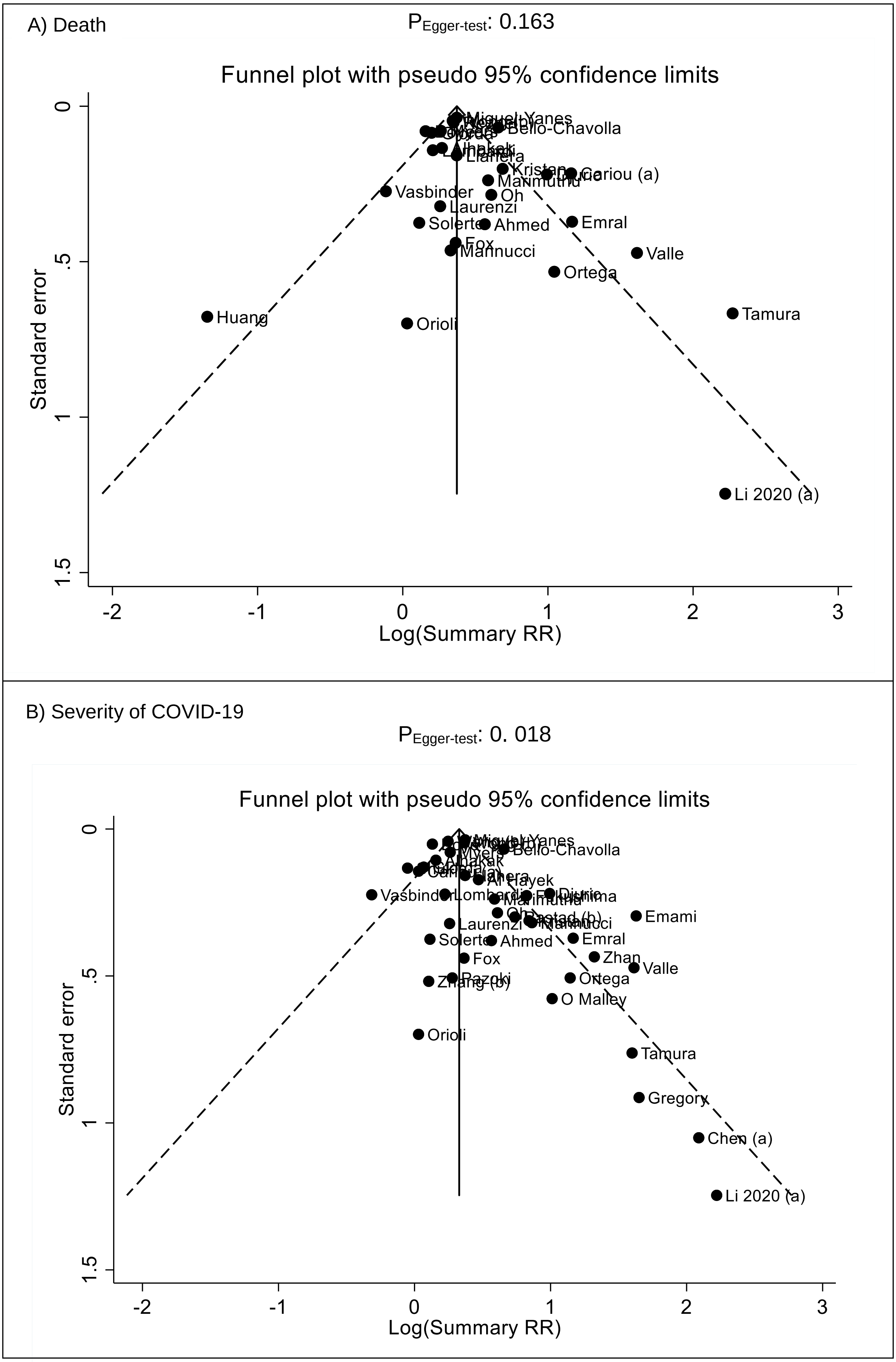

ESM Figure 124: Funnel plot for association between **CKD** and A) death and B) severity of COVID-19 in patients with diabetes and COVID-19

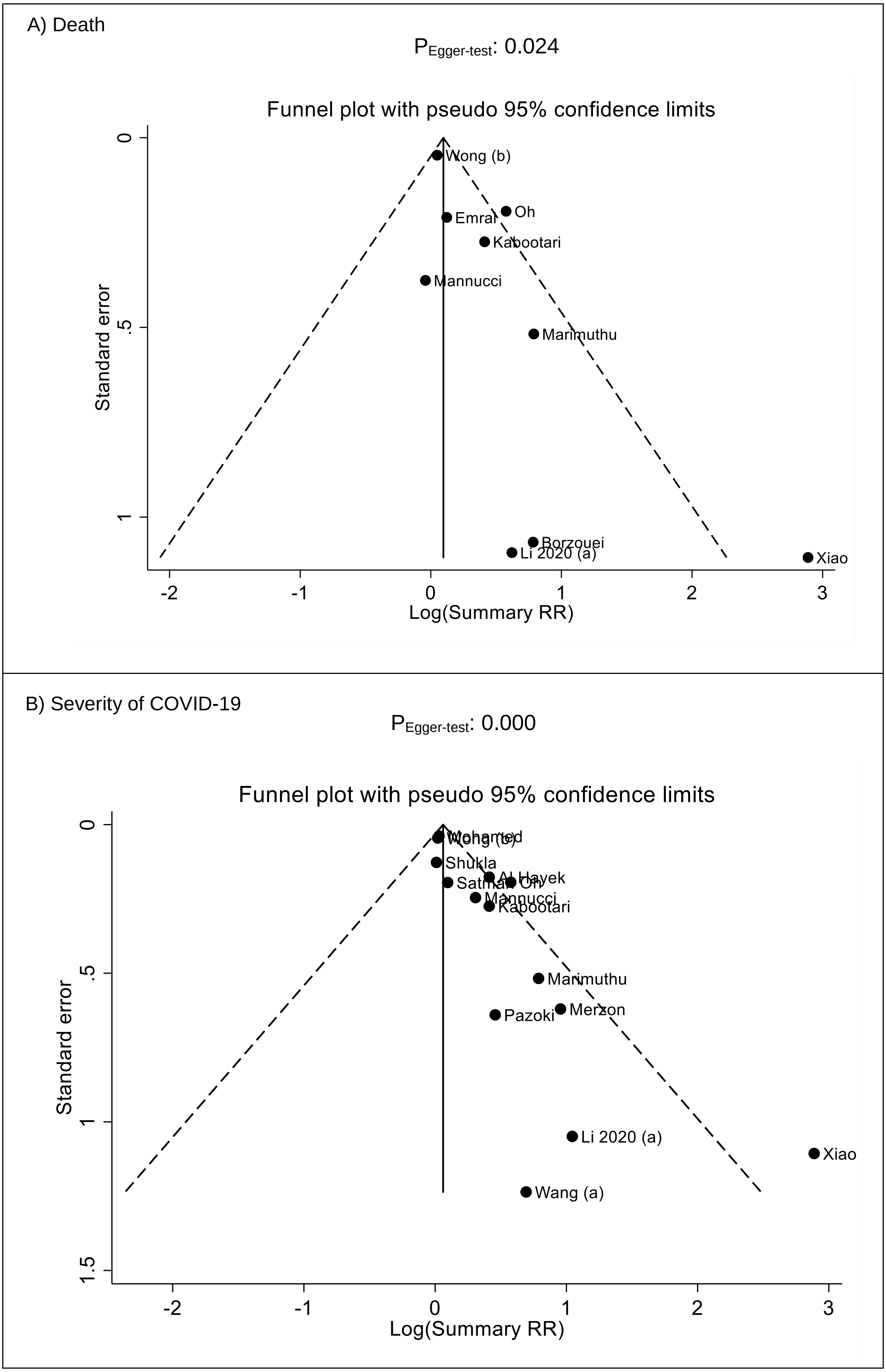

ESM Figure 125: Funnel plot for association between **chronic pulmonary disease** (not specified) and A) death and B) severity of COVID-19 in patients with diabetes and COVID-19

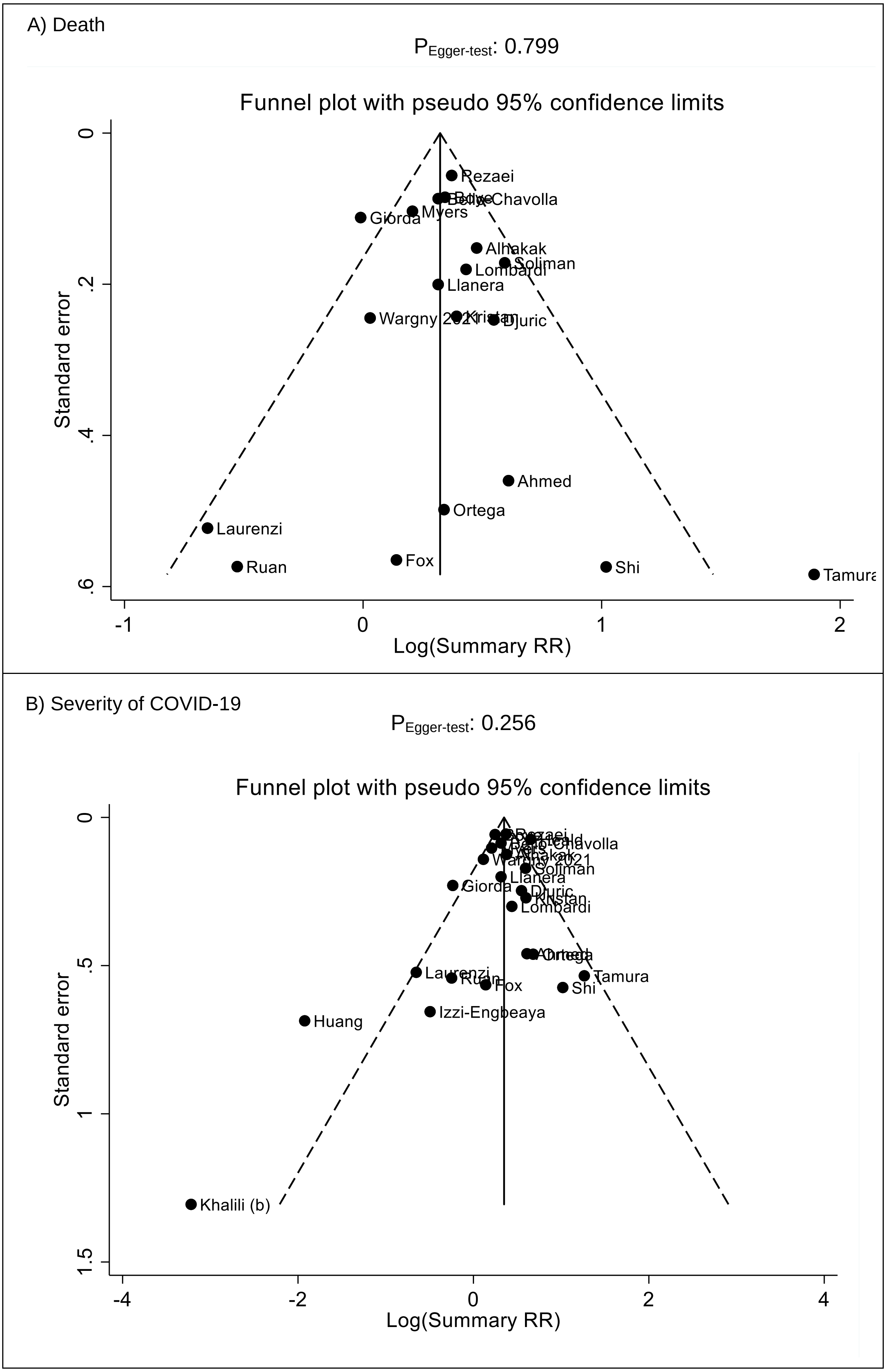

ESM Figure 126: Funnel plot for association between **COPD** and A) death and B) severity of COVID-19 in patients with diabetes and COVID-19

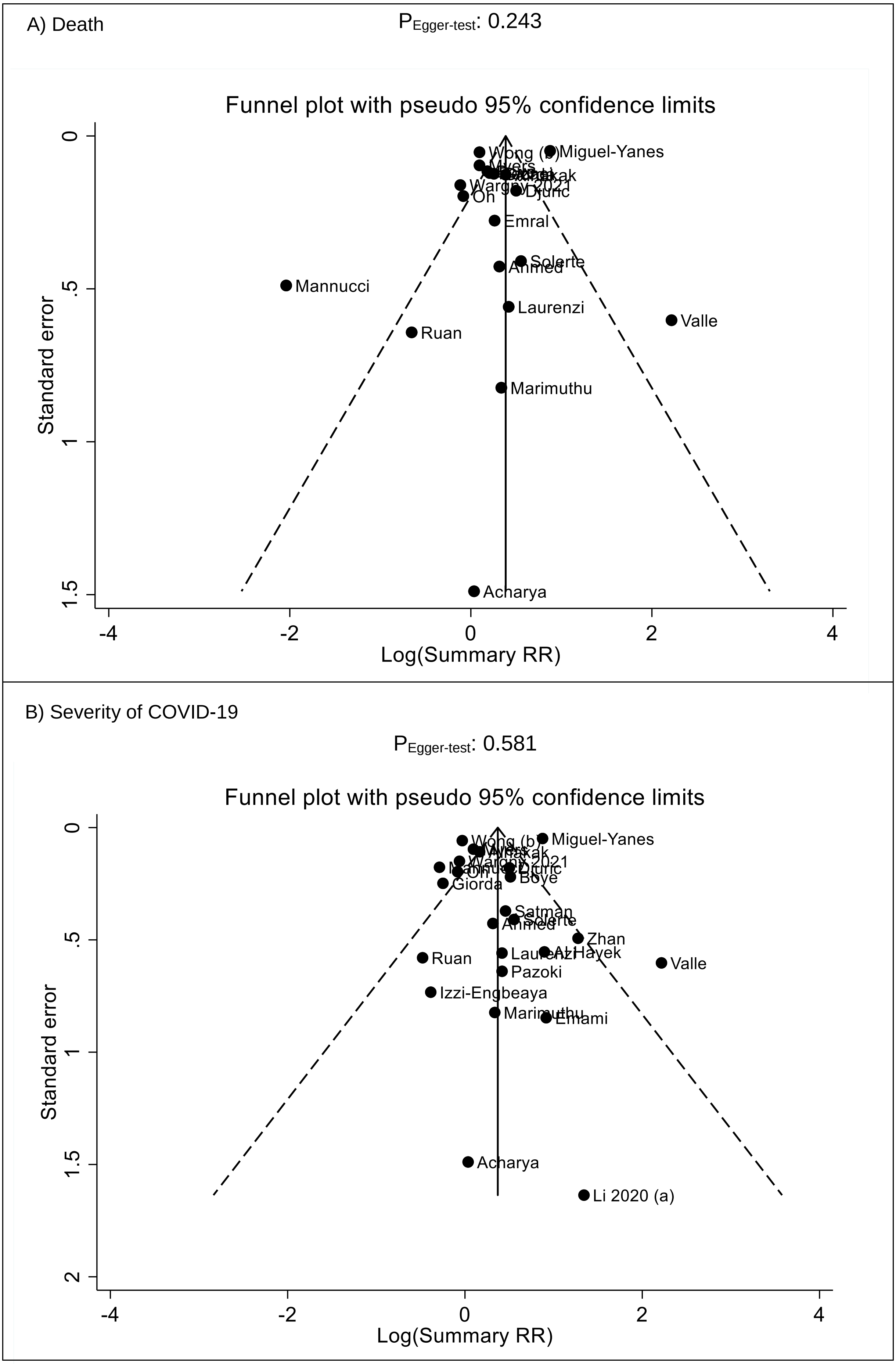

ESM Figure 127: Funnel plot for association between **cancer** and A) death and B) severity of COVID-19 in patients with diabetes and COVID-19

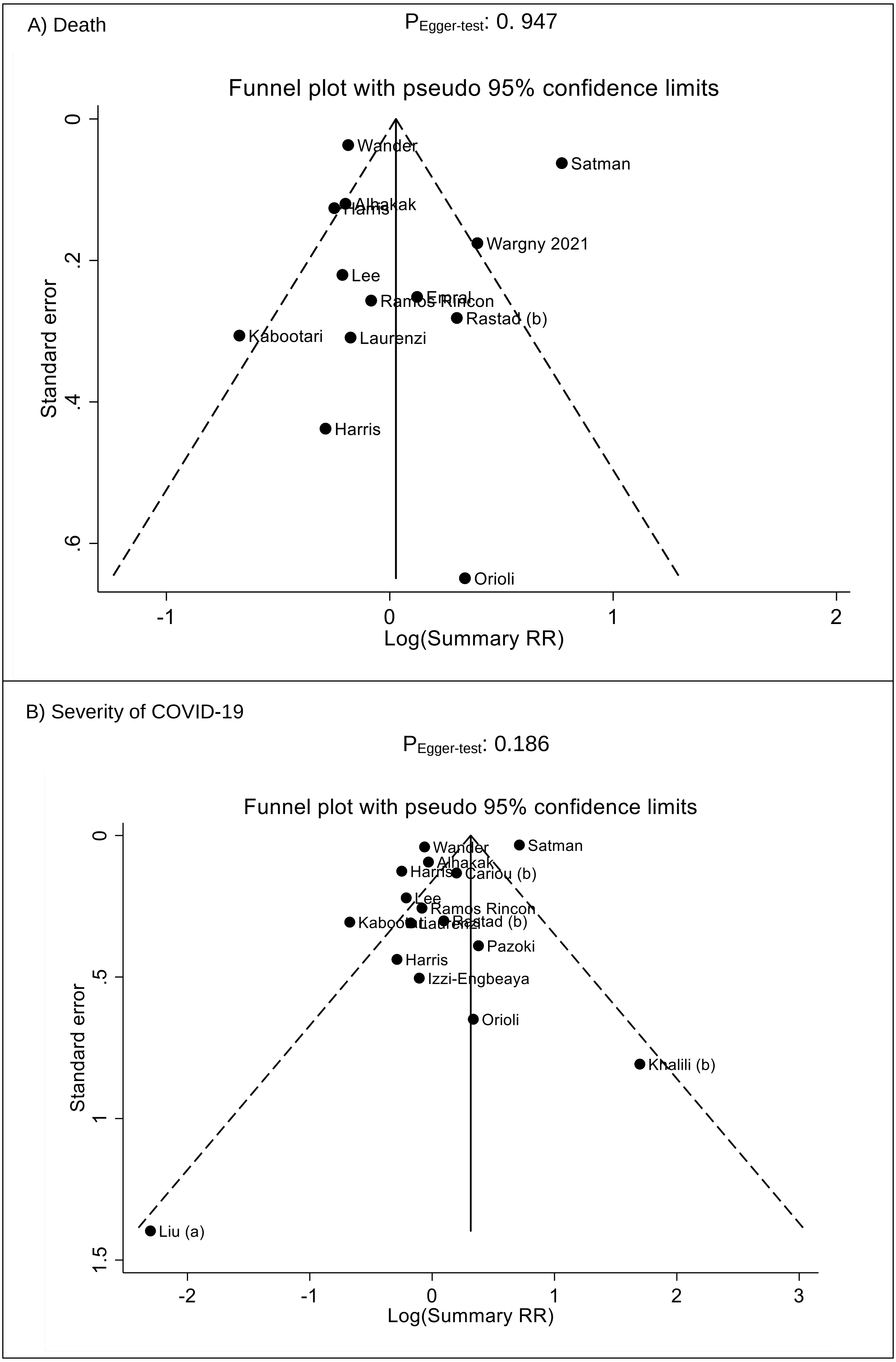

ESM Figure 128: Funnel plot for association between **statins use** and A) death and B) severity of COVID-19 in patients with diabetes and COVID-19

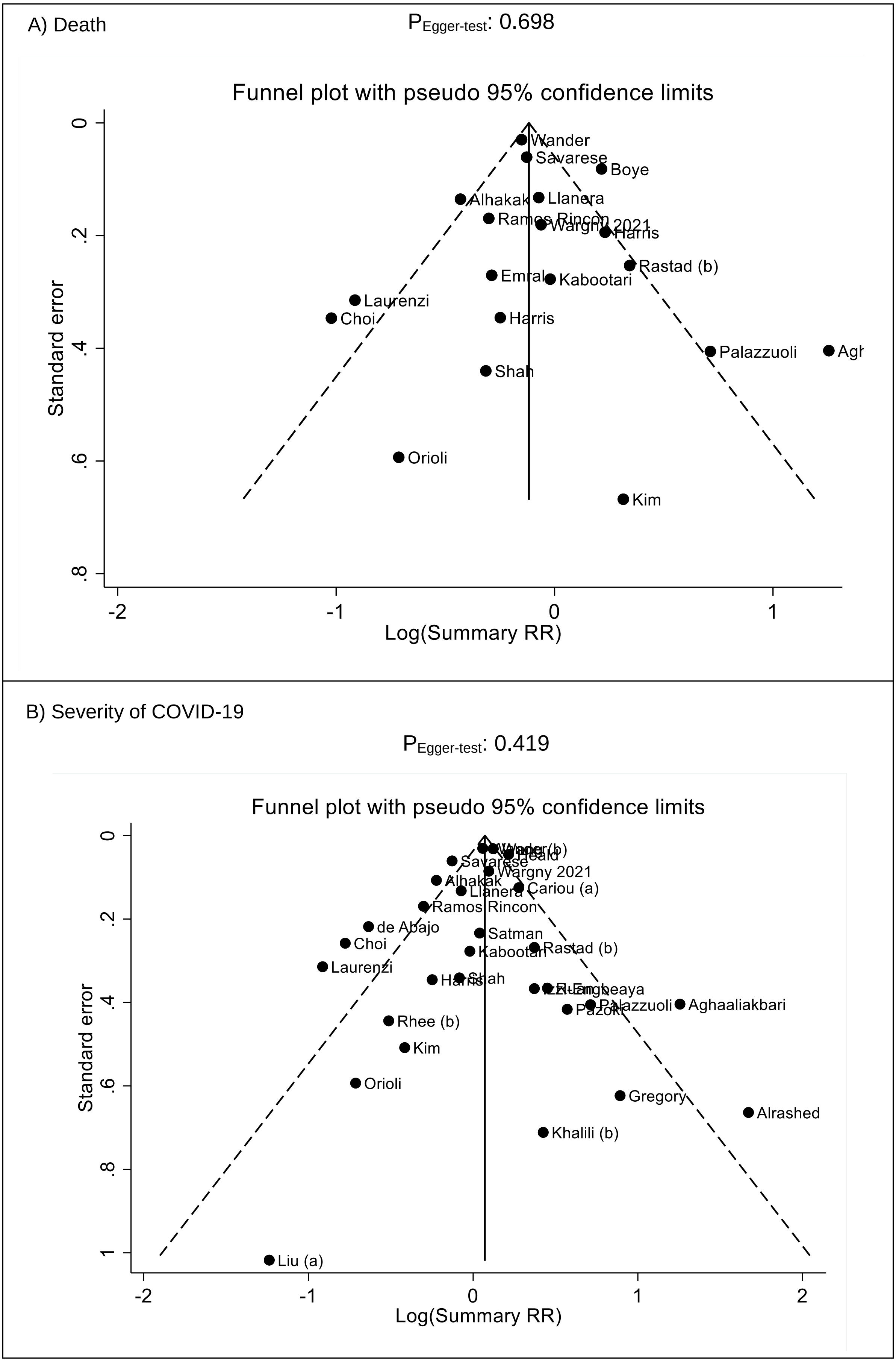

ESM Figure 129: Funnel plot for association between **RAAS inhibitor** use and A) death and B) severity of COVID-19 in patients with diabetes and COVID-19

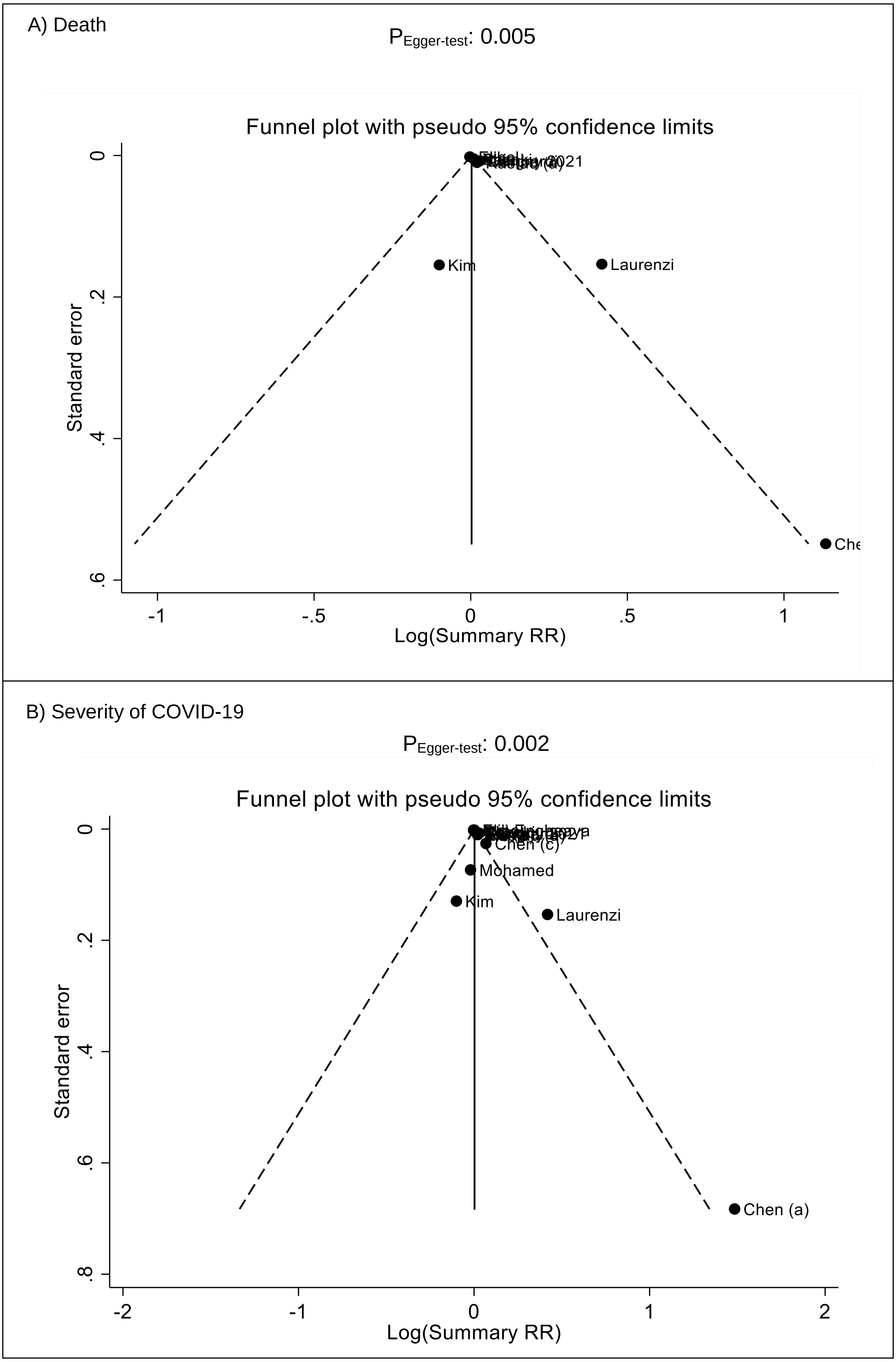

ESM Figure 130: Funnel plot for association between **CRP** and A) death and B) severity of COVID-19 in patients with diabetes and COVID-19

A) Death

No data

B) Severity of COVID-19

$P_{Egger\text{-}test}$: 0.383

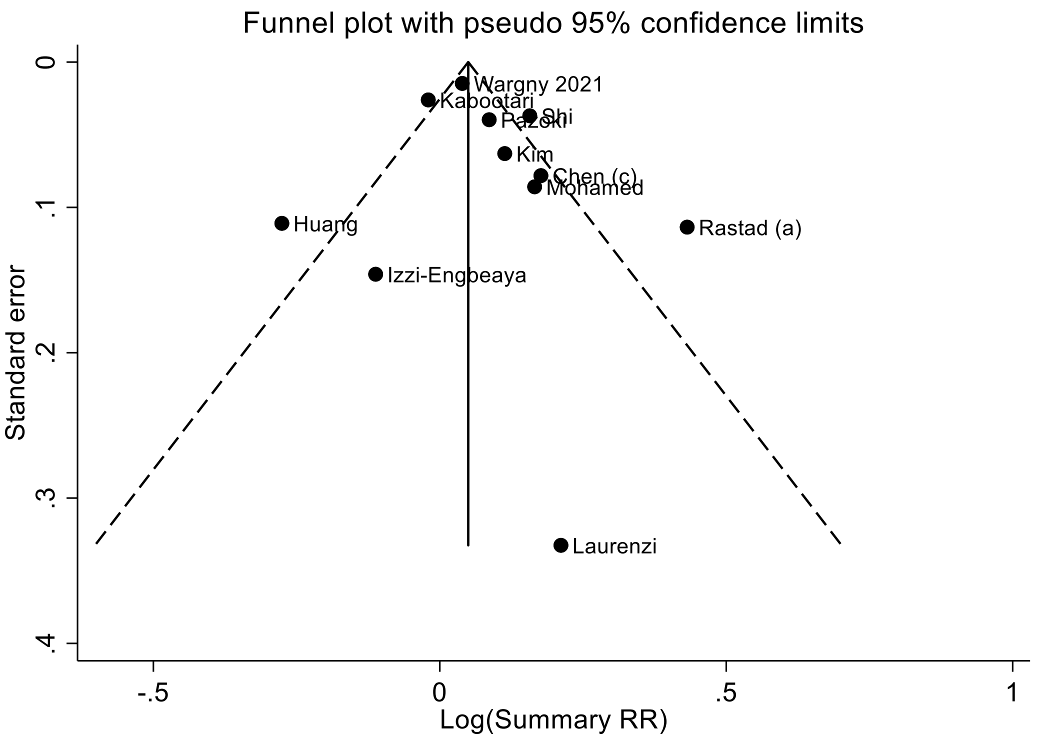

ESM Figure 131: Funnel plot for association between **white blood cell count** and A) death and B) severity of COVID-19 in patients with diabetes and COVID-19

A) Death

No data

B) Severity of COVID-19

$P_{Egger-test}$: 0.013

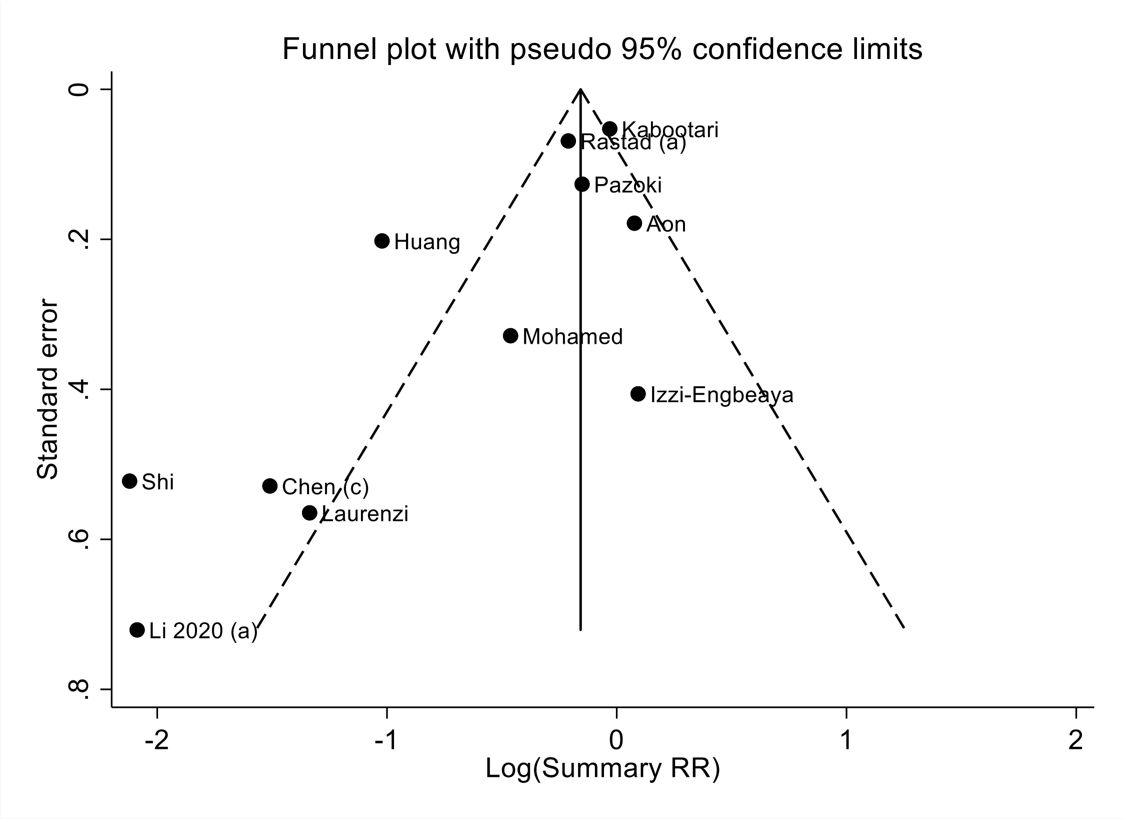

ESM Figure 132: Funnel plot for association between **lymphocyte count** and A) death and B) severity of COVID-19 in patients with diabetes and COVID-19

References

[1] Huang S, Wang J, Liu F, et al. (2020) COVID-19 patients with hypertension have more severe disease: a multicenter retrospective observational study. Hypertens res

[2] Posso M, Comas M, Román M, et al. (2020) Comorbidities and Mortality in Patients With COVID-19 Aged 60 Years and Older in a University Hospital in Spain. Arch Bronconeumol 56(11): 756-758

[3] Slaunwhite AK, Gan WQ, Xavier C, Zhao B, Buxton JA, Desai R (2020) Overdose and risk factors for coronavirus disease 2019. Drug Alcohol Depend 212: 108047-108047

[4] Bean DM, Kraljevic Z, Searle T, et al. (2020) Angiotensin-converting enzyme inhibitors and angiotensin II receptor blockers are not associated with severeCOVID-19infection in a multi-siteUKacute hospital trust. European Journal of Heart Failure 22(6): 967-974. 10.1002/ejhf.1924

[5] Bramante C, Ingraham N, Murray T, et al. (2020) Observational Study of Metformin and Risk of Mortality in Patients Hospitalized with Covid-19. medRxiv. 10.1101/2020.06.19.20135095

[6] Fadini GP, Morieri ML, Longato E, et al. Exposure to dipeptidyl-peptidase-4 inhibitors andCOVID-19 among people with type 2 diabetes: A case-control study. Diabetes Obesity & Metabolism. 10.1111/dom.14097

[7] Williamson EJ, Walker AJ, Bhaskaran K, et al. (2020) OpenSAFELY: factors associated with COVID-19 death in 17 million patients. Nature. 10.1038/s41586-020-2521-4

[8] Zhang J, Kong W, Xia P, et al. (2020) Impaired Fasting Glucose and Diabetes Are Related to Higher Risks of Complications and Mortality Among Patients With Coronavirus Disease 2019. Front Endocrinol (Lausanne) 11: 525. 10.3389/fendo.2020.00525

[9] Alattar R, Ibrahim TBH, Shaar SH, et al. (2020) Tocilizumab for the treatment of severe coronavirus disease 2019. J Med Virol. 10.1002/jmv.25964

[10] Bhargava A, Fukushima EA, Levine M, et al. (2020) Predictors for Severe COVID-19 Infection. Clinical infectious diseases : an official publication of the Infectious Diseases Society of America. 10.1093/cid/ciaa674

[11] Cen Y, Chen X, Shen Y, et al. (2020) Risk factors for disease progression in mild to moderate COVID-19 patients- a multi-center observational study. Clin microbiol infect

[12] Cummings MJ, Baldwin MR, Abrams D, et al. (2020) Epidemiology, clinical course, and outcomes of critically ill adults with COVID-19 in New York City: a prospective cohort study. Lancet 395(10239): 1763-1770. 10.1016/s0140-6736(20)31189-2

[13] Daniel EF, Joseph C, Magdalena ES, et al. (2020) Famotidine Use is Associated with Improved Clinical Outcomes in Hospitalized COVID-19 Patients: A Retrospective Cohort Study. medRxiv. 10.1101/2020.05.01.20086694

[14] Docherty AB, Harrison EM, Green CA, et al. (2020) Features of 20 133 UK patients in hospital with covid-19 using the ISARIC WHO Clinical Characterisation Protocol: prospective observational cohort study. Bmj 369: m1985. 10.1136/bmj.m1985

[15] Escalera-Antezana JP, Lizon-Ferrufino NF, Maldonado-Alanoca A, et al. (2020) Risk factors for mortality in patients with Coronavirus Disease 2019 (COVID-19) in Bolivia: An analysis of the first 107 confirmed cases. Infez Med 28(2): 238-242

[16] Fabio C, Antonella C, Patrizia R-Q, et al. (2020) Early predictors of clinical outcomes of COVID-19 outbreak in Milan, Italy. Clinical Immunology. 10.1016/j.clim.2020.108509

[17] Francesca B, Maria Elena F, Tiziano C, et al. (2020) Predictors of severe or lethal COVID-19, including Angiotensin Converting Enzyme Inhibitors and Angiotensin II Receptor Blockers, in a sample of infected Italian citizens. medRxiv. 10.1101/2020.05.21.20109082

[18] Galloway JB, Norton S, Barker RD, et al. (2020) A clinical risk score to identify patients with COVID-19 at high risk of critical care admission or death: An observational cohort study. The Journal of infection. 10.1016/j.jinf.2020.05.064

[19] Guan W-J, Liang W-H, Zhao Y, et al. (2020) Comorbidity and its impact on 1590 patients with COVID-19 in China: a nationwide analysis. Eur respir j 55(5)

[20] Hirsch JS, Ng JH, Ross DW, et al. (2020) Acute kidney injury in patients hospitalized with COVID-19. Kidney international. 10.1016/j.kint.2020.05.006

[21] Hu L, Chen S, Fu Y, et al. (2020) Risk Factors Associated with Clinical Outcomes in 323 COVID-19 Hospitalized Patients in Wuhan, China. Clinical infectious diseases : an official publication of the Infectious Diseases Society of America. 10.1093/cid/ciaa539

[22] Huadong Y, Ana MV, Amrita V, et al. (2020) Role of Drugs Affecting the Renin-Angiotensin-Aldosterone System on Susceptibility and Severity of COVID-19: A Large Case-Control Study from Zheijang Province, China. medRxiv. 10.1101/2020.04.24.20077875

[23] Huang R, Zhu L, Xue L, et al. (2020) Clinical findings of patients with coronavirus disease 2019 in Jiangsu province, China: A retrospective, multi-center study. PLoS neglected tropical diseases 14(5): e0008280. 10.1371/journal.pntd.0008280

[24] Hur K, Price CPE, Gray EL, et al. (2020) Factors Associated With Intubation and Prolonged Intubation in Hospitalized Patients With COVID-19. Otolaryngol Head Neck Surg: 194599820929640-194599820929640
[25] Imam Z, Odish F, Gill I, et al. (2020) Older age and comorbidity are independent mortality predictors in a large cohort of 1305 COVID-19 patients in Michigan, United States. Journal of internal medicine. 10.1111/joim.13119
[26] Jang JG, Hur J, Choi EY, Hong KS, Lee W, Ahn JH (2020) Prognostic Factors for Severe Coronavirus Disease 2019 in Daegu, Korea. Journal of Korean medical science. 10.3346/jkms.2020.35.e209
[27] Lee JY, Kim HA, Huh K, et al. (2020) Risk Factors for Mortality and Respiratory Support in Elderly Patients Hospitalized with COVID-19 in Korea. Journal of Korean medical science. 10.3346/jkms.2020.35.e223
[28] Li P, Chen L, Liu Z, et al. (2020) Clinical Features and Short-term Outcomes of Elderly Patients With COVID-19. Int j infect dis
[29] Lin F, Jun F, Hui-Xian X, et al. (2020) Influence factors of death risk among COVID-19 patients in Wuhan, China: a hospital-based case-cohort study. medRxiv. 10.1101/2020.03.13.20035329
[30] Lindsay K, Shikha G, Alissa OH, et al. (2020) Interim Analysis of Risk Factors for Severe Outcomes among a Cohort of Hospitalized Adults Identified through the U.S. Coronavirus Disease 2019 (COVID-19)-Associated Hospitalization Surveillance Network (COVID-NET). medRxiv. 10.1101/2020.05.18.20103390
[31] Ling H, Shaoqiu C, Yuanyuan F, et al. (2020) Risk Factors Associated with Clinical Outcomes in 323 COVID-19 Patients in Wuhan, China. medRxiv. 10.1101/2020.03.25.20037721
[32] Matthew JC, Matthew RB, Darryl A, et al. (2020) Epidemiology, clinical course, and outcomes of critically ill adults with COVID-19 in New York City: a prospective cohort study. medRxiv. 10.1101/2020.04.15.20067157
[33] Palaiodimos L, Kokkinidis DG, Li W, et al. (2020) Severe obesity, increasing age and male sex are independently associated with worse in-hospital outcomes, and higher in-hospital mortality, in a cohort of patients with COVID-19 in the Bronx, New York. Metabolism 108: 154262-154262
[34] Paolo Giorgi R, Massimiliano M, Debora F, et al. (2020) Characteristics and outcomes of a cohort of SARS-CoV-2 patients in the Province of Reggio Emilia, Italy. medRxiv. 10.1101/2020.04.13.20063545
[35] Shen X, Lin F, Jun F, et al. (2020) Acute kidney injury at early stage as a negative prognostic indicator of patients with COVID-19: a hospital-based retrospective analysis. medRxiv. 10.1101/2020.03.24.20042408
[36] Shi Q, Zhang X, Jiang F, et al. (2020) Clinical Characteristics and Risk Factors for Mortality of COVID-19 Patients With Diabetes in Wuhan, China: A Two-Center, Retrospective Study. Diabetes Care. 10.2337/dc20-0598
[37] Simonnet A, Chetboun M, Poissy J, et al. (2020) High Prevalence of Obesity in Severe Acute Respiratory Syndrome Coronavirus-2 (SARS-CoV-2) Requiring Invasive Mechanical Ventilation. Obesity (Silver Spring)
[38] Suleyman G, Fadel RA, Malette KM, et al. (2020) Clinical Characteristics and Morbidity Associated With Coronavirus Disease 2019 in a Series of Patients in Metropolitan Detroit. JAMA network open 3(6): e2012270. 10.1001/jamanetworkopen.2020.12270
[39] Targher G, Mantovani A, Wang XB, et al. (2020) Patients with diabetes are at higher risk for severe illness from COVID-19. Diabetes & metabolism. 10.1016/j.diabet.2020.05.001
[40] Tian G, Qiao C, Zhangsheng Y, et al. (2020) History of Coronary Heart Disease Increases the Mortality Rate of Coronavirus Disease 2019 (COVID-19) Patients: A Nested Case-Control Study Based on Publicly Reported Confirmed Cases in Mainland China. medRxiv. 10.1101/2020.03.23.20041848
[41] Toussie D, Voutsinas N, Finkelstein M, et al. (2020) Clinical and Chest Radiography Features Determine Patient Outcomes In Young and Middle Age Adults with COVID-19. Radiology: 201754-201754
[42] Wang L, He WB, Yu XM, et al. (2020) Coronavirus disease 2019 in elderly patients: Characteristics and prognostic factors based on 4-week follow-up. Journal of Infection 80(6): 639-645. 10.1016/j.jinf.2020.03.019
[43] Wu C, Chen X, Cai Y, et al. (2020) Risk Factors Associated With Acute Respiratory Distress Syndrome and Death in Patients With Coronavirus Disease 2019 Pneumonia in Wuhan, China. JAMA internal medicine. 10.1001/jamainternmed.2020.0994
[44] Wu J, Zhang J, Sun X, et al. (2020) Influence of diabetes mellitus on the severity and fatality of SARS-CoV-2 infection. Diabetes obes metab
[45] Yan Y, Yang Y, Wang F, et al. (2020) Clinical characteristics and outcomes of patients with severe covid-19 with diabetes. 8(1)

[46] Yan Z, Yanhui C, Minxue S, et al. (2020) Comorbid Diabetes Mellitus was Associated with Poorer Prognosis in Patients with COVID-19: A Retrospective Cohort Study. medRxiv. 10.1101/2020.03.24.20042358
[47] Ye C, Zhang S, Zhang X, et al. (2020) Impact of comorbidities on patients with COVID-19: A large retrospective study in Zhejiang, China. J Med Virol. 10.1002/jmv.26183
[48] Zhang Y, Cui Y, Shen M, et al. (2020) Association of diabetes mellitus with disease severity and prognosis in COVID-19: A retrospective cohort study. Diabetes Res Clin Pract 165: 108227-108227
[49] Zhang YT, Deng AP, Hu T, et al. (2020) [Clinical outcomes of COVID-19 cases and influencing factors in Guangdong province]. Zhonghua liu xing bing xue za zhi = Zhonghua liuxingbingxue zazhi 41(0): E057. 10.3760/cma.j.cn112338-20200318-00378
[50] Zhichao F, Jennifer L, Shanhu Y, et al. (2020) The Use of Adjuvant Therapy in Preventing Progression to Severe Pneumonia in Patients with Coronavirus Disease 2019: A Multicenter Data Analysis. medRxiv. 10.1101/2020.04.08.20057539
[51] Zhou F, Yu T, Du RH, et al. (2020) Clinical course and risk factors for mortality of adult inpatients with COVID-19 in Wuhan, China: a retrospective cohort study. Lancet 395(10229): 1054-1062. 10.1016/s0140-6736(20)30566-3
[52] Yang J, Zheng Y, Gou X, et al. (2020) Prevalence of comorbidities and its effects in patients infected with SARS-CoV-2: a systematic review and meta-analysis. International Journal of Infectious Diseases 94: 91-95. 10.1016/j.ijid.2020.03.017
[53] Abbas HM, Nassir KF, Aga QAA, et al. (2021) Presenting the characteristics, smoking versus diabetes, and outcome among patients hospitalized with COVID-19. Journal of Medical Virology 93(3): 1556-1567. 10.1002/jmv.26487
[54] Abderrahim O, Stanislas G, Isabelle Clerc U, et al. (2020) Follow-Up of Multi-Organ Dysfunction and Inflammation Using Biomarker Kinetics in Patients with Severe COVID-19 Disease and Association with Disease Outcomes: Results from a Referral Center Cohort in the North East of France. SSRN. 10.2139/ssrn.3590489
[55] Abdollahi A, Sarvestani HK, Rafat Z, et al. (2020) The Association Between the Level of Serum 25(OH) Vitamin D, Obesity, and underlying Diseases with the risk of Developing COVID-19 Infection: A case-control study of hospitalized patients in Tehran, Iran. J Med Virol. 10.1002/jmv.26726
[56] Abohamr SI, Abazid RM, Aldossari MA, et al. (2020) Clinical characteristics and in-hospital mortality of COVID-19 adult patients in Saudi Arabia. Saudi Med J 41(11): 1217-1226. 10.15537/smj.2020.11.25495
[57] Abohamr SI, Aldossari MA, Alaklobi FA, et al. (2020) Clinical characteristics and in-hospital outcome of medical staff infected with COVID-19 in Saudi Arabia. A retrospective single-center study. Saudi Med J 41(12): 1336-1343. 10.15537/smj.2020.12.25514
[58] Abraha HE, Gessesse Z, Gebrecherkos T, et al. (2021) Clinical features and risk factors associated with morbidity and mortality among patients with COVID-19 in northern Ethiopia. Int J Infect Dis 105: 776-783. 10.1016/j.ijid.2021.03.037
[59] Acharya D, Park J, Lee Y, et al. (2020) Clinical Characteristics of the COVID-19 Patients with Pneumonia Detected by Computerized Tomography but Negative for Infiltration by X-ray. Healthcare (Basel) 8(4). 10.3390/healthcare8040518
[60] Adibul Hassan R, Mirza Naqi Z, Asma A, et al. (2020) COVID-19 Pneumonia: Epidemiology, Clinical Characteristics and Outcomes from a Developing Country. A Single Center Experience from Pakistan. SSRN. 10.2139/ssrn.3711651
[61] Adnan IQ (2020) Pre-existing Cardiovascular Disease in United States Population at High Risk for Severe COVID-19 Infection. medRxiv. 10.1101/2020.05.11.20089714
[62] Adrish M, Chilimuri S, Sun H, Mantri N, Yugay A, Zahid M (2020) The Association of Renin-Angiotensin-Aldosterone System Inhibitors With Outcomes Among a Predominantly Ethnic Minority Patient Population Hospitalized With COVID-19: The Bronx Experience. Cureus 12(9): e10217. 10.7759/cureus.10217
[63] Agarwal N, Biswas B, Lohani P (2020) Epidemiological determinants of COVID-19 infection and mortality: A study among patients presenting with severe acute respiratory illness during the pandemic in Bihar, India. Niger Postgrad Med J 27(4): 293-301. 10.4103/npmj.npmj_301_20
[64] Ahlström B, Frithiof R, Hultström M, Larsson IM, Strandberg G, Lipcsey M (2021) The swedish covid-19 intensive care cohort: Risk factors of ICU admission and ICU mortality. Acta Anaesthesiol Scand 65(4): 525-533. 10.1111/aas.13781
[65] Aidin R, Elin Allansson K, Araz R, et al. (2021) Severe COVID-19 in People with Type 1 and Type 2 Diabetes in Sweden: A Nationwide Retrospective Case-Control Study.
[66] Akhtar A, Bano S, Iqbal A, et al. (2020) Clinical Characteristics of Severe Covid Pneumonia: Exploring New Trends in ICU. ResearchSquare. 10.21203/rs.3.rs-69224/v1

[67] Akyüz A, Işık F, Aslan B, et al. (2021) The effect of RAAS inhibitors on acute hypoxemic respiratory failure and in-hospital mortality in the hypertensive Covid-19 patients. Clin Exp Hypertens: 1-10. 10.1080/10641963.2021.1916947
[68] Al Kuwari HM, Rahim HFA, Abu-Raddad LJ, et al. (2020) Epidemiological investigation of the first 5685 cases of SARS-CoV-2 infection in Qatar, 28 February-18 April 2020. Bmj Open 10(10). 10.1136/bmjopen-2020-040428
[69] Aladağ N, Atabey RD (2020) The role of concomitant cardiovascular diseases and cardiac biomarkers for predicting mortality in critical COVID-19 patients. Acta Cardiol: 1-8. 10.1080/00015385.2020.1810914
[70] Alahmad B, Al-Shammari AA, Bennakhi A, Al-Mulla F, Ali H (2020) Fasting Blood Glucose and COVID-19 Severity: Nonlinearity Matters. Diabetes Care 43(12): 3113-3116. 10.2337/dc20-1941
[71] Alamdari NM, Afaghi S, Rahimi FS, et al. (2020) Mortality Risk Factors among Hospitalized COVID-19 Patients in a Major Referral Center in Iran. Tohoku J Exp Med 252(1): 73-84. 10.1620/tjem.252.73
[72] Alamdari NM, Rahimi FS, Afaghi S, et al. (2020) The impact of metabolic syndrome on morbidity and mortality among intensive care unit admitted COVID-19 patients. Diabetes Metab Syndr 14(6): 1979-1986. 10.1016/j.dsx.2020.10.012
[73] Alamri F, Alsofayan Y, AlRuthia Y, et al. (2021) Predictors of Hospitalization Among Older Adults with COVID-19 in Saudi Arabia: A Cross-Sectional Study of a Nationally Representative Sample. Risk Manag Healthc Policy 14: 875-886. 10.2147/rmhp.S294786
[74] Alan P, Osman K, Jennifer M, et al. (2020) Disparities in COVID-19 Hospitalizations and Mortality among Black and Hispanic Patients: Cross-Sectional Analysis from the Greater Houston Metropolitan Area. medRxiv. 10.1101/2020.08.19.20177956
[75] Albitar O, Ballouze R, Ooi JP, Ghadzi SMS (2020) Risk factors for mortality among COVID-19 patients. Diabetes Research and Clinical Practice 166. 10.1016/j.diabres.2020.108293
[76] AlDossary R, Alnimr A, Aljindan R, et al. (2020) Predictors of illness severity in COVID-19 cases. ResearchSquare. 10.21203/rs.3.rs-120489/v1
[77] Alejandra C-M, Carlos MG-L, Mercedes A, Ana Cristina S, Hector L-F (2020) Municipality-level predictors of COVID-19 mortality in Mexico: a cautionary tale. medRxiv. 10.1101/2020.07.11.20151522
[78] Alguwaihes AM, Al-Sofiani ME, Megdad M, et al. (2020) Diabetes and Covid-19 among hospitalized patients in Saudi Arabia: a single-centre retrospective study. Cardiovasc Diabetol 19(1): 205-205
[79] Ali AES, Gianfranco Umberto M, Yolanda L, Carter MI, Kari AM (2020) CLINICAL COURSE AND OUTCOME OF COVID-19 ACUTE RESPIRATORY DISTRESS SYNDROME: DATA FROM A NATIONAL REPOSITORY. medRxiv. 10.1101/2020.10.16.20214130
[80] Ali H, Alshukry A, Marafie SK, et al. (2020) Outcomes of COVID-19: Disparities by ethnicity. Infect Genet Evol: 104639. 10.1016/j.meegid.2020.104639
[81] Alqahtani AM, AlMalki ZS, Alalweet RM, et al. (2020) Assessing the Severity of Illness in Patients With Coronavirus Disease in Saudi Arabia: A Retrospective Descriptive Cross-Sectional Study. Front Public Health 8: 593256. 10.3389/fpubh.2020.593256
[82] Al-Sabah S, Al-Haddad M, Al-Youha S, Jamal M, Almazeedi S (2020) COVID-19: Impact of obesity and diabetes on disease severity. Clin Obes 10(6): e12414. 10.1111/cob.12414
[83] Al-Salameh A, Lanoix JP, Bennis Y, et al. (2020) The association between body mass index class and coronavirus disease 2019 outcomes. Int J Obes (Lond): 1-6. 10.1038/s41366-020-00721-1
[84] Alsayer R, Alsharif H, Baadani A, Kalam K (2021) Clinical and Epidemiological Characteristics of COVID-19 Mortality in Saudi Arabia: A Retrospective Multi-Center Study. ResearchSquare. 10.21203/rs.3.rs-276027/v1
[85] Alshukry A, Ali H, Ali Y, et al. (2020) Clinical characteristics of coronavirus disease 2019 (COVID-19) patients in Kuwait. PLoS One 15(11): e0242768. 10.1371/journal.pone.0242768
[86] Alshukry A, Bu Abbas M, Ali Y, et al. (2021) Clinical characteristics and outcomes of COVID-19 patients with diabetes mellitus in Kuwait. Heliyon 7(4): e06706. 10.1016/j.heliyon.2021.e06706
[87] Altunok ES, Alkan M, Kamat S, et al. (2020) Clinical characteristics of adult patients hospitalized with laboratory-confirmed COVID-19 pneumonia. J Infect Chemother. 10.1016/j.jiac.2020.10.020
[88] Amaouche S, Letaief Z, Buls N, Allard S, Mey J (2020) The relation between ACEI/ARB use and COVID-19 severity in RT-PCR–confirmed cases: A retrospective case-control study. ResearchSquare. 10.21203/rs.3.rs-33177/v1
[89] Amit P, Parloop B, Surabhi M, et al. (2020) A Comparative COVID 19 Characterizations and Clinical Course Analysis between ICU and Non ICU Settings. medRxiv. 10.1101/2020.10.07.20208389

[90] Amitava B, Laura P, Steve H, et al. (2020) Estimating excess 1- year mortality from COVID-19 according to underlying conditions and age in England: a rapid analysis using NHS health records in 3.8 million adults. medRxiv. 10.1101/2020.03.22.20040287
[91] Andreano A, Murtas R, Tunesi S, Gervasi F, Magnoni P, Russo AG (2021) Development of a multivariable model predicting mortality risk from comorbidities in an Italian cohort of 18,286 confirmed COVID-19 cases aged 40 years or older. Epidemiol Prev 45(1-2): 100-109. 10.19191/ep21.1-2.P100.044
[92] An-Li W, Xiaobo Z, Yasmin H (2020) Comorbidity and Sociodemographic determinants in COVID-19 Mortality in an US Urban Healthcare System. medRxiv. 10.1101/2020.06.11.20128926
[93] Ann BN, Gaurav AU, Ben C, et al. (2020) Outcomes and Cardiovascular Comorbidities in a Predominantly African-American Population with COVID-19. medRxiv. 10.1101/2020.06.28.20141929
[94] Anthony S, Daniel MB, Rebecca B, et al. (2020) Pre-existing cardiovascular disease rather than cardiovascular risk factors drives mortality in COVID-19. medRxiv. 10.1101/2020.12.02.20242933
[95] Antoni S-A, Belchin K, Minerva M-H, et al. (2020) PROGNOSTIC FACTORS IN SPANISH COVID-19 PATIENTS: A CASE SERIES FROM BARCELONA. medRxiv. 10.1101/2020.06.18.20134510
[96] Aoun M, Khalil R, Mahfoud W, et al. (2021) Age and multimorbidities as poor prognostic factors for COVID-19 in hemodialysis: a Lebanese national study. BMC Nephrol 22(1): 73. 10.1186/s12882-021-02270-9
[97] Arnaud DE, Apoline SK, Risgou OA, et al. (2021) Predictors of severe hypoxemia among COVID-19 patients in Burkina Faso (West Africa): findings from hospital based cross-sectional study. Int J Infect Dis. 10.1016/j.ijid.2021.04.007
[98] Arvinte C, Singh M, Marik PE (2020) Serum Levels of Vitamin C and Vitamin D in a Cohort of Critically Ill COVID-19 Patients of a North American Community Hospital Intensive Care Unit in May 2020: A Pilot Study. Med Drug Discov 8: 100064. 10.1016/j.medidd.2020.100064
[99] Asare S, Sandio A, Opara IN, et al. (2020) Higher Obesity Trends Among African Americans Are Associated with Increased Mortality in Infected COVID-19 Patients Within the City of Detroit. SN Compr Clin Med: 1-3. 10.1007/s42399-020-00385-y
[100] Ayan S, Mohammad Moinul A, Tarek-Ul Q, et al. (2020) CHARACTERISTICS, MANAGEMENT AND OUTCOMES OF CRITICALLY ILL COVID-19 PATIENTS ADMITTED TO ICU IN HOSPITALS IN BANGLADESH: A RETROSPECTIVE STUDY. medRxiv. 10.1101/2020.09.24.20201285
[101] Ayaz A, Arshad A, Malik H, Ali H, Hussain E, Jamil B (2020) Risk factors for intensive care unit admission and mortality in hospitalized COVID-19 patients. Acute Crit Care. 10.4266/acc.2020.00381
[102] Azoulay E, Fartoukh M, Darmon M, et al. (2020) Increased mortality in patients with severe SARS-CoV-2 infection admitted within seven days of disease onset. Intensive Care Medicine 46(9): 1714-1722. 10.1007/s00134-020-06202-3
[103] Azwar MK, Setiati S, Rizka A, Fitriana I, Saldi SRF, Safitri ED (2020) Clinical Profile of Elderly Patients with COVID-19 Hospitalized in Indonesia's National General Hospital. Acta Medica Indonesiana 52(3): 199-205
[104] Badawi A, Vasileva D (2021) Comparative profile for COVID-19 cases from China and North America: Clinical symptoms, comorbidities and disease biomarkers. World J Clin Cases 9(1): 118-132. 10.12998/wjcc.v9.i1.118
[105] Bae JH, Choi SK, Kim NH, Lee J, Kim SG (2021) Use of Renin-Angiotensin-Aldosterone System Inhibitors and Severe COVID-19 Outcomes in Patients with Hypertension: A Nationwide Cohort Study. Diabetes Metab J. 10.4093/dmj.2020.0279
[106] Banerjee A, Chen S, Pasea L, et al. (2021) Excess deaths in people with cardiovascular diseases during the COVID-19 pandemic. Eur J Prev Cardiol. 10.1093/eurjpc/zwaa155
[107] Baqi S, Naz A, Sayeed MA, et al. (2021) Clinical Characteristics and Outcome of Patients With Severe COVID-19 Pneumonia at a Public Sector Hospital in Karachi, Pakistan. Cureus 13(2): e13107. 10.7759/cureus.13107
[108] Barbu MG, Thompson RJ, Thompson DC, Cretoiu D, Suciu N (2020) The Impact of SARS-CoV-2 on the Most Common Comorbidities-A Retrospective Study on 814 COVID-19 Deaths in Romania. Frontiers in Medicine 7. 10.3389/fmed.2020.567199
[109] Barlass U, Wiliams B, Dhana K, et al. (2020) Marked Elevation of Lipase in COVID-19 Disease: A Cohort Study. Clin Transl Gastroenterol 11(7): e00215-e00215
[110] Barry M, AlMohaya A, AlHijji A, et al. (2020) Clinical Characteristics and Outcome of Hospitalized COVID-19 Patients in a MERS-CoV Endemic Area. J Epidemiol Glob Health 10(3): 214-221. 10.2991/jegh.k.200806.002
[111] Bashar F, Haytham Abu K, Dolev P, et al. (2020) Abnormal Baseline liver function tests are associated with death or mechanical ventilation in COVID-19. 10.22541/AU.160396940.06060173/V1

[112] Bauer AZ, Gore R, Sama SR, et al. (2020) Hypertension, medications, and risk of severe COVID-19: A Massachusetts community-based observational study. J Clin Hypertens (Greenwich). 10.1111/jch.14101
[113] Bavaro DF, Diella L, Fabrizio C, et al. (2021) Peculiar clinical presentation of COVID-19 and predictors of mortality in the elderly: A multicentre retrospective cohort study. Int J Infect Dis 105: 709-715. 10.1016/j.ijid.2021.03.021
[114] Bellini B, Cresci B, Cosentino C, et al. (2021) Obesity as a risk factor for hospitalization in COronaVirus Disease-19 (COVID-19) patients: Analysis of the Tuscany regional database. Nutrition Metabolism and Cardiovascular Diseases 31(3): 769-773. 10.1016/j.numecd.2020.11.030
[115] Bepouka BI, Mandina M, Makulo JR, et al. (2020) Predictors of mortality in COVID-19 patients at Kinshasa University Hospital, Democratic Republic of the Congo, from March to June 2020. Pan Afr Med J 37: 105. 10.11604/pamj.2020.37.105.25279
[116] Biscarini S, Colaneri M, Ludovisi S, et al. (2020) The obesity paradox: Analysis from the SMAtteo COvid-19 REgistry (SMACORE) cohort. Nutrition Metabolism and Cardiovascular Diseases 30(11): 1920-1925. 10.1016/j.numecd.2020.07.047
[117] Borie R, Savale L, Dossier A, et al. (2020) Glucocorticoids with low-dose anti-IL1 anakinra rescue in severe non-ICU COVID-19 infection: A cohort study. PLoS One 15(12): e0243961. 10.1371/journal.pone.0243961
[118] Boulle A, Davies MA, Hussey H, et al. (2020) Risk factors for COVID-19 death in a population cohort study from the Western Cape Province, South Africa. Clin Infect Dis. 10.1093/cid/ciaa1198
[119] Bowles KH, McDonald M, Barron Y, Kennedy E, O'Connor M, Mikkelsen M (2021) Surviving COVID-19 After Hospital Discharge: Symptom, Functional, and Adverse Outcomes of Home Health Recipients. Annals of Internal Medicine 174(3): 316-+. 10.7326/m20-5206
[120] Bramante CT, Ingraham NE, Murray TA, et al. (2021) Metformin and risk of mortality in patients hospitalised with COVID-19: a retrospective cohort analysis. Lancet Healthy Longev 2(1): e34-e41. 10.1016/s2666-7568(20)30033-7
[121] Bravi F, Flacco ME, Carradori T, et al. (2020) Predictors of severe or lethal COVID-19, including Angiotensin Converting Enzyme inhibitors and Angiotensin II Receptor Blockers, in a sample of infected Italian citizens. Plos One 15(6). 10.1371/journal.pone.0235248
[122] Budweiser S, Bas S, Jörres R, et al. (2020) Patients’ treatment limitations as predictive factor for mortality in COVID-19: Results from hospitalized patients of a hotspot region for SARS-CoV-2 infections. ResearchSquare. 10.21203/rs.3.rs-113642/v1
[123] Burrell AJ, Pellegrini B, Salimi F, et al. (2020) Outcomes for patients with COVID-19 admitted to Australian intensive care units during the first four months of the pandemic. Med J Aust. 10.5694/mja2.50883
[124] Byeon KH, Kim DW, Kim J, Choi BY, Choi B, Cho KD (2021) Factors affecting the survival of early COVID-19 patients in South Korea: An observational study based on the Korean National Health Insurance big data. Int J Infect Dis 105: 588-594. 10.1016/j.ijid.2021.02.101
[125] Cagla O, Adem D, Nurcan Akbas G (2020) Epidemiological characteristics of Covid-19 patients in XXX province, in XXX. 10.22541/AU.160837979.90389790/V1
[126] Caliskan T, Saylan B (2020) Smoking and comorbidities are associated with COVID-19 severity and mortality in 565 patients treated in Turkey: a retrospective observational study. Rev Assoc Med Bras (1992) 66(12): 1679-1684. 10.1590/1806-9282.66.12.1679
[127] Calvisi Laura S, Giuseppe Alvise R, Marina S, et al. (2021) Thromboembolism risk is higher among patients with diabetes and COVID-19 and is associated to poor clinical outcome. medRxiv. 10.1101/2021.04.17.21255540
[128] Cao Y, Han X, Gu J, et al. (2020) Prognostic value of baseline clinical and HRCT findings in 101 patients with severe COVID-19 in Wuhan, China. Sci Rep 10(1): 17543. 10.1038/s41598-020-74497-9
[129] Cariou B, Pichelin M, Goronflot T, et al. (2021) Phenotypic characteristics and prognosis of newly diagnosed diabetes in hospitalized patients with COVID-19: Results from the CORONADO study. Diabetes Res Clin Pract: 108695. 10.1016/j.diabres.2021.108695
[130] Carlos Nicolás P-G, Daniel E-V, Manuel M-B, et al. (2020) Death in COVID-19. A Non-ICU Spanish Series: The SADDEN DEATH Study. SSRN. 10.2139/ssrn.3606434
[131] Carolina P, Marcos Antonio Ferreira J, Andreia Insabralde de Queiroz C, et al. (2020) Temporal analysis of the clinical evolution of confirmed cases of COVID-19 in the state of Mato Grosso do Sul - Brazil. medRxiv. 10.1101/2020.09.21.20198812
[132] Carrasco-Sanchez FJ, Lopez-Carmona MD, Martinez-Marcos FJ, et al. (2021) Admission hyperglycaemia as a predictor of mortality in patients hospitalized with COVID-19 regardless of diabetes status: data from the Spanish SEMI-COVID-19 Registry. Annals of Medicine 53(1): 103-116. 10.1080/07853890.2020.1836566

[133] Carrillo-Vega MF, Salinas-Escudero G, Garcia-Pena C, Gutierrez-Robledo LM, Parra-Rodriguez L (2020) Early estimation of the risk factors for hospitalization and mortality by COVID-19 in Mexico. Plos One 15(9). 10.1371/journal.pone.0238905
[134] Cars T, Hergens M, Bell M, et al. (2021) Risk factors for COVID-19-related death, hospitalization and intensive care: A population-wide study of all inhabitants in Stockholm. ResearchSquare. 10.21203/rs.3.rs-419368/v1
[135] Carukshi A, Nuwan W, Surangi J, et al. (2020) Sri Lanka's Successful Containment of COVID-19 Through Its Rapid and Early Response: Clinical & Epidemiological Evidence from the Initial Case Series. SSRN. 10.2139/ssrn.3687372
[136] Casas-Rojo JM, Antón-Santos JM, Millán-Núñez-Cortés J, et al. (2020) Características clínicas de los pacientes hospitalizados con COVID-19 en España: resultados del Registro SEMI-COVID-19. Rev clin esp (Ed impr)
[137] Cattelan AM, Di Meco E, Trevenzoli M, et al. (2020) Clinical characteristics and laboratory biomarkers changes in COVID-19 patients requiring or not intensive or sub-intensive care: a comparative study. BMC Infect Dis 20(1): 934. 10.1186/s12879-020-05647-7
[138] Cavayas YA, Noël A, Brunette V, et al. (2020) Early experience with critically ill patients with COVID-19 in Montreal. Can J Anaesth: 1-10. 10.1007/s12630-020-01816-z
[139] Cellina M, Gibelli D, Valenti Pittino C, Toluian T, Marino P, Oliva G (2020) Risk Factors of Fatal Outcome in Patients With COVID-19 Pneumonia. Disaster Med Public Health Prep: 1-8. 10.1017/dmp.2020.346
[140] Cesar Maquilon O, Jonas Alberto G, Monica Antolini T, et al. (2021) Clinical and Demographic Characteristics on Admission and Their Prognostic Value at Discharge in 529 Consecutive COVID-19 Patients in Santiago, Chile. Clinical Medicine & Research 10(1). 10.11648/J.CMR.20211001.11
[141] Chaibi S, Boussier J, Hajj WE, et al. (2020) Liver function test abnormalities are associated with a poorer prognosis in Covid-19 patients: Results of a French cohort. Clin Res Hepatol Gastroenterol. 10.1016/j.clinre.2020.10.002
[142] Chakravarty D, Ratnani P, Sobotka S, et al. (2021) Increased Hospitalization and Mortality from COVID-19 in Prostate Cancer Patients. Cancers 13(7). 10.3390/cancers13071630
[143] Chang M, Park Y, Kim B, Park D (2020) Risk Factors for Progression to Patients with Severe Stage of COVID-19. ResearchSquare. 10.21203/rs.3.rs-26356/v1
[144] Chang MC, Hwang JM, Jeon JH, Kwak SG, Park D, Moon JS (2020) Fasting Plasma Glucose Level Independently Predicts the Mortality of Patients with Coronavirus Disease 2019 Infection: A Multicenter, Retrospective Cohort Study. Endocrinol Metab (Seoul) 35(3): 595-601. 10.3803/EnM.2020.719
[145] Chang MH, Moonesinghe R, Truman BI (2021) COVID-19 Hospitalization by Race and Ethnicity: Association with Chronic Conditions Among Medicare Beneficiaries, January 1-September 30, 2020. J Racial Ethn Health Disparities: 1-10. 10.1007/s40615-020-00960-y
[146] Huang CL, Wang YM, Li XW, et al. (2020) Clinical features of patients infected with 2019 novel coronavirus in Wuhan, China. Lancet 395(10223): 497-506. 10.1016/s0140-6736(20)30183-5
[147] Chen K, Lei Y, He Y, et al. (2021) Clinical Outcomes of Hospitalized COVID-19 Patients with Renal Injury: A multi-hospital observational study from Wuhan. ResearchSquare. 10.21203/rs.3.rs-239498/v1
[148] Chen W, Hu C, Huang L, et al. (2020) Epidemiological and clinical characteristics of COVID-19 patients in Nanjing. ResearchSquare. 10.21203/rs.3.rs-18007/v1
[149] Chen X, Chen P, Djakpo D, Lin Y, Zhang R, Wang Z (2020) Clinical characteristics of 208 patients with COVID-19 in a surrounding city of Wuhan, China. ResearchSquare. 10.21203/rs.3.rs-20783/v1
[150] Huang J, Cheng A, Lin S, Zhu Y, Chen G (2020) Individualized prediction nomograms for disease progression in mild COVID-19. J med virol
[151] Cheng Q, Liao Y, Lin Y, et al. (2020) Clinical characteristics and prognostic factors in elderly patients with COVID-19. ResearchSquare. 10.21203/rs.3.rs-31123/v1
[152] Chetboun M, Raverdy V, Labreuche J, et al. (2021) BMI and pneumonia outcomes in critically ill COVID-19 patients: an international multicenter study. Obesity (Silver Spring). 10.1002/oby.23223
[153] Chilimuri S, Sun HZ, Alemam A, et al. (2020) Predictors of Mortality in Adults Admitted with COVID-19: Retrospective Cohort Study from New York City. Western Journal of Emergency Medicine 21(4): 779-784. 10.5811/westjem.2020.6.47919
[154] Chishinga N, Gandhi NR, Onwubiko UN, et al. (2020) Characteristics and Risk Factors for Hospitalization and Mortality among Persons with COVID-19 in Atlanta Metropolitan Area. medRxiv. 10.1101/2020.12.15.20248214
[155] Christian Omar R-P, Erika Areli R-G, Cristina Elizabeth M-M, et al. (2020) Laboratory Parameters and Complication Risk Factors in Mexican COVID-19 Patients: A Cross-Sectional Study. SSRN. 10.2139/ssrn.3634850

[156] Collaborative TO, Elizabeth W, Alex JW, et al. (2020) OpenSAFELY: factors associated with COVID-19-related hospital death in the linked electronic health records of 17 million adult NHS patients. medRxiv. 10.1101/2020.05.06.20092999
[157] Collard D, Nurmohamed NS, Kaiser Y, et al. (2021) Cardiovascular risk factors and COVID-19 outcomes in hospitalised patients: a prospective cohort study. BMJ Open 11(2): e045482. 10.1136/bmjopen-2020-045482
[158] Coppelli A, Giannarelli R, Aragona M, et al. (2020) Hyperglycemia at Hospital Admission Is Associated With Severity of the Prognosis in Patients Hospitalized for COVID-19: The Pisa COVID-19 Study. Diabetes Care 43(10): 2345-2348. 10.2337/dc20-1380
[159] Cugno M, Gualtierotti R, Casazza G, et al. (2021) Mortality in Patients with COVID-19 on Renin Angiotensin System Inhibitor Long-Term Treatment: An Observational Study Showing that Things Are Not Always as They Seem. Adv Ther: 1-8. 10.1007/s12325-021-01704-y
[160] Cui X, Gallini JW, Jasien CL, Mrug M (2020) Autosomal Dominant Polycystic Kidney Disease does not significantly alter major COVID-19 outcomes among veterans. medRxiv. 10.1101/2020.11.25.20238675
[161] Cummins L, Ebyarimpa I, Cheetham N, Tzortziou Brown V, Brennan K, Panovska-Griffiths J (2021) Factors associated with COVID-19 related hospitalisation, critical care admission and mortality using linked primary and secondary care data. Influenza Other Respir Viruses. 10.1111/irv.12864
[162] Hui H, Yingqian Z, Xin Y, et al. (2020) Clinical and radiographic features of cardiac injury in patients with 2019 novel coronavirus pneumonia. medRxiv. 10.1101/2020.02.24.20027052
[163] Dai CL, Kornilov SA, Roper RT, et al. (2021) Characteristics and Factors Associated with COVID-19 Infection, Hospitalization, and Mortality Across Race and Ethnicity. medRxiv. 10.1101/2020.10.14.20212803
[164] Dalvir K, Irina Chis S, Alexander S, et al. (2020) The Characteristics, Dynamics, and the Risk of Death in COVID-19 Positive Dialysis Patients in London, UK. Kidney 1(11). 10.34067/KID.0004502020
[165] Darquennes G, Le Corre P, Le Moine O, Loas G (2021) Association between Functional Inhibitors of Acid Sphingomyelinase (FIASMAs) and Reduced Risk of Death in COVID-19 Patients: A Retrospective Cohort Study. Pharmaceuticals (Basel) 14(3). 10.3390/ph14030226
[166] David De L, Yori R, Nicolas B, et al. (2020) Clinical and Demographic Characteristics of COVID-19 Patients Admitted in a Tertiary Care Hospital in the Dominican Republic. medRxiv. 10.1101/2020.12.11.20247437
[167] de Jong M, Woodward M, Peters SAE (2020) Diabetes and COVID-19-Related Mortality in Women and Men in the UK Biobank: Comparisons With Influenza/Pneumonia and Coronary Heart Disease. Diabetes Care. 10.2337/dc20-2378
[168] De Smet D, De Smet K, Herroelen P, Gryspeerdt S, Martens GA (2020) Serum 25(OH)D Level on Hospital Admission Associated With COVID-19 Stage and Mortality. Am J Clin Pathol. 10.1093/ajcp/aqaa252
[169] de Souza CD, de Arruda Magalhães AJ, Lima AJ, et al. (2020) Clinical manifestations and factors associated with mortality from COVID-19 in older adults: Retrospective population-based study with 9807 older Brazilian COVID-19 patients. Geriatr Gerontol Int 20(12): 1177-1181. 10.1111/ggi.14061
[170] de Souza CDF, Leal TC, Santos LG (2020) Circulatory System Diseases in Patients with COVID-19: Description of Clinical and Epidemiological Profile of 197 Deaths. Arquivos Brasileiros De Cardiologia 115(2): 281-283. 10.36660/abc.20200453
[171] de Souza FSH, Hojo-Souza NS, Batista BDO, da Silva CM, Guidoni DL (2021) On the analysis of mortality risk factors for hospitalized COVID-19 patients: A data-driven study using the major Brazilian database. PLoS One 16(3): e0248580. 10.1371/journal.pone.0248580
[172] Deeb A, Khawaja K, Sakrani N, et al. (2021) Impact of Ethnicity and Underlying Comorbidity on COVID-19 Inhospital Mortality: An Observational Study in Abu Dhabi, UAE. Biomed Res Int 2021: 6695707. 10.1155/2021/6695707
[173] Deng F, Zhang L, Lyu L, et al. (2021) Increased levels of ferritin on admission predicts intensive care unit mortality in patients with COVID-19. Med Clin (Engl Ed) 156(7): 324-331. 10.1016/j.medcle.2020.11.015
[174] Dennis JM, Mateen BA, Sonabend R, et al. (2020) Type 2 Diabetes and COVID-19-Related Mortality in the Critical Care Setting: A National Cohort Study in England, March-July 2020. Diabetes Care. 10.2337/dc20-1444
[175] Dennison D, Al Khabori M, Al Mamari S, et al. (2021) Circulating activated neutrophils in COVID-19: An independent predictor for mechanical ventilation and death. Int J Infect Dis 106: 155-159. 10.1016/j.ijid.2021.03.066
[176] Denova-Gutierrez E, Lopez-Gatell H, Alomia-Zegarra JL, et al. (2020) The Association of Obesity, Type 2 Diabetes, and Hypertension with Severe Coronavirus Disease 2019 on Admission Among Mexican Patients. Obesity 28(10): 1826-1832. 10.1002/oby.22946

[177] Di Castelnuovo A, Bonaccio M, Costanzo S, et al. (2020) Common cardiovascular risk factors and in-hospital mortality in 3,894 patients with COVID-19: survival analysis and machine learning-based findings from the multicentre Italian CORIST Study. Nutrition Metabolism and Cardiovascular Diseases 30(11): 1899-1913. 10.1016/j.numecd.2020.07.031
[178] Didier C, Nick SN, Yannick K, et al. (2020) Cardiovascular risk factors are independently associated with COVID-19 mortality: a prospective cohort study. medRxiv. 10.1101/2020.10.01.20205229
[179] Dimitrios G, Evangelia M, Xenia P, Kostantinos S, Kriton-Ioannis R, Petros G (2020) Predictors of mortality in hospitalized COVID-19 patients in Athens, Greece. medRxiv. 10.1101/2020.10.12.20211193
[180] Dionita Rani K, Anggi Lukman W (2020) Current prevalence, characteristics, and comorbidities of patients with COVID-19 in Indonesia. 3(2). 10.22146/JCOEMPH.57325
[181] Dosi R, Jain G, Mehta A (2020) Clinical Characteristics, Comorbidities,and Outcome among 365 Patients of Coronavirus Disease 2019 at a Tertiary Care Centre in Central India. J Assoc Physicians India 68(9): 20-23
[182] Drozd M, Pujades-Rodriguez M, Lillie PJ, et al. (2021) Non-communicable disease, sociodemographic factors, and risk of death from infection: a UK Biobank observational cohort study. Lancet Infect Dis. 10.1016/s1473-3099(20)30978-6
[183] Ebekozien O, Agarwal S, Noor N, et al. (2021) Inequities in Diabetic Ketoacidosis Among Patients With Type 1 Diabetes and COVID-19: Data From 52 US Clinical Centers. J Clin Endocrinol Metab 106(4): e1755-e1762. 10.1210/clinem/dgaa920
[184] Eduardo O, Amay P, Arnaldo L-R, et al. (2020) ICU Outcomes and Survival in Patients with Severe COVID-19 in the Largest Health Care System in Central Florida. medRxiv. 10.1101/2020.08.25.20181909
[185] Edward B, Cristian T, Sergio F-B, et al. (2020) The natural history of symptomatic COVID-19 in Catalonia, Spain: a multi-state model including 109,367 outpatient diagnoses, 18,019 hospitalisations, and 5,585 COVID-19 deaths among 5,627,520 people. medRxiv. 10.1101/2020.07.13.20152454
[186] Efren M-Z, Carlos MH-S (2020) Survival in adult inpatients with COVID-19. medRxiv. 10.1101/2020.05.25.20110684
[187] Efren M-Z, Oliver M-C, Ivan D-E, Carlos MH-S (2020) Predictors of severe symptomatic laboratory-confirmed SARS-COV-2 reinfection. medRxiv. 10.1101/2020.10.14.20212720
[188] El Aidaoui K, Haoudar A, Khalis M, et al. (2020) Predictors of Severity in Covid-19 Patients in Casablanca, Morocco. Cureus 12(9): e10716. 10.7759/cureus.10716
[189] ElAbd R, AlTarrah D, AlYouha S, et al. (2021) Angiotensin-Converting Enzyme (ACE) Inhibitors and Angiotensin Receptor Blockers (ARB) Are Protective Against ICU Admission and Mortality for Patients With COVID-19 Disease. Front Med (Lausanne) 8: 600385. 10.3389/fmed.2021.600385
[190] Elizabeth S, Suzy G, Chris M, et al. (2020) Ethnicity and risk of death in patients hospitalised for COVID-19 infection: an observational cohort study in an urban catchment area. medRxiv. 10.1101/2020.05.05.20092296
[191] El-Jawahri A, Bohossian HB, Paasche-Orlow MK, et al. (2021) Clinical Outcomes of Patients Hospitalized with Coronavirus Disease 2019 (COVID-19) in Boston. J Gen Intern Med: 1-7. 10.1007/s11606-021-06622-x
[192] Elliott J, Bodinier B, Whitaker M, et al. (2021) COVID-19 mortality in the UK Biobank cohort: revisiting and evaluating risk factors. Eur J Epidemiol 36(3): 299-309. 10.1007/s10654-021-00722-y
[193] El-Solh AA, Meduri UG, Lawson Y, Carter M, Mergenhagen KA (2021) Clinical Course and Outcome of COVID-19 Acute Respiratory Distress Syndrome: Data From a National Repository. J Intensive Care Med: 885066621994476. 10.1177/0885066621994476
[194] Emad K, Kareem I, Abdallah A, et al. (2020) Predictive Factors of Intubation in COVID-19 Patients: A Better Answer to the Ventilator Shortage Crisis? SSRN. 10.2139/ssrn.3578762
[195] Emami A, Javanmardi F, Akbari A, et al. (2020) Survival rate in hypertensive patients with COVID-19. Clin Exp Hypertens: 1-4. 10.1080/10641963.2020.1812624
[196] Eman Mohamed k, Enas D, Aliae M-H, et al. (2020) Impact of comorbidities on COVID-19 outcome. medRxiv. 10.1101/2020.11.28.20240267
[197] Eman Mohamed k, Noha A-E, Enas D, et al. (2020) Surveillance study of acute neurological manifestations among 439 Egyptian patients with COVID-19 in Assiut and Aswan university hospitals. medRxiv. 10.1101/2020.10.28.20221879
[198] Endalkachew Hailu M, Tigist Workneh L, Ishmael Shemsedin H, et al. (2020) Predictors of Death in Severe COVID-19 Patients at Millennium COVID-19 Care Center in Ethiopia: A Case-Control Study. medRxiv. 10.1101/2020.10.07.20205575

[199] Erinoso OA, Wright KO, Anya S, et al. (2020) Clinical characteristics, predictors of symptomatic coronavirus disease 2019 and duration of hospitalisation in a cohort of 632 Patients in Lagos State, Nigeria. Niger Postgrad Med J 27(4): 285-292. 10.4103/npmj.npmj_272_20
[200] Erlina B, Ari Fahrial S, Ahmad Jabir R, et al. (2020) The emergence of COVID-19 in Indonesia: analysis of predictors of infection and mortality using independent and clustered data approaches. medRxiv. 10.1101/2020.07.10.20147942
[201] Eshrati B, Baradaran HR, Erfanpoor S, Mohazzab A, Moradi Y (2020) Investigating the factors affecting the survival rate in patients with COVID-19: A retrospective cohort study. Med J Islam Repub Iran 34: 88. 10.34171/mjiri.34.88
[202] Esme M, Koca M, Dikmeer A, et al. (2020) Older Adults With Coronavirus Disease 2019; A Nationwide Study in Turkey. J Gerontol A Biol Sci Med Sci. 10.1093/gerona/glaa219
[203] Etkin Y, Conway AM, Silpe J, et al. (2021) Acute Arterial Thromboembolism in Patients with COVID-19 in the New York City Area. Ann Vasc Surg 70: 290-294. 10.1016/j.avsg.2020.08.085
[204] Ewan C, Rebecca B, Daniel B, et al. (2020) Supplementing the National Early Warning Score (NEWS2) for anticipating early deterioration among patients with COVID-19 infection. medRxiv. 10.1101/2020.04.24.20078006
[205] Ewen MH, Annemarie BD, Benjamin B, et al. (2020) Ethnicity and Outcomes from COVID-19: The ISARIC CCP-UK Prospective Observational Cohort Study of Hospitalised Patients. SSRN. 10.2139/ssrn.3618215
[206] Fadini GP, Morieri ML, Boscari F, et al. (2020) Newly-diagnosed diabetes and admission hyperglycemia predict COVID-19 severity by aggravating respiratory deterioration. Diabetes Res Clin Pract 168: 108374. 10.1016/j.diabres.2020.108374
[207] Fagard K, Gielen E, Deschodt M, Devriendt E, Flamaing J (2020) Risk factors for severe COVID-19 disease and death in patients aged 70 and over: a retrospective observational cohort study. ResearchSquare. 10.21203/rs.3.rs-101387/v1
[208] Fahad M, Michael L, Srikant R, et al. (2020) Clinical Outcomes of Critically Ill Patients with COVID-19 by Race. medRxiv. 10.1101/2020.09.07.20190074
[209] Fan Y, Guo T, Yan F, et al. (2020) Association of Statin Use With the In-Hospital Outcomes of 2019-Coronavirus Disease Patients: A Retrospective Study. Front Med (Lausanne) 7: 584870. 10.3389/fmed.2020.584870
[210] Fang H, Liu Q, Xi M, et al. (2020) Impact of comorbidities on clinical prognosis in 1280 patients with different types of COVID-19. J Investig Med. 10.1136/jim-2020-001555
[211] Farhana A, Shun Y, John M, Xingping Q, Paing Soe L, Fred O (2020) Independent Predictors of In-Hospital Mortality in Patients with COVID-19 in London, United Kingdom: An Observational Cohort Study. SSRN. 10.2139/ssrn.3640553
[212] Fei L, Yue C, Chao G, et al. (2020) Clinical Course And Risk Factors For In-hospital Death In Critical COVID-19 In Wuhan, China. medRxiv. 10.1101/2020.09.26.20189522
[213] Felipe L, Alan XB, Serena L, et al. (2020) Clinical, Behavioral and Social Factors Associated with Racial Disparities in Hospitalized and Ambulatory COVID-19 Patients from an Integrated Health Care System in Georgia. medRxiv. 10.1101/2020.07.08.20148973
[214] Fernanda Sumika Hojo S, Natália Satchiko H-S, Ben Dêivide de Oliveira B, Cristiano Maciel da S, Daniel Ludovico G (2020) On the Analysis of Mortality Risk Factors for Hospitalized COVID-19 Patients: a Data-driven Study Using the Major Brazilian Database. medRxiv. 10.1101/2020.09.24.20200766
[215] Fernandes ND, Cummings BM, Naber CE, et al. (2020) Adult COVID-19 Patients Cared For in a Pediatric ICU Embedded in a Regional Biothreat Center: Disease Severity and Outcomes. Health Secur. 10.1089/hs.2020.0225
[216] Foy BH, Carlson JCT, Reinertsen E, et al. (2020) Association of Red Blood Cell Distribution Width With Mortality Risk in Hospitalized Adults With SARS-CoV-2 Infection. JAMA Netw Open 3(9): e2022058. 10.1001/jamanetworkopen.2020.22058
[217] Frank RC, Mendez SR, Stevenson EK, Guseh JS, Chung M, Silverman MG (2020) Obesity and the Risk of Intubation or Death in Patients With Coronavirus Disease 2019. Crit Care Med 48(11): e1097-e1101. 10.1097/ccm.0000000000004553
[218] Freites Nuñez DD, Leon L, Mucientes A, et al. (2020) Risk factors for hospital admissions related to COVID-19 in patients with autoimmune inflammatory rheumatic diseases. Ann Rheum Dis 79(11): 1393-1399
[219] Fried MW, Crawford JM, Mospan AR, et al. (2020) Patient Characteristics and Outcomes of 11,721 Patients with COVID19 Hospitalized Across the United States. Clin Infect Dis. 10.1093/cid/ciaa1268
[220] Frontera JA, Sabadia S, Lalchan R, et al. (2020) A Prospective Study of Neurologic Disorders in Hospitalized COVID-19 Patients in New York City. Neurology. 10.1212/wnl.0000000000010979

[221] Galindo J, Lutz J, Izquierdo M, Parra K, Prieto L, Carrillo J (2021) Characteristics and Clinical Course of Adult in-Patients With SARS-CoV-2 Pneumonia in Bogotá, Colombia. ResearchSquare. 10.21203/rs.3.rs-144087/v1
[222] Gao F, Zheng KI, Yan HD, et al. (2021) Association and Interaction Between Serum Interleukin-6 Levels and Metabolic Dysfunction-Associated Fatty Liver Disease in Patients With Severe Coronavirus Disease 2019. Front Endocrinol (Lausanne) 12: 604100. 10.3389/fendo.2021.604100
[223] Gao M, Piernas C, Astbury NM, et al. (2021) Associations between body-mass index and COVID-19 severity in 6·9 million people in England: a prospective, community-based, cohort study. Lancet Diabetes Endocrinol. 10.1016/s2213-8587(21)00089-9
[224] Gault N, Esposito-Farese M, Revest M, et al. (2021) Chronic use of Renin-Angiotensin-Aldosterone-System blockers and mortality in COVID-19: a multicenter prospective cohort and literature review. Fundam Clin Pharmacol. 10.1111/fcp.12683
[225] Gazzaruso C, Paolozzi E, Valenti C, et al. (2020) Association between antithrombin and mortality in patients with COVID-19. A possible link with obesity. Nutrition Metabolism and Cardiovascular Diseases 30(11): 1914-1919. 10.1016/j.numecd.2020.07.040
[226] Ge C, Jiang Y, Peng Q, Wu L, Duan K, Yang J (2020) Clinical characteristics of hospitalized patients with COVID-19 in Yueyang, Hunan, China. ResearchSquare. 10.21203/rs.3.rs-37583/v1
[227] Ge Y, Sun S, Shen Y (2021) Estimation of case-fatality rate in COVID-19 patients with hypertension and diabetes mellitus in the New York state: a preliminary report. Epidemiol Infect 149: e14. 10.1017/s0950268821000066
[228] Geng MJ, Wang LP, Ren X, et al. (2021) Risk factors for developing severe COVID-19 in China: an analysis of disease surveillance data. Infect Dis Poverty 10(1): 48. 10.1186/s40249-021-00820-9
[229] Genny C, Nina Mendez D, Kassandra DSZ, et al. (2020) Clinical course and severity outcome indicators among COVID 19 hospitalized patients in relation to comorbidities distribution Mexican cohort. medRxiv. 10.1101/2020.07.31.20165480
[230] Georges JL, Cochet H, Roger G, et al. (2020) [Association of hypertension and antihypertensive agents and the severity of COVID-19 pneumonia. A monocentric French prospective study]. Ann Cardiol Angeiol (Paris) 69(5): 247-254. 10.1016/j.ancard.2020.09.030
[231] Gerotziafas GT, Sergentanis TN, Voiriot G, et al. (2020) Derivation and Validation of a Predictive Score for Disease Worsening in Patients with COVID-19. Thromb Haemost. 10.1055/s-0040-1716544
[232] Ghada Omer Hamad Abd E-R, Maysoun Ahmed Awad Y, Doaa Salih Ibrahim M (2021) Prediction of COVID-19 mortality among hospitalized patients in Sudan. medRxiv. 10.1101/2021.03.09.21253179
[233] Gharebaghi N, Farshid S, Boroofeh B, et al. (2021) Evaluation of epidemiology, clinical features, prognosis, diagnosis and treatment outcomes of patients with COVID-19 in West Azerbaijan Province. Int J Clin Pract: e14108. 10.1111/ijcp.14108
[234] Giannis D, Allen S, Tsang J, et al. (2021) Post-Discharge Thromboembolic Outcomes and Mortality of Hospitalized COVID-19 Patients: The CORE-19 Registry. Blood. 10.1182/blood.2020010529
[235] Giannouchos TV, Sussman RA, Mier JM, Poulas K, Farsalinos K (2020) Characteristics and risk factors for COVID-19 diagnosis and adverse outcomes in Mexico: an analysis of 89,756 laboratory-confirmed COVID-19 cases. Eur respir j
[236] Gillian S, Michael M, Trevor AS, John W, Tom L (2021) Ethnicity, pre-existing comorbidities, and outcomes of hospitalised patients with COVID-19. 6. 10.12688/WELLCOMEOPENRES.16580.1
[237] Girardin JL, Seixas A, Ramos Cejudo J, et al. (2021) Contribution of pulmonary diseases to COVID-19 mortality in a diverse urban community of New York. Chron Respir Dis 18: 1479973120986806. 10.1177/1479973120986806
[238] Gobbato M, Clagnan E, Burba I, et al. (2020) Clinical, demographical characteristics and hospitalisation of 3,010 patients with Covid-19 in Friuli Venezia Giulia Region (Northern Italy). A multivariate, population-based, statistical analysis. Epidemiol Prev 44(5-6 Suppl 2): 226-234. 10.19191/ep20.5-6.S2.122
[239] Goel N, Spalgais S, Mrigpuri P, Khanna M, Menon B, Kumar R (2020) Characteristics of COVID-19 at a non-COVID tertiary pulmonary care centre in Delhi, India. Monaldi Arch Chest Dis 90(4). 10.4081/monaldi.2020.1568
[240] Gok M, Cetinkaya H, Kandemir T, et al. (2021) Chronic kidney disease predicts poor outcomes of COVID-19 patients. Int Urol Nephrol: 1-8. 10.1007/s11255-020-02758-7
[241] Golpe R, Pérez-de-Llano LA, Dacal D, Guerrero-Sande H, Pombo-Vide B, Ventura-Valcárcel P (2020) Risk of severe COVID-19 in hypertensive patients treated with renin-angiotensin-aldosterone system inhibitors. Med Clin (Engl Ed) 155(11): 488-490. 10.1016/j.medcle.2020.06.028

[242] Gómez AM, Henao DC, Muñoz OM, et al. (2021) Glycemic control metrics using flash glucose monitoring and hospital complications in patients with COVID-19. Diabetes Metab Syndr 15(2): 499-503. 10.1016/j.dsx.2021.02.008
[243] Gómez Antúnez M, Muiño Míguez A, Bendala Estrada AD, et al. (2020) Clinical Characteristics and Prognosis of COPD Patients Hospitalized with SARS-CoV-2. Int J Chron Obstruct Pulmon Dis 15: 3433-3445. 10.2147/copd.S276692
[244] Gonçalves TJM, Gonçalves S, Guarnieri A, et al. (2020) Prevalence of obesity and hypovitaminosis D in elderly with severe acute respiratory syndrome coronavirus 2 (SARS-CoV-2). Clin Nutr ESPEN 40: 110-114. 10.1016/j.clnesp.2020.10.008
[245] Goodman KE, Magder LS, Baghdadi JD, et al. (2020) Impact of Sex and Metabolic Comorbidities on COVID-19 Mortality Risk Across Age Groups: 66,646 Inpatients Across 613 U.S. Hospitals. Clin Infect Dis. 10.1093/cid/ciaa1787
[246] Gottlieb M, Sansom S, Frankenberger C, Ward E, Hota B (2020) Clinical Course and Factors Associated With Hospitalization and Critical Illness Among COVID-19 Patients in Chicago, Illinois. Acad Emerg Med 27(10): 963-973
[247] Gouri K, Sriya G, Maria D, et al. (2020) Clinical characteristics and Outcomes of 500 patients with COVID Pneumonia : Results from a Single center(Southend University Hospital). medRxiv. 10.1101/2020.08.13.20163030
[248] Grasselli G, Greco M, Zanella A, et al. (2020) Risk Factors Associated With Mortality Among Patients With COVID-19 in Intensive Care Units in Lombardy, Italy. Jama Internal Medicine 180(10): 1345-1355. 10.1001/jamainternmed.2020.3539
[249] Grippo F, Navarra S, Orsi C, et al. (2020) The Role of COVID-19 in the Death of SARS-CoV-2-Positive Patients: A Study Based on Death Certificates. J Clin Med 9(11). 10.3390/jcm9113459
[250] Grivas P, Khaki AR, Wise-Draper TM, et al. (2021) Association of Clinical Factors and Recent Anti-Cancer Therapy with COVID-19 Severity among Patients with Cancer: A Report from the COVID-19 and Cancer Consortium. Annals of oncology : official journal of the European Society for Medical Oncology. 10.1016/j.annonc.2021.02.024
[251] Gu T, Chu Q, Yu Z, et al. (2020) History of coronary heart disease increased the mortality rate of patients with COVID-19: a nested case-control study. BMJ Open 10(9): e038976. 10.1136/bmjopen-2020-038976
[252] Gu T, Mack JA, Salvatore M, et al. (2020) Characteristics Associated With Racial/Ethnic Disparities in COVID-19 Outcomes in an Academic Health Care System. Jama Network Open 3(10). 10.1001/jamanetworkopen.2020.25197
[253] Guisado-Clavero M, Gil A, Álvarez M, et al. (2020) Clinical Characteristics of SARS-CoV-2 pneumonia diagnosed in a primary care practice in Madrid (Spain). ResearchSquare. 10.21203/rs.3.rs-42357/v2
[254] Guisado-Vasco P, Valderas-Ortega S, Carralón-González MM, et al. (2020) Clinical characteristics and outcomes among hospitalized adults with severe COVID-19 admitted to a tertiary medical center and receiving antiviral, antimalarials, glucocorticoids, or immunomodulation with tocilizumab or cyclosporine: A retrospective observational study (COQUIMA cohort). EClinicalMedicine 28: 100591. 10.1016/j.eclinm.2020.100591
[255] Gul N, Usman U, Ahmed U, Ali M, Shaukat A, Imran MM (2021) Clinical characteristics and outcomes of COVID-19 pneumonia patients from an intensive care unit in Faisalabad, Pakistan. International journal of clinical practice: e14152. 10.1111/ijcp.14152
[256] Gulseth HL, Helland E, Johansen KI, et al. (2020) Deaths after confirmed SARS-CoV-2 in Norway. Tidsskr Nor Laegeforen 140(18). 10.4045/tidsskr.20.0693
[257] Günal Ö, Türe E, Bayburtlu M, et al. (2020) [Evaluation of Patients Diagnosed with COVID-19 in Terms of Risk Factors]. Mikrobiyol Bul 54(4): 575-582. 10.5578/mb.69811
[258] Guozhi X, Ming W, Yanru H, et al. (2020) Pro-Inflammatory Cytokines Are Associated with Cardiac Injury Among Critically Ill Patients with COVID-19. SSRN. 10.2139/ssrn.3615916
[259] Gupta A, Nayan N, Nair R, et al. (2021) Diabetes Mellitus and Hypertension Increase Risk of Death in Novel Corona Virus Patients Irrespective of Age: a Prospective Observational Study of Co-morbidities and COVID-19 from India. SN Compr Clin Med: 1-8. 10.1007/s42399-021-00851-1
[260] Gupta N, Ish P, Kumar R, et al. (2020) Evaluation of the clinical profile, laboratory parameters and outcome of two hundred COVID-19 patients from a tertiary centre in India. Monaldi Arch Chest Dis 90(4). 10.4081/monaldi.2020.1507
[261] Gupta R, Agrawal R, Bukhari Z, et al. (2021) Higher comorbidities and early death in hospitalized African-American patients with Covid-19. BMC Infect Dis 21(1): 78. 10.1186/s12879-021-05782-9
[262] Gupta S, Coca SG, Chan L, et al. (2020) AKI Treated with Renal Replacement Therapy in Critically Ill Patients with COVID-19. J Am Soc Nephrol. 10.1681/asn.2020060897

[263] Gupta S, Wang W, Hayek SS, et al. (2020) Association Between Early Treatment With Tocilizumab and Mortality Among Critically Ill Patients With COVID-19. JAMA Intern Med. 10.1001/jamainternmed.2020.6252
[264] Gutierrez JP, Bertozzi SM (2020) Non-communicable diseases and inequalities increase risk of death among COVID-19 patients in Mexico. PLoS One 15(10): e0240394. 10.1371/journal.pone.0240394
[265] Guyi W, Chenfang W, Quan Z, et al. (2020) 2019å ç¶çæ¯çèå¹´æ£èçä¸´åºç¹ç¹åéççå±é©å ç´ ./ 2019å ç¶çæ¯çèå¹´æ£èçä¸´åºç¹ç¹åéççå±é©å ç´ ./ Clinical characteristics and the risk factors for severe events of elderly coronavirus disease 2019 patients. 45(5)
[266] Gvozdanović Z, Farčić N, Šimić H, et al. (2021) The Impact of Education, COVID-19 and Risk Factors on the Quality of Life in Patients with Type 2 Diabetes. Int J Environ Res Public Health 18(5). 10.3390/ijerph18052332
[267] Haase N, Plovsing R, Christensen S, et al. (2021) Characteristics, interventions, and longer term outcomes of COVID-19 ICU patients in Denmark-A nationwide, observational study. Acta Anaesthesiologica Scandinavica 65(1): 68-75. 10.1111/aas.13701
[268] Hafiz M, Icksan AG, Harlivasari AD, Aulia R, Susanti F, Eldinia L (2020) Clinical, Radiological Features and Outcome of COVID-19 patients in a Secondary Hospital in Jakarta, Indonesia. J Infect Dev Ctries 14(7): 750-757. 10.3855/jidc.12911
[269] Hägg S, Jylhävä J, Wang Y, et al. (2020) Age, Frailty, and Comorbidity as Prognostic Factors for Short-Term Outcomes in Patients With Coronavirus Disease 2019 in Geriatric Care. J Am Med Dir Assoc 21(11): 1555-1559.e1552. 10.1016/j.jamda.2020.08.014
[270] Halalau A, Odish F, Imam Z, et al. (2020) Epidemiology, Clinical Characteristics, and Outcomes of a Large Cohort of COVID-19 Outpatients in Michigan. ResearchSquare. 10.21203/rs.3.rs-30750/v1
[271] Hamad A, Abdullah A, Mohammad Bu A, et al. (2020) Clinical Characteristics and Outcomes of Diabetic COVID-19 patients in Kuwait. medRxiv. 10.1101/2020.08.20.20178525
[272] Hamer M, Gale CR, Batty GD (2020) Diabetes, glycaemic control, and risk of COVID-19 hospitalisation: Population-based, prospective cohort study. Metabolism 112: 154344. 10.1016/j.metabol.2020.154344
[273] Han D, Peng CF, Meng R, et al. (2020) Estimating the release of inflammatory factors and use of glucocorticoid therapy for COVID-19 patients with comorbidities. Aging-Us 12(22): 22413-22424
[274] Han H, Xu Z, Cheng X, et al. (2020) Descriptive, Retrospective Study of the Clinical Characteristics of Asymptomatic COVID-19 Patients. mSphere 5(5). 10.1128/mSphere.00922-20
[275] Gupta AK, Nethan ST, Mehrotra R (2020) Tobacco use as a well-recognized cause of severe COVID-19 manifestations. Respir Med 176: 106233. 10.1016/j.rmed.2020.106233
[276] Haozhe S, Ahmed A, Nikhitha M, et al. (2020) Predictors of Mortality in Adult Population Admitted with COVID-19: A Retrospective Cohort Study from New York City.
[277] Harrison SL, Fazio-Eynullayeva E, Lane DA, Underhill P, Lip GYH (2020) Comorbidities associated with mortality in 31,461 adults with COVID-19 in the United States: A federated electronic medical record analysis. PLoS Med 17(9): e1003321. 10.1371/journal.pmed.1003321
[278] Harthi S, AlOsali M, Ismaili R, et al. (2020) Clinical characteristics of confirmed cases of COVID-19 admitted at Al-Nahdha hospital, Oman: a cross-sectional descriptive study. ResearchSquare. 10.21203/rs.3.rs-39988/v1
[279] Hashemi N, Viveiros K, Redd WD, et al. (2020) Impact of Chronic Liver Disease on Outcomes of Hospitalized Patients with COVID-19: A Multicenter United States Experience. Liver international : official journal of the International Association for the Study of the Liver 40(10): 2515-2521. 10.1111/liv.14583
[280] Hashim MJ, Alsuwaidi AR, Khan G (2020) Population Risk Factors for COVID-19 Mortality in 93 Countries. J Epidemiol Glob Health 10(3): 204-208. 10.2991/jegh.k.200721.001
[281] Hassan A, Yasser A, Fadi K, et al. (2020) The Outcome of COVID-19 Patients with Acute Myocardial Infarction. medRxiv. 10.1101/2020.07.21.20156349
[282] He H, Jin Z, Ren Y, et al. (2020) Clinical Course and Features of Critical Patients with COVID-19: A Single- Center, Retrospective Study from Wuhan Huoshenshan Hospital. ResearchSquare. 10.21203/rs.3.rs-89722/v1
[283] Hernandez-Galdamez DR, Gonzalez-Block MA, Romo-Duenas DK, et al. (2020) Increased Risk of Hospitalization and Death in Patients with COVID-19 and Pre-existing Noncommunicable Diseases and Modifiable Risk Factors in Mexico. Archives of Medical Research 51(7): 683-689. 10.1016/j.arcmed.2020.07.003
[284] Hernandez-Romieu AC, Adelman MW, Hockstein MA, et al. (2020) Timing of Intubation and Mortality Among Critically Ill Coronavirus Disease 2019 Patients: A Single-Center Cohort Study. Crit Care Med 48(11): e1045-e1053. 10.1097/ccm.0000000000004600

[285] Herrmann J, Adam EH, Notz Q, et al. (2020) COVID-19 Induced Acute Respiratory Distress Syndrome-A Multicenter Observational Study. Front Med (Lausanne) 7: 599533. 10.3389/fmed.2020.599533
[286] Hesong Z, Tianlu Z, Xingwei H, et al. (2020) Impact of Chronic Comorbidities on Progression and Prognosis in Patients with COVID-19: A Retrospective Cohort Study in 1031 Hospitalized Cases in Wuhan, China. medRxiv. 10.1101/2020.06.14.20125997
[287] Hilser JR, Han Y, Biswas S, et al. (2021) Association of serum HDL cholesterol and apolipoprotein A1 levels with risk of severe SARS-CoV-2 infection. Journal of lipid research 62: 100061. 10.1016/J.JLR.2021.100061
[288] Hirashima T, Arai T, Kitajima H, et al. (2021) Factors significantly associated with COVID-19 severity in symptomatic patients: A retrospective single-center study. J Infect Chemother 27(1): 76-82. 10.1016/j.jiac.2020.09.022
[289] Holman N, Knighton P, Kar P, et al. (2020) Risk factors for COVID-19-related mortality in people with type 1 and type 2 diabetes in England: a population-based cohort study. Lancet Diabetes Endocrinol 8(10): 823-833. 10.1016/s2213-8587(20)30271-0
[290] Hong Gang R, Xingyi G, Kevin B, et al. (2020) Risk Factors for ICU Admission, Mechanical Ventilation and Mortality in Hospitalized Patients with COVID-19 in Hubei, China. medRxiv. 10.1101/2020.08.31.20184952
[291] Hosseinzadeh R, Goharrizi M, Bahardoust M, et al. (2021) Should all patients with hypertension be worried about developing severe coronavirus disease 2019 (COVID-19)? Clin Hypertens 27(1): 3. 10.1186/s40885-021-00161-7
[292] Huh K, Lee R, Ji W, et al. (2020) Impact of obesity, fasting plasma glucose level, blood pressure, and renal function on the severity of COVID-19: A matter of sexual dimorphism? Diabetes Res Clin Pract 170: 108515. 10.1016/j.diabres.2020.108515
[293] Iaccarino G, Grassi G, Borghi C, et al. (2020) Age and Multimorbidity Predict Death Among COVID-19 Patients Results of the SARS-RAS Study of the Italian Society of Hypertension. Hypertension 76(2): 366-372. 10.1161/hypertensionaha.120.15324
[294] Iannelli A, Bouam S, Schneck AS, et al. (2020) The Impact of Previous History of Bariatric Surgery on Outcome of COVID-19. A Nationwide Medico-Administrative French Study. Obes Surg: 1-9. 10.1007/s11695-020-05120-z
[295] Ida Ayu K, Marina E, Md Ikhsan M, Jerry N, Nadya M (2020) The Impact of Coronavirus Disease 2019 Pandemic on People with Diabetes in Indonesia: A Cross Sectional National Scale Web-Survey. In:
[296] Iftimie S, López-Azcona AF, Vicente-Miralles M, et al. (2020) Risk factors associated with mortality in hospitalized patients with SARS-CoV-2 infection. A prospective, longitudinal, unicenter study in Reus, Spain. PLoS One 15(9): e0234452. 10.1371/journal.pone.0234452
[297] Ioannou GN, Locke E, Green P, et al. (2020) Risk Factors for Hospitalization, Mechanical Ventilation, or Death Among 10131 US Veterans With SARS-CoV-2 Infection. Jama Network Open 3(9). 10.1001/jamanetworkopen.2020.22310
[298] Islam MZ, Riaz BK, Islam A, et al. (2020) Risk factors associated with morbidity and mortality outcomes of COVID-19 patients on the 28th day of the disease course: a retrospective cohort study in Bangladesh. Epidemiol Infect 148: e263. 10.1017/s0950268820002630
[299] Jaime B, Max S, Jesus A, et al. (2020) Risk of infection and hospitalization by Covid-19 in Mexico: a case-control study. medRxiv. 10.1101/2020.05.24.20104414
[300] Jain SK, Parsanathan R, Levine SN, Bocchini JA, Holick MF, Vanchiere JA (2020) The potential link between inherited G6PD deficiency, oxidative stress, and vitamin D deficiency and the racial inequities in mortality associated with COVID-19. Free Radic Biol Med 161: 84-91. 10.1016/j.freeradbiomed.2020.10.002
[301] Jakob CEM, Borgmann S, Duygu F, et al. (2021) First results of the "Lean European Open Survey on SARS-CoV-2-Infected Patients (LEOSS)". Infection 49(1): 63-73. 10.1007/s15010-020-01499-0
[302] Jayanama K, Srichatrapimuk S, Thammavaranucupt K, et al. (2021) The association between body mass index and severity of Coronavirus Disease 2019 (COVID-19): A cohort study. PLoS One 16(2): e0247023. 10.1371/journal.pone.0247023
[303] Jegede O, Anand Raman A, Tiongson B, Garlapati PR, Hershberger J, Gayam V (2020) Clinical characteristics, hospital course, and outcomes among covid-19 positive patients with mental illness in a community hospital in new york city. International Journal of Mental Health. 10.1080/00207411.2020.1845567
[304] Jesús Ojino S-G, Alan Omar G-V, Alondra G-B, Juan Pablo R-G, Eva J-H, Octavio G-C (2020) Clinical Characteristics of Severe COVID-19 Patients in an ICU in Mexico. SSRN. 10.2139/ssrn.3605185
[305] Jia-Hui Z, Liu Y, Jing Y, et al. (2020) Clinical Characteristics and Cardiac Injury Description of 419 Cases of COVID-19 in Shenzhen, China. SSRN. 10.2139/ssrn.3556659

[306] Jiandong Z, Gary T, Sharen L, et al. (2020) Renin-angiotensin system blockers and mortality in COVID-19: a territory-wide study from Hong Kong. medRxiv. 10.1101/2020.12.21.20248645
[307] Jiandong Z, Sharen L, Xiansong W, et al. (2020) Development of a predictive risk model for severe COVID-19 disease using population-based administrative data. medRxiv. 10.1101/2020.10.21.20217380
[308] Jiang Y, Abudurexiti S, An MM, Cao D, Wei J, Gong P (2020) Risk factors associated with 28-day all-cause mortality in older severe COVID-19 patients in Wuhan, China: a retrospective observational study. Sci Rep 10(1): 22369. 10.1038/s41598-020-79508-3
[309] Joseph EE, Natalie A, Hongwei J, et al. (2020) Pre-Existing Characteristics Associated with Covid-19 Illness Severity. medRxiv. 10.1101/2020.04.29.20084533
[310] Jouffroy R, Lemoine S, Derkenne C, et al. (2020) Prehospital management of acute respiratory distress in suspected COVID-19 patients. Am J Emerg Med. 10.1016/j.ajem.2020.09.022
[311] Judit V-G, Rosa Maria V-H, Montserrat C, et al. (2020) Risk factors for SARS-CoV-2 infection, hospitalisation, and death in Catalonia, Spain: a population-based cross-sectional study. medRxiv. 10.1101/2020.08.26.20182303
[312] Jugal K, Aakanksha B, Heena, Geeta Y, Nitesh G, Ramesh M (2020) Risk Factors of COVID-19 among Patients Attending a Tertiary Care Centre in Delhi: A Case Control Study. 5(4)
[313] kaabi N, Nuaimi A, Harbi M, et al. (2020) Universal health care access for all residents reduce mortality in COVID-19 patients in Abu Dhabi, UAE: A retrospective multicenter cohort study. ResearchSquare. 10.21203/rs.3.rs-87753/v1
[314] Kaeuffer C, Le Hyaric C, Fabacher T, et al. (2020) Clinical characteristics and risk factors associated with severe COVID-19: prospective analysis of 1,045 hospitalised cases in North-Eastern France, March 2020. Euro Surveill 25(48). 10.2807/1560-7917.Es.2020.25.48.2000895
[315] Kalyanaraman Marcello R, Dolle J, Grami S, et al. (2020) Characteristics and outcomes of COVID-19 patients in New York City's public hospital system. PLoS One 15(12): e0243027. 10.1371/journal.pone.0243027
[316] Kandil E, Attia AS, Youssef MR, et al. (2020) African Americans Struggle With the Current COVID-19. Ann Surg 272(3): e187-e190. 10.1097/sla.0000000000004185
[317] Kang MK, Kim KO, Kim MC, et al. (2020) Clinical characteristics of coronavirus disease 2019 patients with diarrhea in Daegu. Korean J Intern Med 35(6): 1261-1269. 10.3904/kjim.2020.196
[318] Karagiannidis C, Mostert C, Hentschker C, et al. (2020) Case characteristics, resource use, and outcomes of 10 021 patients with COVID-19 admitted to 920 German hospitals: an observational study. Lancet Respiratory Medicine 8(9): 853-862. 10.1016/s2213-2600(20)30316-7
[319] Kayina CA, Haritha D, Soni L, et al. (2020) Epidemiological & clinical characteristics & early outcome of COVID-19 patients in a tertiary care teaching hospital in India: A preliminary analysis. Indian J Med Res 152(1 & 2): 100-104. 10.4103/ijmr.IJMR_2890_20
[320] Ken-Dror G, Wade C, Sharma S, et al. (2020) COVID-19 outcomes in UK centre within highest health and wealth band: a prospective cohort study. BMJ Open 10(11): e042090. 10.1136/bmjopen-2020-042090
[321] Khan M, Khan H, Khan S, Nawaz M (2020) Epidemiological and clinical characteristics of coronavirus disease (COVID-19) cases at a screening clinic during the early outbreak period: a single-centre study. J Med Microbiol 69(8): 1114-1123. 10.1099/jmm.0.001231
[322] Khan MS, Dogra R, Miriyala LKV, et al. (2021) Clinical characteristics and outcomes of patients with Corona Virus Disease 2019 (COVID-19) at Mercy Health Hospitals, Toledo, Ohio. PLoS One 16(4): e0250400. 10.1371/journal.pone.0250400
[323] Khorrami Z, Nili S, Sharifi H, Eybpoosh S, Shokoohi M (2020) Association of cigarette smoking, obesity, and underlying medical conditions with COVID-19 hospitalization and mortality in Iran: A nationwide retrospective ecological study. Med J Islam Repub Iran 34: 133. 10.34171/mjiri.34.133
[324] Khourssaji M, Chapelle V, Evenepoel A, et al. (2020) A biological profile for diagnosis and outcome of COVID-19 patients. Clin Chem Lab Med 58(12): 2141-2150. 10.1515/cclm-2020-0626
[325] Khunti K, Knighton P, Zaccardi F, et al. (2021) Prescription of glucose-lowering therapies and risk of COVID-19 mortality in people with type 2 diabetes: a nationwide observational study in England. Lancet Diabetes Endocrinol 9(5): 293-303. 10.1016/s2213-8587(21)00050-4
[326] Kim D, Adeniji N, Latt N, et al. (2020) Predictors of Outcomes of COVID-19 in Patients with Chronic Liver Disease: US Multi-center Study. Clin Gastroenterol Hepatol. 10.1016/j.cgh.2020.09.027
[327] Kim SR, Nam SH, Kim YR (2020) Risk Factors on the Progression to Clinical Outcomes of COVID-19 Patients in South Korea: Using National Data. Int J Environ Res Public Health 17(23). 10.3390/ijerph17238847
[328] Kim SW, Kim SM, Kim YK, et al. (2021) Clinical Characteristics and Outcomes of COVID-19 Cohort Patients in Daegu Metropolitan City Outbreak in 2020. J Korean Med Sci 36(1): e12. 10.3346/jkms.2021.36.e12

[329] Kim SY, Yoo DM, Min C, Wee JH, Kim JH, Choi HG (2020) Analysis of Mortality and Morbidity in COVID-19 Patients with Obesity Using Clinical Epidemiological Data from the Korean Center for Disease Control & Prevention. Int J Environ Res Public Health 17(24). 10.3390/ijerph17249336
[330] Kirillov Y, Timofeev S, Avdalyan A, Nikolenko VN, Gridin L, Sinelnikov MY (2021) Analysis of Risk Factors in COVID-19 Adult Mortality in Russia. J Prim Care Community Health 12: 21501327211008050. 10.1177/21501327211008050
[331] Klonoff DC, Messler JC, Umpierrez GE, et al. (2021) Association Between Achieving Inpatient Glycemic Control and Clinical Outcomes in Hospitalized Patients With COVID-19: A Multicenter, Retrospective Hospital-Based Analysis. Diabetes Care 44(2): 578-585. 10.2337/dc20-1857
[332] Ko JY, Danielson ML, Town M, et al. (2020) Risk Factors for COVID-19-associated hospitalization: COVID-19-Associated Hospitalization Surveillance Network and Behavioral Risk Factor Surveillance System. Clin Infect Dis. 10.1093/cid/ciaa1419
[333] Koh H, Moh AMC, Yeoh E, et al. (2021) Diabetes predicts severity of COVID-19 infection in a retrospective cohort: A mediatory role of the inflammatory biomarker C-reactive protein. J Med Virol 93(5): 3023-3032. 10.1002/jmv.26837
[334] Kolhe NV, Fluck RJ, Selby NM, Taal MW (2020) Acute kidney injury associated with COVID-19: A retrospective cohort study. PLoS Med 17(10): e1003406. 10.1371/journal.pmed.1003406
[335] Kolinski JM, Sundararajan SK, Swartz S, Naik KV, Gupta N (2020) COVID-19 Experience in a Wisconsin Academic Medical Center. Wmj 119(3): 198-201
[336] Kragholm K, Gerds T, Fosbøl E, et al. (2020) Association Between Ibuprofen Exposure and Severe COVID-19 Infection: A Nationwide Register-Based Study. ResearchSquare. 10.21203/rs.3.rs-26355/v1
[337] Kumar A, Kumar P, Dungdung A, Kumar Gupta A, Anurag A, Kumar A (2020) Pattern of liver function and clinical profile in COVID-19: A cross-sectional study of 91 patients. Diabetes Metab Syndr 14(6): 1951-1954. 10.1016/j.dsx.2020.10.001
[338] Laake JH, Buanes EA, Småstuen MC, et al. (2021) Characteristics, management and survival of ICU patients with coronavirus disease-19 in Norway, March-June 2020. A prospective observational study. Acta Anaesthesiol Scand 65(5): 618-628. 10.1111/aas.13785
[339] Laires PA, Dias S, Gama A, et al. (2021) The Association Between Chronic Disease and Serious COVID-19 Outcomes and Its Influence on Risk Perception: Survey Study and Database Analysis. JMIR Public Health Surveill 7(1): e22794. 10.2196/22794
[340] Lally MA, Tsoukas P, Halladay CW, O'Neill E, Gravenstein S, Rudolph JL (2020) Metformin is Associated with Decreased 30-Day Mortality Among Nursing Home Residents Infected with SARS-CoV2. J Am Med Dir Assoc. 10.1016/j.jamda.2020.10.031
[341] Lanini S, Montaldo C, Nicastri E, et al. (2020) COVID-19 disease-Temporal analyses of complete blood count parameters over course of illness, and relationship to patient demographics and management outcomes in survivors and non-survivors: A longitudinal descriptive cohort study. PLoS One 15(12): e0244129. 10.1371/journal.pone.0244129
[342] Lee SC, Son KJ, Han CH, Jung JY, Park SC (2020) Impact of comorbid asthma on severity of coronavirus disease (COVID-19). Scientific Reports 10(1). 10.1038/s41598-020-77791-8
[343] Lei G, dan j, xiaocheng c, et al. (2020) Prognostic value of CD4+T cell in patients with severe COVID-19. 10.22541/AU.158948998.84575439
[344] Leigh AE, McCall J, Burke RV, Rome R, Raines AM (2020) Predictors of functional dependence after COVID-19: A retrospective examination among veterans. Am J Phys Med Rehabil. 10.1097/phm.0000000000001614
[345] Lenin D-R, Francisco R-P, Francisca S-J, Gerardo S-L, Paulina C-H (2020) The role of metabolic comorbidity in COVID-19 mortality of middle-aged adults. The case of Mexico. medRxiv. 10.1101/2020.12.15.20244160
[346] Lenze EJ, Mattar C, Zorumski CF, et al. (2020) Fluvoxamine vs Placebo and Clinical Deterioration in Outpatients With Symptomatic COVID-19: A Randomized Clinical Trial. Jama 324(22): 2292-2300. 10.1001/jama.2020.22760
[347] Leon-Abarca JA, Memon RS, Rehan B, Iftikhar M, Chatterjee A (2020) The impact of COVID-19 in diabetic kidney disease and chronic kidney disease: A population-based study. Acta Biomed 91(4): e2020161. 10.23750/abm.v91i4.10380
[348] Leong A, Cole JB, Brenner LN, Meigs JB, Florez JC, Mercader JM (2021) Cardiometabolic risk factors for COVID-19 susceptibility and severity: A Mendelian randomization analysis. PLoS Med 18(3): e1003553. 10.1371/journal.pmed.1003553
[349] Leulseged T, Alemahu D, Hassen I, et al. (2020) Determinants of Developing Symptomatic Disease in Ethiopian COVID-19 Patients: A Case-Control Study. ResearchSquare. 10.21203/rs.3.rs-136541/v1
[350] Li G, Deng Q, Feng J, Li F, Xiong N, He Q (2020) Clinical Characteristics of Diabetic Patients with COVID-19. J Diabetes Res 2020: 1652403. 10.1155/2020/1652403

[351] Li H, Zhang J, Zhang J, et al. (2020) Clinical Characteristics and Prognosis of Patients with COVID-19 Combineded with or without Diabetes, Hypertension or Coronary. ResearchSquare. 10.21203/rs.3.rs-36840/v1
[352] Li HQ, Tian SH, Chen T, et al. (2020) Newly diagnosed diabetes is associated with a higher risk of mortality than known diabetes in hospitalized patients withCOVID-19. Diabetes Obesity & Metabolism 22(10): 1897-1906. 10.1111/dom.14099
[353] Li J, Zhang Y, Wang F, et al. (2020) Cardiac damage in patients with the severe type of coronavirus disease 2019 (COVID-19). BMC Cardiovasc Disord 20(1): 479. 10.1186/s12872-020-01758-w
[354] Palaiodimos L, Chamorro-Pareja N, Karamanis D, et al. (2020) Diabetes is associated with increased risk for in-hospital mortality in patients with COVID-19: a systematic review and meta-analysis comprising 18,506 patients. Hormones (Athens): 1-10. 10.1007/s42000-020-00246-2
[355] Li L, Sun W, Han M, Ying Y, Wang Q (2020) A Study on the Predictors of Disease Severity of COVID-19. Med Sci Monit 26: e927167. 10.12659/msm.927167
[356] Li W, Yu W, Liao J, et al. (2020) Analysis of Initial Clinical and CT Features of COVID-19 Pneumonia in Different Clinical Types. ResearchSquare. 10.21203/rs.3.rs-30395/v1
[357] Li X, Lei X, Wang J, Kou L, Yang Z (2021) Risk Factors for Severe Cases of Inpatients with COVID-19 in Henan Province, China: A Multicenter Retrospective Cohort Study. ResearchSquare. 10.21203/rs.3.rs-150954/v1
[358] Li Y, Regan J, Fajnzylber J, et al. (2020) Liver Fibrosis Index FIB-4 Is Associated With Mortality in COVID-19. Hepatol Commun 5(3): 434-445. 10.1002/hep4.1650
[359] Li Y, Wang Z, Hui Y, et al. (2020) Clinical Characteristics of 77 Novel Coronavirus 2019 Infected Patients with Respiratory Failure in the Terminal Stage in Wuhan. SSRN. 10.2139/ssrn.3551325
[360] Gao YD, Ding M, Dong X, et al. (2020) Risk factors for severe and critically ill COVID-19 patients: A review. Allergy. 10.1111/all.14657
[361] Lian JS, Jin X, Hao SR, et al. (2020) Analysis of Epidemiological and Clinical Features in Older Patients With Coronavirus Disease 2019 (COVID-19) Outside Wuhan. Clinical Infectious Diseases 71(15): 740-747. 10.1093/cid/ciaa242
[362] Liao Y, Feng Y, Wang B, et al. (2020) Clinical characteristics and prognostic factors of COVID-19 patients progression to severe: a retrospective, observational study. Aging (Albany NY) 12(19): 18853-18865. 10.18632/aging.103931
[363] Lin L, Zhong C, Rao S, Lin H, Huang R, Chen F (2021) Clinical characteristics of 78 cases of patients infected with coronavirus disease 2019 in Wuhan, China. Exp Ther Med 21(1): 7. 10.3892/etm.2020.9439
[364] Liu J, Zhang S, Wu ZX, et al. (2020) Clinical outcomes of COVID-19 in Wuhan, China: a large cohort study. Annals of Intensive Care 10(1). 10.1186/s13613-020-00706-3
[365] Wang D, Liu Y, Zeng F, et al. (2021) Evaluation of the role and usefulness of clinical pharmacists at the Fangcang Hospital during COVID-19 outbreak. Int J Clin Pract: e14271. 10.1111/ijcp.14271
[366] Liu L, Wei W, Yang K, et al. (2020) Glycemic control before admission is an important determinant of prognosis in patients with coronavirus disease 2019. J Diabetes Investig. 10.1111/jdi.13431
[367] Wu J, Li W, Shi X, et al. (2020) Early antiviral treatment contributes to alleviate the severity and improve the prognosis of patients with novel coronavirus disease (COVID-19). J intern med
[368] Liu S, Zhang B, You J, Chen L, Yuan H, Zhang S (2020) Elevated fasting blood glucose at admission is associated with poor outcomes in patients with COVID-19. Diabetes Metab. 10.1016/j.diabet.2020.08.004
[369] Liu SP, Zhang Q, Wang W, et al. (2020) Hyperglycemia is a strong predictor of poor prognosis in COVID-19. Diabetes Research and Clinical Practice 167. 10.1016/j.diabres.2020.108338
[370] Liu SQ, Luo HY, Wang YC, et al. (2020) Clinical characteristics and risk factors of patients with severe COVID-19 in Jiangsu province, China: a retrospective multicentre cohort study. Bmc Infectious Diseases 20(1). 10.1186/s12879-020-05314-x
[371] Liu XQ, Xue S, Xu JB, et al. (2021) Clinical characteristics and related risk factors of disease severity in 101 COVID-19 patients hospitalized in Wuhan, China. Acta Pharmacol Sin: 1-12. 10.1038/s41401-021-00627-2
[372] Liu Y, Lu R, Wang J, et al. (2020) Diabetes, even newly defined by HbA1c testing, is associated with an increased risk of in-hospital death in adults with COVID-19. ResearchSquare. 10.21203/rs.3.rs-93792/v1
[373] Liu ZM, Li JP, Huang JL, et al. (2020) Association Between Diabetes and COVID-19: A Retrospective Observational Study With a Large Sample of 1,880 Cases in Leishenshan Hospital, Wuhan. Frontiers in Endocrinology 11. 10.3389/fendo.2020.00478

[374] Lohia P, Kapur S, Benjaram S, Pandey A, Mir T, Seyoum B (2021) Metabolic syndrome and clinical outcomes in patients infected with COVID-19: Does age, sex, and race of the patient with metabolic syndrome matter? J Diabetes. 10.1111/1753-0407.13157
[375] Loice Achieng O, Nyamai M, Paul S, et al. (2020) EPIDEMIOLOGICAL AND CLINICAL CHARACTERISTICS OF COVID-19 PATIENTS IN KENYA. medRxiv. 10.1101/2020.11.09.20228106
[376] Long Q, Zhou C, Wang Y, et al. (2020) Admission biochemical test associated with the prognosis of COVID-19: a multi-centered retrospective cohort study. ResearchSquare. 10.21203/rs.3.rs-23644/v1
[377] Lowe R, Ferrari M, Nasim-Mohi M, et al. (2020) Clinical characteristics and outcome of critically ill COVID-19 patients with Acute Kidney Injury: A single centre cohort study. ResearchSquare. 10.21203/rs.3.rs-40934/v1
[378] Lu W, Fang J, Chen B, et al. (2020) Clinical Risk Factors for Long-term Hospital Stay in Common Patients with coronavirus disease 2019(COVID-19). ResearchSquare. 10.21203/rs.3.rs-34982/v1
[379] Lu Y, Sun K, Guo S, et al. (2020) Early Warning Indicators of Severe COVID-19: A Single-Center Study of Cases From Shanghai, China. Front Med (Lausanne) 7: 432-432
[380] Lucar J, Wingler MJB, Cretella DA, et al. (2021) Epidemiology, Clinical Features, and Outcomes of Hospitalized Adults with COVID-19: Early Experience from an Academic Medical Center in Mississippi. South Med J 114(3): 144-149. 10.14423/smj.0000000000001222
[381] Lui GC-Y, Terry Cheuk-Fung Y, Vincent Wai-Sun W, et al. (2020) Adverse Clinical Outcomes in COVID-19 Versus SARS: A Territory-Wide Cohort Study in Hong Kong. SSRN. 10.2139/ssrn.3572866
[382] Luo H, Liu S, Wang Y, et al. (2020) Age differences in clinical features and outcomes in patients with COVID-19, Jiangsu, China: a retrospective, multicentre cohort study. BMJ Open 10(10): e039887. 10.1136/bmjopen-2020-039887
[383] Luo LM, Fu ML, Li YY, et al. (2020) The potential association between common comorbidities and severity and mortality of coronavirus disease 2019: A pooled analysis. Clinical Cardiology 43(12): 1478-1493. 10.1002/clc.23465
[384] Luz Yupari I, Bardales Aguirre L, Rodriguez Azabache J, Barros Sevillano J, Rodríguez Díaz A (2021) Risk Factors For Mortality From COVID-19 In Hospitalized Patients: A Logistic Regression Model. Rev Fac Med Hum 21(1): 19-27
[385] Lv Z, Lv S (2021) Clinical characteristics and analysis of risk factors for disease progression of COVID-19: A retrospective Cohort Study. Int J Biol Sci 17(1): 1-7. 10.7150/ijbs.50654
[386] Ma Y, Zhu DS, Chen RB, et al. (2020) Association of Overlapped and Un-overlapped Comorbidities with COVID-19 Severity and Treatment Outcomes: A Retrospective Cohort Study from Nine Provinces in China. Biomed Environ Sci 33(12): 893-905. 10.3967/bes2020.123
[387] Maddaloni E, D'Onofrio L, Alessandri F, et al. (2020) Clinical features of patients with type 2 diabetes with and without Covid-19: A case control study (CoViDiab I). Diabetes Res Clin Pract 169: 108454. 10.1016/j.diabres.2020.108454
[388] Maghbooli Z, Sahraian MA, Ebrahimi M, et al. (2020) Vitamin D sufficiency, a serum 25-hydroxyvitamin D at least 30 ng/mL reduced risk for adverse clinical outcomes in patients with COVID-19 infection. PLoS One 15(9): e0239799. 10.1371/journal.pone.0239799
[389] Magro B, Zuccaro V, Novelli L, et al. (2021) Predicting in-hospital mortality from Coronavirus Disease 2019: A simple validated app for clinical use. PLoS One 16(1): e0245281. 10.1371/journal.pone.0245281
[390] Mahmood M, Khurshid S, Khan M, et al. (2021) Risk factors associated with mortality in COVID-19 patients: a retrospective case control study. ResearchSquare. 10.21203/rs.3.rs-123226/v1
[391] Malavazos AE, Secchi F, Basilico S, et al. (2021) Abdominal obesity phenotype is associated with COVID-19 chest X-ray severity score better than BMI-based obesity. Eat Weight Disord: 1-15. 10.1007/s40519-021-01173-w
[392] Malin H, Joshua R, Cara AR, et al. (2020) COVID-19 Infections and Clinical Outcomes in Patients with Multiple Myeloma in New York City: A Cohort Study from Five Academic Centers. 1(3). 10.1158/2643-3230.BCD-20-0102
[393] Mari T, Hiroshi O, Sho S, et al. (2021) Risk factors for severity on admission and the disease progression during hospitalization in a large cohort of COVID-19 patients in Japan. medRxiv. 10.1101/2021.04.02.21254809
[394] Maripuu M, Bendix M, Öhlund L, Widerström M, Werneke U (2020) Death Associated With Coronavirus (COVID-19) Infection in Individuals With Severe Mental Disorders in Sweden During the Early Months of the Outbreak-An Exploratory Cross-Sectional Analysis of a Population-Based Register Study. Front Psychiatry 11: 609579. 10.3389/fpsyt.2020.609579
[395] Marmarchi F, Liu M, Rangaraju S, et al. (2021) Clinical Outcomes of Critically Ill Patients with COVID-19 by Race. J Racial Ethn Health Disparities: 1-5. 10.1007/s40615-021-00966-0

[396] Martensson J, Engerstrom L, Walther S, Grip J, Berggren RK, Larsson E (2020) COVID-19 critical illness in Sweden: characteristics and outcomes at a national population level. Critical Care and Resuscitation 22(4): 312-320
[397] Martin D, David H, Said L, et al. (2020) Characteristics, Management and Prognosis of Elderly Patients with COVID-19 Admitted in the ICU During the First Wave: Insights from the COVID-ICU Study. SSRN. 10.2139/ssrn.3758021
[398] Martina B, Federico M, Edlira S, et al. (2020) Predictors of Worse Prognosis in Young Adults Hospitalized with COVID-19 Pneumonia: A Multi-Center Italian Study (COVID-UNDER50). SSRN. 10.2139/ssrn.3727959
[399] Martínez-Botía P, Bernardo Á, Acebes-Huerta A, et al. (2021) Clinical Management of Hypertension, Inflammation and Thrombosis in Hospitalized COVID-19 Patients: Impact on Survival and Concerns. J Clin Med 10(5). 10.3390/jcm10051073
[400] Martins PR, Araujo AAD, Pereira LX, et al. (2021) Factors Associated with Mortality among Hospitalized Patients with COVID-19: A Retrospective Cohort Study. American Journal of Tropical Medicine and Hygiene 104(1): 103-105. 10.4269/ajtmh.20-1170
[401] Martos-Benítez FD, Soler-Morejón CD, García-Del Barco D (2021) Chronic comorbidities and clinical outcomes in patients with and without COVID-19: a large population-based study using national administrative healthcare open data of Mexico. Intern Emerg Med: 1-11. 10.1007/s11739-020-02597-5
[402] Marwah M, Marwah S, Blann A, Morrissey H, Ball P, Wandroo FA (2021) Analysis of laboratory blood parameter results for patients diagnosed with COVID-19, from all ethnic group populations: A single centre study. Int J Lab Hematol. 10.1111/ijlh.13538
[403] Mash RJ, Presence-Vollenhoven M, Adeniji A, et al. (2021) Evaluation of patient characteristics, management and outcomes for COVID-19 at district hospitals in the Western Cape, South Africa: descriptive observational study. BMJ Open 11(1): e047016. 10.1136/bmjopen-2020-047016
[404] Massimo C, Haseeb M, Matt JK, Noel DM (2020) Contrasting factors associated with COVID-19-related ICU and death outcomes: interpretable multivariable analyses of the UK CHESS dataset. In:
[405] Matangila JR, Nyembu RK, Telo GM, et al. (2020) Clinical characteristics of COVID-19 patients hospitalized at Clinique Ngaliema, a public hospital in Kinshasa, in the Democratic Republic of Congo: A retrospective cohort study. PLoS One 15(12): e0244272. 10.1371/journal.pone.0244272
[406] Matsuzawa Y, Ogawa H, Kimura K, et al. (2020) Renin-angiotensin system inhibitors and the severity of coronavirus disease 2019 in Kanagawa, Japan: a retrospective cohort study. Hypertens Res 43(11): 1257-1266. 10.1038/s41440-020-00535-8
[407] Matteo R, Paolo B, Angela B, et al. (2020) Obesity Is One of the Strongest Risk Factor for Respiratory Failure and Death in COVID-19 Patients: A Retrospective Multicentric Cohort Study. SSRN. 10.2139/ssrn.3578779
[408] Mazori AY, Bass IR, Chan L, et al. (2021) Hyperglycemia is Associated With Increased Mortality in Critically Ill Patients With COVID-19. Endocr Pract 27(2): 95-100. 10.1016/j.eprac.2020.12.015
[409] McCarty TR, Hathorn KE, Redd WD, et al. (2020) How Do Presenting Symptoms and Outcomes Differ by Race/Ethnicity Among Hospitalized Patients with COVID-19 Infection? Experience in Massachusetts. Clin Infect Dis. 10.1093/cid/ciaa1245
[410] McCracken JA, Nakeshbandi M, Arace J, Riley WJ, Sharma R (2020) COVID-19 related deaths in an urban academic medical center in Brooklyn - a descriptive case series. Transl Med Commun 5(1): 12. 10.1186/s41231-020-00065-y
[411] McGurnaghan SJ, Weir A, Bishop J, et al. (2021) Risks of and risk factors for COVID-19 disease in people with diabetes: a cohort study of the total population of Scotland. Lancet Diabetes Endocrinol 9(2): 82-93. 10.1016/S2213-8587(20)30405-8
[412] McKeigue PM, Weir A, Bishop J, et al. (2020) Rapid Epidemiological Analysis of Comorbidities and Treatments as risk factors for COVID-19 in Scotland (REACT-SCOT): A population-based case-control study. PLoS Med 17(10): e1003374. 10.1371/journal.pmed.1003374
[413] Md. Shahed M, Abdullah Al M, Prodipta C, Sheikh Mohammad A, Mohammad Sorowar H (2020) Clinical manifestations of patients with Coronavirus Disease 2019 (COVID- 19) attending at hospitals in Bangladesh. medRxiv. 10.1101/2020.07.30.20165100
[414] Md. Ziaul I, Baizid Khoorshid R, Islam ANMS, et al. (2020) Risk factors associated with morbidity and mortality outcomes of COVID-19 patients on the 14th and 28th day of the disease course: a retrospective cohort study in Bangladesh. medRxiv. 10.1101/2020.08.17.20176586
[415] Mehanna O, El Askary A, Ali E, El Esawy B, FathAlla T, Gharib AF (2021) Impact of Obesity and Its Associated Comorbid Conditions on COVID-19 Presentation. Diabetes Metab Syndr Obes 14: 409-415. 10.2147/dmso.S287779

[416] Mehar Muhammad I, Umair A, Umer U, Majid A, Aamir S, Noor G (2020) Neutrophil/Lymphocyte Ratio – A Marker of COVID-19 Pneumonia Severity. 10.22541/AU.159969693.39051590
[417] Mehta A, Vasudevan S, Parkash A, Sharma A, Vashist T, Krishna V (2021) COVID-19 mortality in cancer patients: a report from a tertiary cancer centre in India. PeerJ 9: e10599. 10.7717/peerj.10599
[418] Mehta HB, Li S, Goodwin JS (2021) Risk Factors Associated With SARS-CoV-2 Infections, Hospitalization, and Mortality Among US Nursing Home Residents. JAMA Netw Open 4(3): e216315. 10.1001/jamanetworkopen.2021.6315
[419] Mejía F, Medina C, Cornejo E, et al. (2020) Oxygen saturation as a predictor of mortality in hospitalized adult patients with COVID-19 in a public hospital in Lima, Peru. PLoS One 15(12): e0244171. 10.1371/journal.pone.0244171
[420] Mesipogu R, Adeula V, Bingi T, et al. (2020) Assessment of mortality in correlation with comorbidities in COVID-19 patients of Telangana state. ResearchSquare. 10.21203/rs.3.rs-95462/v1
[421] Micallef S, Piscopo TV, Casha R, et al. (2020) The first wave of COVID-19 in Malta; a national cross-sectional study. PLoS One 15(10): e0239389. 10.1371/journal.pone.0239389
[422] Miike S, Sakamoto N, Washino T, et al. (2020) Critically ill patients with COVID-19 in Tokyo, Japan: A single-center case series. J Infect Chemother. 10.1016/j.jiac.2020.10.019
[423] Ming L, Colette JS, Sam D, et al. (2021) Clinical Outcomes of Patients With and Without HIV Hospitalised with COVID-19 in England During the Early Stages of the Pandemic: A Matched Retrospective Multicentre Analysis (RECEDE-C19 Study). SSRN. 10.2139/ssrn.3771328
[424] Mingyu L, Shuolong H, Qiaohong L, et al. (2020) Outcomes and Prognostic Factors in 70 Non-Survivors and 595 Survivors with COVID-19 in Wuhan, China. SSRN. 10.2139/ssrn.3572885
[425] Miriam N, Yury G, Ganesh R, Diego P, Alec JS (2021) COVID-19 Hospitalizations in Five California Hospitals. medRxiv. 10.1101/2021.01.29.21250788
[426] Moghaddam Tabrizi F, Rasmi Y, Hosseinzadeh E, et al. (2021) Diabetes is associated with higher mortality and severity in hospitalized patients with COVID-19. Excli j 20: 444-453. 10.17179/excli2021-3403
[427] Mohamed MO, Gale CP, Kontopantelis E, et al. (2020) Sex Differences in Mortality Rates and Underlying Conditions for COVID-19 Deaths in England and Wales. Mayo Clin Proc 95(10): 2110-2124. 10.1016/j.mayocp.2020.07.009
[428] Mohamed NE, Benn EKT, Astha V, et al. (2021) Association between chronic kidney disease and COVID-19-related mortality in New York. World J Urol: 1-7. 10.1007/s00345-020-03567-4
[429] Mohammadi M, Varpaei H, Amini M (2020) Intubation prognosis in COVID-19 patients and associated factors: a cross-sectional study. ResearchSquare. 10.21203/rs.3.rs-115894/v1
[430] Mohamud M, Hashi A, Mohamed A, Yusuf A, Ali I, Ahmed M (2020) Clinical Characteristics, Comorbidities, Initial Management and Outcome of COVID-19 Infected Patients Admitted to Intensive Care Unit in Somalia: A National Retrospective Study. ResearchSquare. 10.21203/rs.3.rs-66767/v1
[431] Mohsen K, Ahmad T, Sareh Z, et al. (2020) Clinical, Laboratory, and Imaging Features of 148 Patients with COVID-19 in Bushehr: A Report from the South of Iran. medRxiv. 10.1101/2020.08.11.20172692
[432] Monterrubio-Flores E, Ramírez-Villalobos MD, Espinosa-Montero J, et al. (2021) Characterizing a two-pronged epidemic in Mexico of non-communicable diseases and SARS-Cov-2: factors associated with increased case-fatality rates. Int J Epidemiol. 10.1093/ije/dyab008
[433] Montrucchio G, Sales G, Rumbolo F, et al. (2020) Effectiveness of Mid-Regional Pro-Adrenomedullin (MR-proADM) as Prognostic Marker in COVID-19 Critically Ill Patients: an Observational Prospective Study. ResearchSquare. 10.21203/rs.3.rs-56715/v1
[434] Moon SJ, Rhee EJ, Jung JH, et al. (2020) Independent Impact of Diabetes on the Severity of Coronavirus Disease 2019 in 5,307 Patients in South Korea: A Nationwide Cohort Study. Diabetes Metab J 44(5): 737-746. 10.4093/dmj.2020.0141
[435] Moon SS, Lee K, Park J, Yun S, Lee YS, Lee DS (2020) Clinical Characteristics and Mortality Predictors of COVID-19 Patients Hospitalized at Nationally-Designated Treatment Hospitals. J Korean Med Sci 35(36): e328. 10.3346/jkms.2020.35.e328
[436] Moreno-Perez O, Andres M, Leon-Ramirez JM, et al. (2020) Experience with tocilizumab in severe COVID-19 pneumonia after 80 days of follow-up: A retrospective cohort study. Journal of Autoimmunity 114. 10.1016/j.jaut.2020.102523
[437] Morys F, Dagher A (2021) Poor Metabolic Health Increases COVID-19-Related Mortality in the UK Biobank Sample. Front Endocrinol (Lausanne) 12: 652765. 10.3389/fendo.2021.652765
[438] Mostaghim A, Sinha P, Bielick C, et al. (2020) Clinical outcomes and inflammatory marker levels in patients with Covid-19 and obesity at an inner-city safety net hospital. PLoS One 15(12): e0243888. 10.1371/journal.pone.0243888
[439] Motaib I, Zbiri S, Elamari S, Dini N, Chadli A, El Kettani C (2021) Obesity and Disease Severity Among Patients With COVID-19. Cureus 13(2): e13165. 10.7759/cureus.13165

[440] Mughal MS, Kaur IP, Jaffery AR, et al. (2020) COVID-19 patients in a tertiary US hospital: Assessment of clinical course and predictors of the disease severity. Respir Med 172: 106130. 10.1016/j.rmed.2020.106130
[441] Muhammad R, Ogunti R, Ahmed B, et al. (2021) Clinical Characteristics and Predictors of Mortality in Minority Patients Hospitalized with COVID-19 Infection. J Racial Ethn Health Disparities: 1-11. 10.1007/s40615-020-00961-x
[442] Murillo-Zamora E, Mendoza-Cano O, Delgado-Enciso I, Hernandez-Suarez CM (2021) Predictors of severe symptomatic laboratory-confirmed SARS-CoV-2 reinfection. Public Health 193: 113-115. 10.1016/j.puhe.2021.01.021
[443] Murthy S, Archambault PM, Atique A, et al. (2021) Characteristics and outcomes of patients with COVID-19 admitted to hospital and intensive care in the first phase of the pandemic in Canada: a national cohort study. CMAJ Open 9(1): E181-e188. 10.9778/cmajo.20200250
[444] Mutair A, Mutairi A, Alhumaid S, Abdullah S, Zaidi A, Al-Omari A (2021) Examining and Investigating the Impact of Demographic Characteristics and Chronic Disease with Mortality of COVID-19: Retrospective Study. ResearchSquare. 10.21203/rs.3.rs-274197/v1
[445] Naeem KB, Hachim MY, Hachim IY, et al. (2020) Acute cardiac injury is associated with adverse outcomes, including mortality in COVID-19 patients. A single-center experience. Saudi Med J 41(11): 1204-1210. 10.15537/smj.2020.11.25466
[446] Nair S, Gasmelseed H, Khan A, et al. (2021) Higher Mortality from Covid19 in Patients with Renal Dysfunction in A Multicultural Multi-Ethnic Population. ResearchSquare. 10.21203/rs.3.rs-243317/v1
[447] Najera H, Ortega-Avila AG (2021) Health and Institutional Risk Factors of COVID-19 Mortality in Mexico, 2020. Am J Prev Med 60(4): 471-477. 10.1016/j.amepre.2020.10.015
[448] Ñamendys-Silva SA, Alvarado-Ávila PE, Domínguez-Cherit G, et al. (2021) Outcomes of patients with COVID-19 in the intensive care unit in Mexico: A multicenter observational study. Heart Lung 50(1): 28-32. 10.1016/j.hrtlng.2020.10.013
[449] Naomi H, Peter K, Partha K, et al. (2020) Type 1 and Type 2 Diabetes and COVID-19 Related Mortality in England: A Cohort Study in People with Diabetes. SSRN. 10.2139/ssrn.3605226
[450] Nasir N, Habib K, Iffat K, Khan N, Muhammad ZA, Mahmood SF (2021) Clinical characteristics and outcomes of COVID-19: Experience at a major tertiary care center in Pakistan. J Infect Dev Ctries 15(4): 480-489. 10.3855/jidc.14345
[451] Neves PD, Sato V, Mohrbacher S, et al. (2020) AKI due to COVID-19 in the intensive care unit: Analysis of a Brazilian Center. Journal of The American Society of Nephrology 31
[452] Newman CB (2020) Mortality in COVID-19: Further Evidence for a Sex-Based Difference in the OpenSAFELY Study. J Womens Health (Larchmt). 10.1089/jwh.2020.8777
[453] Newton S, Zollinger B, Freeman J, et al. (2020) Factors associated with clinical severity in Emergency Department patients presenting with symptomatic SARS-CoV-2 infection. medRxiv. 10.1101/2020.12.08.20246017
[454] Nicholson CJ, Wooster L, Sigurslid HH, et al. (2020) Estimating Risk of Mechanical Ventilation and Mortality Among Adult COVID-19 patients Admitted to Mass General Brigham: The VICE and DICE Scores. medRxiv. 10.1101/2020.09.14.20194670
[455] Nicolás P-R, Efraín N-O, María de Jesús G-L, et al. (2020) Clinical Data, Comorbidities, and Mortality of COVID-19 in the State of Guanajuato, Mexico until May 20, 2020. Central Asian Journal of Global Health 9(1). 10.5195/CAJGH.2020.527
[456] Nicole Fouda M, Emilienne E, Marcel M, et al. (2021) Clinical profile and factors associated with COVID-19 in Cameroon: a prospective cohort study. medRxiv. 10.1101/2021.02.19.21252071
[457] Nijman G, Wientjes M, Ramjith J, et al. (2021) Risk factors for in-hospital mortality in laboratory-confirmed COVID-19 patients in the Netherlands: A competing risk survival analysis. PLoS One 16(3): e0249231. 10.1371/journal.pone.0249231
[458] Niu Y, Zhan Z, Li J, et al. (2021) Development of a Predictive Model for Mortality in Hospitalized Patients With COVID-19. Disaster Med Public Health Prep: 1-9. 10.1017/dmp.2021.8
[459] Nnamendys-Silva SA, Alvarado-Avila PE, Dominguez-Cherit G, et al. (2021) Outcomes of patients with COVID-19 in the intensive care unit in Mexico: A multicenter observational study. Heart & Lung 50(1): 28-32. 10.1016/j.hrtlng.2020.10.013
[460] Nogueira PJ, Nobre MD, Costa A, et al. (2020) The Role of Health Preconditions on COVID-19 Deaths in Portugal: Evidence from Surveillance Data of the First 20293 Infection Cases. Journal of Clinical Medicine 9(8). 10.3390/jcm9082368
[461] O'Hearn M, Liu J, Cudhea F, Micha R, Mozaffarian D (2021) Coronavirus Disease 2019 Hospitalizations Attributable to Cardiometabolic Conditions in the United States: A Comparative Risk Assessment Analysis. J Am Heart Assoc 10(5): e019259. 10.1161/jaha.120.019259
[462] Olivas-Martínez A, Cárdenas-Fragoso JL, Jiménez JV, et al. (2021) In-hospital mortality from severe COVID-19 in a tertiary care center in Mexico City; causes of death, risk factors and the impact of hospital saturation. PLoS One 16(2): e0245772. 10.1371/journal.pone.0245772

[463] Omar Yaxmehen B-C, Armando G-D, Neftali EA-V, et al. (2020) Unequal impact of structural health determinants and comorbidity on COVID-19 severity and lethality in older Mexican adults: Looking beyond chronological aging. medRxiv. 10.1101/2020.05.12.20098699
[464] Omer A, Bengu S (2020) Retrospective analysis of severe COVID-19 pneumonia patients treated with lopinavir/ritonavir: A comparison with survivor and non-survivor patients. Southern African Journal of Infectious Diseases 35(1). 10.4102/SAJID.V35I1.233
[465] Omrani AS, Almaslamani MA, Daghfal J, et al. (2020) The first consecutive 5000 patients with Coronavirus Disease 2019 from Qatar; a nation-wide cohort study. Bmc Infectious Diseases 20(1). 10.1186/s12879-020-05511-8
[466] Oozdemir N, Dizdar O, Yazici O, et al. (2021) Clinical features and outcomes of COVID-19 in patients with solid tumors: Turkish National Registry Data. International Journal of Cancer 148(10): 2407-2415. 10.1002/ijc.33426
[467] Ordoñez E, Montalvo E, Paez M, et al. (2021) Mortality and Risk Factors in Patients With COVID-19 in Ecuador: A Cross-Sectional Analytic Study. ResearchSquare. 10.21203/rs.3.rs-261732/v1
[468] Orison OW, Juan PC-B (2020) Diabetes and Mortality Among 1.6 Million Adult Patients Screened for SARS-CoV-2 in Mexico. medRxiv. 10.1101/2020.11.25.20238345
[469] Òscar M, Pere L, Sònia J, et al. (2020) Frequency, Risk Factors, Clinical Characteristics and Outcomes of Spontaneous Pneumothorax in Patients with COVID-19 Results of the UMC-19-S7. SSRN. 10.2139/ssrn.3619814
[470] Osibogun A, Balogun M, Abayomi A, et al. (2021) Outcomes of COVID-19 patients with comorbidities in southwest Nigeria. PLoS One 16(3): e0248281. 10.1371/journal.pone.0248281
[471] Pablos JL, Galindo M, Carmona L, et al. (2020) Clinical outcomes of hospitalised patients with COVID-19 and chronic inflammatory and autoimmune rheumatic diseases: a multicentric matched cohort study. Annals of the Rheumatic Diseases 79(12): 1544-1549. 10.1136/annrheumdis-2020-218296
[472] Pacheco-Pantoja EL, Ferreyro-Bravo FA, Ceballos-Cruz ÁE (2020) COVID-19, diabetes, obesidad e hipertensión arterial: 60 días de pandemia en México. Revista Mexicana de Endocrinología, Metabolismo y Nutrición 7(2): 68-79. 10.24875/RME.20000042
[473] Padilla-Raygoza N, Flores-Vargas G, Navarro-Olivos E, et al. (2020) Analysis of fatality of cases by the new coronavirus, in the Mexican state of Guanajuato: A cross-sectional study. ResearchSquare. 10.21203/rs.3.rs-112119/v1
[474] Panagiotou OA, Kosar CM, White EM, et al. (2021) Risk Factors Associated With All-Cause 30-Day Mortality in Nursing Home Residents With COVID-19. JAMA Intern Med 181(4): 439-448. 10.1001/jamainternmed.2020.7968
[475] Pantea Stoian A, Pricop-Jeckstadt M, Pana A, et al. (2020) Death by SARS-CoV 2: a Romanian COVID-19 multi-centre comorbidity study. Sci Rep 10(1): 21613. 10.1038/s41598-020-78575-w
[476] Papageorgiou N, Providencia R, Saberwal B, et al. (2020) Ethnicity and COVID-19 cardiovascular complications: a multi-center UK cohort. Am J Cardiovasc Dis 10(4): 455-462
[477] Park BE, Lee JH, Park HK, et al. (2021) Impact of Cardiovascular Risk Factors and Cardiovascular Diseases on Outcomes in Patients Hospitalized with COVID-19 in Daegu Metropolitan City. J Korean Med Sci 36(2): e15. 10.3346/jkms.2021.36.e15
[478] Park JG, Kang MK, Lee YR, et al. (2020) Fibrosis-4 index as a predictor for mortality in hospitalised patients with COVID-19: a retrospective multicentre cohort study. BMJ Open 10(11): e041989. 10.1136/bmjopen-2020-041989
[479] Park S, Lee H, Youk T, Jeon H (2021) Risk Factors for Mortality of Patients with COVID-19 in Korea: A Nationwide Population-based Study. ResearchSquare. 10.21203/rs.3.rs-143694/v1
[480] Park SC, Won SY, Kim NH, et al. (2021) Risk factors for severe acute respiratory syndrome coronavirus 2 (SARS-CoV-2) infections: a nationwide population-based study. Ann Transl Med 9(3): 211. 10.21037/atm-20-5958
[481] Park SD, Kim SW, Moon JS, et al. (2020) Impact of Social Distancing Due to Coronavirus Disease 2019 on the Changes in Glycosylated Hemoglobin Level in People with Type 2 Diabetes Mellitus. Diabetes Metab J. 10.4093/dmj.2020.0226
[482] Parra-Bracamonte GM, Lopez-Villalobos N, Parra-Bracamonte FE (2020) Clinical characteristics and risk factors for mortality of patients with COVID-19 in a large data set from Mexico. Ann Epidemiol 52: 93-98.e92. 10.1016/j.annepidem.2020.08.005
[483] Parra-Bracamonte GM, Parra-Bracamonte FE, Lopez-Villalobos N, Lara-Rivera AL (2020) Chronic kidney disease is a very significant comorbidity for high risk of death in patients with COVID-19 in Mexico. Nephrology (Carlton). 10.1111/nep.13827
[484] Pasquel FJ, Messler J, Booth R, et al. (2021) Characteristics of and Mortality Associated With Diabetic Ketoacidosis Among US Patients Hospitalized With or Without COVID-19. JAMA Netw Open 4(3): e211091. 10.1001/jamanetworkopen.2021.1091

[485] Pedro Emanuel F, Maria Cristina A, Sabrina Daniela C, Jorge Augusto P, Ruben Oscar C, Alejandro Javier K (2021) Association between comorbidities and death from COVID-19 in different age groups. medRxiv. 10.1101/2021.04.12.21255365
[486] Peña JE, Rascón-Pacheco RA, Ascencio-Montiel IJ, et al. (2020) Hypertension, Diabetes and Obesity, Major Risk Factors for Death in Patients With COVID-19 in Mexico. Arch Med Res. 10.1016/j.arcmed.2020.12.002
[487] Peng XJ, Chen YF, Deng LF, et al. (2020) Clinical features of critically ill patients infected with SARS-CoV-2 outside Wuhan with and without diabetes. International Journal of Diabetes in Developing Countries 40(4): 482-490. 10.1007/s13410-020-00888-3
[488] Peters SAE, MacMahon S, Woodward M (2021) Obesity as a risk factor for COVID-19 mortality in women and men in the UK biobank: Comparisons with influenza/pneumonia and coronary heart disease. Diabetes Obes Metab 23(1): 258-262. 10.1111/dom.14199
[489] Phelps M, Christensen DM, Gerds T, et al. (2020) Cardiovascular comorbidities as predictors for severe COVID-19 infection or death. Eur Heart J Qual Care Clin Outcomes. 10.1093/ehjqcco/qcaa081
[490] Phyo Kyaw M, Sanda S, Aung Aung L, et al. (2020) Rates and Risk Factors Associated with COVID-19 Infection Among Myanmar Ethnic Minority Doctors in UK NHS. SSRN. 10.2139/ssrn.3733606
[491] Pimentel S, Nascimento B, Franco J, et al. (2021) Echocardiography at Bedside For Predicting Mortality of COVID-19 Patients Beyond Clinical Data – Data From the PROVAR-COVID Study. ResearchSquare. 10.21203/rs.3.rs-435447/v1
[492] Poblador-Plou B, Carmona-Pirez J, Ioakeim-Skoufa I, et al. (2020) Baseline Chronic Comorbidity and Mortality in Laboratory-Confirmed COVID-19 Cases: Results from the PRECOVID Study in Spain. International Journal of Environmental Research and Public Health 17(14). 10.3390/ijerph17145171
[493] Poon J, Ho KS, Herrera Y, Jean R (2020) CLINICAL OUTCOMES IN DIABETIC VS NON-DIABETIC PATIENTS WITH SEVERE COVID-19. Chest 158(4): 688A-688A. 10.1016/j.chest.2020.08.647
[494] Portoles J, Marques M, Lopez-Sanchez P, et al. (2020) Chronic kidney disease and acute kidney injury in the COVID-19 Spanish outbreak. Nephrology Dialysis Transplantation 35(8): 1353-1361. 10.1093/ndt/gfaa189
[495] Pourhoseingholi M, Yousefi H, Manesh H, et al. (2021) Case characteristics, clinical data, and outcomes of hospitalized COVID-19 patients in Qom province, Iran: a prospective cohort study. ResearchSquare. 10.21203/rs.3.rs-365321/v1
[496] Prado-Galbarro FJ, Sanchez-Piedra C, Gamiño-Arroyo AE, Cruz-Cruz C (2020) Determinants of survival after severe acute respiratory syndrome coronavirus 2 infection in Mexican outpatients and hospitalised patients. Public Health 189: 66-72. 10.1016/j.puhe.2020.09.014
[497] Preethi R, Mahesh G, Abhilash P, et al. (2020) Red blood cell distribution width (RDW) in Hospitalized COVID-19 Patients. medRxiv. 10.1101/2020.06.29.20143081
[498] Priya DGO, S. , Meena MS, Priyadharshini CB, Vijay Anand V (2021) Factors influencing the outcome of COVID-19 patients admitted in a tertiary care hospital, Madurai.- a cross-sectional study. Clin Epidemiol Glob Health: 100705-100705
[499] Qijun G, Yingfu H, Zhiguo D, Feng X, Jing W, Jing W (2020) The Epidemiological Characteristics of 2019 Novel Coronavirus Diseases (COVID-19) in Jingmen, China. SSRN. 10.2139/ssrn.3548755
[500] Qing Qing W, Qi Y, Tongtong H, et al. (2020) Clinical Characteristics and Outcome in Coronavirus Disease 2019 (COVID-19) Patient with and Without Hypertension: Retrospective Study. SSRN. 10.2139/ssrn.3576770
[501] Qureshi AI, Baskett WI, Huang W, et al. (2021) Acute Ischemic Stroke and COVID-19 An Analysis of 27 676 Patients. Stroke 52(3): 905-912. 10.1161/strokeaha.120.031786
[502] Rahim F, Amin S, Noor M, et al. (2020) Mortality of Patients With Severe COVID-19 in the Intensive Care Unit: An Observational Study From a Major COVID-19 Receiving Hospital. Cureus 12(10): e10906. 10.7759/cureus.10906
[503] Rahul S, Shubhra U, Abubaker S, Jeanette A, Devika K (2020) Early experience with COVD-19 patients at tertiary care teaching hospital in southwestern United states. medRxiv. 10.1101/2020.05.15.20094284
[504] Raimondi F, Novelli L, Ghirardi A, et al. (2021) Covid-19 and gender: lower rate but same mortality of severe disease in women-an observational study. BMC Pulm Med 21(1): 96. 10.1186/s12890-021-01455-0
[505] Ramadan HK, Mahmoud MA, Aburahma MZ, et al. (2020) Predictors of Severity and Co-Infection Resistance Profile in COVID-19 Patients: First Report from Upper Egypt. Infect Drug Resist 13: 3409-3422. 10.2147/idr.S272605

[506] Ramon Perez T, Felipe Perez G, Juan R, et al. (2020) Prevalence and risk factors for mortality related to COVID-19 in a severely affected area of Madrid, Spain. medRxiv. 10.1101/2020.05.25.20112912
[507] Ramos-Rincon JM, Buonaiuto V, Ricci M, et al. (2020) Clinical Characteristics and Risk Factors for Mortality in Very Old Patients Hospitalized with COVID-19 in Spain. J Gerontol A Biol Sci Med Sci. 10.1093/gerona/glaa243
[508] Rao S, Ali K, Dennis J, Berdine G, Test V, Nugent K (2020) Analysis of Glucose Levels in Patients Hospitalized With COVID-19 During the First Phase of This Pandemic in West Texas. J Prim Care Community Health 11: 2150132720958533. 10.1177/2150132720958533
[509] Richard KS, Tavis O, Tamas SG, Michael PS, Marjolein d, Arun JS (2020) The FIB-4 Index Is Associated with Need for Mechanical Ventilation and 30-day Mortality in Patients Admitted with COVID-19. The Journal of Infectious Diseases
[510] Rivera-Izquierdo M, Valero-Ubierna MD, R-delAmo JL, et al. (2020) Sociodemographic, clinical and laboratory factors on admission associated with COVID-19 mortality in hospitalized patients: A retrospective observational study. Plos One 15(6). 10.1371/journal.pone.0235107
[511] Rizo-Téllez SA, Méndez-García LA, Flores-Rebollo C, et al. (2020) The Neutrophil-to-Monocyte Ratio and Lymphocyte-to-Neutrophil Ratio at Admission Predict In-Hospital Mortality in Mexican Patients with Severe SARS-CoV-2 Infection (Covid-19). Microorganisms 8(10). 10.3390/microorganisms8101560
[512] Rodilla E, Saura A, Jiménez I, et al. (2020) Association of Hypertension with All-Cause Mortality among Hospitalized Patients with COVID-19. J Clin Med 9(10). 10.3390/jcm9103136
[513] Rodriguez F, Solomon N, de Lemos JA, et al. (2020) Racial and Ethnic Differences in Presentation and Outcomes for Patients Hospitalized with COVID-19: Findings from the American Heart Association's COVID-19 Cardiovascular Disease Registry. Circulation. 10.1161/circulationaha.120.052278
[514] Rodriguez-Gonzalez CG, Chamorro-de-Vega E, Valerio M, et al. (2021) COVID-19 in hospitalised patients in Spain: a cohort study in Madrid. International Journal of Antimicrobial Agents 57(2). 10.1016/j.ijantimicag.2020.106249
[515] Rosita B, Rakesh B, Janeri F, et al. (2021) Mortality reduction in ICU-admitted COVID-19 patients in Suriname after treatment with convalescent plasma acquired via gravity filtration. medRxiv. 10.1101/2021.04.14.21255104
[516] Rossi PG, Marino M, Formisano D, et al. (2020) Characteristics and outcomes of a cohort of COVID-19 patients in the Province of Reggio Emilia, Italy. Plos One 15(8). 10.1371/journal.pone.0238281
[517] Rubio-Rivas M, Corbella X, Mora-Luján JM, et al. (2020) Predicting Clinical Outcome with Phenotypic Clusters in COVID-19 Pneumonia: An Analysis of 12,066 Hospitalized Patients from the Spanish Registry SEMI-COVID-19. J Clin Med 9(11). 10.3390/jcm9113488
[518] Ruocco G, McCullough PA, Tecson KM, et al. (2020) Mortality Risk Assessment Using CHA(2)DS(2)-VASc Scores in Patients Hospitalized With Coronavirus Disease 2019 Infection. Am J Cardiol 137: 111-117. 10.1016/j.amjcard.2020.09.029
[519] Ruth S, Fleur C, Andrew B, et al. (2020) Early Epidemiological and Clinical Analysis of the First 200 Patients with COVID-19 Admitted. SSRN. 10.2139/ssrn.3576791
[520] Saand AR, Flores M, Kewan T, et al. (2021) Does inpatient hyperglycemia predict a worse outcome in COVID-19 intensive care unit patients? Journal of Diabetes 13(3): 253-260. 10.1111/1753-0407.13137
[521] Sainsbury C, Wang JY, Gokhale K, et al. (2021) Sodium-glucose co-transporter-2 inhibitors and susceptibility toCOVID-19: A population-based retrospective cohort study. Diabetes Obesity & Metabolism 23(1): 263-269. 10.1111/dom.14203
[522] Salerno S, Zhao Z, Sankar SP, et al. (2021) Patterns of repeated diagnostic testing for COVID-19 in relation to patient characteristics and outcomes. Journal of Internal Medicine 289(5): 726-737. 10.1111/joim.13213
[523] Sami R, Soltaninejad F, Amra B, et al. (2020) A one-year hospital-based prospective COVID-19 open-cohort in the Eastern Mediterranean region: The Khorshid COVID Cohort (KCC) study. PLoS One 15(11): e0241537. 10.1371/journal.pone.0241537
[524] Sánchez Díaz JS, Peniche Moguel KG, González Escudero EA, et al. (2021) Glycosylated hemoglobin as a predictor of mortality in severe pneumonia by COVID-19. Expert Rev Respir Med. 10.1080/17476348.2021.1926988
[525] Sandeep B, Aakriti S, Vinitaa J, et al. (2020) Clinical Profile of First 1000 COVID-19 Cases Admitted at Tertiary Care Hospitals and the Correlates of their Mortality: An Indian Experience. medRxiv. 10.1101/2020.11.16.20232223
[526] Sands K, Wenzel R, McLean L, et al. (2020) Patient characteristics and admitting vital signs associated with COVID-19 related mortality among patients admitted with non-critical illness. Infection control and hospital epidemiology: 1-20. 10.1017/ice.2020.461

[527] Santos MM, Lucena EES, Lima KC, Brito AAC, Bay MB, Bonfada D (2020) Survival and predictors of deaths of patients hospitalised due to COVID-19 from a retrospective and multicentre cohort study in Brazil. Epidemiol Infect 148: e198. 10.1017/s0950268820002034
[528] Sapey E, Gallier S, Mainey C, et al. (2020) Ethnicity and risk of death in patients hospitalised for COVID-19 infection in the UK: an observational cohort study in an urban catchment area. BMJ Open Respir Res 7(1). 10.1136/bmjresp-2020-000644
[529] Sara T, Anna K, Per Å, Jan J, Patrik G-J (2020) Risk Factors for Mortality in Adult and Elderly COVID-19 Patients in a Swedish University Hospital. SSRN. 10.2139/ssrn.3678594
[530] Sarah H-F, Pablo M, Ignacio M-F, et al. (2020) COVID-19 Outcomes in 4712 consecutively confirmed SARS-CoV2 cases in the city of Madrid. medRxiv. 10.1101/2020.05.22.20109850
[531] Sarah-Jeanne S, Geetanjali DD, Qihan Y, et al. (2021) The Obesity-COVID-19 Relationship: How Much Do Diabetes and Age Matter? SSRN
[532] Schlicht K, Rohmann N, Geisler C, et al. (2020) Circulating levels of soluble Dipeptidylpeptidase-4 are reduced in human subjects hospitalized for severe COVID-19 infections. Int J Obes (Lond) 44(11): 2335-2338. 10.1038/s41366-020-00689-y
[533] Schmidt M, Hajage D, Demoule A, et al. (2021) Clinical characteristics and day-90 outcomes of 4244 critically ill adults with COVID-19: a prospective cohort study. Intensive Care Medicine 47(1): 60-73. 10.1007/s00134-020-06294-x
[534] Schönfeld D, Arias S, Bossio JC, Fernández H, Gozal D, Pérez-Chada D (2021) Clinical presentation and outcomes of the first patients with COVID-19 in Argentina: Results of 207079 cases from a national database. PLoS One 16(2): e0246793. 10.1371/journal.pone.0246793
[535] Seaton RA, Gibbons CL, Cooper L, et al. (2020) Survey of antibiotic and antifungal prescribing in patients with suspected and confirmed COVID-19 in Scottish hospitals. J Infect 81(6): 952-960. 10.1016/j.jinf.2020.09.024
[536] Shabrawishi M, Al-Gethamy MM, Naser AY, et al. (2020) Clinical, radiological and therapeutic characteristics of patients with COVID-19 in Saudi Arabia. PLoS One 15(8): e0237130-e0237130
[537] Shang J, Wang Q, Zhang HP, et al. (2021) The Relationship Between Diabetes Mellitus and COVID-19 Prognosis: A Retrospective Cohort Study in Wuhan, China. American Journal of Medicine 134(1): E6-E14. 10.1016/j.amjmed.2020.05.033
[538] Shaoping H, Min H, Xiaolu L, Ting Z, Hongzhou L (2020) Significance of neutrophil-to-lymphocyte ratio, platelet-to-lymphocyte ratio for predicting clinical outcomes in COVID-19. medRxiv. 10.1101/2020.05.04.20090431
[539] Rahman S, Singh K, Dhingra S, et al. (2020) The Double Burden of the COVID-19 Pandemic and Polypharmacy on Geriatric Population - Public Health Implications. Ther Clin Risk Manag 16: 1007-1022. 10.2147/tcrm.S272908
[540] Sheshah E, Sabico S, Albakr RM, et al. (2020) Prevalence of Diabetes, Management and Outcomes among Covid-19 Adult Patients Admitted in a Specialized Tertiary Hospital in Riyadh, Saudi Arabia. Diabetes Res Clin Pract: 108538. 10.1016/j.diabres.2020.108538
[541] Shi Y, Yu X, Zhao H, et al. (2020) Host susceptibility to severe COVID-19: a retrospective analysis of 487 case outside Wuhan. ResearchSquare. 10.21203/rs.3.rs-16021/v1
[542] Ravindra VM, Grandhi R, Delic A, et al. (2021) Impact of COVID-19 on the hospitalization, treatment, and outcomes of intracerebral and subarachnoid hemorrhage in the United States. PLoS One 16(4): e0248728. 10.1371/journal.pone.0248728
[543] Shu L, Wang XY, Li MQ, et al. (2021) Clinical characteristics of moderate COVID-19 patients aggravation in Wuhan Stadium Cabin Hospital: A 571 cases of retrospective cohort study. Journal of Medical Virology 93(2): 1133-1140. 10.1002/jmv.26414
[544] Simona I, Ana FL-A, Immaculada V, et al. (2020) First and second waves of coronavirus disease-19: A comparative study in hospitalized patients in Reus, Spain. medRxiv. 10.1101/2020.12.10.20246959
[545] Simone L, Chiara M, Emanuele N, et al. (2020) COVID-19 Disease - Temporal Analyses of Complete Blood Count Parameters Over Course of Illness, and Relationship to Patient Demographics and Management Outcomes in Survivors and Non-Survivors: A Longitudinal Descriptive Cohort Study. SSRN. 10.2139/ssrn.3634864
[546] Soares RDM, Mattos LR, Raposo LM (2020) Risk Factors for Hospitalization and Mortality due to COVID-19 in Espirito Santo State, Brazil. American Journal of Tropical Medicine and Hygiene 103(3): 1184-1190. 10.4269/ajtmh.20-0483
[547] Soh TV, Dzawani M, Noorlina N, Nik F, Norazmi A (2020) Clinical characteristics of severe acute respiratory syndrome Coronavirus 2 (SARS-CoV2) patients in Hospital Tengku Ampuan Afzan. Med J Malaysia 75(5): 479-484
[548] Song SL, Hays SB, Panton CE, et al. (2020) Statin Use Is Associated with Decreased Risk of Invasive Mechanical Ventilation in COVID-19 Patients: A Preliminary Study. Pathogens 9(9). 10.3390/pathogens9090759

[549] Soni SL, Kajal K, Yaddanapudi LN, et al. (2021) Demographic & clinical profile of patients with COVID-19 at a tertiary care hospital in north India. Indian J Med Res 153(1 & 2): 115-125. 10.4103/ijmr.IJMR_2311_20
[550] Sosa-Rubi SG, Seiglie JA, Chivardi C, et al. (2021) Incremental Risk of Developing Severe COVID-19 Among Mexican Patients With Diabetes Attributed to Social and Health Care Access Disadvantages. Diabetes Care 44(2): 373-380. 10.2337/dc20-2192
[551] Sourij H, Aziz F, Bräuer A, et al. (2020) COVID-19 fatality prediction in people with diabetes and prediabetes using a simple score upon hospital admission. Diabetes Obes Metab. 10.1111/dom.14256
[552] Souza GFdA, Praciano GdAF, Ferreira Neto OdC, et al. (2021) Factors associated with psychic symptomatology in diabetics during the COVID-19 pandemic. Rev Bras Saúde Mater Infant (Online) 21(supl.1): 177-186
[553] Sterling RK, Oakes T, Gal TS, Stevens MP, deWit M, Sanyal AJ (2020) The FIB-4 Index Is Associated with Need for Mechanical Ventilation and 30-day Mortality in Patients Admitted with COVID-19. The Journal of infectious diseases. 10.1093/infdis/jiaa550
[554] Stoian AP, Pricop-Jeckstadt M, Pana A, et al. (2020) Death by SARS-CoV 2: a Romanian COVID-19 multi-centre comorbidity study. Scientific Reports 10(1). 10.1038/s41598-020-78575-w
[555] Suardi LR, Pallotto C, Esperti S, et al. (2020) Risk factors for non-invasive/invasive ventilatory support in patients with COVID-19 pneumonia: A retrospective study within a multidisciplinary approach. International Journal of Infectious Diseases 100: 258-263. 10.1016/j.ijid.2020.09.012
[556] Su-Jeong K, Chang Gyu J, Ji Yeon L, Gunwoo K, Hyun Jung J, Han-Ki P (2020) Characterization of asthma and risk factors for delayed SARS-CoV-2 clearance in adult COVID-19 inpatients in Daegu. 10.22541/AU.159620967.71557506
[557] Sun Y, Guan X, Jia L, et al. (2021) Independent and combined effects of hypertension and diabetes on clinical outcomes in patients with COVID-19: A retrospective cohort study of Huoshen Mountain Hospital and Guanggu Fangcang Shelter Hospital. J Clin Hypertens (Greenwich) 23(2): 218-231. 10.1111/jch.14146
[558] Surendra H, Elyazar IR, Djaafara BA, et al. (2021) Clinical characteristics and mortality associated with COVID-19 in Jakarta, Indonesia: A hospital-based retrospective cohort study. Lancet Reg Health West Pac 9: 100108. 10.1016/j.lanwpc.2021.100108
[559] Sutter W, Duceau B, Vignac M, et al. (2020) Association of diabetes and outcomes in patients with COVID-19: Propensity score-matched analyses from a French retrospective cohort. Diabetes Metab 47(4): 101222. 10.1016/j.diabet.2020.101222
[560] Svensson P, Hofmann R, Häbel H, Jernberg T, Nordberg P (2021) Association between cardiometabolic disease and severe COVID-19: a nationwide case-control study of patients requiring invasive mechanical ventilation. BMJ Open 11(2): e044486. 10.1136/bmjopen-2020-044486
[561] Swandari P, Ronny I, Krispinus D, et al. (2020) Characteristics of COVID-19 fatality cases in East Kalimantan, Indonesia. medRxiv. 10.1101/2020.08.01.20166470
[562] Szente Fonseca SN, de Queiroz Sousa A, Wolkoff AG, et al. (2020) Risk of hospitalization for Covid-19 outpatients treated with various drug regimens in Brazil: Comparative analysis. Travel Med Infect Dis 38: 101906. 10.1016/j.tmaid.2020.101906
[563] Tabrizi FM, Rasmi Y, Hosseinzadeh E, et al. (2021) DIABETES IS ASSOCIATED WITH INCREASED MORTALITY AND DISEASE SEVERITY IN HOSPITALIZED PATIENTS WITH COVID-19. Excli Journal 20: 444-453. 10.17179/excli2021-3403
[564] Tanaka S, De Tymowski C, Assadi M, et al. (2020) Lipoprotein concentrations over time in the intensive care unit COVID-19 patients: Results from the ApoCOVID study. PLoS One 15(9): e0239573. 10.1371/journal.pone.0239573
[565] Tang Q, Liu Y, Fu Y, et al. (2020) A Comprehensive Evaluation of Early Predictors of Disease Progression in Patients with COVID-19: A Case Control Study. ResearchSquare. 10.21203/rs.3.rs-50527/v1
[566] Tartof SY, Qian L, Hong V, et al. (2020) Obesity and Mortality Among Patients Diagnosed With COVID-19: Results From an Integrated Health Care Organization. Ann Intern Med 173(10): 773-781. 10.7326/m20-3742
[567] Tehrani S, Killander A, Aastrand P, Jakobsson J, Gille-Johnson P (2021) Risk factors for death in adult COVID-19 patients: Frailty predicts fatal outcome in older patients. International Journal of Infectious Diseases 102: 415-421. 10.1016/j.ijid.2020.10.071
[568] Terlecki M, Wojciechowska W, Klocek M, et al. (2021) Association between cardiovascular disease, cardiovascular drug therapy, and in-hospital outcomes in patients with COVID-19: data from a large single-center registry in Poland. Kardiol Pol. 10.33963/kp.15990
[569] Tetlow S, Segiet-Swiecicka A, O'Sullivan R, et al. (2020) ACE-Inhibitors, Angiotensin Receptor Blockers and Endothelial Injury in COVID-19. Journal of internal medicine. 10.1111/joim.13202

[570] Thompson JV, Meghani NJ, Powell BM, et al. (2020) Patient characteristics and predictors of mortality in 470 adults admitted to a district general hospital in England with Covid-19. Epidemiology and Infection 148. 10.1017/s0950268820002873
[571] Tigist WL, Ishmael SH, Endalkachew HM, et al. (2020) Characteristics and outcome profile of Hospitalized African COVID-19 patients: The Ethiopian Context. medRxiv. 10.1101/2020.10.27.20220640
[572] Tigist WL, Kindalem GA, Ishmael SH, et al. (2020) COVID-19 Disease Severity and Determinants among Ethiopian Patients: A study of the Millennium COVID-19 Care Center. medRxiv. 10.1101/2020.10.09.20209999
[573] Toh DJ, Rowe E, Nelson R, et al. (2021) Outcomes for the first wave of hospitalised patients with COVID-19 in the South Australian context: a retrospective audit. Intern Med J 51(2): 189-198. 10.1111/imj.15106
[574] Tomi J, Sharon N, Patricia K, Kuan-lin H (2020) Mortality and risk factors among US Black, Hispanic, and White patients with COVID-19. medRxiv. 10.1101/2020.09.08.20190686
[575] Tomi J, Sharon N, Patricia K, Kuan-lin H (2020) Sex-specificity of mortality risk factors among hospitalized COVID-19 patients in New York City: prospective cohort study. medRxiv. 10.1101/2020.07.29.20164640
[576] Toussie D, Voutsinas N, Finkelstein M, et al. (2020) Clinical and Chest Radiography Features Determine Patient Outcomes in Young and Middle-aged Adults with COVID-19. Radiology 297(1): E197-E206. 10.1148/radiol.2020201754
[577] Tsai S, Nguyen H, Ebrahimi R, et al. (2021) COVID-19 associated mortality and cardiovascular disease outcomes among US women veterans. Sci Rep 11(1): 8497. 10.1038/s41598-021-88111-z
[578] Tu Y, Yang P, Zhou Y, et al. (2021) Risk factors for mortality of critically ill patients with COVID-19 receiving invasive ventilation. Int J Med Sci 18(5): 1198-1206. 10.7150/ijms.50039
[579] Turcotte JJ, Meisenberg BR, MacDonald JH, et al. (2020) Risk factors for severe illness in hospitalized Covid-19 patients at a regional hospital. PLoS One 15(8): e0237558. 10.1371/journal.pone.0237558
[580] Turgay Yıldırım Ö, Kaya Ş (2021) The atherogenic index of plasma as a predictor of mortality in patients with COVID-19. Heart Lung 50(2): 329-333. 10.1016/j.hrtlng.2021.01.016
[581] Ursula KW, Jason DM, Katherine M, Alyssa C, Marie B, Scott AR (2021) Comorbidity Risk Factors Contributing to COVID-19 Related Deaths in Florida, March 1, 2020-January 16, 2021. medRxiv. 10.1101/2021.04.14.21255434
[582] Vahabi N, Salehi M, Duarte JD, Mollalo A, Michailidis G (2021) County-level longitudinal clustering of COVID-19 mortality to incidence ratio in the United States. Sci Rep 11(1): 3088. 10.1038/s41598-021-82384-0
[583] van der Velden PG, Marchand M, Cuelenaere B, Das M (2020) Pre-outbreak determinants of perceived risks of corona infection and preventive measures taken. A prospective population-based study. Plos One 15(7). 10.1371/journal.pone.0234600
[584] Varma E, Shankaranarayanan D, Neupane SP, et al. (2020) High c-reactive protein and D-dimer on admission predict the development of AKI in patients hospitalized with COVID-19. Journal of The American Society of Nephrology 31
[585] Velasco-Rodríguez D, Alonso-Dominguez JM, Vidal Laso R, et al. (2021) Development and validation of a predictive model of in-hospital mortality in COVID-19 patients. PLoS One 16(3): e0247676. 10.1371/journal.pone.0247676
[586] Vena A, Giacobbe DR, Di Biagio A, et al. (2020) Clinical characteristics, management and in-hospital mortality of patients with COVID-19 In Genoa, Italy. Clinical microbiology and infection : the official publication of the European Society of Clinical Microbiology and Infectious Diseases. 10.1016/j.cmi.2020.07.049
[587] Vergara P, Rossi L, Biagi A, et al. (2021) Role of comorbidities on the mortality in patients with SARS-CoV-2 infection: an Italian cohort study. Minerva Med. 10.23736/s0026-4806.21.07187-1
[588] Waasila J, Cheryl C, Maureen M, et al. (2020) COVID-19 in-hospital mortality in South Africa: the intersection of communicable and non-communicable chronic diseases in a high HIV prevalence setting. medRxiv. 10.1101/2020.12.21.20248409
[589] Wang F, Cao J, Yu Y, et al. (2020) Epidemiological characteristics of patients with severe COVID-19 infection in Wuhan, China: evidence from a retrospective observational study. Int J Epidemiol. 10.1093/ije/dyaa180
[590] Wang G, Wu C, Zhang Q, et al. (2020) Epidemiological and Clinical Features of Corona Virus Disease 2019 (COVID-19) in Changsha, China. SSRN. 10.2139/ssrn.3548770
[591] Wang G, Wu C, Zhang Q, et al. (2020) Clinical characteristics and the risk factors for severe events of elderly coronavirus disease 2019 patients. Zhong Nan Da Xue Xue Bao Yi Xue Ban 45(5): 542-548. 10.11817/j.issn.1672-7347.2020.200292

[592] Wang J, Cooper JM, Gokhale K, et al. (2021) Association of Metformin with Susceptibility to COVID-19 in People with Type 2 Diabetes. J Clin Endocrinol Metab 106(5): 1255-1268. 10.1210/clinem/dgab067
[593] Wang J, Zhu L, Liu LG, et al. (2020) Overweight and Obesity are Risk Factors of Severe Illness in Patients with COVID-19. Obesity 28(11): 2049-2055. 10.1002/oby.22979
[594] Wang K, Qiu Z, Liu J, et al. (2020) Analysis of the clinical characteristics of 77 COVID-19 deaths. Sci Rep 10(1): 16384. 10.1038/s41598-020-73136-7
[595] Wang L, Foer D, Bates DW, Boyce JA, Zhou L (2020) Risk factors for hospitalization, intensive care, and mortality among patients with asthma and COVID-19. J Allergy Clin Immunol 146(4): 808-812
[596] Wang S, Chen Z, Lin Y, et al. (2020) Clinical characteristics of 199 discharged patients with COVID-19 in Fujian Province: A multicenter retrospective study between January 22nd and February 27th, 2020. PLoS One 15(11): e0242307. 10.1371/journal.pone.0242307
[597] Wang S, Chen Z, Lin Y, et al. (2020) Clinical characteristics of COVID-19 in Fujian Province: a multicenter retrospective study. ResearchSquare. 10.21203/rs.3.rs-21268/v1
[598] Wang SF, Ma P, Zhang SJ, et al. (2020) Fasting blood glucose at admission is an independent predictor for 28-day mortality in patients with COVID-19 without previous diagnosis of diabetes: a multi-centre retrospective study. Diabetologia 63(10): 2102-2111. 10.1007/s00125-020-05209-1
[599] Wang W, Zhao Z, Liu X, et al. (2020) Clinical features and potential risk factors for discerning the critical cases and predicting the outcome of patients with COVID-19. J Clin Lab Anal 34(10): e23547. 10.1002/jcla.23547
[600] Wang Z, Wang Z (2021) Identification of risk factors for in-hospital death of COVID - 19 pneumonia -- lessions from the early outbreak. BMC Infect Dis 21(1): 113. 10.1186/s12879-021-05814-4
[601] Wei W, Sivapalasingam S, Mellis S, Geba GP, Jalbert JJ (2021) A Retrospective Study of COVID-19-Related Urgent Medical Visits and Hospitalizations After Outpatient COVID-19 Diagnosis in the US. Adv Ther: 1-18. 10.1007/s12325-021-01742-6
[602] Wen XS, Jiang D, Gao L, et al. (2021) Clinical characteristics and predictive value of lower CD4(+)T cell level in patients with moderate and severe COVID-19: a multicenter retrospective study. BMC Infect Dis 21(1): 57. 10.1186/s12879-020-05741-w
[603] Wollenstein-Betech S, Silva AAB, Fleck JL, Cassandras CG, Paschalidis IC (2020) Physiological and socioeconomic characteristics predict COVID-19 mortality and resource utilization in Brazil. PLoS One 15(10): e0240346. 10.1371/journal.pone.0240346
[604] Wonjun J, Kyungmin H, Minsun K, et al. (2020) Effect of underlying comorbidities on the infection and severity of COVID-19 in South Korea. medRxiv. 10.1101/2020.05.08.20095174
[605] Woolcott OO, Bergman RN (2020) Mortality Attributed to COVID-19 in High-Altitude Populations. High Alt Med Biol. 10.1089/ham.2020.0098
[606] Woolcott OO, Castilla-Bancayán JP (2021) The effect of age on the association between diabetes and mortality in adult patients with COVID-19 in Mexico. Sci Rep 11(1): 8386. 10.1038/s41598-021-88014-z
[607] Wu JF, Huang JQ, Zhu GC, et al. (2020) Elevation of blood glucose level predicts worse outcomes in hospitalized patients with COVID-19: a retrospective cohort study. Bmj Open Diabetes Research & Care 8(1). 10.1136/bmjdrc-2020-001476
[608] Wu SS, Xue LP, Legido-Quigley H, et al. (2020) Understanding factors influencing the length of hospital stay among non-severe COVID-19 patients: A retrospective cohort study in a Fangcang shelter hospital. Plos One 15(10). 10.1371/journal.pone.0240959
[609] Wu X, Wang T, Zhou Y, et al. (2020) Different Laboratory Abnormalities in COVID-19 Patients with Hypertension or Diabetes. Virol Sin: 1-4. 10.1007/s12250-020-00296-1
[610] Xie J, Zu YH, Alkhatib A, et al. (2021) Metabolic Syndrome and COVID-19 Mortality Among Adult Black Patients in New Orleans. Diabetes Care 44(1): 188-193. 10.2337/dc20-1714
[611] Xu M, Yang W, Huang T, Zhou J (2020) Diabetic patients with COVID-19 need more attention and better glycemic control. World J Diabetes 11(12): 644-653. 10.4239/wjd.v11.i12.644
[612] Yacobitti A, Otero L, Doldan Arrubarrena V, et al. (2021) Clinical characteristics of vulnerable populations hospitalized and diagnosed with COVID-19 in Buenos Aires, Argentina. Sci Rep 11(1): 9679. 10.1038/s41598-021-87552-w
[613] Yan Q, Zuo PY, Cheng L, et al. (2021) Acute Kidney Injury Is Associated With In-hospital Mortality in Older Patients With COVID-19. Journals of Gerontology Series a-Biological Sciences and Medical Sciences 76(3): 456-462. 10.1093/gerona/glaa181
[614] Yan X, Han X, Peng D, et al. (2020) Clinical Characteristics and Prognosis of 218 Patients With COVID-19: A Retrospective Study Based on Clinical Classification. Front Med (Lausanne) 7: 485. 10.3389/fmed.2020.00485

[615] Yan YM, Yang F, Zhu XX, et al. (2020) Analysis of clinical features and pulmonary CT features of coronavirus disease 2019 (COVID-19) patients with diabetes mellitus. Endokrynologia Polska 71(5): 367-375. 10.5603/EP.a2020.0055
[616] Yang P, Wang N, Wang J, Luo A, Gao F, Tu Y (2020) Admission fasting plasma glucose is an independent risk factor for 28-day mortality in patients with COVID-19. J Med Virol. 10.1002/jmv.26608
[617] Yao X, Liu S, Wang J, et al. (2020) The clinical characteristics and prognosis of COVID-19 patients with cerebral strokeï¼ša retrospective study of 113 cases from one single center. Eur j neurosci
[618] Yao Y, Tian J, Meng X, Kan H, Zhou L, Wang W (2020) Progression of Severity in Coronavirus Disease 2019 Patients Before Treatment and a Self-Assessment Scale to Predict Disease Severity. ResearchSquare. 10.21203/rs.3.rs-96164/v1
[619] Yazdanpanah Y, French CCI (2021) Impact on disease mortality of clinical, biological, and virological characteristics at hospital admission and overtime in COVID-19 patients. Journal of Medical Virology 93(4): 2149-2159. 10.1002/jmv.26601
[620] Yeon Hee K, YeHee K, Soo Young K, Kwangsoo K (2020) How closely is COVID-19 related to HCoV, SARS, and MERS? : Clinical comparison of coronavirus infections and identification of risk factors influencing the COVID-19 severity using common data model (CDM). medRxiv. 10.1101/2020.11.23.20237487
[621] Yetmar ZA, Challener DW, Tleyjeh IM, et al. (2021) Association Between Chronic Statin Use and 30-Day Mortality in Hospitalized Patients With COVID-19. Mayo Clin Proc Innov Qual Outcomes 5(2): 442-446. 10.1016/j.mayocpiqo.2021.02.002
[622] Yizhou Y, Marco T, Rebeka P, Nuno Santos L, Martins LM (2020) Alzheimer's and Parkinson's diseases predict different COVID-19 outcomes, a UK Biobank study. medRxiv. 10.1101/2020.11.05.20226605
[623] Yogini VC, Francesco Z, Clare LG, et al. (2020) Patterns of Multimorbidity and Risk of Severe SARS-CoV-2 Infection: an observational study in the U.K. medRxiv. 10.1101/2020.10.21.20216721
[624] Yomayusa N, Acevedo K, Reina A, et al. (2020) Clinical course, biomarkers, management and outcomes of patients hospitalised due to COVID-19 in Colombia. ResearchSquare. 10.21203/rs.3.rs-57978/v1
[625] Yordanov Y, Dinh A, Bleibtreu A, et al. (2021) Clinical characteristics and factors associated with hospital admission or death in 43,103 adult outpatients with COVID-19 managed with the Covidom telesurveillance solution: a prospective cohort study. Clin Microbiol Infect. 10.1016/j.cmi.2021.04.010
[626] Yoshida Y, Gillet SA, Brown MI, et al. (2021) Clinical characteristics and outcomes in women and men hospitalized for coronavirus disease 2019 in New Orleans. Biol Sex Differ 12(1): 20. 10.1186/s13293-021-00359-2
[627] Young, Bob, Valabhji, Jonathan (2020) Type 1 and Type 2 Diabetes and COVID-19 Related Mortality in England: A Whole Population Study.
[628] Yousaf A, Studney M, El-Hamdani M, Patton-Tackett E (2020) Cardiovascular Outcomes of Diabetic Covid-19 Patients: A Multi-center Research Network Study. Circulation 142
[629] Yuan N, Ji H, Sun N, et al. (2021) Pseudo-safety in a cohort of patients with COVID-19 discharged home from the emergency department. Emerg Med J. 10.1136/emermed-2020-210041
[630] Yuchang Z, Jiajuan Y, Chengzhong X, et al. (2020) The Effects of Diabetes and Hypertension on the Severity of COVID-19 — Yichang, Hubei Province, 2020. 2(43). 10.46234/CCDCW2020.135
[631] Yun K, Lee JS, Kim EY, Chandra H, Oh BL, Oh J (2020) Severe COVID-19 Illness: Risk Factors and Its Burden on Critical Care Resources. Front Med (Lausanne) 7: 583060. 10.3389/fmed.2020.583060
[632] Yunfei L, Yong F, Bo W, et al. (2020) Clinical Characteristics and Risk Factors for Developed COVID-19 Patients Transferring to Designated Hospital in Jianghan Fangcang Shelter Hospital: A Retrospective, Observational Study. SSRN. 10.2139/ssrn.3576896
[633] Yupari-Azabache I, Bardales-Aguirre L, Rodriguez-Azabache J, Barros-Sevillano JS, Rodríguez-Diaz A (2021) Covid-19 mortality risk factors in hospitalized patients: A logistic regression model. 21(1)
[634] Zahra R-E, Celeste M, Mae SB, et al. (2020) Greater risk of severe COVID-19 in non-White ethnicities is not explained by cardiometabolic, socioeconomic, or behavioural factors, or by 25(OH)-vitamin D status: study of 1,326 cases from the UK Biobank. medRxiv. 10.1101/2020.06.01.20118943
[635] Zali A, Gholamzadeh S, Mohammadi G, et al. (2020) Baseline Characteristics and Associated Factors of Mortality in COVID-19 Patients; an Analysis of 16000 Cases in Tehran, Iran. Arch Acad Emerg Med 8(1): e70
[636] Zandkarimi E, Moradi G, Mohsenpour B (2020) The Prognostic Factors Affecting the Survival of Kurdistan Province COVID-19 Patients: A Cross-sectional Study From February to May 2020. Int J Health Policy Manag. 10.34172/ijhpm.2020.155

[637] Zengin S, Avcı S, Yılmaz S (2020) Clinical and basic cardiovascular features of patients with COVID-19 admitted to a tertiary care center in Turkey. Journal of Surgery & Medicine (JOSAM) 4(5): 367-370. 10.28982/josam.727935
[638] Zhang B, Dong C, Li S, Song X, Wei W, Liu L (2020) Triglyceride to High-Density Lipoprotein Cholesterol Ratio is an Important Determinant of Cardiovascular Risk and Poor Prognosis in Coronavirus Disease-19: A Retrospective Case Series Study. Diabetes Metab Syndr Obes 13: 3925-3936. 10.2147/dmso.S268992
[639] Zhang C, Fu H, Zhang L, et al. (2020) Clinical characteristics study of elderly patients aged 75 or older with COVID-19 pneumonia in China. ResearchSquare. 10.21203/rs.3.rs-67737/v1
[640] Zhang H, Cao X, Kong M, et al. (2020) Clinical and hematological characteristics of 88 patients with COVID-19. Int J Lab Hematol 42(6): 780-787
[641] Zhang L, Fan T, Yang S, et al. (2020) Comparison of clinical characteristics of COVID-19 between elderly patients and young patients: a study based on a 28-day follow-up. Aging (Albany NY) 12(20): 19898-19910. 10.18632/aging.104077
[642] Zhang S, Liu L, Yang B, et al. (2020) Clinical characteristics of 134 convalescent patients with COVID-19 in Guizhou, China. Respir Res 21(1): 314. 10.1186/s12931-020-01580-0
[643] Zhang W, Li C, Xu Y, et al. (2021) Hyperglycemia and Correlated High Levels of Inflammation Have a Positive Relationship with the Severity of Coronavirus Disease 2019. Mediators Inflamm 2021: 8812304. 10.1155/2021/8812304
[644] Zhang Y, Sha T, Wu F, et al. (2021) Hypertension in Patients Hospitalized with COVID-19 in Wuhan, China. Int Heart J 62(2): 337-343. 10.1536/ihj.20-323
[645] Zhao Y, Nie H-X, Hu K, et al. (2020) Abnormal immunity of non-survivors with COVID-19: predictors for mortality. Infect Dis Poverty 9(1): 108-108
[646] Zheng KI, Gao F, Wang XB, et al. (2020) Obesity as a risk factor for greater severity of COVID-19 in patients with metabolic associated fatty liver disease. Metabolism: clinical and experimental 108: 154244. 10.1016/j.metabol.2020.154244
[647] Zhong R, Chen L, Zhang Q, et al. (2020) Which Factors, Smoking, Drinking Alcohol, Betel Quid Chewing, or Underlying Diseases, Are More Likely to Influence the Severity of COVID-19? Front Physiol 11: 623498. 10.3389/fphys.2020.623498
[648] Zhong Y, Zhao L, Wu G, et al. (2020) Impact of renin-angiotensin system inhibitors use on mortality in severe COVID-19 patients with hypertension: a retrospective observational study. J Int Med Res 48(12): 300060520979151. 10.1177/0300060520979151
[649] Zhu B, Jin SW, Wu LP, et al. (2020) J-shaped association between fasting blood glucose levels and COVID-19 severity in patients without diabetes. Diabetes Research and Clinical Practice 168. 10.1016/j.diabres.2020.108381
[650] Zhu Z, Hasegawa K, Ma B, Fujiogi M, Camargo CA, Jr., Liang L (2020) Association of obesity and its genetic predisposition with the risk of severe COVID-19: Analysis of population-based cohort data. Metabolism 112: 154345. 10.1016/j.metabol.2020.154345
[651] Zimering MB, Razzaki T, Tsang T, Shin JJ (2020) Inverse Association between Serotonin 2A Receptor Antagonist Medication Use and Mortality in Severe COVID-19 Infection. Endocrinol Diabetes Metab J 4(4): 1-5
[652] Zinellu A, Arru F, De Vito A, et al. (2021) The De Ritis ratio as prognostic biomarker of in-hospital mortality in COVID-19 patients. Eur J Clin Invest 51(1): e13427. 10.1111/eci.13427
[653] Zinu P, Nadia S, Chun Shing Jefferson W, Karen A, Michael Anthony M (2020) Obesity, old age and frailty are the true risk factors for COVID-19 mortality and not chronic disease or ethnicity in Croydon. medRxiv. 10.1101/2020.08.12.20156257
[654] Zou L, Dai L, Zhang Y, et al. (2020) Clinical Characteristics and Risk Factors for Disease Severity and Death in Patients With Coronavirus Disease 2019 in Wuhan, China. Front Med (Lausanne) 7: 532. 10.3389/fmed.2020.00532
[655] Boytsov SA, Pogosova NV, Paleev FN, et al. (2021) Clinical Characteristics and Factors Associated with Poor Outcomes in Hospitalized Patients with Novel Coronavirus Infection COVID-19. Kardiologiia 61(2): 4-14. 10.18087/cardio.2021.2.n1532
[656] Drapkina OM, Karpov OE, Lukyanov MM, et al. (2020) Prospective in-hospital registry of patients with suspected or documented COVID-19 infection and community-acquired pneumonia (TARGET-VIP): characteristics of patients and assessment of in-hospital outcomes. Cardiovascular Therapy and Prevention 19(6). 10.15829/1728-8800-2020-2727
[657] Georges JL, Cochet H, Roger G, et al. (2020) Association entre l'hypertension artérielle, les traitements inhibiteurs du système rénine angiotensine et les formes graves de COVID-19. Étude prospective monocentrique française./ [Association of hypertension and antihypertensive agents and the severity of COVID-19 pneumonia. A monocentric French prospective study]. 69(5)
[658] Lu J, Zhang Y, Cheng G, et al. (2020) [Clinical characteristics and outcomes of adult critically ill patients with COVID-19 in Honghu, Hubei Province]. Nan fang yi ke da xue xue bao = Journal of Southern Medical University 40(6): 778-785. 10.12122/J.ISSN.1673-4254.2020.06.02

[659] Navarrete-Mejia PJ, Lizaraso-Soto FA, Velasco-Guerrero JC, Loro-Chero LM (2020) Diabetes mellitus and arterial hypertension as a risk factor of mortality in patients with Covid-19. 13(4)
[660] Ramos-Peñafiel CO, Santos-González B, Flores-López EN, et al. (2020) Usefulness of the neutrophil-to-lymphocyte, monocyte-to-lymphocyte and lymphocyte-to-platelet ratios for the prognosis of COVID-19-associated complications. Gac Med Mex 156(5): 405-411. 10.24875/gmm.M20000428
[661] Vila-Corcoles A, Ochoa-Gondar O, Torrente-Fraga C, et al. (2020) Evaluation of incidence and risk profile for suffering Covid-19 infection by underlying conditions among middle-aged and older adults in Tarragona. Revista Espanola De Salud Publica 94
[662] Yacobitti A, Otero LJ, Doldan Arruabarrena VS, et al. (2021) [Hospitalized population diagnosed with Covid-19 in public health centers in the southeastern region of Greater Buenos Aires]. Rev Fac Cien Med Univ Nac Cordoba 78(1): 17-24. 10.31053/1853.0605.v78.n1.31146
[663] 覃伟, 胡冰竹, 张竹, et al. (2020) Clinical Characteristics and Death Risk Factors of Severe COVID-19/ 重症新型冠状病毒肺炎的临床特征及死亡因素分析.(08)
[664] Abd El-Raheem GOH, Mohamed DSI, Yousif MAA, Elamin HES (2021) Characteristics and severity of COVID-19 among Sudanese patients during the waves of the pandemic. Sci Afr 14: e01033. 10.1016/j.sciaf.2021.e01033
[665] Aleanizy FS, Alqahtani FY, Alanazi MS, et al. (2021) Clinical characteristics and risk factors of patients with severe COVID-19 in Riyadh, Saudi Arabia: A retrospective study. J Infect Public Health 14(9): 1133-1138. 10.1016/j.jiph.2021.07.014
[666] AlKhafaji D, Al Argan R, Albaker W, et al. (2022) The Impact of Vitamin D Level on the Severity and Outcome of Hospitalized Patients with COVID-19 Disease. Int J Gen Med 15: 343-352. 10.2147/ijgm.S346169
[667] Aziz F, Reisinger A, Aberer F, et al. (2021) Simplified Acute Physiology Score 3 Performance in Austrian COVID-19 Patients with and Without Diabetes. ResearchSquare. 10.21203/rs.3.rs-1192557/v1
[668] Bhaskaran K, Bacon S, Evans SJ, et al. (2021) Factors associated with deaths due to COVID-19 versus other causes: population-based cohort analysis of UK primary care data and linked national death registrations within the OpenSAFELY platform. Lancet Reg Health Eur 6: 100109. 10.1016/j.lanepe.2021.100109
[669] Bushman D, Davidson A, Pathela P, et al. (2021) Risk Factors for Death Among Hospitalized Patients Aged 21-64 Years Diagnosed with COVID-19-New York City, March 13-April 9, 2020. J Racial Ethn Health Disparities: 1-16. 10.1007/s40615-021-01098-1
[670] Danne TKO (2021) COVID-19 („coronavirus disease 2019“) und Typ-1-Diabetes. Der Diabetologe. 10.1007/s11428-021-00776-2
[671] Diedisheim M, Dancoisne E, Gautier JF, et al. (2021) Diabetes Increases Severe COVID-19 Outcomes Primarily in Younger Adults. J Clin Endocrinol Metab 106(9): e3364-e3368. 10.1210/clinem/dgab393
[672] Emi U, Masahide H, Kazuki S, et al. (2021) Impact of Untreated Diabetes and COVID-19 Related Diabetes on Severe COVID-19. SSRN
[673] Gokhale K, Mostafa SA, Wang J, et al. (2022) The clinical profile and associated mortality in people with and without diabetes with Coronavirus disease 2019 on admission to acute hospital services. Endocrinol Diabetes Metab 5(1): e00309. 10.1002/edm2.309
[674] Kim SW, Jeon JH, Moon JS, Kim MK (2021) High Fibrosis-4 Index Is Related with Worse Clinical Outcome in Patients with Coronavirus Disease 2019 and Diabetes Mellitus: A Multicenter Observational Study. Endocrinol Metab (Seoul) 36(4): 800-809. 10.3803/EnM.2021.1040
[675] Rysz S, Fagerlund M, Rimes-Stigare C, Larsson E, Jalde F, Mårtensson J (2021) Covid -19, An Acute on Chronic Metabolic Syndrome? HbA1c-A Marker of Severe Infection. ResearchSquare. 10.21203/rs.3.rs-518074/v1
[676] Rysz S, Jonsson Fagerlund M, Rimes-Stigare C, Larsson E, Campoccia Jalde F, Mårtensson J (2022) Chronic dysglycemia and risk of SARS-CoV-2 associated respiratory failure in hospitalized patients. Acta Anaesthesiol Scand 66(1): 48-55. 10.1111/aas.13982
[677] Savino M, Santhakumaran S, Evans KM, et al. (2021) Outcomes of patients with COVID-19 on kidney replacement therapy: a comparison among modalities in England. Clin Kidney J 14(12): 2573-2581. 10.1093/ckj/sfab160
[678] Sharif N, Ahmed SN, Opu RR, et al. (2021) Prevalence and impact of diabetes and cardiovascular disease on clinical outcome among patients with COVID-19 in Bangladesh. Diabetes Metab Syndr 15(3): 1009-1016. 10.1016/j.dsx.2021.05.005
[679] Tavakoli N, Hashemi-Madani N, Malek M, et al. (2021) Comorbidities Associated with Complicated Hospital Course and Death in COVID-19 Patients: A Retrospective Study from Iran. ResearchSquare. 10.21203/rs.3.rs-1036149/v1

[680] Velásquez García HA, Wilton J, Smolina K, et al. (2021) Mental Health and Substance Use Associated with Hospitalization among People with COVID-19: A Population-Based Cohort Study. Viruses 13(11). 10.3390/v13112196
[681] Zhou Y, Yang J, Xu C, et al. (2020) The Effects of Diabetes and Hypertension on the Severity of COVID-19 - Yichang, Hubei Province, 2020. China CDC Wkly 2(43): 833-837. 10.46234/ccdcw2020.135
[682] 孙海燕, 冯晓云, 林毅, et al. (2020) Clinical Characteristics of Patients with Diabetes Mellitus Complicated with COVID-19. 中华糖尿病杂志
[683] Arvind KS, Vaseem Naheed B, Sonali S, et al. (2021) Cardiovascular Risk Factors and Outcomes in COVID-19: Hospital-Based Prospective Study in India. medRxiv. 10.1101/2021.09.19.21263788
[684] Al Mutair A, Al Mutairi A, Alhumaid S, et al. (2021) Examining and investigating the impact of demographic characteristics and chronic diseases on mortality of COVID-19: Retrospective study. PLoS One 16(9): e0257131. 10.1371/journal.pone.0257131
[685] Abbas HM, Nassir KF, Al Khames Aga QA, et al. (2020) Presenting characteristics, smoking versus diabetes and outcome among patients hospitalized with COVID-19. Journal of medical virology 93(3): 1556-1567. 10.1002/jmv.26487
[686] Wang W, Chai Z, Cooper ME, et al. (2021) High Fasting Blood Glucose Level With Unknown Prior History of Diabetes Is Associated With High Risk of Severe Adverse COVID-19 Outcome. Front Endocrinol (Lausanne) 12: 791476. 10.3389/fendo.2021.791476
[687] Natanael JS, Rita CR-S, Andrea JFF, et al. (2021) Combined association of obesity and other cardiometabolic diseases with severe COVID-19 outcomes: a nationwide cross-sectional study of 21,773 Brazilian adult and elderly inpatients. medRxiv. 10.1101/2021.05.14.21257204
[688] Héctor Alexander Velásquez G, James W, Kate S, et al. (2021) Mental health and substance use associated with hospitalization among people with laboratory confirmed diagnosis of COVID-19 in British Columbia: a population-based cohort study. medRxiv. 10.1101/2021.08.26.21262697
[689] Cicilia W, Nurul Ulfah H, Yenny S, Tiwi N, Ign. Erik Sapta Y, Tri Budi WR (2021) Etiology COVID-19 mortality in Jakarta hospital in the first COVID-19 outbreak in Indonesia. Current Pediatric Research 25(3)
[690] (2021) Patient care and clinical outcomes for patients with COVID-19 infection admitted to African high-care or intensive care units (ACCCOS): a multicentre, prospective, observational cohort study. Lancet 397(10288): 1885-1894. 10.1016/s0140-6736(21)00441-4
[691] Abdallah E, Al Helal B, Asad R, et al. (2021) Incidence and Outcomes of Acute Kidney Injury in Critically Ill Patients with Coronavirus Disease 2019. Saudi J Kidney Dis Transpl 32(1): 84-91. 10.4103/1319-2442.318551
[692] AbdelGhaffar MM, Omran D, Elgebaly A, et al. (2022) Prediction of mortality in hospitalized Egyptian patients with Coronavirus disease-2019: A multicenter retrospective study. PLoS One 17(1): e0262348. 10.1371/journal.pone.0262348
[693] Abdelwahab HW, Shaltout SW, Fouda AM, et al. (2021) Sex difference in hospitalized patients with COVID-19 infection. Chest Disease Reports 8(1). 10.4081/CDR.2021.9842
[694] Abidin NHZ, Lee CK, Zakaria SA, Ramaiah Y, Hilmi ZS, Maule SS (2021) Clinical course and outcomes of ventilated critically ill COVID-19 patients in Selangor state, Malaysia. Journal of Emergency and Critical Care Medicine 5(January). 10.21037/jeccm-20-82
[695] Abo ElNaga HH, AbdelHalim HA, Abdellatif M, et al. (2021) Wallop of Symptoms and Co-morbidities on COVID-19 Outcome. Open Respir Med J 15: 46-51. 10.2174/1874306402115010046
[696] Adejumo OA, Ogunniyan T, Adesola S, et al. (2021) Clinical presentation of COVID-19-positive and -negative patients in Lagos Nigeria: A comparative study. Niger Postgrad Med J 28(2): 75-80. 10.4103/npmj.npmj_547_21
[697] Adhani R, Kania D, Purwaningayu JH, et al. (2022) Risk Factors of Hipertension and Diabetes Mellitus on COVID-19 Mortality. Open Access Macedonian Journal of Medical Sciences 10: 145-150. 10.3889/oamjms.2022.6951
[698] Afify S, Eysa B, Hamid FA, et al. (2021) Survival and outcomes for co-infection of chronic hepatitis C with and without cirrhosis and COVID-19: A multicenter retrospective study. World J Gastroenterol 27(42): 7362-7375. 10.3748/wjg.v27.i42.7362
[699] Afşin A, Tibilli H, Hoşoğlu Y, et al. (2021) Fibrinogen-to-albumin ratio predicts mortality in COVID-19 patients admitted to the intensive care unit. Adv Respir Med. 10.5603/ARM.a2021.0098
[700] Agca M, Tuncay E, Yıldırım E, et al. (2021) Is Obesity a Potential Risk factor for Poor Prognosis of COVID-19? Infect Chemother 53(2): 319-331. 10.3947/ic.2021.0026
[701] Aggarwal R, Agrawal A, Gurtoo A, Suman V, Meena S, Prakash A (2021) High Mortality Unaffected by Age, Gender and Steroid Use is the Hallmark of COVID-19 in Diabetes: Observations from a Retrospective Analysis during Peak of 2020 Pandemic in India. J Assoc Physicians India 69(5): 25-29

[702] Aggarwal R, Bhatia R, Kulshrestha K, et al. (2021) Clinicoepidemiological Features and Mortality Analysis of Deceased Patients with COVID-19 in a Tertiary Care Center. Indian J Crit Care Med 25(6): 622-628. 10.5005/jp-journals-10071-23848
[703] Agrupis KA, Smith C, Suzuki S, et al. (2021) Epidemiological and clinical characteristics of the first 500 confirmed COVID-19 inpatients in a tertiary infectious disease referral hospital in Manila, Philippines. Trop Med Health 49(1): 48. 10.1186/s41182-021-00340-0
[704] Ahmed W, Al Obaidli AAK, Joseph P, et al. (2021) Outcomes of patients with end stage kidney disease on dialysis with COVID-19 in Abu Dhabi, United Arab Emirates; from PCR to antibody. BMC Nephrol 22(1): 198. 10.1186/s12882-021-02378-y
[705] Ahsan T, Rani B, Siddiqui R, et al. (2021) Clinical Variants, Characteristics, and Outcomes Among COVID-19 Patients: A Case Series Analysis at a Tertiary Care Hospital in Karachi, Pakistan. Cureus 13(4): e14761. 10.7759/cureus.14761
[706] Ak C, Sayar S, Polat ZP, Kilic ET, Ozdil K (2021) Clinical and Laboratory Factors Associated with Severe Disease Course in Turkish Patients with COVID-19 Infection. Iranian Red Crescent Medical Journal 23(2). 10.32592/ircmj.2021.23.2.283
[707] Akerele IO, Oreh AC, Kawu MB, et al. (2021) Clinical presentation and hospitalisation duration of 201 coronavirus disease 2019 patients in Abuja, Nigeria. Afr J Prim Health Care Fam Med 13(1): e1-e8. 10.4102/phcfm.v13i1.2940
[708] Akhavizadegan H, Aghaziarati M, Balalemi MGR, et al. (2021) Relationship Between Comorbidity, Chronic Diseases, ICU Hospitalization, and Death Rate in the Elderly With Coronavirus Infection. Salmand-Iranian Journal of Ageing 16(1): 86-101. 10.32598/sija.16.1.3161.1
[709] Akhtar H, Khalid S, Rahman FU, et al. (2021) Presenting Characteristics, Comorbidities, and Outcomes Among Patients With COVID-19 Hospitalized in Pakistan: Retrospective Observational Study. JMIR Public Health Surveill 7(12): e32203. 10.2196/32203
[710] Akyol DYRENS (2021) The Effect of Body Mass Index on the Mortality of Patients Followed up in the Intensive Care Unit with COVID-19 Diagnosis. Yoğun Bakım Ünitesinde COVID-19 Tanısı ile Takip Edilen Hastalarda Beden Kitle Índeksinin Mortaliteye Etkisi 17(4): 392-399. 10.4274/BMJ.galenos.2021.2021.10-10
[711] Al Azzi Y, Ajaimy M, Liriano-Ward LE, et al. (2020) Clinical outcomes of hospitalized kidney transplant recipients with COVID-19 in a predominantly minority population. Journal of the American Society of Nephrology 31: 277-278
[712] Al Bahrani BJ, Mehdi I, Khamis FA, Al Farsi AM, Fahdi FA, Al Lawati NA (2021) COVID-19 amongst cancer patients: An experience from Oman. J Pak Med Assoc 71(11): 2563-2570. 10.47391/jpma.11915
[713] Al Balushi A, AlShekaili J, Al Kindi M, et al. (2021) Immunological predictors of disease severity in patients with COVID-19. Int J Infect Dis 110: 83-92. 10.1016/j.ijid.2021.06.056
[714] Al Dossary R, Alnimr A, Aljindan R, et al. (2021) Predictors of Illness Severity in COVID-19 Cases in Saudi Arabia. Infect Drug Resist 14: 4097-4105. 10.2147/idr.S333300
[715] Al Harbi M, Al Kaabi N, Al Nuaimi A, et al. (2022) Clinical and laboratory characteristics of patients hospitalised with COVID-19: clinical outcomes in Abu Dhabi, United Arab Emirates. BMC Infect Dis 22(1): 136. 10.1186/s12879-022-07059-1
[716] Al Harthi S, Al Osali M, Al Ismaili R, et al. (2021) Clinical Characteristics of Confirmed Cases of COVID-19 Admitted at Al Nahdha Hospital, Oman: A Cross-Sectional Descriptive Study. Cureus 13(8): e17343. 10.7759/cureus.17343
[717] Al Mutair A, Elhazmi A, Alhumaid S, et al. (2021) Examining the Clinical Prognosis of Critically Ill Patients with COVID-19 Admitted to Intensive Care Units: A Nationwide Saudi Study. Medicina (Kaunas) 57(9). 10.3390/medicina57090878
[718] Alakeel YS, Alharbi EF, Alhaidal HA, et al. (2022) The effects of the antecedent use of inhaled steroid on the clinical course of COVID-19: A retrospective study of asthmatic patients. J Infect Public Health 15(1): 56-64. 10.1016/j.jiph.2021.12.003
[719] Alali AS, Alshehri AO, Assiri A, et al. (2021) Demographics, comorbidities, and outcomes among young and middle-aged COVID-19 patients in Saudi Arabia. Saudi Pharm J 29(8): 833-842. 10.1016/j.jsps.2021.06.005
[720] Alam MM, Khokhar M, Qasim AP, Jaan A, Mehboob NUH, Qasim JA (2020) Association of clinical presentation & comorbidity among covid-19 patients admitted in tertiary care hospital. Medical Forum Monthly 31(11): 39-43
[721] Al-Ansari RY, Alshaer A, Al-Anazi A, et al. (2021) ABO in Correlation to the Requirement of Mechanical Ventilation and Mortality in Critically Ill Patients With COVID-19. J Hematol 10(2): 64-70. 10.14740/jh821
[722] Albanghali M, Alghamdi S, Alzahrani M, et al. (2022) Clinical Characteristics and Treatment Outcomes of Mild to Moderate COVID-19 Patients at Tertiary Care Hospital, Al Baha, Saudi Arabia: A Single Centre Study. J Infect Public Health 15(3): 331-337. 10.1016/j.jiph.2022.02.001

[723] Alghamdi S (2021) Clinical characteristics and treatment outcomes of severe (ICU) COVID-19 patients in Saudi Arabia: A single centre study. Saudi Pharm J 29(10): 1096-1101. 10.1016/j.jsps.2021.08.008
[724] Alhasan KA, Shalaby MA, Temsah MH, et al. (2021) Factors That Influence Mortality in Critically Ill Patients with SARS-CoV-2 Infection: A Multicenter Study in the Kingdom of Saudi Arabia. Healthcare (Basel) 9(12). 10.3390/healthcare9121608
[725] Alhumaid S, Al Mutair A, Al Alawi Z, et al. (2021) Clinical features and prognostic factors of intensive and non-intensive 1014 COVID-19 patients: an experience cohort from Alahsa, Saudi Arabia. Eur J Med Res 26(1): 47. 10.1186/s40001-021-00517-7
[726] Ali Z, Velasquez J, Chen-Joea C, Zaveri Y, Martin C, Shalikar H (2021) The Effects of Co-morbidities on COVID-19 Patients Admitted to the Hospital. Family Medicine and Medical Science Research 10(2). 10.35248/2327-4972.21.10.261
[727] Alkan S, Akça A, Şener A, et al. (2022) Evaluation of the Hospitalized Coronavirus Disease 2019 Patients in First 3 Months of the Pandemic. Turk Thorac J 23(1): 52-57. 10.5152/TurkThoracJ.2022.21118
[728] Almutairi D, Alqahtani R, Alghamdi A, et al. (2021) Tracheotomy Outcomes in 71 COVID-19 Patients: A Multi-Centric Study in Saudi Arabia. Clin Pract 11(4): 947-953. 10.3390/clinpract11040109
[729] Al-Nimer MSM, Merza TA, Mohammed KYMY, Mohammed HA (2021) Blood cells indices are determinants of the covid-19 outcome: A cross-sectional study from kurdistan region-iraq. Electronic Journal of General Medicine 18(5). 10.29333/ejgm/11013
[730] Al-Ozairi E, Brown R, Hamdan Y, et al. (2021) Risk of mortality among inpatients with COVID-19 and type 2 diabetes: National data from Kuwait. Endocrinol Diabetes Metab 4(4): e00287. 10.1002/edm2.287
[731] Alsayer RM, Alsharif HM, Al Baadani AM, Kalam KA (2021) Clinical and epidemiological characteristics of COVID-19 mortality in Saudi Arabia. Saudi Med J 42(10): 1083-1094. 10.15537/smj.2021.42.10.20210396
[732] Álvarez-Arroyo L, Carrera-Hueso FJ, El-Qutob D, et al. (2021) Descriptive study of a cohort of COVID-19 hospitalized patients in Spain. Gac Med Mex 157(1): 76-83. 10.24875/gmm.M21000525
[733] Alvarez-Esteban PC, Del Barrio E, Rueda OM, Rueda C (2021) Predicting COVID-19 progression from diagnosis to recovery or death linking primary care and hospital records in Castilla y Leon (Spain). PLoS One 16(9). 10.1371/journal.pone.0257613
[734] Álvarez-Maldonado P, Hernández-Ríos G, Ambríz-Mondragón JC, et al. (2021) Characteristics and mortality of Mexican patients with COVID-19 and mechanical ventilation. Gac Med Mex 157(1): 97-101. 10.24875/gmm.20000568
[735] Alves T, Guimarães RS, Souza SF, et al. (2021) Influence of nutritional assistance on mortality by COVID-19 in critically ill patients. Clin Nutr ESPEN 44: 469-471. 10.1016/j.clnesp.2021.05.016
[736] Amado-Tineo J, Ayala-Garcia R, Apolaya-Segura M, et al. (2021) Factors associated with mortality in severe SARS-CoV-2 infection in a Peruvian hospital. Revista Del Cuerpo Medico Del Hospital Nacional Almanzor Aguinaga Asenjo 14(3): 280-286. 10.35434/rcmhnaaa.2021.143.1245
[737] Amato JN, Castelo PM, Cirino F, et al. (2021) Assessing Predictive Factors of COVID-19 Outcomes: A Retrospective Cohort Study in the Metropolitan Region of São Paulo (Brazil). Medicina (Kaunas) 57(10). 10.3390/medicina57101068
[738] Ameet D, Reema K, Zafer K, et al. (2021) Epidemiology, clinical presentation and management of COVID-19 associated Mucormycosis: A single center experience from Pune, Western India. medRxiv. 10.1101/2021.09.15.21263622
[739] Amiel ADD, Nicole M, Amani Daoud D, et al. (2021) Pre-infection 25-hydroxyvitamin D3 levels and association with severity of COVID-19 illness. medRxiv. 10.1101/2021.06.04.21258358
[740] Andersen KM, Bates BA, Rashidi ES, et al. (2022) Long-term use of immunosuppressive medicines and in-hospital COVID-19 outcomes: a retrospective cohort study using data from the National COVID Cohort Collaborative. Lancet Rheumatol 4(1): e33-e41. 10.1016/s2665-9913(21)00325-8
[741] Andrade-Castellanos CA, Colunga-Lozano LE (2021) Epidemiologic factors associated with mortality among hospitalized adult patients with COVID-19 in the state of Jalisco, Mexico. Medicina Interna de Mexico 37(3): 366-372. 10.24245/mim.v37i3.5107
[742] Ángeles-Garay U, Velázquez-García JA, Hernández-González C, et al. (2021) [Factors associated to death in patients hospitalized due to COVID-19]. Rev Med Inst Mex Seguro Soc 59(5): 423-430
[743] Anonymous (2020) An epidemiological study of laboratory confirmed COVID-19 cases admitted in Dhaka Medical College Hospital. Journal of Medicine (Bangladesh) 21(2): 69-75. 10.3329/jom.v21i2.50208
[744] Anonymous (2020) Demographic and clinical profile of 190 COVID-19 patients in a tertiary care private hospital of Dhaka, Bangladesh: An observational study. Journal of Medicine (Bangladesh) 21(2): 82-88. 10.3329/jom.v21i2.50210

[745] Antos A, Kwong ML, Balmorez T, Villanueva A, Murakami S (2021) Unusually High Risks of COVID-19 Mortality with Age-Related Comorbidities: An Adjusted Meta-Analysis Method to Improve the Risk Assessment of Mortality Using the Comorbid Mortality Data. Infect Dis Rep 13(3): 700-711. 10.3390/idr13030065
[746] Anudeep A, Somu C, Kumar JS (2020) Clinical profile and outcomes of critically ill covid-19 patients admitted in a tertiary care hospital. Annals of Tropical Medicine and Public Health 23(19). 10.36295/ASRO.2020.232141
[747] Aparisi Á, Catalá P, Amat-Santos IJ, et al. (2021) Chronic use of renin-angiotensin-aldosterone inhibitors in hypertensive COVID-19 patients: Results from a Spanish registry and meta-analysis. Med Clin (Barc). 10.1016/j.medcli.2021.04.005
[748] Aparisi A, Iglesias-Echeverria C, Ybarra-Falcon C, et al. (2021) Low-density lipoprotein cholesterol levels are associated with poor clinical outcomes in COVID-19. Nutrition Metabolism and Cardiovascular Diseases 31(9): 2619-2627. 10.1016/j.numecd.2021.06.016
[749] Archibald P, Thorpe R (2021) Chronic Medical Conditions as Predictors of the Likelihood of PTSD among Black Adults: Preparing for the Aftermath of COVID-19. Health Soc Work 46(4): 268-276. 10.1093/hsw/hlab025
[750] Arikan H, Ozturk S, Tokgoz B, et al. (2021) Characteristics and outcomes of acute kidney injury in hospitalized COVID-19 patients: A multicenter study by the Turkish society of nephrology. PLoS One 16(8): e0256023. 10.1371/journal.pone.0256023
[751] Arroyo-Díaz JA, Julve J, Vlacho B, et al. (2021) Previous Vitamin D Supplementation and Morbidity and Mortality Outcomes in People Hospitalised for COVID19: A Cross-Sectional Study. Front Public Health 9: 758347. 10.3389/fpubh.2021.758347
[752] Arutyunov GP, Tarlovskaya EI, Arutyunov AG, et al. (2021) International register “dynamics analysis of comorbidities in sars-cov-2 survivors” (Aktiv sars-cov-2): Analysis of predictors of short-term adverse outcomes in covid-19. Russian Journal of Cardiology 26(4): 116-131. 10.15829/1560-4071-2021-4470
[753] Ascencio-Montiel IJ, Tomás-López JC, Álvarez-Medina V, et al. (2022) A Multimodal Strategy to Reduce the Risk of Hospitalization/death in Ambulatory Patients with COVID-19. Arch Med Res. 10.1016/j.arcmed.2022.01.002
[754] Ashktorab H, Folake A, Pizuorno A, et al. (2021) COVID-19 among African Americans and Hispanics: Does gastrointestinal symptoms impact the outcome? World J Clin Cases 9(28): 8374-8387. 10.12998/wjcc.v9.i28.8374
[755] Assaad S, Zrounba P, Cropet C, Blay JY (2021) Mortality of patients with solid and haematological cancers presenting with symptoms of COVID-19 with vs without detectable SARS-COV-2: a French nationwide prospective cohort study. Br J Cancer 125(5): 658-671. 10.1038/s41416-021-01452-4
[756] Azarbakhsh H, Jokari K, Moftakhar L, et al. (2021) Epidemiological characteristics of patients with COVID-19 in Southwest of Iran from February 19 to June 20, 2020. Med J Islam Repub Iran 35: 116. 10.47176/mjiri.35.116
[757] Azarkar Z, Salehiniya H, Kazemi T, Abbaszadeh H (2021) Epidemiological, imaging, laboratory, and clinical characteristics and factors related to mortality in patients with COVID-19: a single-center study. Osong Public Health Res Perspect 12(3): 169-176. 10.24171/j.phrp.2021.0012
[758] Aziz F, Aberer F, Bräuer A, et al. (2021) COVID-19 In-Hospital Mortality in People with Diabetes Is Driven by Comorbidities and Age-Propensity Score-Matched Analysis of Austrian National Public Health Institute Data. Viruses 13(12). 10.3390/v13122401
[759] Badedi M, Alnami A, Darraj H, et al. (2021) Clinical characteristics and ABO blood groups in COVID-19 patients, Saudi Arabia. Medicine (Baltimore) 100(30): e26738. 10.1097/md.0000000000026738
[760] Badedi M, Darraj H, Alnami AQ, et al. (2021) Epidemiological and Clinical Characteristics of Deceased COVID-19 Patients. Int J Gen Med 14: 3809-3819. 10.2147/ijgm.S320713
[761] Bağ Soytaş R, Ünal D, Arman P, et al. (2021) Factors affecting mortality in geriatric patients hospitalized with COVID-19. Turk J Med Sci 51(2): 454-463. 10.3906/sag-2008-91
[762] Baguma S, Okot C, Onira N, et al. (2021) Factors associated with mortality among the COVID-19 patients treated at Gulu Regional Referral Hospital: A retrospective study. ResearchSquare. 10.21203/rs.3.rs-1191937/v1
[763] Baguma S, Okot C, Onira N, et al. (2021) Characteristics of the COVID-19 patients treated at Gulu Regional Referral Hospital, Northern Uganda: A cross-sectional study. ResearchSquare. 10.21203/rs.3.rs-1193578/v1
[764] Bai Y, Liang Y, Wang F, et al. (2021) Clinical course and outcomes of COVID-19 patients with a history of cerebrovascular disease: a retrospective study in Wuhan. Ann Transl Med 9(12): 988. 10.21037/atm-21-2237

[765] Bailly L, Fabre R, Courjon J, Carles M, Dellamonica J, Pradier C (2022) Obesity, diabetes, hypertension and severe outcomes among inpatients with coronavirus disease 2019: a nationwide study. Clin Microbiol Infect 28(1): 114-123. 10.1016/j.cmi.2021.09.010
[766] Bairwa M, Kumar R, Ajmal M, Bahurupi Y, Kant R (2021) Predictors of critical illness and mortality based on symptoms and initial physical examination for patients with SARS-CoV-2: A retrospective cohort study. J Infect Public Health 14(8): 1028-1034. 10.1016/j.jiph.2021.06.010
[767] Ballin M, Bergman J, Kivipelto M, Nordström A, Nordström P (2021) Excess Mortality After COVID-19 in Swedish Long-Term Care Facilities. J Am Med Dir Assoc 22(8): 1574-1580.e1578. 10.1016/j.jamda.2021.06.010
[768] Barbera LK, Kamis KF, Abdo MH, et al. (2021) Risk factors for hospitalization in people with HIV and COVID-19. Topics in Antiviral Medicine 29(1): 206-207
[769] Barman Roy D, Gupta V, Tomar S, et al. (2021) Epidemiology and Risk Factors of COVID-19-Related Mortality. Cureus 13(12): e20072. 10.7759/cureus.20072
[770] Barochiner J, Martínez R, Conti PR, Marín MJ, Ko EA, Osorno MS (2021) Use of renin-angiotensin system inhibitors and clinical predictors of COVID-19 severity in a tertiary hospital in the city of Buenos Aires. Infez Med 29(2): 209-215
[771] Basheer M, Saad E, Hagai R, Assy N (2021) Clinical Predictors of Mortality and Critical Illness in Patients with COVID-19 Pneumonia. Metabolites 11(10). 10.3390/metabo11100679
[772] Bastola A, Shrestha S, Nepal R, et al. (2021) Clinical Mortality Review of COVID-19 Patients at Sukraraj Tropical and Infectious Disease Hospital, Nepal; A Retrospective Study. Trop Med Infect Dis 6(3). 10.3390/tropicalmed6030137
[773] Baynouna AlKetbi LM, Nagelkerke N, Abdelbaqi H, et al. (2021) Risk Factors for SARS-CoV-2 Infection Severity in Abu Dhabi. J Epidemiol Glob Health 11(4): 344-353. 10.1007/s44197-021-00006-4
[774] Beatty K, Kavanagh PM (2021) A retrospective cohort study of outcomes in hospitalised COVID-19 patients during the first pandemic wave in Ireland. Ir J Med Sci: 1-11. 10.1007/s11845-021-02753-6
[775] Beiser DG, Jarou ZJ, Kassir AA, et al. (2021) Predicting 30-day return hospital admissions in patients with COVID-19 discharged from the emergency department: A national retrospective cohort study. J Am Coll Emerg Physicians Open 2(6): e12595. 10.1002/emp2.12595
[776] Bellia A, Andreadi A, Giudice L, et al. (2021) Atherogenic Dyslipidemia on Admission Is Associated With Poorer Outcome in People With and Without Diabetes Hospitalized for COVID-19. Diabetes Care 44(9): 2149-2157. 10.2337/dc20-2838
[777] Berrajaa S, Berrichi S, Bouayed Z, et al. (2021) Diabetes as a predictive factor for severe form and high mortality risk of COVID-19: Retrospective cohort study of 188 cases. Ann Med Surg (Lond) 72: 103095. 10.1016/j.amsu.2021.103095
[778] Bertino V, Credendino O, Sorrentino L, et al. (2021) [Acute severe respiratory distress in chronic haemodialytic patients affected by SARS-CoV-2 pneumonia: prevalence and associated factors. A single-centre experience from Cardarelli Hospital in Naples (Italy)]. G Ital Nefrol 38(6)
[779] Bertuzzi AF, Ciccarelli M, Marrari A, et al. (2021) Impact of active cancer on COVID-19 survival: a matched-analysis on 557 consecutive patients at an Academic Hospital in Lombardy, Italy. Br J Cancer 125(3): 358-365. 10.1038/s41416-021-01396-9
[780] Betancourt LATCASRGSARMMGMDAMFELVAZHVHRCC (2021) Características Y Desenlaces Clínicos De Pacientes Con Covid19 En La Primera Ola. Acta Colombiana de Cuidado Intensivo. 10.1016/j.acci.2021.12.002
[781] Bhalla S, Sharma B, Smith D, et al. (2021) Investigating Unhealthy Alcohol Use As an Independent Risk Factor for Increased COVID-19 Disease Severity: Observational Cross-sectional Study. JMIR Public Health Surveill 7(11): e33022. 10.2196/33022
[782] Bhanuprasad K, Manesh A, Devasagayam E, et al. (2021) Risk factors associated with the mucormycosis epidemic during the COVID-19 pandemic. Int J Infect Dis 111: 267-270. 10.1016/j.ijid.2021.08.037
[783] Bhargava A, Sharma M, Akagi E, Szpunar SM, Saravolatz L (2021) Predictors for in-hospital mortality from coronavirus disease 2019 (COVID-19) infection among adults aged 18-65 years. Infect Control Hosp Epidemiol 42(6): 772-775. 10.1017/ice.2020.1390
[784] Bhat A, Mascarenhas DG, Manjunath J, Anand Kumar R, Suresh S, Fernandes G (2021) Clinical and biomarker profile of covid-19 patients admitted in a tertiary care hospital. Biomedicine (India) 41(2): 268-273. 10.51248/.v41i2.794
[785] Bhattacharyya A, Halder S, Mandal T, et al. (2021) Effect of Angiotensin Converting Enzyme Inhibitors/Angiotensin Receptor Blockers on COVID-19 outcome: A Record Based Observational Study in West Bengal. J Assoc Physicians India 69(7): 11-12
[786] Bintoro SUY, Dwijayanti NMI, Pramudya D, et al. (2021) Hematologic and coagulopathy parameter as a survival predictor among moderate to severe COVID-19 patients in non- ICU ward: a

single-center study at the main referral hospital in Surabaya, East Java, Indonesia. F1000Res 10: 791. 10.12688/f1000research.53803.3
[787] Bisigniano L, Rosa-Diez G, Tagliafichi V, et al. (2021) [COVID-19 infection in chronic dialysis patients in Argentina]. Medicina (B Aires) 81(6): 916-921
[788] Bittaye S, Jagne A, Badjan A, et al. (2021) A Retrospective Cross Sectional Study of Clinical Characteristics and Prognostic Factors of Covid 19 Patients Admitted to a Gambian Teaching Hospital. ResearchSquare. 10.21203/rs.3.rs-603762/v1
[789] Bobdey S, Chawla N, Behera V, et al. (2021) An analysis of mortality and survival of COVID 19 patients admitted to a tertiary care hospital in Maharashtra, India. Med J Armed Forces India 77(Suppl 2): S353-s358. 10.1016/j.mjafi.2021.02.004
[790] Boietti BR, Mirofsky M, Valentini R, et al. (2021) [Descriptive analysis of 4776 patients admitted to medical clinic services for COVID-19. Results of the Argentine Multi-Center Registry - REMA-COVID-19]. Medicina (B Aires) 81(5): 703-714
[791] Bonnet G, Weizman O, Trimaille A, et al. (2021) Characteristics and outcomes of patients hospitalized for COVID-19 in France: The Critical COVID-19 France (CCF) study. Arch Cardiovasc Dis 114(5): 352-363. 10.1016/j.acvd.2021.01.003
[792] Bonora E, Fedeli U, Schievano E, et al. (2021) SARS-CoV-2 and COVID-19 in diabetes mellitus. Population-based study on ascertained infections, hospital admissions and mortality in an Italian region with ~5 million inhabitants and ~250,000 diabetic people. Nutr Metab Cardiovasc Dis 31(9): 2612-2618. 10.1016/j.numecd.2021.06.010
[793] Bouziotis J, Arvanitakis M, Preiser JC (2022) Association of body mass index with COVID-19 related in-hospital death. Clin Nutr. 10.1016/j.clnu.2022.01.017
[794] Branco CG, Duarte I, Gameiro J, et al. (2021) Presentation and outcomes of chronic kidney disease patients with COVID-19. J Bras Nefrol. 10.1590/2175-8239-jbn-2021-0071
[795] Brojakowska A, Eskandari A, Bisserier M, et al. (2021) Comorbidities, sequelae, blood biomarkers and their associated clinical outcomes in the Mount Sinai Health System COVID-19 patients. PLoS One 16(7): e0253660. 10.1371/journal.pone.0253660
[796] Brook R, Lim HY, Ho P, Choy KW (2021) Risk factors and early prediction of clinical deterioration and mortality in adult COVID-19 inpatients: an Australian tertiary hospital experience. Intern Med J. 10.1111/imj.15631
[797] Campi I, Gennari L, Merlotti D, et al. (2021) Vitamin D and COVID-19 severity and related mortality: a prospective study in Italy. BMC Infect Dis 21(1): 566. 10.1186/s12879-021-06281-7
[798] Caplin B, Ashby D, McCafferty K, et al. (2021) Risk of COVID-19 Disease, Dialysis Unit Attributes, and Infection Control Strategy among London In-Center Hemodialysis Patients. Clin J Am Soc Nephrol 16(8): 1237-1246. 10.2215/cjn.03180321
[799] Carlos Ignacio Dominguez C, Ricardo Gallardo G, Mariluna Corvalan G, Rodrigo Sanchez M, Gabriel Sandoval S, Jaime Rappoport S (2021) Mortalidad Quirúrgica durante pandemia de coronavirus en un centro universitario. 73(3). 10.35687/S2452-4549%Y00%I1221
[800] Casillas E, Jr., Wu G, Iantorno S, et al. (2021) COVID-19: Highlighting Health Disparities in the Los Angeles Latinx Community. Clin Med Res 19(4): 161-168. 10.3121/cmr.2021.1654
[801] Castilla J, Guevara M, Miqueleiz A, et al. (2021) Risk Factors of Infection, Hospitalization and Death from SARS-CoV-2: A Population-Based Cohort Study. J Clin Med 10(12). 10.3390/jcm10122608
[802] Cavallaro M, Moiz H, Keeling MJ, McCarthy ND (2021) Contrasting factors associated with COVID-19-related ICU admission and death outcomes in hospitalised patients by means of Shapley values. medRxiv. 10.1101/2020.12.03.20242941
[803] Cecelja M, Lewis CM, Shah AM, Chowienczyk P (2021) Cardiovascular health and risk of hospitalization with COVID-19: A Mendelian Randomization study. JRSM Cardiovasc Dis 10: 20480040211059374. 10.1177/20480040211059374
[804] Cereda A, Toselli M, Palmisano A, et al. (2022) Coronary calcium score as a predictor of outcomes in the hypertensive Covid-19 population: results from the Italian (S) Core-Covid-19 Registry. Hypertens Res 45(2): 333-343. 10.1038/s41440-021-00798-9
[805] Chachkhiani D, Isakadze M, Villemarette-Pittman NR, Devier DJ, Lovera JF (2021) Altered mental status predicts length of stay but not death in a community-based cohort of hospitalized COVID-19 patients. Clin Neurol Neurosurg 210: 106977. 10.1016/j.clineuro.2021.106977
[806] Chandarana H, Pisuchpen N, Krieger R, et al. (2021) Association of body composition parameters measured on CT with risk of hospitalization in patients with Covid-19. Eur J Radiol 145: 110031. 10.1016/j.ejrad.2021.110031
[807] Charoenngam N, Ilori TO, Holick MF, Hochberg NS, Apovian CM (2021) Self-identified Race and COVID-19-Associated Acute Kidney Injury and Inflammation: a Retrospective Cohort Study of Hospitalized Inner-City COVID-19 Patients. J Gen Intern Med 36(11): 3487-3496. 10.1007/s11606-021-06931-1

[808] Charvi P, Sonali P, Purwa D, Rupeshkumar D (2021) A study of the clinico-epidemiological profile of COVID-19 patients admitted in a tertiary care hospital in India. Journal of Clinical and Diagnostic Research 15(4)
[809] Chávez-Íñiguez JS, Cano-Cervantes JH, Maggiani-Aguilera P, et al. (2021) Mortality and evolution between community and hospital-acquired COVID-AKI. PLoS One 16(11): e0257619. 10.1371/journal.pone.0257619
[810] Chen C, Zhang Y, Zhao X, Tao M, Yan W, Fu Y (2021) Hypoalbuminemia - An Indicator of the Severity and Prognosis of COVID-19 Patients: A Multicentre Retrospective Analysis. Infect Drug Resist 14: 3699-3710. 10.2147/idr.S327090
[811] Chen L, Chen J, Wu Y, et al. (2021) Clinical Characteristics and Outcomes of Hypertensive Patients Infected with COVID-19: A Retrospective Study. Int J Gen Med 14: 4619-4628. 10.2147/ijgm.S324077
[812] Chen Q, Wang L, Li C, et al. (2021) Chronic Cardio-Metabolic Disease Increases the Risk of Worse Outcomes Among Hospitalized Patients With COVID-19: A Multicenter, Retrospective, and Real-World Study. J Am Heart Assoc 10(12): e018451. 10.1161/jaha.120.018451
[813] Chen X, Zheng L, Ye S, et al. (2021) Research on Influencing Factors and Classification of Patients With Mild and Severe COVID-19 Symptoms. Front Cell Infect Microbiol 11: 670823. 10.3389/fcimb.2021.670823
[814] Chhiba KD, Patel GB, Vu THT, et al. (2020) Prevalence and characterization of asthma in hospitalized and nonhospitalized patients with COVID-19. J Allergy Clin Immunol 146(2): 307-314.e304. 10.1016/j.jaci.2020.06.010
[815] Chin HH, Chin YH, Yap YL, et al. (2021) A retrospective cross-sectional study on the risk factors and survival outcome of End Stage Kidney Disease patients receiving regular maintenance haemodialysis with COVID-19 infection in Hospital Enche' Besar Hajjah Khalsom, Kluang. Med J Malaysia 76(6): 845-852
[816] Cho SM, Premraj L, Fanning J, et al. (2021) Ischemic and Hemorrhagic Stroke Among Critically Ill Patients With Coronavirus Disease 2019: An International Multicenter Coronavirus Disease 2019 Critical Care Consortium Study. Crit Care Med 49(12): e1223-e1233. 10.1097/ccm.0000000000005209
[817] Choi YJ, Park JY, Lee HS, et al. (2021) Variable effects of underlying diseases on the prognosis of patients with COVID-19. PLoS One 16(7): e0254258. 10.1371/journal.pone.0254258
[818] Cini Oliveira M, de Araujo Eleuterio T, de Andrade Corrêa AB, et al. (2021) Fatores associados ao óbito em casos confirmados de COVID-19 no estado do Rio de Janeiro. BMC Infect Dis 21(1): 687. 10.1186/s12879-021-06384-1
[819] Clouston SAP, Luft BJ, Sun E (2021) Clinical risk factors for mortality in an analysis of 1375 patients admitted for COVID treatment. Sci Rep 11(1): 23414. 10.1038/s41598-021-02920-w
[820] Colombo C, Cipolli M, Daccò V, et al. (2021) Clinical course and risk factors for severe COVID-19 among Italian patients with cystic fibrosis: a study within the Italian Cystic Fibrosis Society. Infection: 1-9. 10.1007/s15010-021-01737-z
[821] Conte C, Esposito A, De Lorenzo R, et al. (2021) Epicardial adipose tissue characteristics, obesity and clinical outcomes in COVID-19: A post-hoc analysis of a prospective cohort study. Nutr Metab Cardiovasc Dis 31(7): 2156-2164. 10.1016/j.numecd.2021.04.020
[822] Cordero-Franco HF, De La Garza-Salinas LH, Gomez-Garcia S, Moreno-Cuevas JE, Vargas-Villarreal J, González-Salazar F (2021) Risk Factors for SARS-CoV-2 Infection, Pneumonia, Intubation, and Death in Northeast Mexico. Front Public Health 9: 645739. 10.3389/fpubh.2021.645739
[823] Cordova E, Mykietiuk A, Sued O, et al. (2021) Clinical characteristics and outcomes of hospitalized patients with SARS-CoV-2 infection in a Latin American country: Results from the ECCOVID multicenter prospective study. PLoS One 16(10): e0258260. 10.1371/journal.pone.0258260
[824] Coronado-Vázquez V, Ramírez-Durán MDV, Gómez-Salgado J, et al. (2021) Evolution of a Cohort of COVID-19 Infection Suspects Followed-Up from Primary Health Care. J Pers Med 11(6). 10.3390/jpm11060459
[825] Couderc AL, Correard F, Hamidou Z, et al. (2021) Factors Associated With COVID-19 Hospitalizations and Deaths in French Nursing Homes. J Am Med Dir Assoc 22(8): 1581-1587.e1583. 10.1016/j.jamda.2021.06.023
[826] Cristelli MP, Viana LA, Dantas MTC, et al. (2021) The Full Spectrum of COVID-19 Development and Recovery Among Kidney Transplant Recipients. Transplantation 105(7): 1433-1444. 10.1097/tp.0000000000003751
[827] Cruz EG, Broca Garcia BE, Sandoval DM, et al. (2021) Renal Resistive Index as a Predictor of Acute Kidney Injury and Mortality in COVID-19 Critically Ill Patients. Blood Purif: 1-8. 10.1159/000517469

[828] Cueto-Manzano AM, Espinel-Bermúdez MC, Hernández-González SO, et al. (2021) Risk factors for mortality of adult patients with COVID-19 hospitalised in an emerging country: a cohort study. BMJ Open 11(7): e050321. 10.1136/bmjopen-2021-050321
[829] Da Silva PV, De Oliveira SB, Escalante JJC, et al. (2021) Risk Factors for Death Among 120,804 Hospitalized Patients with Confirmed COVID-19 in Sao Paulo, Brazil. American Journal of Tropical Medicine and Hygiene 105(1): 88-92. 10.4269/ajtmh.20-1598
[830] Dadgari A, Mirrezaei SM, Talebi SS, Gheshlaghi YA, Rasaf MR (2021) Investigating Some Risk Factors Related to the COVID-19 Pandemic in the Middle-aged and Elderly. Salmand-Iranian Journal of Ageing 16(1): 102-111. 10.32598/sija.16.1.3172.1
[831] Dai CL, Kornilov SA, Roper RT, et al. (2021) Characteristics and Factors Associated With Coronavirus Disease 2019 Infection, Hospitalization, and Mortality Across Race and Ethnicity. Clin Infect Dis 73(12): 2193-2204. 10.1093/cid/ciab154
[832] Dai W, Lund H, Chen Y, et al. (2021) Hypertriglyceridemia during hospitalization independently associates with mortality in patients with COVID-19. J Clin Lipidol 15(5): 724-731. 10.1016/j.jacl.2021.08.002
[833] Darmadi DPCRRHRA Inflammatory laboratory findings associated with severe illness among hospitalized individuals with COVID-19 in Medan, Indonesia: a cross-sectional study (preprint). In:
[834] Das P, Akhtar Z, Mah EMS, et al. (2021) Establishing a sentinel surveillance system for the novel COVID-19 in a resource-limited country: methods, system attributes and early findings. BMJ Open 11(12): e055169. 10.1136/bmjopen-2021-055169
[835] David AZ, Brigid MW, Sadeer A-K, et al. (2021) Pre-Existing and Pragmatically-Derived Immunohematologic Phenotypes Predict All-Cause and COVID-19-Specific Mortality: A 'Population Immunology' Approach. SSRN
[836] de Abajo FJ, Rodríguez-Miguel A, Rodríguez-Martín S, Lerma V, García-Lledó A (2021) Impact of in-hospital discontinuation with angiotensin receptor blockers or converting enzyme inhibitors on mortality of COVID-19 patients: a retrospective cohort study. BMC Med 19(1): 118. 10.1186/s12916-021-01992-9
[837] de Havenon A, Ney JP, Callaghan B, et al. (2021) Characteristics and Outcomes Among US Patients Hospitalized for Ischemic Stroke Before vs During the COVID-19 Pandemic. JAMA Netw Open 4(5): e2110314. 10.1001/jamanetworkopen.2021.10314
[838] De Rosa FG, Palazzo A, Rosso T, et al. (2021) Risk Factors for Mortality in COVID-19 Hospitalized Patients in Piedmont, Italy: Results from the Multicenter, Regional, CORACLE Registry. J Clin Med 10(9). 10.3390/jcm10091951
[839] Della-Morte D, Pacifici F, Ricordi C, et al. (2021) Low level of plasminogen increases risk for mortality in COVID-19 patients. Cell Death Dis 12(8): 773. 10.1038/s41419-021-04070-3
[840] Denson JL, Gillet AS, Zu Y, et al. (2021) Metabolic Syndrome and Acute Respiratory Distress Syndrome in Hospitalized Patients With COVID-19. JAMA Netw Open 4(12): e2140568. 10.1001/jamanetworkopen.2021.40568
[841] Di Filippo L, De Lorenzo R, Sciorati C, et al. (2021) Adiponectin to leptin ratio reflects inflammatory burden and survival in COVID-19. Diabetes Metab 47(6): 101268. 10.1016/j.diabet.2021.101268
[842] Díaz-Lazo A, Otivo RM, Nuñez EL, Lopez EA (2021) Clinical and epidemiological characteristics of patients with COVID-19 at a high-altitude hospital. Horizonte Medico 21(2). 10.24265/HORIZMED.2021.V21N2.09
[843] Dinesh A, Mallick T, Arreglado TM, Altonen BL, Engdahl R (2021) Outcomes of COVID-19 Admissions in the New York City Public Health System and Variations by Hospitals and Boroughs During the Initial Pandemic Response. Front Public Health 9: 570147. 10.3389/fpubh.2021.570147
[844] Dinh A, Mercier JC, Jaulmes L, et al. (2021) Safe Discharge Home With Telemedicine of Patients Requiring Nasal Oxygen Therapy After COVID-19. Front Med (Lausanne) 8: 703017. 10.3389/fmed.2021.703017
[845] Dmytriw AA, Dibas M, Phan K, et al. (2022) Acute ischaemic stroke associated with SARS-CoV-2 infection in North America. J Neurol Neurosurg Psychiatry. 10.1136/jnnp-2021-328354
[846] Dmytriw AA, Phan K, Schirmer C, et al. (2021) Ischemic stroke associated with COVID-19 and racial outcome disparity in North America. Stroke 52(SUPPL 1). 10.1161/str.52.suppl-1.P111
[847] Dominguez G, Garrido C, Cornejo M, Danke K, Acuna M (2021) Comorbidities and demographic factors associated with severe COVID-19 outcomes in a Chilean hospital. Revista Medica De Chile 149(8): 1141-1149
[848] Dos Santos GA, Pimenta R, Viana NI, et al. (2021) Shorter leukocyte telomere length is associated with severity of COVID-19 infection. Biochem Biophys Rep 27: 101056. 10.1016/j.bbrep.2021.101056
[849] Drapkina OM, Karpov OE, Lukyanov MM, et al. (2020) PROSPECTIVE IN-HOSPITAL REGISTRY OF PATIENTS WITH SUSPECTED OR DOCUMENTED COVID-19 INFECTION AND COMMUNITYACQUIRED PNEUMONIA (TARGET-VIP): CHARACTERISTICS OF PATIENTS AND

ASSESSMENT OF IN-HOSPITAL OUTCOMES. Cardiovascular Therapy and Prevention 19(6): 79-88. 10.15829/1728-8800-2020-2727
[850] Dres M, Hajage D, Lebbah S, et al. (2021) Characteristics, management, and prognosis of elderly patients with COVID-19 admitted in the ICU during the first wave: insights from the COVID-ICU study : Prognosis of COVID-19 elderly critically ill patients in the ICU. Ann Intensive Care 11(1): 77. 10.1186/s13613-021-00861-1
[851] Dudar I, Krasyuk E, Shymova A, et al. (2021) COVID-19 in patients with chronic kidney disease stage 5. Ukrainian Journal of Nephrology and Dialysis(2): 38-47. 10.31450/ukrjnd.2(70).2021.05
[852] Dutta A, Jevalikar G, Sharma R, et al. (2021) Low FT3 is an independent marker of disease severity in patients hospitalized for COVID-19. Endocr Connect 10(11): 1455-1462. 10.1530/ec-21-0362
[853] Eid RA, Attia AM, Hassan M, Shaker MA, Kamal MA (2021) Demographic, clinical, and laboratory characteristics of patients with COVID-19 during the second and third waves of the pandemic in Egypt. J Infect Public Health 14(10): 1358-1366. 10.1016/j.jiph.2021.08.009
[854] El-Malky AM, Albalawi YA, Albalawi RK, et al. (2021) Independently associated variables with higher morbidity and mortality: A scientific perspective of a cohort of orthopedic patients with Covid-19. Medical Science 25(110): 930-945
[855] Elyana Sri S, Septi Sewi M, Verarica S (2021) Risk Factors of Covid-19 Confirmed Died Patients in Dr. Kariadi Hospital: A Retrospective Study. Indonesian Journal of Tropical and Infectious Disease 9(1). 10.20473/IJTID.V9I1.22609
[856] Fang X, Cai J, Liang Q, Li X, Bian Y (2020) Clinical features and chest CT findings in moderate and severe COVID-19 patients: an analysis of 506 cases from Wuhan Huoshenshan Hospital. Chinese Journal of Clinical Infectious Diseases 13(3)
[857] Farooque I, Farooque U, Karimi S, et al. (2021) Clinical Presentations and Outcomes of Coronavirus Disease 2019 in Patients With Solid Tumors. Cureus 13(6): e15452. 10.7759/cureus.15452
[858] Farooqui MA, Almegren A, Binrushud SR, et al. (2021) Incidence and Outcome of Acute Kidney Injury in Patients Hospitalized With Coronavirus Disease-19 at a Tertiary Care Medical Center in Saudi Arabia. Cureus 13(10): e18927. 10.7759/cureus.18927
[859] Fatimah SA, Reem SA, Edward BD, et al. (2021) Epidemiology and Clinical Characteristics in Individuals with Confirmed SARS-CoV-2 Infection During the Early COVID-19 Pandemic in Saudi Arabia. medRxiv. 10.1101/2021.07.13.21260428
[860] Fernández-Cruz A, Ruiz-Antorán B, Muñoz-Gómez A, et al. (2020) A Retrospective Controlled Cohort Study of the Impact of Glucocorticoid Treatment in SARS-CoV-2 Infection Mortality. Antimicrob Agents Chemother 64(9). 10.1128/aac.01168-20
[861] Ferre A, Marquion F, Delord M, et al. (2021) Association of Ventilator Type with Hospital Mortality in Critically Ill Patients with SARS-CoV2 Infection: A Retrospective Study. ResearchSquare. 10.21203/rs.3.rs-565958/v1
[862] Fervers P, Kottlors J, Große Hokamp N, et al. (2021) Coronary artery calcification on low-dose chest CT is an early predictor of severe progression of COVID-19-A multi-center, multi-vendor study. PLoS One 16(7): e0255045. 10.1371/journal.pone.0255045
[863] Fitzgerald KC, Mecoli CA, Douglas M, et al. (2022) Risk Factors for Infection and Health Impacts of the Coronavirus Disease 2019 (COVID-19) Pandemic in People With Autoimmune Diseases. Clin Infect Dis 74(3): 427-436. 10.1093/cid/ciab407
[864] Foo O, Hiu S, Teare D, Syed AA, Razvi S (2021) A global country-level analysis of the relationship between obesity and COVID-19 cases and mortality. Diabetes Obes Metab 23(12): 2697-2706. 10.1111/dom.14523
[865] Fouda Mbarga N, Epee E, Mbarga M, et al. (2021) Clinical profile and factors associated with COVID-19 in Yaounde, Cameroon: A prospective cohort study. PLoS One 16(5): e0251504. 10.1371/journal.pone.0251504
[866] Fried MW, Crawford JM, Mospan AR, et al. (2021) Patient Characteristics and Outcomes of 11 721 Patients With Coronavirus Disease 2019 (COVID-19) Hospitalized Across the United States. Clin Infect Dis 72(10): e558-e565. 10.1093/cid/ciaa1268
[867] Fu C, Stoeckle JH, Masri L, et al. (2021) COVID-19 outcomes in hospitalized patients with active cancer: Experiences from a major New York City health care system. Cancer 127(18): 3466-3475. 10.1002/cncr.33657
[868] Fujita K, Kashihara E, Kanai O, et al. (2021) Increasing Burden of Nursing Care on the Treatment of COVID-19 Patients in the Aging Society: Analyses During the First to the Third Wave of Pandemic in Kyoto City, Japan. Front Med (Lausanne) 8: 767110. 10.3389/fmed.2021.767110
[869] Galindo JL, Lutz JR, Izquierdo MA, Parra K, Prieto LM, Carrillo JA (2021) Characteristics and Clinical Course of Adult in Patients with SARS-CoV-2 Pneumonia at High Altitude. Can Respir J 2021: 5590879. 10.1155/2021/5590879

[870] Gandhi Periyasamy A, Kathirvel S, Rehman T, Singh A (2021) Demographic and clinical factors associated with early hospital Coronovirus disease 2019 deaths in a low middle income setting: A record-based analysis of 20,641 deaths from India. Monaldi Arch Chest Dis. 10.4081/monaldi.2021.1890
[871] García S, Cuatepotzo-Burgos FM, Toledo-Lozano CG, et al. (2021) Neurological Manifestations and Outcomes in a Retrospective Cohort of Mexican Inpatients with SARS-CoV-2 Pneumonia: Design of a Risk Profile. Healthcare (Basel) 9(11). 10.3390/healthcare9111501
[872] García Yanez J, Ayala Diaz N, Morales Torres RM, et al. (2021) Epidemiology and risk factors for intensive care and mortality in COVID-19 hospitalized patients. Rev esp med prev salud pública 26(1): 17-24
[873] García-Alvarado FJ, Muñoz-Hernández MA, Moran Guel E, González-Martínez MDR, Macías Corral MA, Delgado-Aguirre HA (2021) Risk Factors and Clinical Phenotypes Associated with Severity in Patients with COVID-19 in Northeast Mexico. Vector Borne Zoonotic Dis 21(9): 720-726. 10.1089/vbz.2021.0016
[874] García-Lamberechts EJ, Miró Ò, Fragiel M, et al. (2021) A case-control analysis of stroke in COVID-19 patients: Results of unusual manifestations of COVID-19-study 11. Acad Emerg Med 28(11): 1236-1250. 10.1111/acem.14389
[875] Garlapati PR, Kumar S, Patel M, et al. (2022) Clinical Characteristics and Predictors of Mortality in Obese African-Americans with COVID-19: a Single-Center Retrospective Study. J Racial Ethn Health Disparities: 1-8. 10.1007/s40615-021-01206-1
[876] Gault N, Esposito-Farèse M, Revest M, et al. (2021) Chronic use of renin-angiotensin-aldosterone system blockers and mortality in COVID-19: A multicenter prospective cohort and literature review. Fundam Clin Pharmacol 35(6): 1141-1158. 10.1111/fcp.12683
[877] Ge E, Li Y, Wu S, Candido E, Wei X (2021) Association of pre-existing comorbidities with mortality and disease severity among 167,500 individuals with COVID-19 in Canada: A population-based cohort study. PLoS One 16(10): e0258154. 10.1371/journal.pone.0258154
[878] Gervasi F, Andreano A, Russo AG (2021) Metabolic syndrome and risk of COVID-19-related hospitalization: a large, population-based cohort study carried out during the first European outbreak of SARS-CoV-2 infection in the Metropolitan area of Milan (Lombardy Region, Northern Italy). Epidemiol Prev 45(6): 477-485. 10.19191/ep21.6.115
[879] Ghadamgahi F, Tapak L, Bashirian S, Amiri R, Roshanaei G (2021) The effect of underlying diabetes disease on clinical outcome and survival in patients with Covid-19: a propensity score matching study. J Diabetes Metab Disord 20(2): 1-9. 10.1007/s40200-021-00922-z
[880] Ghanei M, Keyvani H, Haghdoost A, et al. (2021) The risk factors and related hospitalizations for cases with positive and negative COVID-19 tests: A case-control study. Int Immunopharmacol 98: 107894. 10.1016/j.intimp.2021.107894
[881] Ghazzay H, Al Mutori HS, Al- Rudaini MS, Reesi HA (2021) Clinical Characteristics and Outcome of SARS-CoV-2 Patients. An Experience from Anbar province - West of Iraq. Revista Bionatura 6(4): 2265-2269. 10.21931/RB/2021.06.04.20
[882] Ghoraba MA, Albadi MA, Ibrahim AA, et al. (2021) Clinical and demographic characteristics of patients with coronavirus disease 2019 in Security Forces Hospital, Riyadh, Saudi Arabia. J Family Med Prim Care 10(2): 947-952. 10.4103/jfmpc.jfmpc_1484_20
[883] Ghouth ASB, Al-Waleedi AA, Fahriani M, Nainu F, Harapan H (2021) Early Determination of Case Fatality Rate of Covid-19 Pandemic During the Ongoing Yemeni Armed Conflict. Disaster Med Public Health Prep: 1-5. 10.1017/dmp.2021.250
[884] Giannis D, Allen SL, Tsang J, et al. (2021) Postdischarge thromboembolic outcomes and mortality of hospitalized patients with COVID-19: the CORE-19 registry. Blood 137(20): 2838-2847. 10.1182/blood.2020010529
[885] Gimeno-Miguel A, Bliek-Bueno K, Poblador-Plou B, et al. (2021) Chronic diseases associated with increased likelihood of hospitalization and mortality in 68,913 COVID-19 confirmed cases in Spain: A population-based cohort study. PLoS One 16(11): e0259822. 10.1371/journal.pone.0259822
[886] Giusti S, Chazin S, Vaitla P, et al. (2021) Observational Study of the Clinical Characteristics and Short-Term Outcomes of Kidney Transplant Recipients Diagnosed With COVID-19 Infection (SARS-CoV-2) Requiring Hospitalization in New Orleans. Ochsner J 21(4): 329-334. 10.31486/toj.21.0008
[887] Glybochko P, Fomin V, Moiseev S, et al. (2020) Clinical outcomes of patients with COVID-19 admitted for respiratory support to the intensive care units in Russia. Klinicheskaya farmakologiya i terapiya 29(3): 25-36
[888] Goehler A, Hsu TH, Seiglie JA, et al. (2021) Visceral Adiposity and Severe COVID-19 Disease: Application of an Artificial Intelligence Algorithm to Improve Clinical Risk Prediction. Open Forum Infect Dis 8(7): ofab275. 10.1093/ofid/ofab275
[889] GÖKÇE AÖAS, Iacute, Ler VG, Uuml, NdoĞDu ME (2021) BÍNGÖL ÍLÍNDEKÍ 65 YAŞ ÜSTÜ COVID-19 HASTALARININ

EPÍDEMÍYOLOJÍK ÖZELLÍKLERÍNÍN ÍNCELENMES&Iacute. Eskişehir Türk Dünyası Uygulama ve Araştırma Merkezi Halk Sağlığı Dergisi 6(3): 264-270. 10.35232/estudamhsd.902272
[890] Goodman KE, Magder LS, Baghdadi JD, et al. (2021) Impact of Sex and Metabolic Comorbidities on Coronavirus Disease 2019 (COVID-19) Mortality Risk Across Age Groups: 66 646 Inpatients Across 613 U.S. Hospitals. Clin Infect Dis 73(11): e4113-e4123. 10.1093/cid/ciaa1787
[891] Gopalan N, Senthil S, Prabakar NL, et al. (2022) Predictors of mortality among hospitalized COVID-19 patients and risk score formulation for prioritizing tertiary care-An experience from South India. PLoS One 17(2): e0263471. 10.1371/journal.pone.0263471
[892] Goto N, Wada Y, Ikuyama Y, et al. (2021) The usefulness of a combination of age, body mass index, and blood urea nitrogen as prognostic factors in predicting oxygen requirements in patients with coronavirus disease 2019. J Infect Chemother 27(12): 1706-1712. 10.1016/j.jiac.2021.08.009
[893] Goyal N, Sodani AK, Jain R, Ram H (2021) Do Elevated Levels of Inflammatory Biomarkers Predict the Risk of Occurrence of Ischemic Stroke in SARS-CoV2 ?: An Observational Study. J Stroke Cerebrovasc Dis 30(11): 106063. 10.1016/j.jstrokecerebrovasdis.2021.106063
[894] Gray WK, Navaratnam AV, Day J, Wendon J, Briggs TWR (2021) Changes in COVID-19 in-hospital mortality in hospitalised adults in England over the first seven months of the pandemic: An observational study using administrative data. Lancet Reg Health Eur 5: 100104. 10.1016/j.lanepe.2021.100104
[895] Guarin G, Lo KB, Bhargav R, et al. (2021) Factors associated with hospital readmissions among patients with COVID-19: A single-center experience. J Med Virol 93(9): 5582-5587. 10.1002/jmv.27104
[896] Gujski M, Jankowski M, Rabczenko D, Goryński P, Juszczyk G (2022) The Prevalence of Acute Respiratory Distress Syndrome (ARDS) and Outcomes in Hospitalized Patients with COVID-19-A Study Based on Data from the Polish National Hospital Register. Viruses 14(1). 10.3390/v14010076
[897] Gunadi, Hakim MS, Wibawa H, et al. (2021) Is the Infection of the SARS-CoV-2 Delta Variant Associated With the Outcomes of COVID-19 Patients? Front Med (Lausanne) 8: 780611. 10.3389/fmed.2021.780611
[898] Gunadi, Hakim MS, Wibawa H, et al. (2021) Association between prognostic factors and the outcomes of patients infected with SARS-CoV-2 harboring multiple spike protein mutations. Sci Rep 11(1): 21352. 10.1038/s41598-021-00459-4
[899] Gunal O, Sezer O, Ustun GU, et al. (2021) Angiotensin-converting enzyme-1 gene insertion/deletion polymorphism may be associated with COVID-19 clinical severity: a prospective cohort study. Ann Saudi Med 41(3): 141-146. 10.5144/0256-4947.2021.141
[900] Gupta A, Katoch N, Gulati R, Kumar V, Singh S, Garg K (2021) COVID-19 mortality and its risk factors: A single-center observational study. Journal of Acute Disease 10(6): 252-257. 10.4103/2221-6189.330745
[901] Gutiérrez-Abejón E, Herrera-Gómez F, Martín-García D, Tamayo E, Álvarez FJ (2021) A Population-Based Registry Analysis on Hospitalized COVID-19 Patients with Previous Cardiovascular Disease: Clinical Profile, Treatment, and Predictors of Death. J Cardiovasc Dev Dis 8(12). 10.3390/jcdd8120167
[902] Haase N, Plovsing R, Christensen S, et al. (2021) Characteristics, interventions and longer-term outcomes of COVID-19 ICU patients in Denmark - a nationwide, observational study. Acta anaesthesiologica Scandinavica 65(1): 68-75. 10.1111/aas.13701
[903] Hadi YB, Naqvi SFZ, Kupec JT, Sarwari AR (2020) Characteristics and outcomes of COVID-19 in patients with HIV: a multi-center research network study. AIDS (London, England) 34(13): F3-F8. 10.1097/QAD.0000000000002666
[904] Hadinejad Z, Tabari YS, Sajadi Z, Ghadicolaei HT (2021) Clinical Characteristics and Risk Factors of COVID-19 and Related Deaths in Elderly Patients. Salmand-Iranian Journal of Ageing 16(1): 128-138. 10.32598/sija.16.1.658.2
[905] Hamdan M, Badrasawi M, Zidan S, et al. (2021) Risk factors associated with hospitalization owing to COVID-19: a cross-sectional study in Palestine. J Int Med Res 49(12): 3000605211064405. 10.1177/03000605211064405
[906] Harbuwono DS, Handayani D, Wahyuningsih ES, et al. (2022) Impact of diabetes mellitus on COVID-19 clinical symptoms and mortality: Jakarta's COVID-19 epidemiological registry. Prim Care Diabetes 16(1): 65-68. 10.1016/j.pcd.2021.11.002
[907] Hartmann B, Verket M, Balfanz P, et al. (2021) Glycemic Variability Is Associated With All-Cause Mortality In COVID-19 Patients With ARDSFindings From The COVAS Cohort. ResearchSquare. 10.21203/rs.3.rs-1111538/v1
[908] Hasabo EA, Ayyad FA, Alam Eldeen SAM, et al. (2021) Clinical manifestations, complications, and outcomes of patients with COVID-19 in Sudan: a multicenter observational study. Trop Med Health 49(1): 91. 10.1186/s41182-021-00382-4

[909] Hasani Azad M, Khorrami F, Kazemi Jahromi M, et al. (2021) Clinical and Epidemiological Characteristics of Hospitalized COVID-19 Patients in Hormozgan, Iran: A Retrospective, Multicenter Study. Arch Iran Med 24(5): 434-444. 10.34172/aim.2021.62
[910] Hasani WSR, Ganapathy SS, Lin CZ, et al. (2021) Comorbidities and clinical features related to severe outcomes among COVID-19 cases in Selangor, Malaysia. Western Pac Surveill Response J 12(1): 46-52. 10.5365/wpsar.2020.11.3.007
[911] Heini S, Toni L, Kari A, Ulrike B, Tuija L (2021) Predictors of hospitalisation and death due to SARS-CoV-2 infection in Finland: a population-based register study with implications to vaccinations. medRxiv. 10.1101/2021.07.04.21259954
[912] Henein MY, Bytyçi I, Nicoll R, et al. (2021) Combined Cardiac Risk Factors Predict COVID-19 Related Mortality and the Need for Mechanical Ventilation in Coptic Clergy. J Clin Med 10(10). 10.3390/jcm10102066
[913] Henkens M, Raafs A, Verdonschot J, et al. (2021) Age is the Main Determinant of COVID-19 Related in-Hospital Mortality with Minimal Impact of Pre-Existing Comorbidities: A Retrospective Cohort Study. ResearchSquare. 10.21203/rs.3.rs-955049/v1
[914] Herrera Cartaya CE, Betancourt Cervantes J, Lage Dávila A, et al. (2021) Variables associated with severity in COVID-19 patients. Int j med surg sci (Print) 8(3): 1-15. 10.32457/ijmss.v8i3.1612
[915] Herrmann Y, Starck T, Brindl N, et al. (2021) A description and analysis of covid-19 in a populationrepresentative cohort. Topics in Antiviral Medicine 29(1): 244-245
[916] Hicks JT, Burnett E, Matanock A, et al. Hospitalizations for COVID-19 Among American Indian and Alaska Native Adults (>= 18 Years Old) - New Mexico, March-September 2020. Journal of Racial and Ethnic Health Disparities. 10.1007/s40615-021-01196-0
[917] Hicks JT, Das S, Matanock A, Griego-Fisher A, Sosin D (2021) Characteristics of Persons with Secondary Detection of SARS-CoV-2 ≥90 days After First Detection - New Mexico, 2020. J infect dis. 10.1093/infdis/jiab448
[918] Homauni A, Ghadami L, Abdollahi H, Shabestari AN, Asanjarani B (2021) Study of clinical features of patients with COVID-19 hospitalized in an educational hospital in Iran. J Educ Health Promot 10(1): 202. 10.4103/jehp.jehp_971_20
[919] Hozayen SM, Zychowski D, Benson S, et al. (2021) Outpatient and inpatient anticoagulation therapy and the risk for hospital admission and death among COVID-19 patients. EClinicalMedicine 41: 101139. 10.1016/j.eclinm.2021.101139
[920] Htun YM, Win TT, Aung A, et al. (2021) Initial presenting symptoms, comorbidities and severity of COVID-19 patients during the second wave of epidemic in Myanmar. Trop Med Health 49(1): 62. 10.1186/s41182-021-00353-9
[921] Huang Y, Hu Z, Hu D, et al. (2021) Clinical characteristics, risk factors, and cardiac manifestations of cancer patients with COVID-19. J Appl Physiol (1985) 131(3): 966-976. 10.1152/japplphysiol.00325.2021
[922] Hueda-Zavaleta M, Copaja-Corzo C, Bardales-Silva F, Flores-Palacios R, Barreto-Rocchetti L, Benites-Zapata VA (2021) Factors associated with mortality due to COVID-19 in patients from a public hospital in Tacna, Peru. Rev Peru Med Exp Salud Publica 38(2): 214-223. 10.17843/rpmesp.2021.382.7158
[923] Ilias I, Diamantopoulos A, Botoula E, et al. (2021) Covid-19 and Growth Hormone/Insulin-Like Growth Factor 1: Study in Critically and Non-Critically Ill Patients. Front Endocrinol (Lausanne) 12: 644055. 10.3389/fendo.2021.644055
[924] Inanc IH, Bursa N, Gultepe A, Bayramoğlu M, Sabanoglu C, Inanc FA (2022) Association among CO-RADS score, co-morbid diseases, and short-term prognosis in COVID-19 infection. Eur Rev Med Pharmacol Sci 26(2): 653-663. 10.26355/eurrev_202201_27892
[925] Ioannou GN, Locke E, Green P, et al. (2020) Risk Factors for Hospitalization, Mechanical Ventilation, or Death Among 10 131 US Veterans With SARS-CoV-2 Infection. JAMA Netw Open 3(9): e2022310. 10.1001/jamanetworkopen.2020.22310
[926] Iqbal M, Catherine K, Ang Z, et al. (2021) Identifying risk factors for COVID-19 severity and mortality in the UK Biobank. medRxiv. 10.1101/2021.05.10.21256935
[927] Iroungou BA, Mangouka LG, Bivigou-Mboumba B, et al. (2021) Demographic and Clinical Characteristics Associated With Severity, Clinical Outcomes, and Mortality of COVID-19 Infection in Gabon. JAMA Netw Open 4(9): e2124190. 10.1001/jamanetworkopen.2021.24190
[928] Jassat W, Cohen C, Tempia S, et al. (2021) Risk factors for COVID-19-related in-hospital mortality in a high HIV and tuberculosis prevalence setting in South Africa: a cohort study. Lancet HIV 8(9): e554-e567. 10.1016/s2352-3018(21)00151-x
[929] Jassat W, Cohen C, Tempia S, et al. (2021) A national cohort study of COVID-19 in-hospital mortality in South Africa: the intersection of communicable and non-communicable chronic diseases in a high HIV prevalence setting. medRxiv. 10.1101/2020.12.21.20248409

[930] Javanmard S, Mohammadifard N, Nasirian M, et al. (2021) Noncommunicable disease, clinical course and COVID-19 prognosis: results based on I-CORE Registry. East Mediterr Health J 27(11): 1036-1044. 10.26719/emhj.21.052
[931] Jeon WH, Seon JY, Park SY, Oh IH (2021) Association of Metabolic Syndrome with COVID-19 in the Republic of Korea. Diabetes Metab J. 10.4093/dmj.2021.0105
[932] Jimeno S, Ventura PS, Castellano JM, et al. (2021) Prognostic implications of neutrophil-lymphocyte ratio in COVID-19. Eur J Clin Invest 51(1): e13404. 10.1111/eci.13404
[933] Job R, Qader MA, Torres P, et al. (2021) Renin-angiotensin System Blocker in COVID-19. A Single Center Study. J Cardiovasc Pharmacol. 10.1097/fjc.0000000000001189
[934] Jonker PKC, van der Plas WY, Steinkamp PJ, et al. (2021) Perioperative SARS-CoV-2 infections increase mortality, pulmonary complications, and thromboembolic events: A Dutch, multicenter, matched-cohort clinical study. Surgery 169(2): 264-274. 10.1016/j.surg.2020.09.022
[935] Jorge AA-B, Montserrat AR-A, José CJ-L, et al. (2021) Clinical Characteristics and Risk Factors for Mortality of 4 865 Hospitalized COVID-19 Patients at Alternate Care Site in Mexico City. SSRN
[936] Jung A, Orenti A, Dunlevy F, et al. (2021) Factors for severe outcomes following SARS-CoV-2 infection in people with cystic fibrosis in Europe. ERJ Open Res 7(4). 10.1183/23120541.00411-2021
[937] Jylhävä, Juulia, Wang, et al. (2020) Age, frailty and comorbidity as prognostic factors for short-term outcomes in patients with COVID-19 in geriatric care. Journal of the American Medical Directors Association
[938] Kafan S, Tadbir Vajargah K, Sheikhvatan M, et al. (2021) Predicting Risk Score for Mechanical Ventilation in Hospitalized Adult Patients Suffering from COVID-19. Anesth Pain Med 11(2): e112424. 10.5812/aapm.112424
[939] Kahkoska AR, Abrahamsen TJ, Alexander GC, et al. (2021) Association Between Glucagon-Like Peptide 1 Receptor Agonist and Sodium-Glucose Cotransporter 2 Inhibitor Use and COVID-19 Outcomes. Diabetes Care 44(7): 1564-1572. 10.2337/dc21-0065
[940] Kantroo V, Kanwar MS, Goyal P, et al. (2021) Mortality and Clinical Outcomes among Patients with COVID-19 and Diabetes. Med Sci (Basel) 9(4). 10.3390/medsci9040065
[941] Karvar ŞGH, Görgülü Y, Tezcan, et al. (2021) Clinical classification of SARS-CoV-2 positive patients;evaluation of laboratory and radiological findings. Flora 26(3): 401-409. 10.5578/FLORA.20219708
[942] Kaswa R, Yogeswaran P, Cawe B (2021) Clinical outcomes of hospitalised COVID-19 patients at Mthatha Regional Hospital, Eastern Cape, South Africa: A retrospective study. S Afr Fam Pract (2004) 63(1): e1-e5. 10.4102/safp.v63i1.5253
[943] Katkat F, Kalyoncuoğlu M, Karahan S, et al. (2021) Does coexistence of fragmented QRS and cardiovascular disease have the ability to predict the mortality in hospitalized, critically ill patients with COVID-19? Anatol J Cardiol 25(11): 803-810. 10.5152/AnatolJCardiol.2021.13611
[944] Kaverina E, Persuad T (2021) ASSOCIATION BETWEEN COMORBIDITIES AND DISEASE SEVERITY IN COVID-19 PATIENTS OF AN INFECTIOUS DISEASES HOSPITAL IN RUSSIA. Archiv Euromedica 11(1): 12-14. 10.35630/2199-885x/2021/11/1.1
[945] Khalangot M, Sheichenko N, Gurianov V, et al. (2022) Relationship between hyperglycemia, waist circumference, and the course of COVID-19: Mortality risk assessment. Exp Biol Med (Maywood) 247(3): 200-206. 10.1177/15353702211054452
[946] Khamis F, Al Awaidy S, Shaaibi MA, et al. (2021) Epidemiological Characteristics of Hospitalized Patients with Moderate versus Severe COVID-19 Infection: A Retrospective Cohort Single Centre Study. Diseases 10(1). 10.3390/diseases10010001
[947] Kim C, Stierman B, Hales CM, Ogden CL (2021) Race and Hispanic-origin disparities in underlying medical conditions associated with severe COVID-19 illness: U.S. adults, 2015-2018. National Health Statistics Report 154(5). 10.15620/cdc:104188
[948] Kim E, Kim YC, Park JY, Jung J, Lee JP, Kim H (2021) Evaluation of the Prognosis of COVID-19 Patients According to the Presence of Underlying Diseases and Drug Treatment. Int J Environ Res Public Health 18(10). 10.3390/ijerph18105342
[949] Kingery JR, Bf Martin P, Baer BR, et al. (2021) Thirty-Day Post-Discharge Outcomes Following COVID-19 Infection. J Gen Intern Med 36(8): 2378-2385. 10.1007/s11606-021-06924-0
[950] Kompaniyets L, Pennington AF, Goodman AB, et al. (2021) Underlying Medical Conditions and Severe Illness Among 540,667 Adults Hospitalized With COVID-19, March 2020-March 2021. Prev Chronic Dis 18: E66. 10.5888/pcd18.210123
[951] Kong KA, Jung S, Yu M, Park J, Kang IS (2021) Association Between Cardiovascular Risk Factors and the Severity of Coronavirus Disease 2019: Nationwide Epidemiological Study in Korea. Front Cardiovasc Med 8: 732518. 10.3389/fcvm.2021.732518
[952] Kumar H, Dixit S, Gupta N, et al. (2021) Impact of Co-morbidities on outcome of COVID-19 patients: An observational study among patients admitted to intensive care unit. Journal of Clinical and Diagnostic Research 15(7): 48-51. 10.7860/JCDR/2021/48792.15163

[953] Kuno T, So M, Iwagami M, Takahashi M, Egorova NN (2021) The association of statins use with survival of patients with COVID-19. J Cardiol 79(4): 494-500. 10.1016/j.jjcc.2021.12.012
[954] Kuno T, Takahashi M, So M, Egorova NN (2021) The association of anticoagulation before admission and survival of patients with COVID-19. J Cardiol 79(4): 489-493. 10.1016/j.jjcc.2021.12.007
[955] Lami F, Elfadul M, Rashak HA, et al. (2021) Risk Factors of COVID-19 Critical Outcomes in the Eastern Mediterranean Region: A Multicountry Retrospective Study. JMIR Public Health Surveill. 10.2196/32831
[956] Landolfo M, Maino A, Di Salvo E, Fiorini G, Peterlana D, Borghi C (2022) Renin-angiotensin system modulation and outcomes in patients hospitalized for interstitial SARS-CoV2 pneumonia: a cohort study. Intern Emerg Med: 1-7. 10.1007/s11739-022-02929-7
[957] Laxminarayan R, B CM, G VT, Arjun Kumar KV, Wahl B, Lewnard JA (2021) SARS-CoV-2 infection and mortality during the first epidemic wave in Madurai, south India: a prospective, active surveillance study. Lancet Infect Dis 21(12): 1665-1676. 10.1016/s1473-3099(21)00393-5
[958] Lazcano U, Cuadrado-Godia E, Grau M, et al. (2021) Increased COVID-19 Mortality in People With Previous Cerebrovascular Disease: A Population-Based Cohort Study. Stroke: Strokeaha121036257. 10.1161/strokeaha.121.036257
[959] Lee JH, Hwang YM, Cho Y, Oh IY (2021) Prognostic impact of atrial fibrillation in patients with severe acute respiratory syndrome coronavirus 2 infection. Medicine (Baltimore) 100(33): e26993. 10.1097/md.0000000000026993
[960] León Álvarez JL, Calderón Martínez M, Gutiérrez Rojas AR (2021) Analysis of mortality and comorbidity due to covid-19 in Cuba. Rev cuba med 60(2): e2117-e2117
[961] Leoni MLG, Lombardelli L, Colombi D, et al. (2021) Prediction of 28-day mortality in critically ill patients with COVID-19: Development and internal validation of a clinical prediction model. PLoS One 16(7): e0254550. 10.1371/journal.pone.0254550
[962] Leulseged TW, Abebe KG, Hassen IS, et al. (2022) COVID-19 disease severity and associated factors among Ethiopian patients: A study of the millennium COVID-19 care center. PLoS One 17(1): e0262896. 10.1371/journal.pone.0262896
[963] Leulseged TW, Alemahu DG, Hassen IS, et al. (2021) Factors associated with development of symptomatic disease in Ethiopian COVID-19 patients: a case-control study. BMC Infect Dis 21(1): 759. 10.1186/s12879-021-06465-1
[964] Leulseged TW, Hassen IS, Maru EH, et al. (2021) Characteristics and outcome profile of hospitalized African patients with COVID-19: The Ethiopian context. PLoS One 16(11): e0259454. 10.1371/journal.pone.0259454
[965] Li H, Yan B, Gao R, et al. (2021) Abnormal Glucose Metabolism Leads To Severe COVID-19 Due To Hyperinflammation: A Cross-Sectional Study. ResearchSquare. 10.21203/rs.3.rs-1178181/v1
[966] Li M, He Z, Yang J, et al. (2021) Clinical Characteristics, Outcomes, and Risk Factors of Disease Severity in Patients With COVID-19 and With a History of Cerebrovascular Disease in Wuhan, China: A Retrospective Study. Front Neurol 12: 706478. 10.3389/fneur.2021.706478
[967] Li P, Zheng X, Ulsa MC, et al. (2021) Poor sleep behavior burden and risk of COVID-19 mortality and hospitalization. Sleep 44(8). 10.1093/sleep/zsab138
[968] Li Y, Li X, Zhou Y, et al. (2021) A More Important Role of Diabetes Mellitus in Mortality in Elderly Critical COVID-19 Patients: a Single-center Retrospective Study. ResearchSquare. 10.21203/rs.3.rs-1183640/v1
[969] Linneberg A, Kampmann FB, Israelsen SB, et al. (2021) The Association of Low Vitamin K Status with Mortality in a Cohort of 138 Hospitalized Patients with COVID-19. Nutrients 13(6). 10.3390/nu13061985
[970] Lip GYH, Genaidy A, Tran G, Marroquin P, Estes C, Sloop S (2022) Effects of multimorbidity on incident COVID-19 events and its interplay with COVID-19 event status on subsequent incident myocardial infarction (MI). Eur J Clin Invest: e13760. 10.1111/eci.13760
[971] Liu B, Spokes P, He W, Kaldor J (2021) High risk groups for severe COVID-19 in a whole of population cohort in Australia. BMC Infect Dis 21(1): 685. 10.1186/s12879-021-06378-z
[972] Liu D, Zheng Y, Kang J, et al. (2021) Not Only High Number and Specific Comorbidities but Also Age Are Closely Related to Progression and Poor Prognosis in Patients With COVID-19. Front Med (Lausanne) 8: 736109. 10.3389/fmed.2021.736109
[973] Liu D, Zheng Y, Kang J, et al. (2021) High number and specific comorbidities independently of age are closely related to progression and poor prognosis in patients with COVID-19. ResearchSquare. 10.21203/rs.3.rs-576949/v2
[974] Liu J, Zhang JF, Ma HN, et al. (2021) Clinical Characteristics and Factors Associated with Disease Progression of Mild to Moderate COVID-19 Patients in a Makeshift (Fangcang) Hospital: A Retrospective Cohort Study. Ther Clin Risk Manag 17: 841-850. 10.2147/tcrm.S314734

[975] Liu JJ, Sloan ME, Owings AH, et al. (2021) Increased ACE2 Levels and Mortality Risk of Patients With COVID-19 on Proton Pump Inhibitor Therapy. Am J Gastroenterol 116(8): 1638-1645. 10.14309/ajg.0000000000001311
[976] López-Bueno R, Torres-Castro R, Koyanagi A, Smith L, Soysal P, Calatayud J (2021) Associations between recently diagnosed conditions and hospitalization due to COVID-19 in patients aged 50 years and older- A SHARE-based analysis. J Gerontol A Biol Sci Med Sci. 10.1093/gerona/glab199
[977] Lopez-Castaneda S, Garcia-Larragoiti N, Cano-Mendez A, et al. (2021) Inflammatory and Prothrombotic Biomarkers Associated With the Severity of COVID-19 Infection. Clinical and Applied Thrombosis-Hemostasis 27. 10.1177/1076029621999099
[978] Lugon JR, Neves P, Pio-Abreu A, do Nascimento MM, Sesso R (2022) Evaluation of central venous catheter and other risk factors for mortality in chronic hemodialysis patients with COVID-19 in Brazil. Int Urol Nephrol 54(1): 193-199. 10.1007/s11255-021-02920-9
[979] Ma W, Nguyen LH, Yue Y, et al. (2021) Associations between predicted vitamin D status, vitamin D intake, and risk of SARS-CoV-2 infection and Coronavirus Disease 2019 severity. Am J Clin Nutr. 10.1093/ajcn/nqab389
[980] Maddani SS, Gupta N, Umakanth S, Joylin S, Saravu K (2021) Neutrophil-Lymphocyte Ratio in Patients with COVID-19 as a Simple Tool to Predict Requirement of Admission to a Critical Care Unit. Indian J Crit Care Med 25(5): 535-539. 10.5005/jp-journals-10071-23801
[981] Maestre-Muñiz MM, Arias Á, Mata-Vázquez E, et al. (2021) Long-Term Outcomes of Patients with Coronavirus Disease 2019 at One Year after Hospital Discharge. J Clin Med 10(13). 10.3390/jcm10132945
[982] Mahdavi A, Moravej M, Aliramezany M (2022) Electrocardiographic abnormalities and troponin levels provide insight on the prognosis of COVID-19 patients: A single center experience from Iran. Journal of Emergency Practice and Trauma 8(1): 37-42. 10.34172/jept.2021.29
[983] Malek Mahdavi A, Varshochi M, Hajialilo M, Dastgiri S, Khabbazi R, Khabbazi A (2021) Factors associated with COVID-19 and its outcome in patients with rheumatoid arthritis. Clin Rheumatol 40(11): 4527-4531. 10.1007/s10067-021-05830-4
[984] Marimuthu Y, Kunnavil R, Anil NS, et al. (2021) Clinical profile and risk factors for mortality among COVID-19 inpatients at a tertiary care centre in Bengaluru, India. Monaldi Arch Chest Dis 91(3). 10.4081/monaldi.2021.1724
[985] Martínez-Murillo C, Ramos Peñafiel C, Basurto L, et al. (2021) COVID-19 in a country with a very high prevalence of diabetes: The impact of admission hyperglycaemia on mortality. Endocrinol Diabetes Metab 4(3): e00279. 10.1002/edm2.279
[986] Martínez-Urbistondo M, Gutiérrez-Rojas Á, Andrés A, et al. (2021) Severe Lymphopenia as a Predictor of COVID-19 Mortality in Immunosuppressed Patients. J Clin Med 10(16). 10.3390/jcm10163595
[987] Martinot M, Eyriey M, Gravier S, et al. (2021) Predictors of mortality, ICU hospitalization, and extrapulmonary complications in COVID-19 patients. Infect Dis Now 51(6): 518-525. 10.1016/j.idnow.2021.07.002
[988] Mayneris-Perxachs J, Russo MF, Ramos R, et al. (2021) Blood Hemoglobin Substantially Modulates the Impact of Gender, Morbid Obesity, and Hyperglycemia on COVID-19 Death Risk: A Multicenter Study in Italy and Spain. Front Endocrinol (Lausanne) 12: 741248. 10.3389/fendo.2021.741248
[989] Mazaherpour H, Soofian M, Farahani E, et al. (2021) Frequency of Cardiovascular Complications and Its Association with Prognosis of COVID-19 Patients. Biomed Res Int 2021: 7073348. 10.1155/2021/7073348
[990] McCarty TR, Hathorn KE, Redd WD, et al. (2021) How Do Presenting Symptoms and Outcomes Differ by Race/Ethnicity Among Hospitalized Patients With Coronavirus Disease 2019 Infection? Experience in Massachusetts. Clin Infect Dis 73(11): e4131-e4138. 10.1093/cid/ciaa1245
[991] McKay B, Meyers M, Rivard L, Stoltzfus J, Rammohan G, Stankewicz H (2021) Predicting 30 - Day outcomes in emergency department patients discharged with COVID-19. Am J Emerg Med 50: 513-517. 10.1016/j.ajem.2021.08.077
[992] Md. Shahed M, Abdullah ALM, Mohammad Sorowar H (2021) Death profiling of hospitalized patients with COVID-19: Experience from a specialized hospital in Bangladesh. medRxiv. 10.1101/2021.07.04.21259979
[993] Medetal, Iacute, BeyoĞLu AE, et al. (2022) PATIENT CHARACTERISTICS AND RISK FACTORS FOR MORTALITY IN 504 HOSPITALIZED PATIENTS DUE TO COVID-19. COVID-19 NEDENÍYLE HASTANEYE YATIRILMIŞ 504 HASTANIN ÖZELLÍKLERÍ VE MORTALÍTE AÇISINDAN RÍSK FAKTÖRLERÍ 85(1): 1-8. 10.26650/IUITFD.932128

[994] Mekolo D, Bokalli FA, Chi FM, et al. (2021) Clinical and epidemiological characteristics and outcomes of patients hospitalized for COVID-19 in Douala, Cameroon. Pan Afr Med J 38: 246. 10.11604/pamj.2021.38.246.28169
[995] Melillo F, Napolano A, Loffi M, et al. (2022) Myocardial injury in patients with SARS-CoV-2 pneumonia: Pivotal role of inflammation in COVID-19. Eur J Clin Invest 52(1): e13703. 10.1111/eci.13703
[996] Merino J, Joshi AD, Nguyen LH, et al. (2021) Diet quality and risk and severity of COVID-19: a prospective cohort study. Gut 70(11): 2096-2104. 10.1136/gutjnl-2021-325353
[997] Merza MA, Aswad SM, Sulaiman HM, Abdulah DM, Rasheed WS, Taib NI (2021) Clinical and epidemiological characteristics and outcomes of Coronavirus disease-19 patients in a large longitudinal study. Int J Health Sci (Qassim) 15(4): 29-41
[998] Miftode E, Miftode L, Coman I, et al. (2021) Diabetes Mellitus-A Risk Factor for Unfavourable Outcome in COVID-19 Patients-The Experience of an Infectious Diseases Regional Hospital. Healthcare (Basel) 9(7). 10.3390/healthcare9070788
[999] Miró Ò, Alquézar-Arbé A, Llorens P, et al. (2021) Comparison of the demographic characteristics and comorbidities of patients with COVID-19 who died in Spanish hospitals based on whether they were or were not admitted to an intensive care unit. Med Intensiva (Engl Ed) 45(1): 14-26. 10.1016/j.medin.2020.09.002
[1000] Moctezuma-Velázquez P, Miranda-Zazueta G, Ortiz-Brizuela E, et al. (2022) NAFLD determined by Dallas Steatosis Index is associated with poor outcomes in COVID-19 pneumonia: a cohort study. Intern Emerg Med. 10.1007/s11739-022-02933-x
[1001] Moftakhar L, Piraee E, Mohammadi Abnavi M, Moftakhar P, Azarbakhsh H, Valipour A (2021) Epidemiological Features and Predictors of Mortality in Patients with COVID-19 with and without Underlying Hypertension. Int J Hypertens 2021: 7427500. 10.1155/2021/7427500
[1002] Mohamed Ali S, Musa A, Omar Muhammed K, et al. (2021) Prolonged corrected QT interval in hospitalized patients with coronavirus disease 2019 in Dubai, United Arab Emirates: a single-center, retrospective study. J Int Med Res 49(11): 3000605211056834. 10.1177/03000605211056834
[1003] Mohammad Javad Z, Mohammad Javad Z, Mehrab S, et al. (2021) The Prevalence of Cardiovascular Risk Factors in Fatal Cases of COVID-19 in Fars Province, Iran. international cardiovascular research journal 15(1)
[1004] Mohsin FM, Nahrin R, Tonmon TT, et al. (2021) Lifestyle and Comorbidity-Related Risk Factors of Severe and Critical COVID-19 Infection: A Comparative Study Among Survived COVID-19 Patients in Bangladesh. Infect Drug Resist 14: 4057-4066. 10.2147/idr.S331470
[1005] Monárrez-Espino J, Zubía-Nevárez CI, Reyes-Silva L, et al. (2021) Clinical Factors Associated with COVID-19 Severity in Mexican Patients: Cross-Sectional Analysis from a Multicentric Hospital Study. Healthcare (Basel) 9(7). 10.3390/healthcare9070895
[1006] Mondal S, Singha A, Das D, et al. (2021) Prevalence of covid-19 infection and identification of risk factors among asymptomatic healthcare workers: A serosurvey involving multiple hospitals in west bengal. Journal of the Indian Medical Association 119(5): 21-27
[1007] Monfared A, Madani A, Taromsari M, et al. (2021) The Predictors of COVID-19 Disease Outcomes in Health Care Workers. ResearchSquare. 10.21203/rs.3.rs-954124/v1
[1008] Monserrat Villatoro J, Mejía-Abril G, Díaz García L, et al. (2022) A Case-Control of Patients with COVID-19 to Explore the Association of Previous Hospitalisation Use of Medication on the Mortality of COVID-19 Disease: A Propensity Score Matching Analysis. Pharmaceuticals (Basel) 15(1). 10.3390/ph15010078
[1009] Moon HJ, Kim K, Kang EK, Yang HJ, Lee E (2021) Prediction of COVID-19-related Mortality and 30-Day and 60-Day Survival Probabilities Using a Nomogram. J Korean Med Sci 36(35): e248. 10.3346/jkms.2021.36.e248
[1010] Moradzadeh R, Jamalian SM, Nazari J, et al. (2021) Age-standardized mortality rate and predictors of mortality among COVID-19 patients in Iran. J Educ Health Promot 10(1): 169. 10.4103/jehp.jehp_946_20
[1011] Motaib I, Zbiri S, Elamari S, Haoudar A, Chadli A, El Kettani C (2021) Cardiovascular Risk Factors and the Severity of COVID-19 Disease. Cureus 13(6): e15486. 10.7759/cureus.15486
[1012] Nabatchian F, Ashtiani M, Davoudi M, Teimourpour A, Davoudi N (2021) A Multivariate Analysis Model of Changes in Some Laboratory Parameters in Response to COVID-19, Diabetes, Gender, and Age. Clin Lab 67(8). 10.7754/Clin.Lab.2021.210106
[1013] Nag A, Ghosh S, Pal A, Saha A, Mandal SK (2021) Clinical and Biochemical Profile of COVID-19 Patients Admitted in a Tertiary Care Hospital of Kolkata. Journal of Clinical and Diagnostic Research 15(2)
[1014] Nair SC, Gasmelseed HI, Khan AA, et al. (2021) Assessment of mortality from COVID-19 in a multicultural multi-ethnic patient population. BMC Infect Dis 21(1): 1115. 10.1186/s12879-021-06762-9

[1015] Naqvi SF, Lakhani DA, Sohail AH, et al. (2021) Patients with idiopathic pulmonary fibrosis have poor clinical outcomes with COVID-19 disease: a propensity matched multicentre research network analysis. BMJ Open Respir Res 8(1). 10.1136/bmjresp-2021-000969
[1016] Naser MN, Al-Ghatam R, Darwish AH, et al. (2021) Risk factors, predictions, and progression of acute kidney injury in hospitalized COVID-19 patients: An observational retrospective cohort study. PLoS One 16(9): e0257253. 10.1371/journal.pone.0257253
[1017] Nasrullah A, Jayakrishnan T, Wedgeworth P, et al. (2021) Characteristics and Outcomes of COVID-19 Patients Admitted to Intensive Care Units in a Large Health System in Western Pennsylvania. Cureus 13(7): e16552. 10.7759/cureus.16552
[1018] Nateghi S, Gomari MM, Hosamirudsari H, et al. (2021) A historical cohort study to investigation of statins safety in COVID-19 hospitalized patients. Therapie. 10.1016/j.therap.2021.10.006
[1019] Navayi M, Fanoodi A, Salmani F, Abedi F, Shetty S, Riahi SM (2021) Over 60 years of age as an independent prognostic factor of in-hospital mortality among COVID-19 patients: a cohort study in an Iranian high-incidence area. Public Health 200: 33-38. 10.1016/j.puhe.2021.09.007
[1020] Nawghare P, Jain S, Chandnani S, et al. (2022) Predictors of Severity and Mortality in Chronic Liver Disease Patients With COVID-19 During the Second Wave of the Pandemic in India. Cureus 14(1): e20891. 10.7759/cureus.20891
[1021] Nazish ZZFTBHAK (2021) Risk factors associated with mortality in COVID-19 patients in Nishtar Hospital Multan. Professional Medical Journal 28(12): 1696-1700. 10.29309/TPMJ/2021.28.12.6632
[1022] Neda B, Ivats N, Hristo D, et al. (2021) Presentation, characteristics, treatments and outcomes of mechanically ventilated patients with COVID-19 in Bulgaria. medRxiv. 10.1101/2021.12.17.21267655
[1023] Nehara HR, Kumawat S, Gupta J, et al. (2022) Coronavirus Disease, Diabetes and Glucocorticoid a Terrible Trio for Invasive Mucormycosis: An Observational Study from Northwest Rajasthan. J Assoc Physicians India 69(12): 11-12
[1024] Nguyen NT, Chinn J, De Ferrante M, Kirby KA, Hohmann SF, Amin A (2021) Male gender is a predictor of higher mortality in hospitalized adults with COVID-19. PLoS One 16(7): e0254066. 10.1371/journal.pone.0254066
[1025] Nicolau J, Ayala L, Sanchís P, et al. (2021) Influence of nutritional status on clinical outcomes among hospitalized patients with COVID-19. Clin Nutr ESPEN 43: 223-229. 10.1016/j.clnesp.2021.04.013
[1026] Nimavat N, Singh S, Singh P, Singh SK, Sinha N (2021) Vitamin D deficiency and COVID-19: A case-control study at a tertiary care hospital in India. Ann Med Surg (Lond) 68: 102661. 10.1016/j.amsu.2021.102661
[1027] Ninomiya T, Otsubo K, Hoshino T, et al. (2021) Risk factors for disease progression in Japanese patients with COVID-19 with no or mild symptoms on admission. BMC Infect Dis 21(1): 850. 10.1186/s12879-021-06574-x
[1028] Nishida Y, Mita T, Hiki M, Matsushita Y, Naito T, Watada H (2022) Retrospective Study on the Effects of Glucose Abnormality on COVID-19 Outcomes in Japan. Diabetes Ther: 1-15. 10.1007/s13300-022-01206-2
[1029] Nogales Vasconcelos AM, Ishitani L, Abreu DMX, França E (2021) Covid Adult Mortality in Brazil: An Analysis of Multiple Causes of Death. Front Public Health 9: 788932. 10.3389/fpubh.2021.788932
[1030] Nuñez-Ramos JA, Forero Illera E, Quintero Barrios JL, et al. (2021) Prognostic Factors in Hospitalized COVID-19 Patients at the Start of the Pandemic in Colombia: A Retrospective Cohort Study. Cureus 13(5): e14865. 10.7759/cureus.14865
[1031] Nwaru CA, Santosa A, Franzén S, Nyberg F (2022) Occupation and COVID-19 diagnosis, hospitalisation and ICU admission among foreign-born and Swedish-born employees: a register-based study. J Epidemiol Community Health. 10.1136/jech-2021-218278
[1032] Odeh MM, Al Qaissieh R, Tarifi AA, et al. (2021) A prediction model of risk factors for complications among SARS-CoV2 positive patients: Cases from Jordan. J Infect Public Health 14(6): 689-695. 10.1016/j.jiph.2021.02.010
[1033] Oguz EG, Yeter HH, Akcay OF, et al. (2022) Predictive value of neutrophil-to-lymphocyte ratio in terms of need for intensive care unit and mortality in maintenance hemodialysis patients with COVID-19. Hemodial Int. 10.1111/hdi.13001
[1034] Olak AS, Susuki AM, Kanashiro M, Paoliello MMB, Aschner M, Urbano MR (2021) Risk factors associated with COVID-19-induced death in patients hospitalized in intensive care units (ICUs) in a city in Southern Brazil. Toxicol Rep 8: 1565-1568. 10.1016/j.toxrep.2021.07.020
[1035] Oliveira J, Gameiro J, Bernardo J, et al. (2021) Impact of Chronic RAAS Use in Elderly COVID-19 Patients: A Retrospective Analysis. J Clin Med 10(14). 10.3390/jcm10143147

[1036] Omran D, Al Soda M, Bahbah E, et al. (2021) Predictors of severity and development of critical illness of Egyptian COVID-19 patients: A multicenter study. PLoS One 16(9): e0256203. 10.1371/journal.pone.0256203
[1037] Ouedraogo E, Allard L, Bihan H, et al. (2021) The association of metabolic syndrome and COVID-19 deterioration. Nutrition Metabolism and Cardiovascular Diseases 31(11): 3236-3242. 10.1016/j.numecd.2021.08.036
[1038] Owings AH, Sloan ME, Liu JJ, Glover SC, Abraham GE, Senitko M (2021) Proton pump inhibitor use is associated with increased mortality in african american COVID-19 patients. American Journal of Respiratory and Critical Care Medicine 203(9). 10.1164/ajrccm-conference.2021.203.1_MeetingAbstracts.A3817
[1039] Özyilmaz SÖAEE, Uçar H, Ermiş E, Allahverdiyev S (2020) Relationship between mortality and troponin i levels in hospitalized patients with novel Coronavirus (COVID-19). Anatolian Journal of Cardiology 24(SUPPL 1): 112-113
[1040] Padmaprakash KV, Vardhan V, Thareja S, et al. (2021) Clinical characteristics and clinical predictors of mortality in hospitalised patients of COVID 19 : An Indian study. Med J Armed Forces India 77(Suppl 2): S319-s332. 10.1016/j.mjafi.2021.01.009
[1041] Page-Wilson G, Arakawa R, Nemeth S, et al. (2021) Obesity is independently associated with septic shock, renal complications, and mortality in a multiracial patient cohort hospitalized with COVID-19. PLoS One 16(8): e0255811. 10.1371/journal.pone.0255811
[1042] Panda S, Roy S, Garg RK, et al. (2022) COVID-19 disease in hospitalized young adults in India and China: Evaluation of risk factors predicting progression across two major ethnic groups. J Med Virol 94(1): 272-278. 10.1002/jmv.27315
[1043] Pandit RA, Gagana BN, Vaity C, et al. (2021) Clinical Characteristics and Outcomes of COVID-19 Patients Hospitalized in Intensive Care Unit. Indian J Crit Care Med 25(9): 992-1000. 10.5005/jp-journals-10071-23945
[1044] Pandita A, Gillani FS, Shi Y, et al. (2021) Predictors of severity and mortality among patients hospitalized with COVID-19 in Rhode Island. PLoS One 16(6): e0252411. 10.1371/journal.pone.0252411
[1045] Patel KHK, Li X, Quint JK, Ware JS, Peters NS, Ng FS (2021) Increasing adiposity and the presence of cardiometabolic morbidity is associated with increased Covid-19-related mortality: results from the UK Biobank. BMC Endocr Disord 21(1): 144. 10.1186/s12902-021-00805-7
[1046] Patel SR, Mukkera SR, Tucker L, et al. (2021) Characteristics, Comorbidities, Complications, and Outcomes Among 802 Patients With Severe Acute Respiratory Syndrome Coronavirus 2 in a Community Hospital in Florida. Crit Care Explor 3(5): e0416. 10.1097/cce.0000000000000416
[1047] Patil A, K C, Shenoy P, et al. (2021) A Prospective Longitudinal Study Evaluating The Influence of Immunosuppressives and Other Factors On COVID-19 in Autoimmune Rheumatic Diseases. ResearchSquare. 10.21203/rs.3.rs-805748/v1
[1048] Patone M, Thomas K, Hatch R, et al. (2021) Mortality and critical care unit admission associated with the SARS-CoV-2 lineage B.1.1.7 in England: an observational cohort study. Lancet Infect Dis 21(11): 1518-1528. 10.1016/s1473-3099(21)00318-2
[1049] Peñuelas O, Del Campo-Albendea L, de Aledo ALG, et al. (2021) Long-term survival of mechanically ventilated patients with severe COVID-19: an observational cohort study. Ann Intensive Care 11(1): 143. 10.1186/s13613-021-00929-y
[1050] Pérez-Cruz E, Castañón-González JA, Ortiz-Gutiérrez S, Garduño-López J, Luna-Camacho Y (2021) Impact of obesity and diabetes mellitus in critically ill patients with SARS-CoV-2. Obes Res Clin Pract 15(4): 402-405. 10.1016/j.orcp.2021.05.001
[1051] Pescatore JM, Sarmiento J, Hernandez-Acosta RA, Skaathun B, Quesada-Rodriguez N, Rezai K (2021) Glycemic control is associated with lower odds of mortality and successful extubation in severe COVID-19. J Osteopath Med 122(2): 111-115. 10.1515/jom-2021-0182
[1052] Pezel T, Garot P, Hovasse T, et al. (2021) Prognostic value of pre-hospitalization stress perfusion cardiovascular magnetic resonance to predict death in patients hospitalized for COVID-19. Arch Cardiovasc Dis 114(12): 781-792. 10.1016/j.acvd.2021.10.004
[1053] Philipose Z, Smati N, Wong CSJ, Aspey K, Mendall MA (2020) Obesity, old age and frailty are the true risk factors for COVID-19 mortality and not chronic disease or ethnicity. medRxiv. 10.1101/2020.08.12.20156257
[1054] Pop-Vicas A, Haleem A, Osman F, et al. (2021) Risk Factors and Mortality for Atypical Presentation of COVID-19 Infection in Hospitalized Patients - Lessons From the Early Pandemic. Wmj 120(2): 94-99
[1055] Prado PRD, Gimenes FRE, Lima MVM, Prado VBD, Soares CP, Amaral TLM (2021) Risk factors for death due to COVID-19, in the state of Acre, Brazil, 2020: a retrospective cohort study. Epidemiol Serv Saude 30(3): e2020676. 10.1590/s1679-49742021000300018
[1056] Prasad N, Behera MR, Bhatt M, et al. (2021) Outcomes of symptomatic coronavirus disease 19 in maintenance hemodialysis patient in India. Semin Dial 34(5): 360-367. 10.1111/sdi.13000

[1057] Purroy F, Arqué G (2021) Influence of thromboembolic events in the prognosis of COVID-19 hospitalized patients. Results from a cross sectional study. PLoS One 16(6): e0252351. 10.1371/journal.pone.0252351
[1058] Pustahija T, Ristić M, Medić S, et al. (2021) Epidemiological characteristics of COVID-19 travel-associated cases in Vojvodina, Serbia, during 2020. PLoS One 16(12): e0261840. 10.1371/journal.pone.0261840
[1059] Raghavan A, Nanditha A, Satheesh K, et al. (2021) Profile and prognosis of patients hospitalized for COVID-19 virus infection with and without diabetes - An observational study from South India. Diabetes Metab Syndr 15(4): 102143. 10.1016/j.dsx.2021.05.016
[1060] Raghubir SK, Kartik M, Harshad CA, et al. (2021) Greater Covid-19 Severity and Mortality in Hospitalized Patients in Second (Delta Variant) Wave Compared to the First: Single Centre Prospective Study in India. medRxiv. 10.1101/2021.09.03.21263091
[1061] Rahman MA, Shanjana Y, Tushar MI, et al. (2021) Hematological abnormalities and comorbidities are associated with COVID-19 severity among hospitalized patients: Experience from Bangladesh. PLoS One 16(7). 10.1371/journal.pone.0255379
[1062] Ramirez-Sandoval JC, Castillos-Ávalos VJ, Paz-Cortés A, et al. (2021) Very Low Vitamin D Levels are a Strong Independent Predictor of Mortality in Hospitalized Patients with Severe COVID-19. Arch Med Res. 10.1016/j.arcmed.2021.09.006
[1063] Ramkumar R, Rani D, Bhattacharjee S, et al. (2021) Epidemiology and clinical characteristics of COVID- 19 patients requiring critical care in a Tertiary care teaching hospital. J Anaesthesiol Clin Pharmacol 37(3): 366-370. 10.4103/joacp.JOACP_585_20
[1064] Rana R, Ranjan V, Kumar N, et al. (2021) Association of Underlying Comorbidities and progression of COVID-19 Infection Among 2586 Patients Hospitalized in the National Capital Region of India: A Retrospective Cohort Study. ResearchSquare. 10.21203/rs.3.rs-1184849/v1
[1065] Rangaswami M, Anitha M, Udhayanandhini G, Mariyappan T (2021) Clinical characteristics and outcome of covid-19 patients at government tertiary care institution in Vellore, Tamil Nadu, South India. International Archives of Integrated Medicine 8(6): 17-24
[1066] Razjouyan J, Helmer DA, Lynch KE, et al. (2021) Smoking Status and Factors associated with COVID-19 In-hospital Mortality among U.S. Veterans. Nicotine Tob Res. 10.1093/ntr/ntab223
[1067] Rehman FU, Omair SF, Memon F, et al. (2021) The Relation of ABO Blood Group to the Severity of Coronavirus Disease: A Cross-Sectional Study From a Tertiary Care Hospital in Karachi. Cureus 13(7): e16598. 10.7759/cureus.16598
[1068] Rehman S, Rehman N, Mumtaz A, Jiang J (2021) Association of Mortality-Related Risk Factors in Patients with COVID-19: A Retrospective Cohort Study. Healthcare (Basel) 9(11). 10.3390/healthcare9111468
[1069] Resurreccion WK, Hulsizer J, Shi Z, et al. (2021) Association of Sickle Cell Trait with Risk and Mortality of COVID-19: Results from the United Kingdom Biobank. Am J Trop Med Hyg 105(2): 368-371. 10.4269/ajtmh.20-1657
[1070] Reyes M, Cordova O, Montero Z, Suarez D, Calvo Y, Andrade N (2021) Predisposing clinical factors for admission to the Intensive Care Unit in patients with COVID-19. Investigacion Clinica 62: 110-116
[1071] Riahi T, Shokri S, Faiz SHR, et al. (2021) Clinical and Laboratory Predictors of Mortality in Hospitalized COVID-19 Patients. Iranian Red Crescent Medical Journal 23(5). 10.32592/ircmj.2021.23.5.531
[1072] Richardson S, Gitlin J, Kozel Z, et al. (2021) In-Hospital 30-Day Survival Among Young Adults With Coronavirus Disease 2019: A Cohort Study. Open Forum Infect Dis 8(6): ofab233. 10.1093/ofid/ofab233
[1073] Ritter LA, Britton N, Heil EL, et al. (2021) The Impact of Corticosteroids on Secondary Infection and Mortality in Critically Ill COVID-19 Patients. J Intensive Care Med 36(10): 1201-1208. 10.1177/08850666211032175
[1074] Robles-Pérez E, González-Díaz B, Miranda-García M, Borja-Aburto VH (2021) Infection and death by COVID-19 in a cohort of healthcare workers in Mexico. Scand J Work Environ Health 47(5): 349-355. 10.5271/sjweh.3970
[1075] Rodríguez A, Lista N, Flores Montes M, et al. (2021) COVID-19 in intensive care: which patients are most at risk of requiring mechanical ventilation? Actual SIDA infectol 29(105): 34-41
[1076] Roshan Kumar J, Anil S, Basant T, Indu KC, Shiv Kumar S (2021) Predisposing factors associated with the severity of the illness in adults with Covid-19 in Nepal. medRxiv. 10.1101/2021.05.16.21257280
[1077] Roushani JTDOMJ, Ip JTYYATLCRMPO, Garg AX, Blake PG (2021) Acute kidney injury requiring renal replacement therapy in people with COVID-19 disease in Ontario, Canada: a prospective analysis of risk factors and outcomes. Clinical Kidney Journal

[1078] Russo V, Bottino R, D'Andrea A, et al. (2021) Chronic Oral Anticoagulation and Clinical Outcome in Hospitalized COVID-19 Patients. Cardiovasc Drugs Ther: 1-8. 10.1007/s10557-021-07194-y
[1079] Saad EJ, Correa Barovero MA, Marucco FA, et al. (2021) [Clinical and epidemiological characteristics of patients hospitalized for SARS-CoV-2 infection in two hospitals in Córdoba]. Rev Fac Cien Med Univ Nac Cordoba 78(3): 303-312. 10.31053/1853.0605.v78.n3.32518
[1080] Šabanović Adilović A, Rizvanović N, Kovačević M, Adilović H (2021) Clinical characteristics, comorbidities and mortality in critically ill mechanically ventilated patients with Covid-19: a retrospective observational study. Med Glas (Zenica) 18(2): 378-383. 10.17392/1394-21
[1081] Safizadeh F, Nguyen TNM, Brenner H, Schöttker B (2021) Association of renin-angiotensin-aldosterone system inhibition with Covid-19 hospitalization and all-cause mortality in the UK biobank. Br J Clin Pharmacol. 10.1111/bcp.15192
[1082] Salamat MSS, Malundo AFG, Abad CLR, et al. (2021) Characteristics and factors associated with mortality of 200 COVID-19 patients at a Philippine COVID-19 tertiary referral center. Acta Medica Philippina 55(2): 173-182. 10.47895/AMP.V55I2.2845
[1083] Salerno S, Sun Y, Morris EL, et al. (2021) Comprehensive evaluation of COVID-19 patient short- and long-term outcomes: Disparities in healthcare utilization and post-hospitalization outcomes. PLoS One 16(10): e0258278. 10.1371/journal.pone.0258278
[1084] Salesi M, Sami R, Ahmadi SH, et al. (2021) The severity of symptoms and outcomes of patients admitted with rheumatic diseases and COVID-19;a prospective cohort study in Iran. Immunopathologia Persa 7(2): 8-8. 10.34172/ipp.2021.34
[1085] Salinas-Aguirre JE, Sánchez-García C, Rodríguez-Sanchez R, Rodríguez-Muñoz L, Díaz-Castaño A, Bernal-Gómez R (2021) Clinical characteristics and comorbidities associated with mortality in patients with COVID-19 in Coahuila (Mexico)☆ Características clínicas y comorbilidades asociadas a mortalidad en pacientes con COVID-19 en Coahuila (México). Revista clinica espanola
[1086] Samimagham HR, Arabi M, Hooshyar D, Kazemijahromi M (2020) The association of non-steroidal anti-inflammatory drugs with COVID-19 severity and mortality. Archives of Clinical Infectious Diseases 15(4): 1-5. 10.5812/ARCHCID.106847
[1087] Sampathkumar, Hanumaiah H, Rajiv A, et al. (2021) Incidence, Risk Factors and Outcome of COVID-19 Associated AKI- A Study from South India. J Assoc Physicians India 69(6): 11-12
[1088] Samuel LJ, Gaskin DJ, Trujillo AJ, Szanton SL, Samuel A, Slade E (2021) Race, ethnicity, poverty and the social determinants of the coronavirus divide: U.S. county-level disparities and risk factors. BMC Public Health 21(1): 1250. 10.1186/s12889-021-11205-w
[1089] Sandeep B, Bansidhar T, Dinesh J, et al. (2021) Secondary infections modify the overall course of hospitalized COVID-19 patients: A retrospective study from a network of hospitals across North India. medRxiv. 10.1101/2021.09.27.21264070
[1090] Sandoval M, Nguyen DT, Vahidy FS, Graviss EA (2021) Risk factors for severity of COVID-19 in hospital patients age 18-29 years. PLoS One 16(7): e0255544. 10.1371/journal.pone.0255544
[1091] Sands KE, Wenzel RP, McLean LE, et al. (2021) Patient characteristics and admitting vital signs associated with coronavirus disease 2019 (COVID-19)-related mortality among patients admitted with noncritical illness. Infect Control Hosp Epidemiol 42(4): 399-405. 10.1017/ice.2020.461
[1092] Sansone NMS, Boschiero MN, Ortega MM, et al. (2022) Severe Acute Respiratory Syndrome by SARS-CoV-2 Infection or Other Etiologic Agents Among Brazilian Indigenous Population: An Observational Study from the First Year of Coronavirus Disease (COVID)-19 Pandemic. Lancet Reg Health Am 8: 100177. 10.1016/j.lana.2021.100177
[1093] Santorelli G, McCooe M, Sheldon TA, Wright J, Lawton T (2021) Ethnicity, pre-existing comorbidities, and outcomes of hospitalised patients with COVID-19 [version 1;peer review: 1 not approved]. Wellcome Open Research 6: 1-11. 10.12688/WELLCOMEOPENRES.16580.1
[1094] Santoro F, Nuñez-Gil IJ, Vitale E, et al. (2022) Antiplatelet therapy and outcome in COVID-19: the Health Outcome Predictive Evaluation Registry. Heart 108(2): 130-136. 10.1136/heartjnl-2021-319552
[1095] Sara IT, Sara FS, Aalaa KS, Shereen AB, Shaimaa AA, Mariam KY (2021) Baseline Characteristics and Outcomes of 180 Egyptian COVID-19 Patients Admitted to Quarantine Hospitals of Ain Shams University: A Retrospective Comparative Study. medRxiv. 10.1101/2021.06.11.21258743
[1096] Sardenberg R, Roberto G, Silva C, et al. (2021) Factors Related to Survival in Intensive Care Unit Patients with Covid-19: A study from a Single Center in Brazil. ResearchSquare. 10.21203/rs.3.rs-892542/v1
[1097] Sardinha DM, do Socorro Pompeu de Loiola R, Ferreira A, et al. (2021) Risk factors associated with the severity of COVID-19 in a region of the Brazilian Amazon. Sci Rep 11(1): 20569. 10.1038/s41598-021-00009-y

[1098] Scarpioni R, Valsania T, Albertazzi V, et al. (2021) Acute kidney injury, a common and severe complication in hospitalized patients during the COVID-19 pandemic. J Nephrol 34(4): 1019-1024. 10.1007/s40620-021-01087-x
[1099] Schilling J, Lehfeld AS, Schumacher D, et al. (2021) Disease severity of the first COVID-19 wave in Germany using reporting data from the national notification system. J Health Monit 5(Suppl 11): 2-19. 10.25646/7170
[1100] Senewe Felly PPNEMRLAM, Sulistiyowati N (2021) Pengaruh penyakit penyerta/komorbid dan karakteristik individu dengan kejadian COVID-19 di Kota Bogor tahun 2020. Jurnal Ekologi Kesehatan 20(2): 69-79. https://ejournal2.litbang.kemkes.go.id/index.php/jek/article/view/5114
[1101] Serván-Mori E, Seiglie JA, Gómez-Dantés O, Wirtz VJ (2022) Hospitalisation and mortality from COVID-19 in Mexican indigenous people: a cross-sectional observational study. J Epidemiol Community Health 76(1): 16-23. 10.1136/jech-2020-216129
[1102] Shabaka A, Gruss E, Landaluce-Triska E, et al. (2021) Late thrombotic complications after SARS-CoV-2 infection in hemodialysis patients. Hemodial Int 25(4): 507-514. 10.1111/hdi.12935
[1103] Shadrivova O, Gusev D, Vashukova M, et al. (2021) COVID-19-Associated Pulmonary Aspergillosis in Russia. J Fungi (Basel) 7(12). 10.3390/jof7121059
[1104] Shapiro AE, Bender Ignacio RA, Whitney BM, et al. (2021) Factors associated with severity of COVID-19 disease in a multicenter cohort of people with HIV in the United States, March-December 2020. medRxiv. 10.1101/2021.10.15.21265063
[1105] Sharif N, Opu RR, Ahmed SN, et al. (2021) Prevalence and impact of comorbidities on disease prognosis among patients with COVID-19 in Bangladesh: A nationwide study amid the second wave. Diabetes Metab Syndr 15(4): 102148. 10.1016/j.dsx.2021.05.021
[1106] Sharma S, Paneru HR, Shrestha GS, Shrestha PS, Acharya SP (2021) Characteristics and Outcome of Patients with COVID-19 Undergoing Invasive Mechanical Ventilation for Respiratory Failure in a Tertiary Level Hospital in Nepal. J Nepal Health Res Counc 19(2): 396-401. 10.33314/jnhrc.v19i2.3623
[1107] Sharma S, Sharma AK, Dalela G, et al. (2021) Association of SARS CoV-2 cycle threshold (Ct) with outcomes in COVID-19: Hospital-based study. Journal of Association of Physicians of India 69(7)
[1108] Shi Q, Zhao K, Yu J, et al. (2020) Clinical characteristics of 101 COVID-19 nonsurvivors in Wuhan, China: a retrospective study. medRxiv. 10.1101/2020.03.04.20031039
[1109] Shin EK, Choi HY, Hayes N (2021) Anatomy of comorbidity complexity of COVID-19: the case of hospitalized South Korean patients. Epidemiology and health: e2021035. 10.4178/epih.e2021035
[1110] Shirley C, Ebenezer O-M, Delali A (2021) Epidemiological Study of COVID-19 Infections: Case of Ga East Municipal Hospital Treatment Centre - Kwabenya-Ghana. medRxiv. 10.1101/2021.08.11.21261917
[1111] Shoaib N, Noureen N, Munir R, et al. (2021) COVID-19 severity: Studying the clinical and demographic risk factors for adverse outcomes. PLoS One 16(8): e0255999. 10.1371/journal.pone.0255999
[1112] Sidhu G, Samson R, Nedunchezian SH, Srivastav S, Dixit N, Le Jemtel TH (2021) COVID 19 in-hospital mortality, body mass index and obesity related conditions. J Diabetes Complications 35(12): 108054. 10.1016/j.jdiacomp.2021.108054
[1113] Sindhu C, Prasad P, Elumalai R, Matcha J (2022) Clinical profile and outcomes of COVID-19 patients with acute kidney injury: a tertiary centre experience from South India. Clin Exp Nephrol 26(1): 36-44. 10.1007/s10157-021-02123-7
[1114] Singhal R, Cardoso VR, Wiggins T, et al. (2021) 30-day morbidity and mortality of sleeve gastrectomy, Roux-en-Y gastric bypass and one anastomosis gastric bypass: a propensity score-matched analysis of the GENEVA data. Int J Obes (Lond): 1-8. 10.1038/s41366-021-01048-1
[1115] Sinha S, Swami R, Shakir A, Salman Ali S, Bansode J, Mehta K (2021) Clinical Profile and Outcome of Hemodialysis Patients with SARS COV2 Infection in a Tertiary Care Centre in Mumbai, India. Indian J Nephrol 31(5): 442-448. 10.4103/ijn.IJN_377_20
[1116] Siobhán BC, Elliot M, Alexander E, et al. (2021) Factors Associated With Clinical Progression to Severe COVID-19 in People With Cystic Fibrosis: A Global Observational Study. SSRN
[1117] Son M, Jo YS, Jo S, Park K (2021) Impact of COVID-19 on Heart Failure Patients in South Korea. Int Heart J 62(5): 1083-1090. 10.1536/ihj.21-198
[1118] Son M, Jo YS, Jo S, Park K (2021) Impact of COVID-19 on Heart Failure Patients in South Korea A Nationwide Population-Based Study. International Heart Journal 62(5): 1083-1090. 10.1536/ihj.21-198
[1119] Song J, Park DW, Cha JH, et al. (2021) Clinical course and risk factors of fatal adverse outcomes in COVID-19 patients in Korea: a nationwide retrospective cohort study. Sci Rep 11(1): 10066. 10.1038/s41598-021-89548-y

[1120] Soria MLJB, Quiwa LQ, Calvario MKJS, Duya JED, Punongbayan RB, Ting FIL (2021) The philippine coronavirus disease 2019 (Covid-19) profile study: Clinical profile and factors associated with mortality of hospitalized patients. Phillippine Journal of Internal Medicine 59(1): 37-58
[1121] Stanetić K, Stanetić B, Petrović V, et al. (2021) The Influence of Different Risk Factors on COVID-19 Outcomes in Adult Patients - An Observational-Descriptive Study. Acta Med Acad 50(2): 308-316. 10.5644/ama2006-124.348
[1122] Stefano M, Andrea B, Daniela C, et al. (2021) Malnutrition risk as a negative prognostic factor in COVID-19 patients. Clin Nutr ESPEN 45: 369-373. 10.1016/j.clnesp.2021.07.016
[1123] Sterling RK, Shin D, Shin Y, et al. (2021) FIB-4 Predicts Need for Mechanical Ventilation in a Multi-ethnic National cohort of COVID-19. Hepatology communications. 10.1002/hep4.1737
[1124] Sticchi A, Cereda A, Toselli M, et al. (2021) Diabetes and mortality in patients with COVID-19: Are we missing the link? Anatol J Cardiol 25(6): 376-379. 10.5152/AnatolJCardiol.2021.29132
[1125] Sultan M, Kene D, Waganew W, et al. (2021) Clinical Characteristics of COVID-19 Related Deaths in Ethiopia. Ethiop J Health Sci 31(2): 223-228. 10.4314/ejhs.v31i2.3
[1126] Sultan Mehmood KZeHANHMYJMWSS (2021) CLINICAL PROFILES AND OUTCOMES OF PATIENTS ADMITTED WITH MODERATE TO CRITICAL CORONAVIRUS DISEASE 2019 (COVID-19). Pakistan Armed Forces Medical Journal(2): 694-694
[1127] Sümer ŞUO, Aktuğ-Demir N, Çifci ŞTB, Kılınçer A, Türk-Dağı H, Demir LS (2020) Clinical and laboratory characteristics of COVID-19 cases followed in selçuk university faculty of medicine. Klimik Dergisi 33(2): 122-127. 10.5152/kd.2020.26
[1128] Sun L, Shen L, Fan J, et al. (2020) Clinical features of patients with coronavirus disease 2019 from a designated hospital in Beijing, China. J Med Virol 92(10): 2055-2066. 10.1002/jmv.25966
[1129] Sun Y, Jiang N, Li Z, et al. (2021) A Retrospective Study of 268 Patients with SARS-CoV-2 Infection to Evaluate the Association Between Blood Glucose and Severity of COVID-19 Pneumonia and Patient Mortality. Med Sci Monit 27: e932156. 10.12659/msm.932156
[1130] Sundaramurthy R, Balasubramanian S, Ganesan V, et al. (2021) Clinical and Laboratory Factors in Predicting Mortality Among COVID-19 RT-PCR Positive Patients: A Retrospective Observational Study From a Tertiary Care Center. Cureus 13(11): e19791. 10.7759/cureus.19791
[1131] Sunnetcioglu A, Sunnetcioglu M, Gurbuz E, Bedirhanoglu S, Erginoguz A, Celik S (2021) Serum 25(OH)D Deficiency and High D-Dimer Levels are Associated with COVID-19 Pneumonia. Clin Lab 67(7). 10.7754/Clin.Lab.2020.201050
[1132] Tabares RG, González FAA, Villa EO, Reyes SFR, Echevarría IC (2020) Predictors of poor prognosis in patients with COVID-19. Revista Cubana de Medicina Militar 49(4): 1-16
[1133] Takeshita Y, Terada J, Fujita R, et al. (2021) Coronary artery calcium score may be a novel predictor of COVID-19 prognosis: a retrospective study. BMJ Open Respir Res 8(1). 10.1136/bmjresp-2021-000923
[1134] Tawakul AA, Alharbi AH, Basahal AM, et al. (2021) Neurological Symptoms and Complications of COVID-19 Among Patients in a Tertiary Hospital in Saudi Arabia. Cureus 13(11): e19200. 10.7759/cureus.19200
[1135] Tenreiro P, Ramalho A, Santos P (2022) COVID-19 patients followed in Portuguese Primary Care: a retrospective cohort study based on the national case series. Fam Pract 39(1): 52-58. 10.1093/fampra/cmab098
[1136] Terada M, Ohtsu H, Saito S, et al. (2021) Risk factors for severity on admission and the disease progression during hospitalisation in a large cohort of patients with COVID-19 in Japan. BMJ Open 11(6): e047007. 10.1136/bmjopen-2020-047007
[1137] Toofan F, Hosseini SM, Alimohammadzadeh K, Jafari M, Bahadori M (2021) Impact of comorbidities on mortality in hospitalized patients with COVID-19: An experience from Iran. Journal of Education and Health Promotion 10(1). 10.4103/jehp.jehp_1589_20
[1138] Toori KU, Qureshi MA, Chaudhry A (2022) Pre-morbidity and COVID-19 disease outcomes in Pakistani population: A cross-sectional study. Pak J Med Sci 38(1): 287-292. 10.12669/pjms.38.1.4235
[1139] Tornhammar P, Jernberg T, Bergström G, et al. (2021) Association of cardiometabolic risk factors with hospitalisation or death due to COVID-19: population-based cohort study in Sweden (SCAPIS). BMJ Open 11(9): e051359. 10.1136/bmjopen-2021-051359
[1140] Torre GM, Montelongo-Mercado EA, Noyola-Villalobos HF, et al. (2021) Epidemiology of hospitalized patients with COVID-19 in a tertiary care hospital. Gac Med Mex 157(3): 237-244. 10.24875/gmm.M21000552
[1141] Trimaille A, Ribeyrolles S, Fauvel C, et al. (2021) Cardiovascular Characteristics and Outcomes of Young Patients with COVID-19. J Cardiovasc Dev Dis 8(12). 10.3390/jcdd8120165
[1142] Tripathi S, Sayed IA, Dapul H, et al. (2021) Risk Factors for Critical Coronavirus Disease 2019 and Mortality in Hospitalized Young Adults: An Analysis of the Society of Critical Care Medicine Discovery Viral Infection and Respiratory Illness Universal Study (VIRUS) Coronavirus Disease 2019 Registry. Crit Care Explor 3(8): e0514. 10.1097/cce.0000000000000514

[1143] Tsuchihashi Y, Arima Y, Takahashi T, et al. (2021) Clinical Characteristics and Risk Factors for Severe Outcomes of Novel Coronavirus Infection, January-March 2020, Japan. J Epidemiol 31(8): 487-494. 10.2188/jea.JE20200519
[1144] Turan O, Mirici A, Duru Akçalı S, et al. (2021) Characteristics of Hospitalized COVID-19 Patients and Parameters Associated with Severe Pneumonia. International journal of clinical practice: e14786. 10.1111/ijcp.14786
[1145] Umakanthan S, Senthil S, John S, et al. (2021) The protective role of statins in COVID-19 patients: a retrospective observational study. Transl Med Commun 6(1): 22. 10.1186/s41231-021-00102-4
[1146] Umeh C, Maguwudze S, Torbela A, et al. (2021) Predictors of Mortality in COVID-19 Patients in Southern California - Retrospective Multicenter Study. Cureus 13(9): e18137. 10.7759/cureus.18137
[1147] Umeh C, Tuscher L, Ranchithan S, Watanabe K, Gupta R (2022) Predictors of COVID-19 Mortality in Critically Ill ICU Patients: A Multicenter Retrospective Observational Study. Cureus Journal of Medical Science 14(1): 6-6. 10.7759/cureus.20952
[1148] Ushigome E, Hamaguchi M, Sudo K, et al. (2022) Impact of untreated diabetes and COVID-19-related diabetes on severe COVID-19. Heliyon 8(1): e08801. 10.1016/j.heliyon.2022.e08801
[1149] Vakrani GP, Nambakam T (2021) Outcome of COVID-19 in patients requiring haemodialysis - A retrospective observational study. Journal of Clinical and Diagnostic Research 15(11): OC23-OC25. 10.7860/JCDR/2021/51889.15613
[1150] Vera-Zertuche JM, Mancilla-Galindo J, Tlalpa-Prisco M, et al. (2021) Obesity is a strong risk factor for short-term mortality and adverse outcomes in Mexican patients with COVID-19: a national observational study. Epidemiol Infect 149: e109. 10.1017/s0950268821001023
[1151] Verma A, Patyal A, Mathur M, Choudhary S, Mathur N (2021) Sociodemographic and clinical characteristics associated with COVID mortality among hospitalized patients in Rajasthan: A retrospective observational study. J Family Med Prim Care 10(9): 3319-3324. 10.4103/jfmpc.jfmpc_445_21
[1152] Verna EC, Landis C, Brown RS, Jr., et al. (2021) Factors Associated with Readmission in the US Following Hospitalization with COVID-19. Clin Infect Dis. 10.1093/cid/ciab464
[1153] Vetrano DL, Tazzeo C, Palmieri L, et al. (2021) Comorbidity status of deceased COVID-19 in-patients in Italy. Aging Clin Exp Res 33(8): 2361-2365. 10.1007/s40520-021-01914-y
[1154] Viera-Segura O, Vega-Magaña N, García-Chagollán M, et al. (2021) A Comprehensive Descriptive Epidemiological and Clinical Analysis of SARS-CoV-2 in West-Mexico during COVID-19 Pandemic 2020. Int J Environ Res Public Health 18(20). 10.3390/ijerph182010644
[1155] Vinay B, Mitali S, Ankita V, Sonal J (2021) Comparison of Clinical, Haematological, Biochemical Findings and Significance of Co-morbidities amongst COVID-19 Positive Survivors and Nonsurvivors. Journal of Clinical and Diagnostic Research 15(4)
[1156] Violi F, Ceccarelli G, Cangemi R, et al. (2021) Arterial and venous thrombosis in coronavirus 2019 disease (Covid-19): relationship with mortality. Intern Emerg Med 16(5): 1231-1237. 10.1007/s11739-020-02621-8
[1157] Vitoria Berg C, Thais Araujo S, Julio C-A, Marcelo R-A (2021) Risk Assessment and Rationalization of Health Resource Allocation: Lessons From the Brazilian COVID-19 Cohort in 2020. SSRN
[1158] Volkman HR, Pérez-Padilla J, Wong JM, et al. (2021) Characteristics and clinical outcomes of patients hospitalized with laboratory-confirmed COVID-19-Puerto Rico, March-August 2020. PLoS One 16(12): e0260599. 10.1371/journal.pone.0260599
[1159] Weizman O, Mika D, Cellier J, et al. (2021) Characteristics and impact of cardiovascular comorbidities on coronavirus disease 2019 in women: A multicentre cohort study. Arch Cardiovasc Dis 114(5): 394-406. 10.1016/j.acvd.2021.04.002
[1160] Wen X, Gao L, Jiang D, et al. (2020) Clinical characteristics and predictive value of low CD4+T cell count in patients with moderate and severe COVID-19: A multicenter retrospective study. ResearchSquare. 10.21203/rs.3.rs-46009/v2
[1161] Wilkinson TJ, Lightfoot CJ, Smith AC, Yates T, Khunti K, Zaccardi F (2022) Association of Ethnicity and Socioeconomic Status With COVID-19 Hospitalization and Mortality in Those With and Without Chronic Kidney Disease. Kidney Int Rep 7(2): 334-338. 10.1016/j.ekir.2021.11.040
[1162] Wilkinson TJ, Yates T, Baker LA, Zaccardi F, Smith AC (2021) Sarcopenic obesity and the risk of hospitalization or death from coronavirus disease 2019: findings from UK Biobank. JCSM Rapid Commun. 10.1002/rco2.47
[1163] Windham S, Wilson MP, Fling C, et al. (2021) Elevated glycohemoglobin is linked to critical illness in CoVID-19: a retrospective analysis. Ther Adv Infect Dis 8: 20499361211027390. 10.1177/20499361211027390
[1164] Wu S, Zhou K, Misra-Hebert A, Bena J, Kashyap SR (2022) Impact of Metabolic Syndrome on Severity of COVID-19 Illness. Metab Syndr Relat Disord. 10.1089/met.2021.0102

[1165] Xiao F, Zhou YC, Zhang MB, et al. (2021) Hyperglycemia and blood glucose deterioration are risk factors for severe COVID-19 with diabetes: A two-center cohort study. J Med Virol. 10.1002/jmv.27556
[1166] Yadla M, Vadakkeveetil AK, Cherian A, Rahul (2021) Clinical features and outcomes of 84 COVID-Positive hemodialysis patients in a resource poor setting from India. Saudi J Kidney Dis Transpl 32(2): 504-509. 10.4103/1319-2442.335463
[1167] Yanagisawa S, Oikawa Y, Takagi S, et al. (2021) HbA1c level may be a risk factor for oxygen therapy requirement in patients with coronavirus disease 2019. J Diabetes Investig. 10.1111/jdi.13743
[1168] Yang HH, Wu TJ, Yu AC, Wells C, Orshansky G, Lee JT (2022) Predictors of Death, Survival, Need for Intubation, and Need for Oxygen Support Among Admitted COVID-19 Patients of the Veterans Affairs Greater Los Angeles Healthcare System. Mil Med. 10.1093/milmed/usab550
[1169] Yanti B, Zulfikar T, Kurniawan FD, et al. (2021) The presentation of clinical characteristics, comorbidities, and outcomes among covid-19 confirmed patients in banda aceh, Indonesia. Bali Medical Journal 10(2): 562-566. 10.15562/bmj.v10i2.2415
[1170] Yates T, Summerfield A, Razieh C, et al. (2022) A population-based cohort study of obesity, ethnicity and COVID-19 mortality in 12.6 million adults in England. Nat Commun 13(1): 624. 10.1038/s41467-022-28248-1
[1171] Yates T, Zaccardi F, Islam N, et al. (2021) Obesity, chronic disease, age, and in-hospital mortality in patients with covid-19: analysis of ISARIC clinical characterisation protocol UK cohort. BMC Infect Dis 21(1): 717. 10.1186/s12879-021-06466-0
[1172] Yen TE, Kim A, Benson ME, Ratnaparkhi S, Woolley AE, Mc Causland FR (2022) Serum Sodium, Patient Symptoms, and Clinical Outcomes in Hospitalized Patients with COVID-19. J Prim Care Community Health 13: 21501319211067349. 10.1177/21501319211067349
[1173] Yip TC, Wong VW, Lui GC, et al. (2021) Current and Past Infections of HBV Do Not Increase Mortality in Patients With COVID-19. Hepatology 74(4): 1750-1765. 10.1002/hep.31890
[1174] Yordanov Y, Dinh A, Bleibtreu A, et al. (2021) Clinical characteristics and factors associated with hospital admission or death in 43 103 adult outpatients with coronavirus disease 2019 managed with the Covidom telesurveillance solution: a prospective cohort study. Clin Microbiol Infect 27(8): 1158-1166. 10.1016/j.cmi.2021.04.010
[1175] Younossi ZM, Stepanova M, Lam B, et al. (2021) Independent Predictors of Mortality Among Patients With NAFLD Hospitalized With COVID-19 Infection. Hepatol Commun. 10.1002/hep4.1802
[1176] Yue J, Zhou Y, Xu H, et al. (2021) Characteristic analysis and comparison of glycolipid metabolism in patients with coronavirus disease 2019 in common condition and severe cases. Journal of Shanghai Jiaotong University (Medical Science) 41(3): 355-359. 10.3969/j.issn.1674-8115.2021.03.012
[1177] Zafar M, Karkhanis M, Shahbaz M, et al. (2021) Vitamin D levels and mortality with SARS-COV-2 infection: a retrospective two-centre cohort study. Postgrad Med J. 10.1136/postgradmedj-2021-140564
[1178] Zeng J, Liu X, Wang S, et al. (2021) The association between BMI and metabolically unhealthy status with COVID-19 mortality: Based on 3019 inpatients from Wuhan, China. Nutr Metab Cardiovasc Dis 31(11): 3219-3226. 10.1016/j.numecd.2021.07.030
[1179] Zhang H, Wu Y, He Y, et al. (2021) Age-Related Risk Factors and Complications of Patients With COVID-19: A Population-Based Retrospective Study. Front Med (Lausanne) 8: 757459. 10.3389/fmed.2021.757459
[1180] Zhang Y, Khullar D, Wang F, et al. (2021) Socioeconomic variation in characteristics, outcomes, and healthcare utilization of COVID-19 patients in New York City. PLoS One 16(7): e0255171. 10.1371/journal.pone.0255171
[1181] Zhang ZQ, Wan JQ, Zhu SK, et al. (2021) Analysis of cardiovascular disease factors on SARS-CoV-2 infection severity. Med Clin (Barc). 10.1016/j.medcli.2021.09.014
[1182] Zhao Y, Xing H (2022) Influence of Fasting Plasma Glucose Level on Admission of COVID-19 Patients: A Retrospective Study. J Diabetes Res 2022: 7424748. 10.1155/2022/7424748
[1183] Zirpe K, Pote P, Deshmukh A, Gurav SK, Tiwari AM, Suryawanshi P (2021) A Retrospective Analysis of Risk Factors of COVID-19 Associated Mucormycosis and Mortality Predictors: A Single-Center Study. Cureus 13(10): e18718. 10.7759/cureus.18718
[1184] Zirpe KG, Tiwari AM, Gurav SK, et al. (2021) Timing of invasive mechanical ventilation and mortality among patients with severe COVID-19-associated acute respiratory distress syndrome (CARDS). Indian J Crit Care Med 25(5): 493-498. 10.5005/jp-journals-10071-23816
[1185] 刘明科, 胡良波, 应洁, 陈暇女, 郭定波, 李鹤 (2020) Comparison of Clinical and CT Imaging Features of Mild and Severe Patients with COVID-19. 暨南大学学报(自然科学与医学版). 10.11778/j.jdxb.2020.05.004

[1186] 季烨龙, 吴洋, 冷燕, 张艺, 张爱宁, 夏中元 (2020) Clinical Features and Prognosis of Patients with Novel Coronavirus Pneumonia Complicated with Diabetes. 海南医学院学报. 10.13210/j.cnki.jhmu.20200617.004
[1187] 熊鹏, 孙文武, 朱珉, 朱明闯, 李骏, 喻莉 (2020) Clinical Characteristics of 660 Patients with COVID-19. 实用医学杂志
[1188] 郭 宁黄葛胡鑫 (2021) Analysis of acute myocardial injury in patients with novel coronavirus pneumonia and its risk factors. Journal of Modern Medicine & Health 37(3): 388-395. 10.3969/j.issn.1009-5519.2021.03.008
[1189] 陈琳, 杨小平, 郑南红, et al. (2020) Clinical Characteristics of 67 Discharged Cases of Corona Virus Disease 2019. 中华医院感染学杂志
[1190] 陶飞, 杨飞, 阮细祥, 夏德志, 李家胜, 吴翔 (2020) Clinical Characteristics of 382 Hospitalized Patients with COVID-19. 实用医学杂志
[1191] 黄澄澄, 庞科, 高红丽 (2020) Clinical Characteristics of Coronavirus Disease 2019 in A Chongqing Hospital: Analysis of 89 Cases. 第三军医大学学报
[1192] Rawshani A, Kjölhede EA, Rawshani A, et al. (2021) Severe COVID-19 in people with type 1 and type 2 diabetes in Sweden: A nationwide retrospective cohort study. Lancet Reg Health Eur 4: 100105. 10.1016/j.lanepe.2021.100105
[1193] Tjede F, Francesco I, Joana Gomes D, et al. (2021) Age Specific Associations Between Underlying Conditions and Hospitalisation, Death and In-Hospital Death Among Confirmed COVID-19 Cases: A Multi-Country Study Based on Surveillance Data. SSRN
[1194] Gao M, Wang Q, Piernas C, et al. (2022) Associations between body composition, fat distribution and metabolic consequences of excess adiposity with severe COVID-19 outcomes: observational study and Mendelian randomisation analysis. Int J Obes (Lond): 1-8. 10.1038/s41366-021-01054-3
[1195] Mondal MK, Jahan S, Yeasmeen S, et al. (2021) Demographic and Clinical Profile of COVID-19 ICU Patients of Bangabandhu Sheikh Mujib Medical University, Bangladesh. Mymensingh Med J 30(4): 1117-1123
[1196] Ando W, Horii T, Uematsu T, Hanaki H, Atsuda K, Otori K (2021) Impact of overlapping risks of type 2 diabetes and obesity on coronavirus disease severity in the United States. Sci Rep 11(1): 17968. 10.1038/s41598-021-96720-x
[1197] Cromer SJ, Colling C, Schatoff D, et al. (2022) Newly diagnosed diabetes vs. pre-existing diabetes upon admission for COVID-19: Associated factors, short-term outcomes, and long-term glycemic phenotypes. J Diabetes Complications: 108145. 10.1016/j.jdiacomp.2022.108145
[1198] Dave JA, Tamuhla T, Tiffin N, et al. (2021) Risk factors for COVID-19 hospitalisation and death in people living with diabetes: A virtual cohort study from the Western Cape Province, South Africa. Diabetes Res Clin Pract 177: 108925. 10.1016/j.diabres.2021.108925
[1199] De Luca G, Nardin M, Algowhary M, et al. (2021) Renin-angiotensin system inhibitors and mortality among diabetic patients with STEMI undergoing mechanical reperfusion during the COVID-19 pandemic. Diabet Epidemiol Manag 4: 100022. 10.1016/j.deman.2021.100022
[1200] Demeterco-Berggren C, Ebekozien O, Rompicherla S, et al. (2022) Age and Hospitalization Risk in People With Type 1 Diabetes and COVID-19: Data From the T1D Exchange Surveillance Study. J Clin Endocrinol Metab 107(2): 410-418. 10.1210/clinem/dgab668
[1201] Gao M, Piernas C, Astbury NM, et al. (2021) Associations between body-mass index and COVID-19 severity in 6.9 million people in England: a prospective, community-based, cohort study. Lancet Diabetes & Endocrinology 9(6): 350-359. 10.1016/s2213-8587(21)00089-9
[1202] Ge J, Pletcher MJ, Lai JC (2021) Outcomes of SARS-CoV-2 Infection in Patients With Chronic Liver Disease and Cirrhosis: A National COVID Cohort Collaborative Study. Gastroenterology 161(5): 1487-1501.e1485. 10.1053/j.gastro.2021.07.010
[1203] Lin L, Chen Z, Ding T, et al. (2021) Newly-Diagnosed Diabetes and Sustained Hyperglycemia are Associated with Poorer Outcomes in COVID-19 Inpatients Without Pre-Existing Diabetes. Diabetes Metab Syndr Obes 14: 4469-4482. 10.2147/dmso.S332819
[1204] Makker J, Sun H, Patel H, et al. (2021) Impact of Prediabetes and Type-2 Diabetes on Outcomes in Patients with COVID-19. Int J Endocrinol 2021: 5516192. 10.1155/2021/5516192
[1205] Gokhale CN, Chavhan SS, Mahajan HN, Adsul BB, Kumbhar MA, Ingale AR (2021) A comparative cross-sectional analysis on outcomes of Covid-19 patients requiring dialysis. J Family Med Prim Care 10(9): 3228-3232. 10.4103/jfmpc.jfmpc_12_21

[1206] Md Ikhsan M, Dante Saksono H, Ida Ayu K, et al. (2022) Increased Risk of Poor Clinical Outcome in COVID-19 Patients with Diabetes Mellitus and in-hospital Mortality Predictors: A Retrospective Cohort from a Tertiary Hospital in Indonesia. medRxiv. 10.1101/2021.12.30.21266217
[1207] Adir Y, Humbert M, Saliba W (2021) Association between sex and SARS-CoV-2 infection and hospitalisation as a result of COVID-19. Lancet Respir Med 9(8): e75-e76. 10.1016/s2213-2600(21)00239-3
[1208] Vare AA, Yellambkar S, Farheen A, Nandedkar V, Bhombe SS, Shah R (2021) Incidence, cumulative mortality and factors affecting the outcome of COVID-19-associated mucormycosis from Western India. Indian J Ophthalmol 69(12): 3678-3683. 10.4103/ijo.IJO_1767_21
[1209] Ong AN, Tan CC, Cañete MT, Lim BA, Robles J (2021) Association Between Metformin Use and Mortality among Patients with Type 2 Diabetes Mellitus Hospitalized for COVID-19 Infection. J ASEAN Fed Endocr Soc 36(2): 133-141. 10.15605/jafes.036.02.20
[1210] Arturo V-C, Eva Fabiana M, Nancy Segovia C, et al. (2021) Características clínico-epidemiológicas de pacientes confirmados con COVID-19 del Departamento de Alto Paraná, Paraguay. 11(1)
[1211] Asgari AHES-MSRMASNM (2021) Vitamin D Insufficiency in Disease Severity and Prognosis of the Patients With SARS Corona Virus-2 Infection. Acta Medica Iranica 59(11): 662-668
[1212] 凌学斌, 季永能, 林初芬, 李天发, 王军, 蔡毅 (2020) Clinical Presentations , Laboratory Examination and CT Characteristics in 457 Cases with Mild of COVID-2019. 暨南大学学报(自然科学与医学版). 10.11778/j.jdxb.2020.05.002
[1213] Lesniak C, Ong R, Akula MS, et al. (2021) Inpatient glycemic control and outcome of COVID-19 patients: A retrospective cohort. SAGE Open Med 9: 20503121211039105. 10.1177/20503121211039105
[1214] Filiz M, Yilmaz G, Fidan G, et al. (2021) A Tertiary Care Hospital Experience During the COVID-19 Pandemic. Flora Infeksiyon Hastaliklari Ve Klinik Mikrobiyoloji Dergisi 26(2): 257-266. 10.5578/flora.20219805
[1215] Danielle KL, Jessica EM, Siroon B, et al. (2020) Outcomes in Patients with Overweight and Obesity Hospitalized with COVID-19: An International, Multi-Center Retrospective Analysis. SSRN. 10.2139/ssrn.3688247
[1216] Cortez KJC, Demot BA, Bartolo SS, et al. (2021) Clinical characteristics and outcomes of COVID-19 patients in a tertiary hospital in Baguio City, Philippines. Western Pacific Surveillance and Response 12(4): 11-11. 10.5365/wpsar.2021.12.4.852
[1217] Wen X-S, Jiang D, Gao L, et al. (2021) Clinical characteristics and predictive value of lower CD4⁺T cell level in patients with moderate and severe COVID-19: a multicenter retrospective study. BMC Infect Dis 21(1): 57-57. 10.1186/s12879-020-05741-w
[1218] Abdalbary M, Kakani E, Ahmed Y, Shea M, Neyra JA, El-Husseini A (2021) Characteristics and outcomes of prisoners hospitalized due to COVID-19 disease. Clin Nephrol. 10.5414/cn110658
[1219] Nithya G, Lamech TM, Arumugam V, et al. (2021) A clinical study on the changing dynamics of disease severity, management strategies and outcomes of COVID-19 in patients requiring haemodialysis. J Nephrol 34(4): 999-1006. 10.1007/s40620-021-01072-4
[1220] Slusarczyk A, Tracz A, Gronkiewicz M, Jureczko L, Baczkowska T (2021) Outcomes of COVID-19 in hospitalized kidney and liver transplant recipients: a single-center experience. Polish Archives of Internal Medicine-Polskie Archiwum Medycyny Wewnetrznej 131(11). 10.20452/pamw.16070
[1221] Mashaqi S, Lee-Iannotti J, Rangan P, et al. (2021) Obstructive sleep apnea and COVID-19 clinical outcomes during hospitalization: a cohort study. J Clin Sleep Med 17(11): 2197-2204. 10.5664/jcsm.9424
[1222] Moll-Bernardes R, de Sousa AS, Macedo AVS, et al. (2021) IL-10 and IL-12 (P70) Levels Predict the Risk of Covid-19 Progression in Hypertensive Patients: Insights From the BRACE-CORONA Trial. Front Cardiovasc Med 8: 702507. 10.3389/fcvm.2021.702507
[1223] Mohamed NE, Benn EK, Astha V, et al. (2021) COVID-19 in patients with and without cancer: Examining differences in patient characteristics and outcomes. J Cancer Biol 2(1): 25-32. 10.46439/cancerbiology.2.019
[1224] Martínez-Vega RA, Jing W, Ortega-Villa AM, et al. (2021) Risk Prediction of Death in Inpatient Adults With COVID-19 from Mexico. Res Sq. 10.21203/rs.3.rs-996535/v1
[1225] Nematshahi M, Neamatshahi M, attarian f, Rahimi F, Soroosh D (2021) Risk Factors for Readmission in the Patients with COVID-19 Admitted to Hospital. ResearchSquare. 10.21203/rs.3.rs-646002/v1
[1226] Andrew A, Ming Lai K, Timothy B, Alyssa V, Shin M (2021) Unusually high risks of COVID-19 mortality with age-related comorbidities: An adjusted method to improve the risk assessment of mortality using the comorbid mortality data. medRxiv. 10.1101/2021.05.20.21257550

[1227] Leutner M, Kaleta M, Bellach L, et al. (2021) Insulin as Monotherapy and in Combination with Other Glucose-Lowering Drugs Is Related to Increased Risk of Diagnosis of Pneumonia: A Longitudinal Assessment over Two Years. Journal of Personalized Medicine 11(10). 10.3390/jpm11100984
[1228] Munblit D, Nekliudov NA, Bugaeva P, et al. (2021) Stop COVID Cohort: An Observational Study of 3480 Patients Admitted to the Sechenov University Hospital Network in Moscow City for Suspected Coronavirus Disease 2019 (COVID-19) Infection. Clin Infect Dis 73(1): 1-11. 10.1093/cid/ciaa1535
[1229] Quintero SC, Martínez MS, Garrido ABC (2022) [Risk factors for severity and mortality in adults testing positive for COVID-19 in the IV Health Area of Albacete]. Enferm Clin. 10.1016/j.enfcli.2022.01.005
[1230] Ko JY, Danielson ML, Town M, et al. (2021) Risk Factors for Coronavirus Disease 2019 (COVID-19)-Associated Hospitalization: COVID-19-Associated Hospitalization Surveillance Network and Behavioral Risk Factor Surveillance System. Clin Infect Dis 72(11): e695-e703. 10.1093/cid/ciaa1419
[1231] Espiritu AI, Chiu HHC, Sy MCC, Anlacan VMM, Jamora RDG (2021) The outcomes of patients with diabetes mellitus in The Philippine CORONA Study. Sci Rep 11(1): 24436. 10.1038/s41598-021-03898-1
[1232] Valverde-López F, Tendero-Peinado C, Lecuona-Muñoz M, et al. (2021) A Gastroenterologist' clinical experience in COVID 19 and in-hospital mortality and length of stay analysis. Postgrad Med 133(6): 592-598. 10.1080/00325481.2021.1949200
[1233] 周岐龙 卢熊刘潘 (2021) Analysis on clinical characteristics and prognosis in 248 cases of COVID-19 in Chongqing city. Journal of Modern Medicine & Health 37(15): 2559-2564. 10.3969/j.issn.1009-5519.2021.15.010
[1234] van Son J, Oussaada SM, Şekercan A, et al. (2021) Overweight and Obesity Are Associated With Acute Kidney Injury and Acute Respiratory Distress Syndrome, but Not With Increased Mortality in Hospitalized COVID-19 Patients: A Retrospective Cohort Study. Front Endocrinol (Lausanne) 12: 747732. 10.3389/fendo.2021.747732
[1235] (2021) Tuberculosis and COVID-19 co-infection: description of the global cohort. Eur Respir J. 10.1183/13993003.02538-2021
[1236] Sutiningsih D, Azzahra NA, Prabowo Y, et al. (2021) COVID-19 deaths and associated demographic factors in Central Java, Indonesia. Germs 11(2): 255-265. 10.18683/germs.2021.1262
[1237] Zolnikova OYDNL, Potskherashvili ND, Puzakov KB, et al. (2020) Clinical Traits of SARS-CoV-2 Infection. Russian Journal of Gastroenterology, Hepatology, Coloproctology 30(6): 28-39. 10.22416/1382-4376-2020-30-6-28-39
[1238] 覃伟, 胡冰竹, 张竹, et al. (2020) Clinical Characteristics and Death Risk Factors of Severe COVID-19. 中华结核和呼吸杂志. 10.3760/cma.j.cn112147-20200320-00380
[1239] Sahin I, Haymana C, Demir T, et al. (2022) Clinical Characteristics and Outcomes of COVID-19 Patients with Overweight and Obesity: Turkish Nationwide Cohort Study (TurCObesity). Exp Clin Endocrinol Diabetes 130(2): 115-124. 10.1055/a-1552-4449
[1240] Aggarwal A, Shrivastava A, Kumar A, Ali A (2020) Clinical and Epidemiological Features of SARS-CoV-2 Patients in SARI Ward of a Tertiary Care Centre in New Delhi. J Assoc Physicians India 68(7): 19-26
[1241] Alkhouli M, Nanjundappa A, Annie F, Bates MC, Bhatt DL (2020) Sex Differences in Case Fatality Rate of COVID-19: Insights From a Multinational Registry. Mayo Clin Proc 95(8): 1613-1620. 10.1016/j.mayocp.2020.05.014
[1242] Barone MTU, Harnik SB, de Luca PV, et al. (2020) The impact of COVID-19 on people with diabetes in Brazil. Diabetes Res Clin Pract 166: 108304. 10.1016/j.diabres.2020.108304
[1243] Barone MTU, Villarroel D, de Luca PV, et al. (2020) COVID-19 impact on people with diabetes in South and Central America (SACA region). Diabetes Res Clin Pract 166: 108301. 10.1016/j.diabres.2020.108301
[1244] Deiana G, Azara A, Dettori M, et al. (2020) Deaths in SARS-Cov-2 Positive Patients in Italy: The Influence of Underlying Health Conditions on Lethality. Int J Environ Res Public Health 17(12). 10.3390/ijerph17124450
[1245] Joseph VT, Nevan M, Bethan MP, et al. (2020) Patient characteristics and predictors of mortality in 470 adults admitted to a district general hospital in England with Covid-19. medRxiv. 10.1101/2020.07.21.20153650
[1246] Kalyanaraman Marcello R, Dolle J, Grami S, et al. (2020) Characteristics and Outcomes of COVID-19 Patients in New York City's Public Hospital System. medRxiv. 10.1101/2020.05.29.20086645

[1247] Karagiannidis C, Mostert C, Hentschker C, et al. (2020) Case characteristics, resource use, and outcomes of 10 021 patients with COVID-19 admitted to 920 German hospitals: an observational study. Lancet Respir Med. 10.1016/s2213-2600(20)30316-7
[1248] Omrani AS, Almaslamani MA, Daghfal J, et al. (2020) The first consecutive 5000 patients with Coronavirus Disease 2019 from Qatar; a nation-wide cohort study. BMC Infect Dis 20(1): 777. 10.1186/s12879-020-05511-8
[1249] Koc M, Sumbul HE, Gulumsek E, et al. (2020) Disease Severity Affects Ventricular Repolarization Parameters in Patients With COVID-19. Arq Bras Cardiol 115(5): 907-913. 10.36660/abc.20200482
[1250] Majeed J, Ajmera P, Goyal RK (2020) Delineating clinical characteristics and comorbidities among 206 COVID-19deceasedpatients in India: Emerging significance of renin angiotensin system derangement. Diabetes Res Clin Pract 167: 108349. 10.1016/j.diabres.2020.108349
[1251] Mannucci E, Nreu B, Monami M (2020) Factors associated with increased all-cause mortality during the COVID-19 pandemic in Italy. Int J Infect Dis 98: 121-124. 10.1016/j.ijid.2020.06.077
[1252] Gao F, Zheng KI, Wang XB, et al. (2020) Obesity Is a Risk Factor for Greater COVID-19 Severity. Diabetes Care. 10.2337/dc20-0682
[1253] Lian J, Jin X, Hao S, et al. (2020) Epidemiological, clinical, and virological characteristics of 465 hospitalized cases of coronavirus disease 2019 (COVID-19) from Zhejiang province in China.
[1254] Liang W-H, Guan W-J, Li C-C, et al. (2020) Clinical characteristics and outcomes of hospitalised patients with COVID-19 treated in Hubei (epicentre) and outside Hubei (non-epicentre): a nationwide analysis of China. Eur respir j 55(6)
[1255] Nikpouraghdam M, Jalali Farahani A, Alishiri G, et al. (2020) Epidemiological characteristics of coronavirus disease 2019 (COVID-19) patients in IRAN: A single center study. J Clin Virol 127: 104378-104378
[1256] Price-Haywood EG, Burton J, Fort D, Seoane L (2020) Hospitalization and Mortality among Black Patients and White Patients with Covid-19. N Engl j med
[1257] Peter GvdV, Miquelle M, Boukje C, Marcel D (2020) Pre-outbreak determinants of perceived risks of corona infection and preventive measures taken. A prospective population-based study. medRxiv. 10.1101/2020.04.01.20049957
[1258] Ahrenfeldt L, Nielsen C, Möller S, Christensen K, Lindahl-Jacobsen R (2020) Burden and prevalence of risk factors for severe COVID-19 disease in the ageing European population – A SHARE-based analysis. ResearchSquare. 10.21203/rs.3.rs-73657/v1
[1259] Ajay B, Dr. Balamurugan G, Dr. Abhijit Sanjay S, Dr. Amarjeet Ekamaresh T, Dr. Girish Davinder B, Dr. Eham Lalit A (2020) The Impact of Co-Morbidities on Outcomes in Patients Suffering from COVID-19 – A Retrospective Study of 1393 Cases in a Tertiary Care Centre in Mumbai, India. SSRN. 10.2139/ssrn.3717560
[1260] Akbariqomi M, Hosseini MS, Rashidiani J, et al. (2020) Clinical characteristics and outcome of hospitalized COVID-19 patients with diabetes: A single-center, retrospective study in Iran. Diabetes Res Clin Pract 169: 108467. 10.1016/j.diabres.2020.108467
[1261] Akkus C, Yilmaz H, Mizrak S, Adibelli Z, Akdas O, Duran C (2020) Development of pancreatic injuries in the course of COVID-19. Acta Gastroenterol Belg 83(4): 585-592
[1262] Akter F, Mannan A, Mehedi HMH, et al. (2020) Clinical characteristics and short term outcomes after recovery from COVID-19 in patients with and without diabetes in Bangladesh. Diabetes Metab Syndr 14(6): 2031-2038. 10.1016/j.dsx.2020.10.016
[1263] Al Hayek AA, Robert AA, Alotaibi ZK, Al Dawish M (2020) Clinical characteristics of hospitalized and home isolated COVID-19 patients with type 1 diabetes. Diabetes Metab Syndr 14(6): 1841-1845. 10.1016/j.dsx.2020.09.013
[1264] Alguwaihes AM, Al-Sofiani ME, Megdad M, et al. (2020) Diabetes and Covid-19 among hospitalized patients in Saudi Arabia: a single-centre retrospective study. Cardiovasc Diabetol 19(1): 205. 10.1186/s12933-020-01184-4
[1265] Alkundi A, Mahmoud I, Musa A, Naveed S, Alshawwaf M (2020) Clinical characteristics and outcomes of COVID-19 hospitalized patients with diabetes in the United Kingdom: A retrospective single centre study. Diabetes Research & Clinical Practice 165. 10.1016/j.diabres.2020.108263
[1266] Altonen BL, Arreglado TM, Leroux O, Murray-Ramcharan M, Engdahl R (2020) Characteristics, comorbidities and survival analysis of young adults hospitalized with COVID-19 in New York City. PLoS One 15(12): e0243343-e0243343
[1267] Alvarez-Lopez DI, Espinoza-Molina MP, Cruz-Loustaunau ID, Alvarez-Hernandez G (2020) Diabetes and hypertension as factors associated with fatality from Covid-19 in Sonora, Mexico, 2020. Salud Publica De Mexico 62(5): 456-457. 10.21149/11546
[1268] Al-Wahaibi K, Al-Wahshi Y, Mohamed Elfadil O (2020) Myocardial Injury Is Associated with Higher Morbidity and Mortality in Patients with 2019 Novel Coronavirus Disease (COVID-19). SN Compr Clin Med: 1-7. 10.1007/s42399-020-00569-6

[1269] Alzaid F, Julla JB, Diedisheim M, et al. (2020) Monocytopenia, monocyte morphological anomalies and hyperinflammation characterise severe COVID-19 in type 2 diabetes. EMBO Mol Med 12(10): e13038. 10.15252/emmm.202013038
[1270] Amadu I, Ahinkorah BO, Afitiri AR, et al. (2021) Assessing sub-regional-specific strengths of healthcare systems associated with COVID-19 prevalence, deaths and recoveries in Africa. PLoS One 16(3): e0247274. 10.1371/journal.pone.0247274
[1271] Ana O-G, Erwin C, Jesus A-D, et al. (2020) Sex Differences in the Outcome of Patients with Confirmed SARS-CoV-2 Infection. SSRN. 10.2139/ssrn.3624222
[1272] Anurag A, Jha PK, Kumar A (2020) Differential white blood cell count in the COVID-19: A cross-sectional study of 148 patients. Diabetes Metab Syndr 14(6): 2099-2102. 10.1016/j.dsx.2020.10.029
[1273] Arasteh K (2020) Prevalence of Comorbidities and Risks Associated with COVID-19 Among Black and Hispanic Populations in New York City: an Examination of the 2018 New York City Community Health Survey. J Racial Ethn Health Disparities: 1-7. 10.1007/s40615-020-00844-1
[1274] Arellano-Llamas AA, HernÁNdez-Caballero Á (2020) COVID-19 en niños y adolescentes mexicanos hasta el 10 de mayo de 2020. Enfoque en pacientes con diabetes. Revista Mexicana de Endocrinología, Metabolismo y Nutrición 7(2): 80-86. 10.24875/RME.20000055
[1275] Ashinyo ME, Duti V, Dubik SD, et al. (2020) Clinical characteristics, treatment regimen and duration of hospitalization among COVID-19 patients in Ghana: a retrospective cohort study. The Pan African medical journal 37(Suppl 1): 9. 10.11604/PAMJ.SUPP.2020.37.9.25718
[1276] Azhar D, Raida F, Oksana Y, et al. (2020) Clinical Characteristics and Risk Factors for Disease Severity and Mortality of COVID-19 Patients with Diabetes Mellitus in Kazakhstan: A Nationwide Study. SSRN. 10.2139/ssrn.3727953
[1277] Badrah M, Riad A, Kassem I, Boccuzzi M, Klugar M (2021) Craniofacial pain in COVID-19 patients with diabetes mellitus: Clinical and laboratory description of 21 cases. J Med Virol 93(5): 2616-2619. 10.1002/jmv.26866
[1278] Bandar A, Maaweya H, Haitham A, et al. (2020) Influenza co-infection associated with severity and mortality in COVID-19 patients. 10.22541/AU.159985934.49280104
[1279] Bandopadhyay S, Sarkar S, Das D, et al. (2020) Clinical characteristics of hospitalized patients with 2019 novel coronavirus infection in tertiary care centres of three states of India. Journal of the Indian Medical Association 118(5)
[1280] Basit KA, Zafar AB, Fawwad A, Waris N, Shaheen F, Basit A (2021) Psychometric Analysis for fear of COVID-19 Scale (FCV-19S) and its association with depression in patients with diabetes: A cross sectional study from a Tertiary Care Centre in Karachi, Pakistan. Diabetes Metab Syndr 15(3): 733-737. 10.1016/j.dsx.2021.03.008
[1281] Best JH, Mohan SV, Kong AM, et al. (2020) Baseline Demographics and Clinical Characteristics Among 3471 US Patients Hospitalized with COVID-19 and Pulmonary Involvement: A Retrospective Study. Adv Ther 37(12): 4981-4995. 10.1007/s12325-020-01510-y
[1282] Beurnier A, Jutant EM, Jevnikar M, et al. (2020) Characteristics and outcomes of asthmatic patients with COVID-19 pneumonia who require hospitalisation. European Respiratory Journal 56(5). 10.1183/13993003.01875-2020
[1283] Bhandari S, Rankawat G, Singh A, Gupta V, Kakkar S (2020) Impact of glycemic control in diabetes mellitus on management of COVID-19 infection. Int J Diabetes Dev Ctries: 1-6. 10.1007/s13410-020-00868-7
[1284] Bhatti R, Khamis AH, Khatib S, Shiraz S, Matfin G (2020) Clinical Characteristics and Outcomes of Patients With Diabetes Admitted for COVID-19 Treatment in Dubai: Single-Centre Cross-Sectional Study. JMIR Public Health Surveill 6(4): e22471. 10.2196/22471
[1285] Biswas P, Das D, Pandey P, et al. (2020) Frequency of comorbidities & their association with intensive care unit admission in hospitalised patients with 2019 novel coronavirus infection in tertiary care centres of three states of India. Journal of the Indian Medical Association 118(6)
[1286] Boari GEM, Chiarini G, Bonetti S, et al. (2020) Prognostic factors and predictors of outcome in patients with COVID-19 and related pneumonia: a retrospective cohort study. Biosci Rep 40(12). 10.1042/bsr20203455
[1287] Boniface M, Betty Roberta N, John Jude Kweku A, Collins K (2021) Acute uncontrolled hyperglycemia, a non-specific presentation and predictor of covid-19 severity- a report of three cases. 6(1). 10.30574/GSCARR.2021.6.1.0001
[1288] Cai Y, Shi S, Yang F, et al. (2020) Fasting blood glucose level is a predictor of mortality in patients with COVID-19 independent of diabetes history. Diabetes Res Clin Pract 169: 108437. 10.1016/j.diabres.2020.108437
[1289] Capone S, Abramyan S, Ross B, et al. (2020) Characterization of Critically Ill COVID-19 Patients at a Brooklyn Safety-Net Hospital. Cureus 12(8): e9809. 10.7759/cureus.9809

[1290] Cardona-Hernandez R, Cherubini V, Iafusco D, Schiaffini R, Luo X, Maahs DM (2020) Children and youth with diabetes are not at increased risk for hospitalization due to COVID-19. Pediatr Diabetes. 10.1111/pedi.13158
[1291] Carrasco-Sánchez FJ, López-Carmona MD, Martínez-Marcos FJ, et al. (2020) Admission hyperglycemiaas a predictor of mortality in patients hospitalized with COVID-19 regardless of diabetes status: Data from the Spanish SEMI-COVID-19 Registry. Annals of medicine: 1-22. 10.1080/07853890.2020.1836566
[1292] Carrillo-Vega MF, Salinas-Escudero G, García-Peña C, Gutiérrez-Robledo LM, Parra-Rodríguez L (2020) Early estimation of the risk factors for hospitalization and mortality by COVID-19 in Mexico. PLoS One 15(9): e0238905. 10.1371/journal.pone.0238905
[1293] Chen Y, Chen J, Gong X, et al. (2020) Clinical Characteristics and Outcomes of Type 2 Diabetes Patients Infected with COVID-19: A Retrospective Study. Engineering (Beijing) 6(10): 1170-1177. 10.1016/j.eng.2020.05.017
[1294] Chen Z, Zhang F, Hu W, et al. (2020) Laboratory markers associated with COVID-19 progression in patients with or without comorbidity: A retrospective study. J Clin Lab Anal: e23644. 10.1002/jcla.23644
[1295] Clift AK, Coupland CAC, Keogh RH, et al. (2020) Living risk prediction algorithm (QCOVID) for risk of hospital admission and mortality from coronavirus 19 in adults: national derivation and validation cohort study. Bmj 371: m3731. 10.1136/bmj.m3731
[1296] Conway J, Gould A, Westley R, et al. (2020) Characteristics of patients with diabetes hospitalised for COVID-19 infection-a brief case series report. Diabetes Res Clin Pract 169: 108460. 10.1016/j.diabres.2020.108460
[1297] Corcillo A, Cohen S, Game D, Karalliedde J (2020) High prevalence of Afro-Caribbean ethnicity and hypoglycaemia in patients with diabetes and end stage renal disease hospitalized with COVID-19. Nephrology (Carlton). 10.1111/nep.13809
[1298] Croft A, Bucca A, Jansen JH, et al. (2020) First-time Diabetic Ketoacidosis in Type 2 Diabetics With Covid-19 Infection: A Novel Case Series. J Emerg Med 59(5): e193-e197. 10.1016/j.jemermed.2020.07.017
[1299] De Luca G, Cercek M, Jensen LO, et al. (2020) Impact of COVID-19 pandemic and diabetes on mechanical reperfusion in patients with STEMI: insights from the ISACS STEMI COVID 19 Registry. Cardiovasc Diabetol 19(1): 215. 10.1186/s12933-020-01196-0
[1300] Dhont S, Callens R, Stevens D, et al. (2020) Myotonic dystrophy type 1 as a major risk factor for severe COVID-19? Acta Neurol Belg: 1-5. 10.1007/s13760-020-01514-z
[1301] Edagawa S, Kobayashi F, Kodama F, et al. (2020) Epidemiological features after emergency declaration in Hokkaido and report of 15 cases of COVID-19 including 3 cases requiring mechanical ventilation. Glob Health Med 2(2): 112-117. 10.35772/ghm.2020.01024
[1302] Elamari S, Motaib I, Zbiri S, Elaidaoui K, Chadli A, Elkettani C (2020) Characteristics and outcomes of diabetic patients infected by the SARS-CoV-2. Pan Afr Med J 37: 32. 10.11604/pamj.2020.37.32.25192
[1303] Emami A, Javanmardi F, Akbari A, et al. (2020) Characteristics of deceased patients with CoVID-19 after the first peak of the epidemic in Fars province, Iran. Infect Ecol Epidemiol 10(1): 1781330. 10.1080/20008686.2020.1781330
[1304] Estedlal A, Jeddi M, Heydari S, Dabbaghmanesh M (2020) The Association between Poor Glycemic Control in Diabetes Mellitus and Progression of COVID-19. ResearchSquare. 10.21203/rs.3.rs-107735/v1
[1305] Farhana A, Adnan M, Mehedi HMH, et al. (2020) Clinical manifestations along with biochemical and psychological outcomes of COVID-19 cases in diabetic individuals in Bangladesh. medRxiv. 10.1101/2020.09.24.20200790
[1306] Fawaz A, Jean-Baptiste J, Marc D, et al. (2020) Monocyte class switch and hyperinflammation characterise severe COVID-19 in type 2 diabetes. medRxiv. 10.1101/2020.06.02.20119909
[1307] Fayol A, Livrozet M, Boutouyrie P, et al. (2021) Cardiac performance in patients hospitalized with COVID-19: a 6 month follow-up study. ESC Heart Fail. 10.1002/ehf2.13315
[1308] Fengxue Z (2020) Glycosylated hemoglobin is associated withsystemic inflammation, hypercoagulability, andprognosis of COVID-19 patients- Fengxue Zhu - Peking University People's Hospital, Beijing , China. 4(2)
[1309] Francisco R-S, Ana B-A, Rossana T-A, et al. (2021) Fraction of COVID-19 hospitalizations and deaths attributable to chronic diseases. medRxiv. 10.1101/2021.04.12.21255346
[1310] Galvez-Romero JL, Palmeros-Rojas O, Real-Ramirez FA, et al. Cyclosporine A plus low-dose steroid treatment in COVID-19 improves clinical outcomes in patients with moderate to severe disease: A pilot study. Journal of Internal Medicine. 10.1111/joim.13223
[1311] Gao YC, Liu T, Zhong WJ, et al. (2020) Risk of Metformin in Patients With Type 2 Diabetes With COVID-19: A Preliminary Retrospective Report. Cts-Clinical and Translational Science 13(6): 1055-1059. 10.1111/cts.12897

[1312] Gou L, Xiang M, Ran X, et al. (2021) Hyperosmolarity Deserves More Attention in Critically Ill COVID-19 Patients with Diabetes: A Cohort-Based Study. Diabetes Metab Syndr Obes 14: 47-58. 10.2147/dmso.S284148
[1313] Hachim MY, Hachim IY, Naeem KB, Hannawi H, Salmi IA, Hannawi S (2020) D-dimer, Troponin, and Urea Level at Presentation With COVID-19 can Predict ICU Admission: A Single Centered Study. Front Med (Lausanne) 7: 585003. 10.3389/fmed.2020.585003
[1314] Han M, Ma K, Wang X, et al. (2021) Immunological Characteristics in Type 2 Diabetes Mellitus Among COVID-19 Patients. Front Endocrinol (Lausanne) 12: 596518. 10.3389/fendo.2021.596518
[1315] Hu W, Luo S, Lu Z, et al. (2020) Diabetes patients with comorbidities had unfavorable outcomes following COVID-19: a retrospective study. ResearchSquare. 10.21203/rs.3.rs-57456/v2
[1316] Imran MM, Ahmed U, Usman U, Ali M, Shaukat A, Gul N (2020) Neutrophil/Lymphocyte Ratio - A Marker of COVID-19 Pneumonia Severity. Int J Clin Pract: e13698. 10.1111/ijcp.13698
[1317] Inass S, Khaled Ahmed A, Alia S, Hassan S, Mazen Ahmed A (2020) The Psychological Impact of the COVID-19 Pandemic on Egyptian Diabetic Patients. 10.22541/AU.160239783.34402594/V1
[1318] Jacob Allan U, Bahar B, Atul S, et al. (2021) Clinical Risk, Sociodemographic Factors, and SARS-CoV-2 Infection Over Time in Ontario, Canada. medRxiv. 10.1101/2021.04.28.21256052
[1319] Jacob L, Rickwood S, Rathmann W, Kostev K (2021) Change in glucose-lowering medication regimens in individuals with type 2 diabetes mellitus during the COVID-19 pandemic in Germany. Diabetes Obesity & Metabolism 23(4): 910-915. 10.1111/dom.14293
[1320] Jesus Arturo R-Q, Sr., Crystell G-P, Sr., German Alberto N-R, Sr., et al. (2020) Features of patients that died for COVID-19 in a Hospital in the south of Mexico: A observational cohort study. medRxiv. 10.1101/2020.09.21.20199117
[1321] Jin-Kui Y, Jian-Min J, Shi L, et al. (2020) New onset COVID-19-related diabetes: an indicator of mortality. medRxiv. 10.1101/2020.04.08.20058040
[1322] Jurado A, Martín MC, Abad-Molina C, et al. (2020) COVID-19: age, Interleukin-6, C-reactive protein, and lymphocytes as key clues from a multicentre retrospective study. Immun Ageing 17: 22. 10.1186/s12979-020-00194-w
[1323] Kamioka H, Mori Y, Horiuchi T, et al. (2020) Association of Daily Home-Based Hot Water Bathing and Glycemic Control in Ambulatory Japanese Patients with Type 2 Diabetes Mellitus During the COVID-19 Pandemic: A Multicenter Cross-Sectional Study. Diabetes Metab Syndr Obes 13: 5059-5069. 10.2147/dmso.S279270
[1324] Karatas S, Yesim T, Beysel S (2021) Impact of lockdown COVID-19 on metabolic control in type 2 diabetes mellitus and healthy people. Prim Care Diabetes. 10.1016/j.pcd.2021.01.003
[1325] Koufakis T, Metallidis S, Zebekakis P, Kotsa K (2021) Intestinal SGLT1 as a therapeutic target in COVID-19-related diabetes: A "two-edged sword" hypothesis. Br J Clin Pharmacol. 10.1111/bcp.14800
[1326] Koufakis T, Pavlidis AN, Metallidis S, Kotsa K (2021) Sodium-glucose co-transporter 2 inhibitors in COVID-19: meeting at the crossroads between heart, diabetes and infectious diseases. Int J Clin Pharm: 1-4. 10.1007/s11096-021-01256-9
[1327] Laires PA, Nunes C (2020) Population-based Estimates or High Risk of Severe COVID-19 Disease due to Age and Underlying Health Conditions. Acta Medica Portuguesa 33(11): 720-725. 10.20344/amp.14222
[1328] Li YN, Li M, Wang MD, et al. (2020) Acute cerebrovascular disease following COVID-19: a single center, retrospective, observational study. Stroke and Vascular Neurology 5(3): 279-284. 10.1136/svn-2020-000431
[1329] Liu D, Wang Y, Lan L, et al. (2020) Overall reduced lymphocyte subsets worsening disease severity and prognosis in COVID-19 severe cases with diabetes mellitus in Chengdu, China. ResearchSquare. 10.21203/rs.3.rs-20385/v1
[1330] Lockhart SM, Lockhart SM, Calvo-Latorre J, et al. (2021) The excess insulin requirement in severe COVID-19 compared to non-COVID-19 viral pneumonitis is related to the severity of respiratory failure and pre-existing diabetes. Endocrinol Diabetes Metab. 10.1002/edm2.228
[1331] Lorincz-Comi N, Zhu X (2021) Cardiometabolic risks of SARS-CoV-2 hospitalization using Mendelian Randomization. Sci Rep 11(1): 7848. 10.1038/s41598-021-86757-3
[1332] Maddaloni E, D'Onofrio L, Alessandri F, et al. (2020) Cardiometabolic multimorbidity is associated with a worse Covid-19 prognosis than individual cardiometabolic risk factors: a multicentre retrospective study (CoViDiab II). Cardiovascular Diabetology 19(1). 10.1186/s12933-020-01140-2
[1333] Mansour A, Sajjadi-Jazi SM, Kasaeian A, et al. (2020) Clinical characteristics and outcomes of diabetics hospitalized for COVID-19 infection: a single-centered, retrospective, observational study. Excli j 19: 1533-1543. 10.17179/excli2020-2988
[1334] Masuda M, Tomonaga O (2021) Study on the effects of changes in lifestyle of patients with diabetes on glycaemic control before and after the declaration of the state of emergency in Japan. Diabetol Int: 1-9. 10.1007/s13340-021-00505-6

[1335] Min Hyuk C, Hyunmin A, Han Seok R, et al. (2020) Clinical Characteristics and Disease Progression in Early-Stage COVID-19 Patients in South Korea: The Retrospective Cohort Study. SSRN. 10.2139/ssrn.3582818
[1336] Mingyang W, shuqiong H, Jun L, et al. (2020) Association between comorbidities and the risk of death in patients with COVID-19: sex-specific differences. medRxiv. 10.1101/2020.05.22.20109579
[1337] Mishra Y, Pathak BK, Mohakuda SS, et al. (2020) Relation of D-dimer levels of COVID-19 patients with diabetes mellitus. Diabetes Metab Syndr 14(6): 1927-1930. 10.1016/j.dsx.2020.09.035
[1338] Monterrubio-Flores E, Ramírez-Villalobos M, Espinoza-Montero J, et al. (2020) Impact of a Double Epidemic in Mexico: Non-Communicable Diseases Increase the Case Fatality Rate with Covid-19. ResearchSquare. 10.21203/rs.3.rs-80669/v1
[1339] Musche V, Kohler H, Bäuerle A, et al. (2021) COVID-19-Related Fear, Risk Perception, and Safety Behavior in Individuals with Diabetes. Healthcare (Basel) 9(4). 10.3390/healthcare9040480
[1340] Nafakhi H, Alareedh M, Al-Buthabhak K, Shaghee F, Nafakhi A, Kasim S (2020) Predictors of adverse in-hospital outcome and recovery in patients with diabetes mellitus and COVID-19 pneumonia in Iraq. Diabetes Metab Syndr 15(1): 33-38. 10.1016/j.dsx.2020.12.014
[1341] Oley MH, Oley MC, Kepel BJ, et al. (2021) ICAM-1 levels in patients with covid-19 with diabetic foot ulcers: A prospective study in southeast asia. Ann Med Surg (Lond) 63: 102171. 10.1016/j.amsu.2021.02.017
[1342] Rahila B, Amar O, Samara K, Seemin S, Glenn M (2020) Clinical characteristics and outcomes in diabetes patients admitted with COVID-19 in Dubai: a cross-sectional single centre study. medRxiv. 10.1101/2020.07.08.20149096
[1343] Ricchio M, Tassone B, Pelle M, et al. (2020) Characteristics, Management, and Outcomes of Elderly Patients With Diabetes in a COVID-19 Unit: Lessons Learned. ResearchSquare. 10.21203/rs.3.rs-80915/v1
[1344] Saha A, Ahsan MM, Quader MT, et al. (2021) Clinical characteristics and outcomes of COVID-19 infected diabetic patients admitted in ICUs of the southern region of Bangladesh. Diabetes Metab Syndr 15(1): 229-235. 10.1016/j.dsx.2020.12.037
[1345] Saqib A, Solanki P, Carroll M, Gough A, Oguntolu V (2020) Impact of COVID-19 on glycaemic control: a retrospective cohort study in a local district general hospital. Practical Diabetes 37(5): 164-165. 10.1002/pdi.2293
[1346] Seclen SN, Nunez-Robles E, Yovera-Aldana M, Arias-Chumpitaz A (2020) Incidence of COVID-19 infection and prevalence of diabetes, obesity and hypertension according to altitude in Peruvian population. Diabetes Research and Clinical Practice 169. 10.1016/j.diabres.2020.108463
[1347] Seligman B, Ferranna M, Bloom DE (2021) Social determinants of mortality from COVID-19: A simulation study using NHANES. Plos Medicine 18(1). 10.1371/journal.pmed.1003490
[1348] Soliman AT, Prabhakaran Nair A, Al Masalamani MS, et al. (2020) Prevalence, clinical manifestations, and biochemical data of type 2 diabetes mellitus versus nondiabetic symptomatic patients with COVID-19: A comparative study. Acta Biomed 91(3): e2020010. 10.23750/abm.v91i3.10214
[1349] Sosa-Rubí SG, Seiglie JA, Chivardi C, et al. (2020) Incremental Risk of Developing Severe COVID-19 Among Mexican Patients With Diabetes Attributed to Social and Health Care Access Disadvantages. Diabetes Care. 10.2337/dc20-2192
[1350] Su MH, Yuan J, Peng JR, Wu MJ, Yang YS, Peng YG (2020) Clinical prediction model for mortality of adult diabetes inpatients with COVID-19 in Wuhan, China: A retrospective pilot study. Journal of Clinical Anesthesia 66. 10.1016/j.jclinane.2020.109927
[1351] Taye GM, Bose L, Beressa TB, et al. (2020) COVID-19 Knowledge, Attitudes, and Prevention Practices Among People with Hypertension and Diabetes Mellitus Attending Public Health Facilities in Ambo, Ethiopia. Infection and Drug Resistance 13: 4203-4214. 10.2147/idr.S283999
[1352] Tee LY, Alhamid SM, Tan JL, et al. (2020) COVID-19 and Undiagnosed Pre-diabetes or Diabetes Mellitus Among International Migrant Workers in Singapore. Frontiers in Public Health 8. 10.3389/fpubh.2020.584249
[1353] Vamvini M, Lioutas V-A, Middelbeek RJW (2020) Characteristics and Diabetes Control in Adults With Type 1 Diabetes Admitted With COVID-19 Infection. Diabetes Care 43(10): e120-e122
[1354] Wu LD, Girgis CM, Cheung NW (2020) COVID-19 and diabetes: Insulin requirements parallel illness severity in critically unwell patients. Clinical Endocrinology 93(4): 390-393. 10.1111/cen.14288
[1355] Yan AF, Sun XM, Zheng JG, et al. (2020) Perceived risk, behavior changes and Health-related outcomes during COVID-19 pandemic: Findings among adults with and without diabetes in China. Diabetes Research and Clinical Practice 167. 10.1016/j.diabres.2020.108350
[1356] Yang G, Shengzhi S, Ye S (2020) Estimation of case-fatality rate in COVID-19 patients with hypertension and diabetes mellitus in the New York State. medRxiv. 10.1101/2020.08.20.20178962
[1357] Yates T, Zaccardi F, Islam N, et al. (2021) Obesity, ethnicity and risk of critical care, mechanical ventilation and mortality in patients admitted to hospital with COVID-19: Analysis of the ISARIC CCP-UK cohort. Obesity (Silver Spring). 10.1002/oby.23178

[1358] Yuan S, Li H, Chen C, Wang F, Wang DW (2021) Association of glycosylated haemoglobin HbA1c levels with outcome in patients with COVID-19: A Retrospective Study. J Cell Mol Med 25(7): 3484-3497. 10.1111/jcmm.16431
[1359] Yuli C, Shaobo S, Fan Y, et al. (2020) Fasting Glucose Level Is a Predictor of Mortality in Patients with COVID-19 Independent of Diabetes History. SSRN. 10.2139/ssrn.3605224
[1360] Zhang Y, Wang J, Tan N, et al. (2021) Risk Factors in Patients with Diabetes Hospitalized for COVID-19: Findings from a Multicenter Retrospective Study. J Diabetes Res 2021: 3170190. 10.1155/2021/3170190
[1361] Zhao R, Sun Y, Zhang Y, et al. (2020) Distinguishable Immunologic Characteristics of COVID-19 Patients with Comorbid Type 2 Diabetes Compared with Nondiabetic Individuals. Mediators Inflamm 2020: 6914878. 10.1155/2020/6914878
[1362] Zhao Y, Liu Y, Yi F, et al. (2021) Type 2 diabetes mellitus impaired nasal immunity and increased the risk of hyposmia in COVID-19 mild pneumonia patients. Int Immunopharmacol 93: 107406. 10.1016/j.intimp.2021.107406
[1363] Zhou W, Ye S, Wang W, Li S, Hu Q (2020) Clinical Features of COVID-19 Patients with Diabetes and Secondary Hyperglycemia. J Diabetes Res 2020: 3918723. 10.1155/2020/3918723
[1364] Zhou W, Ye SD, Wang W, Li SM, Hu QG (2020) Clinical Features of COVID-19 Patients with Diabetes and Secondary Hyperglycemia. Journal of Diabetes Research 2020. 10.1155/2020/3918723
[1365] 李丹, 何兵, 周晓阳, 陆文峰, 张洁 (2020) Analysis on 28 death cases of COVID-19/ 28例新型冠状病毒肺炎死亡病例分析. 10.12138/J.ISSN.1671-9638.20206860
[1366] Coqueiro JM (2020) Covid-19 and diabetes mellitus: an analysis of the videos released by Rede Globo de Televisão. RECIIS (Online) 14(4): 843-856
[1367] Lahera T, Ruiz C, Aquevedo A, Cotoras P, Uribe J, Montenegro C (2020) [Features of 50 patients with SARS-CoV2 admitted to an intensive care unit and requiring mechanical ventilation]. Rev Med Chil 148(12): 1725-1733. 10.4067/s0034-98872020001201725
[1368] Marin-Sanchez A (2020) Basic clinical characteristics in the first 100 fatal cases of COVID-19 in Colombia. Revista Panamericana De Salud Publica-Pan American Journal of Public Health 44. 10.26633/rpsp.2020.87
[1369] Molochkov AV, Karateev DE, Ogneva EY, et al. (2020) Comorbidities and predicting the outcome of COVID-19: the treatment results of 13,585 patients hospitalized in the Moscow Region. Almanac of Clinical Medicine 48. 10.18786/2072-0505-2020-48-040
[1370] Profili F, Ballo P, Balzi D, et al. (2020) [Chronic diseases and risk of symptomatic COVID-19: results of a case-population study on a sample of patients in the Local Health Unit 'Toscana Centro' (Tuscany Region, Central Italy)]. Epidemiol Prev 44(5-6 Suppl 2): 308-314. 10.19191/ep20.5-6.S2.131
[1371] Sandra Patrícia de Oliveira V, Diógenes José Gusmão C (2020) COVID-19: A IMPORTÂNCIA DO AUTOCUIDADO APOIADO A PACIENTES COM DIABETES TIPO II, FAVORECIDO PALA EQUIPE DE APOIO MATRICIAL NASF-AB. 6(12). 10.29327/217514.6.12-30
[1372] Wang G, Wu C, Zhang Q, et al. (2020) Clinical characteristics and the risk factors for severe events of elderly coronavirus disease 2019 patients. Zhong nan da xue xue bao Yi xue ban = Journal of Central South University Medical sciences 45(5): 542-548. 10.11817/J.ISSN.1672-7347.2020.200292
[1373] Xiaoxu S, Fanghong S, Jing M, et al. (2020) Study on glycemic profiles and emotional scales in diabetic patients after the outbreak of COVID-19. Chinese Journal of Endocrinology and Metabolism 36(8). 10.3760/CMA.J.CN311282-20200611-00431
[1374] 杨艾利, 王晓光, 卫静, et al. (2020) Analysis of Influencing Factors of Blood Glucose Levels in Diabetic Patients during Epidemic of COVID-19/ 新型冠状病毒肺炎疫情期间糖尿病患者血糖水平的影响因素分析.(07)
[1375] Abdelghani M, Hamed MG, Said A, Fouad E (2022) Evaluation of perceived fears of COVID-19 virus infection and its relationship to health-related quality of life among patients with diabetes mellitus in Egypt during pandemic: a developing country single-center study. Diabetol Int 13(1): 108-116. 10.1007/s13340-021-00511-8
[1376] Abdelsamie AM, Abdelazim HM, Elnems MG, Abdelhakam RB, Abdelalim AA (2022) Covid-19-Related Acute Invasive Fungal Sinusitis: Clinical Features and Outcomes. Int Arch Otorhinolaryngol 26(1): e152-e157. 10.1055/s-0041-1740947
[1377] Abdleghani Y, Mbarek C (2020) Exploring Causal relationship between risk factors and vulnerability to COVID-19Cases of Italy, Spain, France, Greece, Portugal, Morocco and South Africa. In:
[1378] Aboumrad M, Shiner B, Riblet N, Huizenga H, Neupane N, Young-Xu Y (2021) Trends in COVID-19 cases and clinical management in Veterans Health Administration medical facilities: A national cohort study. PLoS One 16(7): e0246217. 10.1371/journal.pone.0246217

[1379] Adake PAAHSSPM (2022) Clinical Features of COVID-19 Patients with Preexisting Hypothyroidism: A Retrospective Study. Medical Laboratory Journal 16(1): 9-12. 10.29252/mlj.16.1.9
[1380] Ahrenfeldt LJ, Nielsen CR, Möller S, Christensen K, Lindahl-Jacobsen R (2020) Burden and prevalence of risk factors for severe COVID-19 disease in the ageing European population - A SHARE-based analysis. Res Sq. 10.21203/rs.3.rs-73657/v1
[1381] Alcántara-Alonso E, Molinar-Ramos F, González-López JA, et al. (2021) High triglyceride to HDL-cholesterol ratio as a biochemical marker of severe outcomes in COVID-19 patients. Clin Nutr ESPEN 44: 437-444. 10.1016/j.clnesp.2021.04.020
[1382] Ana Paula Andrade B, Lucimeire Cardoso D, Thiago C-S, et al. (2021) Post-Acute COVID Syndrome, the Aftermath of Mild to Severe COVID-19 in Brazilian Patients. medRxiv. 10.1101/2021.06.07.21258520
[1383] Anwar MZ, Masud R, Rafique I, et al. (2021) Impact of BMI on Covid-19 Clinical Features and its Management: A Cross Sectional Study. Pakistan Journal of Medical & Health Sciences 15(6): 1948-1951. 10.53350/pjmhs211561948
[1384] Areekal B, Sukumaran ST, Andrews AM, et al. (2021) Persistence of symptoms after acute COVID-19 infection- An experience from a tertiary care centre in South India. Journal of Clinical and Diagnostic Research 15(6): LC05-LC08. 10.7860/JCDR/2021/49600.14967
[1385] Arora U, Priyadarshi M, Katiyar V, et al. (2021) Novel risk factors for Coronavirus disease-associated mucormycosis(CAM): a case-control study during the outbreak in India. The Journal of infection. 10.1016/j.jinf.2021.12.039
[1386] Arturas Z (2022) Longitudinal symptom and clinical outcome analysis of hospitalized COVID-19 patients. medRxiv. 10.1101/2022.01.11.22268908
[1387] Avatef Fazeli M, Rezaei L, Javadirad E, et al. (2021) Increased incidence of rhino-orbital mucormycosis in an educational therapeutic hospital during the COVID-19 pandemic in western Iran: An observational study. Mycoses 64(11): 1366-1377. 10.1111/myc.13351
[1388] Balamanikandan P, Kumar SSPB, Bermio VS (2021) A study on the severity and outcome of covid 19 in obese patients. Journal of Advanced Medical and Dental Sciences Research 9(7): 45-50. 10.21276/jamdsr
[1389] Baldini M, Chiapella MN, Fernández A, Guardia S, Sala H (2021) Evaluation of the pulmonary function of patients with severe coronavirus 2019 disease three months after diagnosis. Medicina (B Aires) 81(5): 715-721
[1390] Banoei MM, Dinparastisaleh R, Zadeh AV, Mirsaeidi M (2021) Machine-learning-based COVID-19 mortality prediction model and identification of patients at low and high risk of dying. Crit Care 25(1): 328. 10.1186/s13054-021-03749-5
[1391] Barani S, Bhatnagar T, Natarajan M, et al. (2022) Health-related quality of life among COVID-19 individuals: A cross-sectional study in Tamil Nadu, India. Clin Epidemiol Glob Health 13: 100943. 10.1016/j.cegh.2021.100943
[1392] Barbhaya D, Franco S, Gandhi K, et al. (2021) Characteristics and Outcomes of COVID-19 Infection from an Urban Ambulatory COVID-19 Clinic-Guidance for Outpatient Clinicians in Triaging Patients. J Prim Care Community Health 12: 21501327211017016. 10.1177/21501327211017016
[1393] Barrett CE, Koyama AK, Alvarez P, et al. (2022) Risk for Newly Diagnosed Diabetes >30 Days After SARS-CoV-2 Infection Among Persons Aged <18 Years - United States, March 1, 2020-June 28, 2021. MMWR Morb Mortal Wkly Rep 71(2): 59-65. 10.15585/mmwr.mm7102e2
[1394] Baslılar S, Pehlivan O (2021) Evaluation of factors affecting the frequency and clinical course of COVID-19 in patients using anti-TNF-alpha agents. Rev Assoc Med Bras (1992) 67(9): 1286-1292. 10.1590/1806-9282.20210568
[1395] Behzadnia H, Omrani SN, Nozari-Golsefid H, et al. (2020) Ischemic stroke and intracerebral hemorrhage in patients with COVID-19. Romanian Journal of Neurology/ Revista Romana de Neurologie 19(3): 166-170. 10.37897/RJN.2020.3.5
[1396] Benites-Flores IR, Valdivia-Vega RP, Alcalde-Ruiz SF, Espinoza-Rojas HJ (2021) Clinical characteristics of acute kidney injury in the first 13 critically ill patients infected with SARS-CoV-2 (COVID-19) at a Peruvian hospital;a preliminary report. Journal of Nephropathology 10(2): 1-6. 10.34172/jnp.2021.15
[1397] Bhandari S, Bhargava S, Samdhani S, et al. (2021) COVID-19, Diabetes and Steroids: The Demonic Trident for Mucormycosis. Indian J Otolaryngol Head Neck Surg: 1-4. 10.1007/s12070-021-02883-4
[1398] Bhanumurthy LKP, Sekhar PRLM (2021) Post coronavirus disease mucormycosis involving the mandible: A case report with brief note on literature. Journal of Oral and Maxillofacial Pathology : JOMFP 25(3): 407-410. 10.4103/jomfp.jomfp_355_21
[1399] Bhargava D, Ahirwal R, Dubey S, et al. (2021) COVID Induced Functional Exhaustion and Persistently Reduced Lymphocytes as Vital Contributing Factors for Post-COVID Rhino-orbital and Cerebral Mucormycosis in Patients with Diabetes: Report from the Indian Sub-continent. Head Neck Pathol: 1-6. 10.1007/s12105-021-01382-w

[1400] Birabaharan M, Kaelber DC, Pettus JH, Smith DM (2022) Risk of New-Onset Type 2 Diabetes Mellitus in 600,055 Persons after COVID-19: a cohort study. Diabetes Obes Metab. 10.1111/dom.14659
[1401] Birtay T, Bahadir S, Kabacaoglu E, Yetiz O, Demirci MF, Genctoy G (2021) Prognosis of patients hospitalized with a diagnosis of COVID-19 pneumonia in a tertiary hospital in Turkey. Ann Saudi Med 41(6): 327-335. 10.5144/0256-4947.2021.327
[1402] Blanc F, Waechter C, Vogel T, et al. (2021) Therapeutic prevention of COVID-19 in elderly: a case-control study. Geroscience 43(5): 2333-2343. 10.1007/s11357-021-00397-z
[1403] Bonato M, Semenzato U, Tinè M, et al. (2021) Risk Factors for Development and Severity of COVID-19 in COPD Patients. Front Med (Lausanne) 8: 714570. 10.3389/fmed.2021.714570
[1404] Bouba Y, Tsinda EK, Fonkou MDM, Mmbando GS, Bragazzi NL, Kong JD (2021) The Determinants of the Low COVID-19 Transmission and Mortality Rates in Africa: A Cross-Country Analysis. Front Public Health 9: 751197. 10.3389/fpubh.2021.751197
[1405] Brito-Zerón P, Gracia-Tello B, Robles A, et al. (2021) Characterization and Outcomes of SARS-CoV-2 Infection in Patients with Sarcoidosis. Viruses 13(6). 10.3390/v13061000
[1406] Čapková R, Ševela S, Petrášková H, Ryšánková M, Brůha R (2021) ICU mortality of covid-19 patients - our experience. Vnitrni Lekarstvi 67(5): e23-e27. 10.36290/vnl.2021.084
[1407] Chandrasekaran ND, Velure Raja Rao MR, Sathish T (2021) Clinical characteristics and outcomes of COVID-19 patients with prediabetes. Diabetes Metab Syndr 15(4): 102192. 10.1016/j.dsx.2021.102192
[1408] Charfeddine S, Ibn Hadj Amor H, Jdidi J, et al. (2021) Long COVID 19 Syndrome: Is It Related to Microcirculation and Endothelial Dysfunction? Insights From TUN-EndCOV Study. Front Cardiovasc Med 8: 745758. 10.3389/fcvm.2021.745758
[1409] Chatterjee S, Datey A, Sengupta S, et al. (2021) Clinical, Virological, Immunological, and Genomic Characterization of Asymptomatic and Symptomatic Cases With SARS-CoV-2 Infection in India. Front Cell Infect Microbiol 11: 725035. 10.3389/fcimb.2021.725035
[1410] Chaturvedi H, Issac R, Sharma SK, Gupta R (2021) Progressive left and right heart dysfunction in coronavirus disease-19: Prospective echocardiographic evaluation. Eur Heart J Cardiovasc Imaging. 10.1093/ehjci/jeab268
[1411] Chavan RP, Ingole SM, Nazir HA, Desai WV, Kanchewad GS (2022) Mucormycosis in COVID-19 pandemic: study at tertiary hospital in India. Eur Arch Otorhinolaryngol: 1-10. 10.1007/s00405-022-07282-1
[1412] Chen CCWWX (2021) Investigation of the factors potentially responsible for the significant different prevalence of COVID-19 between African-Africans and African-Americans. Aims Allergy and Immunology 5(3): 184-191. 10.3934/Allergy.2021014
[1413] Chodick G, Nutman A, Yiekutiel N, Shalev V (2020) Angiotensin-converting enzyme inhibitors and angiotensin-receptor blockers are not associated with increased risk of SARS-CoV-2 infection. J Travel Med 27(5). 10.1093/jtm/taaa069
[1414] da Silva WM, Brito PDS, de Sousa G, et al. (2022) Deaths due to COVID-19 in a state of northeastern Brazil: spatiotemporal distribution, sociodemographic and clinical and operational characteristics. Trans R Soc Trop Med Hyg 116(2): 163-172. 10.1093/trstmh/trab098
[1415] Darmadi D, Pakpahan C, Ruslie RH, Rezano A (2021) Inflammatory laboratory findings associated with severe illness among hospitalized individuals with COVID-19 in Medan, Indonesia: a cross-sectional study. F1000Res 10: 1246. 10.12688/f1000research.74758.2
[1416] Dave TV, Gopinathan Nair A, Hegde R, et al. (2021) Clinical Presentations, Management and Outcomes of Rhino-Orbital-Cerebral Mucormycosis (ROCM) Following COVID-19: A Multi-Centric Study. Ophthalmic Plast Reconstr Surg 37(5): 488-495. 10.1097/iop.0000000000002030
[1417] de Mestral C, Gomez D, Wilton AS, et al. (2022) A Population-Based Analysis of Diabetes-Related Care Measures, Foot Complications, and Amputation During the COVID-19 Pandemic in Ontario, Canada. JAMA Netw Open 5(1): e2142354. 10.1001/jamanetworkopen.2021.42354
[1418] Desai EJ, Pandya A, Upadhya I, Patel T, Banerjee S, Jain V (2021) Epidemiology, Clinical Features and Management of Rhino Orbital Mucormycosis in Post COVID 19 Patients. Indian J Otolaryngol Head Neck Surg 74(1): 1-5. 10.1007/s12070-021-02807-2
[1419] Deshmukh V, Shaikh AK, Habbu P (2021) Type 2 diabetes and COVID-19 related mortality in solapur: A whole population study. Int J Pharm Sci Rev Res 67(2): 83-86. 10.47583/ijpsrr.2021.v67i02.014
[1420] Dieter De S, Kristof De S, Pauline H, Stefaan G, Geert Antoine M (2020) Vitamin D deficiency as risk factor for severe COVID-19: a convergence of two pandemics. In:
[1421] Djaharuddin I, Munawwarah S, Nurulita A, Ilyas M, Tabri NA, Lihawa N (2021) Comorbidities and mortality in COVID-19 patients. Gac Sanit 35 Suppl 2: S530-s532. 10.1016/j.gaceta.2021.10.085
[1422] Donamou JBACLM, Camara DTDA, Abékan RJM, et al. (2021) Caractéristiques épidémiologiques et cliniques des patients COVID-19 admis en réanimation à l'hôpital Donka de

Conakry, Guinée: étude descriptive des 140 premiers cas hospitalisés. Anesthésie & Réanimation. 10.1016/j.anrea.2021.01.001
[1423] El-Kholy NA, El-Fattah AMA, Khafagy YW (2021) Invasive Fungal Sinusitis in Post COVID-19 Patients: A New Clinical Entity. Laryngoscope 131(12): 2652-2658. 10.1002/lary.29632
[1424] Elsa du B, Cari S, Remy D, et al. (2021) Communicable and non-communicable co-morbidities and the presentation of COVID-19 in an African setting of high HIV-1 and tuberculosis prevalence. medRxiv. 10.1101/2021.05.11.21256479
[1425] Ersan G, Akkiraz Bac G, Yuksel O, et al. (2020) The Demographic and Clinical Features of 479 COVID-19 Patients: A Single-center Experience. Mediterranean Journal of Infection Microbes and Antimicrobials 9: 8-8. 10.4274/mjima.galenos.2020.2020.9
[1426] Estiri H, Strasser ZH, Brat GA, Semenov YR, Patel CJ, Murphy SN (2021) Evolving phenotypes of non-hospitalized patients that indicate long COVID. BMC Med 19(1): 249. 10.1186/s12916-021-02115-0
[1427] Ferguson J, Rosser JI, Quintero O, et al. (2020) Characteristics and Outcomes of Coronavirus Disease Patients under Nonsurge Conditions, Northern California, USA, March-April 2020. Emerg Infect Dis 26(8): 1679-1685. 10.3201/eid2608.201776
[1428] Fernández-de-Las-Peñas C, Florencio LL, Gómez-Mayordomo V, Cuadrado ML, Palacios-Ceña D, Raveendran AV (2021) Proposed integrative model for post-COVID symptoms. Diabetes Metab Syndr 15(4): 102159. 10.1016/j.dsx.2021.05.032
[1429] Florien SvR, Linda PTJ, Maarten van S, Frans HR, Geert-Jan G, Sander van D (2021) Cardiovascular vulnerability predicts hospitalisation in primary care clinically suspected and confirmed COVID-19 patients: a model development and validation study. medRxiv. 10.1101/2021.05.12.21257075
[1430] Garg R, Bharangar S, Gupta S, Bhardwaj S (2021) Post Covid-19 Infection Presenting as Rhino-Orbital Mycosis. Indian J Otolaryngol Head Neck Surg: 1-8. 10.1007/s12070-021-02722-6
[1431] Goshayeshi L, Akbari Rad M, Bergquist R, Allahyari A, Hashemzadeh K, Hoseini B (2021) Demographic and clinical characteristics of severe Covid-19 infections: a cross-sectional study from Mashhad University of Medical Sciences, Iran. BMC Infect Dis 21(1): 656. 10.1186/s12879-021-06363-6
[1432] Güler T, Yurdakul FG, Acar Sivas F, et al. (2021) Rehabilitative management of post-acute COVID-19: clinical pictures and outcomes. Rheumatol Int 41(12): 2167-2175. 10.1007/s00296-021-05003-1
[1433] Guzmán-Castro S, Chora-Hernandez LD, Trujillo-Alonso G, et al. (2021) COVID-19 associated Mucormycosis, diabetes and steroid therapy: Experience in a Single Centre in Western Mexico. Mycoses. 10.1111/myc.13383
[1434] Harada M, Nishi T, Maeda T, Tanno K, Nishiya N, Arima H (2021) How do patients with chronic illnesses respond to a public health crisis? Evidence from diabetic patients in Japan during the COVID-19 pandemic. SSM Popul Health 16: 100961. 10.1016/j.ssmph.2021.100961
[1435] Hasan MZ, Biswas NK, Aziz AM, Chowdhury J, Haider SS, Sarker M (2021) Clinical profile and short-term outcomes of RT-PCR- positive patients with COVID-19: a cross-sectional study in a tertiary care hospital in Dhaka, Bangladesh. BMJ Open 11(12): e055126. 10.1136/bmjopen-2021-055126
[1436] Hawale D, Dhok A, Jain P (2021) Relation of crp, ferritin and procalcitonin level in people infection with covid-19. Indian Journal of Forensic Medicine and Toxicology 15(2): 4472-4478. 10.37506/ijfmt.v15i2.15085
[1437] Hendre A, Ghule A, Bhale R (2021) Comparison of demographical, clinical, comorbid condition and laboratory findings among Covid 19 patients who received mechanical ventilation in a tertiary care center. European Journal of Molecular and Clinical Medicine 8(4): 1174-1182
[1438] Huang CA, Mahmood O, Mills P, et al. (2021) Ethnic Variation in Hospitalized COVID-19 Patients and Correlation with Coagulopathy, Thrombosis, Prognosis and 30 Day Mortality Outcomes in California's Central Valley. Blood 138: 4074-4074. 10.1182/blood-2021-145608
[1439] Hussein AARM, Galal I, Mohamed MMAR, Ibrahim MEAA, Ahmed SB (2021) Survival and 30-day hospital outcome in hospitalized coronavirus disease 2019-infected patients in Upper Egypt: Multicenter study. Egyptian Journal of Chest Diseases and Tuberculosis 70(2): 254-261. 10.4103/ejcdt.ejcdt_103_20
[1440] Jabbar AA (2021) The correlation study between some biochemical parameters of 256 covid-19 cases considering diabetes. Indian Journal of Forensic Medicine and Toxicology 15(4): 277-287. 10.37506/ijfmt.v15i4.16718
[1441] Jain K, Surana A, Choudhary TS, Vaidya S, Nandedkar S, Purohit M (2022) Clinical and histology features as predictor of severity of mucormycosis in post-COVID-19 patients: An experience from a rural tertiary setting in Central India. SAGE Open Med 10: 20503121221074785. 10.1177/20503121221074785

[1442] Jain M, Tyagi R, Tyagi R, Jain G (2021) Post-COVID-19 Gastrointestinal Invasive Mucormycosis. Indian J Surg: 1-3. 10.1007/s12262-021-03007-6
[1443] Jamil M, Bhattacharya PK, Barman B, et al. (2021) Clinical and Demographic Profile of COVID-19 Patients: A Tertiary Level Hospital-Based Study From Northeast India. Cureus 13(10): e18881. 10.7759/cureus.18881
[1444] Joensen LE, Steenberg JL, Madsen KP, Willaing I (2021) What people with diabetes in Denmark worry about during the COVID-19 pandemic: A longitudinal study of the first 3 months of the COVID-19 pandemic. Diabet Med 38(11): e14665. 10.1111/dme.14665
[1445] Kamel FO, Magadmi RM, Alqutub ST, et al. (2021) Clinical and hematologic presentations of adults with COVID-19 patients in Jeddah: A case control study. J Infect Public Health 14(6): 709-716. 10.1016/j.jiph.2021.03.007
[1446] Kanecki K, Nitsch-Osuch A, Gorynski P, et al. (2021) Hospitalizations for COVID-19 in Poland: a study based on data from a national hospital register. Polish Archives of Internal Medicine-Polskie Archiwum Medycyny Wewnetrznej 131(6): 535-540. 10.20452/pamw.15946
[1447] Karagiannidis C, Mostert C, Hentschker C, et al. (2020) Case characteristics, resource use, and outcomes of 10â□ˆ021 patients with COVID-19 admitted to 920 German hospitals: an observational study. Lancet Respir Med 8(9): 853-862. 10.1016/S2213-2600(20)30316-7
[1448] Khan H, Miles S, Chopra T, Saksena R, Kavvoura F (2021) An audit comparing the difference in outcomes between patients with diabetes admitted during first and second wave of the covid-19 pandemic in a large district general hospital. Diabetic Medicine 38
[1449] Kumar HM, Sharma P, Rudramurthy SM, et al. (2022) Serum iron indices in COVID-19-associated mucormycosis: A case-control study. Mycoses 65(1): 120-127. 10.1111/myc.13391
[1450] Kumar MCMBACSRR (2021) Ferritin - The key model inflammatory marker in diabetic and non-diabetic COVID-19. Asian Journal of Medical Sciences 12(12): 23-31. 10.3126/ajms.v12i12.39717
[1451] Kute VB, Meshram HS, Patel HV, et al. (2021) Clinical Profiles and Outcomes of COVID-19 in Kidney Transplant Recipients: Experience From a High-Volume Public Sector Transplant Center in India. Exp Clin Transplant 19(9): 899-909. 10.6002/ect.2021.0188
[1452] Lalau J-D (2020) COVID and type 2 diabetes: National surveys in France and their analysis. In:
[1453] Lalchhuanawma A, Sanghi D, Chawngthu L (2021) The prevalence of comorbidities and associated risk factors among covid-19 deceased in Mizoram, India. International Journal of Research in Pharmaceutical Sciences 12(3): 1822-1832. 10.26452/ijrps.v12i3.4765
[1454] Lee SW, Moon SY, Yon DK (2021) Epidemiological and clinical characteristics of 5,628 patients with coronavirus disease 2019 in South Korea: A nationwide multicenter study. Allergy Asthma & Respiratory Disease 9(3): 136-140. 10.4168/aard.2021.9.3.136
[1455] Mahmood K, Al-Nori HAM, Ali Z (2021) Serological and epidemiological study on COVID-19 infections in Nineveh governorate. Current Pediatric Research 25(8): 821-828
[1456] Mahmood ZS, Fadhil HY, Adhiah AH (2021) Estimation of hematological parameters of disease severity in iraqi patients with covid-19. Iraqi Journal of Science 62(10): 3487-3496. 10.24996/ijs.2021.62.10.8
[1457] Maldonado-Cabrera A, Angulo-Molina A, Haque U, et al. (2021) Acute Inflammatory Mediators in Young Adult Patients with COVID-19 in Mexico. Pathogens 10(8). 10.3390/pathogens10081056
[1458] Marks BE, Khilnani A, Meyers A, et al. (2021) Increase in the Diagnosis and Severity of Presentation of Pediatric Type 1 and Type 2 Diabetes during the COVID-19 Pandemic. Horm Res Paediatr 94(7-8): 275-284. 10.1159/000519797
[1459] Marquès M, Correig E, Ibarretxe D, et al. (2021) Long-term exposure to PM₁₀ above WHO guidelines exacerbates COVID-19 severity and mortality. Environ Int 158: 106930-106930. 10.1016/j.envint.2021.106930
[1460] Martinuzzi ALN, Manzanares W, Quesada E, et al. (2021) Nutritional risk and clinical outcomes in critically ill adult patients with COVID-19. Nutr Hosp 38(6): 1119-1125. 10.20960/nh.03749
[1461] Medina Fuentes G, Carbajales León EB, Carbajales León AI, Figueredo González Y, Montiel Martínez L (2021) Clinical-epidemiological characteristics of patients confirmed to the disease in the post-COVID-19 stage in Camagüey. Multimed (Granma) 25(3): e2165-e2165
[1462] Medina GN (2021) Análisis espacio-temporal de la tasa de letalidad por coronavirus dada la preexistencia de diabetes en municipios del noreste de México. Espacio Abierto 30(2): 87-87. 10.5281/zenodo.4966096
[1463] Meel R, Van Blydenstein SA (2021) Demographic, clinical, electrocardiographic and echocardiographic characteristics of patients hospitalized with COVID-19 and cardiac disease at a tertiary hospital, South Africa. Cardiovasc Diagn Ther 11(6): 1228-1240. 10.21037/cdt-21-459
[1464] Melekhov AV, Sayfullin MA, Petrovichev VS, Agaeva AI, Golubykh KY, Nikitin IG (2021) Association of Computer Tomography Features of COVID-19 with Outcomes, Clinical and Laboratory

Parameters. Russian Archives of Internal Medicine 11(6): 447-456. 10.20514/2226-6704-2021-11-6-447-456
[1465] Mesri M, Rouhani MR, Koohestani HR, Azani H, Ahad A, Karimy M (2021) Clinical features and outcomes of suspected and confirmed covid-19 patients in Saveh, Iran, 2020. Journal of Mazandaran University of Medical Sciences 30(194): 51-61
[1466] Mikusova V, Mikus J, Pridavkova D, Plichtova M, Martinka E, Mokan M (2021) Risk Factors of Hypoglycaemia in Prae-Covid-19 Era. Lekarsky Obzor 70(12): 485-489
[1467] Mir MM, Mir R, Alghamdi MAA, et al. (2021) Strong Association of Angiotensin Converting Enzyme-2 Gene Insertion/Deletion Polymorphism with Susceptibility to SARS-CoV-2, Hypertension, Coronary Artery Disease and COVID-19 Disease Mortality. J Pers Med 11(11). 10.3390/jpm11111098
[1468] Mistanoglu Ozatag D, Korkmaz P, Aslan H, Percin D (2021) Evaluation of the Clinical and Laboratory Characteristics and Factors Determining the Severity of the Disease in Hospitalized COVID-19 Patients. Flora Infeksiyon Hastaliklari Ve Klinik Mikrobiyoloji Dergisi 26(4): 646-654. 10.5578/flora.20219609
[1469] Mohabbat HDSC, Raza MT, Ahmed IU, Eva IJ, Tasnimul KCP, Gupta SD (2021) Immediate and post-COVID complications of symptomatic and asymptomatic COVID-19 patients in Bangladesh: a cross-sectional retrospective study. Asian Journal of Medical and Biological Research 7(2): 191-201. 10.3329/ajmbr.v7i2.54999
[1470] Moya-Salazar J, Cañari B, Sánchez-Llanos A, et al. (2021) Risk factors in rural Andean population with COVID-19: a retrospective cohort study. Infectio 25(4): 256-261
[1471] Müller A, Soyano A, Chirinos L, et al. (2021) COVID-19: Hematological and laboratory findings in patients from a private hospital in Caracas, Venezuela. Correlation with mortality. Gaceta Medica de Caracas 129(2): 279-291. 10.47307/GMC.2021.129.2.2
[1472] Murujosa AC, Pasik NI, Giuliani Quaglierini SA, et al. (2022) [Descriptive study of infection by SARS-CoV-2 in adults with diabetes]. Medicina (B Aires) 82(1): 28-34
[1473] Nafilyan V, Islam N, Mathur R, et al. (2021) Ethnic differences in COVID-19 mortality during the first two waves of the Coronavirus Pandemic: a nationwide cohort study of 29 million adults in England. Eur J Epidemiol 36(6): 605-617. 10.1007/s10654-021-00765-1
[1474] Nayar M, Varghese C, Kanwar A, et al. (2021) SARS-CoV-2 infection is associated with an increased risk of idiopathic acute pancreatitis but not pancreatic exocrine insufficiency or diabetes: long-term results of the COVIDPAN study. Gut. 10.1136/gutjnl-2021-326218
[1475] Nehara HR, Agrawal S, Chhimpa AR, Ih S, Arakeri A, Sirohi P (2021) Clinical, laboratory and radiological features and outcomes of moderate to severe COVID-19 patients: A descriptive retrospective study. Journal of Acute Disease 10(2): 62-70. 10.4103/2221-6189.312154
[1476] Nunes MC, Hale MJ, Mahtab S, et al. (2022) Clinical characteristics and histopathology of COVID-19 related deaths in South African adults. PLoS One 17(1): e0262179. 10.1371/journal.pone.0262179
[1477] Nyman MA, Jose T, Croghan IT, et al. (2022) Utilization of an Electronic Health Record Integrated Risk Score to Predict Hospitalization Among COVID-19 Patients. J Prim Care Community Health 13: 21501319211069748. 10.1177/21501319211069748
[1478] Olanipekun T, Abe TA, Effoe VS, et al. (2021) Intensive care unit hospitalizations and outcomes in patients with severe COVID-19 during summer and fall surges in Georgia. World J Crit Care Med 10(6): 369-376. 10.5492/wjccm.v10.i6.369
[1479] Omland T, Prebensen C, Jonassen C, et al. (2021) Soluble ST2 concentrations associate with in-hospital mortality and need for mechanical ventilation in unselected patients with COVID-19. Open Heart 8(2). 10.1136/openhrt-2021-001884
[1480] Oralia N-J, Sr., Luis Alberto Q-T, Sr., Erika ER-T, Sr., Guillermo F-A, Sr. (2021) COVID-19 Patients Analysis using Superheat Map and Bayesian Network to identify Comorbidities Correlations under Different Scenarios. medRxiv. 10.1101/2021.05.11.21257055
[1481] Özköse F, Yavuz M (2022) Investigation of interactions between COVID-19 and diabetes with hereditary traits using real data: A case study in Turkey. Comput Biol Med 141: 105044. 10.1016/j.compbiomed.2021.105044
[1482] Patil S, Gondhali G, Patil R, Kasture L (2021) Post-COVID-19 lung fibrosis: Study of 600 cases in tertiary care setting in India. American Journal of Respiratory and Critical Care Medicine 203(9). 10.1164/ajrccm-conference.2021.203.1_MeetingAbstracts.A2502
[1483] Peter L, Peter B, Jr., Robert R, et al. (2021) A machine learning approach for identification of gastrointestinal predictors for the risk of COVID-19 related hospitalization. medRxiv. 10.1101/2021.08.27.21262728
[1484] Petrakis V, Trypsianis G, Panagopoulos P, Papazoglou D, Papanas N (2021) Diabetes Mellitus as the Major Factor of Prolonged Hospitalisation in Mild or Moderate COVID-19 Pneumonia. Exp Clin Endocrinol Diabetes. 10.1055/a-1468-4296

[1485] Pezo Dianderas KM, Fernández DRC, Serna REP (2021) Características epidemiológicas de los pacientes atendidos por COVID-19 en el Servicio de Emergencia del Hospital Militar Central Luis Arias Schreiber. Horizonte Medico 21(3): 1DUMMT-1DUMMT. 10.24265/horizmed.2021.v21n3.04
[1486] Poblete G, Cox W, Dasso A, Begher S (2021) Epidemiological and clinical characteristics of patients hospitalized with COVID-19 in Buenos Aires cityEsta obra está bajo una licencia de CreativeCommons Reconocimiento-NoComercial 4.0Internacionalhttps://creativecommons.org/licenses/by-nc/4.0/i. Actual SIDA infectol 29(105): 42-48
[1487] Prajapati AC (2021) A hospital based cross sectional study to find out factors associated with disease severity and length of hospital stay in covid-19 patients in tertiary care hospital of ahmedabad city. Indian Journal of Community Health 33(2): 256-259. 10.47203/IJCH.2021.v33i02.006
[1488] Pretorius E, Vlok M, Venter C, et al. (2021) Persistent clotting protein pathology in Long COVID/Post-Acute Sequelae of COVID-19 (PASC) is accompanied by increased levels of antiplasmin. Cardiovasc Diabetol 20(1): 172. 10.1186/s12933-021-01359-7
[1489] Puerta JL, Torrego-Ellacuría M, Del Rey-Mejías A, Biénzobas López C (2022) Comorbidities of Primary Care patients with COVID-19 during the first wave of the SARS-CoV-2 pandemic in the Community of Madrid. Rev Esp Quimioter 35(1): 63-70. 10.37201/req/114.2021
[1490] Qayyum W, Khan SA, Saeed L, Jawad M, Khan MF, Ullah I (2021) Corona Virus Disease-19 Clinical And Laboratory Characteristics Associated With Disease Severity In Patients Presenting At A Tertiary Care Hospital Of Peshawar. J Ayub Med Coll Abbottabad 33(3): 507-512
[1491] Qureshi QH, Ashraf T, Rehman K, Khosa MK, Akash MSH (2021) Therapeutic interventions of remdesivir in diabetic and nondiabetic COVID-19 patients: A prospective observational study conducted on Pakistani population. J Med Virol 93(12): 6732-6736. 10.1002/jmv.27256
[1492] Ramírez-Aldana R, Gomez-Verjan JC, Bello-Chavolla OY, García-Peña C (2021) Spatial epidemiological study of the distribution, clustering, and risk factors associated with early COVID-19 mortality in Mexico. PLoS One 16(7): e0254884. 10.1371/journal.pone.0254884
[1493] Ran J, Zhao S, Han L, et al. (2021) Increase in Diabetes Mortality Associated With COVID-19 Pandemic in the U.S. Diabetes Care 44(7): e146-e147. 10.2337/dc21-0213
[1494] Rangankar V, Koganti DV, Lamghare P, et al. (2021) Correlation Between CT Severity Scoring and Diabetes Mellitus in Patients With COVID-19 Infection. Cureus 13(12): e20199. 10.7759/cureus.20199
[1495] Rehatta N, Chandra S, Sari D, et al. (2021) Comorbidities and COVID-19 Status Influence the Survival Rate of Geriatric Patients in Intensive Care Units: a Prospective Cohort Study From the Indonesian Society of Anaesthesiology and Intensive Therapy. ResearchSquare. 10.21203/rs.3.rs-969262/v1
[1496] Reyes C, Pistillo A, Fernández-Bertolín S, et al. (2021) Characteristics and outcomes of patients with COVID-19 with and without prevalent hypertension: a multinational cohort study. BMJ Open 11(12): e057632. 10.1136/bmjopen-2021-057632
[1497] Rinderknecht MD, Klopfenstein Y (2021) Predicting critical state after COVID-19 diagnosis: model development using a large US electronic health record dataset. NPJ Digit Med 4(1): 113. 10.1038/s41746-021-00482-9
[1498] Robey RC, Kemp K, Hayton P, et al. (2021) Pulmonary Sequelae at 4 Months After COVID-19 Infection: A Single-Centre Experience of a COVID Follow-Up Service. Adv Ther 38(8): 4505-4519. 10.1007/s12325-021-01833-4
[1499] Roth A, Chan PS, Jonas W (2021) Addressing the Long COVID Crisis: Integrative Health and Long COVID. Glob Adv Health Med 10: 21649561211056597. 10.1177/21649561211056597
[1500] Rowlands AV, Dempsey PC, Gillies C, et al. (2021) Association Between Accelerometer-Assessed Physical Activity and Severity of COVID-19 in UK Biobank. Mayo Clin Proc Innov Qual Outcomes 5(6): 997-1007. 10.1016/j.mayocpiqo.2021.08.011
[1501] Sadiq F, Ayoub K, Riaz F, Razzaq S, Farooq A, Khalid M (2021) Acute Respiratory Distress Syndrome in Confirmed and Suspected Cases of Covid-19: A Cross Sectional Study. Pakistan Journal of Medical & Health Sciences 15(6): 1176-1179. 10.53350/pjmhs211561176
[1502] Santana MF, Frank CHM, Almeida TVR, et al. (2021) Hemorrhagic and thrombotic manifestations in the central nervous system in COVID-19: A large observational study in the Brazilian Amazon with a complete autopsy series. PLoS One 16(9): e0255950. 10.1371/journal.pone.0255950
[1503] Sardinha DM, Lima KV, Ferreira AL, et al. (2021) Clinical and spatial characteristics of Severe Acute Respiratory Syndrome by COVID-19 in Indigenous of Brazil. medRxiv. 10.1101/2020.10.24.20218701
[1504] Sayed TM, Arshad SH, Aalam M, Khan F, Majeed S (2021) ICU stay and mortality between vaccinated and non-vaccinated patients of Covid-19;A comparative study. Pakistan Journal of Medical and Health Sciences 15(9): 2789-2792. 10.53350/pjmhs211592789
[1505] Sen M, Honavar SG, Bansal R, et al. (2021) Epidemiology, clinical profile, management, and outcome of COVID-19-associated rhino-orbital-cerebral mucormycosis in 2826 patients in India -

Collaborative OPAI-IJO Study on Mucormycosis in COVID-19 (COSMIC), Report 1. Indian J Ophthalmol 69(7): 1670-1692. 10.4103/ijo.IJO_1565_21
[1506] Shah D, Talwar D, Kumar S, Acharya S, Dubey A (2021) Mucormycosis as a complication of LOIN G COVID: A case series. Medical Science 25(112): 1331-1337
[1507] Shestakova MV, Kononenko IV, Kalmykova ZA, Zheleznyakova AV, Mokrysheva NG (2020) High level of glycated hemoglobin (hba1c) in patients with covid-19 is a marker of the severity of the infection but not an indicator of previous diabetes mellitus. Diabetes Mellitus 23(6): 504-513. 10.14341/DM12702
[1508] Shubham R, Anupreet k, Ramna T (2021) Gendered Differentials in Symptoms, Morbidity, and Case Fatality Rate in COVID-19 Pandemic in India: A Study of Karnataka State. SSRN. 10.2139/ssrn.3865604
[1509] Sipahi S, Dheir H, Toçoğlu A, et al. (2021) Characteristics and mortality determinants of COVID-19 patients undergoing hemodialysis. Turk J Med Sci 51(2): 421-427. 10.3906/sag-2006-54
[1510] Sirisati RSKCSLAGKBNRKS (2021) Identification of Mucormycosis in post Covid-19 case using Deep CNN. Turkish Journal of Computer and Mathematics Education 12(9): 3441-3450
[1511] Spanakis EK, Yoo A, Ajayi ON, et al. (2021) Excess Mortality in COVID-19-Positive Versus COVID-19-Negative Inpatients With Diabetes: A Nationwide Study. Diabetes Care 44(9): e169-e170. 10.2337/dc20-2350
[1512] Su YYDCDG, Ng RH, Wang KCJLSHSZRXJKSA, et al. (2022) Multiple Early Factors Anticipate Post-Acute COVID-19 Sequelae. Cell. 10.1016/j.cell.2022.01.014
[1513] Sukmagautama C, Hartono NANE, Hayati K, Aditya B, Marthasari R (2021) Clinical characteristics, degree of severity and mortality rate of COVID-19 patient in Central Java, Indonesia. Malaysian Journal of Medicine and Health Sciences 17: 108-112
[1514] Swedish Herbal Institute AB (2021) Efficacy of Adaptogens in Patients With Long COVID-19. In:
[1515] Syed U, Rizvi A, Hassan SA, Syed U, Syed I (2021) Frequency of covid outcome in covid patients with pre-existing different co-morbid conditions. Pakistan Journal of Medical and Health Sciences 15(10): 2534-2536. 10.53350/pjmhs2115102534
[1516] Tahmina Z, Morshed N, Rawshan Ara P, et al. (2021) Overview of 254 mild cases of COVID-19 in Bangladeshi cohort: a cross-sectional observation. International Journal of Advances in Medicine. 10.18203/2349-3933.IJAM20211896
[1517] Tañada MRPK, Tanchee-Ngo MJ (2021) Association of protein-calorie intake and clinical outcomes among critically-ill covid-19 patients: A retrospective study. Phillippine Journal of Internal Medicine 59(3): 246-254
[1518] Tendulkar P, Pandey P, Panda PK, Bhadoria AS, Kulshreshtha P (2022) Descriptive Epidemiology of COVID-19 Deaths during the First Wave of Pandemic in India: A Single-center Experience. Indian J Crit Care Med 26(1): 71-75. 10.5005/jp-journals-10071-24085
[1519] Toori KU, Qureshi MA, Chaudhry A, Safdar MF (2021) Neutrophil to lymphocyte ratio (NLR) in COVID-19: A cheap prognostic marker in a resource constraint setting. Pak J Med Sci 37(5): 1435-1439. 10.12669/pjms.37.5.4194
[1520] Tzur Bitan D, Kridin K, Cohen AD, Weinstein O (2021) COVID-19 hospitalisation, mortality, vaccination, and postvaccination trends among people with schizophrenia in Israel: a longitudinal cohort study. Lancet Psychiatry 8(10): 901-908. 10.1016/s2215-0366(21)00256-x
[1521] Vardhan H, Kumar A, Shyama S, et al. (2021) Clinical Profile and Outcome of Haemodialysis in Patients With COVID-19 - A Single Centre Experience. Cureus 13(8): e17170. 10.7759/cureus.17170
[1522] Vila-Córcoles ÁO-G, Olga T-F, Cristina V-R, et al. (2020) Evaluación de la incidencia y perfil de riesgo de Covid-19 según comorbilidad previa en adultos &#880550 años del área de Tarragona. Revista Española de Salud Pública 94
[1523] Vladimir P, Sokolova LK, Cherviakova SA, et al. (2021) Effects of COVID-19 and Diabetes Mellitus on Apolipoprotein A1 Level in the Blood Plasma of Patients. SSRN
[1524] Vysochyna IL, Nikolaenko-Kamyshova TP, Biesiedin OM (2021) Postcovid syndrome: regarding the risks of developing thrombotic complications in patients with ischemic limb lesions (clinical cases). Medical Perspectives-Medicni Perspektivi 26(3): 205-211. 10.26641/2307-0404.2021.3.242332
[1525] Whiteside DM, Basso MR, Naini SM, et al. (2022) Outcomes in post-acute sequelae of COVID-19 (PASC) at 6 months post-infection Part 1: Cognitive functioning. Clin Neuropsychol: 1-23. 10.1080/13854046.2022.2030412
[1526] Widiastuti L (2021) Perbedaan kualitas hidup pasien COVID-19 dengan komorbid. Jurnal Keperawatan Silampari 5(1): 233-239. https://journal.ipm2kpe.or.id/index.php/JKS/article/view/2833/1727
[1527] World Health Organization ROfA (2020) The effects of COVID-19 in people living with diabetes – based on information as at 31 October 2020. In:

[1528] Yousif MYEEMMFAAOAMSAKATAAMAONAHMAKAH (2021) Clinical Characteristics And Analysis Of Risk Factors For Disease Progression Of COVID-19 Among Patients Within Wad Medani Isolation Centers, Gezira State: A Retrospective Cohort Study From Sudan. In:
[1529] Zeng J, Qi XX, Cai WW, Pan YP, Xie Y (2021) Retrospective analysis of influencing factors on the efficacy of mechanical ventilation in severe and critical COVID-19 patients. World J Clin Cases 9(31): 9481-9490. 10.12998/wjcc.v9.i31.9481
[1530] Zhang C, Lin T, Guo L, et al. (2021) Clinical characteristics and outcome of COVID-19 in elderly patients. Journal of Xi'an Jiaotong University (Medical Sciences) 42(6): 915-922. 10.7652/jdyxb202106021
[1531] Zhou P, Gao J, Biggerstaff K, Orengo-Nania S, Channa R (2021) Impact of the COVID-19 Pandemic on Diabetic Macular Edema Patient Outcomes at a Tertiary Care Veterans Affairs Hospital. Investigative Ophthalmology & Visual Science 62(8)
[1532] 关欣, 关海霞, 谷野, et al. (2020) Prolonged Duration of Positive Virus-PCR Result in Patients with Coronavirus Disease 2019 May Be Associated with Diabetes Mellitus. 中华内分泌代谢杂志
[1533] 占婷婷, 郑海鹏, 麦玉珍, 李文锋, 卓友光 (2020) The Correlation between Serological Dynamic Evolution and the Severity of Coronavirus Disease 2019. 广东医学
[1534] 安薇, 夏飞, 陈敏, et al. (2020) Analysis of Clinical Features of 11 Death Cases Caused by COVID-19. 实用医学杂志
[1535] 张长升, 胡晶, 占志兵, 董铮, 柳琦, 陶佳意 (2020) Correlation between Serum Cystatin C and Prognosis of Patients with COVID-19. 实用医学杂志
[1536] 陆文峰, 张洁, 李丹, 何兵, 周晓阳 (2020) Analysis on 28 death cases of COVID-19. 中国感染控制杂志. 10.12138/j.issn.1671-9638.20206860
[1537] 马宇航, 周小建, 章志健, et al. (2020) Clinic and Metabolitic Characteristics in 165 Ordinary COVID-19 Patients. 中华内分泌代谢杂志. 10.3760/cma.j.cn311282-20200403-00244
[1538] Donamou J, Camara D, Traore AD, et al. (2021) COVID-19 and diabetes: frequency, clinical signs and mortality in intensive care COVID at Donka national hospital in Conakry. Medecine d'Afrique Noire 68(1): 5-12
[1539] Williams DM, Davies J, Field BCT, et al. (2021) COVID-19 outcomes in people with diabetes in Wales: a secondary analysis of the ABCD audit. British Journal of Diabetes 21(2): 222-227. 10.15277/bjd.2021.319
[1540] Akter A, Tabassum N, Rahman A (2021) Prevalence of severe acute respiratory syndrome Coronavirus-2 among the young people and association between diabetes, hypertension, and severe acute respiratory syndrome Coronavirus-2. Biomedical and Biotechnology Research Journal 5(2): 134-138. 10.4103/bbrj.bbrj-220-20
[1541] Al-Terehi MN, Jawad MA, Kadhim AJ, Abed AS (2021) Impact of diabetes mellitus medications in some biomarkers of covid-19 infected patients. International Journal of Pharmaceutical Quality Assurance 12(3): 184-186. 10.25258/ijpqa.12.3.3
[1542] Caycho-Rodriguez T, Valencia PD, Vilca LW, et al. (2022) Sociodemographic and Health Predictors of Concern about COVID-19 Infection in Cuban Patients with Type 2 Diabetes Mellitus. Electronic Journal of General Medicine 19(2): 7-7. 10.29333/ejgm/11620
[1543] Seiglie JA, Serván-Mori E, Manne-Goehler J, et al. (2021) Diabetes mellitus as a risk factor for SARS-CoV-2 test positivity in Mexico: A propensity score matched study. Diabetes Res Clin Pract 178: 108953. 10.1016/j.diabres.2021.108953
[1544] Liu H, Wu X, Nie H, Luo Y (2022) Risk factors analysis on the death of COVID-19 patients combined with diabetes mellitus. Medical Journal of Wuhan University 43(1): 6-10. 10.14188/j.1671-8852.2020.0757
[1545] Hossain MI, Parvin S, Islam MS, et al. (2021) Demographic profile and outcome of patients admitted to a COVID dedicated hospital in Bangladesh during the second wave. Medicine (Baltimore) 100(37): e27281. 10.1097/md.0000000000027281
[1546] Babamahmoodi F, Ahangarkani F, Charati JY, et al. (2021) Clinical and para-clinical findings and risk factors among COVID-19 survived and deceased patients in north of Iran, 2019-2020. Journal of Mazandaran University of Medical Sciences 31(201): 70-82
[1547] Biju RA, Jacob JA (2021) Incidence of newly detected diabetes among covid-19 patients in atertiary care hospital. Journal of Global Trends in Pharmaceutical Sciences 12(3): 9596-9598
[1548] Ahmed GK, Khedr EM, Hamad DA, Meshref TS, Hashem MM, Aly MM (2021) Long term impact of Covid-19 infection on sleep and mental health: A cross-sectional study. Psychiatry Res 305: 114243. 10.1016/j.psychres.2021.114243

[1549] Akbari A, Fathabadi A, Razmi M, et al. (2022) Characteristics, risk factors, and outcomes associated with readmission in COVID-19 patients: A systematic review and meta-analysis. Am J Emerg Med 52: 166-173. 10.1016/j.ajem.2021.12.012
[1550] Crankson S, Pokhrel S, Anokye NK (2022) Determinants of COVID-19-Related Length of Hospital Stays and Long COVID in Ghana: A Cross-Sectional Analysis. Int J Environ Res Public Health 19(1). 10.3390/ijerph19010527
[1551] Daugherty SE, Guo Y, Heath K, et al. (2021) Risk of clinical sequelae after the acute phase of SARS-CoV-2 infection: retrospective cohort study. Bmj 373: n1098. 10.1136/bmj.n1098
[1552] Ellen JT, Dylan MW, Alex JW, et al. (2021) Risk factors for long COVID: analyses of 10 longitudinal studies and electronic health records in the UK. In:
[1553] Emma R-P, Abdel D, Xiaohui S, Phillip JC, Ajay MS, Martin CG (2021) Differential impact of Covid-19 on incidence of diabetes mellitus and cardiovascular diseases in acute, post-acute and long Covid-19: population-based cohort study in the United Kingdom. medRxiv. 10.1101/2021.12.13.21267723
[1554] Fernández-de-Las-Peñas C, Guijarro C, Torres-Macho J, et al. (2021) Diabetes and the Risk of Long-term Post-COVID Symptoms. Diabetes 70(12): 2917-2921. 10.2337/db21-0329
[1555] Husain I, O'Neill J, Mudge R, et al. (2021) Clinical Characteristics Associated with Return Visits to the Emergency Department after COVID-19 Diagnosis. West J Emerg Med 22(6): 1257-1261. 10.5811/westjem.2021.9.52824
[1556] Jacob L, Koyanagi A, Smith L, et al. (2021) Prevalence of, and factors associated with, long-term COVID-19 sick leave in working-age patients followed in general practices in Germany. Int J Infect Dis 109: 203-208. 10.1016/j.ijid.2021.06.063
[1557] Jahanzeb M, Syed Muhammad Jawad Z, Uzma I, et al. (2021) Post-acute COVID-19 syndrome and its prolonged effects: An updated systematic review. medRxiv. 10.1101/2021.05.09.21256911
[1558] Jindal G, Sethi A, Bhargarva K, et al. (2022) Imaging findings in invasive rhino-orbito-cerebral mucormycosis in post-COVID-19 patients. Proc (Bayl Univ Med Cent) 35(1): 32-34. 10.1080/08998280.2021.1981100
[1559] Lewek J, Jatczak-Pawlik I, Maciejewski M, Jankowski P, Banach M (2021) COVID-19 and cardiovascular complications – the preliminary results of the LATE-COVID study. Archives of Medical Science 17(3): 818-822. 10.5114/aoms/134211
[1560] Li Y, Han X, Huang J, et al. (2021) Follow-up study of pulmonary sequelae in discharged COVID-19 patients with diabetes or secondary hyperglycemia. Eur J Radiol 144: 109997. 10.1016/j.ejrad.2021.109997
[1561] Mechi A, Al-Khalidi A, Al-Darraji R, et al. (2021) Long-term persistent symptoms of COVID-19 infection in patients with diabetes mellitus. Int J Diabetes Dev Ctries: 1-4. 10.1007/s13410-021-00994-w
[1562] Mitra S, Janweja M, Sengupta A (2021) Post-COVID-19 rhino-orbito-cerebral mucormycosis: a new addition to challenges in pandemic control. Eur Arch Otorhinolaryngol: 1-6. 10.1007/s00405-021-07010-1
[1563] Mittal J, Ghosh A, Bhatt SP, Anoop S, Ansari IA, Misra A (2021) High prevalence of post COVID-19 fatigue in patients with type 2 diabetes: A case-control study. Diabetes Metab Syndr 15(6): 102302. 10.1016/j.dsx.2021.102302
[1564] Mohiuddin Chowdhury ATM, Karim MR, Ali MA, Islam J, Li Y, He S (2021) Clinical Characteristics and the Long-Term Post-recovery Manifestations of the COVID-19 Patients-A Prospective Multicenter Cross-Sectional Study. Front Med (Lausanne) 8: 663670. 10.3389/fmed.2021.663670
[1565] Nambiar M, Varma SR, Damdoum M (2021) Post-Covid alliance-mucormycosis, a fatal sequel to the pandemic in India. Saudi J Biol Sci 28(11): 6461-6464. 10.1016/j.sjbs.2021.07.004
[1566] Rajueni K, Ambekar R, Solanki H, Momin AA, Pawar S (2021) Assessment of the possible causes of diabetes mellitus developed in patients post covid-19 treatment in a tertiary care hospital. International Journal of Pharmacy and Pharmaceutical Sciences 13(9): 11-15. 10.22159/ijpps.2021v13i9.42508
[1567] Rezel-Potts EDASXCPSAGM Differential impact of Covid-19 on incidence of diabetes mellitus and cardiovascular diseases in acute, post-acute and long Covid-19: population-based cohort study in the United Kingdom (preprint). In:
[1568] Shirley C, Subhash P, Nana Kwame A (2021) Determinants of COVID-19 Related Length of Hospital Stays and Long Covid in Ghana: A Cross-Sectional Analysis. SSRN
[1569] Yaksi N, Teker AG, Imre A (2022) Long COVID in Hospitalized COVID-19 Patients: A Retrospective Cohort Study. Iranian Journal of Public Health 51(1): 88-95
[1570] Mir M, Jones L, Levine S (2021) POST-COVID-19-ASSOCIATED ASPERGILLUS EMPYEMA AND PARENCHYMAL ABSCESS. Chest 160(4): A355-A355. 10.1016/j.chest.2021.07.357

[1571] Jones R, Davis A, Stanley B, et al. (2021) Risk Predictors and Symptom Features of Long COVID Within a Broad Primary Care Patient Population Including Both Tested and Untested Patients. Pragmat Obs Res 12: 93-104. 10.2147/por.S316186
[1572] Islam MK, Molla MMA, Hasan P, et al. (2021) Persistence of sleep disturbance among post-COVID patients: Findings from a 2-month follow-up study in a Bangladeshi cohort. J Med Virol. 10.1002/jmv.27397
[1573] Zhang J, Tao Y, Xu ZH, Liu YH (2021) Type 2 Diabetes Increased the Risk of Hyposmia in COVID-19 Mild Pneumonia Patients by Suppressing Nasal Immunity. Journal of Immunology 206
[1574] Diallo A, Pichelin M, Wargny M, et al. (2022) Influenza vaccination and prognosis for COVID-19 in hospitalized patients with diabetes: Results from the CORONADO study. Diabetes Obes Metab 24(2): 343-347. 10.1111/dom.14577
[1575] Guo J, Lin WW, Zucker JE, et al. (2022) Inflammation and Mortality in COVID-19 Hospitalized Patients With and Without Type 2 Diabetes. J Clin Endocrinol Metab 107(5): e1961-e1968. 10.1210/clinem/dgac003
[1576] Leng Y, Chen M, Dai M, et al. (2021) Minimized glycemic fluctuation decreases the risk of severe illness and death in patients with COVID-19. J Med Virol 93(7): 4060-4062. 10.1002/jmv.26584
[1577] Ramon J, Llaurado G, Guerri R, et al. (2022) Acute-to-Chronic Glycemic Ratio as a Predictor of COVID-19 Severity and Mortality. Diabetes Care 45(1): 255-258. 10.2337/dc21-1321
[1578] Casas-Rojo JM, Antón-Santos JM, Millán-Núñez-Cortés J, et al. (2020) Clinical characteristics of patients hospitalized with COVID-19 in Spain: Results from the SEMI-COVID-19 Registry. Rev Clin Esp. 10.1016/j.rce.2020.07.003
[1579] Jackson T, Deibert D, Wyatt G, et al. (2020) Classification of aerosol-generating procedures: a rapid systematic review. BMJ Open Respir Res 7(1). 10.1136/bmjresp-2020-000730
[1580] Chioma I-E, Walter D, Anjali A, et al. (2020) Severe COVID-19 and Diabetes: A Retrospective Cohort Study from Three London Teaching Hospitals. medRxiv. 10.1101/2020.08.07.20160275
[1581] Halvatsiotis P, Kotanidou A, Tzannis K, et al. (2020) Demographic and clinical features of critically ill patients with COVID-19 in Greece: The burden of diabetes and obesity. Diabetes Res Clin Pract 166: 108331. 10.1016/j.diabres.2020.108331
[1582] Jing Liang J, Liu J, Chen Y, et al. (2020) Characteristics of laboratory findings of COVID-19 patients with comorbid diabetes mellitus. Diabetes Res Clin Pract 167: 108351. 10.1016/j.diabres.2020.108351
[1583] Raoufi M, Khalili S, Mansouri M, Mahdavi A, Khalili N (2020) Well-controlled vs poorly-controlled diabetes in patients with COVID-19: Are there any differences in outcomes and imaging findings? Diabetes Res Clin Pract 166: 108286. 10.1016/j.diabres.2020.108286
[1584] Yi H, Lu F, Jin X, et al. (2020) Clinical Characteristics and Outcomes of Coronavirus Disease 2019 Infections among Diabetics: a Retrospective and Multicenter Study in China. J Diabetes. 10.1111/1753-0407.13098
[1585] (2020) Preliminary Estimates of the Prevalence of Selected Underlying Health Conditions Among Patients with Coronavirus Disease 2019 - United States, February 12-March 28, 2020. MMWR Morbidity and mortality weekly report 69(13): 382-386. 10.15585/mmwr.mm6913e2
[1586] Adams ML, Katz DL, Grandpre J (2020) Population-Based Estimates of Chronic Conditions Affecting Risk for Complications from Coronavirus Disease, United States. Emerging infectious diseases 26(8). 10.3201/eid2608.200679
[1587] Alsofayan YM, Althunayyan SM, Khan AA, Hakawi AM, Assiri AM (2020) Clinical characteristics of COVID-19 in Saudi Arabia: A national retrospective study. Journal of infection and public health. 10.1016/j.jiph.2020.05.026
[1588] Argenziano MG, Bruce SL, Slater CL, et al. (2020) Characterization and clinical course of 1000 patients with coronavirus disease 2019 in New York: retrospective case series. Bmj 369: m1996. 10.1136/bmj.m1996
[1589] Bhatraju PK, Ghassemieh BJ, Nichols M, et al. (2020) Covid-19 in Critically Ill Patients in the Seattle Region - Case Series. N Engl J Med 382(21): 2012-2022
[1590] Bicheng Z, Xiaoyang Z, Yanru Q, et al. (2020) Clinical characteristics of 82 death cases with COVID-19. medRxiv: 2020.2002.2026.20028191. 10.1101/2020.02.26.20028191
[1591] Bode B, Garrett V, Messler J, et al. (2020) Glycemic Characteristics and Clinical Outcomes of COVID-19 Patients Hospitalized in the United States. J Diabetes Sci Technol: 1932296820924469. 10.1177/1932296820924469
[1592] Buckner FS, McCulloch DJ, Atluri V, et al. (2020) Clinical Features and Outcomes of 105 Hospitalized patients with COVID-19 in Seattle, Washington. Clinical infectious diseases : an official publication of the Infectious Diseases Society of America. 10.1093/cid/ciaa632
[1593] Cai Q, Chen F, Wang T, et al. (2020) Obesity and COVID-19 Severity in a Designated Hospital in Shenzhen, China. Diabetes care
[1594] Deng SQ, Peng HJ (2020) Characteristics of and Public Health Responses to the Coronavirus Disease 2019 Outbreak in China. Journal of clinical medicine 9(2). 10.3390/jcm9020575

[1595] Desai R, Singh S, Parekh T, Sachdeva S, Sachdeva R, Kumar G (2020) COVID-19 and diabetes mellitus: A need for prudence in elderly patients from a pooled analysis. Diabetes Metab Syndr 14(4): 683-685. 10.1016/j.dsx.2020.05.021
[1596] Du X, Li H, Dong L, Li X, Tian M, Dong J (2020) Clinical features of hemodialysis patients with COVID-19: a single-center retrospective study on 32 patients. Clinical and experimental nephrology: 1-7. 10.1007/s10157-020-01904-w
[1597] Du Y, Tu L, Zhu P, et al. (2020) Clinical Features of 85 Fatal Cases of COVID-19 from Wuhan. A Retrospective Observational Study. Am J Respir Crit Care Med 201(11): 1372-1379
[1598] Gao Q, Hu Y, Dai Z, Xiao F, Wang J, Wu J (2020) The epidemiological characteristics of 2019 novel coronavirus diseases (COVID-19) in Jingmen, Hubei, China. Medicine (Baltimore) 99(23): e20605-e20605
[1599] Garg S, Kim L, Whitaker M, et al. (2020) Hospitalization Rates and Characteristics of Patients Hospitalized with Laboratory-Confirmed Coronavirus Disease 2019 - COVID-NET, 14 States, March 1-30, 2020. MMWR Morbidity and mortality weekly report 69(15): 458-464. 10.15585/mmwr.mm6915e3
[1600] Gémes K, Talbäck M, Modig K, et al. (2020) Burden and prevalence of prognostic factors for severe COVID-19 in Sweden. Eur J Epidemiol 35(5): 401-409
[1601] Gianpaolo B, Elisabetta B, Ciro C, et al. (2020) SARS-COV-2 comorbidity network and outcome in hospitalized patients in Crema, Italy. medRxiv. 10.1101/2020.04.14.20053090
[1602] Guo T, Shen Q, Guo W, et al. (2020) Clinical Characteristics of Elderly Patients with COVID-19 in Hunan Province, China: A Multicenter, Retrospective Study. Gerontology: 1-9
[1603] Guo W, Li M, Dong Y, et al. (2020) Diabetes is a risk factor for the progression and prognosis of COVID-19. Diabetes Metab Res Rev: e3319-e3319
[1604] Hong KS, Lee KH, Chung JH, et al. (2020) Clinical Features and Outcomes of 98 Patients Hospitalized with SARS-CoV-2 Infection in Daegu, South Korea: A Brief Descriptive Study. Yonsei Med J 61(5): 431-437
[1605] Huang C, Wang Y, Li X, et al. (2020) Clinical features of patients infected with 2019 novel coronavirus in Wuhan, China. Lancet 395(10223): 497-506
[1606] Itelman E, Wasserstrum Y, Segev A, et al. (2020) Clinical Characterization of 162 COVID-19 patients in Israel: Preliminary Report from a Large Tertiary Center. Isr Med Assoc J 22(5): 271-274
[1607] José Manuel Casas R, Juan Miguel Antón S, Jesús Millán N-C, et al. (2020) Clinical characteristics of patients hospitalized with COVID-19 in Spain: results from the SEMI-COVID-19 Network. medRxiv. 10.1101/2020.05.24.20111971
[1608] Khamis F, Al-Zakwani I, Al Naamani H, et al. (2020) Clinical characteristics and outcomes of the first 63 adult patients hospitalized with COVID-19: An experience from Oman. Journal of infection and public health. 10.1016/j.jiph.2020.06.002
[1609] Leung C (2020) Clinical features of deaths in the novel coronavirus epidemic in China. Rev Med Virol 30(3): e2103-e2103
[1610] Li X, Wang L, Yan S, et al. (2020) Clinical characteristics of 25 death cases with COVID-19: A retrospective review of medical records in a single medical center, Wuhan, China. Int J Infect Dis 94: 128-132
[1611] Lian J, Jin X, Hao S, et al. (2020) Analysis of Epidemiological and Clinical features in older patients with Corona Virus Disease 2019 (COVID-19) out of Wuhan. Clin infect dis
[1612] Lijiao Z, Jialu L, Mingfeng L, et al. (2020) Risk assessment of progression to severe conditions for patients with COVID-19 pneumonia: a single-center retrospective study. medRxiv. 10.1101/2020.03.25.20043166
[1613] liu l, Gao J-y (2020) Clinical characteristics of 51 patients discharged from hospital with COVID-19 in Chongqing， China. medRxiv. 10.1101/2020.02.20.20025536
[1614] Michael GA, Samuel LB, Cody LS, et al. (2020) Characterization and Clinical Course of 1000 Patients with COVID-19 in New York: retrospective case series. medRxiv. 10.1101/2020.04.20.20072116
[1615] Qiao S, Kailiang Z, Jia Y, et al. (2020) Clinical characteristics of 101 non-surviving hospitalized patients with COVID-19: A single center, retrospective study. medRxiv. 10.1101/2020.03.04.20031039
[1616] Qin X, Qiu S, Yuan Y, et al. (2020) Clinical Characteristics and Treatment of Patients Infected with COVID-19 in Shishou, China. SSRN. 10.2139/ssrn.3541147
[1617] Shabto JM, Loerinc L, O'Keefe GA, O'Keefe J (2020) Characteristics and outcomes of COVID-19 positive patients with diabetes managed as outpatients. Diabetes Res Clin Pract 164: 108229. 10.1016/j.diabres.2020.108229
[1618] Shijiao Y, Xingyue S, Feng L, et al. (2020) Clinical Characteristics of Coronavirus Disease 2019 in Hainan, China. medRxiv. 10.1101/2020.03.19.20038539
[1619] Wang Z, Du Z, Zhu F (2020) Glycosylated hemoglobin is associated with systemic inflammation, hypercoagulability, and prognosis of COVID-19 patients. Diabetes Res Clin Pract 164: 108214-108214

[1620] Xiufeng J, Jianxin T, Hui W, et al. (2020) Clinical features and management of severe COVID-19: A retrospective study in Wuxi, Jiangsu Province, China. medRxiv. 10.1101/2020.04.10.20060335
[1621] Yang Q, Xie L, Zhang W, et al. (2020) Analysis of the clinical characteristics, drug treatments and prognoses of 136 patients with coronavirus disease 2019. J clin pharm ther
[1622] Yanli L, Wenwu S, Jia L, et al. (2020) Clinical features and progression of acute respiratory distress syndrome in coronavirus disease 2019. MedRxiv: 2020.2002.2017.20024166. 10.1101/2020.02.17.20024166
[1623] Zhang JJ, Dong X, Cao YY, et al. (2020) Clinical characteristics of 140 patients infected with SARS-CoV-2 in Wuhan, China. Allergy. 10.1111/all.14238
[1624] Zheng Y, Xiong C, Liu Y, et al. (2020) Epidemiological and clinical characteristics analysis of COVID-19 in the surrounding areas of Wuhan, Hubei Province in 2020. Pharmacol Res 157: 104821. 10.1016/j.phrs.2020.104821
[1625] Alkundi, Mahmoud, Musa, Naveed, Alshawwaf (2020) Clinical characteristics and outcomes of COVID-19 hospitalized patients with diabetes in the United Kingdom: a retrospective single centre study. (Special issue on diabetes and COVID-19: the IDF perspective.). Diabetes Research and Clinical Practice 16525
[1626] Al-Kuraishy HM, Al-Gareeb AI, Alblihed M, Cruz-Martins N, Batiha GE (2021) COVID-19 and Risk of Acute Ischemic Stroke and Acute Lung Injury in Patients With Type II Diabetes Mellitus: The Anti-inflammatory Role of Metformin. Front Med (Lausanne) 8: 644295. 10.3389/fmed.2021.644295
[1627] Belice T, Demir I (2020) The gender differences as a risk factor in diabetic patients with COVID-19. Iran J Microbiol 12(6): 625-628. 10.18502/ijm.v12i6.5038
[1628] Bhatti R, Khamis AH, Khatib S, Shiraz S, Matfin G (2020) Clinical Characteristics and Outcomes of Patients With Diabetes Admitted for COVID-19 Treatment in Dubai: Single-Centre Cross-Sectional Study. JMIR Public Health Surveill 6(4): e22471-e22471
[1629] Cai YL, Shi SB, Yang F, et al. (2020) Fasting blood glucose level is a predictor of mortality in patients with COVID-19 independent of diabetes history. Diabetes Research and Clinical Practice 169. 10.1016/j.diabres.2020.108437
[1630] Chacko SR, DeJoy R, 3rd, Lo KB, et al. (2021) Association of Pre-Admission Statin Use With Reduced In-Hospital Mortality in COVID-19. Am J Med Sci. 10.1016/j.amjms.2021.03.001
[1631] Chen ZS, Zhang FR, Hu WH, et al. (2021) Laboratory markers associated with COVID-19 progression in patients with or without comorbidity: A retrospective study. Journal of Clinical Laboratory Analysis 35(1). 10.1002/jcla.23644
[1632] Dai W, Wu J, Li TF, et al. (2021) Clinical outcomes for COVID-19 patients with diabetes mellitus treated with convalescent plasma transfusion in Wuhan, China. Journal of Medical Virology 93(4): 2321-2331. 10.1002/jmv.26712
[1633] Dyusupova A, Faizova R, Yurkovskaya O, et al. (2021) Clinical characteristics and risk factors for disease severity and mortality of COVID-19 patients with diabetes mellitus in Kazakhstan: A nationwide study. Heliyon 7(3): e06561. 10.1016/j.heliyon.2021.e06561
[1634] Elemam NM, Hannawi H, Salmi IA, Naeem KB, Alokaily F, Hannawi S (2021) Diabetes mellitus as a comorbidity in COVID-19 infection in the United Arab Emirates. Saudi Med J 42(2): 170-180. 10.15537/smj.2021.2.25700
[1635] Fei L, Yue C, Chao G, et al. (2021) Effects of Diabetes and Blood Glucose on COVID-19 Mortality: A Retrospective Observational Study. medRxiv. 10.1101/2021.01.21.20202119
[1636] Guo T, Shen Q, Ouyang X, et al. (2021) Clinical Findings in Diabetes Mellitus Patients with COVID-19. J Diabetes Res 2021: 7830136. 10.1155/2021/7830136
[1637] Hayek S, Ben-Shlomo Y, Balicer R, et al. (2021) Preinfection glycaemic control and disease severity among patients with type 2 diabetes and COVID-19: A retrospective, cohort study. Diabetes Obes Metab. 10.1111/dom.14393
[1638] Jiang N, Liu YN, Bao J, et al. (2021) Clinical features and risk factors associated with severe COVID-19 patients in China. Chin Med J (Engl) 134(8): 944-953. 10.1097/cm9.0000000000001466
[1639] Khadija H, Saira A, Arhsee K, Touseef K, Zufana N, Tahani A (2020) The Clinical Characteristics and Outcomes of COVID-19 Infections in Patients with Diabetes at a Tertiary Care Center in the UAE. Journal of diabetes & metabolism 26(4). 10.1159/000512232
[1640] Kshanti IA, Aji G, Eprilliawati M, et al. (2020) Clinical presentation and outcome of covid-19 infection in type 2 diabetes mellitus: A preliminary data from a tertiary hospital in jakarta during the early days of the pandemic. Bali Medical Journal 9(3)
[1641] Liu D, Wang Y, Zhao B, et al. (2021) Overall reduced lymphocyte especially T and B subsets closely related to the poor prognosis and the disease severity in severe patients with COVID-19 and diabetes mellitus. Diabetol Metab Syndr 13(1): 5. 10.1186/s13098-020-00622-3
[1642] Llanera DK, Rebekah W, Haika S, et al. (2021) Clinical Characteristics of COVID-19 Patients in a Regional Population with Diabetes Mellitus: The ACCREDIT Study. SSRN. 10.2139/ssrn.3785999

[1643] Malik SUF, Chowdhury PA, Hakim A, Islam MS, Alam MJ, Azad AK (2021) Blood biochemical parameters for assessment of COVID-19 in diabetic and non-diabetic subjects: a cross-sectional study. Int J Environ Health Res: 1-14. 10.1080/09603123.2021.1879741
[1644] Manciuc C, Nemescu D, Vata A, Lacatusu GA (2021) SARS-CoV-2 infection and diabetes mellitus: A North Eastern Romanian experience. Exp Ther Med 21(3): 279. 10.3892/etm.2021.9710
[1645] María Í, Patricia P-M, Pablo V-B, et al. (2021) ACE gene variants rise the risk of severe COVID-19 in patients with hypertension, dyslipidemia or diabetes. A pilot study. medRxiv. 10.1101/2021.03.24.21253576
[1646] Md. Shahed M, Abdullah Al M, Mohammad Sorowar H (2021) Comparing between survived and deceased patients with Diabetes Mellitus and COVID-19 in Bangladesh: A cross- sectional study from COVID-19 dedicated hospital. medRxiv. 10.1101/2021.04.04.21254884
[1647] Mithal A, Jevalikar G, Sharma R, et al. (2021) High prevalence of diabetes and other comorbidities in hospitalized patients with COVID-19 in Delhi, India, and their association with outcomes. Diabetes Metab Syndr 15(1): 169-175. 10.1016/j.dsx.2020.12.029
[1648] Moftakhar L, Moftakhar P, Piraee E, Ghaem H, Valipour A, Azarbakhsh H (2021) Epidemiological characteristics and outcomes of COVID-19 in diabetic versus non-diabetic patients. Int J Diabetes Dev Ctries: 1-6. 10.1007/s13410-021-00930-y
[1649] Ramos-Yataco A, Ramos-Yataco A, Farfan-Garcia RC, et al. (2021) Dka in patients with pre-existing type 2 diabetes mellitus related to covid-19: A case series. Endocrinol Diabetes Metab Case Rep 2021(1). 10.1530/EDM-20-0148
[1650] Randhawa G, Syed KA, Singh K, et al. (2021) The relationship between obesity, hemoglobin A1c and the severity of COVID-19 at an urban tertiary care center in New York City: a retrospective cohort study. BMJ Open 11(1): e044526. 10.1136/bmjopen-2020-044526
[1651] Ricchio M, Tassone B, Pelle MC, et al. (2021) Characteristics, Management, and Outcomes of Elderly Patients with Diabetes in a Covid-19 Unit: Lessons Learned from a Pilot Study. Medicina (Kaunas) 57(4). 10.3390/medicina57040341
[1652] Shrestha E, Charkviani M, Musurakis C, et al. (2021) Type 2 diabetes is associated with increased risk of critical respiratory illness in patients COVID-19 in a community hospital. Obes Med 22: 100316. 10.1016/j.obmed.2020.100316
[1653] Soni M, Gopalakrishnan R, Vaishya R, Prabu P (2020) D-dimer level is a useful predictor for mortality in patients with COVID-19: Analysis of 483 cases. Diabetes Metab Syndr 14(6): 2245-2249. 10.1016/j.dsx.2020.11.007
[1654] Sourij H, Aziz F, Brauer A, et al. (2021) COVID-19 fatality prediction in people with diabetes and prediabetes using a simple score upon hospital admission. Diabetes Obesity & Metabolism 23(2): 589-598. 10.1111/dom.14256
[1655] Sun Y, Zhao R, Hu Z, et al. (2020) Differences in the Clinical and Hematological Characteristics of COVID-19 Patients with and without Type 2 Diabetes. J Diabetes Res 2020: 1038585. 10.1155/2020/1038585
[1656] Varma A, Dergaa I, Mohammed AR, et al. (2021) Covid-19 and diabetes in primary care - How do hematological parameters present in this cohort? Expert Rev Endocrinol Metab: 1-7. 10.1080/17446651.2021.1909472
[1657] Wang F, Yang Y, Dong K, et al. (2020) CLINICAL CHARACTERISTICS OF 28 PATIENTS WITH DIABETES AND COVID-19 IN WUHAN, CHINA. Endocrine Practice 26(6): 668-674. 10.4158/ep-2020-0108
[1658] Wang Z, Du Z, Zhao X, Guo F, Wang T, Zhu F (2021) Determinants of Increased Fibrinogen in COVID-19 Patients With and Without Diabetes and Impaired Fasting Glucose. Clin Appl Thromb Hemost 27: 1076029621996445. 10.1177/1076029621996445
[1659] Carlson N, Nelveg-Kristensen KE, Freese Ballegaard E, et al. (2021) Increased vulnerability to COVID-19 in chronic kidney disease. J Intern Med. 10.1111/joim.13239
[1660] Ramesh J, Reddy SLS, Rajesh M, Varghese J (2021) Evaluation of simple and cost-effective immuno- haematological markers to predict outcome in hospitalized severe COVID-19 patients, with a focus on diabetes mellitus - A retrospective study in Andhra Pradesh, India. Diabetes Metab Syndr 15(3): 739-745. 10.1016/j.dsx.2021.03.025
[1661] Demidova TY, Lobanova KG, Oynotkinova OS, Perekhodov SN, Antsiferov MB (2021) Clinical and laboratory characteristics of patients with COVID-19 and concomitant type 2 diabetes. Cardiovasc Ther Prev 20(1): 47-58. 10.15829/1728-8800-2021-2750
[1662] Maurio González H, Luis Miguel Osoria M, Yoydel Santines A, Leandro León R, Annis Almenares G, Vilma Rosa Pérez R (2020) Caracterización de pacientes diabéticos contagiados por la COVID-19 ingresados en el hospital OGR. Torino, Italia. 24(3)
[1663] Wang Z, He J, Cheng Y, Xu J, Xiang G, Yue L (2020) Clinical characteristics and outcomes of COVID-19 infected patients with type 2 diabetes mellitus. Chinese Journal of Endocrinology and Metabolism 36(8)

[1664] Zhang P, Wang M, Wang Y, et al. (2020) Risk factors associated with the progression of COVID-19 in elderly diabetes patients. Diabetes Res Clin Pract 171: 108550. 10.1016/j.diabres.2020.108550

[1665] Adil RS, Thaer AH, Mohammed HF, Khwam RH (2021) A Comprehensive Analysis of Biochemical Indexes in Patients with COVID-19 Infection. SSRN

[1666] Ahmed AS, Alotaibi WS, Aldubayan MA, et al. (2021) Factors Affecting the Incidence, Progression, and Severity of COVID-19 in Type 1 Diabetes Mellitus. Biomed Res Int 2021: 1676914. 10.1155/2021/1676914

[1667] AlanÍS-Naranjo JM, Hammeken-Larrondo EF, Silva-Arroyo MG (2021) Diabetic Ketoacidosis as a Feature of COVID-19 in Patients with Diabetes: A Case Series. Revista Mexicana de Endocrinología, Metabolismo y Nutrición 8: 1-10. 10.24875/rme.20000103

[1668] Ali A-R, Hamed A-R (2021) Diabetic Ketoacidosis among COVID-19 Patients Admitted to Suhar Hospital, Oman: Clinical Characteristics and Outcomes. Journal of diabetes & metabolism 12(4)

[1669] Anton IC, Botnariu EG, Coculescu EC, Boanca M, Mititelu-Tartau L (2021) PARTICULAR ASPECTS OF EVOLUTION OF SARS-COV-2 INFECTION IN TYPE 2 DIABETIC PATIENTS. Romanian Journal of Legal Medicine 29(1): 53-59. 10.4323/rjlm.2021.53

[1670] Bonyek-Silva I, Machado AFA, Cerqueira-Silva T, et al. (2021) LTB₄-Driven Inflammation and Increased Expression of <i>ALOX5</i>/<i>ACE2</i> During Severe COVID-19 in Individuals With Diabetes. Diabetes 70(9): 2120-2130. 10.2337/db20-1260

[1671] Ciarambino T, Ciaburri F, Paoli VD, Caruso G, Giordano M, D'Avino M (2021) Arterial Hypertension and Diabetes Mellitus in COVID-19 Patients: What Is Known by Gender Differences? J Clin Med 10(16). 10.3390/jcm10163740

[1672] Corcillo A, Cohen S, Game D, Karalliedde J (2020) High prevalence of Afro-Caribbean ethnicity and hypoglycaemia in patients with diabetes and end stage renal disease hospitalised with COVID-19. Nephrology (Carlton, Vic) 26(3): 252-254. 10.1111/nep.13809

[1673] Davoudi Z, Darazam IA, Saberian F, et al. (2021) Clinical course and outcome in diabetic patients with COVID-19. Immunopathologia Persa 7(2): 8-8. 10.34172/ipp.2021.23

[1674] de Mélo KC, de Souza Lôbo JL, da Silva Junior AG, do Carmo RF, de Souza CDF (2021) Obesity and the COVID-19: Analysis of the clinical and epidemiological profiles of 138 individuals. Rev Assoc Med Bras (1992) 67Suppl 1(Suppl 1): 29-34. 10.1590/1806-9282.67.Suppl1.20200725

[1675] Donamou J, Bangoura A, Camara LM, et al. (2021) Epidemiological and clinical characteristics of COVID-19 patients admitted to the intensive care unit of Donka hospital in Conakry, Guinea: Descriptive study of the first 140 hospitalised cases. Anesthesie et Reanimation. 10.1016/j.anrea.2021.01.001

[1676] Estedlal A, Jeddi M, Heydari ST, Jahromi MG, Dabbaghmanesh MH (2021) Impacts of diabetes mellitus on clinical and para-clinical parameters among COVID-19 patients. J Diabetes Metab Disord 20(2): 1-9. 10.1007/s40200-021-00844-w

[1677] Fosse-Edorh S, Mandereau-Bruno L (2021) Hospitalizations for COVID-19 in the first half of 2020 in people pharmacologically treated for diabetes in France. Bulletin Epidemiologique Hebdomadaire 4(19): 2-8

[1678] Gupta PSTHARBK (2021) Association of Glycosylated hemoglobin with Radiological severity, Systemic inflammation and Hypercoagulability in COVID -19 patients in a hospital from Central India: A Monocentric Retrospective Observational study. Asian Journal of Medical Sciences 12(6): 1-5. 10.3126/ajms.v12i6.33574

[1679] Íñiguez M, Pérez-Matute P, Villoslada-Blanco P, et al. (2021) ACE Gene Variants Rise the Risk of Severe COVID-19 in Patients With Hypertension, Dyslipidemia or Diabetes: A Spanish Pilot Study. Front Endocrinol (Lausanne) 12: 688071. 10.3389/fendo.2021.688071

[1680] Jose S-O, Ofelia A-G, Guillermo de A-J, et al. (2021) PROBABILITY OF HOSPITALIZATION AND DEATH AMONG COVID-19 PATIENTS WITH COMORBIDITY DURING OUTBREAKS OCCURRING IN MEXICO CITY. medRxiv. 10.1101/2021.12.07.21267287

[1681] Kempegowda PMEJAWLTLZDHCJAKMANPBS (2020) Effect of COVID-19 Infection on the Clinical Course of Diabetic Ketoacidosis (DKA) in People with Type 1 and Type 2 Diabetes (preprint). In:

[1682] Khan MUF, Ali BR, Mohammed HQ, et al. (2022) Serum level estimation of some biomarkers in diabetic and non-diabetic COVID-19 infected patients. Appl Nanosci: 1-8. 10.1007/s13204-021-02167-x

[1683] Kishan JH, Kumar VS (2021) Role of glycaemic control in predicting severity of illness in COVID 19 patients with diabetes mellitus. J Clin Diagn Res 15(5): 25-27. 10.7860/JCDR/2021/48021.14934

[1684] Kumar B, Mittal M, Gopalakrishnan M, Garg MK, Misra S (2021) Effect of plasma glucose at admission on COVID-19 mortality: experience from a tertiary hospital. Endocr Connect 10(6): 589-598. 10.1530/ec-21-0086

[1685] Leon-Abarca JA, Portmann-Baracco A, Bryce-Alberti M, et al. (2021) Diabetes increases the risk of COVID-19 in an altitude dependent manner: An analysis of 1,280,806 Mexican patients. PLoS One 16(8): e0255144. 10.1371/journal.pone.0255144
[1686] Maltezou HC, Pavli A, Tsonou P, et al. (2022) Role of diabetes mellitus in the clinical course and outcome of SARS-CoV-2 infected patients. Hormones (Athens). 10.1007/s42000-021-00342-x
[1687] Minuljo TT, Anindita YPC, Seno HNH, Pemayun TGD, Sofro MAU (2020) Karakteristik Dan Keluaran Pasien COVID-19 Dengan Dm Di Rs Umum Pusat Dr. Kariadi (Tinjauan Pasien Periode Maret-Juli 2020). In. Vol 7
[1688] Miri C, Charii H, Bouazzaoui MA, et al. (2021) D-dimer Level and Diabetes in the COVID-19 Infection. Clin Appl Thromb Hemost 27: 10760296211045902. 10.1177/10760296211045902
[1689] Mirsoleymani S, Taherifard E, Taherifard E, et al. (2021) Predictors of mortality among covid-19 patients with or without comorbid diabetes mellitus. Acta Medica Iranica 59(7): 393-399. 10.18502/acta.v59i7.7018
[1690] Morshed MSAMAHMS (2021) Comparing between survived and deceased patients with Diabetes Mellitus and COVID-19 in Bangladesh: A cross sectional study from a COVID-19 dedicated hospital. In:
[1691] Nasr El-Din A, Ata KAE-S, Abdel-Gawad AR, Fahmy NF (2021) Impact of High Serum Levels of MMP-7, MMP-9, TGF-ß and PDGF Macrophage Activation Markers on Severity of COVID-19 in Obese-Diabetic Patients. Infect Drug Resist 14: 4015-4025. 10.2147/IDR.S329004
[1692] Patel U, Deluxe L, Salama C, et al. (2021) Evaluation of Characteristics and Outcomes for Patients with Diabetic Ketoacidosis (DKA) With and Without COVID-19 in Elmhurst Queens During Similar Three-Month Periods in 2019 and 2020. Cureus 13(7): e16427. 10.7759/cureus.16427
[1693] Poaty H, Poaty GE, Ndziessi G, et al. (2021) Diabetes and covid-19 in congolese patients. African Health Sciences 21(3): 1100-1106. 10.4314/ahs.v21i3.18
[1694] Rengarajan L, Kempegowda P, Melson E, et al. (2021) Effect of covid-19 infection on diabetic ketoacidosis (DKA) in people with type 1 and type 2 diabetes. Diabetic Medicine 38
[1695] Schiller M, Solger K, Leipold S, Kerl HU, Kick W (2021) Diabetes-associated nephropathy and obesity influence COVID-19 outcome in type 2 diabetes patients. J Community Hosp Intern Med Perspect 11(5): 590-596. 10.1080/20009666.2021.1957555
[1696] Tronko MD, Cherviakova SA, Pushkarev VBYB, Kovzun OI, Pushkarev VM, Sokolova LK (2021) Apolipoprotein A1 Level in Plasma of Patients with Diabetes and Diabetic Patients with COVID-19 as a Possible Marker of Disease (preprint). In:
[1697] Tronko MD, Cherviakova SA, Vladimir P, et al. (2021) Apolipoprotein A1 Level in Plasma of Patients with Diabetes and Diabetic Patients with COVID-19 as a Possible Marker of Disease. SSRN
[1698] Uchihara M, Bouchi R, Kodani N, et al. (2022) Impact of newly diagnosed diabetes on coronavirus disease 2019 severity and hyperglycemia. J Diabetes Investig. 10.1111/jdi.13754
[1699] Unluguzel Ustun G, Keskin A, Aci R, Arslanbek Erdem M, Ari M (2021) Association between Hb A(1c) and Severity of COVID-19 Patients. Hemoglobin 45(2): 124-128. 10.1080/03630269.2021.1926278
[1700] Usman A, Bliden KP, Cho A, et al. (2022) Metformin use in patients hospitalized with COVID-19: lower inflammation, oxidative stress, and thrombotic risk markers and better clinical outcomes. J Thromb Thrombolysis: 1-9. 10.1007/s11239-022-02631-7
[1701] Vultur MA, Hedi-Katalin S, Ianosi MB, et al. (2021) ASSOCIATION BETWEEN DIABETES MELLITUS AND SARS-CoV-2 INFECTION - A RETROSPECTIVE STUDY IN THE PULMONOLOGY CLINIC OF TARGU MURE. Medical-Surgical Journal-Revista Medico-Chirurgicala 125(3): 376-385. 10.22551/msj.2021.03.08
[1702] Xie W, Wu N, Wang B, et al. (2021) Fasting plasma glucose and glucose fluctuation are associated with COVID-19 prognosis regardless of pre-existing diabetes. Diabetes Res Clin Pract 180: 109041. 10.1016/j.diabres.2021.109041
[1703] М. В Ш, И. В К, З. А К, А. В Ж, Н. Г М (2021) Повышенный уровень гликированного гемоглобина (HbA 1c) у больных с COVID-19 является маркером тяжести течения инфекции, но не индикатором предшествующего сахарного диабета. 23(6). 10.14341/DM12702-9873
[1704] Chacko SR, DeJoy R, Lo KB, et al. (2021) Association of Pre-Admission Statin Mortality in COVID-19. American Journal of the Medical Sciences 361(6): 725-730
[1705] Pavithra S, Lavanya S, Vaishnavi P, Rosario AR, Priyadharshini A, Ahalya SP (2021) The Impact of COVID-19 on Diabetes Mellitus and Hypertension: A Retrospective Study. Journal of Pharmaceutical Research International 33(43B): 68-77. 10.9734/JPRI/2021/v33i43B32526
[1706] Chao WC, Tseng CH, Wu CL, Shih SJ, Yi CY, Chan MC (2020) Higher glycemic variability within the first day of ICU admission is associated with increased 30-day mortality in ICU patients with sepsis. Ann Intensive Care 10(1): 17. 10.1186/s13613-020-0635-3

[1707] Al Argan R, Alkhafaji D, Al Elq A, et al. (2021) The Impact of Diabetes Mellitus and Hyperglycemia on the Severity and Outcome of Patients with COVID-19 Disease: A Single-Center Experience. Int J Gen Med 14: 9445-9457. 10.2147/ijgm.S338800
[1708] Deng YP, Xie W, Liu T, et al. (2021) Association of diabetes with severity and mortality in hospitalized patients with COVID-19 in Wuhan, China: a single-centered, retrospective study. Arch Endocrinol Metab 65(5): 596-608. 10.20945/2359-3997000000384
[1709] Sifuentes-Osornio JA-GOD-A-JGD-D-L-SJH-LEB-PHHLL-AOR-HAR-T PROBABILITY OF HOSPITALIZATION AND DEATH AMONG COVID-19 PATIENTS WITH COMORBIDITY DURING OUTBREAKS OCCURRING IN MEXICO CITY (preprint). In:
[1710] Santos LG, Baggio JAO, Leal TC, et al. (2021) Prevalence of Systemic Arterial Hypertension and Diabetes Mellitus in Individuals with COVID-19: A Retrospective Study of Deaths in Pernambuco, Brazil. Arq Bras Cardiol 117(2): 416-422. 10.36660/abc.20200885
[1711] Uchihara M, Bouchi R, Kodani N, et al. (2022) Impact of newly diagnosed diabetes on COVID-19 severity and hyperglycemia. Journal of Diabetes Investigation. 10.1111/jdi.13754
[1712] Merzon E, Green I, Shpigelman M, et al. (2021) [HEMOGLOBIN A1C AND MORTALITY FROM COVID-19]. Harefuah 160(10): 645-650
[1713] Abu-Jamous B, Anisimovich A, Baxter J, et al. (2020) Associations of comorbidities and medications with COVID-19 outcome: A retrospective analysis of real-world evidence data. medRxiv. 10.1101/2020.08.20.20174169
[1714] Al-Sofiani ME, Albunyan S, Alguwaihes AM, Kalyani RR, Golden SH, Alfadda A (2021) Determinants of mental health outcomes among people with and without diabetes during the COVID-19 outbreak in the Arab Gulf Region. J Diabetes 13(4): 339-352. 10.1111/1753-0407.13149
[1715] Belikina DV, Malysheva ES, Petrov AV, et al. (2020) COVID-19 in patients with diabetes: Clinical course, metabolic status, inflammation, and coagulation disorder. Sovrem Tehnol Med 12(5): 6-18. 10.17691/stm2020.12.5.01
[1716] Bowe B, Cai M, Xie Y, Gibson AK, Maddukuri G, Al-Aly Z (2020) Acute Kidney Injury in a National Cohort of Hospitalized US Veterans with COVID-19. Clin J Am Soc Nephrol. 10.2215/cjn.09610620
[1717] Chen C, Li J, Liu L, et al. (2020) Relations of Treatments for Cardiometabolic Co-Morbidities with Critical Outcomes and Mortality in Hospitalized Patients with Coronavirus Disease 2019 (COVID-19): A Retrospective Cohort Study. SSRN. 10.2139/ssrn.3696829
[1718] Cheng X, Liu YM, Li H, et al. (2020) Metformin Is Associated with Higher Incidence of Acidosis, but Not Mortality, in Individuals with COVID-19 and Pre-existing Type 2 Diabetes. Cell Metab 32(4): 537-547.e533. 10.1016/j.cmet.2020.08.013
[1719] Cheng X, Xin S, Chen Y, et al. (2021) Effects of metformin, insulin on COVID-19 patients with pre-existed type 2 diabetes: A multicentral retrospective study. Life Sci 275: 119371. 10.1016/j.lfs.2021.119371
[1720] De Abajo FJ, Antonio R-M, Sara R-M, Victoria L, Alberto G-L, Group M-ACS (2021) IN-HOSPITAL CONTINUATION WITH ANGIOTENSIN RECEPTOR BLOCKERS IS ASSOCIATED WITH A LOWER MORTALITY RATE THAN CONTINUATION WITH ANGIOTENSIN CONVERTING ENZYME INHIBITORS IN COVID-19 PATIENTS A RETROSPECTIVE COHORT STUDY. medRxiv. 10.1101/2021.02.01.21250853
[1721] Galvez-Romero JL, Palmeros-Rojas O, Real-Ramírez FA, et al. (2020) Cyclosporine A plus low-dose steroid treatment in COVID-19 improves clinical outcomes in patients with moderate to severe disease. A pilot study. J Intern Med. 10.1111/joim.13223
[1722] Israelsen SB, Pottegård A, Sandholdt H, Madsbad S, Thomsen RW, Benfield T (2021) Comparable COVID-19 outcomes with current use of GLP-1 receptor agonists, DPP-4 inhibitors or SGLT-2 inhibitors among patients with diabetes who tested positive for SARS-CoV-2. Diabetes Obes Metab. 10.1111/dom.14329
[1723] Jiang N, Chen Z, Yin X, et al. (2020) Association of metformin with mortality or ARDS in patients with COVID-19 and type 2 diabetes: a retrospective cohort study. Diabetes Res Clin Pract: 108619. 10.1016/j.diabres.2020.108619
[1724] Huang N, Pérez P, Kato T, et al. (2021) SARS-CoV-2 infection of the oral cavity and saliva. Nat Med. 10.1038/s41591-021-01296-8
[1725] Saeed O, Castagna F, Agalliu I, et al. (2020) Statin Use and In-Hospital Mortality in Patients With Diabetes Mellitus and COVID-19. J Am Heart Assoc 9(24): e018475. 10.1161/jaha.120.018475
[1726] Shen Y, Fan X, Zhang L, et al. (2021) Thresholds of Glycemia and the Outcomes of COVID-19 Complicated With Diabetes: A Retrospective Exploratory Study Using Continuous Glucose Monitoring. Diabetes Care 44(4): 976-982. 10.2337/dc20-1448
[1727] Yang L, Xu D, Tan Y, et al. (2020) Association Between Cystatin C, Cystatin C Rangeability and Mortality of COVID-19 Patients With or Without Type 2 Diabetes Mellitus: An Opportunistic Retrospective Analysis. ResearchSquare. 10.21203/rs.3.rs-112220/v1

[1728] Yu B, Li C, Sun Y, Wang DW (2020) Insulin Treatment Is Associated with Increased Mortality in Patients with COVID-19 and Type 2 Diabetes. Cell Metab. 10.1016/j.cmet.2020.11.014
[1729] Zhou JH, Wu B, Wang WX, et al. (2020) No significant association between dipeptidyl peptidase-4 inhibitors and adverse outcomes of COVID-19. World J Clin Cases 8(22): 5576-5588. 10.12998/wjcc.v8.i22.5576
[1730] Demidova TY, Lobanova KG, Perekhodov SN, Antsiferov MB (2021) Clinical characteristics of patients with covid-19 depending on the treatment received and the presence of type 2 diabetes mellitus. Diabetes Mellitus 24(3): 231-242. 10.14341/DM12764
[1731] Demidova TY, Lobanova KG, Perekhodov SN, Antsiferov MB, Oynotkinova OS (2021) Retrospective analysis of clinical outcomes of patients with COVID-19 depending on receiving antihypertensive, lipid -lowering and antihypertensive therapy. Terapevticheskii Arkhiv 93(10): 1193-1202. 10.26442/00403660.2021.10.201072
[1732] Vahedian-Azimi A, Rahimibashar F, Najafi A, et al. (2021) Associastion of In-hospital Use of Statins, Aspirin, and Renin-Angiotensin-Aldosterone Inhibitors with Mortality and ICU Admission Due to COVID-19. Adv Exp Med Biol 1327: 205-214. 10.1007/978-3-030-71697-4_17
[1733] Yang L, Xu D, Tan Y, et al. (2021) Association Between the Concentration and Rangeability of Cystatin C and Mortality of COVID-19 Patients With or Without Type 2 Diabetes Mellitus: A Retrospective Analysis. Front Endocrinol (Lausanne) 12: 642452. 10.3389/fendo.2021.642452
[1734] Blanchard C, Perennec T, Smati S, et al. (2021) History of bariatric surgery and COVID-19 outcomes in patients with type 2 diabetes: Results from the CORONADO study. Obesity (Silver Spring). 10.1002/oby.23314
[1735] Pan M, Vasbinder A, Anderson E, et al. (2021) Angiotensin-Converting Enzyme Inhibitors, Angiotensin II Receptor Blockers, and Outcomes in Patients Hospitalized for COVID-19. J Am Heart Assoc 10(24): e023535. 10.1161/jaha.121.023535
[1736] Lohia P, Kapur S, Benjaram S, et al. (2021) Statins and clinical outcomes in hospitalized COVID-19 patients with and without Diabetes Mellitus: a retrospective cohort study with propensity score matching. Cardiovasc Diabetol 20(1): 140. 10.1186/s12933-021-01336-0
[1737] Al-Salameh A, Bennis Y, Cariou B, Lalau JD (2021) The association between metformin treatment and COVID-19 outcomes according to metformin continuation during hospitalisation. Diabetes Metab 47(6): 101297. 10.1016/j.diabet.2021.101297
[1738] Ahmed OJ, Ai-Wasiti EA, Jamil D, Al-Aubaidy HA (2021) Changes in the Levels of Biochemical Markers Following Coronavirus Infection in Patients with Liver Disease, Renal Disease and Diabetes Mellitus as Compared to Control Participants: A Cross Sectional Study. Journal of Pharmaceutical Research International 33(30B): 141-148. 10.9734/JPRI/2021/v33i30B31648
[1739] Elrayess MA, Cyprian FS, Abdallah AM, et al. (2021) Metabolic Signatures of Type 2 Diabetes Mellitus and Hypertension in COVID-19 Patients With Different Disease Severity. Front Med (Lausanne) 8: 788687. 10.3389/fmed.2021.788687
[1740] Goyal A, Gupta Y, Kalaivani M, P AP, Ambekar S, Tandon N (2021) SARS-CoV-2 seroprevalence in individuals with type 1 and type 2 diabetes compared to controls. Endocrine practice : official journal of the American College of Endocrinology and the American Association of Clinical Endocrinologists. 10.1016/j.eprac.2021.12.009
[1741] Hussein M, Fathy W, Eid RA, et al. (2021) Relative Frequency and Risk Factors of COVID-19 Related Headache in a Sample of Egyptian Population: A Hospital-Based Study. Pain Med 22(9): 2092-2099. 10.1093/pm/pnab020
[1742] Meher D, Kar S (2021) Effect of pandemic on Quality of life in Diabetics (QOLID) assessment: Data from a teaching hospital in Bhubaneswar, Odisha, India. J Family Med Prim Care 10(10): 3780-3784. 10.4103/jfmpc.jfmpc_287_21
[1743] Sandhu AT, Kohsaka S, Lin S, Woo CY, Goldstein MK, Heidenreich PA (2021) Renin-angiotensin-aldosterone system inhibitors and SARS-CoV-2 infection: an analysis from the veteran's affairs healthcare system. Am Heart J 240: 46-57. 10.1016/j.ahj.2021.06.004
[1744] Bhuiyan AFMT, Sudipta Deb N, Shuva D, et al. (2021) Clinical observation of high-flow nasal cannula (HFNC) with non-rebreather mask (NRM) use on severe or critically ill COVID-19 diabetic patients. medRxiv. 10.1101/2021.10.13.21264946
[1745] Ahirwar AK, Asia P, Sakarde A, Kaim K (2020) COVID -19 outbreak - Diabetes aspect and perspective. Curr Med Res Pract 10(3): 134. 10.1016/j.cmrp.2020.05.005
[1746] Deng F, Gao D, Ma X, et al. (2020) Corticosteroids in diabetes patients infected with COVID-19. Ir J Med Sci: 1-3. 10.1007/s11845-020-02287-3
[1747] Deng M, Jiang L, Ren Y, Liao J (2020) Can We Reduce Mortality of COVID-19 if We do Better in Glucose Control? Med Drug Discov 7: 100048. 10.1016/j.medidd.2020.100048
[1748] Fysekidis M, Cohen R, Al-Salameh A (2020) More Studies are Needed on the Link between Metformin and Decreased Mortality in Diabetic COVID-19 Patients. Am J Trop Med Hyg. 10.4269/ajtmh.20-0766

[1749] Idris I (2020) HYPERGLYCAEMIA AMONG PATIENTS WITH DIABETES IS ASSOCIATED WITH WORSE COVID-19 OUTCOME COMPARED WITH OPTIMAL GLUCOSE CONTROL. Diabetes Obesity & Metabolism 22(7): 1227-1227
[1750] Idris I (2020) STUDY DISSECTING COVID-19 RELATED MORTALITY IN PEOPLE WITH TYPE 1 AND TYPE 2 DIABETES. Diabetes Obesity & Metabolism 22(7): 1228-1228
[1751] Mittal S, Madan K, Mohan A (2020) COVID-19 and steroid therapy: Impact on diabetes. Prim Care Diabetes. 10.1016/j.pcd.2020.07.013
[1752] Montagnani A, Pieralli F, Gnerre P, et al. (2020) Diabetes and CoViD-19: Experience from the frontline of Internal Medicine wards in Italy. Diabetes Res Clin Pract 167: 108335. 10.1016/j.diabres.2020.108335
[1753] Zhang B, Liu S, Zhang L, Dong Y, Zhang S (2020) Admission fasting blood glucose predicts 30-day poor outcome in patients hospitalized for COVID-19 pneumonia. Diabetes Obes Metab. 10.1111/dom.14132
[1754] Akkus E, Sahin M (2020) Related molecular mechanisms of COVID-19, hypertension, and diabetes. Am J Physiol Endocrinol Metab 318(6): E881. 10.1152/ajpendo.00164.2020
[1755] Arlt W, Dekkers OM, Léger J, Semple RK (2020) Endocrinology in the time of COVID-19. Eur J Endocrinol 183(1): E1-e2. 10.1530/eje-20-0386
[1756] Assistance Publique - Hôpitaux de P (2020) Long-term Use of Drugs That Could Prevent the Risk of Serious COVID-19 Infections or Make it Worse. clinicaltrialsgov
[1757] Best F (2020) Time for a Change: Personal Experiences With COVID-19 and Diabetes. J Diabetes Sci Technol: 1932296820928083. 10.1177/1932296820928083
[1758] Bing LIU, Xuefei LIU, Min Z (2020) Paying close attention to diabetic patients with novel coronavirus infection. Chinese Journal of Endocrinology and Metabolism
[1759] Birkeland KI (2020) Some Lessons Learned About Diabetes and COVID-19 During the Early Stage of the Epidemic in Norway. J Diabetes Sci Technol: 1932296820929371. 10.1177/1932296820929371
[1760] Blanke CD (2020) In response: Diabetes is a risk factor for the progression and prognosis of COVID-19. Diabetes Metab Res Rev: e3331. 10.1002/dmrr.3331
[1761] Bloomgarden Z (2020) Is the type of diabetes treatment relevant to outcome of COVID-19? J Diabetes. 10.1111/1753-0407.13047
[1762] Caruso I, Giorgino F (2020) The diabetic lung: an easy target for SARS-CoV-2? Diabetes Metab Res Rev: e3346. 10.1002/dmrr.3346
[1763] Caruso P, Longo M, Esposito K, Maiorino MI (2020) Type 1 diabetes triggered by covid-19 pandemic: A potential outbreak? Diabetes Res Clin Pract 164: 108219. 10.1016/j.diabres.2020.108219
[1764] Catholic University of the Sacred H (2020) Prognostic Factors Keeping Track for Covid19 Pneumonia. clinicaltrialsgov
[1765] Ceriello A (2020) Hyperglycemia and the worse prognosis of COVID-19. Why a fast blood glucose control should be mandatory. Diabetes Res Clin Pract 163: 108186. 10.1016/j.diabres.2020.108186
[1766] Ceriello A, Schnell O (2020) COVID-19: Considerations of Diabetes and Cardiovascular Disease Management. J Diabetes Sci Technol: 1932296820930025. 10.1177/1932296820930025
[1767] Ceriello A, Stoian AP, Rizzo M (2020) COVID-19 and diabetes management: What should be considered? Diabetes Res Clin Pract 163: 108151. 10.1016/j.diabres.2020.108151
[1768] Chatterjee S (2020) SGLT-2 inhibitors for COVID-19 - A miracle waiting to happen or just another beat around the bush? Primary care diabetes. 10.1016/j.pcd.2020.05.013
[1769] Chee YJ, Ng SJH, Yeoh E (2020) Diabetic ketoacidosis precipitated by Covid-19 in a patient with newly diagnosed diabetes mellitus. Diabetes Res Clin Pract 164: 108166. 10.1016/j.diabres.2020.108166
[1770] Coppell KJ, Hall RM, Downie M, et al. (2020) Diabetes and COVID-19-the meeting of two pandemics: what are the concerns? The New Zealand medical journal 133(1514): 85-87
[1771] Dalan R (2020) Metformin, neutrophils and COVID-19 infection. Diabetes Res Clin Pract 164: 108230. 10.1016/j.diabres.2020.108230
[1772] de Galan BE (2020) Relative Limited Impact of COVID-19 on Diabetes: A Personal View. J Diabetes Sci Technol: 1932296820930270. 10.1177/1932296820930270
[1773] D'Marco L, Puchades MJ, Romero-Parra M, Gorriz JL (2020) Diabetic Kidney Disease and COVID-19: The Crash of Two Pandemics. Frontiers in medicine 7: 199. 10.3389/fmed.2020.00199
[1774] Fadini GP, Morieri ML, Longato E, Avogaro A (2020) Prevalence and impact of diabetes among people infected with SARS-CoV-2. J Endocrinol Invest 43(6): 867-869
[1775] Fang L, Karakiulakis G, Roth M (2020) Are patients with hypertension and diabetes mellitus at increased risk for COVID-19 infection? Lancet Respir Med 8(4): e21-e21
[1776] Gangopadhyay KK (2020) "Does having diabetes increase chances of contracting COVID-19 infection?". Diabetes Metab Syndr 14(5): 765-766. 10.1016/j.dsx.2020.05.048

[1777] Hamdy O, Gabbay RA (2020) Early Observation and Mitigation of Challenges in Diabetes Management of COVID-19 Patients in Critical Care Units. Diabetes Care. 10.2337/dc20-0944
[1778] Heaney AI, Griffin GD, Simon EL (2020) Newly diagnosed diabetes and diabetic ketoacidosis precipitated by COVID-19 infection. The American journal of emergency medicine. 10.1016/j.ajem.2020.05.114
[1779] Hussain A, do Vale Moreira NC (2020) Clinical considerations for patients with diabetes in times of COVID-19 pandemic. Diabetes Metab Syndr 14(4): 451-453. 10.1016/j.dsx.2020.03.005
[1780] Jagat JM, Kalyan KG, Subir R (2020) Use of pioglitazone in people with type 2 diabetes mellitus with coronavirus disease 2019 (COVID-19): Boon or bane? Diabetes Metab Syndr 14(5): 829-831. 10.1016/j.dsx.2020.06.015
[1781] Jennings GL (2020) Coronavirus disease 2019 (COVID-19): angiotensin-converting enzyme inhibitors, angiotensin II receptor blockers and cardiovascular disease. The Medical journal of Australia 212(11): 502-503.e501. 10.5694/mja2.50622
[1782] John TJ, John K (2020) What Is the Role of Angiotensin-Converting Enzyme 2 (ACE2) in COVID-19 Infection in Hypertensive Patients With Diabetes? The Canadian journal of cardiology 36(6): 969.e963. 10.1016/j.cjca.2020.03.049
[1783] Klonoff DC (2020) The Coronavirus 2019 Pandemic and Diabetes: An International Perspective. J Diabetes Sci Technol: 1932296820933075-1932296820933075
[1784] Krejner-Bienias A, Grzela K, Grzela T (2020) Do novel drugs for diabetes help in COVID-19? Another brick in the wall? J Diabetes. 10.1111/1753-0407.13050
[1785] Kumar Singh A, Singh R (2020) Is Metformin ahead in the race as a repurposed host-directed therapy for patients with diabetes and COVID-19? Diabetes Res Clin Pract: 108268. 10.1016/j.diabres.2020.108268
[1786] Vafadar Moradi E, Teimouri A, Rezaee R, et al. (2020) Increased age, neutrophil-to-lymphocyte ratio (NLR) and white blood cells count are associated with higher COVID-19 mortality. Am J Emerg Med 40: 11-14. 10.1016/j.ajem.2020.12.003
[1787] Melish JS (2020) Covid-19 and Diabetes in Hawaii. J Diabetes Sci Technol: 1932296820931127. 10.1177/1932296820931127
[1788] Nantes University H (2020) "Coronavirus SARS-CoV2 and Diabetes Outcomes". clinicaltrialsgov
[1789] Naruse K (2020) Does glycemic control rescue type 2 diabetic patients from COVID-19-related deaths? Journal of diabetes investigation. 10.1111/jdi.13320
[1790] Rubino F, Amiel SA, Zimmet P, et al. (2020) New-Onset Diabetes in Covid-19. N Engl J Med. 10.1056/NEJMc2018688
[1791] Sardu C, Gargiulo G, Esposito G, Paolisso G, Marfella R (2020) Impact of diabetes mellitus on clinical outcomes in patients affected by Covid-19. Cardiovasc Diabetol 19(1): 76-76
[1792] Shearer D (2020) COVID-19: The Underestimated Pandemic Impacting People With Diabetes. J Diabetes Sci Technol: 1932296820929389. 10.1177/1932296820929389
[1793] Stefan N, Birkenfeld AL, Schulze MB, Ludwig DS (2020) Obesity and impaired metabolic health in patients with COVID-19. Nature reviews Endocrinology 16(7): 341-342. 10.1038/s41574-020-0364-6
[1794] Tongji H (2020) A Single-center Retrospective Study to Find Prediction Factors Related to Mortality of COVID-19 Patients. clinicaltrialsgov
[1795] Yates T, Razieh C, Zaccardi F, Davies MJ, Khunti K (2020) Obesity and risk of COVID-19: analysis of UK biobank. Primary care diabetes. 10.1016/j.pcd.2020.05.011
[1796] Sardu C, D'Onofrio N, Balestrieri ML, et al. (2020) Outcomes in Patients With Hyperglycemia Affected by Covid-19: Can We Do More on Glycemic Control? Diabetes Care. 10.2337/dc20-0723
[1797] (2020) 377. Diabetes as a prognostic factor for mortality in Coronavirus Disease 19 (COVID-19): a systematic review and meta-analysis comprising 18,506 patients. Open Forum Infectious Diseases 7. 10.1093/OFID/OFAA439.572
[1798] Aihong W (2020) Timely blood glucose management for the outbreakof 2019 novel coronavirus disease (COVID-19) is urgently needed - Aihong Wang - PLA Strategic Support Force Characteristic Medical Center, Beijing, China. 4(2)
[1799] Ashktorab H, Pizuorno A, Dalivand MM, et al. (2021) Clinical Characteristics, Gastrointestinal Manifestations And Outcomes Of Covid-19 Patients Among Minorities In The Usa. Gastroenterology 160(6): S-217. 10.1016/S0016-5085(21)01260-9
[1800] Aso Y, Iijima T, Tomaru T, Jojima T, Usui I (2021) No Negative Impact of a National State of Emergency by COVID-19 Outbreak on Hemoglobin A1c Levels in Patients with Type 2 Diabetes Living in Semi-Rural Japan. Am J Med Sci. 10.1016/j.amjms.2021.03.010
[1801] Beliard K, Wilkes M, Yau M, Aluf A, Rapaport R (2021) SARS-CoV-2 infection-related diabetes mellitus. J Diabetes 13(6): 523-524. 10.1111/1753-0407.13173
[1802] Bellido V, Perez A (2020) Consequences of COVID-19 on people with diabetes. Endocrinologia Diabetes Y Nutricion 67(6): 355-356. 10.1016/j.endinu.2020.04.001

[1803] Bornstein SR, Rubino F, Ludwig B, et al. (2021) Consequences of the COVID-19 pandemic for patients with metabolic diseases. Nat Metab 3(3): 289-292. 10.1038/s42255-021-00358-y
[1804] Chernoff A (2021) Editor's Comment. Caution: The Intersection of Diabetes and COVID-19. Endocr Res 46(2): 55-56. 10.1080/07435800.2021.1894820
[1805] Choudhary P, Wilmot EG, Owen K, et al. (2020) A roadmap to recovery: ABCD recommendations on risk stratification of adult patients with diabetes in the post-COVID-19 era. Diabet Med: e14462. 10.1111/dme.14462
[1806] Chung SM, Ahn JH, Moon JS (2020) The Risk of Diabetes on Clinical Outcomes in Patients with Coronavirus Disease 2019: A Retrospective Cohort Study (Diabetes Metab J 2020;44:405-13). Diabetes & Metabolism Journal 44(4): 625-626. 10.4093/dmj.2020.0167
[1807] College GCSHM, Research C (2020) To determine whether diabetes in COVID-19 patients is associated with more complications. CTRI
[1808] Dardano A, Del Prato S (2021) Metformin: an inexpensive and effective treatment in people with diabetes and COVID-19? Lancet Healthy Longev 2(1): e6-e7. 10.1016/s2666-7568(20)30047-7
[1809] Dhurandhar NV, Akheruzzaman M, Hegde V (2020) Potentially modifiable factors to reduce severity of COVID-19 in type 2 diabetes. Nutr Diabetes 10(1): 30. 10.1038/s41387-020-00133-0
[1810] Dr.Nicole B, Dr.Anirban M (2020) Disproportionate COVID-19 Related Mortality Amongst African Americans in Four Southern States in the United States. medRxiv. 10.1101/2020.06.08.20124297
[1811] El Guendouz F, Derrou S, Benabdelfedil Y, Ouleghzal H, Safi S (2020) Diabetes mellitus and COVID-19: factors associated with bad prognosis. Clinical Diabetology 9(4): 205-207. 10.5603/dk.2020.0020
[1812] Fernández García L, Puentes Gutiérrez AB, García Bascones M (2020) Relationship between obesity, diabetes and ICU admission in COVID-19 patients. Med Clin (Engl Ed) 155(7): 314-315. 10.1016/j.medcle.2020.06.008
[1813] Fritschka E (2020) Diabetes begünstigt Nierenversagen bei COVID-19-Patienten : Corona-Pandemie -- Autor: E. Fritschka. MMW Fortschr Med 162(18): 31-31
[1814] Ganesan SK, Venkatratnam P, Mahendra J, Devarajan N (2020) Increased mortality of COVID-19 infected diabetes patients: role of furin proteases. Int J Obes (Lond) 44(12): 2486-2488. 10.1038/s41366-020-00670-9
[1815] Garcia LF, Gutierrez ABP, Bascones MG (2020) Relationship between obesity, diabetes and ICU admission in COVID-19 patients. Medicina Clinica 155(7): 314-315. 10.1016/j.medcli.2020.06.009
[1816] Gastélum-Cano JM, Islas-Osuna MA, Arízaga-Berber JA (2020) Higher values of fasting blood glucose and glycated hemoglobin are not associated with mortality in Covid-19 Mexican patients. Prim Care Diabetes. 10.1016/j.pcd.2020.10.014
[1817] Gentile S, Strollo F, Mambro A, Ceriello A (2020) COVID-19, ketoacidosis and new-onset diabetes: Are there possible cause and effect relationships among them? Diabetes Obes Metab 22(12): 2507-2508. 10.1111/dom.14170
[1818] Gulseth HL, Hjellvik V, Karlstad O, et al. (2020) Risk of morbidity and mortality of COVID-19 in subjects with diabetes in Norway. Diabetologia 63(SUPPL 1): S145-S145
[1819] Gupta K (2020) Covid-19 and Diabetes – A Bad Combination. J Pharm Sci Res 12(6): 805-806
[1820] Gutierrez-Ortiz C, Garcia-Villasante E, Pinedo-Torres I (2020) COVID-19 and Diabetic Foot: Will the Lamp Burn Bright? Int J Low Extrem Wounds 19(4): 391. 10.1177/1534734620948764
[1821] Harrington DM, O'Reilly M (2020) The reimagination of school-based physical activity research in the COVID-19 era. PLoS Med 17(8): e1003267. 10.1371/journal.pmed.1003267
[1822] Hillson R (2020) COVID-19: diabetes and death. A call to action. Practical Diabetes 37(3): 76-78. 10.1002/pdi.2271
[1823] Lambadiari V, Kousathana F, Raptis A, Katogiannis K, Kokkinos A, Ikonomidis I (2020) Pre-Existing Cytokine and NLRP3 Inflammasome Activation and Increased Vascular Permeability in Diabetes: A Possible Fatal Link With Worst COVID-19 Infection Outcomes? Front Immunol 11: 557235. 10.3389/fimmu.2020.557235
[1824] Legris P, Vaillard L, Nonciaux C, et al. (2021) Diabetes is not associated with COVID-19-related mortality in older institutionalized people. Diabetes Metab 47(3): 101235. 10.1016/j.diabet.2021.101235
[1825] Leng Y, Chen M, Dai M, et al. (2020) Minimize glycemic fluctuation decrease the risk of severe illness and death in patients with COVID-19. J Med Virol. 10.1002/jmv.26584
[1826] Liang X, Xu J, Xiao WW, Shi L, Yang HY The association of diabetes with COVID-19 disease severity: evidence from adjusted effect estimates. Hormones-International Journal of Endocrinology and Metabolism. 10.1007/s42000-020-00259-x
[1827] Ling P, Luo S, Zheng X, Cai G, Weng J (2021) Elevated fasting blood glucose within the first week of hospitalization was associated with progression to severe illness of COVID-19 in patients with

preexisting diabetes: A multicenter observational study. J Diabetes 13(1): 89-93. 10.1111/1753-0407.13121
[1828] Liu Y, Fu XX, Xie CG (2020) Efficacy and safety of chloroquine and hydroxychloroquine in the treatment of patients with COVID-19 combined with diabetes mellitus A protocol for systematic review and meta-analysis. Medicine 99(37). 10.1097/md.0000000000022031
[1829] Liu Y, Xie HY, Gao H, Xie CG (2020) Efficacy and safety of DPP-4 inhibitor in the treatment of patients with COVID-19 combined with diabetes mellitus A protocol for systematic review and meta-analysis. Medicine 99(41). 10.1097/md.0000000000022592
[1830] Liu Y, Yang Y, Chen YL, Zhou LY, Xiong Q, Xie CG (2020) The relationship between hyperglycemia and the infection of COVID-19 in diabetic patients A protocol for systematic review and meta-analysis. Medicine 99(36). 10.1097/md.0000000000021806
[1831] Lui DTW, Tan KCB (2020) Is metformin a miracle or a menace in COVID-19 patients with type 2 diabetes? J Diabetes Investig. 10.1111/jdi.13484
[1832] Malhotra A, Hepokoski M, McCowen KC, Shyy JYJ (2020) ACE2, Metformin, and COVID-19. Iscience 23(9). 10.1016/j.isci.2020.101425
[1833] Maria Panagiota A, George D, Panagiotis H (2020) The role of diabetes mellitus and obesity in COVID 19 patients. Pneumon 33(3)
[1834] McGovern AP, Thomas NJ, Vollmer SJ, Hattersley AT, Mateen BA, Dennis JM (2021) The disproportionate excess mortality risk of COVID-19 in younger people with diabetes warrants vaccination prioritisation. Diabetologia 64(5): 1184-1186. 10.1007/s00125-021-05404-8
[1835] Moin ASM, Al-Qaissi A, Sathyapalan T, Atkin SL, Butler AE (2021) Do biomarkers of COVID-19 severity simply reflect a stress response in type 2 diabetes: Biomarker response to hypoglycemia. Metabolism 114: 154417. 10.1016/j.metabol.2020.154417
[1836] Moon SJ, Rhee EJ, Lee WY, Yoon KH (2020) Independent Impact of Diabetes on the Severity of Coronavirus Disease 2019 in 5,307 Patients in South Korea: A Nationwide Cohort Study (Diabetes Metab J 2020;44:737-46). Diabetes Metab J 44(6): 942-943. 10.4093/dmj.2020.0266
[1837] Perez-Martinez P, Sanchez FJC, Gomez JC, Gomez-Huelgas R (2020) Solving one of the pieces of the puzzle: COVID-19 and type 2 diabetes. Revista Clinica Espanola 220(8): 507-510. 10.1016/j.rce.2020.05.003
[1838] Petrakis V, Panagopoulos P, Papazoglou D, Papanas N (2020) Diabetes Mellitus and Hypertension as Major Risk Factors of Mortality From Covid-19 Pneumonia. Exp clin endocrinol diabetes
[1839] Peykari N, Eybpoosh S, Safikhani H, Haghdoost AA, Tabatabaei-Malazy O, Larijani B (2020) Non-communicable Diseases and COVID-19; a double-edged sword A Special Communication from IRAN. J Diabetes Metab Disord: 1-5. 10.1007/s40200-020-00683-1
[1840] Pitocco D, Viti L, Santoliquido A, et al. (2021) Diabetic neuropathy: a risk factor for severe COVID-19? Acta Diabetol 58(5): 669-670. 10.1007/s00592-020-01658-7
[1841] Ploner T, Riedmann N, Hartig F, Ress C, Sourij H, Kaser S (2020) Characteristics of COVID-19 Patients with and without Diabetes mellitus: Data from a Outpatient Screening Clinic. Wiener Klinische Wochenschrift 132: S355-S355
[1842] Raony I, de Figueiredo CS (2020) Retinal outcomes of COVID-19: Possible role of CD147 and cytokine storm in infected patients with diabetes mellitus. Diabetes Research and Clinical Practice 165. 10.1016/j.diabres.2020.108280
[1843] Rego Lorca D, Rouco Fernandez A, Jimenez Santos M, Saenz-Frances F, Burgos-Blasco B, Donate Lopez J (2020) Bilateral retinal vein occlusion and diabetic retinopathy after COVID-19. Acta Ophthalmol. 10.1111/aos.14718
[1844] Riahi S, Lo KB, Anastasopoulou C, Rangaswami J (2021) Insulin Use and Poor COVID-19 Outcomes among Diabetes Patients: Association Not Necessarily Causation. Endocr Res 46(2): 53-54. 10.1080/07435800.2021.1894821
[1845] Sartore G, Ragazzi E, Faccin L, Lapolla A (2020) A role of glycation and methylation for SARS-CoV-2 infection in diabetes? Med Hypotheses 144: 110247-110247
[1846] Sathish T, de Mello GT, Cao YT (2021) Is newly diagnosed diabetes a stronger risk factor than pre-existing diabetes for COVID-19 severity? Journal of Diabetes 13(2): 177-178. 10.1111/1753-0407.13125
[1847] Schernthaner G (2021) Can glucose-lowering drugs affect the prognosis of COVID-19 in patients with type 2 diabetes? Lancet Diabetes Endocrinol 9(5): 251-252. 10.1016/s2213-8587(21)00059-0
[1848] Singh AK, Singh R (2020) Is metformin ahead in the race as a repurposed host-directed therapy for patients with diabetes and COVID-19? Diabetes Research and Clinical Practice 165. 10.1016/j.diabres.2020.108268
[1849] Sourij H, Aziz F, Brauer A, et al. (2020) COVID-19 and Diabetes Mellitus - Data from a multi-center Austrian Registry. Wiener Klinische Wochenschrift 132: S366-S367

[1850] Strandberg TE, Kivimäki M (2021) Increased mortality risk associated with statins in the CORONADO study. Diabetes Metab 47(3): 101250. 10.1016/j.diabet.2021.101250
[1851] Tadic M, Cuspidi C (2021) The influence of diabetes and hypertension on outcome in COVID-19 patients: Do we mix apples and oranges? J Clin Hypertens (Greenwich) 23(2): 235-237. 10.1111/jch.14145
[1852] Taher N, Huda MSB, Chowdhury TA (2020) COVID-19 and diabetes: What have we learned so far? Clinical Medicine 20(4): E87-E90. 10.7861/clinmed.2020-0261
[1853] Tascioglu D, Yalta K, Yetkin E (2020) Hypertension and diabetes mellitus in patients with COVID 19: a viewpoint on mortality. Cardiovasc Endocrinol Metab 9(3): 108-109. 10.1097/xce.0000000000000213
[1854] Trimboli P, Bernasconi E, Buetti N (2020) SARS-CoV-2 viral shedding among diabetic patients: from upper to lower respiratory tract. Endocrine: 1-2. 10.1007/s12020-020-02551-7
[1855] Yang Y, Chen X, Chen YL, Xie HY, Xie CG, Ying LP (2020) The influence of hypoglycemia and hyperglycemia on the adverse outcome of COVID-19 combined with diabetes mellitus A protocol for systematic review and meta-analysis. Medicine 99(44). 10.1097/md.0000000000022587
[1856] Yang Y, Zhong W, Tian Y, Xie CG, Fu XX, Zhou H (2020) The effect of diabetes on mortality of COVID-19 A protocol for systematic review and meta-analysis. Medicine 99(27). 10.1097/md.0000000000020913
[1857] Zhang M, Gao W (2020) COVID-19 and diabetes cutaneous comorbidity. Metabol Open 7: 100055. 10.1016/j.metop.2020.100055
[1858] Ababneh HAFmAMHKaALAMA-RK (2021) Clinical Outcomes of COVID-19 in Hematological Malignancies in Jordan : Tertiary Cancer Center Experience. Blood 138(19): 5039-5039
[1859] Abuyousef SASONE, Elajez REEZIA-AEBRGRRAHFA-AMK, Karawia AAFARKAAAA-AAKMAMA-HM (2021) Early Predictors of Intensive Care Unit Admission among COVID-19 Patients in Qatar. Open Forum Infectious Diseases 8(Suppl 1): S299-S299
[1860] Agodi A, Montineri A, Manuele R, et al. (2021) Risk factors for severe and fatal COVID-19 among patients admitted to an Italian hospital. European Journal of Public Health 31. 10.1093/eurpub/ckab164.098
[1861] Aguilar-Soto MCDMM (2021) Proportion of Patients With HbA1c Corresponding to Prediabetes and Diabetes Ranges Without Previous Diagnosis in a Covid-19 Unit in Mexico City. Journal of the Endocrine Society 5(Supplement_1): A349-A349. 10.1210/jendso/bvab048.711
[1862] Agurto HS, Veramendi-Schultz I, Vasquez-Elera L, et al. (2021) Prevalence and clinical characteristics of gastrointestinal manifestations in covid-19 patients in peru: A multicenter cohort study. Endoscopy 53(SUPPL 1): S267-S267. 10.1055/s-0041-1724999
[1863] Ahmad H, Butt M, Narayan Y, et al. (2021) Comparing the mortality of diabetic and non-diabetic patients with COVID-19 in a uk district general hospital. Revista Argentina de Endocrinología y Metabolismo 58
[1864] Akingboye AA, Mahmood F, Ogunwobi OO, Nightingale P, Amiruddin N, Reay M (2021) Risk of COVID-19 related admissions in cancer patients in a UK metropolitan region. Colorectal Disease 23(SUPPL 1): 113-113. 10.1111/codi.15825
[1865] Al Azzi Y, Pynadath C, Loarte P, et al. (2021) Variation of mortality from sars-cov-2 infection in kidney transplant recipients over the course of the pandemic. American Journal of Transplantation 21(SUPPL 4): 626-626. 10.1111/ajt.16848
[1866] Alabbood MMWAEAZ (2021) A High Incidence of New Onset Diabetes Among Patients with Covid-19 Admitted to Hospital. Endocrine practice : official journal of the American College of Endocrinology and the American Association of Clinical Endocrinologists 27(12): S38-S39
[1867] Alattef D, Al-Hamadani FY, Jamaah AK, et al. (2021) The Experience with Hospitalized COVID-19 Patients in Al-Basra, Iraq: Predictors of the disease severity. Latin American Journal of Pharmacy 40(SI): 291-300
[1868] Alhaddad I, Hammoudeh A, Mdanat E, et al. (2021) COVID-19 pandemic triggers acute myocardial infarction, stroke, and out-of-hospital cardiac arrest in non-infected individuals: The final results of the JoCORE study. European Heart Journal 42(SUPPL 1): 2746-2746. 10.1093/eurheartj/ehab724.2746
[1869] Allo IJT, Tiksnadi BB, Prakoso PP, et al. (2021) Association between hypertension and inhospital mortality in COVID-19 patients. Journal of Hypertension 39(E-SUPPL 2): e18-e19. 10.1097/01.hjh.0000752628.61386.bd
[1870] Almeida C, Sofia Rodrigues Reis M, Alferes D, et al. (2021) Predictors of mortality of covid-19 in chronic hemodialysis patients. Nephrology Dialysis Transplantation 36(SUPPL 1): i461-i462. 10.1093/ndt/gfab098.0016
[1871] Almistehi WMAAMBBA (2021) Incidence of Diabetic Ketoacidosis in Hospitalized COVID19 Patients With Type 2 Diabetes. Journal of the Endocrine Society 5(Supplement_1): A346-A347. 10.1210/jendso/bvab048.706

[1872] Alonso GT, Noor N, Rioles N, Odugbesan O, Ebekozien O, Gallagher MP (2021) Description of presenting symptoms and outcomes among patients with type 1 diabetes and confirmed or suspected COVID-19 in the United States. Pediatr Diabetes 22(SUPPL 29): 83-84. 10.1111/pedi.13198
[1873] Alpizar-Rodriguez D, Irazoque-Palazuelos F, Rodriguez-Reyne TS, et al. (2021) Factors associated with mortality in patients with rheumatic diseases and COVID-19 in Mexico. Annals of the Rheumatic Diseases 80(SUPPL 1): 904-904. 10.1136/annrheumdis-2021-eular.3342
[1874] Amanzai A, Masood J, Pandya N, et al. (2021) ATRIAL FIBRILLATION AND COVID-19 A DEADLY COMBINATION: A RETROSPECTIVE COHORT STUDY IN A LARGE TERTIARY CARE TEACHING HOSPITAL. Chest 160(4): A255-A255. 10.1016/j.chest.2021.07.263
[1875] Amrapala A, Win M, Ainley A (2021) Use of angiotensin-converting-enzyme inhibitors and angiotensin receptor blockers in COVID-19 infection does not adversely affect clinical outcomes including need for non-invasive and invasive ventilation. Thorax 76(Suppl 2): A170-A170. 10.1136/thorax-2021-BTSabstracts.298
[1876] Anwar F, Kuriakose B, Khadija S, et al. (2021) Mechanically ventilated patients with SARS-CoV-2 infection: A singleinstitution analysis. Critical Care Medicine 49(1 SUPPL 1): 95-95. 10.1097/01.ccm.0000726764.79786.c3
[1877] Aouam A, Boujamline S, Marrakchi W, et al. (2021) Profil évolutif du diabète au cours de l’infection à coronavirus. Annales d'Endocrinologie 82(5): 514-514. 10.1016/j.ando.2021.08.767
[1878] Arikan IH, Ozturk S, Tokgoz B, et al. (2021) Acute kidney injury in hospitalized COVID-19 patients: A multicentre study by Turkish society of nephrology. Nephrology Dialysis Transplantation 36(SUPPL 1): i249-i249. 10.1093/ndt/gfab082.0011
[1879] Arjun S, Farraj K, Yeroushalmi K, et al. (2020) EVALUATING OBESITY PARADOX IN COVID 19: INSIGHTS FROM A SAFETY-NET HOSPITAL IN NEW YORK. Chest 158(4): A302-A302. 10.1016/j.chest.2020.08.301
[1880] Ayub A, Ijaz S, Qudrat SM, et al. (2021) The Impact of COVID 19 Pandemic on Type 1 Diabetes Mellitus: An experience of a Tertiary Care Hospital in a resource limited country. Hormone Research in Paediatrics 94(SUPPL 1): 258-258
[1881] Azharul IM, Md Mamunur R, Masuda AF, Masrura H, Md Azizur R (2021) Complete pulmonary function profiles of 267 post COVID patients with exertional dyspnea in Bangladesh. Respirology 26(SUPPL 3): 59-60. 10.1111/resp.14149
[1882] Aziz F, Brauer A, Ciardi C, et al. (2021) Impact of diabetes on Intensive Care Unit admission and in-hospital mortality in patients hospitalized for COVID-19 in Austria. Wiener Klinische Wochenschrift 133(SUPPL 6): 225-225
[1883] Baili H, Bouchareb S, Touil I, et al. (2021) Diabète de primodécouverte chez les patients atteints de covid-19 dans l’unité d’isolement de l’hôpital de Mahdia. Annales d'Endocrinologie 82(5): 525-525. 10.1016/j.ando.2021.08.802
[1884] Baireddy MNSSDKSH (2021) Severe Obesity: A Critical Risk Factor for In-Hospital Complications and Mortality in the Hispanic Population. Open Forum Infectious Diseases 8(Suppl 1): S271-S271
[1885] Baksh ASASSDMCAHAITRC (2021) COVID-19 and older adults with Down syndrome: Presentation, complications and risk for mortality. Alzheimer's & Dementia 17(S10): e056200-e056200. 10.1002/alz.056200
[1886] Balanchivadze NFRSJKP (2020) Hematologic Characteristics of Surviving Versus Non-Surviving COVID-19 Patients in Critical Care Units: Inner-City Hospital Experience, Detroit, MI. Blood 136(Supplement 1): 33-34. 10.1182/BLOOD-2020-135869
[1887] Bansal V, Singh R, Bhurwal A, Rathore S, Kashyap R (2021) Obesity is a risk factor for increased COVID-19 severity: A systemic review and meta-regression. Critical Care Medicine 49(1 SUPPL 1): 43-43. 10.1097/01.ccm.0000726356.00193.e7
[1888] Beltran-Ostos A, Moreno S, Vega-Hoyos D, et al. (2021) Clinical characteristics of severe infections by sars-cov-2 in Colombian patients with covid-19 and rheumatic diseases: A case series study. Journal of Clinical Rheumatology 27(SUPPL 1): S44-S44
[1889] Ben Cheikh A, Ghali H, Bhiri S, et al. (2021) Clinical characteristics, outcomes, and risk factors for mortality in hospitalized patients with COVID-19 from the start of the pandemic in Sahloul university hospital. Antimicrobial Resistance and Infection Control 10(SUPPL 1). 10.1186/s13756-021-00974-z0
[1890] Benyakhlef S, Abdellaoui W, Derkaoui N, Rouf S, Latrech H (2021) Newly-onset type 1 diabetes mellitus triggered by COVID-19: Original case report. Hormone Research in Paediatrics 94(SUPPL 1): 260-260
[1891] Berardo JMDTBFL (2021) The Association between the Baseline Glycemic Control and Antihyperglycemic Therapy with the Severity of SAR-CoV2 Infection in Ethnic Minority Patients with Diabetes. Diabetes 70. 10.2337/db21-177-LB

[1892] Bhagat M, Bevill B (2021) CAVITATION DUE TO COVID-19: A CASE OF SECONDARY ATYPICAL MYCOBACTERIAL INFECTION POST-COVID-19. Chest 160(4): A284-A284. 10.1016/j.chest.2021.07.290
[1893] Birtolo LIIFDACSPSDAFCMCACFGBSMVLCMSRP (2021) ACE-I and ARBS do not influence the chest CT presentation and 1-year survival of COVID-19 patients: Italian multicentre registry. European heart journal supplements : journal of the European Society of Cardiology 23(Suppl G)
[1894] Blair PW, Brandsma JENJ, Richard SA, Striegel DCJMRCEMALCDJSTDBTPSABCD (2021) Phenotypic Differences Between Distinct Immune Biomarker Clusters During the 'Hyperinflammatory' Middle-Phase of COVID-19. Open Forum Infectious Diseases 8(Suppl 1): S320-S321
[1895] Blanco X, Carreras D, Arauco Brown AR (2021) Relationship between history of obesity, hypertension, diabetes and COVID-19 requiring hospitalization with the presence of persistent dyspnea on exertion and abnormal lung imaging in convalescent patients recovering from COVID-19. Am J Respir Crit Care Med 203(9). 10.1164/ajrccm-conference.2021.203.1_MeetingAbstracts.A3093
[1896] Bobadilla RG, Flores C, Francis G, Bengualid V (2020) Association between influenza co-infection and poor outcomes in patients hospitalized with COVID-19. Open Forum Infectious Diseases 7(SUPPL 1): S163-S163. 10.1093/ofid/ofaa417.372
[1897] Bonifacio L, Csizmar V, Pereira AP, et al. (2021) Evaluation of long-term clinical findings among COVID-19 survivors: preliminary results of a prospective cohort study (Recovida). Antimicrobial Resistance and Infection Control 10(SUPPL 1). 10.1186/s13756-021-00974-z0
[1898] Borella N, Wang L, Ross J, Baldridge M, Stone S (2021) COVID-19 is Associated with Increased Mortality and Acute Hyperglycemic Emergencies in Patients with Type 2 Diabetes. Hormone Research in Paediatrics 94(SUPPL 2): 45-46
[1899] Brancale JADSMAJSLK, Hajduk AM, Kang YMATWFP, Jastreboff AM, Lipska KJ (2021) The Impact of Baseline A1C and Admission Glucose on Mortality among Patients with Diabetes Hospitalized with COVID-19. Diabetes 70. 10.2337/db21-1014-P
[1900] Brar G, Pinheiro LC, Shusterman M, et al. (2020) COVID-19 severity and outcomes inhospitalized patients with cancer at a New York Citytertiary medical center: A matched cohort study. Clinical Cancer Research 26(18 SUPPL). 10.1158/1557-3265.COVID-19-S10-01
[1901] Bratsch NA, Campbell N, McAuley J, Close RM (2020) Characteristics and epidemiology of a native american community with a high prevalence of COVID-19. Open Forum Infectious Diseases 7(SUPPL 1): S287-S287. 10.1093/ofid/ofaa439.631
[1902] Busch J, Neto JPR (2021) INCREASED MORTALITY OF COVID-19 IN PATIENTS WITH DIABETES: A RETROSPECTIVE COHORT STUDY IN A HEALTH PLAN IN BRAZIL. Value in Health 24: S86-S86
[1903] Campbell B, Motz K, Pitman M, Fletcher K, Cohen S, Gelbard A (2021) SARS-CoV-2 infection and post-extubation laryngeal function. Otolaryngology - Head and Neck Surgery 165(1 SUPPL): P121-P121. 10.1177/01945998211030908e
[1904] Castañeda-Mendez PF, Castillo-Álvarez JL, Barragán-Reyes A, et al. (2020) Clinical characteristics and mortality of an initial cohort of COVID-19 patients in México City. Open Forum Infectious Diseases 7(SUPPL 1): S287-S287. 10.1093/ofid/ofaa439.632
[1905] Cendrero RMRC, Barquero MVMH, Leo EV, et al. (2021) Impacto de la segunda ola de infección de covid-19 en pacientes de hemodiálisis extremadura (España). Nephrology Dialysis Transplantation 36(SUPPL 1): i481-i481. 10.1093/ndt/gfab098.0061
[1906] Chamorro-Pareja N, Karamanis D, Zavras PD, et al. (2020) Diabetes as a prognostic factor for mortality in coronavirus disease 19 (COVID-19): A systematic review and meta-analysis comprising 18,506 patients. Open Forum Infectious Diseases 7(SUPPL 1): S258-S258. 10.1093/ofid/ofaa417.572
[1907] Chan KH, Patel B, Farouji I, Suleiman A, Slim J (2020) Markers for mortality in COVID-19 patients with atrial fibrillation or flutter. Open Forum Infectious Diseases 7(SUPPL 1): S262-S262. 10.1093/ofid/ofaa417.582
[1908] Chan SB, Smith-Garcia J, Collier C (2020) 40 Racial Disparity and Covid-19 Outcomes: An Emergency Department Study. Annals of Emergency Medicine 76(4): S16-S17. 10.1016/j.annemergmed.2020.09.050
[1909] Charoenngam N, Holick MF, Apovian C (2021) Hyperglycemia at Hospital Presentation Associated with Severity and Mortality in COVID-19 Patients without Type 2 Diabetes. Diabetes 70. 10.2337/db21-181-LB
[1910] Chastain DB, Osae SP, Burt AM, et al. (2020) Baseline characteristics, comorbidities, and outcomes of COVID-19 patients hospitalized in Southwest Georgia, u.s.-an interim analysis of an early hot spot. Open Forum Infectious Diseases 7(SUPPL 1): S251-S251. 10.1093/ofid/ofaa417.559
[1911] Chatzis G, Vlachopoulou M, Gawish AE, et al. (2021) presentation of SARS-CoV-2 (COVID-19 in patients with congestive heart failure. A Retrospective study. Atherosclerosis 331: e289-e289. 10.1016/j.atherosclerosis.2021.06.890

[1912] Chaudhary S, Lo KB, Matta A, Azmaiparashvili Z, Benzaquen S, Patarroyo-Aponte G (2020) VENTILATOR MECHANICS AND OUTCOMES IN CRITICALLY ILL PATIENTS WITH COVID-19 INFECTION. Chest 158(4): A627-A627. 10.1016/j.chest.2020.08.591
[1913] Chaudhuri SR, Majumder A, Sanyal D, Biswas A, Bhattacharjee K (2021) Uncontrolled Hyperglycemia Predicts Ventilatory Support and Duration of Hospital Stay in People with Diabetes and COVID-19. Diabetes 70. 10.2337/db21-627-P
[1914] Chaudhuri SRMASDBABK (2021) Admission Blood Glucose but Not HbA1c Predicts Mortality in People with Diabetes Hospitalized with COVID-19 Infection. Diabetes 70. 10.2337/db21-1098-P
[1915] Cheng D, Desai K, Calderwood C, Otamas A, O'Sullivan N, Ainley A (2020) Late Breaking Abstract - Risk factors of mortality for COVID-19 patients in a North East London cohort: a retrospective analysis. European Respiratory Journal 56. 10.1183/13993003.congress-2020.3594
[1916] Cherrez-Ojeda I, Espinoza Plaza J, Sanchez EJ, Matheus EP, Gochicoa-Rangel LG (2021) Pulmonary evaluation of post-COVID-19 patients: An ecuadorian experience. American Journal of Respiratory and Critical Care Medicine 203(9). 10.1164/ajrccm-conference.2021.203.1_MeetingAbstracts.A3800
[1917] Chiarini G, Boari GEM, Bonetti S, et al. (2021) Role of hypertension, cardiovascular disease and cardiovascular risk factors on outcome in hospitalized COVID-19 patients. Journal of Hypertension 39(SUPPL 1): e205-e205. 10.1097/01.hjh.0000746760.12054.6f
[1918] Chopra A, Arora A, Kukreja I, Gupta A, Verma V, Shukla A (2022) POSA383 Identification of Risk Factors Associated with COVID-19 Hospitalization. Value in Health 25(1): S248-S248. 10.1016/j.jval.2021.11.1209
[1919] Coca A, Burballa C, Pérez FJC, et al. (2021) Male sex is associated with in-hospital death in non-dialysis CKD patients with COVID-19. Nephrology Dialysis Transplantation 36(SUPPL 1): i184-i184. 10.1093/ndt/gfab092.0084
[1920] Collins T, Patel V, Babajanians A, Kubomoto S (2021) Outcomes in inflammatory arthropathy patients hospitalized for COVID-19. Annals of the Rheumatic Diseases 80(SUPPL 1): 228-229. 10.1136/annrheumdis-2021-eular.482
[1921] Corcillo A, Li A, Ayoub K, et al. (2021) Clinical features and sequelae of a cohort of people with type 2 diabetes who required initiation of insulin treatment when hospitalised with severe covid-19. Diabetic Medicine 38
[1922] Corona R, Cunha A (2021) Socioeconomic and clinical factors associated with death and hospitalization of health professionals affected by COVID-19 in Brazil, 2020. Occupational and Environmental Medicine 78(SUPPL 1): A126-A126. 10.1136/OEM-2021-EPI.344
[1923] Danielson L, Allemyar A, Pignanelli M, Monye J, Pinsker R, Patel B (2021) The Effect of COVID-19 on Diabetic Ketoacidosis Management and Outcomes: The Experience of Two NYC Hospitals. American Journal of Respiratory and Critical Care Medicine 203(9)
[1924] Dasa O, Cohen SA, Zheng Y, et al. (2021) Do disparities in cardiovascular comorbidities account for excess covid-19 morbidity and mortality in African Americans? Circulation 143(SUPPL 1). 10.1161/circ.143.suppl_1.042
[1925] Davenport C, Osae S, Thomas G, et al. (2021) Association between Comorbidity Treatment Status and In-Hospital Mortality among COVID-19 Patients in Southwest Georgia, U.S. JACCP Journal of the American College of Clinical Pharmacy 4(12): 1694-1695. 10.1002/jac5.1561
[1926] Davenport COSP, Thomas GYHN, Henao Martínez AF, Franco-Paredes CCDB (2021) Evaluating the Relationship Between Comorbidity Treatment Status and In-hospital Mortality Among COVID-19 Patients. Open Forum Infectious Diseases 8(Suppl 1): S271-S271
[1927] de Lucena DD, de Brito IA, Cristelli MP, Medina-Pestana JO, Tedesco-Silva H, Rangel EB (2021) Relationship Between Diabetes Mellitus and Covid-19 Prognosis in Kidney Transplant Patients. American Journal of Transplantation 21: 629-629
[1928] El Halabi M, Feghali J, Bahk J, et al. (2021) A NOVEL EVIDENCE-BASED PREDICTOR TOOL FOR HOSPITALIZATION AND LENGTH OF STAY: INSIGHTS FROM PATIENTS WITH COVID-19 IN NEW YORK CITY. Chest 160(4): A553-A554. 10.1016/j.chest.2021.07.536
[1929] El Horri M, Touati SNE, Khachaa I, et al. (2021) Fibrinogen and D-dimer are significantly associated with severity of COVID-19 disease. Research and Practice in Thrombosis and Haemostasis 5(SUPPL 2). 10.1002/rth2.12589
[1930] Elabidin AZ, Pereira L, Namdarizandi V, et al. (2021) Elevated BNP Is Associated with Higher Risk of Cardiac Arrest in Diabetic Patients Hospitalized with COVID-19. Diabetes 70. 10.2337/db21-361-P
[1931] Esteban-Lucia L, Devesa-Arbiol A, Zambrano Chacon MA, et al. (2021) Cardiovascular predictors associated with poor short-term prognosis in patients diagnosed with SARS-CoV-2 in Spain. European Heart Journal 42(SUPPL 1): 2468-2468. 10.1093/eurheartj/ehab724.2468
[1932] Esteban-Lucia L, Zambrano Chacon MA, Venegas Rodriguez AM, et al. (2021) Calcium channel blockers and beta-blockers could increased mortality of patients with COVID-19. European Heart Journal 42(SUPPL 1): 3006-3006. 10.1093/eurheartj/ehab724.3006

[1933] Esteban-Lucia L, Zambrano Chacon MA, Venegas Rodriguez AM, et al. (2021) Effect of angiotensin-converting enzyme inhibitors and angiotensin receptor blockers on the short-term prognosis of COVID-19 infection. European Heart Journal 42(SUPPL 1): 2969-2969. 10.1093/eurheartj/ehab724.2969
[1934] Fanning JP, Barnett AWGARLPBDHSDBPCHSJGLBFJGMRCS-MC (2021) Case-control study of risk factors for stroke among critically-ill patients with SARS-CoV-2: an analysis of the COVID-19 critical care consortium (CCCC) global registry. BMJ Neurology Open 3(Suppl 1): A13-A14. 10.1136/bmjno-2021-ANZAN.35
[1935] Fatade YA, Collins LF, Collins LF, Almuwaqqat ZCZPMQAOI (2021) The Effects of Race and Comorbidity Burden on Inflammatory Biomarkers Among Persons Hospitalized with COVID-19. Open Forum Infectious Diseases 8(Suppl 1): S322-S323
[1936] Fatmi RE, Saidi EI, Kanoun RY, et al. (2021) Clinical charcteristics and risk factors of severity of covid19 infection in patients with hematological malignancies. HemaSphere 5(SUPPL 2): 752-752. 10.1097/HS9.0000000000000566
[1937] Ferreira DB, Marques FM, Almeida SB, et al. (2020) CARACTERÍSTICAS CLÍNICAS E DESFECHOS DE PACIENTES COM DOENÇAS HEMATOLÓGICAS E DIAGNÓSTICO DE SARS-COV-2 EM HOSPITAL PÚBLICO TERCIÁRIO. Hematology, Transfusion and Cell Therapy 42: 520-520. 10.1016/J.HTCT.2020.10.878
[1938] Fisher M, Yunes M, Mokrzycki MH, Golestaneh L, Coco M (2020) ESKD patients hospitalized with COVID-19: Early outcomes in Bronx, New York. Journal of the American Society of Nephrology 31: 262-262
[1939] Fleischer JG, Pallay RA, O'Shea CF, Corwin DS (2021) Analysis of disease severity and sex specific data among hospitalized covid19 patients. American Journal of Respiratory and Critical Care Medicine 203(9). 10.1164/ajrccm-conference.2021.203.1_MeetingAbstracts.A1509
[1940] Formoso M, Falconieri M, Tagliente S, et al. (2020) Gender differences in patients with SARS-CoV2:a retrospective observational cohort study. Italian Journal of Medicine 14(SUPPL 2): 121-121
[1941] Fumagalli S, Pelagalli G, Trevisan C, et al. (2021) Atrial fibrillation and COVID-19 in older patients: A complex, dangerous, association. An analysis of the GeroCovid Registry. Europace 23(SUPPL 3): iii169-iii169. 10.1093/europace/euab116.154
[1942] Gallagher MP, Noor N, Odugbesan O, Rioles N, Ebekozien O, Alonso GT (2021) Race/Ethnicity Disparities of presenting symptoms and outcomes among patients with type 1 diabetes with confirmed or suspected COVID-19 in the United States. Pediatr Diabetes 22(SUPPL 29): 84-None. 10.1111/pedi.13198
[1943] Gallardo-Rincón HMAM-JLAL-GJS-MER, Mujica-Rosales RT-CR (2021) Diabetes and Comorbidities Risk Assessment in Hospitalization and Fatalities from the Mexican COVID-19 Surveillance System. Diabetes 70. 10.2337/db21-1068-P
[1944] Galloway JSRCFRJBARCSLNS (2020) 185 A clinical risk score to identify patients with COVID-19 at high risk of critical care admission or death: An observational cohort study. Emergency Medicine Journal : EMJ 37(12): 843-844. 10.1136/emj-2020-rcemabstracts.38
[1945] García GF, Saponara LGP, Escudero PS, et al. (2021) Influence of sars-cov-2 infection on mortality in patients under renal substitute treatment. Nephrology Dialysis Transplantation 36(SUPPL 1): i482-i482. 10.1093/ndt/gfab098.0064
[1946] Garcia Moreno FJ, Chamorro De Vega E, Rodriguez Gonzalez C, et al. (2021) Analysis of factors related to the clinical course of covid-19 infection in patients with hypertension. European Journal of Hospital Pharmacy 28(SUPPL 1): A123-A123. 10.1136/ejhpharm-2021-eahpconf.253
[1947] Garg SGSTNDS (2021) Comparison of Clinical Characteristics and Outcomes Among the Patients with and Without Type 2 Diabetes, Infected with Sars-Cov-2, Hospitalized During the Second Wave of COVID-19 Pandemic in India. Endocrine practice : official journal of the American College of Endocrinology and the American Association of Clinical Endocrinologists 27(12): S44-S44
[1948] Garibay-Vega BR, Castañeda-Mendez PF, Castillo-Álvarez JL, et al. (2020) Risk factors associated with requiring invasive mechanical ventilation in patients with SARS-COV-2 infection: Experience in a tertiary care hospital in Mexico City. Open Forum Infectious Diseases 7(SUPPL 1): S268-S268. 10.1093/ofid/ofaa417.593
[1949] Gerardine S, Jaimes EA, Derkach A, et al. (2021) AKI in COVID-19 patients and history of cancer: Role of d-dimer as a potential risk factor. Journal of the American Society of Nephrology 32: 113-113
[1950] Ghoneim S, Butt MU, Saleh S, Fass R (2021) THE RISK OF COVID-19 IS HIGHER IN FEMALES USING PROTON PUMP INHIBITORS. Gastroenterology 160(6): S-436. 10.1016/S0016-5085(21)01756-X
[1951] Glassberg B, Trivedi A, Truglio J (2021) SEVERE NEW-ONSET TYPE 2 DIABETES MELLITUS IN YOUNG-ADULT PATIENT POST-COVID-19 INFECTION. Journal of General Internal Medicine 36(SUPPL 1): S266-S266

[1952] Goel NN, Soh H, Villars M, et al. (2021) Predictors of pre-icu duration and its association with mortality in COVID-19 infection: A secondary analysis of the stop-covid registry. American Journal of Respiratory and Critical Care Medicine 203(9). 10.1164/ajrccm-conference.2021.203.1_MeetingAbstracts.A1493
[1953] Gokce A, Ozer A, Soyiler V, Gundogdu ME (2021) Investigation of Epidemiological Characteristics of Patients over Age 65 with Covid-19 ın a City. European Journal of Public Health 31. 10.1093/eurpub/ckab165.182
[1954] Goli K, Galvan NT, Cotton R, Goss JA, O'Mahony CA, Rana A (2021) COVID-19 related deaths across the u.s. in kidney transplant recipients. American Journal of Transplantation 21(SUPPL 4): 353-353. 10.1111/ajt.16847
[1955] Golpayegany SOSP, Thomas GYHN, Martínez AFHF-PCCDB (2021) Factors of Social Determinants of Health Associated with Length of Stay in COVID-19 Patients with Multimorbidity in Southwest Georgia, United States. Open Forum Infectious Diseases 8(Suppl 1): S333-S334
[1956] Graczyk S, Khan N, Cosimi R (2021) Effect of hospitalized COVID-19 patient demographics and treatments on length of stay and morbidity. JACCP Journal of the American College of Clinical Pharmacy 4(9): 1226-1226. 10.1002/jac5.1481
[1957] Graham JK, Failla K (2021) COVID positive patients at a community healthcare system in Southern California. Stratification of severity of illness and survivability based on individual patient characteristics and underlying conditions. American Journal of Respiratory and Critical Care Medicine 203(9). 10.1164/ajrccm-conference.2021.203.1_MeetingAbstracts.A2526
[1958] Grewal SD, Pour AA, Heidari-Bateni G, et al. (2021) Risk factors for in-hospital mortality among patients hospitalized with COVID-19 and AKI. Journal of the American Society of Nephrology 32: 73-73
[1959] Guo L, Peng X, Jiang C, et al. (2020) CLINICAL CHARACTERISTICS OF 537 CASES OF COVID-19 IN DIFFERENT ALTITUDE OF SICHUAN PROVINCE, CHINA: A RETROSPECTIVE STUDY. Chest 158(4): A2497-A2497. 10.1016/j.chest.2020.09.069
[1960] Gupta KMLKMAGJ, Brar I (2021) Risk Factors Associated with 30-Day Mortality in a Large Cohort of Patients who Received Remdesivir and Corticosteroids for Severe COVID-19. Open Forum Infectious Diseases 8(Suppl 1): S375-S376
[1961] Gupta N, Shah J, Sarmiento J, Ilyas N, Ornstein V (2021) Characteristics of COVID-19 readmissions in an urban community hospital. American Journal of Respiratory and Critical Care Medicine 203(9). 10.1164/ajrccm-conference.2021.203.1_MeetingAbstracts.A3785
[1962] Haarhaus M, Santos C, Haase M, Veiga PM, Lucas C, Macário F (2021) Risk prediction of covid-19 incidence and mortality in a large multi-national haemodialysis cohort: Implications for management in outpatient settings. Nephrology Dialysis Transplantation 36(SUPPL 1): i456-i457. 10.1093/ndt/gfab098.006
[1963] Hachem RY, Chaftari A-MMNHNDHJYSBBASRCAFGEBAMNDTSSD, Mulanovich VE, Adachi JBJKFGBJTGEYTHA, Slavin MTBSVKDP, Malek ARII (2021) International Multicenter Study Comparing Cancer to Non-Cancer Patients with COVID-19: Impact of Risk Factors and Treatment Modalities on Outcome. Open Forum Infectious Diseases 8(Suppl 1): S15-S15
[1964] Hafiz M, Nalamalapu S, Kragel E, et al. (2021) Outcomes of COVID-19 patients with underlying asthma and COPD: Analysis from a rural setting in the unites states. American Journal of Respiratory and Critical Care Medicine 203(9). 10.1164/ajrccm-conference.2021.203.1_MeetingAbstracts.A2513
[1965] Hasibul Hasan ATM (2021) In hospital mortality and short-term outcome of acute ischemic stroke patients contracting SARS CoV-2 infection: Experience from a dedicated stroke unit in Bangladesh. Journal of the Neurological Sciences 429. 10.1016/j.jns.2021.119700
[1966] Hawa F, Kassab IA, Berinstein JA, et al. (2020) Hepatic steatosis is associated with increased disease severity in patients with novel coronavirus disease 2019 (covid-19). Hepatology 72(1 SUPPL): 276A-276A. 10.1002/hep.31579
[1967] Hayat A, Hogsand T, Shafqat SA, Mohandas C (2021) Incidence of deterioration, variance and recovery of e-glomerular filtration rate renal function in patients with diabetes hospitalised with covid-19. Diabetic Medicine 38
[1968] Hehar JTELSW (2021) The Relationship Between Age, Gender, Race, Diabetes and Obesity on Clinical Outcomes in a Large Cohort of Patients Hospitalized for Covid-19 in Metropolitan Detroit. Journal of the Endocrine Society 5(Supplement_1): A351-A351. 10.1210/jendso/bvab048.715
[1969] Henderson J, Britton Z, Hall C, et al. (2021) COVID-19 infection and stroke in hospitalised patients: A retrospective analysis of incidence, stroke character, risk factors and mortality. European Stroke Journal 6(1 SUPPL): 290-291. 10.1177/23969873211034932
[1970] Hendra H, Sudusinghe D, Greenan-Barrett J, et al. (2021) Impact of early fluid administration on inpatient mortality and acute kidney injury in patients with COVID-19 disease. Nephrology Dialysis Transplantation 36(SUPPL 1): i245-i246. 10.1093/ndt/gfab082.003

[1971] Herc E, Yared NF, Kudirka A, Suleyman G (2020) Characteristics and outcomes of COVID-19 patients with fungal infections. Open Forum Infectious Diseases 7(SUPPL 1): S253-S253. 10.1093/ofid/ofaa417.561
[1972] Hernandez-Acosta RA, Sarmiento JPPHMRK (2021) Clinical Variables Associated with COVID-19 Mortality and ICU Admission in a Public Safety-net Hospital in Chicago. Open Forum Infectious Diseases 8(Suppl 1): S250-S251
[1973] Hicks LK, Redd RA, Anderson KC, et al. (2021) Risks for Hospitalization and Death Among Patients with Blood Disorders from the ASH RC COVID-19 Registry for Hematology. Blood 138(19): 3040-3040
[1974] Ho GDJR, Schmittdiel JKJTJPA (2020) Anticoagulant and Antiplatelet Use Not Associated with Improvement in Severe Outcomes in COVID-19 Patients. Blood 136(Supplement 1): 59-59. 10.1182/BLOOD-2020-134254
[1975] Hofler S, Bozkurt L, Eichner M, et al. (2021) Evaluation of glycemic Control in hospitalized Patients with Diabetes Mellitus as a Part of Covid-associated Pneumonia. Wiener Klinische Wochenschrift 133(SUPPL 6): 211-211
[1976] Horii TAWOKAK (2021) Risk Factor for COVID-19 Infection-Related Hospitalization in Patients with Type 2 Diabetes. Diabetes 70. 10.2337/db21-1007-P
[1977] Huber S, Montalvão S, Martins S, et al. (2021) Hemostastatic alterations with disease severity in the early symptomatic phase of COVID-19. Research and Practice in Thrombosis and Haemostasis 5(SUPPL 2). 10.1002/rth2.12589
[1978] Hwang AW, Zackria R, Choi EH, Heineke HS, Ayutyanont N, Kubomoto S (2021) CIRRHOSIS IS ASSOCIATED WITH SIGNIFICANTLY WORSENED CLINICAL OUTCOMES OF COVID-19 INFECTION IN A RETROSPECTIVE ANALYSIS OF 23,474 PATIENTS ACROSS 143 HOSPITALS. Gastroenterology 160(6): S-756. 10.1016/S0016-5085(21)02517-8
[1979] Iftikhar H, Alaee S, Bennett J, et al. (2021) Gloucestershire nhs foundation trust Experience-covid-19 associated mortality in Mechanical ventilation vs non mechanical Ventilation. Thorax 76(SUPPL 1): A116-A117. 10.1136/thorax-2020-BTSabstracts.200
[1980] Igwe CI, Pang YP, Briggs J, Higgins K (2021) Glycaemic control in covid-19 patients in an intensive care unit (ICU) setting. Diabetic Medicine 38(SUPPL 1): 47-47. 10.1111/dme.14556
[1981] Ip AMAIJHNP, Petrillo AVRSRCCMGLLA, Goy AH, Feldman TA (2020) COVID-19 Impact on Lymphoma Patients' Clinical Outcomes - an Observational Cohort Study. Blood 136(Supplement 1): 6-7. 10.1182/BLOOD-2020-141871
[1982] Issac A, Kochuparambil J (2022) POSC1 The Influence of Diabetic History in the Prognosis of Hospitalized COVID-19 Patients: A Prospective Observational Study. Value in Health 25(1): S32-S32. 10.1016/j.jval.2021.11.147
[1983] Italiya B, Vithalani K (2022) POST-COVID-19 PULMONARY ASPERGILLOSIS AND MUCORMYCOSIS CO-INFECTION IN A PATIENT WITH DIABETES: A COMBINED CHALLENGING COMPLICATION. Chest 161(1): A154-A154. 10.1016/j.chest.2021.12.186
[1984] Jain A, Nayak L, Kulkarni UP, et al. (2021) Covid-19 Infection in Hematological Malignancies: Registry Data from India. Blood 138: 1938-1938. 10.1182/blood-2021-149100
[1985] Jakoby MG, Williams VWSLSK, Buhnerkempe MIK (2021) Glycemic Control Is Not an Independent Predictor of Mortality or Length of Stay for Patients Hospitalized with Coronavirus 2019 (COVID-19). Diabetes 70. 10.2337/db21-145-LB
[1986] Jalilova A, Ata A, Demir G, et al. (2021) Does SARS-COV-2 outbreak increase diabetic ketoacidosis in new onset T1DM. Hormone Research in Paediatrics 94(SUPPL 1): 244-244
[1987] Jana K, Janga KC, Greenberg S (2021) AKI in COVID-19: Risk factors for mortality and estimating burden using an ARIMA model. Journal of the American Society of Nephrology 32: 60-60
[1988] Jean-Louis G, Seixas A, Cejudo JR, et al. (2021) Contribution of pulmonary diseases to COVID-19 mortality in a diverse community of New York City. Sleep 44(SUPPL 2): A267-A267. 10.1093/sleep/zsab072.682
[1989] Jevalikar GSRFKJ, Singh ABSDAMA (2021) Lack of Association Between 25-Hydroxyvitamin D Level and Outcomes in Hospitalized Indian Patients With COVID-19. Journal of the Endocrine Society 5(Supplement_1): A277-A278. 10.1210/jendso/bvab048.563
[1990] Kalyoncuoğlu M (2020) Mortality Predictors for severe coronavirus disease 2019 patients who suffered miyocardial injury: A single center experience. Anatolian Journal of Cardiology 24(SUPPL 1): 111-112
[1991] Kamalumpundi VSECLTAS-RAA, Dokun AO, Correia M (2021) Patients With Obesity-Related Hypertension Treated With Renin-Angiotensin-Aldosterone System Antagonists Exhibit Lower Mortality And Less Severe COVID-19: A Retrospective Study. Journal of the Endocrine Society 5(Supplement_1): A296-A296. 10.1210/jendso/bvab048.601
[1992] Kaneko SKHMKTSYUEHTI (2021) Diabetes in Japanese COVID-19 Patients as the Primary Factor of Accelerated Progression to Severe State. Diabetes 70. 10.2337/db21-1090-P

[1993] Kang YMBJADAJSLLKJJAM (2021) Obesity Is Associated With Intensive Care Use and Duration of ICU Stay but Not Mortality Among 3246 Patients Hospitalized With COVID-19. Journal of the Endocrine Society 5(Supplement_1): A61-A62. 10.1210/jendso/bvab048.125
[1994] Kangas-Dick A, Gazivoda V, Ibrahim M, Wiesel O (2020) CLINICAL CHARACTERISTICS AND OUTCOME OF PNEUMOTHORAX IN PATIENTS WITH COVID-19 PNEUMONIA. Chest 158(4): A2648-A2648. 10.1016/j.chest.2020.09.232
[1995] Karmali D, Miller H, Desai V, Weaver M, Frieboes H, Suliman SA (2021) A comparison of risk factors that predict mortality in COVID-19 patients across two surge periods. American Journal of Respiratory and Critical Care Medicine 203(9). 10.1164/ajrccm-conference.2021.203.1_MeetingAbstracts.A3784
[1996] Karpel H, Kreiger-Benson E, Martell S, Luoma K, Mukherjee V (2020) SEX-SPECIFIC OCCUPATIONAL AND CLINICAL CHARACTERISTICS OF CRITICALLY ILL PATIENTS WITH COVID-19 IN NEW YORK CITY. Chest 158(4): A349-A349. 10.1016/j.chest.2020.08.346
[1997] Khan H, Hamadani A, Amir S (2021) Demographics and clinical presentation of Covid-19 patients admitted to a public tertiary care hospital, Lahore. International Journal of Rheumatic Diseases 24(SUPPL 2): 159-160. 10.1111/1756-185X.14200
[1998] Kharbanda S, Vanderpant N, Muralidhara K (2021) A further look into COVID-19 related mortality in patients with diabetes at a District General Hospital. British J Diabetes 21(1): 6-None. 10.15277/bjd.2021.300
[1999] Khasag A, Erdenechuluun B, Byambasukh O (2021) Diabetes and COVID-19 in mongolian patients. Journal of Diabetes Investigation 12(SUPPL 1): 15-15. 10.1111/jdi.13662
[2000] Khruleva Y, Mubayazvamba T, Troitskaya E, Efremovtseva M, Kobalava Z (2021) Hypertension is a risk factor for acute kidney injury and a predictor of mortality in hospitalized patients with COVID-19. Journal of Hypertension 39(SUPPL 1): e406-e406. 10.1097/01.hjh.0000749296.26490.d9
[2001] Krepostman N, Collins M, Merchant K, et al. (2021) Predictors of clinical decompensation in patients presenting with COVID-19 in an urban hospital health system. European Heart Journal 42(SUPPL 1): 2473-2473. 10.1093/eurheartj/ehab724.2473
[2002] Krishnan A, Prichett L, Tao X, et al. (2020) Clinical characteristics and outcomes of COVID-19 patients with and without pre-existing chronic liver disease. Hepatology 72(1 SUPPL): 262A-262A. 10.1002/hep.31579
[2003] Kukreja I, Chopra A, Arora A, Verma V, Shukla A (2022) POSB396 Identification of Risk Factors Associated with ICU Admission in COVID-19 Patients. Value in Health 25(1): S258-S258. 10.1016/j.jval.2021.11.1259
[2004] Kulkarni R, Maharaul M, Kurpad K, et al. (2021) OUTCOMES OF NOVEL CORONAVIRUS DISEASE IN PATIENTS WITH DIABETES MELLITUS: A RETROSPECTIVE ANALYSIS. Critical Care Medicine 49(1): 47-47
[2005] Kumar GJDPDMMNR (2022) IS UNCONTROLLED DIABETES PRIOR TO HOSPITALIZATION A RISK FACTOR FOR WORSE OUTCOMES IN COVID-19? Critical Care Medicine 50: 47-47. 10.1097/01.ccm.0000806836.99336.73
[2006] Kumar S, Vavilin I, Montero A (2021) INFLAMMATORY MARKERS AS PREDICTORS OF INTUBATION IN PATIENTS WITH COVID-19. Chest 160(4): A539-A539. 10.1016/j.chest.2021.07.526
[2007] Lampsas S, Oikonomou E, Siasos G, et al. (2021) Mid-term endothelial dysfunction post COVID-19. European Heart Journal 42(SUPPL 1): 3401-3401. 10.1093/eurheartj/ehab724.3401
[2008] Lebastchi JMFMME, Gopalkrishnan G (2021) Abstract #1042611: Impact of Diabetes and Glycemic Control on Poor Outcomes in Hospitalized Patients with Obesity and COVID-19 Infectionq2. Endocrine Practice 27(6, Supplement): S84-S84. 10.1016/j.eprac.2021.04.645
[2009] Lee S, Balobaid A, Lastinger A, Reece R (2021) Follow-up of hospitalized adults with post-acute COVID-19 syndrome. American Journal of Respiratory and Critical Care Medicine 203(9). 10.1164/ajrccm-conference.2021.203.1_MeetingAbstracts.A2651
[2010] Li Bassi G, Suen J, Dalton HJ, et al. (2021) Factors associated with mortality in patients with COVID-19 requiring mechanical ventilation: An international cohort study from 139 intensive care unit across 6 continents. American Journal of Respiratory and Critical Care Medicine 203(9). 10.1164/ajrccm-conference.2021.203.1_MeetingAbstracts.A1059
[2011] Lobanova KG, Demidova TYU, Plakhotnyaya VM (2021) Impact of type 2 diabetes mellitus on clinical outcomes in patients with COVID-19 and cardiovascular disease. European Heart Journal 42: 2625-2625
[2012] Low M, Kwok A, Lui A, et al. (2021) Clinical outcomes associated with early in-hospital use of metformin, dipeptidyl peptidase-4 inhibitor or insulin in COVID-19 patients with type 2 diabetes: A territory-wide retrospective observational study. Journal of Diabetes Investigation 12(SUPPL 1): 15-15. 10.1111/jdi.13662
[2013] Lui A, Kwok A, Low M, et al. (2021) Preadmission use of DPP4i reduced risk of hyperinflammatory syndrome, in-hospital death or invasive mechanical ventilation in COVID-19

patients with type 2 diabetes: A territory-wide retrospective cohort study. Journal of Diabetes Investigation 12(SUPPL 1): 22-23. 10.1111/jdi.13663
[2014] Lukitsch I, Ortiz AET, Mohammed AE, Walker JB, Mohamed M, Velez JCQ (2020) Morbid obesity, hypertension, and male sex are associated with greater risk for AKI in patients with COVID-19. Journal of the American Society of Nephrology 31: 259-259
[2015] Macauley P, Yaqoob H, Manglani R, GreenbergMd D, Chandy D (2021) Obesity and severe COVID-19: A retrospective analysis of intensive care unit patients at a quaternary medical center. American Journal of Respiratory and Critical Care Medicine 203(9). 10.1164/ajrccm-conference.2021.203.1_MeetingAbstracts.A2510
[2016] Mansour W, Knauert M (2021) Sleep apnea severity and COVID-19 hospitalization outcomes: Interim analysis. Sleep 44(SUPPL 2): A266-A266. 10.1093/sleep/zsab072.679
[2017] Marta GDH, Auclin E, Saoudi Gonzalez N, et al. (2020) Active smoking and severity of COVID-19 infection in cancer patients. Annals of Oncology 31: S1007-S1008. 10.1016/j.annonc.2020.08.1777
[2018] Mathew J, Schechter C, Southern W, Crandall JP, Tomer Y, Agarwal S (2021) Interaction of COVID-19 and preexisting diabetes on hospital presentation of diabetic ketoacidosis. Diabetes 70(SUPPL 1). 10.2337/db21-1023-P
[2019] Mazur K, Kania M, Terlecki M, et al. (2021) Characteristics and predictors of mortality in diabetes patients hospitalised with COVID-19: a single-centre cohort study from Poland. Diabetologia 64(SUPPL 1): 360-360
[2020] McDonnell ME, Simonson DC, Cromwell GGGGMJMKSR, et al. (2021) Stress Hyperglycemia Predicts Mechanical Ventilation and Death in COVID-19 Infected Adults. Diabetes 70. 10.2337/db21-175-LB
[2021] Mechineni A, Samuel A, Manickam R (2021) Diagnostic value of d dimer serum marker in COVID 19 patients. American Journal of Respiratory and Critical Care Medicine 203(9). 10.1164/ajrccm-conference.2021.203.1_MeetingAbstracts.A3831
[2022] Miklin D, Mantha A, Wolfson A, Vaidya A, Depasquale E (2021) Outcomes of COVID-19 infection in heart transplant recipients. European Heart Journal 42(SUPPL 1): 964-964. 10.1093/eurheartj/ehab724.0964
[2023] Mileham KF, Bruinooge SS, Aggarwal C, et al. (2021) Mortality risk for patients undergoing cancer treatment who acquire SARS-CoV-2: ASCO registry. Journal of Clinical Oncology 39(15 SUPPL). 10.1200/JCO.2021.39.suppl.6509
[2024] Millet C, Narvaneni S, Chaudhry S, et al. (2021) THE LONG HAUL: A FOLLOW UP STUDY OF PATIENTS DIAGNOSED WITH COVID-19 ONE YEAR AGO AT AN URBAN MEDICAL CENTER IN NEW JERSEY. Chest 160(4): A566-A567. 10.1016/j.chest.2021.07.547
[2025] Milloh Raz H, Ben Hamo M, Minsky NC, Segal-Lieberman G (2021) Association between BMI and the risk of positive SARS-CoV-2 testing. Obesity Facts 14(SUPPL 1): 175-175. 10.1159/000516203
[2026] Milosavljevic JLJTA (2021) Factors Associated With High Glycemic Variability in Patients With Diabetes Hospitalized With COVID-19. Endocrine practice : official journal of the American College of Endocrinology and the American Association of Clinical Endocrinologists 27(12): S11-S11
[2027] Misotti AM, Cremonesi I, Mascheroni A, Merelli E, Tamburelli B, Tirelli ME (2021) CLINICAL AND NUTRITIONAL CHARACTERISTICS OF NON-ICU PATIENTS WITH SARS-COV-2 INFECTION. Nutrition 87-88: 111326-111326. 10.1016/j.nut.2021.111326
[2028] Mithal AJGSRSAFKJ, Dewan ABS (2021) High Prevalence of Diabetes in Hospitalized Patients With COVID-19 and Its Association With Greater Severity of COVID-19 in Delhi, India. Journal of the Endocrine Society 5(Supplement_1): A342-A342. 10.1210/jendso/bvab048.697
[2029] Mlouki I, Khelij H, Jaoued O, et al. (2021) Hospital-acquired infections associated to COVID-19 intensive care unit at a Tunisian hospital: Determinants factors. Antimicrobial Resistance and Infection Control 10(SUPPL 1). 10.1186/s13756-021-00974-z0
[2030] Mohammed AGA-KM (2021) Covid-19 Could Trigger Diabetes in Healthy Individuals and Worsen Symptoms in Pre-Diagnosed Diabetes Mellitus Patients. Endocrine practice : official journal of the American College of Endocrinology and the American Association of Clinical Endocrinologists 27(12): S26-S27
[2031] Mokoagow MIHDS, Kshanti IARCM (2021) Abstract #1022296: Association between Laboratory Parameters on Admission and Poor Clinical Outcome of COVID-19 in Patients with Type 2 Diabetes Mellitus: A Cross-Sectional Study. Endocrine Practice 27(6, Supplement): S65-S66. 10.1016/j.eprac.2021.04.607
[2032] Moran CA, Oliver N, Szabo BV, et al. (2021) Comorbidity burden is associated with hospitalization for COVID-19 among PWH. Topics in Antiviral Medicine 29(1): 207-207
[2033] Moreno FJGCDVE, Gonzalez CROAMVJVFBSSITBCVDLPA, Alonso AHSMS (2021) 5PSQ-134 Analysis of factors related to the clinical course of COVID-19 infection in patients with

hypertension. European Journal of Hospital Pharmacy Science and Practice 28(Suppl 1): A123-A123. 10.1136/ejhpharm-2021-eahpconf.253
[2034] Morosini UM, Rosso G, Merlotti G, et al. (2021) Increased prevalence of acute kidney injury and mortality in COVID-19 hospitalized patients. Nephrology Dialysis Transplantation 36(SUPPL 1): i258-i259. 10.1093/ndt/gfab082.0034
[2035] Morozova D, Nezhdanov K, Eliseeva O, Minushkina L, Zateyshchikov D (2021) Atherothrombotic events in hospitalized COVID-19 patients are associated with diabetes mellitus. Research and Practice in Thrombosis and Haemostasis 5(SUPPL 2). 10.1002/rth2.12589
[2036] Mossayebi A, Kreit H, Cherukuri SV, et al. (2021) Unmanaged Diabetes as a Poor Prognostic Factor in the Severity of Infection and Recovery Time of Hospitalized COVID-19 Patients. Diabetes 70. 10.2337/4821-845-p
[2037] Mrozikiewicz-Rakowska B, Balut D, Bak PM, Kleibert M, Zielinski J (2021) The Phenomenon of Metformin in COVID-19 Pandemic Among Polish Patients with Type 2 Diabetes Mellitus. American Heart Journal 242: 173-173. 10.1016/j.ahj.2021.10.068
[2038] Muzaffarr Z, Ambut J, Gottlieb S (2021) Obesity and outcomes in patients with COVID-19: A retrospective review from a community hospital. American Journal of Respiratory and Critical Care Medicine 203(9). 10.1164/ajrccm-conference.2021.203.1_MeetingAbstracts.A3838
[2039] Myers CN, Kim J, Stanley P, et al. (2021) Factors associated with hospitalization length of stay in patients with COVID-19, a multivariable analysis. American Journal of Respiratory and Critical Care Medicine 203(9). 10.1164/ajrccm-conference.2021.203.1_MeetingAbstracts.A2639
[2040] Nada KM, Adame M, Baig M, et al. (2021) Long-term health effects of COVID-19 in hospitalized and non-hospitalized patients. American Journal of Respiratory and Critical Care Medicine 203(9). 10.1164/ajrccm-conference.2021.203.1_MeetingAbstracts.A3791
[2041] Natali KM, Chan KH, Atallah L, Nagarakanti S, Slim J (2020) Could anticoagulant use prior to infection with COVID-19 decrease mortality? Open Forum Infectious Diseases 7(SUPPL 1): S342-S342. 10.1093/ofid/ofaa439.746
[2042] Nica AE, Rusu E, Dobjanschi CG, Radulian G (2021) The impact of obesity in patients with SARS-CoV-2 infection. Obesity Facts 14(SUPPL 1): 193-193. 10.1159/000516203
[2043] Niu JSCDMSAE (2021) Prognostic Value of Absolute Lymphocyte Count for Disease Severity and Clinical Outcomes in Adult COVID-19 Inpatients. Open Forum Infectious Diseases 8(Suppl 1): S324-S324
[2044] Omri N, Ben youssef F, Jaoued O, et al. (2021) Associated factors with extended hospital stay among COVID-19 patients in intensive care unit at an university hospital in Tunisia. Antimicrobial Resistance and Infection Control 10(SUPPL 1). 10.1186/s13756-021-00974-z0
[2045] Ostrosky-Frid M, McAdams MC, Xu P, Li MM, Hedayati S (2021) AKI in inpatients with COVID-19: Risk factors and mortality. Journal of the American Society of Nephrology 32: 61-61
[2046] Pan A, Meeks J, Potter T, et al. (2021) COVID-19 susceptibility and mortality among individuals with mild cognitive impairment, alzheimer's disease and related dementias. Stroke 52(SUPPL 1). 10.1161/str.52.suppl-1.MP18
[2047] Pana C, Iordache A, Bogdan C, Stanigut AMS, Tuta LA (2021) Evolution and prognosis of hemodyalisis patients with moderate and severe covid infection: Single hospital centre experience. Nephrology Dialysis Transplantation 36(SUPPL 1): i492-i492. 10.1093/ndt/gfab100.0020
[2048] Pandita A, Gillani FS, Shi Y, et al. (2020) Factors associated with severe COVID-19 among patients hospitalized in Rhode Island. Open Forum Infectious Diseases 7(SUPPL 1): S324-S325. 10.1093/ofid/ofaa439.712
[2049] Patange S, Padhye D, Gandhi A, et al. (2021) Diabesity As The Risk Factor For The Need of Intensive Care Unit for the Management of COVID-19. Obesity Facts 14(SUPPL 1): 193-193. 10.1159/000516203
[2050] Peinado P, Sanz Garcia E, Moreno I, et al. (2020) COVID-19 severe pneumonia in cancer patients: Impact and predictive factors. Annals of Oncology 31: S1024-S1024. 10.1016/j.annonc.2020.08.1824
[2051] Pereira LNVKSAFMTBKBBDM, Shemisa KEAZSV (2021) Metabolic Syndrome Is Associated with a Longer Complicated ICU Stay and Greater Mortality in Patients Hospitalized with COVID-19. Diabetes 70. 10.2337/db21-296-OR
[2052] Peride I, Niculae A, Balas CA, Nechita AM, Checherita IA (2021) The impact of SARS-CoV-2 infection on mortality in CKD patients-a single-center pilot study. Nephrology Dialysis Transplantation 36(SUPPL 1): i261-i261. 10.1093/ndt/gfab082.0039
[2053] Perkins SJ, Holmes AR, Nelson JR, et al. (2020) Clinical Outcomes among COVID-19 Patients Taking Non-Steroidal Anti-Inflammatory Drugs. In. Vol 76, pp S17-None
[2054] Petelina T, Musikhina N, Avdeeva K, et al. (2021) Prospective analysis of inflammatory response markers, endothelial dysfunction and hemostasis parameters in COVID-19 associated pneumonia patients with and without type 2 diabetes mellitus. European Heart Journal 42: 3394-3394

[2055] Pezel T, Garot P, Hovasse T, et al. (2021) Prognostic value of pre-hospitalization stress perfusion CMR to predict death in patients hospitalized for COVID-19. European Heart Journal Cardiovascular Imaging 22(SUPPL 2): ii20-ii21. 10.1093/ehjci/jeab090.011
[2056] Pfützner A, Frankenberger R (2020) Patients with diabetes have a higher risk of severe COVID-19 progression - Which protective measures are possible? Diabetes, Stoffwechsel und Herz 29(6): 367-371
[2057] Powell JLD (2021) COVID-19 Comorbidities predisposes participants from the Jackson Heart Study to disparate Health Outcomes. The FASEB Journal 35(S1). 10.1096/fasebj.2021.35.S1.03603
[2058] Pradnyana IWAS, Jagannatha GNP, Kamardi S, Yasmin AADA (2021) Characteristics and comorbidities of hypertensive patients in sanglah general hospital during covid-19 pandemic. Journal of Hypertension 39(E-SUPPL 2): e8-e9. 10.1097/01.hjh.0000752480.37878.eb
[2059] Ranjani C, Ananthi AS (2021) Covid-19 in Diabetes mellitus: A Case series in tertiary care centre. Indian Journal of Medical Microbiology 39: S59-S59. 10.1016/J.IJMMB.2021.08.202
[2060] Rapista N, Sarkar S, Singh S, Yalamanchili S, D'Adamo C, Jean LG (2020) CLINICAL INDICATORS OF MORTALITY FOR CORONAVIRUS DISEASE 2019 IN AN URBAN HOSPITAL IN BALTIMORE. Chest 158(4): A336-A336. 10.1016/j.chest.2020.08.333
[2061] Raziq F, Al-abcha A, Kemnic T, Prasad R (2020) CHARACTERISTICS, TREATMENTS, AND OUTCOMES OF COVID-19-INFECTED PATIENTS ADMITTED TO THE ICU OF A TERTIARY CARE CENTER IN LANSING, MICHIGAN. Chest 158(4): A654-A654. 10.1016/j.chest.2020.08.615
[2062] Renouard B, Aleksandrovskiy I, Neumeister J, et al. (2021) Impact of BMI on Outcomes in Patients Hospitalized for COVID-19. Annals of Emergency Medicine 78(2): S6-S7. 10.1016/j.annemergmed.2021.07.012
[2063] Richards KL, McClafferty A, Singh R, Herlihy JP, Senussi MH, Omranian A (2021) Characteristics and outcomes of in-hospital cardiac arrests in patients with SARS-CoV-2 infection. American Journal of Respiratory and Critical Care Medicine 203(9). 10.1164/ajrccm-conference.2021.203.1_MeetingAbstracts.A2597
[2064] Rivera J, Girard C, Kim AI, et al. (2021) Neutrophil-to-lymphocyte ratio in COVID-19 as a predictor for hospital admission, need for ICU admission and 30-day mortality. American Journal of Respiratory and Critical Care Medicine 203(9). 10.1164/ajrccm-conference.2021.203.1_MeetingAbstracts.A2627
[2065] Rodriguez GCFLJKJKECMIADPKDTNKMCLMPNDOMR (2021) Clinical Characteristics and Outcomes Among COVID 19 Hospitalized Patients in a Community Hospital in New York City. Journal of the Endocrine Society 5(Supplement_1): A336-A337. 10.1210/jendso/bvab048.686
[2066] Rodriguez GKECFDPLJKJCMIADKTNKMNDOMRPVDY (2021) Obesity and Covid-19: A Major Mortality Risk in Patients Hospitalized With Covid-19. Journal of the Endocrine Society 5(Supplement_1): A53-A53. 10.1210/jendso/bvab048.106
[2067] Rojas-Fermin RASAGAE, Germosen EMCMLAJ (2021) Clinical Characteristics and Outcomes of Patients with COVID-19 Admitted to the Intensive Care Unit in the Dominican Republic. Open Forum Infectious Diseases 8(Suppl 1): S303-S303
[2068] Rompicherla S, Noor N, Kim A, et al. (2021) Strong association between health insurance type and adverse outcomes for pediatric and adolescent patients with type 1 diabetes and COVID-19;Data from the T1D Exchange COVID Registry. Pediatric Diabetes 22(SUPPL 30): 35-36. 10.1111/pedi.13269
[2069] Rougui L, Weze K, Donaldson SV, Mehari A (2021) Predictors of mortality in minority patients admitted to the ICU with COVID-19 infection. American Journal of Respiratory and Critical Care Medicine 203(9). 10.1164/ajrccm-conference.2021.203.1_MeetingAbstracts.A2545
[2070] Ruzhdii N, Barantcevich E (2021) P.0514 Analysis of post-COVID-19 cognitive functioning in young people with type 1 diabetes mellitus. European Neuropsychopharmacology 53: S379-S380. 10.1016/J.EURONEURO.2021.10.486
[2071] Sacher D, Sehgal S, Pandya A, et al. (2021) Obesity outcomes in acute respiratory distress syndrome due to COVID-19: A retrospective single center experience. American Journal of Respiratory and Critical Care Medicine 203(9). 10.1164/ajrccm-conference.2021.203.1_MeetingAbstracts.A2507
[2072] Santoro F, Vitale E, Nunez Gil I, Guerra F, El-Battrawy I, Brunetti ND (2021) Antiplatelet therapy and outcome in patients with COVID-19. Results from a multi-center international prospective registry (HOPE-COVID19). European Heart Journal 42(SUPPL 1): 3002-3002. 10.1093/eurheartj/ehab724.3002
[2073] Sarantis N, Vlachopoulou M, Chatzis G (2021) SARS-COV-2 (COVID-19) anosmia in patients with obesity: A retrospective study. Atherosclerosis 331: e210-e210. 10.1016/j.atherosclerosis.2021.06.643
[2074] Satapathy SK, Qiu H, Kuntzen C, et al. (2020) Liver injury at hospital admission as predictor for in-hospital mortality in patients with COVID-19. Hepatology 72(1 SUPPL): 286A-286A. 10.1002/hep.31579

[2075] Saulnier P-JHSKLMMHJMWMGPCB (2021) Association between Death at Day 28 and Components of Microvascular Disease of with Diabetes Hospitalized for Coronavirus Disease-2019 (COVID-19): The CORONADO Study. Diabetes 70. 10.2337/db21-1017-P
[2076] Seiglie J, Platt J, Jane Cromer S, et al. (2020) Diabetes, obesity and COVID-19 disease: An observational study of outcomes among hospitalized patients in Boston, Massachusetts. Open Forum Infectious Diseases 7(SUPPL 1): S158-S158. 10.1093/ofid/ofaa417.365
[2077] Selvarajan RJBVR (2021) An Observational Study on Glycemic Outcomes, Lifestyle and Psychosocial Health of Patients With Diabetes During Covid-19 Lockdown From Bangalore. Journal of the Endocrine Society 5(Supplement_1): A335-A336. 10.1210/jendso/bvab048.684
[2078] Shabaka A, Gruss E, Landaluce-Triska E, et al. (2021) Thrombotic events after COVID-19 infection in hemodialysis patients∗. Nephrology Dialysis Transplantation 36(SUPPL 1): i409-i409. 10.1093/ndt/gfab097.001
[2079] Shallal A, Morrison AR, Brar I, Suleyman G (2020) Complications and outcomes of obese patients hospitalized with COVID-19. Open Forum Infectious Diseases 7(SUPPL 1): S248-S248. 10.1093/ofid/ofaa417.552
[2080] Sharma A, Rahar S, Sharma M, Baveja G, Kumar V (2021) Study of Clinical Profile, Hematologic Parametersand Outcomes of COVID-19 Patients in a Tertiary Care Centre in North India. Indian Journal of Hematology and Blood Transfusion 37(SUPPL 1): S107-S107. 10.1007/s12288-021-01510-0
[2081] Sher N, Rangan P, Agarwal S, Heise C, Uppalapu S, raschke R (2021) PREDICTOR VARIABLES FOR MORTALITY IN THE 48 HOURS PRIOR TO ENDOTRACHEAL INTUBATION IN PATIENTS WITH COVID-19 PNEUMONIA. Chest 160(4): A528-A528. 10.1016/j.chest.2021.07.515
[2082] Shestakova MV, Kononenko IKZZAMN (2021) HbA1c Level in Severe COVID-19 Patients Is Not Only a Marker of Glycemic Status but Also of Acute Erythrocyte Damage. Diabetes 70. 10.2337/db21-1002-P
[2083] Shi MYALESWHFBKAP, Luk AMRC, Chan JC, Chow E (2021) Long-Term Renin-Angiotensin System Inhibitor Use and Risk of Pneumonia and Pneumonia-Related Death in Type 2 Diabetes. Diabetes 70. 10.2337/db21-1071-P
[2084] Simbaqueba C, Mamlouk O, Dickson K, et al. (2021) Acute kidney injury and major outcomes in cancer patients with no history of chronic kidney disease (CKD) with coronavirus 2019 (COVID-19) infection. Journal of Clinical Oncology 39(15 SUPPL). 10.1200/JCO.2021.39.15_suppl.e18816
[2085] Sinha AK, Pandey DMMTRK, Kumar RSSK (2021) Mortality and Length of Hospitalisation amongst Diabetic and Nondiabetic COVID-19 Critical Patients: A Retrospective Cohort Study. Diabetes 70. 10.2337/db21-1021-P
[2086] Sinvani LMAMAZMCMDMDKBE (2020) Age Does Not Predict Mortality in Hospitalized COVID-19+ Older Adults: Rethinking Resource Allocation Based on Age. Innovation in Aging 4(Supplement_1): 933-933. 10.1093/geroni/igaa057.3419
[2087] So M, Takahashi M, Egorova N, Kuno T (2021) The association of statins use with survival of covid-19 patients. Circulation 144(SUPPL 1). 10.1161/circ.144.suppl-1.9678
[2088] So M, Takahashi M, Egorova N, Kuno T (2021) The association of obesity level and inhospital survival of patients with covid-19 who received corticosteroids. Circulation 144(SUPPL 1). 10.1161/circ.144.suppl-1.10280
[2089] Sprockel J, Murcia AL, Rincon JM, et al. (2021) High sensitivity troponin as a 60-day mortality marker in patients with COVID-19 in intensive care at a Latin American hospital. European Heart Journal 42(SUPPL 1): 1525-1525. 10.1093/eurheartj/ehab724.1525
[2090] Stavileci B, Ozdemir E (2021) The impact of daily troponin-I and D-dimer serum levels on mortality of moderate-to-severe COVID-19 pneumonia patients. European Heart Journal 42(SUPPL 1): 3276-3276. 10.1093/eurheartj/ehab724.3276
[2091] Sultana A, Mahendra M, Bhoomika RS, Nuchin A, Pandey A, Shreedhar S (2021) Clinical profile and laboratory parameters in patients with moderate-severe COVID-19 pneumonia admitted in tertiary care hospital in South India. Indian Journal of Critical Care Medicine 25
[2092] Sun CKKYAMAMRBCCC (2021) Is DPP-4 Use Associated With a Decreased Mortality for COVID-19 Diabetic Patients? A Meta-Analysis. Journal of the Endocrine Society 5(Supplement_1): A347-A348. 10.1210/jendso/bvab048.708
[2093] Suresh A, Srivastava GN (2021) Clinicodemographic profile and outcomes of severe COVID-19 infection in young adults. Indian Journal of Critical Care Medicine 25
[2094] Taweesedt P, Mominkhawaja A, Pattharanitima P, Varon J, Sharma M, Surani S (2021) RESTRICTIVE LUNG DEFECT IN POST-COVID-19 PNEUMONIA. Chest 160(4): A579-A579. 10.1016/j.chest.2021.07.557
[2095] Tee SA, Devine K, Potts A, et al. (2021) COVID-19 in patients with diabetes: Real-world data on factors influencing outcome. British J Diabetes 21(1): 7-None. 10.15277/bjd.2021.300

[2096] Thammavaranucupt K, Jayanama K, Sungkanuparph S, Pathumarak A, Thotsiri S, Suppadungsuk S (2020) Acute kidney injury in COVID-19: Sharing experience from Chakri Naruebodindra Medical Institute. Nephrology 25(SUPPL 4): 27-28. 10.1111/nep.v25.S4
[2097] Thepmankorn P, Heshmati K, Ruane C, et al. (2021) Neurological Manifestations of SARS-CoV-2 Infection in Type 2 Diabetes. Neurology 96(15)
[2098] Tomanoski V, Gjorgjievska G, Nakovska M, et al. (2021) Risk factors for covid-19 mortality in hemodialysis patients. Nephrology Dialysis Transplantation 36(SUPPL 1): i459-i460. 10.1093/ndt/gfab098.0011
[2099] Uribe JPAAMAKFARPPCAPM (2020) BASELINE CHARACTERISTICS AND MORTALITY IN PATIENTS WITH SARS-COV-2 CONFIRMED INFECTION: SINGLE MAJOR ACADEMIC CENTER EXPERIENCE. Chest 158(4): A296-A297. 10.1016/J.CHEST.2020.08.296
[2100] Varshney KGPIR (2021) Risk Factors for COVID-19 Mortality Among People Living with HIV. Health Services Research 56(S2): 84-85. 1111/1475-6773.13843
[2101] Veloz MG, Del Pino P, Ruiz PC, et al. (2020) Influence of pre-existing liver disease in the course of COVID-19. in an area with low incidence of SARSCOV2 infection. Hepatology 72(1 SUPPL): 281A-282A. 10.1002/hep.31579
[2102] Venkata VS, Kiernan G (2020) COVID-19 AND COPD: POOLED ANALYSIS OF OBSERVATIONAL STUDIES. Chest 158(4): A2469-A2469. 10.1016/j.chest.2020.09.046
[2103] Verma A, Chaudhary S (2021) Sociodemographic, clinical and morbidity factors associated with COVID 19 mortality in hospitalized patients: A retrospective observational study. Journal of Hypertension 39(SUPPL 1): e202-e202. 10.1097/01.hjh.0000746720.89890.ca
[2104] Vilar-Compte DDLRMDM-OAP-JCI-MBA-FPC-JPA-AMZ-PPN-L (2021) SARS-CoV-2 Infection in a Referral Cancer Center in Mexico City During the First Year of the COVID-19 Pandemic. Open Forum Infectious Diseases 8(Suppl 1): S255-S255
[2105] Virmani SMJERKTARKM (2021) Abstract #1003696: Diabetes and COVID-19: Identifying Diabetes-Related Predictors of Severe Infection. Endocrine Practice 27(6, Supplement): S50-S50. 10.1016/j.eprac.2021.04.577
[2106] Vlachopoylou M, Hamissa EM, AbdAlgaffar SA, Chatzis G (2021) COVID 19 and the Systematic Use of Aspirin in Diabetics During Hospitalization: A Retrospective Study. American Heart Journal 242: 152-152. 10.1016/j.ahj.2021.10.017
[2107] Volk BM, Roberts CGP, Vantieghem M, et al. (2021) COVID-19 Severity in a Geographically Diverse, US-based, Ambulatory Population with Type 2 Diabetes on a Medically Supervised Ketogenic Diet. Diabetes 70. 10.2337/db21-40-LB
[2108] Vyas A, Khan MH, Husain K, Singh L, Hafiz N, Papayannis I (2021) COVID-19 Infection and Type 2 Diabetes Mellitus: A Retrospective Study. American Journal of Gastroenterology 116: S1327-S1328
[2109] Wang SY, Adejumo P, Onuma OK, Miller EJ, Spatz ES (2021) Characteristics of patients referred to a cardiovascular disease clinic for post-acute sequelae of sars-cov-2 infection. Circulation 144(SUPPL 1). 10.1161/circ.144.suppl_1.11639
[2110] Weinberger J, Rhee C, Klompas M (2021) Incidence, characteristics, and outcomes of ventilator associated events during the COVID-19 pandemic. American Journal of Respiratory and Critical Care Medicine 203(9). 10.1164/ajrccm-conference.2021.203.1_MeetingAbstracts.A2607
[2111] Weizman O, Mika D, Geneste L, et al. (2021) Cardiovascular Comorbidities and Covid-19 in Women. Archives of Cardiovascular Diseases Supplements 13(1): 121-122. 10.1016/j.acvdsp.2020.10.254
[2112] Werneke U, Maripuu M, Bendix M, Öhlund L, Widerstrom M (2021) Death associated with coronavirus (COVID-19) infection in individuals with severe mental disorders in sweden during the early months of the outbreak. European Psychiatry 64(S1): S119-S120. 10.1192/j.eurpsy.2021.339
[2113] Wojnowski K, Nettboy S, Kumar N, Koru-Sengul T, Kumar SD (2021) Sum score to predict need for mechanical ventilation in patients with COVID-19. American Journal of Respiratory and Critical Care Medicine 203(9). 10.1164/ajrccm-conference.2021.203.1_MeetingAbstracts.A2636
[2114] Xiao S, Lanfear D, Williams L (2021) Factors associated with COVID-19 infection and severity in a large insured patient population in metropolitan detroit. American Journal of Respiratory and Critical Care Medicine 203(9). 10.1164/ajrccm-conference.2021.203.1_MeetingAbstracts.A1486
[2115] Ye Y, Yue X, Krueger W, Wegrzyn L (2021) Characteristics and outcomes in a realworld cohort of rheumatoid arthritis patients with covid-19. Annals of the Rheumatic Diseases 80(SUPPL 1): 860-860. 10.1136/annrheumdis-2021-eular.565
[2116] Yescas Garibay KS, Arquiette J, Padilla R, Singh G (2021) Clinical characteristics of patients with sars-cov-2 infection in san joaquin valley: A single center experience. American Journal of Respiratory and Critical Care Medicine 203(9). 10.1164/ajrccm-conference.2021.203.1_MeetingAbstracts.A3807
[2117] Yethindra V, Tagaev T, Zhumabekova A, et al. (2021) Evaluation of Cardiovascular Diseases and Diabetes Mellitus as Predisposing Factors for Mortality in Patients with Coronavirus Disease

2019: Systematic Review and Meta-Analysis. American Heart Journal 242: 146-146. 10.1016/j.ahj.2021.10.003
[2118] Yoo J, Papamanoli A, Grewal P, et al. (2020) N-Terminal Pro-B-Type Natriuretic Peptide and Mortality in Patients without Heart Failure Hospitalized with Severe Covid-19 Pneumonia. Journal of cardiac failure 26(10, Supplement): S73-S73. 10.1016/j.cardfail.2020.09.213
[2119] Almeida CAP, De Oliveira MFA, Sandoval Cabrera CP, et al. (2021) Risk factors for RRT and mortality in patients with COVID-19-related AKI in São Paulo, Brazil. Journal of the American Society of Nephrology 32: 70-70
[2120] Blitshteyn S, Brook J, Minen M, et al. (2021) COVID-19 in patients with pre-existing neurologic disorders: Clinical course and outcomes. Neurology 96(15 SUPPL 1)
[2121] Lozano LEC, Ruiz-Gonzalez MA (2021) Survival predictors of severe SARSCoV-2 pneumonia in the intensive care unit: Single-center study. Critical Care Medicine 49(1 SUPPL 1): 101-101. 10.1097/01.ccm.0000726816.40463.f4
[2122] Gaggero G, Viglino F, Beccuti G, Bertaina S, Broglio F (2021) IMPROVED CMG OUTCOMES IN ADULTS WITH TYPE 1 DIABETES MELLITUS DURING COVID-19 LOCKDOWN IN PIEDMONT, ITALY. Diabetes Technology & Therapeutics 23: A199-A199
[2123] Lombardi R, Ferreiro A, Aroca G, et al. (2021) KIDNEY INVOLVEMENT IN SARS-Co-V-2 INFECTION. A LATIN AMERICAN REGISTRY. Kidney International Reports 6(4): S16-S16. 10.1016/J.EKIR.2021.03.043
[2124] Schaenman J, Byford H, Grogan TR, et al. (2021) Covid-19 infection in kidney versus non-kidney solid organ transplant recipients: A single center study. American Journal of Transplantation 21(SUPPL 4): 622-623. 10.1111/ajt.16848
[2125] Sharma P, Chen V, Fung CM, et al. (2020) COVID-19 outcomes among solid organ transplant recipients: A matched case-control study. Hepatology 72(1 SUPPL): 269A-270A. 10.1002/hep.31579
[2126] Tallon E, Ebekozien O, Sanchez J, et al. (2021) Factors associated with hospitalization in youths and young adults with type 2 diabetes and COVID-19 infection. Pediatric Diabetes 22(SUPPL 30): 34-34. 10.1111/pedi.13269
[2127] Bramante C (2021) Associations with metformin and COVID-19 in the N3C database: A national retrospective cohort study. Obesity 29(SUPPL 2): 64-64. 10.1002/oby.23329
[2128] Al Shamy T, Moctezuma S, Ho K, et al. (2021) The relationship between obesity classifications and outcomes in COVID-19 infection in New York. Obesity 29(SUPPL 2): 178-178. 10.1002/oby.23329
[2129] Bengualid V, Martinez M, Hysenaj Z, Willner DM, Berger J (2020) Clinical characteristics of the first 177 patients admitted with COVID-19 at a bronx community hospital. Open Forum Infectious Diseases 7(SUPPL 1): S254-S254. 10.1093/ofid/ofaa417.564
[2130] Adelsheimer A, Choi J, Evans A (2021) Antiplatelet therapy and covid-19 outcomes in patients with stable coronary artery disease. Circulation 144(SUPPL 1). 10.1161/circ.144.suppl-1.9672
[2131] Aziz F, Brauer A, Ciardi C, et al. (2021) Predictive performance of Simplified Acute Physiology Score 3 in COVID-19 patients with and without diabetes in Austria. Wiener Klinische Wochenschrift 133(SUPPL 6): 223-224
[2132] Kadapatti K, Shendge J, Kulkarni A, et al. (2021) Analysis of mortality in COVID-19 patients admitted to an intensive care unit. Indian Journal of Critical Care Medicine 25
[2133] Nicholas SB, Follett RW, Tacorda TT, et al. (2021) Disparities in CKD risks: Data from the cure-CKD COVID-19 sub-registry. Journal of the American Society of Nephrology 32: 84-84
[2134] Hiles H, Morgan A, Gandhi R, Greig M (2021) Coronavirus disease (covid-19) mortality outcomes in a large teaching hospital in patients with diabetic foot ulceration: A retrospective observational cohort study. Diabetic Medicine 38
[2135] Al-Baka HAA (2022) Diabetes and COVID-19 severity. 3rd International Scientific Conference of Alkafeel University, ISCKU 2021 2386. 10.1063/5.0067026
[2136] Mamlouk O, Turin A, D'Achiardi D, et al. (2021) Clinical characteristics and outcomes of cancer patients with chronic kidney disease and coronavirus disease 2019. Journal of Clinical Oncology 39(15 SUPPL). 10.1200/JCO.2021.39.15_suppl.e18815
[2137] Sonia GBEA, Zulueta MAEDLHAB, Mendizabal LDOJMRSLCL (2021) Association of Serum 1,5-Anhydroglucitol with Severe COVID-19 in T2D patients. Diabetes 70. 10.2337/db21-146-LB
[2138] Pines-Corrales PJ, Molina M, Garcia-Blasco L, et al. (2021) Identification of Specific Risk Factors for Mortality in Patients with Diabetes Mellitus Hospitalized with COVID-19. Diabetes 70. 10.2337/db21-172-LB
[2139] Bassi M, Minuto N, Montobbio C, et al. (2021) GLYCEMIC CONTROL IN YOUNG ITALIAN PATIENTS WITH TYPE 1 DIABETES DURING COVID-19 LOCKDOWN: THE ROLE OF AGE, TYPE OF INSULIN THERAPY, TELEMEDICINE AND PHYSICAL ACTIVITY. Diabetes Technology & Therapeutics 23: A109-A110

[2140] Gupta D, Dewan S, Chauhan M, Jain A (2021) Retrospective observational analysis of inflammatory markers in COVID-19 patients admitted to intensive care unit: As early predictors of disease severity. Indian Journal of Critical Care Medicine 25
[2141] Button E, Ishibashi H, Bliden K, et al. (2021) 1,5-anhydroglucitol is a novel and independent predictor of mortality in patients with covid-19. Circulation 144(SUPPL 1). 10.1161/circ.144.suppl-1.12380
[2142] Shahid Z, Baldrige E, Trufan S, et al. (2021) Risk factors for hospitalization for cancer patients with SARS-CoV-2 infection. Journal of Clinical Oncology 39(15 SUPPL). 10.1200/JCO.2021.39.15_suppl.e18753
[2143] Lebdeh HA, Nanda S (2021) Obesity and severity of COVID-19 infection. Obesity 29(SUPPL 2): 131-131. 10.1002/oby.23329
[2144] Manmathan G, Falconer D, Abdi Z, et al. (2021) Myocardial injury as a predictor of mortality and adverse outcomes in COVID-19. Topics in Antiviral Medicine 29(1): 208-209
[2145] Mateen P, Alsaei A, Onyambu S, Javed MA, Khan A, Sadaka F (2021) The association between HbA1C and outcomes in COVID-19 patients. Critical Care Medicine 49(1 SUPPL 1): 47-47. 10.1097/01.ccm.0000726388.65962.42
[2146] Peterson J, Tam TA, Riehl-Tonn V, et al. (2021) Prospective study of COVID-19 in patients receiving dialysis in Alberta kidney care south. Journal of the American Society of Nephrology 32: 75-75
[2147] Sengar PS, Jha RK, Verma S (2021) Comparison between markers of coagulation dysfunction and inflammation in diabetic and non-diabetic COVID-19. Indian Journal of Critical Care Medicine 25
[2148] Lu M, Rupp LB, Boscarino JA, et al. (2020) Impact of history of chronic viral hepatitis and liver fibrosis on risk of hospitalization and death among patients with SARS-COV-2 infection. Hepatology 72(1 SUPPL): 280A-281A. 10.1002/hep.31579
[2149] Windham SL, Wilson MP, Fling C, et al. (2020) Relationship between patient characteristics and critical illness in patients admitted for COVID-19. Open Forum Infectious Diseases 7(SUPPL 1): S266-S267. 10.1093/ofid/ofaa417.591
[2150] Kreit H, Mossayebi A, Cherukuri S, et al. (2021) Kidney and liver dysfunction predicts COVID-19 severity and length of hospitalization. Obesity 29(SUPPL 2): 121-121. 10.1002/oby.23329
[2151] Yen TE, Kim A, Rutherford H, Ratnaparkhi S, Woolley AE, McCausland FR (2021) Serum sodium and patient symptoms in COVID-19 hospitalizations. Journal of the American Society of Nephrology 32: 106-106
[2152] (2021) 20% of hospitalised COVID-19 patients with diabetes died within 28 days. Medical Brief 2021(0342)
[2153] Abobaker A, Darrat M (2021) The association between biochemically confirmed thyroid gland disorder and morbidity and mortality in patients with COVID-19. J Med Virol 93(12): 6449-6450. 10.1002/jmv.27213
[2154] Ad'hiah AH, Al-Bayatee NT, Ahmed AA (2021) Coronavirus disease 19 and risk of hyperglycemia among Iraqi patients. Egyptian Journal of Medical Human Genetics 22(1): 1-4. 10.1186/s43042-021-00207-3
[2155] Alfishawy M, Elbendary A, Younes A, et al. (2021) Diabetes mellitus and Coronavirus Disease (Covid-19) Associated Mucormycosis (CAM): A wake-up call from Egypt. Diabetes Metab Syndr 15(5): 102195. 10.1016/j.dsx.2021.102195
[2156] Amarasekera N, Sarker P, Manoharan S (2021) Is there a link between the prevalence of cardiovascular disease, diabetes, kidney disease, chronic obstructive pulmonary disease and malignancies and the severity of COVID-19 within the BAME population? Clin Med (Lond) 21(Suppl 2): 46. 10.7861/clinmed.21-2-s46
[2157] Anonymous (2021) "Diabetes and COVID-19" Special Article Collection. Diabetes Care 44(7): 1726-1726. 10.2337/dc21-ie07
[2158] Austin U (2021) Prior Metformin Use in Patients with Diabetes Hospitalized for COVID-19. Internal Medicine Alert 43(4)
[2159] Bergmann A, Schwarz PEH (2020) Diabetes und Covid-19. Diabetes Aktuell 3(18): 85-85. 10.1055/a-1126-5160
[2160] Beyan C, Beyan E (2021) Mean platelet volume may not be responsible for increased male mortality in diabetic patients with severe coronavirus disease. Prim Care Diabetes 15(5): 892. 10.1016/j.pcd.2021.07.014
[2161] Blake A, Collins D, O'Connor E, Bergin C, McLaughlin AM, Martin-Loeches I (2020) Clinical and biochemical characteristics of patients admitted to ICU with SARS-CoV-2. Med Intensiva (Engl Ed) 44(9): 589-590. 10.1016/j.medin.2020.05.003
[2162] Burki T (2022) COVID-19 and diabetes in Africa: a lethal combination. Lancet Diabetes Endocrinol 10(1): 23. 10.1016/s2213-8587(21)00315-6

[2163] Carr MJ, Wright AK, Leelarathna L, et al. (2021) Impact of COVID-19 on diagnoses, monitoring, and mortality in people with type 2 diabetes in the UK. Lancet Diabetes Endocrinol 9(7): 413-415. 10.1016/s2213-8587(21)00116-9
[2164] Cook M (2021) Potential factors linked to high COVID-19 death rates in British minority ethnic groups. Lancet Infect Dis 21(4): e68-e68. 10.1016/S1473-3099(20)30583-1
[2165] Davidson R (2021) Metformin reduces death risk in covid-19 patients with diabetes. US Pharm 46: 3-3
[2166] Fischer S (2020) SARS-CoV-2 and diabetes. Arzneimitteltherapie 38(5): 175-175
[2167] Franke K (2021) COVID-19: Sitagliptin verbessert Prognose bei Patienten mit Diabetes mellitus. Diabetologie Und Stoffwechsel 16(2): 87-87. 10.1055/a-1387-6213
[2168] Fritschka PDmE (2020) Diabetes ist Risikofaktor für akutes Nierenversagen bei COVID-19. Info Diabetologie(4). 10.1007/s15034-020-2168-0
[2169] Geissel W (2021) SARS-CoV-2 und Diabetes: Weniger COVID-19-Tote bei Therapie mit Metformin. CardioVasc 21(2): 20-20. 10.1007/s15027-021-3494-4
[2170] Gregory JM, Moore DJ (2021) Letter to the Editor from Justin M. Gregory: Age and Hospitalization Risk in People With Type 1 Diabetes and COVID-19: Data From the T1D Exchange Surveillance Study. J Clin Endocrinol Metab. 10.1210/clinem/dgab871
[2171] Gregory JM, Slaughter JC, Duffus SH, et al. (2021) Response to Comment on Gregory et al. COVID-19 Severity Is Tripled in the Diabetes Community: A Prospective Analysis of the Pandemic's Impact in Type 1 and Type 2 Diabetes. Diabetes Care 2021;44:526-532. Diabetes Care 44(5): e103-e104. 10.2337/dci21-0005
[2172] Hadjadj S, Wargny M (2021) Glucose-lowering treatments and COVID-19 mortality in T2DM. Nat Rev Endocrinol 17(7): 387-388. 10.1038/s41574-021-00509-x
[2173] Heald AH, Jenkins DA, Chaudhury N, et al. (2022) SARS-CoV-2, diabetes and mortality: month by month variation in mortality rate from June 2020 to June 2021. Cardiovasc Endocrinol Metab 11(1): e0258. 10.1097/xce.0000000000000258
[2174] Heald AH, Jenkins DA, Chaudhury N, et al. (2022) Application of a city wide digital population database for outcome analysis in diabetes: SARS-CoV-2, diabetes and hospital admission rate month by month in Greater Manchester, UK. Cardiovasc Endocrinol Metab 11(1): e0257. 10.1097/xce.0000000000000257
[2175] Heald AH, Livingston M, Moreno G, Gibson JM (2021) The risk factors potentially influencing risk of serious illness/death in people with diabetes, following SARS-CoV-2 infection: What needs to be done from here. Cardiovasc Endocrinol Metab 10(4): 231-232. 10.1097/xce.0000000000000255
[2176] Huang Y, Tu M, Wang S, et al. (2020) Clinical characteristics of laboratory confirmed positive cases of SARS-CoV-2 infection in Wuhan, China: A retrospective single center analysis. Travel Med Infect Dis 36: 101606. 10.1016/j.tmaid.2020.101606
[2177] Jayasinghe S, Misra A, Hills AP (2021) Post-COVID-19 syndrome and type 2 diabetes: Primacy of exercise in prevention and management. Diabetes Metab Syndr 16(1): 102379. 10.1016/j.dsx.2021.102379
[2178] Kautzky-Willer A (2021) Sex disparities in COVID-19 outcomes of diabetic patients. European journal of endocrinology. 10.1530/EJE-21-0721
[2179] Kow CS, Ramachandram DS, Hasan SS (2021) Statin use in patients with diabetes amid COVID-19 pandemic. Diabetes Metab 48(1): 101298. 10.1016/j.diabet.2021.101298
[2180] Kow CS, Ramachandram DS, Hasan SS (2021) Use of aspirin for primary prevention in patients with diabetes during the COVID-19 pandemic. Ir J Med Sci: 1-2. 10.1007/s11845-021-02787-w
[2181] Lopez-Huamanrayme E, Cordova-Huancas C, Garate-Chirinos D, Espinoza-Morales F, Pasquel F (2021) Diabetic ketoacidosis and high mortality among patients with coronavirus disease 2019 in a Peruvian hospital. J Diabetes Investig. 10.1111/jdi.13704
[2182] Mahrooz A (2021) Insulin resistance as a common clinical feature in diabetes mellitus, obesity, hypertension, dyslipidemia, and atherosclerosis deserves more attention in COVID-19. J Res Med Sci 26: 98. 10.4103/jrms.JRMS_1063_20
[2183] Panagiotou G, Tee SA, Ihsan Y, et al. (2020) Original publication: Low serum 25-hydroxyvitamin D (25[OH]D) levels in patients hospitalized with COVID-19 are associated with greater disease severity. Clin Endocrinol (Oxf) 93(5): 629-630. 10.1111/cen.14310
[2184] Pérez B F (2020) Coronavirus y diabetes. Rev chil endocrinol diabetes 13(2): 41-41
[2185] Rao S, Ali K, Rivas M, Nugent K (2021) Diabetic Ketoacidosis in Patients With COVID-19. Am J Med Sci 361(5): 668-669. 10.1016/j.amjms.2020.11.027
[2186] Rhee SY (2021) Effects of a DPP-4 Inhibitor and RAS Blockade on Clinical Outcomes of Patients with Diabetes and COVID-19 (Diabetes Metab J 2021;45:251-9). Diabetes Metab J 45(4): 619-620. 10.4093/dmj.2021.0118

[2187] Rosenthal JA, Awan SF, Fintzi J, Keswani A, Ein D (2021) Asthma is associated with increased risk of intubation but not hospitalization or death in coronavirus disease 2019. Ann Allergy Asthma Immunol 126(1): 93-95. 10.1016/j.anai.2020.10.002
[2188] Routen A, O'Mahoney L, Ayoubkhani D, et al. (2022) Understanding and tracking the impact of long COVID in the United Kingdom. Nat Med 28(1): 11-15. 10.1038/s41591-021-01591-4
[2189] Sardu C, D'Onofrio N, Balestrieri ML, et al. (2020) Hyperglycaemia on admission to hospital and COVID-19. Diabetologia 63(11): 2486-2487. 10.1007/s00125-020-05216-2
[2190] Sathish T (2022) Risk of mortality in COVID-19 patients with newly diagnosed and pre-existing diabetes. Prim Care Diabetes 16(1): 214. 10.1016/j.pcd.2021.12.002
[2191] Seligmann G, Ponnampalam J, Pimento D, Little C (2021) The association of hypertension and diabetes pharmacotherapy with COVID-19 severity and immune signatures: an observational study response. European Heart Journal-Cardiovascular Pharmacotherapy 7(3): E52-E52. 10.1093/ehjcvp/pvaa112
[2192] Shamsoddin E (2021) Is periodontitis associated with the severity of COVID-19? Evid Based Dent 22(2): 66-68. 10.1038/s41432-021-0179-x
[2193] Stiefelhagen P (2020) Je höher der Blutzucker, desto gefährlicher die SARS-CoV-2-Infektion : COVID-19 bei Diabetikern. MMW Fortschr Med 162(20): 15-17. 10.1007/s15006-020-4593-3
[2194] Waterman A (2020) Diabetes and COVID-19 -a collaborative response. Kai Tiaki Nursing New Zealand 26(6): 16-18
[2195] Woolcott OO, Castilla-Bancayan JP (2021) Letter to the Editor From Woolcott and Castilla-Bancayan: "Diabetes Increases Severe COVID-19 Outcomes Primarily in Younger Adults: Age and Diabetes in COVID-19 Severity". Journal of Clinical Endocrinology & Metabolism 106(12): E5273-E5274. 10.1210/clinem/dgab582
[2196] Yan AF, Sun XM, Zheng JE, et al. (2021) Perceived risk, behavior changes and Health-related outcomes during COVID-19 pandemic: Findings among adults with and without diabetes in China (vol 167, 108350, 2020). Diabetes Research and Clinical Practice 177. 10.1016/j.diabres.2021.108881
[2197] Yasri S, Wiwanitkit V (2021) D-dimer, COVID-19 and Diabetes: A Correspondence. Clin Appl Thromb Hemost 27: 10760296211061149. 10.1177/10760296211061149
[2198] Iniguez M, Perez-Matute P, Villoslada-Blanco P, et al. (2021) ACE Gene Variants Rise the Risk of Severe COVID-19 in Patients With Hypertension, Dyslipidemia or Diabetes: A Spanish Pilot Study (vol 12, 688071, 2021). Frontiers in Endocrinology 12. 10.3389/fendo.2021.771445
[2199] Bhadada SK, Pal R (2020) Diabetes mellitus and COVID-19: The ominous duo. International Journal of Noncommunicable Diseases 5(3): 99-101. 10.4103/jncd.jncd_65_20
[2200] Pfützner A, Sorsa T, Nwhator SO, et al. (2020) Why do people with diabetes have a higher risk of severe progressions of a SARS-CoV-2 infection - and what could this have to do with oral hygiene? Diabetes, Stoffwechsel und Herz 29(4): 234-239
[2201] (2022) BLOOD SUGAR HISTORY PROVES VITAL FOR PEOPLE WITH DIABETES WHO CATCH COVID. Community Practitioner 95(1): 12-12
[2202] Alonso NBS (2021) Diabetes mellitus and SARS-CoV-2 infection. Clínica e Investigación en Arteriosclerosis (English Edition) 33(3): 148-150. 10.1016/J.ARTERE.2021.06.003
[2203] (2021) Metformin may reduce mortality risk 3x in patients with COVID-19 and diabetes. Medical Brief 2021(0336)
[2204] Assiut U (2021) Epidemology of Acute Kidney Injury in Diabetic Patients Infected With covid_19 In Assiut University Hospital. clinicaltrialsgov
[2205] Cairo U (2021) COVID-19 Infection in Diabetic Patients. In:
[2206] Darent Valley H (2021) Effect of COVID-19 on diabetes in Black Asian and Minority Ethnic (BAME) patients. ISRCTN registry. 10.1186/ISRCTN15351917
[2207] Fundación Investigación Biomédica Hospital Ramón y C (2020) Controlled, randomized, non-blind trial on the usefulness of pioglitazone treatment in patients with type 2 diabetes mellitus and COVID-19. Ensayo controlado, aleatorizado, no ciego, sobre la utilidad del tratamiento con pioglitazona en pacientes con diabetes mellitus tipo 2 y COVID-19. EU Clinical Trials Register
[2208] Glasgow NHSG, Clyde (2021) Long term effects of COVID-19 in people with diabetes. ISRCTN registry. 10.1186/ISRCTN14643181
[2209] Instituto Nacional de Ciencias Medicas y Nutricion Salvador Z (2020) Continuous Glucose Monitorization in Hospitalized Patients With COVID-19. clinicaltrialsgov
[2210] Mukesh K (2021) Comparison of the serum ferritin level with diabetic and non-diabetic Covid-19 patients. In:
[2211] Patrycja Lesnik MDP (2021) Incidence of COVID-19 disease and the severity of SARS-CoV-2 infection and relationship with angiotensin II type 1 receptor (ATII-1R) concentration in healthcare professionals. In:
[2212] The University of A (2021) Postural orthostatic tachycardia syndrome (POTS) in Long COVID syndrome: A detailed profiling study. In:

[2213] Maulana Azad Medical C (2021) Severity of covid-19 disease in diabetes mellitus patients. CTRI
[2214] Beijing Ditan Hospital Affiliated to Capital Medical U (2021) Effect of fasting blood plasma level on COVID-19 patients. In:
[2215] University of M (2020) Incidence and Predictors of Acute and Chronic Complications in Patients With Diabetes and Severe COVID-19. clinicaltrialsgov
[2216] Self (2021) LDH and Ferritin levels in Diabetes Mellitus. In:
[2217] Physiotherapy MGMIO (2021) Home Based Rehabilitation On Cardio-Respiratory Fitness In Post Covid-19 Type II Diabetes Mellitus Patients-Randomized Controlled Trail. In:
[2218] Bonora BM, Fogar P, Zuin J, et al. (2022) Hyperglycemia, Reduced Hematopoietic Stem Cells, and Outcome of COVID-19. Diabetes. 10.2337/db21-0965
[2219] Chen SLWWJXBCLCG (2020) Is GLP-1R Agonists Effective and Safe in Severe COVID-19 Patients with Type 2 Diabetes? - A Case Report and Literature Review. In:
[2220] Hertanto DM, Sutanto H, Adi S (2021) Case Report: Diabetic nephropathy aggravates the progression and prognosis of COVID-19-associated acute limb ischemia. F1000Res 10: 584. 10.12688/f1000research.54193.2
[2221] Pranave P, Kshirsagar RA, Sardeshmukh A, Warade P, Mishra P (2021) Post COVID-19 Mucormycosis in Immunocompromised Individuals with Uncontrolled Diabetes Mellitus: A Series of Seven Cases. Journal of Clinical and Diagnostic Research 15(11): ZR01-ZR06. 10.7860/jcdr/2021/50087.15646
[2222] Andreas M, Romansyah MA, Zuandra RA (2020) Laporan Kasus Silent Hypoxemia Pada Penderita COVID-19 Dengan Komorbid Diabetes Melitus. In. Vol 7
[2223] Alonso N, Batule S (2021) COVID-19 and diabetes mellitus. Importance of glycemic control. Clin Investig Arterioscler 33(3): 148-150. 10.1016/j.arteri.2021.05.001
[2224] Ng PY, Dalton HJ, White N, Fraser JF (2021) Characteristics and outcomes of patients with COVID-19 admitted to intensive care units-a report from the global COVID-19 critical care consortium. Perfusion 36(1 SUPPL): 39-39. 10.1177/02676591211007763
seems to be an conferecne abstract;
[2225] Aggarwal G, Lippi G, Lavie CJ, Henry BM, Sanchis-Gomar F (2020) Diabetes Mellitus Association with Coronavirus Disease 2019 (COVID-19) Severity and Mortality: A Pooled Analysis. J Diabetes. 10.1111/1753-0407.13091
[2226] Belkhadir J (2020) COVID-19 and diabetes from IDF MENA region. Diabetes Res Clin Pract 166: 108277. 10.1016/j.diabres.2020.108277
[2227] Caballero AE, Ceriello A, Misra A, et al. (2020) COVID-19 in people living with diabetes: An international consensus. J Diabetes Complications 34(9): 107671. 10.1016/j.jdiacomp.2020.107671
[2228] Cappuccio FP, Siani A (2020) Covid-19 and cardiovascular risk: Susceptibility to infection to SARS-CoV-2, severity and prognosis of Covid-19 and blockade of the renin-angiotensin-aldosterone system. An evidence-based viewpoint. Nutr Metab Cardiovasc Dis 30(8): 1227-1235. 10.1016/j.numecd.2020.05.013
[2229] Longo M, Caruso P, Maiorino MI, Bellastella G, Giugliano D, Esposito K (2020) Treating type 2 diabetes in COVID-19 patients: the potential benefits of injective therapies. Cardiovasc Diabetol 19(1): 115. 10.1186/s12933-020-01090-9
[2230] Naruse K (2020) Does glycemic control rescue type 2 diabetes patients from COVID-19-related deaths? Journal of Diabetes Investigation 11(4): 792-794. 10.1111/jdi.13320
[2231] Agarwal S, Agarwal SK (2020) Endocrine changes in SARS-CoV-2 patients and lessons from SARS-CoV. Postgraduate medical journal. 10.1136/postgradmedj-2020-137934
[2232] Ahirwar AK, Asia P, Sakarde A, Kaim K (2020) COVID -19 outbreak – Diabetes aspect and perspective. Current Medicine Research and Practice. 10.1016/j.cmrp.2020.05.005
[2233] Amirfakhryan H, Safari F (2020) Outbreak of SARS-CoV2: Pathogenesis of infection and cardiovascular involvement. Hellenic journal of cardiology : HJC = Hellenike kardiologike epitheorese. 10.1016/j.hjc.2020.05.007
[2234] Bellido V, Pérez A (2020) Consequences of COVID-19 on people with diabetes. Endocrinologia, diabetes y nutricion 67(6): 355-356. 10.1016/j.endinu.2020.04.001
[2235] Bergman M, Jagannathan R, Narayan KMV (2020) Nexus of COVID-19 and diabetes pandemics: Global public health lessons. Diabetes Res Clin Pract 164: 108215. 10.1016/j.diabres.2020.108215
[2236] Bornstein SR, Dalan R, Hopkins D, Mingrone G, Boehm BO (2020) Endocrine and metabolic link to coronavirus infection. Nature reviews Endocrinology 16(6): 297-298. 10.1038/s41574-020-0353-9
[2237] Ferrari F (2020) COVID-19: Updated Data and its Relation to the Cardiovascular System. Arquivos brasileiros de cardiologia 114(5): 823-826. 10.36660/abc.20200215
[2238] Filardi T, Morano S (2020) COVID-19: is there a link between the course of infection and pharmacological agents in diabetes? J Endocrinol Invest: 1-8. 10.1007/s40618-020-01318-1

[2239] Finucane FM, Davenport C (2020) Coronavirus and Obesity: Could Insulin Resistance Mediate the Severity of Covid-19 Infection? Frontiers in Public Health 8. 10.3389/fpubh.2020.00184
[2240] Gupta R, Ghosh A, Singh AK, Misra A (2020) Clinical considerations for patients with diabetes in times of COVID-19 epidemic. Diabetes Metab Syndr 14(3): 211-212
[2241] Mirabelli M, Chiefari E, Puccio L, Foti DP, Brunetti A (2020) Potential Benefits and Harms of Novel Antidiabetic Drugs During COVID-19 Crisis. Int j environ res public health (Online) 17(10)
[2242] Pititto BdA, Ferreira SRG (2020) Diabetes and covid-19: more than the sum of two morbidities. Rev Saude Publica 54: 54-54
[2243] Roncon L, Zuin M, Rigatelli G, Zuliani G (2020) Diabetic patients with COVID-19 infection are at higher risk of ICU admission and poor short-term outcome. J Clin Virol 127: 104354-104354
[2244] Scheen AJ, Marre M, Thivolet C (2020) Prognostic factors in patients with diabetes hospitalized for COVID-19: Findings from the CORONADO study and other recent reports. Diabetes & metabolism. 10.1016/j.diabet.2020.05.008
[2245] Schofield J, Leelarathna L, Thabit H (2020) COVID-19: Impact of and on Diabetes.
[2246] Torres-Tamayo M, Caracas-Portillo NA, Peña-Aparicio B, Juárez-Rojas JG, Medina-Urrutia AX, Martínez-Alvarado MDR (2020) Coronavirus infection in patients with diabetes. Arch Cardiol Mex 90(Supl): 67-76. 10.24875/acm.m20000068
[2247] Abdelhafiz AH, Emmerton D, Sinclair AJ (2021) Diabetes in COVID-19 pandemic-prevalence, patient characteristics and adverse outcomes. Int J Clin Pract: e14112. 10.1111/ijcp.14112
[2248] Abdi A, Jalilian M, Sarbarzeh PA, Vlaisavljevic Z (2020) Diabetes and COVID-19: a systematic review on the current evidences. (Special issue on diabetes and COVID-19: the IDF perspective.). Diabetes Research and Clinical Practice 16667
[2249] Apicella M, Campopiano MC, Mantuano M, Mazoni L, Coppelli A, Del Prato S (2020) COVID-19 in people with diabetes: understanding the reasons for worse outcomes (vol 8, pg 782, 2020). Lancet Diabetes & Endocrinology 8(10): E5-E5
[2250] Arumugam VA, Thangavelu S, Ravindran P, et al. (2020) COVID-19 and the world with co-morbidities of heart disease, hypertension and diabetes. J Pure Appl Microbiol 14(3): 1623-1638. 10.22207/JPAM.14.3.01
[2251] Bae S, Kim SR, Kim MN, Shim WJ, Park SM (2021) Impact of cardiovascular disease and risk factors on fatal outcomes in patients with COVID-19 according to age: a systematic review and meta-analysis. Heart 107(5): 373-380. 10.1136/heartjnl-2020-317901
[2252] Barek MA, Aziz MA, Islam MS (2020) Impact of age, sex, comorbidities and clinical symptoms on the severity of COVID-19 cases: A meta-analysis with 55 studies and 10014 cases. Heliyon 6(12): e05684. 10.1016/j.heliyon.2020.e05684
[2253] Barone MTU, Ngongo B, Harnik SB, et al. (2021) COVID-19 associated with diabetes and other noncommunicable diseases led to a global health crisis. Diabetes Research and Clinical Practice 171. 10.1016/j.diabres.2020.108587
[2254] Chang DJ, Moin T (2020) Coronavirus disease 2019 and type 1 diabetes mellitus. Curr opin endocrinol diabetes obes
[2255] de Almeida-Pititto B, Dualib PM, Zajdenverg L, et al. (2020) Severity and mortality of COVID 19 in patients with diabetes, hypertension and cardiovascular disease: a meta-analysis. Diabetology & Metabolic Syndrome 12(1). 10.1186/s13098-020-00586-4
[2256] Dhar SK, Sachdeva K, Das M (2021) Inflammation and hypercoagulopathy are predominant risk factors of severity in COVID-19 patients with diabetes mellitus: Summary of a meta-analysis. Diabetes Metab Syndr 15(2): 639-641. 10.1016/j.dsx.2021.02.021
[2257] Du P, Li D, Wang A, Shen S, Ma Z, Li X (2021) A Systematic Review and Meta-Analysis of Risk Factors Associated with Severity and Death in COVID-19 Patients. Can J Infect Dis Med Microbiol 2021: 6660930. 10.1155/2021/6660930
[2258] Fleming N, Sacks LJ, Pham CT, Neoh SL, Ekinci EI (2021) An overview of COVID-19 in people with diabetes: Pathophysiology and considerations in the inpatient setting. Diabet Med 38(3): e14509. 10.1111/dme.14509
[2259] Fox C, Kilvert A (2021) Triple jeopardy: old age, frailty and diabetes in COVID-19. Pract Diabetes 38(1): 25-30. 10.1002/pdi.2317
[2260] Gregory JM, Moore DJ (2021) The Dual Burden of Type 1 Diabetes and COVID-19. Ann Intern Med. 10.7326/m21-0367
[2261] Halushko O, Loskutov O, Kuchynska I, Synytsyn M, Boliuk M (2020) THE MAIN CAUSES OF THE COMPLICATED COURSE OF COVID-19 IN PATIENTS WITH DIABETES MELLITUS AND TREATMENT (REVIEW). Georgian Med News(307): 114-120
[2262] Hariyanto TI, Kurniawan A (2020) Metformin use is associated with reduced mortality rate from coronavirus disease 2019 (COVID-19) infection. Obes Med 19: 100290. 10.1016/j.obmed.2020.100290

[2263] Hariyanto TI, Kurniawan A (2020) Statin therapy did not improve the in-hospital outcome of coronavirus disease 2019 (COVID-19) infection. Diabetes Metab Syndr 14(6): 1613-1615. 10.1016/j.dsx.2020.08.023
[2264] Hariyanto TI, Kurniawan A (2021) Dipeptidyl peptidase 4 (DPP4) inhibitor and outcome from coronavirus disease 2019 (COVID-19) in diabetic patients: a systematic review, meta-analysis, and meta-regression. J Diabetes Metab Disord: 1-8. 10.1007/s40200-021-00777-4
[2265] Hussain A, Bhowmik B, Moreira NCD (2020) COVID-19 and diabetes: Knowledge in progress. Diabetes Research and Clinical Practice 162. 10.1016/j.diabres.2020.108142
[2266] Iqbal MZ, Zohra B (2020) COVID-19 and Diabetes Mellitus: Prevalence and Precautions. 26
[2267] Khaled KA (2020) Diabetes mellitus is an important predictor for hospitalization and mortality from the COVID-19 infection: A substantial interface between two outbreaks. Journal of Endocrinology and Metabolism 10(10). 10.14740/JEM.V10I3-4.665
[2268] Kow CS, Hasan SS (2021) Insulin Treatment May Not Be Associated with Increased Mortality in Patients with COVID-19 and Concurrent Diabetes. Endocr Res 46(2): 51-52. 10.1080/07435800.2021.1892748
[2269] Lagunas-Rangel FA, Chávez-Valencia V (2020) Laboratory findings that predict a poor prognosis in COVID-19 patients with diabetes: A meta-analysis. Endocrinol Diabetes Nutr. 10.1016/j.endinu.2020.07.002
[2270] Lazarus G, Suhardi IP, Wiyarta E, Rasyidah RA, Barliana JD (2021) Is there a need to reconsider the use of metformin in COVID-19 patients with type 2 diabetes mellitus? Int J Diabetes Dev Ctries: 1-6. 10.1007/s13410-021-00924-w
[2271] Lee T, Cau A, Cheng MP, et al. (2021) ANGIOTENSIN RECEPTOR BLOCKERS AND ANGIOTENSIN CONVERTING ENZYME INHIBITORS IN COVID-19 -META-ANALYSIS/META-REGRESSION ADJUSTED FOR CONFOUNDING FACTORS. CJC Open. 10.1016/j.cjco.2021.03.001
[2272] Li X, Zhong X, Wang Y, Zeng X, Luo T, Liu Q (2021) Clinical determinants of the severity of COVID-19: A systematic review and meta-analysis. PLoS One 16(5): e0250602. 10.1371/journal.pone.0250602
[2273] Lima-Martínez MM, Carrera Boada C, Madera-Silva MD, Marín W, Contreras M (2020) COVID-19 and diabetes: A bidirectional relationship. Clin investig arterioscler (Ed impr)
[2274] Lui DTW, Tan KCB (2021) Is metformin a miracle or a menace in COVID-19 patients with type 2 diabetes? J Diabetes Investig 12(4): 479-481. 10.1111/jdi.13484
[2275] Lukito AA, Pranata R, Henrina J, Lim MA, Lawrensia S, Suastika K (2020) The Effect of Metformin Consumption on Mortality in Hospitalized COVID-19 patients: a systematic review and meta-analysis. Diabetes Metab Syndr 14(6): 2177-2183. 10.1016/j.dsx.2020.11.006
[2276] Mahrooz A, Muscogiuri G, Buzzetti R, Maddaloni E (2021) The complex combination of COVID-19 and diabetes: pleiotropic changes in glucose metabolism. Endocrine: 1-9. 10.1007/s12020-021-02729-7
[2277] Matsushita K, Ding N, Kou MH, et al. (2020) The Relationship of COVID-19 Severity with Cardiovascular Disease and Its Traditional Risk Factors: A Systematic Review and Meta-Analysis. Global Heart 15(1). 10.5334/gh.814
[2278] Meng M, Zhao QW, Kumar R, Bai C, Deng YL, Wan B (2020) Impact of cardiovascular and metabolic diseases on the severity of COVID-19: a systematic review and meta-analysis. Aging-Us 12(22): 23409-23421
[2279] Mirjalili H, Dastgheib SA, Shaker SH, et al. (2021) Proportion and mortality of Iranian diabetes mellitus, chronic kidney disease, hypertension and cardiovascular disease patients with COVID-19: a meta-analysis. J Diabetes Metab Disord: 1-13. 10.1007/s40200-021-00768-5
[2280] Mishra D, Dey CS (2021) Type-2 diabetes, a co-morbidity in Covid-19: does insulin signaling matter? Biochem Soc Trans 49(2): 987-995. 10.1042/bst20201062
[2281] Mishra P, Parveen R, Bajpai R, Samim M, Agarwal NB (2021) Impact of cardiovascular diseases on severity of COVID-19 patients: A systematic review. Ann Acad Med Singap 50(1): 52-60. 10.47102/annals-acadmedsg.2020367
[2282] Mishra PK, Tandon R, Byrareddy SN (2020) Diabetes and COVID-19 risk: an miRNA perspective. Am J Physiol Heart Circ Physiol 319(3): H604-H609
[2283] Momenzadeh M (2020) Prevalence of diabetes, hypertension and cardiovascular disease in patients with covid-19: A systematic review and meta-analysis. Eurasian Journal of Biosciences 14(1)
[2284] Mostaured K, Nuruzzaman K, Md. Golam M, Juwel R, Saiful I, Iqbal K (2020) Effects of underlying morbidities on the occurrence of deaths in COVID-19 patients: A systematic review and meta-analysis. medRxiv. 10.1101/2020.05.08.20095968
[2285] Zhang Y, Wu L, Yang J, Zhou C, Liu Y (2020) A Nomogram-Based Prediction for Severe Pneumonia in Patients with Coronavirus Disease 2019 (COVID-19). Infect Drug Resist 13: 3575-3582. 10.2147/idr.S261725
[2286] Ng SM (2020) COVID-19 and children with diabetes: emerging knowledge. Pract Diabetes 37(4): 147-148a. 10.1002/pdi.2289

[2287] Pal R, Banerjee M, Mukherjee S, Bhogal RS, Kaur A, Bhadada SK (2021) Dipeptidyl peptidase-4 inhibitor use and mortality in COVID-19 patients with diabetes mellitus: an updated systematic review and meta-analysis. Ther Adv Endocrinol Metab 12: 2042018821996482. 10.1177/2042018821996482
[2288] Palaiodimos L, Chamorro-Pareja N, Karamanis D, et al. Diabetes is associated with increased risk for in-hospital mortality in patients with COVID-19: a systematic review and meta-analysis comprising 18,506 patients. Hormones-International Journal of Endocrinology and Metabolism. 10.1007/s42000-020-00246-2
[2289] Papadopoulos VP, Koutroulos MV, Zikoudi DG, et al. (2021) Diabetes-related acute metabolic emergencies in COVID-19 patients: a systematic review and meta-analysis. Diabetol Int: 1-15. 10.1007/s13340-021-00502-9
[2290] Pfutzner A, Frankenberger R (2020) Patients with Diabetes are at a higher Risk of severe COVID-19 Processes - What Protective Measures are possible? Diabetes Stoffwechsel Und Herz 29(6): 367-371
[2291] Qiu PS, Zhou YJ, Wang F, et al. (2020) Clinical characteristics, laboratory outcome characteristics, comorbidities, and complications of related COVID-19 deceased: a systematic review and meta-analysis. Aging Clinical and Experimental Research 32(9): 1869-1878. 10.1007/s40520-020-01664-3
[2292] Qu H, Zheng Y, Wang YR, et al. (2021) The potential effects of clinical antidiabetic agents on SARS-CoV-2. Journal of Diabetes 13(3): 243-252. 10.1111/1753-0407.13135
[2293] Rahman A, Sathi NJ (2020) Risk factors of the severity of COVID-19: A meta-analysis. Int J Clin Pract: e13916. 10.1111/ijcp.13916
[2294] Rahmani-Kukia N, Abbasi A (2021) Physiological and Immunological Causes of the Susceptibility of Chronic Inflammatory Patients to COVID-19 Infection: Focus on Diabetes. Front Endocrinol (Lausanne) 12: 576412. 10.3389/fendo.2021.576412
[2295] Rimesh P, Mainak B, Ashok K, Sanjay Kumar B (2020) Glycemic efficacy and safety of hydroxychloroquine in type 2 diabetes mellitus: A systematic review and meta.analysis of relevance amid the COVID-19 pandemic. International Journal of Medical Science and Public Health 5(4). 10.4103/JNCD.JNCD_69_20
[2296] Rizwana, Parveen, Nouroz, et al. (2020) Association of diabetes and hypertension with disease severity in COVID-19 patients: a systematic literature review and exploratory meta-analysis. (Special issue on diabetes and COVID-19: the IDF perspective.). Diabetes Research and Clinical Practice 16623
[2297] Romil S, Sawai Singh R, Hira K, et al. (2021) Mortality and Severity in COVID-19 Patients on ACEIs & ARBs - A Meta-Regression Analysis. In:
[2298] Saha S, Al-Rifai RH, Saha S (2021) Diabetes prevalence and mortality in COVID-19 patients: a systematic review, meta-analysis, and meta-regression. J Diabetes Metab Disord: 1-12. 10.1007/s40200-021-00779-2
[2299] Sathish T, Kapoor N, Cao YT, Tapp RJ, Zimmet P (2021) Proportion of newly diagnosed diabetes in COVID-19 patients: A systematic review and meta-analysis. Diabetes Obesity & Metabolism 23(3): 870-874. 10.1111/dom.14269
[2300] Saud A, Amani A, Hala M, et al. (2020) Saudi Scientific Diabetes Society Position Statement: Management of Diabetes Mellitus in the Pandemic of COVID-19. International Journal of Physical Medicine and Rehabilitation 11(05). 10.4236/IJCM.2020.115020
[2301] Scheen AJ (2020) Statins and clinical outcomes with COVID-19: Meta-analyses of observational studies. Diabetes Metab 47(6): 101220. 10.1016/j.diabet.2020.101220
[2302] Shang LX, Shao MJ, Guo QL, et al. (2020) Diabetes Mellitus is Associated with Severe Infection and Mortality in Patients with COVID-19: A Systematic Review and Meta-analysis. Archives of Medical Research 51(7): 700-709. 10.1016/j.arcmed.2020.07.005
[2303] Silverio A, Di Maio M, Citro R, et al. (2021) Cardiovascular risk factors and mortality in hospitalized patients with COVID-19: systematic review and meta-analysis of 45 studies and 18,300 patients. BMC Cardiovasc Disord 21(1): 23. 10.1186/s12872-020-01816-3
[2304] Sinclair JE, Zhu Y, Xu G, et al. (2021) A meta-analysis on the role of pre-existing chronic disease in the cardiac complications of SARS-CoV-2 infection. iScience 24(4): 102264. 10.1016/j.isci.2021.102264
[2305] Singh AK, Khunti K (2020) Assessment of risk, severity, mortality, glycemic control and antidiabetic agents in patients with diabetes and COVID-19: A narrative review. Diabetes Research and Clinical Practice 165. 10.1016/j.diabres.2020.108266
[2306] Singh AK, Singh R, Saboo B, Misra A (2021) Non-insulin anti-diabetic agents in patients with type 2 diabetes and COVID-19: A Critical Appraisal of Literature. Diabetes Metab Syndr 15(1): 159-167. 10.1016/j.dsx.2020.12.026

[2307] Strickland OL, Powell-Young Y, Reyes-Miranda C, Alzaghari O, Giger JN (2020) African-Americans Have a Higher Propensity for Death from COVID-19: Rationale and Causation. J Natl Black Nurses Assoc 31(1): 1-12
[2308] Sun B, Huang S, Zhou J (2020) Perspectives of Antidiabetic Drugs in Diabetes With Coronavirus Infections. Front Pharmacol 11: 592439. 10.3389/fphar.2020.592439
[2309] Sur J, Sharma J, Sharma D (2020) Diabetes Might Augment the Severity of COVID-19: A Current Prospects. Front Cardiovasc Med 7: 613255. 10.3389/fcvm.2020.613255
[2310] Tian WJ, Jiang WL, Yao J, et al. (2020) Predictors of mortality in hospitalized COVID-19 patients: A systematic review and meta-analysis. Journal of Medical Virology 92(10): 1875-1883. 10.1002/jmv.26050
[2311] Tiantia H, Shaodi M, Chenyu S, et al. (2021) The Association Between Hypoglycemic Agents and Clinical Outcomes of COVID-19 in Patients with Diabetes: A Systematic Review and Meta-Analysis. medRxiv. 10.1101/2021.01.26.21250506
[2312] Utin Ilma Agni Ka, Setyo Sri R, Didik Gunawan T (2020) Meta-Analysis: The Effect of Diabetes Mellitus Comorbidity on the Risk of Death in Covid-19 Patients. 5(4). 10.26911/THEIJMED.2020.05.04.12
[2313] Valencia I, Peiro C, Lorenzo O, Sanchez-Ferrer CF, Eckel J, Romacho T (2020) DPP4 and ACE2 in Diabetes and COVID-19: Therapeutic Targets for Cardiovascular Complications? Frontiers in Pharmacology 11. 10.3389/fphar.2020.01161
[2314] Vallianou NG, Evangelopoulos A, Kounatidis D, et al. (2020) Diabetes mellitus and SARS-CoV-2 infection: pathophysiologic mechanisms and implications in management. Curr Diabetes Rev. 10.2174/1573399817666210101110253
[2315] Varikasuvu SR, Varshney S, Dutt N (2020) Markers of coagulation dysfunction and inflammation in diabetic and non-diabetic COVID-19. J Thromb Thrombolysis: 1-6. 10.1007/s11239-020-02270-w
[2316] Vasileios Periklis P, Marios-Vasileios K, Dimitra-Georgia Z, et al. (2021) Acute Metabolic Emergencies in Diabetes and COVID-19: a systematic review and meta-analysis of case reports. medRxiv. 10.1101/2021.01.10.21249550
[2317] Vignesh C, Nyan Lynn T, Waqas ZH, et al. (2020) Predictors of Severity and Mortality Among Patients with Coronavirus Disease 2019: A Systematic Review and Meta-Analysis.
[2318] Viswanathan V, Puvvula A, Jamthikar AD, et al. (2021) Bidirectional link between diabetes mellitus and coronavirus disease 2019 leading to cardiovascular disease: A narrative review. World J Diabetes 12(3): 215-237. 10.4239/wjd.v12.i3.215
[2319] Wang X, Wang SJ, Sun LG, Qin GJ (2020) Prevalence of diabetes mellitus in 2019 novel coronavirus: A meta -analysis. Diabetes Research and Clinical Practice 164
[2320] Wang YX, Chen BX, Li Y, et al. (2021) The use of renin-angiotensin-aldosterone system (RAAS) inhibitors is associated with a lower risk of mortality in hypertensive COVID-19 patients: A systematic review and meta-analysis. Journal of Medical Virology 93(3): 1370-1377. 10.1002/jmv.26625
[2321] Wee AKH (2020) COVID-19's toll on the elderly and those with diabetes mellitus - Is vitamin B12 deficiency an accomplice? Med Hypotheses: 110374-110374
[2322] Wu CC, Lee AJ, Su CH, Huang CY, Islam MM, Weng YC (2021) Statin Use Is Associated with a Decreased Risk of Mortality among Patients with COVID-19. J Clin Med 10(7). 10.3390/jcm10071450
[2323] Wu Y, Li H, Zhang Z, et al. (2021) Risk factors for mortality of coronavirus disease 2019 (COVID-19) patients during the early outbreak of COVID-19: a systematic review and meta-analysis. Ann Palliat Med. 10.21037/apm-20-2557
[2324] Xie YL, Wang JL, Yang HQ, et al. (2021) The risk factors for severe patients with COVID-19 in China: A systematic review and meta-analysis. European Journal of Inflammation 19. 10.1177/20587392211000890
[2325] Yahya Mahamat S, Thibault F, Mathieu Edouard R, et al. (2021) Diabetes, Hypertension, Body Mass Index, Smoking and COVID-19-Related Mortality: A Systematic Review and Meta-Analysis of Observational Studies. SSRN. 10.2139/ssrn.3773509
[2326] Zaki N, Mohamed E, Ibrahim S, Khan G (2020) The influence of comorbidity on the severity of COVID-19 disease: A systematic review and analysis. ResearchSquare. 10.21203/rs.3.rs-37127/v2
[2327] Zangiabadian M, Nejadghaderi SA, Zahmatkesh MM, Hajikhani B, Mirsaeidi M, Nasiri MJ (2021) The Efficacy and Potential Mechanisms of Metformin in the Treatment of COVID-19 in the Diabetics: A Systematic Review. Front Endocrinol (Lausanne) 12: 645194. 10.3389/fendo.2021.645194
[2328] Zheng ZH, Peng F, Xu BY, et al. (2020) Risk factors of critical & mortal COVID-19 cases: A systematic literature review and meta-analysis. Journal of Infection 81(2): E16-E25. 10.1016/j.jinf.2020.04.021

[2329] Zhou X, Cheng Z, Shu D, et al. (2020) Characteristics of mortal COVID-19 cases compared to the survivors. Aging (Albany NY) 12. 10.18632/aging.202216
[2330] Zhou Y, Yang Q, Chi JW, et al. (2020) Comorbidities and the risk of severe or fatal outcomes associated with coronavirus disease 2019: A systematic review and meta-analysis. International Journal of Infectious Diseases 99: 47-56. 10.1016/j.ijid.2020.07.029
[2331] Zuo, Hui, Hussain, A (2020) Comorbid diabetes and the risk of disease severity or death among 8807 COVID-19 patients in China: a meta-analysis. (Special issue on diabetes and COVID-19: the IDF perspective.). Diabetes Research and Clinical Practice 16640
[2332] Jianping W (2020) Perspectives on COVID-19 complicated with diabetes mellitus. 12(4). 10.3760/CMA.J.CN115791-20200304-00127
[2333] Paquot N, Radermecker RP (2020) [Covid-19 and diabetes]. Revue medicale de Liege 75(S1): 138-145
[2334] Plasencia Urizarri Thais M, Aguilera Rodríguez R, Almaguer Mederos L (2020) HIPERTENSIÓN, DIABETES Y ENFERMEDAD RENAL CRÓNICA COMO FACTORES DE RIESGO PARA COVID-19 GRAVE.
[2335] Scheen AJ, Paquot N (2021) [Continuing or stopping metformin in patients with type 2 diabetes exposed to SARS-CoV-2 ?]. Rev Med Liege 76(3): 152-155
[2336] Alhumaid S, Al Mutair A, Al Alawi Z, et al. (2021) Diabetic ketoacidosis in patients with SARS-CoV-2: a systematic review and meta-analysis. Diabetol Metab Syndr 13(1): 120. 10.1186/s13098-021-00740-6
[2337] Debi H, Itu ZT, Amin MT, Hussain F, Hossain MS (2022) Association of serum C-reactive protein (CRP) and D-dimer concentration on the severity of COVID-19 cases with or without diabetes: a systematic review and meta-analysis. Expert Rev Endocrinol Metab 17(1): 83-93. 10.1080/17446651.2022.2002146
[2338] Ganesh A, Randall MD (2022) Does metformin affect outcomes in COVID-19 patients with new or pre-existing diabetes mellitus? A systematic review and meta-analysis. Br J Clin Pharmacol. 10.1111/bcp.15258
[2339] Han T, Ma S, Sun C, et al. (2021) The Association Between Anti-diabetic Agents and Clinical Outcomes of COVID-19 in Patients with Diabetes: A Systematic Review and Meta-Analysis. Arch Med Res. 10.1016/j.arcmed.2021.08.002
[2340] Hariyanto TI, Intan D, Hananto JE, Putri C, Kurniawan A (2021) Pre-admission glucagon-like peptide-1 receptor agonist (GLP-1RA) and mortality from coronavirus disease 2019 (Covid-19): A systematic review, meta-analysis, and meta-regression. Diabetes Res Clin Pract 179: 109031. 10.1016/j.diabres.2021.109031
[2341] Hariyanto TI, Lugito NPH, Yanto TA, Siregar JI, Kurniawan A (2021) Insulin therapy and outcome from coronavirus disease 2019 (COVID-19): A Systematic Review, Meta-Analysis, and Meta-Regression. Endocr Metab Immune Disord Drug Targets. 10.2174/1871530321666210709164925
[2342] Kan C, Zhang Y, Han F, et al. (2021) Mortality Risk of Antidiabetic Agents for Type 2 Diabetes With COVID-19: A Systematic Review and Meta-Analysis. Front Endocrinol (Lausanne) 12: 708494. 10.3389/fendo.2021.708494
[2343] Kurniawati O, Prasetya H, Murti B (2021) Meta-analysis the effects of obesity and type 2 diabetes mellitus on COVID-19 mortality. Journal of Epidemiology and Public Health 6(2): 177-191. 10.26911/jepublichealth.2021.06.02.05
[2344] Li Y, Yang X, Yan P, Sun T, Zeng Z, Li S (2021) Metformin in Patients With COVID-19: A Systematic Review and Meta-Analysis. Front Med (Lausanne) 8: 704666. 10.3389/fmed.2021.704666
[2345] Maddaloni E, D'Onofrio L, Siena A, et al. (2021) Impact of cardiovascular disease on clinical outcomes in hospitalized patients with Covid-19: a systematic review and meta-analysis. Intern Emerg Med 16(7): 1975-1985. 10.1007/s11739-021-02804-x
[2346] Nawangsih EN, Kusmala YY, Rakhmat, II, et al. (2021) Colchicine and mortality in patients with coronavirus disease 2019 (COVID-19) pneumonia: A systematic review, meta-analysis, and meta-regression. Int Immunopharmacol 96: 107723. 10.1016/j.intimp.2021.107723
[2347] Patoulias D, Doumas M (2021) Dipeptidyl Peptidase-4 Inhibitors and COVID-19-Related Deaths among Patients with Type 2 Diabetes Mellitus: A Meta-Analysis of Observational Studies. Endocrinol Metab (Seoul) 36(4): 904-908. 10.3803/EnM.2021.1048
[2348] Poly TN, Islam MM, Li YJ, Lin MC, Hsu MH, Wang YC (2021) Metformin Use Is Associated with Decreased Mortality in COVID-19 Patients with Diabetes: Evidence from Retrospective Studies and Biological Mechanism. J Clin Med 10(16). 10.3390/jcm10163507
[2349] Prattichizzo F, de Candia P, Nicolucci A, Ceriello A (2021) Impact of pre-infection HbA1c levels on COVID-19 prognosis:systematic review and meta-analysis. Diabetes/metabolism research and reviews: e3476. 10.1002/dmrr.3476
[2350] Rico-Martín S, Calderón-García JF, Basilio-Fernández B, Clavijo-Chamorro MZ, Sánchez Muñoz-Torrero JF (2021) Metabolic Syndrome and Its Components in Patients with COVID-19:

Severe Acute Respiratory Syndrome (SARS) and Mortality. A Systematic Review and Meta-Analysis. J Cardiovasc Dev Dis 8(12). 10.3390/jcdd8120162
[2351] Schlesinger SNMLAPKKOHCRM (2020) Risk Phenotypes of Diabetes and Association with COVID-19 Severity and Death – A Living Systematic Review and Meta-Analysis (preprint). In:
[2352] Yang W, Sun X, Zhang J, Zhang K (2021) The effect of metformin on mortality and severity in COVID-19 patients with diabetes mellitus. Diabetes Res Clin Pract 178: 108977. 10.1016/j.diabres.2021.108977
[2353] Yang Y, Cai Z, Zhang J (2021) Insulin Treatment May Increase Adverse Outcomes in Patients With COVID-19 and Diabetes: A Systematic Review and Meta-Analysis. Front Endocrinol (Lausanne) 12: 696087. 10.3389/fendo.2021.696087
[2354] Yang Y, Cai Z, Zhang J (2021) DPP-4 inhibitors may improve the mortality of coronavirus disease 2019: A meta-analysis. PLoS One 16(5): e0251916. 10.1371/journal.pone.0251916
[2355] Zein A, Raffaello WM (2022) Dipeptidyl peptidase-4 (DPP-IV) inhibitor was associated with mortality reduction in COVID-19 - A systematic review and meta-analysis. Prim Care Diabetes 16(1): 162-167. 10.1016/j.pcd.2021.12.008
[2356] Zhao LM, Chen XH, Qiu M (2021) Commentary: Mortality Risk of Antidiabetic Agents for Type 2 Diabetes With COVID-19: A Systematic Review and Meta-Analysis. Front Endocrinol (Lausanne) 12: 825100. 10.3389/fendo.2021.825100
[2357] Zhu Z, Mao Y, Chen G (2021) Predictive value of HbA1c for in-hospital adverse prognosis in COVID-19: A systematic review and meta-analysis. Prim Care Diabetes 15(6): 910-917. 10.1016/j.pcd.2021.07.013
[2358] Han TMSSCZHQGCYCCCEL, Ayaz Ahmed MKKYMRCMGZYHYYZQ (2021) The Association between Hypoglycemic Agents and Clinical Outcomes of COVID-19 in Patients with Diabetes: A Systematic Review and Meta-Analysis (preprint). In:
[2359] Wang W, Sun Y, Wang S, Sun Y (2022) The Relationship Between Insulin Use And Increased Mortality In Patients With COVID-19 And Diabetes: A Meta-Analysis. Endocr Res 47(1): 32-38. 10.1080/07435800.2021.1967376
[2360] Wiyarta E, Wisnu W (2021) Does Insulin Use Worsen the Prognosis of COVID-19 Patients with Type 2 Diabetes Mellitus? A Current Update. Curr Diabetes Rev. 10.2174/1573399818666211117110113
[2361] Ahmed HS, Ahmed HS (2021) Diabetes and covid-19 pandemic: A potential mechanisms: A review. Indian Journal of Forensic Medicine and Toxicology 15(1): 2224-2231. 10.37506/ijfmt.v15i1.13734
[2362] Akbar MR, Pranata R, Wibowo A, Lim MA, Sihite TA, Martha JW (2021) The association between serum prealbumin and poor outcome in COVID-19 - Systematic review and meta-analysis. Eur Rev Med Pharmacol Sci 25(10): 3879-3885. 10.26355/eurrev_202105_25955
[2363] Alkodaymi MS, Omrani OA, Fawzy NA, et al. (2022) Prevalence of post-acute COVID-19 syndrome symptoms at different follow-up periods: A systematic review and meta-analysis. Clin Microbiol Infect. 10.1016/j.cmi.2022.01.014
[2364] Alsagaff MY, Mulia EPB, Maghfirah I, et al. (2021) Association of calcium channel blocker use with clinical outcome of COVID-19: A meta-analysis. Diabetes Metab Syndr 15(5): 102210. 10.1016/j.dsx.2021.102210
[2365] Alves VP, Casemiro FG, Araujo BG, et al. (2021) Factors Associated with Mortality among Elderly People in the COVID-19 Pandemic (SARS-CoV-2): A Systematic Review and Meta-Analysis. Int J Environ Res Public Health 18(15). 10.3390/ijerph18158008
[2366] Amin MT, Fatema K, Arefin S, Hussain F, Bhowmik DR, Hossain MS (2021) Obesity, a major risk factor for immunity and severe outcomes of COVID-19. Biosci Rep 41(8). 10.1042/bsr20210979
[2367] Anne M, Brendan L (2020) Evidence summary: In patients with COVID-19, what characteristics will suggest development of severe disease?
[2368] Audrey J, Wisnu W, Tahapary DL (2022) Association Between Statin Use and Poor Outcomes in COVID-19 Patients with Diabetes Mellitus: A Systematic Review. Curr Diabetes Rev. 10.2174/1573399818666220209095056
[2369] Basra R, Whyte M, Karalliedde J, Vas P (2022) What is the impact of microvascular complications of diabetes on severe COVID-19? Microvasc Res 140: 104310. 10.1016/j.mvr.2021.104310
[2370] Basu A, Agwu JC, Barlow N, Lee B (2021) Hypertension is the major predictor of poor outcomes among inpatients with COVID-19 infection in the UK: a retrospective cohort study. BMJ Open 11(6): e047561. 10.1136/bmjopen-2020-047561
[2371] Bevacqua RJ, Perrone SV (2020) COVID-19: relación entre enzima convertidora de angiotensina 2, sistema cardiovascular y respuesta inmune del huésped. Insuficiencia cardíaca 15(2): 34-51

[2372] Bibi N, Wara B, Morrissey H, Ball P (2020) Emerging evidence on the association between COVID-19 and Type 2 Diabetes. Romanian Journal of Diabetes, Nutrition and Metabolic Diseases 27(4): 410-418. 10.46389/rjd-2020-1059
[2373] Borsche L, Glauner B, von Mendel J (2021) COVID-19 Mortality Risk Correlates Inversely with Vitamin D3 Status, and a Mortality Rate Close to Zero Could Theoretically Be Achieved at 50 ng/mL 25(OH)D3: Results of a Systematic Review and Meta-Analysis. Nutrients 13(10). 10.3390/nu13103596
[2374] Bradley SA, Banach M, Alvarado N, Smokovski I, Bhaskar SMM (2022) Prevalence and impact of diabetes in hospitalized COVID-19 patients: A systematic review and meta-analysis. J Diabetes 14(2): 144-157. 10.1111/1753-0407.13243
[2375] Buscemi S, Corleo D, Randazzo C (2021) Risk Factors for COVID-19: Diabetes, Hypertension, and Obesity. Adv Exp Med Biol 1353: 115-129. 10.1007/978-3-030-85113-2_7
[2376] Cai X, Wu G, Zhang J, Yang L (2021) Risk Factors for Acute Kidney Injury in Adult Patients With COVID-19: A Systematic Review and Meta-Analysis. Front Med (Lausanne) 8: 719472. 10.3389/fmed.2021.719472
[2377] Cai Z, Yang Y, Zhang J (2021) Obesity is associated with severe disease and mortality in patients with coronavirus disease 2019 (COVID-19): a meta-analysis. BMC Public Health 21(1): 1505. 10.1186/s12889-021-11546-6
[2378] Cangelosi G, Grappasonni I, Pantanetti P, et al. (2022) Nurse Case Manager Lifestyle Medicine (NCMLM) in the Type Two Diabetes patient concerning post COVID-19 Pandemic management: Integrated-Scoping literature review. Ann Ig. 10.7416/ai.2022.2500
[2379] Chambergo-Michilot D, Barros-Sevillano S, Rivera-Torrejón O, De la Cruz-Ku GA, Custodio N (2021) Factors associated with COVID-19 in people with Parkinson's disease: a systematic review and meta-analysis. Eur J Neurol 28(10): 3467-3477. 10.1111/ene.14912
[2380] Chong WH, Saha BK, Neu KP (2022) Comparing the clinical characteristics and outcomes of COVID-19-associate pulmonary aspergillosis (CAPA): a systematic review and meta-analysis. Infection 50(1): 43-56. 10.1007/s15010-021-01701-x
[2381] Cordero AG-GCSB-GVFLR-MMEDCJM, Zuazola PNJBJJ, Ramón González-Juanatey J (2020) Mortality associated with cardiovascular disease in patients with COVID-19. REC: CardioClinics. 10.1016/j.rccl.2020.10.005
[2382] Dandona P, Ghanim H (2021) Diabetes, Obesity, COVID-19, Insulin, and Other Antidiabetes Drugs. Diabetes Care 44(9): 1929-1933. 10.2337/dci21-0003
[2383] Faradji-Hazan RN, Garnica-Cuellar JC, HernÁNdez-Arizpe LM, et al. (2020) Recomendaciones de la Sociedad Mexicana de Nutrición y Endocrinología SMNE ante el COVID-19 para el tratamiento de las personas que viven con DM2. Revista Mexicana de Endocrinología, Metabolismo y Nutrición 7(3): 122-131. 10.24875/RME.20000044
[2384] Figliozzi S, Masci PG, Ahmadi N, et al. (2020) Predictors of adverse prognosis in COVID-19: A systematic review and meta-analysis. Eur J Clin Invest 50(10): e13362. 10.1111/eci.13362
[2385] Figueroa-Pizano MD, Campa-Mada AC, Carvajal-Millan E, Martinez-Robinson KG, Chu AR (2021) The underlying mechanisms for severe COVID-19 progression in people with diabetes mellitus: a critical review. AIMS Public Health 8(4): 720-742. 10.3934/publichealth.2021057
[2386] Gangadharan C, Ahluwalia R, Sigamani A (2021) Diabetes and COVID-19: Role of insulin resistance as a risk factor for COVID-19 severity. World J Diabetes 12(9): 1550-1562. 10.4239/wjd.v12.i9.1550
[2387] George TP, Joy SS, Rafiullah M, Siddiqui K (2022) Cytokines in COVID-19 Patients with Diabetes: Systematic Review. Curr Diabetes Rev. 10.2174/1573399818666220118100743
[2388] Gholami B, Gholami S, Loghman AH, et al. (2021) Clinical and Laboratory Predictors of Severity, Criticality, and Mortality in COVID-19: A Multisystem Disease. Adv Exp Med Biol 1318: 369-402. 10.1007/978-3-030-63761-3_22
[2389] Gregg EW, Sophiea MK, Weldegiorgis M (2021) Diabetes and COVID-19: Population Impact 18 Months Into the Pandemic. Diabetes Care 44(9): 1916-1923. 10.2337/dci21-0001
[2390] Harrison SL, Buckley BJR, Rivera-Caravaca JM, Zhang J, Lip GYH (2021) Cardiovascular risk factors, cardiovascular disease, and COVID-19: an umbrella review of systematic reviews. Eur Heart J Qual Care Clin Outcomes 7(4): 330-339. 10.1093/ehjqcco/qcab029
[2391] Heidarpour M, Abhari AP, Sadeghpour N, Shafie D, Sarokhani D (2021) Prediabetes and COVID-19 severity, an underestimated risk factor: A systematic review and meta-analysis. Diabetes Metab Syndr 15(6): 102307. 10.1016/j.dsx.2021.102307
[2392] Hoang T, Tran Thi Anh T (2021) Comparison of Comorbidities in Relation to Critical Conditions among Coronavirus Disease 2019 Patients: A Network Meta-Analysis. Infect Chemother 53(1): 13-28. 10.3947/ic.2020.0136
[2393] Hollstein T (2020) Increased mortality risk: COVID-19 and diabetes - An unholy alliance. Deutsches Arzteblatt International 117(26): A1321-A1323+A1324

[2394] Ibrahim S, Lowe JR, Bramante CT, et al. (2021) Metformin and Covid-19: Focused Review of Mechanisms and Current Literature Suggesting Benefit. Front Endocrinol (Lausanne) 12: 587801. 10.3389/fendo.2021.587801
[2395] Jin S, Hu W (2021) Severity of COVID-19 and Treatment Strategy for Patient With Diabetes. Front Endocrinol (Lausanne) 12: 602735. 10.3389/fendo.2021.602735
[2396] Karla Romero S, David R, Gabriela P-H, Stefanie S, Albert N, Andreas S (2021) The isolated effect of age on the risk of COVID-19 severe outcomes: a systematic review with meta-analysis. In:
[2397] Katsiki N, Gómez-Huelgas R, Mikhailidis DP, Pérez-Martínez P (2021) Narrative review on clinical considerations for patients with diabetes and COVID-19: More questions than answers. Int J Clin Pract 75(11): e14833. 10.1111/ijcp.14833
[2398] Kaur U, Chakrabarti SS, Patel TK (2021) Renin-angiotensin-aldosterone system blockers and region-specific variations in COVID-19 outcomes: findings from a systematic review and meta-analysis. Ther Adv Drug Saf 12: 20420986211011345. 10.1177/20420986211011345
[2399] Khanam R (2021) Bidirectional Relationship between COVID-19 and Diabetes: Role of Renin-Angiotensin-Aldosterone System and Drugs Modulating It. J Pharm Bioallied Sci 13(2): 149-154. 10.4103/jpbs.JPBS_508_20
[2400] Khunti K, Del Prato S, Mathieu C, Kahn SE, Gabbay RA, Buse JB (2021) COVID-19, Hyperglycemia, and New-Onset Diabetes. Diabetes Care 44(12): 2645-2655. 10.2337/dc21-1318
[2401] Kifle ZD, Woldeyohanin AE, Demeke CA (2021) SARS-CoV-2 and diabetes: A potential therapeutic effect of dipeptidyl peptidase 4 inhibitors in diabetic patients diagnosed with COVID-19. Metabol Open 12: 100134. 10.1016/j.metop.2021.100134
[2402] Kifle ZD, Woldeyohanis AE, Demeke CA (2021) A review on protective roles and potential mechanisms of metformin in diabetic patients diagnosed with COVID-19. Metabol Open 12: 100137. 10.1016/j.metop.2021.100137
[2403] Klein F (2021) Severe COVID-19 courses in patients with diabetes quantified in Scotland. Diabetologie Und Stoffwechsel 16(2): 87-90. 10.1055/a-1332-6254
[2404] Koneru GSHH, Abd-elhamed NA, Elsedfy NMAH, et al. (2021) COVID-19 and Diabetes Mellitus: A Complex Interplay. Journal of Pure & Applied Microbiology 15(2): 512-523. 10.22207/JPAM.15.2.16
[2405] Kovalenko JS, Ivanova LA, Korol' IV, Bizheva TV (2021) Diabetes mellitus and covid-19. Features of the mutual influence of the two pandemics. Vestnik Sovremennoi Klinicheskoi Mediciny 14(4): 58-66. 10.20969/VSKM.2021.14(4).58-66
[2406] Kumar R, Rai AK, Phukan MM, et al. (2021) Accumulating Impact of Smoking and Co-morbidities on Severity and Mortality of COVID-19 Infection: A Systematic Review and Meta-analysis. Current Genomics 22(5): 339-352. 10.2174/1389202922666210921101728
[2407] Kushkestani M, Parvani M, Kazemzadeh Y (2021) SARS-COV-2 in type 2 diabetic patients: Possible roles of exercise training as a medicine. Curr Diabetes Rev. 10.2174/1573399817666210901121824
[2408] Lam S (2020) Psychological impact of COVID- 19 on People With Diabetes: Observations from Health Care Practitioners. Australian Diabetes Educator 23(4): 27-36
[2409] Landstra CP, de Koning EJP (2021) COVID-19 and Diabetes: Understanding the Interrelationship and Risks for a Severe Course. Front Endocrinol (Lausanne) 12: 649525. 10.3389/fendo.2021.649525
[2410] Lee T, Cau A, Cheng MP, et al. (2021) Angiotensin Receptor Blockers and Angiotensin-Converting Enzyme Inhibitors in COVID-19: Meta-analysis/Meta-regression Adjusted for Confounding Factors. CJC Open 3(7): 965-975. 10.1016/j.cjco.2021.03.001
[2411] Leippert C (2020) Diabetes unter Covid-19 in Gambia. Diabetes Aktuell 18(8): 312-313. 10.1055/a-1296-1144
[2412] Martorina WJ, Tavares A (2021) Possible role of exogenous melatonin in preventing more serious COVID-19 infection in patients with type 2 diabetes mellitus. Rev Assoc Med Bras (1992) 67Suppl 1(Suppl 1): 18-21. 10.1590/1806-9282.67.Suppl1.20200968
[2413] Nining L, Burhannudin I (2021) DIABETES MELITUS SEBAGAI FAKTOR RISIKO KEPARAHAN DAN KEMATIAN PASIEN COVID-19: META-ANALISIS. 13(1)
[2414] Puri A, He L, Giri M, Wu C, Zhao Q (2022) Comparison of comorbidities among severe and non-severe COVID-19 patients in Asian versus non-Asian populations: A systematic review and meta-analysis. Nurs Open 9(1): 733-751. 10.1002/nop2.1126
[2415] Ramphul K, Lohana P, Ramphul Y, et al. (2021) Hypertension, diabetes mellitus, and cerebrovascular disease predispose to a more severe outcome of COVID-19. Arch Med Sci Atheroscler Dis 6: e30-e39. 10.5114/amsad.2021.105255
[2416] Romil S, Sawai Singh R, Hira K, et al. (2021) Mortality and Severity in COVID-19 Patients on ACEIs & ARBs - A Meta-Regression Analysis. medRxiv. 10.1101/2021.03.14.21253557
[2417] Romil S, Sawai Singh R, Hira K, et al. (2021) Association of Obesity with COVID-19 Severity and Mortality: A Systemic Review and Meta-Regression. medRxiv. 10.1101/2021.05.08.21256845

[2418] Romiti GF, Corica B, Lip GYH, Proietti M (2021) Prevalence and Impact of Atrial Fibrillation in Hospitalized Patients with COVID-19: A Systematic Review and Meta-Analysis. J Clin Med 10(11). 10.3390/jcm10112490
[2419] Sahni S, Gupta G, Sarda R, Pandey S, Pandey RM, Sinha S (2021) Impact of metabolic and cardiovascular disease on COVID-19 mortality: A systematic review and meta-analysis. Diabetes Metab Syndr 15(6): 102308. 10.1016/j.dsx.2021.102308
[2420] Sattar N, Valabhji J (2021) Obesity as a Risk Factor for Severe COVID-19: Summary of the Best Evidence and Implications for Health Care. Curr Obes Rep 10(3): 282-289. 10.1007/s13679-021-00448-8
[2421] Shen Y, Cheng C, Zheng X, et al. (2021) Elevated Procalcitonin Is Positively Associated with the Severity of COVID-19: A Meta-Analysis Based on 10 Cohort Studies. Medicina (Kaunas) 57(6). 10.3390/medicina57060594
[2422] Singh R, Rathore SS, Khan H, et al. (2021) Mortality and Severity in COVID-19 Patients on ACEIs and ARBs-A Systematic Review, Meta-Analysis, and Meta-Regression Analysis. Front Med (Lausanne) 8: 703661. 10.3389/fmed.2021.703661
[2423] Stavroula K, Manisha P, Ben C, Mirela D, Phyo Kyaw M (2021) Impact of Diabetes on COVID-19 Mortality and Hospital Outcomes, a Global Perspective: An ONTOP Systematic Review and Meta-Analysis. SSRN
[2424] Surma S, Banach M, Lewek J (2021) COVID-19 and lipids. The role of lipid disorders and statin use in the prognosis of patients with SARS-CoV-2 infection. Lipids Health Dis 20(1): 141. 10.1186/s12944-021-01563-0
[2425] Tang SW, Leonard BE, Helmeste DM (2022) Long covid, neuropsychiatric disorders, psychotropics, present and future. Acta Neuropsychiatr: 1-43. 10.1017/neu.2022.6
[2426] Tsymbaliuk VI, Tronko MD, Antypkin YG, Kushnirenko SV, Popova VV (2021) Diabetes mellitus in combination with covid-19: Modern views on therapy. Reproductive Endocrinology(57): 8-20. 10.18370/2309-4117.2021.57.8-20
[2427] Tsymbaliuk VI, Tronko MD, Antypkin YG, Popova VV (2020) Diabetes mellitus and COVID-19: Current issues of pathogenesis, clinic and therapy: Literature review. Reproductive Endocrinology(54): 8-21. 10.18370/2309-4117.2020.54.8-21
[2428] Villamonte SB, Caro MKC, Cunanan EC (2021) Prevalence and clinical outcomes of patients with diabetic ketoacidosis/hyperglycemic hyperosmolar syndrome and COVID-19: A systematic review. Phillippine Journal of Internal Medicine 59(2): 101-106
[2429] Vitiello A, Ferrara F (2020) Anti-diabetic therapy in covid-19. Romanian Journal of Diabetes, Nutrition and Metabolic Diseases 27(2): 153-155. 10.46389/rjd-2020-1024
[2430] Wahyu W, Hotma R, Murtiningsih M, Yayat S, Hikmat R (2021) DIABETES MELITUS BECOME A HEALING DISEASE IN COVID-19 PATIENTS (SYSTEMATIC REVIEW). 12(2). 10.36089/NU.V12I2.334
[2431] World Health Organization ROfE (2021) Diabetes in Portugal in the time of the COVID-19 pandemic: reflecting on the year 2020. In:
[2432] Wulan Jasmin ALAAPK (2021) Risk factors for diabetes mellitus patients against COVID-19. Qanun Medika - Medical Journal Faculty of Medicine Muhammadiyah Surabaya 5(2): 163-176. 10.30651/jqm.v5i2.4999
[2433] Xie L, Zhang Z, Wang Q, Chen Y, Lu D, Wu W (2021) COVID-19 and Diabetes: A Comprehensive Review of Angiotensin Converting Enzyme 2, Mutual Effects and Pharmacotherapy. Front Endocrinol (Lausanne) 12: 772865. 10.3389/fendo.2021.772865
[2434] Александра Викторовна Х, Павел Константинович Б, Демьян Степанович П, Алексей Андреевич В (2021) Течение COVID-19 у лиц с сахарным диабетом и ожирением в кластере метаболического синдрома. 65(1). 10.25557/0031-2991.2021.01.102-106
[2435] Alamri W, Alhofaian A, Mersal N (2021) Quality of Life (QoL) among Health Care Workers with Diabetes Mellitus: A Literature Review. Clin Pract 11(4): 801-826. 10.3390/clinpract11040096
[2436] Simon A (2021) Typ-1-Diabetes: Alter und BMI als Risikofaktoren für schwere Covid-19-Verläufe. Diabetologie Und Stoffwechsel 16(5): 352-354. 10.1055/a-1503-8583
[2437] Mudatsir M, Fajar JK, Wulandari L, et al. (2021) Predictors of COVID-19 severity: a systematic review and meta-analysis [version 2;peer review: 2 approved]. In:
[2438] Zein A, Sulistiyana CS, Khasanah U, Wibowo A, Lim MA, Pranata R (2021) Statin and mortality in COVID-19: a systematic review and meta-analysis of pooled adjusted effect estimates from propensity-matched cohorts. Postgrad Med J. 10.1136/postgradmedj-2021-140409
[2439] Gao J, Gao Y, Zhang M, An Z, Wu Y, Zhang Q (2020) Factors Associated with Increased Risk of SARS-CoV-2 Infections in Diabetics. Chinese General Practice 23(35): 4436-4442. 10.12114/j.issn.1007-9572.2020.00.494
[2440] Kastora SPMCBDMMPK (2021) Impact of Diabetes on COVID-19 Mortality and Hospital Outcomes, a Global Perspective: An ONTOP Systematic Review and Meta-Analysis (preprint). In:

[2441] Vasileios Periklis P, Peny A, Stefania-Aspasia B, et al. (2021) Mortality of Diabetes-related Acute Metabolic Emergencies in COVID-19 patients: a systematic review and meta-analysis. In:
[2442] Perú. EsSalud. Instituto de Evaluación de Tecnologías en Salud e I (2020) Comorbilidades como factores de riesgo para mortalidad por COVID-19. In:
[2443] Lichert F (2021) Diabetes/COVID-19: Glykämische Schwellen für unerwünschte Outcomes definiert. Diabetologie Und Stoffwechsel 16(4): 263-263. 10.1055/a-1363-8285
[2444] Krome S (2021) Type 2 diabetes and COVID-19: A dangerous combination. Diabetologie Und Stoffwechsel 16(2): 86-87. 10.1055/a-1332-6372
[2445] Zein A, Raffaello WM (2022) Effect of colchicine on mortality in patients with COVID-19 - A systematic review and meta-analysis. Diabetes Metab Syndr 16(2): 102395. 10.1016/j.dsx.2022.102395
[2446] Shrestha DB, Budhathoki P, Raut S, et al. (2021) New-onset diabetes in COVID-19 and clinical outcomes: A systematic review and meta-analysis. World J Virol 10(5): 275-287. 10.5501/wjv.v10.i5.275
[2447] Nantes University H (2020) "COVID-19 and Diabetes Outcomes". In:
[2448] Afaque SY (2020) Association and treatment of diabetes in patients affected by COVID-19. International Journal of Research in Pharmaceutical Sciences 11(Special Issue 1): 1198-1201. 10.26452/ijrps.v11iSPL1.3591
[2449] Patel R (2021) Cannabinoids for pain and Covid risks of diabetes drugs: A summary of new research into the use of cannabinoids in chronic pain, and the relative risk of glucose-lowering drugs for Covid-19 mortality. Nursing in Practice: The Journal for Today's Primary Care Nurse(120): 8-8
[2450] Smati-Grangeon S, Tramunt B (2021) Prognostic factors of patients with diabetes and Covid-19: evidence from the CORONADO study. Correspondances En Metabolismes Hormones Diabetes Et Nutrition 25(2): 56-59
[2451] Luo M, Jiang B, Xu HJ, et al. (2020) Analysis of influencing factors of death in patients with COVID-19. Chinese Traditional and Herbal Drugs. 10.7501/j.issn.0253-2670.2020.06.010
[2452] Yang H, Yang LC, Zhang RT, Ling YP, Ge QG (2020) [Risks factors for death among COVID-19 patients combined with hypertension, coronary heart disease or diabetes]. Beijing da xue xue bao Yi xue ban = Journal of Peking University Health sciences 52(3): 420-424. 10.19723/j.issn.1671-167X.2020.03.004
[2453] Cernigliaro A, Allotta AV, Scondotto S (2020) [Can diabetes and its related hypoglycemic drug treatment be considered risk factors for health outcomes in COVID-19 patients? The results of a study in the population residing in Sicily Region (Southern Italy)]. Epidemiol Prev 44(5-6 Suppl 2): 315-322. 10.19191/ep20.5-6.S2.132
[2454] Shestakova MV, Vikulova OK, Isakov M, Dedov, II (2020) [Diabetes and COVID-19: analysis of the clinical outcomes according to the data of the russian diabetes registry]. Probl Endokrinol (Mosk) 66(1): 35-46. 10.14341/probl12458
[2455] Т. Ю Д, К. Г Л, С. Н П, М. Б А, О. Ш О (2021) Клинико-лабораторная характеристика пациентов с COVID-19 и сопутствующим сахарным диабетом 2 типа. 20(1)
[2456] Wargny M, Gourdy P, Ludwig L, et al. (2020) Type 1 Diabetes in People Hospitalized for COVID-19: New Insights From the CORONADO Study. Diabetes Care. 10.2337/dc20-1217
[2457] Zhang Y, Li H, Zhang J, et al. (2020) The Clinical Characteristics and Outcomes of Diabetes Mellitus and Secondary Hyperglycaemia Patients with Coronavirus Disease 2019: a Single-center, Retrospective, Observational Study in Wuhan. Diabetes obes metab
[2458] Cho KH, Kim SW, Park JW, Do JY, Kang SH (2020) Effect of Sex on Clinical Outcomes in Patients with Coronavirus Disease: A Population-Based Study. J Clin Med 10(1). 10.3390/jcm10010038
[2459] Noh Y, Oh IS, Jeong HE, Filion KB, Yu OHY, Shin JY (2021) Association Between DPP-4 Inhibitors and COVID-19-Related Outcomes Among Patients With Type 2 Diabetes. Diabetes Care 44(4): e64-e66. 10.2337/dc20-1824
[2460] Ramos-Rincón J, Pérez-Belmonte L, Carrasco-Sánchez F, et al. (2020) Association between prior cardiometabolic therapy and in-hospital mortality in very old patients with type 2 diabetes mellitus hospitalized due to COVID-19. A nationwide observational study in Spain. ResearchSquare. 10.21203/rs.3.rs-133358/v1
[2461] Chen L, Sun W, Liu Y, et al. (2021) Association of Early-Phase In-Hospital Glycemic Fluctuation With Mortality in Adult Patients With Coronavirus Disease 2019. Diabetes Care 44(4): 865-873. 10.2337/dc20-0780
[2462] Calvisi SL, Ramirez GA, Scavini M, et al. (2021) Thromboembolism risk among patients with diabetes/stress hyperglycemia and COVID-19. Metabolism 123: 154845. 10.1016/j.metabol.2021.154845
[2463] Wander PL, Lowy E, Beste LA, et al. (2021) Risk factors for adverse outcomes among 35 879 veterans with and without diabetes after diagnosis with COVID-19. BMJ Open Diabetes Res Care 9(1). 10.1136/bmjdrc-2021-002252

[2464] Lasbleiz A, Cariou B, Darmon P, et al. (2020) Phenotypic Characteristics and Development of a Hospitalization Prediction Risk Score for Outpatients with Diabetes and COVID-19: The DIABCOVID Study. J Clin Med 9(11). 10.3390/jcm9113726
[2465] Tchang BG, Askin G, Sahagun A, et al. (2021) The Independent Risk of Obesity and Diabetes and Their Interaction in COVID-19: A Retrospective Cohort Study. Obesity (Silver Spring) 29(6): 971-975. 10.1002/oby.23172
[2466] Abe T, Egbuche O, Igwe J, et al. (2021) Cardiovascular complications in COVID-19 patients with or without diabetes mellitus. Endocrinol Diabetes Metab 4(2): e00218. 10.1002/edm2.218
[2467] Acharya D, Lee K, Lee DS, Lee YS, Moon SS (2020) Mortality Rate and Predictors of Mortality in Hospitalized COVID-19 Patients with Diabetes. Healthcare (Basel) 8(3). 10.3390/healthcare8030338
[2468] Agarwal S, Schechter C, Southern W, Crandall JP, Tomer Y (2020) Preadmission Diabetes-Specific Risk Factors for Mortality in Hospitalized Patients With Diabetes and Coronavirus Disease 2019. Diabetes Care 43(10): 2339-2344. 10.2337/dc20-1543
[2469] Aghaaliakbari F, Abbasi MA, Ranjbar M, et al. (2020) Angiotensin Converting Enzyme Inhibitors, A Risk Factor of Poor Outcome in Diabetic Patients with COVID-19 Infection. Iran J Kidney Dis 14(6): 482-487
[2470] Ahmed FW, Kirresh OZ, Robinson AV, et al. (2021) A Retrospective Study Assessing the Effect of Diabetes on Mortality in Patients With COVID-19 at a Teaching Hospital in the United Kingdom. Cureus 13(3): e13902. 10.7759/cureus.13902
[2471] Alhakak A, Butt JH, Gerds TA, et al. (2022) Glycated haemoglobin levels among 3295 hospitalized COVID-19 patients, with and without diabetes, and risk of severe infection, admission to an intensive care unit and all-cause mortality. Diabetes Obes Metab 24(3): 499-510. 10.1111/dom.14604
[2472] Alrashed AA, Khan TM, Alhusseini NK, et al. (2021) Severity of COVID-19 infection in ACEI/ARB users in specialty hospitals: A retrospective cohort study. J Infect Public Health 14(6): 726-733. 10.1016/j.jiph.2021.03.004
[2473] Al Hayek AA, Robert AA, Matar AB, et al. (2020) Risk factors for hospital admission among COVID-19 patients with diabetes. A study from Saudi Arabia. Saudi Med J 41(10): 1090-1097. 10.15537/smj.2020.10.25419
[2474] Aon M, Alsaeedi A, Alzafiri A, et al. (2022) Stress Hyperglycemia Ratio as a Prognostic Marker in Diabetic Patients Hospitalized with COVID-19. Infect Dis Rep 14(5): 675-685. 10.3390/idr14050073
[2475] Assaad M, Hekmat-Joo N, Hosry J, et al. (2022) Insulin use in type II diabetic patients: a predictive of mortality in covid-19 infection. Diabetol Metab Syndr 14(1): 85. 10.1186/s13098-022-00857-2
[2476] Bello-Chavolla OY, Bahena-Lopez JP, Antonio-Villa NE, et al. (2020) Predicting Mortality Due to SARS-CoV-2: A Mechanistic Score Relating Obesity and Diabetes to COVID-19 Outcomes in Mexico. J Clin Endocrinol Metab 105(8). 10.1210/clinem/dgaa346
[2477] Barrett CE, Park J, Kompaniyets L, et al. (2021) Intensive Care Unit Admission, Mechanical Ventilation, and Mortality Among Patients With Type 1 Diabetes Hospitalized for COVID-19 in the U.S. Diabetes Care 44(8): 1788-1796. 10.2337/dc21-0604
[2478] Borzouei S, Mohammadian-Khoshnoud M, Omidi T, et al. (2021) Predictors of COVID-19 related death in diabetes patients: A case-control study in Iran. Diabetes Metab Syndr 15(4): 102149. 10.1016/j.dsx.2021.05.022
[2479] Boye KS, Tokar Erdemir E, Zimmerman N, et al. (2021) Risk Factors Associated with COVID-19 Hospitalization and Mortality: A Large Claims-Based Analysis Among People with Type 2 Diabetes Mellitus in the United States. Diabetes Ther 12(8): 2223-2239. 10.1007/s13300-021-01110-1
[2480] Calapod OP, Marin AM, Onisai M, Tribus LC, Pop CS, Fierbinteanu-Braticevici C (2021) The Impact of Increased Fib-4 Score in Patients with Type II Diabetes Mellitus on Covid-19 Disease Prognosis. Medicina (Kaunas) 57(5). 10.3390/medicina57050434
[2481] Cao P, Song Y, Zhuang Z, et al. (2021) Obesity and COVID-19 in Adult Patients With Diabetes. Diabetes 70(5): 1061-1069. 10.2337/db20-0671
[2482] Cariou B, Hadjadj S, Wargny M, et al. (2020) Phenotypic characteristics and prognosis of inpatients with COVID-19 and diabetes: the CORONADO study. Diabetologia 63(8): 1500-1515. 10.1007/s00125-020-05180-x
[2483] Cariou B, Goronflot T, Rimbert A, et al. (2020) Routine use of statins and increased mortality related to COVID-19 in inpatients with type 2 diabetes: Results from the CORONADO study. Diabetes Metab. 10.1016/j.diabet.2020.10.001
[2484] Chai C, Chen K, Li S, et al. (2022) Effect of elevated fasting blood glucose level on the 1-year mortality and sequelae in hospitalized COVID-19 patients: A bidirectional cohort study. J Med Virol 94(7): 3240-3250. 10.1002/jmv.27737
[2485] Charoenngam N, Alexanian SM, Apovian CM, Holick MF (2021) Association between Hyperglycemia at Hospital Presentation and Hospital Outcomes in COVID-19 Patients with and

without Type 2 Diabetes: A Retrospective Cohort Study of Hospitalized Inner-City COVID-19 Patients. Nutrients 13(7). 10.3390/nu13072199
[2486] Chen YC, Yang D, Cheng B, et al. (2020) Clinical Characteristics and Outcomes of Patients With Diabetes and COVID-19 in Association With Glucose-Lowering Medication. Diabetes Care 43(7): 1399-1407. 10.2337/dc20-0660
[2487] Chen X, Chen Y, Wu C, et al. (2020) Coagulopathy is a major extrapulmonary risk factor for mortality in hospitalized patients with COVID-19 with type 2 diabetes. BMJ Open Diabetes Res Care 8(2). 10.1136/bmjdrc-2020-001851
[2488] Chen J, Zhao C, Huang Y, et al. (2021) Malnutrition predicts poor outcomes in diabetic COVID-19 patients in Huangshi, Hubei. J Biomed Res 36(1): 32-38. 10.7555/JBR.35.20210083
[2489] Cheng Y, Yue L, Wang Z, Zhang J, Xiang G (2021) Hyperglycemia associated with lymphopenia and disease severity of COVID-19 in type 2 diabetes mellitus. J Diabetes Complications 35(2): 107809. 10.1016/j.jdiacomp.2020.107809
[2490] Choi HK, Koo H-J, Seok H, et al. (2020). 10.1101/2020.06.12.20129916
[2491] Chung SM, Lee YY, Ha E, et al. (2020) The Risk of Diabetes on Clinical Outcomes in Patients with Coronavirus Disease 2019: A Retrospective Cohort Study. Diabetes Metab J 44(3): 405-413. 10.4093/dmj.2020.0105
[2492] Corcillo A, Cohen S, Li A, Crane J, Kariyawasam D, Karalliedde J (2020) Diabetic retinopathy is independently associated with increased risk of intubation: A single centre cohort study of patients with diabetes hospitalised with COVID-19. Diabetes Res Clin Pract: 108529. 10.1016/j.diabres.2020.108529
[2493] Crouse AB, Grimes T, Li P, Might M, Ovalle F, Shalev A (2020) Metformin Use Is Associated With Reduced Mortality in a Diverse Population With COVID-19 and Diabetes. Front Endocrinol (Lausanne) 11: 600439. 10.3389/fendo.2020.600439
[2494] Dalan R, Ang LW, Tan WYT, et al. (2020) The association of hypertension and diabetes pharmacotherapy with COVID-19 severity and immune signatures: an observational study. Eur Heart J Cardiovasc Pharmacother
[2495] de Abajo FJ, Rodríguez-Martín S, Lerma V, et al. (2020) Use of renin-angiotensin-aldosterone system inhibitors and risk of COVID-19 requiring admission to hospital: a case-population study. Lancet 395(10238): 1705-1714
[2496] Demirci I, Haymana C, Tasci I, et al. (2022) Higher rate of COVID-19 mortality in patients with type 1 than type 2 diabetes: a nationwide study. Endokrynol Pol 73(1): 87-95. 10.5603/EP.a2022.0008
[2497] Souza SM, Quintao APC, Soares MCB, et al. (2022) Survival of patients with diabetes mellitus hospitalized for acute respiratory syndrome due to COVID-19. Rev Inst Med Trop Sao Paulo 64: e74. 10.1590/S1678-9946202264074
[2498] Djuric O, Ottone M, Vicentini M, et al. (2022) Diabetes and COVID-19 testing, positivity, and mortality: A population-wide study in Northern Italy. Diabetes Res Clin Pract 191: 110051. 10.1016/j.diabres.2022.110051
[2499] Damanti S, Ramirez GA, Bozzolo EP, et al. (2021) 6-Month Respiratory Outcomes and Exercise Capacity of COVID-19 Acute Respiratory Failure Patients Treated With CPAP. Intern Med J. 10.1111/imj.15345
[2500] Duan W, Li L, Li X, et al. (2022) Association of blood glucose level and prognosis of inpatients with coexistent diabetes and COVID-19. Endocrine 75(1): 1-9. 10.1007/s12020-021-02923-7
[2501] Elibol A, Eren D, Erdogan MD, et al. (2021) Factors influencing on development of COVID-19 pneumonia and association with oral anti-diabetic drugs in hospitalized patients with diabetes mellitus. Prim Care Diabetes 15(5): 806-812. 10.1016/j.pcd.2021.08.001
[2502] Emami A, Akbari A, Basirat A, et al. (2021) The role of comorbidities on mortality of COVID-19 in patients with diabetes. Obes Med 25: 100352. 10.1016/j.obmed.2021.100352
[2503] Emral R, Haymana C, Demirci I, et al. (2021) Lower COVID-19 Mortality in Patients with Type 2 Diabetes Mellitus Taking Dipeptidyl Peptidase-4 Inhibitors: Results from a Turkish Nationwide Study. Diabetes Ther 12(11): 2857-2870. 10.1007/s13300-021-01133-8
[2504] Erol RS, Sen EC, Ozturk FY, et al. (2022) Does DPP-4 inhibitor treatment affect the clinical outcomes of COVID-19 in type 2 diabetes mellitus patients? North Clin Istanb 9(3): 207-214. 10.14744/nci.2022.34341
[2505] Fernández-Pombo Aa, Santamar�a-Nieto A, Rodríguez-Carnero G, et al. (2000) Factores predictores de formas graves de COVID-19 que requieren hospitalización en pacientes con diabetes mellitus. Galicia Clínica 82(3). 10.22546/62/2561
[2506] Ferrannini G, Lund LH, Benson L, et al. (2022) Association between use of novel glucose-lowering drugs and COVID-19 hospitalization and death in patients with type 2 diabetes: a nationwide registry analysis. Eur Heart J Cardiovasc Pharmacother 9(1): 10-17. 10.1093/ehjcvp/pvac044
[2507] Fox T, Ruddiman K, Lo KB, et al. (2021) The relationship between diabetes and clinical outcomes in COVID-19: a single-center retrospective analysis. Acta Diabetol 58(1): 33-38. 10.1007/s00592-020-01592-8

[2508] Fu Y, Hu L, Ren HW, et al. (2022) Prognostic Factors for COVID-19 Hospitalized Patients with Preexisting Type 2 Diabetes. Int J Endocrinol 2022: 9322332. 10.1155/2022/9322332
[2509] Fukushima T, Chubachi S, Namkoong H, et al. (2023) Clinical significance of prediabetes, undiagnosed diabetes and diagnosed diabetes on critical outcomes in COVID-19: Integrative analysis from the Japan COVID-19 task force. Diabetes Obes Metab 25(1): 144-155. 10.1111/dom.14857
[2510] Ghany R, Palacio A, Dawkins E, et al. (2021) Metformin is associated with lower hospitalizations, mortality and severe coronavirus infection among elderly medicare minority patients in 8 states in USA. Diabetes Metab Syndr 15(2): 513-518. 10.1016/j.dsx.2021.02.022
[2511] Giorda CB, Picariello R, Tartaglino B, et al. (2021) From swab testing to health outcomes within the T2DM population: Impact of diabetes background on COVID19 progression. Diabetes Res Clin Pract 180: 109021. 10.1016/j.diabres.2021.109021
[2512] Gregory JM, Slaughter JC, Duffus SH, et al. (2021) COVID-19 Severity Is Tripled in the Diabetes Community: A Prospective Analysis of the Pandemic's Impact in Type 1 and Type 2 Diabetes. Diabetes Care 44(2): 526-532. 10.2337/dc20-2260
[2513] Hadjadj S, Saulnier PJ, Ruan Y, et al. (2023) Associations of microvascular complications with all-cause death in patients with diabetes and COVID-19: The CORONADO, ABCD COVID-19 UK national audit and AMERICADO study groups. Diabetes Obes Metab 25(1): 78-88. 10.1111/dom.14845
[2514] Hammad MO, Alseoudy MM (2021) The sex-related discrepancy in laboratory parameters of severe COVID-19 patients with diabetes: A retrospective cohort study. Prim Care Diabetes 15(4): 713-718. 10.1016/j.pcd.2021.05.002
[2515] Harris S, Ruan Y, Wild SH, et al. (2022) Association of statin and/or renin-angiotensin-aldosterone system modulating therapy with mortality in adults with diabetes admitted to hospital with COVID-19: A retrospective multicentre European study. Diabetes Metab Syndr 16(5): 102484. 10.1016/j.dsx.2022.102484
[2516] Heald AH, Jenkins DA, Williams R, et al. (2022) The Risk Factors Potentially Influencing Hospital Admission in People with Diabetes, Following SARS-CoV-2 Infection: A Population-Level Analysis. Diabetes Ther 13(5): 1007-1021. 10.1007/s13300-022-01230-2
[2517] Huang J, Zhu L, Bai X, et al. (2020) Multidimensional Analysis of Risk Factors for the Severity and Mortality of Patients with COVID-19 and Diabetes. Infect Dis Ther 9(4): 981-1002. 10.1007/s40121-020-00359-6
[2518] Hui Y, Li Y, Tong X, et al. (2020) The risk factors for mortality of diabetic patients with severe COVID-19: A retrospective study of 167 severe COVID-19 cases in Wuhan. PLoS One 15(12): e0243602. 10.1371/journal.pone.0243602
[2519] Ikram AS, Pillay S (2021) Hyperglycaemia, diabetes mellitus and COVID-19 in a tertiary hospital in KwaZulu-Natal. Journal of Endocrinology, Metabolism and Diabetes of South Africa 27(1): 32-41. 10.1080/16089677.2021.1997427
[2520] Iqbal A, Greig M, Arshad MF, Julian TH, Ee Tan S, Elliott J (2021) Higher admission activated partial thromboplastin time, neutrophil-lymphocyte ratio, serum sodium, and anticoagulant use predict in-hospital COVID-19 mortality in people with Diabetes: Findings from Two University Hospitals in the U.K. Diabetes Res Clin Pract 178: 108955. 10.1016/j.diabres.2021.108955
[2521] Izzi-Engbeaya C, Distaso W, Amin A, et al. (2021) Adverse outcomes in COVID-19 and diabetes: a retrospective cohort study from three London teaching hospitals. BMJ Open Diabetes Res Care 9(1). 10.1136/bmjdrc-2020-001858
[2522] Jayaswal SK, Singh S, Malik PS, et al. (2021) Detrimental effect of diabetes and hypertension on the severity and mortality of COVID-19 infection: A multi-center case-control study from India. Diabetes Metab Syndr 15(5): 102248. 10.1016/j.dsx.2021.102248
[2523] Kabootari M, Habibi Tirtashi R, Hasheminia M, et al. (2022) Clinical features, risk factors and a prediction model for in-hospital mortality among diabetic patients infected with COVID-19: data from a referral centre in Iran. Public Health 202: 84-92. 10.1016/j.puhe.2021.11.007
[2524] Kang IS, Kong KA (2021) Body mass index and severity/fatality from coronavirus disease 2019: A nationwide epidemiological study in Korea. PLoS One 16(6): e0253640. 10.1371/journal.pone.0253640
[2525] Khalili S, Moradi O, Kharazmi AB, Raoufi M, Sistanizad M, Shariat M (2021) Comparison of Mortality Rate and Severity of Pulmonary Involvement in Coronavirus Disease-2019 Adult Patients With and Without Type 2 Diabetes: A Cohort Study. Can J Diabetes 45(6): 524-530. 10.1016/j.jcjd.2020.10.014
[2526] Khalili S, Sabaghian T, Sedaghat M, Soroureddin Z, Askari E, Khalili N (2021) Prevalence, Risk Factors and Outcomes Associated with Acute Kidney Injury in Patients Hospitalized for COVID-19: A Comparative Study between Diabetic and Nondiabetic Patients. J Diabetes Res 2021: 6666086. 10.1155/2021/6666086

[2527] Khunti K, Ruan Y, Davies J, et al. (2022) Association Between SGLT2 Inhibitor Treatment and Diabetic Ketoacidosis and Mortality in People With Type 2 Diabetes Admitted to Hospital With COVID-19. Diabetes Care. 10.2337/dc22-0357
[2528] Kim MK, Jeon JH, Kim SW, et al. (2020) The Clinical Characteristics and Outcomes of Patients with Moderate-to-Severe Coronavirus Disease 2019 Infection and Diabetes in Daegu, South Korea. Diabetes Metab J 44(4): 602-613. 10.4093/dmj.2020.0146
[2529] Kristan MM, Kim YK, Nelson T, et al. (2021) Predictors of Severe COVID-19 in Patients With Diabetes: A Multicenter Review. Endocr Pract 27(8): 842-849. 10.1016/j.eprac.2021.05.011
[2530] Lalau JD, Al-Salameh A, Hadjadj S, et al. (2021) Metformin use is associated with a reduced risk of mortality in patients with diabetes hospitalised for COVID-19. Diabetes Metab 47(5): 101216. 10.1016/j.diabet.2020.101216
[2531] Lampasona V, Secchi M, Scavini M, et al. (2020) Antibody response to multiple antigens of SARS-CoV-2 in patients with diabetes: an observational cohort study. Diabetologia 63(12): 2548-2558. 10.1007/s00125-020-05284-4
[2532] Laurenzi A, Caretto A, Molinari C, et al. (2021) Pre-Existing Diabetes and COVID-Associated Hyperglycaemia in Patients with COVID-19 Pneumonia. Biology (Basel) 10(8). 10.3390/biology10080754
[2533] Lee HY, Ahn J, Park J, et al. (2021) Beneficial Effect of Statins in COVID-19-Related Outcomes-Brief Report: A National Population-Based Cohort Study. Arterioscler Thromb Vasc Biol 41(3): e175-e182. 10.1161/ATVBAHA.120.315551
[2534] Lei M, Lin K, Pi Y, et al. (2020) Clinical Features and Risk Factors of ICU Admission for COVID-19 Patients with Diabetes. J Diabetes Res 2020: 5237840. 10.1155/2020/5237840
[2535] Leon-Pedroza JI, Rodriguez-Cortes O, Flores-Mejia R, Gaona-Aguas CV, Gonzalez-Chavez A (2021) Impact of Metabolic Syndrome in the Clinical Outcome of Disease by SARS-COV-2. Arch Med Res 52(7): 738-745. 10.1016/j.arcmed.2021.04.001
[2536] Li YM, Han XY, Alwalid O, et al. (2020) Baseline characteristics and risk factors for short-term outcomes in 132 COVID-19 patients with diabetes in Wuhan China: A retrospective study. Diabetes Research and Clinical Practice 166. 10.1016/j.diabres.2020.108299
[2537] Li J, Wei Q, Li WX, et al. (2020) Metformin Use in Diabetes Prior to Hospitalization: Effects on Mortality in Covid-19. Endocr Pract 26(10): 1166-1172. 10.4158/EP-2020-0466
[2538] Liu ZL, Bai X, Han X, et al. (2020) The association of diabetes and the prognosis of COVID-19 patients: A retrospective study. Diabetes Research and Clinical Practice 169. 10.1016/j.diabres.2020.108386
[2539] Liu G, Zhang S, Hu H, Liu T, Huang J (2020) The role of neutrophil-lymphocyte ratio and lymphocyte-monocyte ratio in the prognosis of type 2 diabetics with COVID-19. Scott Med J 65(4): 154-160. 10.1177/0036933020953516
[2540] Llanera DK, Wilmington R, Shoo H, et al. (2021) Clinical Characteristics of COVID-19 Patients in a Regional Population With Diabetes Mellitus: The ACCREDIT Study. Front Endocrinol (Lausanne) 12: 777130. 10.3389/fendo.2021.777130
[2541] Llaurado G, Vlacho B, Wargny M, et al. (2022) The association between macrovascular complications and intensive care admission, invasive mechanical ventilation, and mortality in people with diabetes hospitalized for coronavirus disease-2019 (COVID-19). Cardiovasc Diabetol 21(1): 216. 10.1186/s12933-022-01657-8
[2542] Lombardi A, Agarwal S, Schechter C, Tomer Y (2022) In-Hospital Hyperglycemia Is Associated With Worse Outcomes in Patients Admitted With COVID-19. Diabetes Care 45(11): 2683-2688. 10.2337/dc22-0708
[2543] Longmore DK, Miller JE, Bekkering S, et al. (2021) Diabetes and Overweight/Obesity Are Independent, Nonadditive Risk Factors for In-Hospital Severity of COVID-19: An International, Multicenter Retrospective Meta-analysis. Diabetes Care 44(6): 1281-1290. 10.2337/dc20-2676
[2544] Lopez-Huamanrayme E, Garate-Chirinos DD, Espinoza-Morales F, et al. (2021) Association between hyperglycemia treatment and mortality in patients with diabetes and COVID-19 in a Peruvian hospital: A retrospective cohort study. J Clin Transl Endocrinol 26: 100265. 10.1016/j.jcte.2021.100265
[2545] Luk AOY, Yip TCF, Zhang X, et al. (2021) Glucose-lowering drugs and outcome from COVID-19 among patients with type 2 diabetes mellitus: a population-wide analysis in Hong Kong. BMJ Open 11(10): e052310. 10.1136/bmjopen-2021-052310
[2546] Ma Z, Patel N, Vemparala P, Krishnamurthy M (2022) Metformin is associated with favorable outcomes in patients with COVID-19 and type 2 diabetes mellitus. Sci Rep 12(1): 5553. 10.1038/s41598-022-09639-2
[2547] Madaschi S, Resmini E, Bonfadini S, et al. (2022) Predictive markers for clinical outcomes in a cohort of diabetic patients hospitalized for COVID-19. Diabetol Metab Syndr 14(1): 168. 10.1186/s13098-022-00941-7

[2548] Mannucci F, Vitturi G, Benacchio L, et al. (2022) Infection Rates and Impact of Glucose Lowering Medications on the Clinical Course of COVID-19 in People with Type 2 Diabetes: A Retrospective Observational Study. Diabetes Metab Syndr Obes 15: 3093-3101. 10.2147/DMSO.S385646
[2549] Marimuthu Y, Kunnavil R, Satyanarayana N, et al. (2022) Clinical presentation and mortality risk factors for COVID-19 among diabetic patients in a tertiary care center in South India. Indian J Tuberc 69(4): 496-502. 10.1016/j.ijtb.2021.08.015
[2550] Mehta PB, Kohn MA, Koliwad SK, Rushakoff RJ (2021) Lack of association between either outpatient or inpatient glycemic control and COVID-19 illness severity or mortality in patients with diabetes. BMJ Open Diabetes Res Care 9(1). 10.1136/bmjdrc-2021-002203
[2551] Meijer RI, Hoekstra T, van den Oever NCG, et al. (2021) Treatment with a DPP-4 inhibitor at time of hospital admission for COVID-19 is not associated with improved clinical outcomes: data from the COVID-PREDICT cohort study in The Netherlands. J Diabetes Metab Disord 20(2): 1155-1160. 10.1007/s40200-021-00833-z
[2552] Merzon E, Green I, Shpigelman M, et al. (2020) Haemoglobin A1c is a predictor of COVID-19 severity in patients with diabetes. Diabetes Metab Res Rev: e3398. 10.1002/dmrr.3398
[2553] de Miguel-Yanes JM, Jimenez-Garcia R, de Miguel-Diez J, et al. (2022) Impact of Type 2 Diabetes Mellitus on the Incidence and Outcomes of COVID-19 Needing Hospital Admission According to Sex: Retrospective Cohort Study Using Hospital Discharge Data in Spain, Year 2020. J Clin Med 11(9). 10.3390/jcm11092654
[2554] Mirani M, Favacchio G, Carrone F, et al. (2020) Impact of Comorbidities and Glycemia at Admission and Dipeptidyl Peptidase 4 Inhibitors in Patients With Type 2 Diabetes With COVID-19: A Case Series From an Academic Hospital in Lombardy, Italy. Diabetes Care 43(12): 3042-3049. 10.2337/dc20-1340
[2555] Mohamed M, Gouda TM, Hanafy AS, et al. (2021) Clinical, Laboratory and Radiological Predictors of Unfavorable Hospital Admission Course for Diabetic Patients with COVID-19. The Egyptian Journal of Hospital Medicine 83(1): 1321-1329. 10.21608/ejhm.2021.165528
[2556] Mondal S, DasGupta R, Lodh M, et al. (2021) Predictors of new-onset diabetic ketoacidosis in patients with moderate to severe COVID-19 receiving parenteral glucocorticoids: A prospective single-centre study among Indian type 2 diabetes patients. Diabetes Metab Syndr 15(3): 795-801. 10.1016/j.dsx.2021.03.022
[2557] Mondal S, DasGupta R, Lodh M, et al. (2022) Stress hyperglycemia ratio, rather than admission blood glucose, predicts in-hospital mortality and adverse outcomes in moderate-to severe COVID-19 patients, irrespective of pre-existing glycemic status. Diabetes Res Clin Pract 190: 109974. 10.1016/j.diabres.2022.109974
[2558] Morse J, Gay W, Korwek KM, et al. (2021) Hyperglycaemia increases mortality risk in non-diabetic patients with COVID-19 even more than in diabetic patients. Endocrinol Diabetes Metab 4(4): e00291. 10.1002/edm2.291
[2559] Myers AK, Kim TS, Zhu X, Liu Y, Qiu M, Pekmezaris R (2021) Predictors of mortality in a multiracial urban cohort of persons with type 2 diabetes and novel coronavirus 19. J Diabetes. 10.1111/1753-0407.13158
[2560] Nikniaz Z, Somi MH, Dinevari MF, Taghizadieh A, Mokhtari L (2021) Diabesity Associates with Poor COVID-19 Outcomes among Hospitalized Patients. J Obes Metab Syndr 30(2): 149-154. 10.7570/jomes20121
[2561] Numaguchi R, Kurajoh M, Hiura Y, et al. (2022) Glycated hemoglobin level on admission associated with progression to severe disease in hospitalized patients with non-severe coronavirus disease 2019. J Diabetes Investig 13(10): 1779-1787. 10.1111/jdi.13845
[2562] Nyland JE, Nazia TR-K, Kerstin B, et al. (2020) Diabetes, Drug Treatment and Mortality in COVID-19: A Multinational Retrospective Cohort Study. SSRN. 10.2139/ssrn.3725612
[2563] Oh TK, Song IA (2021) Metformin use and risk of COVID-19 among patients with type II diabetes mellitus: an NHIS-COVID-19 database cohort study. Acta Diabetol 58(6): 771-778. 10.1007/s00592-020-01666-7
[2564] Ojeda-Fernandez L, Foresta A, Macaluso G, et al. (2022) Metformin use is associated with a decrease in the risk of hospitalization and mortality in COVID-19 patients with diabetes: A population-based study in Lombardy. Diabetes Obes Metab 24(5): 891-898. 10.1111/dom.14648
[2565] O'Malley G, Ebekozien O, Desimone M, et al. (2020) COVID-19 Hospitalization in Adults with Type 1 Diabetes: Results from the T1D Exchange Multi-Center Surveillance Study. J Clin Endocrinol Metab. 10.1210/clinem/dgaa825
[2566] Orioli L, Servais T, Belkhir L, et al. (2021) Clinical characteristics and short-term prognosis of in-patients with diabetes and COVID-19: A retrospective study from an academic center in Belgium. Diabetes Metab Syndr 15(1): 149-157. 10.1016/j.dsx.2020.12.020

[2567] Ortega E, Corcoy R, Gratacos M, et al. (2021) Risk factors for severe outcomes in people with diabetes hospitalised for COVID-19: a cross-sectional database study. BMJ Open 11(7): e051237. 10.1136/bmjopen-2021-051237
[2568] Ouchi D, Vilaplana-Carnerero C, de Dios V, Giner-Soriano M, Morros R (2022) Antidiabetic treatment and COVID-19 Outcomes: A population-based cohort study in primary health care in Catalonia during the first wave of the pandemic. Prim Care Diabetes 16(6): 753-759. 10.1016/j.pcd.2022.10.001
[2569] Palazzuoli A, Mancone M, De Ferrari GM, et al. (2020) Antecedent Administration of Angiotensin-Converting Enzyme Inhibitors or Angiotensin II Receptor Antagonists and Survival After Hospitalization for COVID-19 Syndrome. J Am Heart Assoc 9(22): e017364. 10.1161/JAHA.120.017364
[2570] Patel AJ, Klek SP, Peragallo-Dittko V, et al. (2021) Correlation of Hemoglobin A1C and Outcomes in Patients Hospitalized With COVID-19. Endocr Pract 27(10): 1046-1051. 10.1016/j.eprac.2021.07.008
[2571] Pazoki M, Keykhaei M, Kafan S, et al. (2021) Risk indicators associated with in-hospital mortality and severity in patients with diabetes mellitus and confirmed or clinically suspected COVID-19. J Diabetes Metab Disord: 1-11. 10.1007/s40200-020-00701-2
[2572] Pazoki M, Chichagi F, Hadadi A, et al. (2021) Association of clinical characteristics, antidiabetic and cardiovascular agents with diabetes mellitus and COVID-19: a 7-month follow-up cohort study. J Diabetes Metab Disord 20(2): 1545-1555. 10.1007/s40200-021-00901-4
[2573] Perez-Belmonte LM, Torres-Pena JD, Lopez-Carmona MD, et al. (2020) Mortality and other adverse outcomes in patients with type 2 diabetes mellitus admitted for COVID-19 in association with glucose-lowering drugs: a nationwide cohort study. BMC Med 18(1): 359. 10.1186/s12916-020-01832-2
[2574] Petrakis V, Panagopoulos P, Trypsianis G, Papazoglou D, Papanas N (2021) Glucose on Admission: Unfavourable Effects on Hospitalisation and Outcomes in Type 2 Diabetes Mellitus Patients with COVID-19 Pneumonia. Exp Clin Endocrinol Diabetes. 10.1055/a-1686-8738
[2575] Pettrone K, Burnett E, Link-Gelles R, et al. (2021) Characteristics and Risk Factors of Hospitalized and Nonhospitalized COVID-19 Patients, Atlanta, Georgia, USA, March-April 2020. Emerg Infect Dis 27(4): 1164-1168. 10.3201/eid2704.204709
[2576] Phan F, Boussouar S, Lucidarme O, et al. (2021) Cardiac adipose tissue volume and IL-6 level at admission are complementary predictors of severity and short-term mortality in COVID-19 diabetic patients. Cardiovasc Diabetol 20(1): 165. 10.1186/s12933-021-01327-1
[2577] Pulido-Perez P, Pondigo-de Los Angeles JA, Hernandez-Hernandez ME, Torres-Rasgado E, Romero JR (2021) Renal function, serum magnesium levels and mortality in COVID-19 patients with type 2 diabetes. Magnes Res 34(1): 20-31. 10.1684/mrh.2021.0481
[2578] Ramesh J, Rajesh M, Varghese J, Reddy SLS (2021) Calculated plasma osmolality at hospital admission correlates well with eGFR and D-Dimer, a simple outcome predictor and guiding tool for management of severe COVID-19 patients. Diabetes Metab Syndr 15(5): 102240. 10.1016/j.dsx.2021.102240
[2579] Ramos-Rincón JM, Pérez-Belmonte LM, Carrasco-Sánchez FJ, et al. (2021) Cardiometabolic therapy and mortality in very old patients with diabetes hospitalized due to COVID-19. J Gerontol A Biol Sci Med Sci. 10.1093/gerona/glab124
[2580] Rastad H, Karim H, Ejtahed HS, et al. (2020) Risk and predictors of in-hospital mortality from COVID-19 in patients with diabetes and cardiovascular disease. Diabetology & Metabolic Syndrome 12(1). 10.1186/s13098-020-00565-9
[2581] Rastad H, Ejtahed HS, Mahdavi-Ghorabi A, et al. (2020) Factors associated with the poor outcomes in diabetic patients with COVID-19. J Diabetes Metab Disord: 1-10. 10.1007/s40200-020-00646-6
[2582] Rezaei N, Montazeri F, Malekpour MR, et al. (2021) COVID-19 in patients with diabetes: factors associated with worse outcomes. J Diabetes Metab Disord 20(2): 1605-1614. 10.1007/s40200-021-00910-3
[2583] Rhee SY, Lee J, Nam H, Kyoung DS, Shin DW, Kim DJ (2021) Effects of a DPP-4 Inhibitor and RAS Blockade on Clinical Outcomes of Patients with Diabetes and COVID-19. Diabetes Metab J 45(2): 251-259. 10.4093/dmj.2020.0206
[2584] Riahi S, Sombra LRS, Lo KB, et al. (2021) Insulin Use, Diabetes Control, and Outcomes in Patients with COVID-19. Endocr Res 46(2): 45-50. 10.1080/07435800.2020.1856865
[2585] Roussel R, Darmon P, Pichelin M, et al. (2021) Use of dipeptidyl peptidase-4 inhibitors and prognosis of COVID-19 in hospitalized patients with type 2 diabetes: A propensity score analysis from the CORONADO study. Diabetes Obes Metab 23(5): 1162-1172. 10.1111/dom.14324
[2586] Ruan Y, Ryder REJ, De P, et al. (2021) A UK nationwide study of people with type 1 diabetes admitted to hospital with COVID-19 infection. Diabetologia 64(8): 1717-1724. 10.1007/s00125-021-05463-x

[2587] Sadidi M, Zare A, Nasrollahzadehsabet M, Dastan F, Mosadegh Khah A, Jafari Asheyani M (2022) The roles of dipeptidyl peptidase-4 inhibitors in prognosis of COVID-19 infection in patients with type 2 diabetes mellitus. J Res Med Sci 27: 62. 10.4103/jrms.jrms_71_22
[2588] Sarigumba M, Aragon J, Kanapi MP (2021) Baseline Glycemic Status and Outcome of Persons with Type 2 Diabetes with COVID-19 Infections: A Single-Center Retrospective Study. J ASEAN Fed Endocr Soc 36(1): 45-49. 10.15605/jafes.036.01.06
[2589] Satman I, Demirci I, Haymana C, et al. (2021) Unexpectedly lower mortality rates in COVID-19 patients with and without type 2 diabetes in Istanbul. Diabetes Res Clin Pract 174: 108753. 10.1016/j.diabres.2021.108753
[2590] Savarese G, Benson L, Sundstrom J, Lund LH (2021) Association between renin-angiotensin-aldosterone system inhibitor use and COVID-19 hospitalization and death: a 1.4 million patient nationwide registry analysis. Eur J Heart Fail 23(3): 476-485. 10.1002/ejhf.2060
[2591] Saygili ES, Karakilic E, Mert E, Sener A, Mirci A (2022) Preadmission usage of metformin and mortality in COVID-19 patients including the post-discharge period. Ir J Med Sci 191(2): 569-575. 10.1007/s11845-021-02823-9
[2592] Seiglie J, Platt J, Cromer SJ, et al. (2020) Diabetes as a Risk Factor for Poor Early Outcomes in Patients Hospitalized With COVID-19. Diabetes Care 43(12): 2938-2944. 10.2337/dc20-1506
[2593] Shah P, Owens J, Franklin J, Jani Y, Kumar A, Doshi R (2020) Baseline use of angiotensin-converting enzyme inhibitor/AT1 blocker and outcomes in hospitalized coronavirus disease 2019 African-American patients. J Hypertens 38(12): 2537-2541. 10.1097/HJH.0000000000002584
[2594] Shang J, Wang Q, Zhang H, et al. (2021) The Relationship Between Diabetes Mellitus and COVID-19 Prognosis: A Retrospective Cohort Study in Wuhan, China. Am J Med 134(1): e6-e14. 10.1016/j.amjmed.2020.05.033
[2595] Shang R, Liu H, Ni J (2021) Evaluation of prognostic indicators for COVID-19 patients with diabetes. Medical Journal of Wuhan University
[2596] Shauly-Aharonov M, Shafrir A, Paltiel O, et al. (2021) Both high and low pre-infection glucose levels associated with increased risk for severe COVID-19: New insights from a population-based study. PLoS One 16(7): e0254847. 10.1371/journal.pone.0254847
[2597] Shestakova MV, Vikulova OK, Elfimova AR, Deviatkin AA, Dedov, II, Mokrysheva NG (2022) Risk factors for COVID-19 case fatality rate in people with type 1 and type 2 diabetes mellitus: A nationwide retrospective cohort study of 235,248 patients in the Russian Federation. Front Endocrinol (Lausanne) 13: 909874. 10.3389/fendo.2022.909874
[2598] Iqbal Z, Ho JH, Adam S, et al. (2020) Managing hyperlipidaemia in patients with COVID-19 and during its pandemic: An expert panel position statement from HEART UK. Atherosclerosis 313: 126-136. 10.1016/j.atherosclerosis.2020.09.008
[2599] Shukla AP, Tchang BG, Lam T, et al. (2021) Preadmission predictors of severe COVID-19 in patients with diabetes mellitus. J Diabetes Complications 35(8): 107967. 10.1016/j.jdiacomp.2021.107967
[2600] Silverii GA, Monami M, Cernigliaro A, et al. (2021) Are diabetes and its medications risk factors for the development of COVID-19? Data from a population-based study in Sicily. Nutr Metab Cardiovasc Dis 31(2): 396-398. 10.1016/j.numecd.2020.09.028
[2601] Smati S, Tramunt B, Wargny M, et al. (2021) Relationship between obesity and severe COVID-19 outcomes in patients with type 2 diabetes: Results from the CORONADO study. Diabetes Obes Metab 23(2): 391-403. 10.1111/dom.14228
[2602] Solerte SB, D'Addio F, Trevisan R, et al. (2020) Sitagliptin Treatment at the Time of Hospitalization Was Associated With Reduced Mortality in Patients With Type 2 Diabetes and COVID-19: A Multicenter, Case-Control, Retrospective, Observational Study. Diabetes Care 43(12): 2999-3006. 10.2337/dc20-1521
[2603] Soliman AR, Abdelaziz TS, Fathy A (2021) Impact of Vitamin D Therapy on the Progress COVID-19: Six Weeks Follow-Up Study of Vitamin D Deficient Elderly Diabetes Patients. Proceedings of Singapore Healthcare. 10.1177/20101058211041405
[2604] Sonmez A, Demirci I, Haymana C, et al. (2021) Clinical characteristics and outcomes of COVID-19 in patients with type 2 diabetes in Turkey: A nationwide study (TurCoviDia). J Diabetes 13(7): 585-595. 10.1111/1753-0407.13171
[2605] Stevens JS, Bogun MM, McMahon DJ, et al. (2021) Diabetic ketoacidosis and mortality in COVID-19 infection. Diabetes Metab 47(6): 101267. 10.1016/j.diabet.2021.101267
[2606] Strollo R, Maddaloni E, Dauriz M, Pedone C, Buzzetti R, Pozzilli P (2021) Use of DPP4 inhibitors in Italy does not correlate with diabetes prevalence among COVID-19 deaths. Diabetes Res Clin Pract 171: 108444. 10.1016/j.diabres.2020.108444
[2607] Tallon EM, Ebekozien O, Sanchez J, et al. (2022) Impact of diabetes status and related factors on COVID-19-associated hospitalization: A nationwide retrospective cohort study of 116,370 adults with SARS-CoV-2 infection. Diabetes Res Clin Pract 194: 110156. 10.1016/j.diabres.2022.110156

[2608] Tamura RE, Said SM, de Freitas LM, Rubio IGS (2021) Outcome and death risk of diabetes patients with Covid-19 receiving pre-hospital and in-hospital metformin therapies. Diabetol Metab Syndr 13(1): 76. 10.1186/s13098-021-00695-8
[2609] Tian Z, Heald AH, Stedman M, et al. (2021) Age of people with type 2 diabetes and the risk of dying following SARS-CoV-2 infection. Int J Clin Pract 75(8): e14053. 10.1111/ijcp.14053
[2610] Tramunt B, Smati S, Coudol S, et al. (2021) Sex disparities in COVID-19 outcomes of inpatients with diabetes: insights from the CORONADO study. Eur J Endocrinol 185(2): 299-311. 10.1530/EJE-21-0068
[2611] Tuan WJ, Lennon RP, Zhang A, Macherla A, Zgierska AE (2022) Risks of Severe COVID-19 Outcomes Among Patients With Diabetic Polyneuropathy in the United States. J Public Health Manag Pract 28(6): 674-681. 10.1097/PHH.0000000000001587
[2612] Valle A, Rodriguez J, Camina F, Martinez-Olmos MA, Ortola JB, Rodriguez-Segade S (2022) At-admission HbA(1c) levels in hospitalized COVID-19 participants with and without known diabetes. Clin Chim Acta 532: 188-192. 10.1016/j.cca.2022.05.027
[2613] Vargas-Vazquez A, Bello-Chavolla OY, Ortiz-Brizuela E, et al. (2021) Impact of undiagnosed type 2 diabetes and pre-diabetes on severity and mortality for SARS-CoV-2 infection. BMJ Open Diabetes Res Care 9(1). 10.1136/bmjdrc-2020-002026
[2614] Vasbinder A, Anderson E, Shadid H, et al. (2022) Inflammation, Hyperglycemia, and Adverse Outcomes in Individuals With Diabetes Mellitus Hospitalized for COVID-19. Diabetes Care 45(3): 692-700. 10.2337/dc21-2102
[2615] Wander PL, Lowy E, Beste LA, et al. (2021) Prior Glucose-Lowering Medication Use and 30-Day Outcomes Among 64,892 Veterans With Diabetes and COVID-19. Diabetes Care 44(12): 2708-2713. 10.2337/dc21-1351
[2616] Wang X, Liu Z, Li J, et al. (2020) Impacts of Type 2 Diabetes on Disease Severity, Therapeutic Effect, and Mortality of Patients With COVID-19. J Clin Endocrinol Metab 105(12). 10.1210/clinem/dgaa535
[2617] Wang B, Glicksberg BS, Nadkarni GN, Vashishth D (2021) Evaluation and management of COVID-19-related severity in people with type 2 diabetes. BMJ Open Diabetes Res Care 9(1). 10.1136/bmjdrc-2021-002299
[2618] Wang Y, Zhang J, Li H, et al. (2021) Prognostic Value of Leucocyte to High-Density Lipoprotein-Cholesterol Ratios in COVID-19 Patients and the Diabetes Subgroup. Front Endocrinol (Lausanne) 12: 727419. 10.3389/fendo.2021.727419
[2619] Wargny M, Potier L, Gourdy P, et al. (2021) Predictors of hospital discharge and mortality in patients with diabetes and COVID-19: updated results from the nationwide CORONADO study. Diabetologia 64(4): 778-794. 10.1007/s00125-020-05351-w
[2620] Wong CKH, Lui DTW, Lui AYC, et al. (2022) Use of DPP4i reduced odds of clinical deterioration and hyperinflammatory syndrome in COVID-19 patients with type 2 diabetes: Propensity score analysis of a territory-wide cohort in Hong Kong. Diabetes Metab 48(1): 101307. 10.1016/j.diabet.2021.101307
[2621] Wong R, Hall M, Vaddavalli R, et al. (2022) Glycemic Control and Clinical Outcomes in U.S. Patients With COVID-19: Data From the National COVID Cohort Collaborative (N3C) Database. Diabetes Care 45(5): 1099-1106. 10.2337/dc21-2186
[2622] Wong CKH, Lui DTW, Lui AYC, et al. (2022) Metformin Use in Relation to Clinical Outcomes and Hyperinflammatory Syndrome Among COVID-19 Patients With Type 2 Diabetes: A Propensity Score Analysis of a Territory-Wide Cohort. Front Endocrinol (Lausanne) 13: 810914. 10.3389/fendo.2022.810914
[2623] Wu B, Zhou JH, Wang WX, et al. (2021) Association Analysis of Hyperlipidemia with the 28-Day All-Cause Mortality of COVID-19 in Hospitalized Patients. Chin Med Sci J 36(1): 17-26. 10.24920/003866
[2624] Xiao YF, He JL, Xu Y, et al. (2021) Major Characteristics of Severity and Mortality in Diabetic Patients With COVID-19 and Establishment of Severity Risk Score. Front Med (Lausanne) 8: 655604. 10.3389/fmed.2021.655604
[2625] Xu Z, Wang Z, Wang S, et al. (2020) The impact of type 2 diabetes and its management on the prognosis of patients with severe COVID-19. J Diabetes 12(12): 909-918. 10.1111/1753-0407.13084
[2626] Yan H, Valdes AM, Vijay A, et al. (2020) Role of Drugs Used for Chronic Disease Management on Susceptibility and Severity of COVID-19: A Large Case-Control Study. Clin Pharmacol Ther 108(6): 1185-1194. 10.1002/cpt.2047
[2627] Yeh HC, Kraschnewski JL, Kong L, et al. (2022) Hospitalization and mortality in patients with COVID-19 with or at risk of type 2 diabetes: data from five health systems in Pennsylvania and Maryland. BMJ Open Diabetes Res Care 10(3). 10.1136/bmjdrc-2022-002774

[2628] Yoo J, Choi Y, Park SA, Seo JY, Ahn CW, Han J (2022) Glycated Albumin and Glycated Albumin/HbA1c Predict the Progression of Coronavirus Disease 2019 from Mild to Severe Disease in Korean Patients with Type 2 Diabetes. J Clin Med 11(9). 10.3390/jcm11092327
[2629] You JH, Lee SA, Chun SY, et al. (2020) Clinical Outcomes of COVID-19 Patients with Type 2 Diabetes: A Population-Based Study in Korea. Endocrinol Metab (Seoul) 35(4): 901-908. 10.3803/EnM.2020.787
[2630] Zeltyn-Abramov EM, Lysenko MA, Frolova NF, et al. (2021) Risk factors of adverse outcome of COVID-19 and experience of Tocilizumab administration in patients on maintenance hemodialysis due to diabetic kidney disease. Diabetes mellitus 24(1): 17-31. 10.14341/dm12688
[2631] Zhan K, Zhang X, Wang B, et al. (2022) Short- and long-term prognosis of glycemic control in COVID-19 patients with type 2 diabetes. QJM 115(3): 131-139. 10.1093/qjmed/hcac020
[2632] Zhang Q, Wei Y, Chen M, Wan Q, Chen X (2020) Clinical analysis of risk factors for severe COVID-19 patients with type 2 diabetes. J Diabetes Complications 34(10): 107666. 10.1016/j.jdiacomp.2020.107666
[2633] Zhang N, Wang C, Zhu F, et al. (2020) Risk Factors for Poor Outcomes of Diabetes Patients With COVID-19: A Single-Center, Retrospective Study in Early Outbreak in China. Front Endocrinol (Lausanne) 11: 571037. 10.3389/fendo.2020.571037
[2634] Zhu L, She ZG, Cheng X, et al. (2020) Association of Blood Glucose Control and Outcomes in Patients with COVID-19 and Pre-existing Type 2 Diabetes. Cell Metab 31(6): 1068-1077 e1063. 10.1016/j.cmet.2020.04.021
[2635] McGuinness LA, Higgins JPT (2020) Risk-of-bias VISualization (robvis): An R package and Shiny web app for visualizing risk-of-bias assessments. Res Synth Methods. 10.1002/jrsm.1411
